# Anomeric Amide Enabled Divergent Synthesis of Unsymmetrical Ureas, Carbamates, Thioesters, and Amides From Aldehydes

**DOI:** 10.1002/anie.9599258

**Published:** 2026-07-08

**Authors:** Jasper L. Tyler, Meiyi Zeng, Julius Wiener, Marie‐Christin Leusmann, Colin Stein, Constantin G. Daniliuc, Frank Glorius

**Affiliations:** ^1^ Organisch‐Chemisches Institut Universität Münster Münster Germany

**Keywords:** aldehydes, anomeric amides, divergent synthesis, hydroxamates, Lossen rearrangement

## Abstract

Divergent synthetic transformations that convert a single precursor into a range of structurally distinct products are powerful tools for rapidly exploring chemical space. Although numerous strategies exist for converting versatile functional handles such as halides and boronic acids into a multitude of reactive intermediates, there remains a pressing need for methodologies that exploit alternative linchpin fragments to open complementary avenues to molecular complexity. Herein, we report a divergent, anomeric amide‐enabled, aldehyde functionalization strategy, allowing access to unsymmetrical ureas, carbamates, thiocarbamates, thioesters, amines, or amides, all in a one‐pot procedure. Unique to this transformation is the formation of *N*‐Boc‐hydroxamate intermediates, which serve as privileged platforms for orthogonal activation via Lossen‐type rearrangements, single‐electron transfer, or nucleophilic substitution, generating a diverse selection of reactive intermediates. Overall, this work establishes *N*‐halo‐*O*‐activated hydroxycarbamate‐type anomeric amides as valuable reagents for aldehyde diversification, offering a complementary approach to molecular complexity generation from feedstock compounds.

## Graphical abstract

An anomeric amide‐enabled divergent aldehyde functionalization sequence provides one‐pot access to unsymmetrical ureas, carbamates, thiocarbamates, thioesters, and amides. Key to this transformation is the formation of *N*‐Boc‐hydroxamate intermediates, which serve as platforms for orthogonal activation via Lossen‐type rearrangements, single‐electron transfer, or nucleophilic substitution, generating a diverse selection of reactive intermediates.

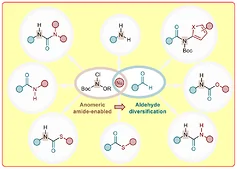

## 1 Introduction

**FIGURE 1:**
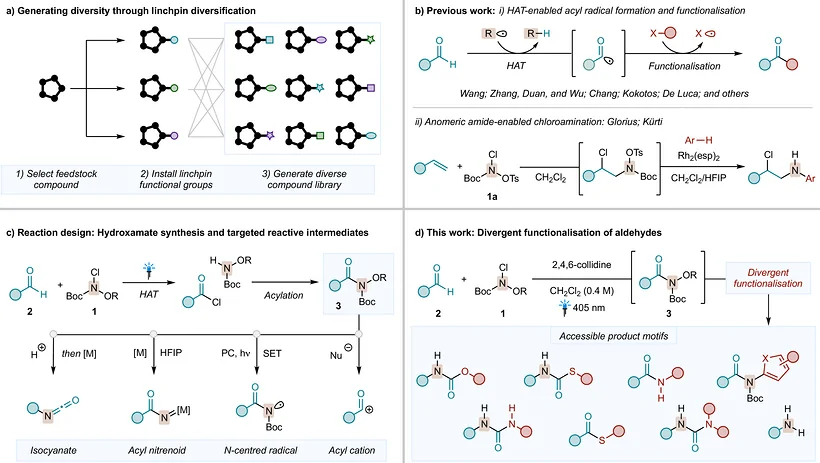
Diversity‐generating synthesis and application of aldehydes to divergent functionalization. a) Generating diversity through linchpin diversification. b) Previous work. c) Reaction design: Hydroxamate synthesis and targeted reactive intermediates. d) This work: Divergent functionalization of aldehydes.

The ability to generate molecular diversity efficiently from simple, readily available precursors is a key objective in contemporary synthetic chemistry [1, 2, 3]. This capability is particularly valuable in discovery‐driven research, where diverse compound collections increase the likelihood of identifying new biologically active motifs [4, 5, 6]. In practice, syntheses that target divergent compound generation are often built around harnessing the multifaceted reactivity of linchpin functional handles such as carboxylic acids [7, 8], halides [9, 10, 11], and boronic acids (Figure 1a) [12, 13, 14]. Accordingly, the synthetic methods concerned with the elaboration of these groups have reached a level of sophistication unmatched in the field of constructing small organic molecules. However, the reliance on specific sets of well‐established reaction protocols for the diversification of these privileged functionalities can lead to uneven exploration across certain regions of chemical space [15, 16]. Therefore, further methods that can access multiple distinct classes of species from alternative but readily available functional groups, such as aldehydes [17, 18] (and their redox‐related alcohols), can provide a complementary strategy for the rapid generation of molecular complexity from a single class of building block.

When conceptualizing a transformation in which aldehydes are employed as a highly versatile functional handle for diversity generation, we were inspired by previous reports of hydrogen atom transfer (HAT) enabled acyl radical formation and subsequent functionalization of aldehydes (Figure 1bi) [19]. For example, studies from Wang [20], as well as those from Zhang, Duan, and Wu [17], along with others [21, 22], have demonstrated that aldehyde derivatization can be achieved via the photocatalytic generation of acyl radicals, followed by interception of this intermediate with a radical trap. The synthesis of alternative product moieties using related systems have also been demonstrated by the groups of Chang [23, 24], Kokotos [25], and De Luca [26], where HAT‐induced radical chlorination of aldehydes provides acyl chloride intermediates that can be subsequently reacted with a variety of external nucleophiles. These prior reports demonstrate that divergent aldehyde functionalization has been established. However, while these transformations are elegant, the intermediates generated are limited to functionalization via a singular reaction mode, restricting the ability to generate diverse compound libraries (see Supporting Information for a detailed comparison of previous methods to our work). Building on these precedents, we envisioned that this general approach could be adapted to provide an operationally simple method to convert aldehydes to a linchpin intermediate that, when engaged in coupling under a range of orthogonal conditions, would facilitate access to numerous classes of compounds.

Our group and that of Kürti have previously shown that *N*‐halo‐*O*‐activated hydroxycarbamates, such as **1a**, enable exceptionally mild, catalyst‐free alkene chloroamination via N−Cl homolytic cleavage followed by radical addition to the olefin (Figure 1bii) [27, 28]. The resulting intermediates have then been demonstrated to act as (formal) nitrene precursors that can be subsequently functionalized in a variety of ways, such as arene C−H insertion and [2+1] cycloaddition, to access a diverse library of nitrogen‐containing compounds. With this in mind, we theorized that anomeric amides of type **1** [29] could be harnessed to facilitate a related reaction sequence in which aldehydes (**2**) are directly converted to the analogous *N*‐Boc‐hydroxamates (**3**) via a sequence of HAT followed by acylation (Figure 1c). We further anticipated that these intermediates (**3**) could be activated under a variety of orthogonal conditions to deliver not only isocyanates (via the Lossen rearrangement) [30, 31, 32] but also reactive species such as acyl nitrenoids [33, 34, 35], nitrogen‐centered radicals (upon single‐electron transfer) [36, 37, 38], and act as acyl cation equivalents. It should be noted that each of these individual transformations targeted here have been reported using alternative strategies [30, 31, 32, 33, 34, 35, 36, 37, 38]. Furthermore, the corresponding products generated can be accessed in a divergent, modular fashion using starting materials such as Cbz‐protected amines [39] and carbamoylzinc pivalates [18], or alternatively through metal‐catalyzed dehydrogenative coupling of formamides [40, 41] (see Supporting Information for a detailed comparison of previous methods to our work). However, these typically afford only a subset of the products accessible through our proposed approach. As such, no current method exists in which a single intermediate structure, derived from aldehydes, can be used to directly access this broad array of reactive intermediates through judicious choice of activation conditions, providing new opportunities for rapid diversity generation.

Herein, we report the use of *N*‐halo‐*O*‐activated hydroxycarbamate‐type anomeric amides (**1**) to convert aromatic and aliphatic aldehydes into unsymmetrical ureas, carbamates, thiocarbamates, thioesters, amines, and amides (Figure 1d). The utility of this transformation is further highlighted by its application to numerous medicinally relevant fragments and has been demonstrated to both facilitate the diversification of complex aldehydes and rapidly generate molecular complexity from feedstock aldehyde building blocks. The unique aspect of this strategy lies in the breadth of transformations accessible from the *N*‐Boc‐hydroxamate intermediates, providing a powerful route to a wide array of new molecular entities from a single aldehyde functional handle in a one‐pot sequence.

## 2 Results and Discussion

**FIGURE 2:**
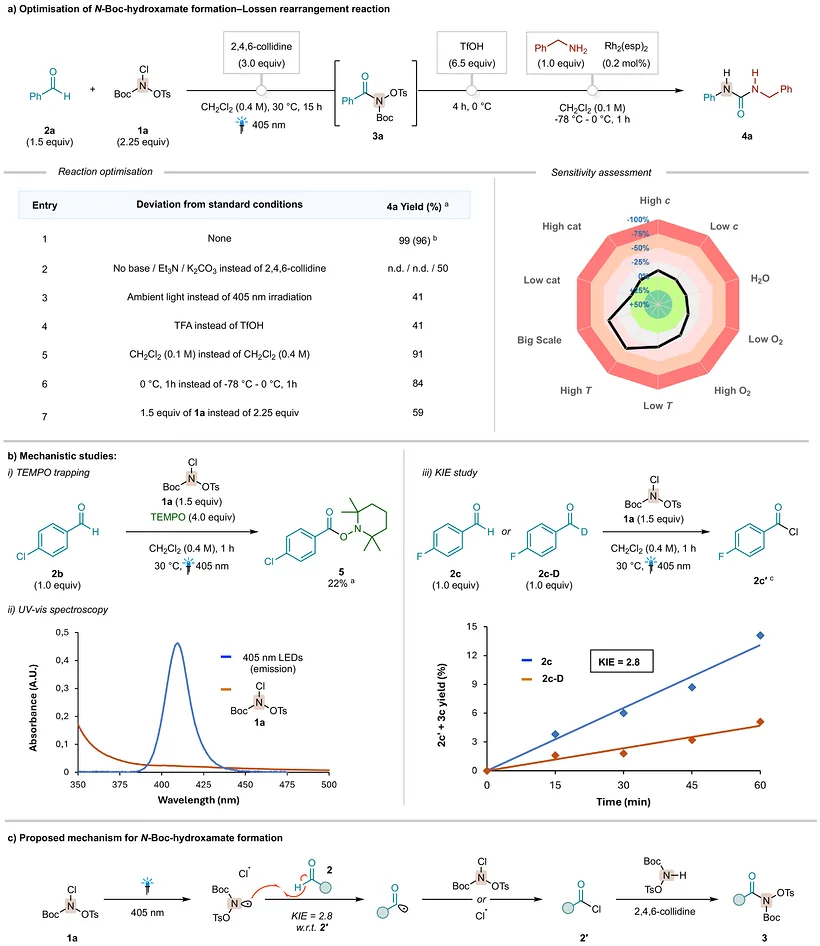
Optimization and mechanistic studies of the conversion of aldehydes to unsymmetrical ureas. a) Reaction optimization and sensitivity screen. b) Mechanistic studies. c) Proposed mechanism. Reactions were performed on 0.1 mmol scale, unless otherwise stated. Reactions irradiated with 18 W blue LEDs (λ_max_ = 405 nm). ^a^ ^1^H NMR yield using dibromomethane as an internal standard. ^b^ Isolated yield. ^c^ 0.05 mmol scale. ^19^F NMR yield, calculated using methyl 4‐fluorobenzoate as an internal standard, was taken as the sum of acyl chloride **2c′** and minor amounts of the corresponding *N*‐Boc‐hydroxamate.

Our studies began with the development of a protocol to facilitate the conversion of an aldehyde into the key *N*‐Boc‐hydroxamate intermediate (**3**), achieved by subjecting benzaldehyde (**2a**) to *N*‐chloro‐*N*‐Boc‐*O*‐(toluenesulfonyl)hydroxylamine (**1a**) in the presence of 2,4,6‐collidine and irradiating with blue light (Figure 2a). We first optimized the aldehyde‐to‐hydroxamate transformation, which proceeded in high efficiency (up to 88% yield, see Supporting Information for optimization details). Having established these conditions, we then evaluated the versatility of this intermediate by converting it into multiple reactive species, each enabling a distinct functionalization mode within a one‐pot process. Although multiple transformations were explored simultaneously (see Supporting Information for details), we explicitly detail below our optimization studies regarding converting *N*‐Boc‐hydroxamate **3** into the corresponding isocyanate as a representative example. This approach was deemed particularly valuable as related hydroxamate‐to‐isocyanate transformations have been previously harnessed to engage a variety of nucleophiles to afford multiple classes of products [32, 42, 43, 44, 45]. Thus, this reactivity represents the methodology with the highest degree of potential divergency [31, 46]. Furthermore, the addition of amine‐based nucleophiles to aldehyde‐derived isocyanates facilitates the modular construction of unsymmetrical ureas, fragments that are of considerable pharmaceutical interest [47]. Accordingly, this transformation was explored in greater detail than the other reactivity modes, which are instead represented by concise sets of examples.

Upon formation of intermediate **3a**, it was demonstrated that this species could be subsequently converted in one‐pot to the desired urea product (**4a**) upon acidic *N*‐deprotection, Lossen rearrangement, and nucleophilic trapping of the isocyanate in 96% isolated yield (Figure 2a, entry 1). It must be noted that previous iterations of similar overall reaction sequences, often performed in the context of drug discovery [48, 49, 50, 51], require multiple synthetic steps, intermediate purifications, and the use of hazardous azide reagents to initiate a Curtius rearrangement [52, 53, 54]. Key to the formation of **3a** was the identity of the base, with hindered pyridines proving optimal in order to suppress decomposition of **1a** (Figure 2a, entry 2). Interestingly, when performing the reaction under ambient light, product formation was observed, albeit in decreased quantities (entry 3). This is consistent with the known propensity of the N─Cl bond in such species to undergo homolytic cleavage even in the dark, suggesting that a minor thermal pathway may also contribute alongside the photochemical bond activation [27]. Subsequent repetitions of this result found that the outcome varied significantly between reactions that were ran under seemingly identical conditions, whereas no such irreproducibility was observed upon blue light irradiation (see Supporting Information for details).

Based on our previous studies, we initially anticipated that both *N*‐deprotection and the subsequent Lossen rearrangement of **3a** could be facilitated by employing Rh_2_esp_2_ [55] in 1,1,1,3,3,3‐hexafluoro‐2‐propanol (HFIP) solvent [27, 35]. Indeed, this was found to provide access to the desired unsymmetrical urea product **4a**, although diminished yields were obtained as solvent addition to the isocyanate intermediate was discovered to be competitive with nucleophilic addition from the amine (see Supporting Information for details). This necessitated the replacement of HFIP with a non‐nucleophilic solvent in combination with a Brønsted acid to first promote Boc deprotection before the addition of the Rh catalyst and amine nucleophile [56, 57, 58, 59, 60]. In this regard, it was discovered that TfOH was the optimal additive, whereas acids with more nucleophilic conjugate bases intercepted the highly electrophilic isocyanate (entry 4). Further screening of conditions revealed that a higher reaction concentration in the initial *N*‐Boc‐hydroxamate formation step (entry 5), low temperature during the addition of acid (entry 6), and an excess of **1a** relative to the aldehyde (entry 7), were all required to achieve a high yield of product **4a** (see Supporting Information for full optimization studies). A sensitivity assessment of these conditions [61, 62] demonstrated that the transformation was largely insensitive to concentration, oxygen content, and the presence of water. The reaction was noticeably impacted by increases in both temperature and scale which reflect the exothermic nature of the deprotection and Lossen rearrangement steps as well as the temperature sensitivity of **1a** (see Supporting Information for sensitivity screen details and DSC studies). Finally, it was discovered that reaction efficiency could be maintained at catalyst loadings as low as 200 ppm, while the complete absence of catalyst led to no formation of product, indicating its requirement in the rearrangement step (see Supporting Information) [63, 64].

To gain a deeper understanding of the reaction pathway for the formation of intermediate *N*‐Boc‐hydroxamate **3**, we conducted a series of mechanistic experiments. In order to confirm that the transformation proceeds via HAT from the aldehyde, we employed TEMPO as a radical trapping agent under standard conditions with 4‐chlorobenzaldehyde (**2b**, Figure 2bi). Complete suppression of the formation of **3** was observed and adduct **5** was instead detected, confirming the intermediacy of the aldehyde‐derived acyl radical in this transformation. UV‐vis spectroscopy demonstrated that only anomeric amide **1a** displays significant overlap between the absorption band of the molecule and the emission band of the light source (Figure 2bii). This suggested that light‐promoted excitation initiates N−Cl bond homolysis to generate the initial radical species. Based on previously reported related systems [23], we hypothesized that acyl radical chlorination is likely to occur via an interaction with **1a**, propagating the radical chain. However, confirmation of this pathway through quantum yield measurements was unfeasible due to the minor contribution of background thermal N–Cl homolysis. Monitoring the reaction by ^1^H NMR demonstrated that the reaction proceeds via the formation of acyl chloride **2′**, with concurrent formation of the corresponding N–H hydroxycarbamate. It could also be deduced that the rate of formation of *N*‐Boc‐hydroxamate **3** is limited by the acylation step, as determined by the observed buildup of the acyl chloride (**2′**) and subsequent slow conversion to **3** (see Supporting Information). With respect to the formation of the acyl chloride however, KIE experiments (Figure 2biii) indicate that the HAT step is likely to be rate‐limiting, with a KIE of 2.8 measured between the reactions of 4‐fluorobenzaldehyde **2c** and its deuterated counterpart (**2c‐D**) [65]. Taking these results together, we can therefore propose a mechanism in which light‐promoted excitation facilitates the homolytic cleavage of **1a** (Figure 2c). The resulting nitrogen‐centered radical can then participate in aldehyde HAT before the corresponding acyl radical generates the acyl chloride (**2′**) via either radical recombination or halogen atom abstraction of **1a**. Finally, base‐promoted acylation with the reduced form of **1a** can then provide the observed *N*‐Boc‐hydroxamate product (**3**). This is consistent with previous reports describing related transformations that proceed through similar sequences of these fundamental steps [21, 23, 26].

**FIGURE 3:**
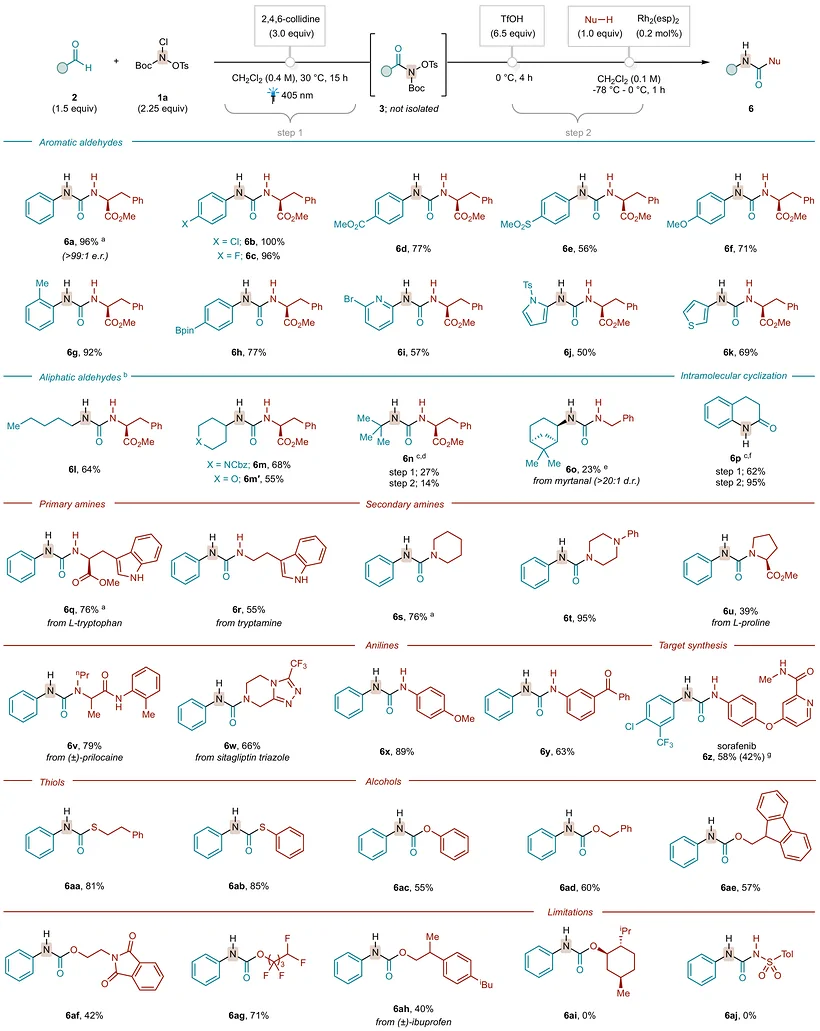
Substrate scope for the divergent synthesis of ureas, carbamates, and thiocarbamates from aldehydes. Reactions performed on 0.2 mmol scale, under argon atmosphere. Reactions irradiated with 18 W blue LEDs (λ_max_ = 405 nm). Isolated yields given. ^a^ Using 2,4,6‐collidine (1.65 equiv). ^b^ Using aldehyde (1.8 equiv) and **1a** (1.5 equiv). ^c^ Intermediate *N*‐Boc‐hydroxamate was isolated. ^d^ Step 1: Using aldehyde **2n** (1.2 equiv), 2,4,6‐collidine (2.0 equiv), DMAP (0.1 equiv), and **1a** (1.0 equiv). Step 2: Standard conditions with **3n** (1.5 equiv). ^e^ Reaction performed on 0.4 mmol scale. ^f^ Step 1: Using aldehyde **2p** (1.2 equiv), 2,4,6‐collidine (2.0 equiv), and **1a** (1.0 equiv). Step 2: Using **3p** (1.0 equiv) and TFA (2.0 equiv), DCM (0.4 M), rt, 6 h then Rh_2_(esp)_2_ (0.2 mol%), HFIP:DCM (0.2 M), 0 °C—rt, 15 h. ^g^ Reaction performed on 1.0 mmol scale.

In order to probe the generality of our procedure, we explored the substrate scope of the newly developed reaction with respect to the identity of the aldehyde (Figure 3). Given that our transformation was developed in the context of accessing a strategy for divergent aldehyde functionalization that can be applied to the modification and unification of complex and medicinally relevant moieties, we employed phenylalanine methyl ester as our model nucleophile (**6a**). To showcase the synthetic utility of the method, the reaction was also performed on a 4 mmol scale, affording (**4a**) in 70% isolated yield (see Supporting Information for details). Pleasingly, we discovered that electron‐deficient (**6b**‐**6e**), electron‐rich (**6f**), and *ortho*‐substituted aromatic aldehydes (**6g**) could be transformed into the desired products in good to excellent yields. Chiral HPLC analysis demonstrated that the stereochemical information of the amino acid stereocenter is fully retained under the reaction conditions (**6a**). Additionally, valuable linchpin fragments such as boronic esters (**6h**) and heterocycles such as pyridines (**6i**), pyrroles (**6j**), and thiophenes (**6k**) could also be incorporated into the targeted unsymmetrical ureas. Furthermore, α‐primary, and α‐secondary aliphatic aldehydes were effective in this one‐pot protocol, allowing the synthesis of amino acid‐derived ureas **6l**‐**6m**. For a series of substrates in which a lower yield of the desired urea product was observed compared to the model substrate, the corresponding *N*‐Boc‐hydroxamate formation step was analyzed independently. It was determined that the yield of this step was also reduced, indicating the initial aldehyde functionalization transformation is largely responsible for this decrease in reaction efficiency (see Supporting Information for details). Investigation of the steric limits of this transformation revealed that the use of α‐tertiary pivaldehyde resulted in a substantially reduced yield of the desired product (**6n**) and necessitated isolation of the intermediate *N*‐Boc‐hydroxamate. The ability to maintain stereointegrity at the aldehyde α‐position was also interrogated through employing myrtanal, with no change in diastereomeric ratio indicating that racemization is not occurring (**6o**). Interestingly, when hydrocinnamaldehyde was employed, the desired Lossen rearrangement was outcompeted by favorable intramolecular C−H insertion of the acyl nitrenoid, providing dihydroquinolinone (**6p**) in 59% yield over 2 steps [35].

We next turned our attention to the application of alternative nucleophiles in an effort to expand the level of product diversity achievable from our *N*‐Boc‐hydroxamate formation–Lossen rearrangement sequence. Primary amines such as tryptophan methyl ester (**6q**) and tryptamine (**6r**) were tolerated in this transformation, as were the secondary amines piperidine (**6s**), *N*‐phenyl piperazine (**6t**), proline methyl ester (**6u**), prilocaine (**6v**), and sitagliptin triazole (**6w**). In addition, primary anilines (**6x**‐**6y**) could be directly applied. It was also demonstrated that the synthesis of the cancer growth blocker sorafenib (**6z**) [66], typically requiring the synthesis and isolation of the key isocyanate reagent from the corresponding amine and triphosgene, could be achieved using our *N‐*Boc‐hydroxamate formation−Lossen rearrangement reaction in one‐pot. Although sulfonamides and particularly hindered nucleophiles were deemed unreactive in this protocol (**6ai**‐**6aj**), we successfully extended this procedure to thiols (**6aa**), thiophenols (**6ab**), phenols (**6ac**), and alcohols (**6ad**‐**6ah**), which facilitated the synthesis of a concise and diverse library of functionalized products.

**FIGURE 4:**
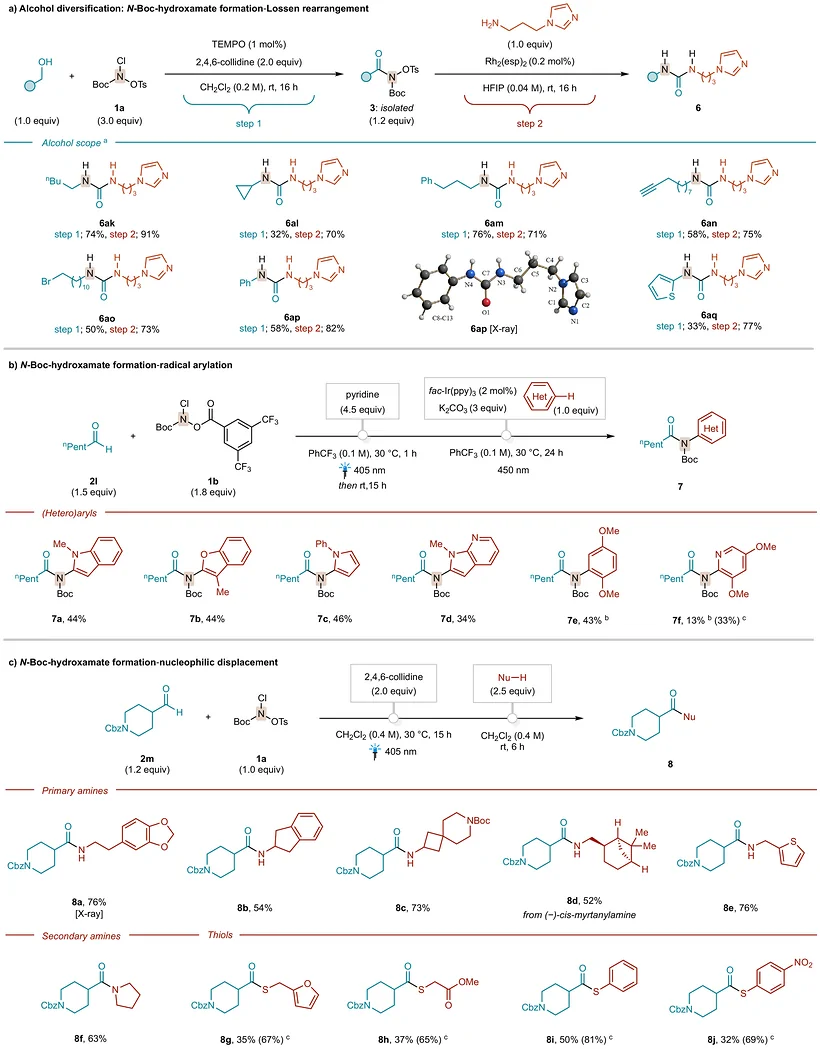
Substrate scope for the divergent synthesis of ureas, amides, and thioesters from alcohols and aldehydes. Reactions were performed on 0.2 mmol scale, under an atmosphere of argon. Reactions irradiated with 18 W blue LEDs (*λ*_max_ = 405 nm) or 18 W blue LEDs (*λ*_max_ = 450 nm). Isolated yields given. ^a^ Step 1 was performed on 0.5 mmol scale, step 2 was performed on 0.2 mmol scale. Isolated yields for the individual steps are reported. ^b^ Reactions performed with aldehyde (0.2 mmol, 1.0 equiv), **1b** (1.2 equiv), pyridine (3.0 equiv), and arene (10 equiv). ^c 1^H NMR yield calculated using dibromomethane as an internal standard is given in parentheses.

The unique versatility of anomeric amides of type **1** is highlighted by the ability of the nitrogen atom to act initially as a source of nitrogen‐centered radicals, followed by a nucleophile during the acylation step, and finally as a nitrenoid precursor. At this juncture, we were intrigued to determine whether reagents of this structure could also facilitate substrate oxidation in the presence of catalytic TEMPO, allowing the same reaction sequence to be performed on redox‐related alcohols [67, 68]. Accordingly, we discovered that subjecting a range of primary alcohol substrates to an excess of **1a**, in the presence of catalytic amounts of TEMPO, enabled the formation of the desired *N*‐Boc‐hydroxamates (Figure 4a). Notably, it was discovered that, due to the increased quantities of byproducts associated with the alcohol oxidation−HAT−acylation sequence, an intermediate purification became necessary in order to obtain improved yields of the urea products formed upon addition of 3‐(1*H*‐imidazol‐1‐yl)propan‐1‐amine (**6ak**‐**6aq**).

Having demonstrated the ability of aldehyde‐ and alcohol‐derived *N*‐Boc‐hydroxamates to undergo Lossen‐type isocyanate formation and functionalization, our focus then shifted toward generating alternative reactive intermediates under orthogonal conditions. Specifically, nitrogen‐centered radicals formed via reduction and N−O mesolytic cleavage were targeted as the unique reactivity profile of these intermediates would allow access to complementary regions of chemical space [69]. We were inspired by the work of both Chang and Xu, who succinctly demonstrated that *N*‐benzoyloxyamides can undergo photoredox‐catalyzed single‐electron transfer (SET) followed by arylation of the resulting electron‐deficient nitrogen‐centered radical [37, 38]. Accordingly, 3,5‐bis(trifluoromethyl) benzoate‐containing anomeric amide **1b** was synthesized and coupled with aldehyde **2l** to promote a modified one‐pot sequence of *N*‐Boc‐hydroxamate formation followed by photoredox‐catalyzed arylation (Figure 4b). Using this strategy allowed the formation of a variety of *N*‐(hetero)aryl amides, directly from the unfunctionalized parent heterocycles, including those derived from indole (**7a**), benzofuran (**7b**), pyrrole (**7c**), 7‐azaindole (**7d**), dimethoxy benzene (**7e**), and pyridine (**7f**), in moderate yields. Given the energetic barrier required to break aromaticity and the electron‐deficient nature of the intermediate radical, it is unsurprising that only polarity‐matched electron‐rich arenes display the desired reactivity (see Supporting Information for all failed substrates).

To further demonstrate the synthetic versatility of the *N*‐Boc‐hydroxamate intermediates, we next explored a complementary approach in which these species, upon in situ generation, were subjected directly to a variety of nucleophiles to obtain the corresponding acylated products. Indeed, this strategy was successful in the synthesis of a concise range of secondary amides, tertiary amides, and thioesters derived from both thiols and thiophenols (Figure 4c, **8a**‐**8j**). In the case of thioketone products (**8g**‐**8j**), instability toward chromatographic purification resulted in lower isolated yields than would be expected based on quantitative ^1^H NMR analysis of the reaction outcome. Interestingly, when attempting to avoid the intermediacy of the *N*‐Boc‐hydroxamate and directly engage the acyl chloride in nucleophilic substitution, a decrease in the yield of the desired product was observed (see Supporting Information for details).

**FIGURE 5:**
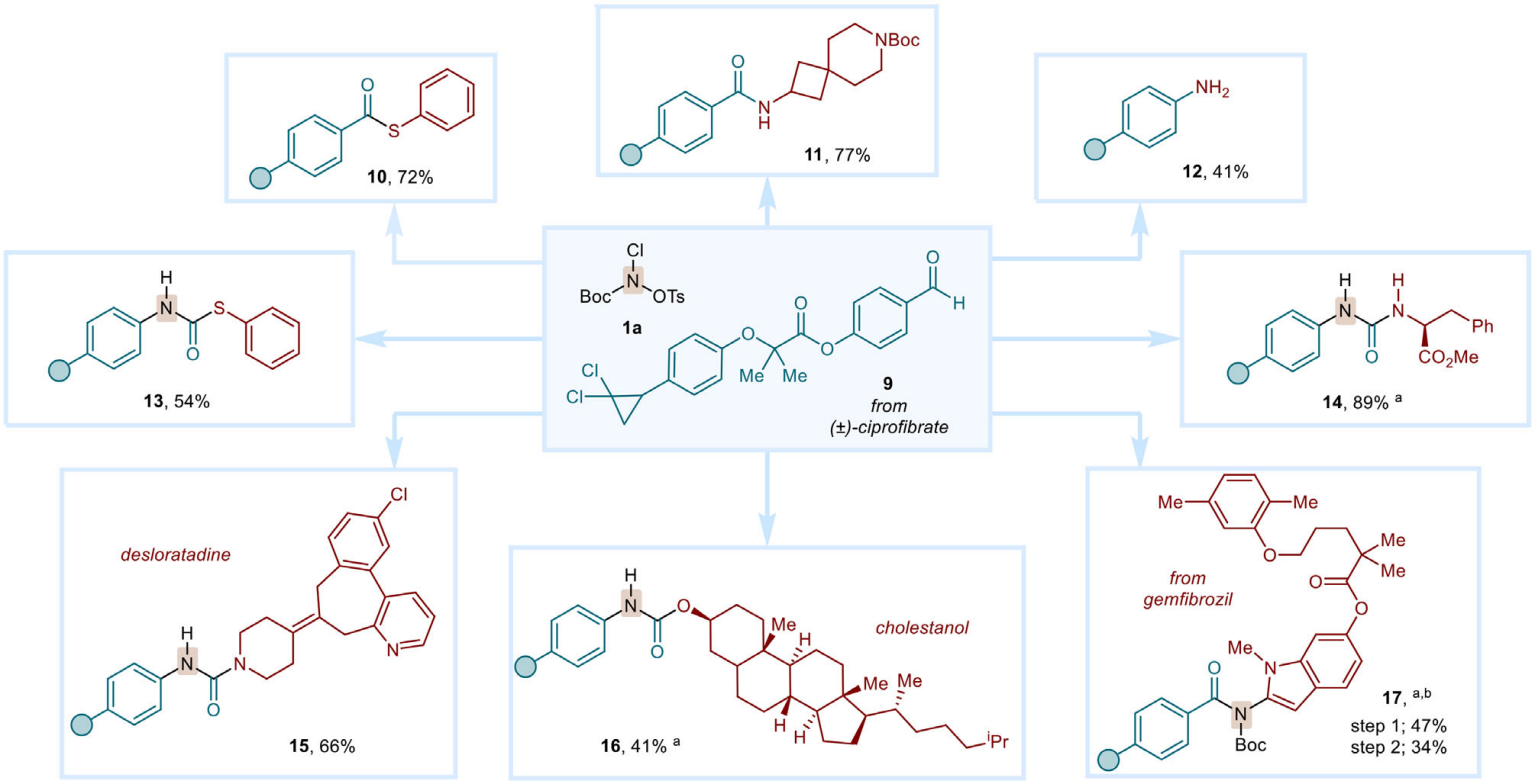
Divergent functionalization of ciprofibrate derivative. Reactions were performed on 0.2 mmol scale, see Supporting Information for full conditions. Isolated yields given. ^a^ Coupling partners containing stereocenters generated the corresponding products as a 1:1 mix of inseparable diastereomers. ^b^ Using anomeric amide **1b**. Intermediate *N‐*Boc‐hydroxamate was isolated before subjection to the next reaction.

To scrutinize the potential applicability of the methodology described herein to allow aldehydes to act as linchpin fragments in diversity‐generating syntheses, we explored the breadth of chemical space that could be accessed from a single medicinally relevant aldehyde derivative. Accordingly, the 4‐benzaldehyde derivative of the hypolipidemic ciprofibrate (**9**) was synthesized and systematically subjected to all our reaction protocol variants (Figure 5). Employing the *N*‐Boc‐hydroxamate formation−nucleophilic displacement strategy facilitated the synthesis of thioester **10** and amide **11**. We next demonstrated that using our *N*‐Boc‐hydroxamate formation−Lossen rearrangement reaction and engaging the in situ generated isocyanate with water could facilitate the formation of the corresponding amine. This proceeds via carbamic acid formation and subsequent decarboxylation to provide **12**. In addition, thiocarbamate (**13**) and urea (**14**) derivatives were accessible via the addition of thiophenols and anilines respectively. This protocol was also extended to the antihistamine desloratadine and the steroid cholestanol to give complex conjugates **15** and **16** in a one‐pot reaction sequence. Furthermore, the *N*‐Boc‐hydroxamate formation−radical arylation strategy was successfully achieved using anomeric amide **1b**, yielding the desired amide product upon coupling with an indole derivative of gemfibrozil (**17**), although an intermediate purification of the *N*‐Boc‐hydroxamate was required in this case.

## 3 Conclusion

In summary, we have developed a complementary approach for the synthesis of unsymmetrical ureas, carbamates, thiocarbamates, thioesters, amines, and amides via the anomeric amide‐enabled diversification of aldehydes. While related divergent functionalization platforms have been reported, the distinguishing feature of this work is the use of an aldehyde‐derived hydroxamate linchpin that enables controlled, condition‐dependent switching between several fundamentally different reaction modes. Mechanistic studies indicated that the formation of the key *N‐*Boc‐hydroxamate intermediate occurs via light‐induced N−Cl bond homolysis, aldehyde hydrogen atom transfer, chlorine radical capture, and acylation. This species could then be activated under a range of orthogonal conditions and coupled with a variety of complex and medicinally relevant nucleophiles and radical acceptors to rapidly generate molecular complexity. The applicability and utility of this strategy were further demonstrated by the late‐stage diversification of the lipid‐lowering agent ciprofibrate into a variety of drug analogues. We anticipate that this concept will be extendable to other classes of starting materials and activation modes, thereby further broadening the scope of divergent functionalization strategies.

## Conflicts of Interest

The authors declare no conflicts of interest.

## Supporting information



Experimental procedures and characterization data for new compounds (PDF). The authors have cited additional references within the Supporting Information [70, 71, 72, 73, 74, 75, 76, 77, 78, 79, 80, 81, 82, 83].
**Supporting File**: anie73404‐sup‐0001‐SuppMat.pdf.

## Data Availability

The data that supports the findings of this study are available in the Supporting Information of this article. Deposition numbers 2492593 (for **6ap**) and 2492594 (for **8a**) contain the supplementary crystallographic data for this paper. These data are provided free of charge by the joint Cambridge Crystallographic Data Centre and Fachinformationszentrum Karlsruhe Access Structures service.
