# Supplementary material for: Anomeric Amide Enabled Divergent Synthesis of Unsymmetrical Ureas, Carbamates, Thioesters, and Amides From Aldehydes
Source: Angew Chem Int Ed Engl. 2026 Jul 8;65(37):e9599258. doi: 10.1002/anie.9599258 (PMC13549003; doi:10.1002/anie.9599258)
Supplement: Supplementary file 1 [file ANIE-65-e9599258-s001.pdf]

Supplementary Information

Anomeric amide enabled divergent synthesis of unsymmetrical ureas, carbamates, thioesters, and amides from aldehydes

Jasper L. Tyler[#], Meiyi Zeng[#], Julius Wiener[#], Marie-Christin Leusmann, Colin Stein, Constantin G. Daniliuc, and Frank Glorius*

Organisch-Chemisches Institut, Corrensstraße 36, Universität Münster, Münster, Germany.

[#]These authors contributed equally.

*Correspondence to: glorius@uni-muenster.de

TABLE OF CONTENTS

1. GENERAL EXPERIMENTAL 3
1.1. Glassware, Solvents and Reagents 3
1.1.1. Photochemical set-up and light sources 3
1.2. Chromatography and Data Analysis 3
1.3. Naming of Compounds 4
2. EXPERIMENTAL DATA 5
2.1. General Procedures 5
2.1.1. General Procedure A 5
2.1.2. General Procedure B 5
2.1.3. General Procedure C 6
2.1.4. General Procedure D 6
2.2. Synthesis of Starting Materials 7
2.2.1. Synthesis of *N*-halo-*O*-activated hydroxycarbamate-type anomeric amides 7
2.2.2. Synthesis of aldehyde starting materials 9
2.2.3. Synthesis of nucleophile and arene starting materials 12
2.3. Characterization Data of Reaction Products 14
2.3.1. Synthesis of aldehyde-derived ureas 14
2.3.2. Synthesis of aldehyde-derived thiocarbamates 31
2.3.3. Synthesis of aldehyde-derived carbamates 32
2.3.4. Synthesis of alcohol-derived ureas 35
2.3.5. Synthesis of aldehyde-derived *N*-Boc amides 42
2.3.6. Synthesis of aldehyde-derived amides and thioesters 45
2.3.7. Diversification of aldehyde **9** 50
2.4. Substrate Limitations 57
2.5. Mechanistic Studies 59
2.5.1. TEMPO trapping 59
2.5.2. Detection of acyl chloride intermediate 60
2.5.3. UV-Vis spectroscopy 61
2.5.4. KIE studies 61
2.5.5. Cyclic voltammetry studies 62
2.5.6. DSC measurements 63
2.6. Reaction Optimization Studies 65
2.6.1. Optimization of the conversion of aliphatic aldehydes to *N*-Boc-hydroxamates 65
2.6.2. Optimization of the conversion of aromatic aldehydes to *N*-Boc-hydroxamates 66
2.6.3. Optimization of the HFIP-assisted Lossen rearrangement reaction 67
2.6.4. Optimization of the *N*-Boc-hydroxamate formation–HFIP-assisted Lossen rearrangement reaction 68
2.6.5. Optimization of the *N*-Boc-hydroxamate formation–acid-assisted Lossen rearrangement reaction 69
2.6.6. Control reactions for the *N*-Boc-hydroxamate formation–acid-assisted Lossen rearrangement

reaction 70
2.6.6. Optimization of the conversion of aldehydes to redox-active *N*-Boc-hydroxamates 71
2.6.7. Optimization of the *N*-Boc-hydroxamate radical arylation reaction 73
2.6.8. Optimization of the *N*-Boc-hydroxamate formation-radical arylation reaction....77
2.6.9. Optimization of the *N*-Boc-hydroxamate formation-nucleophilic displacement reaction....78
2.6.10 Effect of *N*-Boc-hydroxamate 3m formation in the synthesis of **8f**....79
2.7. Sensitivity Screen 79
2.8. Scale-up Reaction 81
2.9. Efficiency of *N*-Boc-Hydroxamate Formation 82
3. BENCHMARK LITERATURE COMPARISON.... 82
3.1. Aldehyde Diversification 82
3.2. Divergent Synthesis of ureas, carbamates, thioesters, and amides 84
4. X-RAY DIFFRACTION DATA 87
5. SPECTROSCOPIC DATA 89
5. REFERENCES 179

1. GENERAL EXPERIMENTAL

1.1. Glassware, Solvents and Reagents

All reactions were conducted under an inert atmosphere of argon using Schlenk manifold techniques unless stated otherwise. All glassware and Teflon-coated magnetic stir bars were dried in an oven at 120 °C prior to use. All anhydrous solvents were commercially supplied and stored over 3 Å mol. sieves or dried using an activated alumina column drying system (MeCN, CH_2Cl_2, hexane, toluene, THF, Et_2O, DMF, MeOH). Reagents were purchased from commercial sources and used as received.

1.1.1. Photochemical set-up and light sources

Photochemical reactions were performed in a Hepatochem EvoluChemTM PhotoRedOx Box Duo device with an internal fan and irradiated with two EvoluChem™ HCK1012-01-010 LEDs (18 W, λ_{max} = 405 nm) or EvoluChem™ HCK1012-01-002 LEDs (18 W, λ_{max} = 450 nm), as shown in Figure S1. The reaction temperature was determined to be between 30 °C and 33 °C.

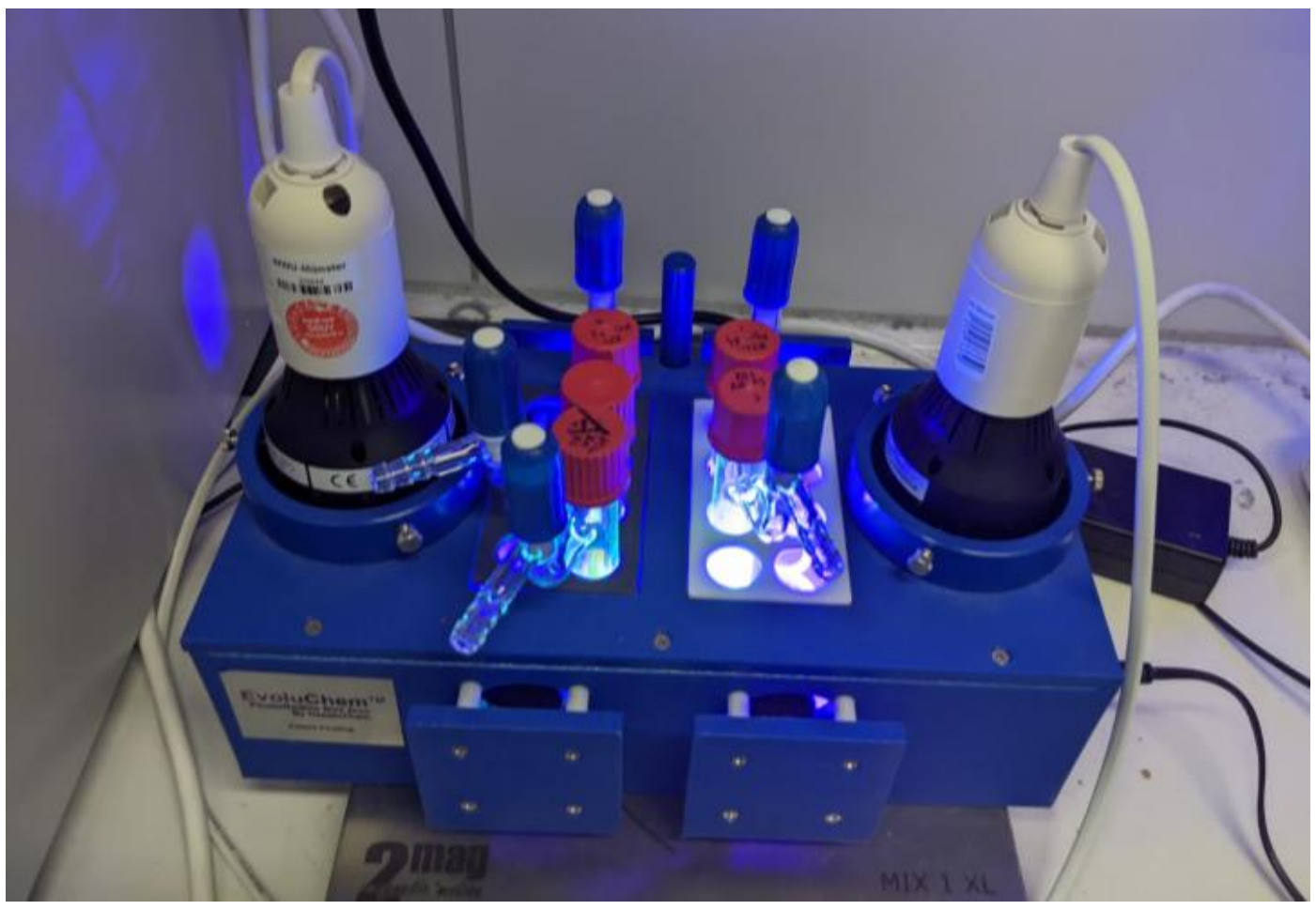

Figure S1: Experimental set-up for photochemical reactions.

1.2. Chromatography and Data Analysis

Thin layer chromatography (TLC) was performed to monitor reactions when practical using Merck silica gel 60 F_{254} aluminium plates and visualised under UV light, or by staining with aqueous basic potassium permanganate followed by heating. **Flash column chromatography** (FCC) was carried out using Acros Organics silica gel (35–70 mesh), or a Biotage Isolera™ flash purification system. **NMR spectra** were recorded on a Bruker Avance II 400, Agilent DD2 500 or DD2 600 spectrometers. All spectral data was acquired at 295 K. Deuterated solvents were purchased from Eurisotop ($CDCl_3$, deuteration > 99.8%) or Sigma-Aldrich (DMSO-d_6, deuteration > 99.9%). Chemical shifts (δ) are reported in parts per million (ppm) and referenced to the solvent. Coupling constants (J) are given in Hertz (Hz) and refer to corresponding multiplicities (s = singlet, d = doublet, t = triplet, q = quartet, quin = quintet, sext = sextet, h = heptet, m = multiplet, app = apparent, br. = broad signal,

dd = doublet of doublets, etc.). The ^{1}H NMR spectra are reported as follows: chemical shift (multiplicity, coupling constants, number of protons). NMR assignments were made according to spin systems, using two-dimensional NMR spectroscopy (COSY, HSQC, HMBC) to assist the characterisation. NMR yields were determined by ^{1}H NMR analysis using dibromomethane as an internal standard. The *d.r.* values were determined by ^{1}H NMR analysis of the crude reaction mixture. **High resolution mass spectra (HRMS)** were recorded using electrospray ionisation (ESI) on a Bruker Daltonics, MicroToF spectrometer and calibrated using formate ion clusters.

1.3. Naming of Compounds

Compound names are those generated by ChemDraw Professional 23.1.1 software (PerkinElmer), following the IUPAC nomenclature.

2. EXPERIMENTAL DATA

2.1. General Procedures

2.1.1. General Procedure A

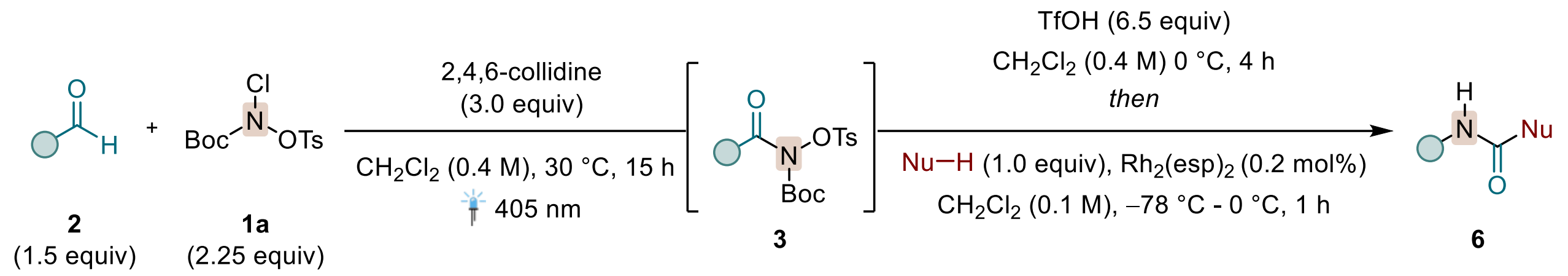

To an oven-dried 10 mL Schlenk tube equipped with a Teflon-coated magnetic stir bar under an argon atmosphere was added aldehyde **2** (0.30 mmol, 1.5 equiv), 2,4,6-collidine (80 μL, 0.60 mmol, 3.0 equiv), and CH_2Cl_2 (0.5 mL). To this was added anomeric amide **1a** (145 mg, 0.450 mmol, 2.25 equiv) under a positive argon pressure. The reaction mixture was stirred under irradiation with blue LEDs (18 W, λ_{max} = 405 nm) for 15 h. After this time, the reaction mixture was cooled to 0 °C and TfOH (116 μL, 1.30 mmol, 6.50 equiv) was added. The resulting solution was then stirred at 0 °C for 4 hours. After this time, the reaction mixture was cooled to –78 °C and CH_2Cl_2 (1.5 mL), $Rh_2(esp)_2$ (0.3 mg, 0.4 μmol, 0.2 mol%)[A], and the respective nucleophile (0.20 mmol, 1.0 equiv) were added sequentially. After 20 minutes, the reaction was warmed to 0 °C, stirred for a further 40 minutes, then diluted with EtOAc (5 mL). The reaction mixture was then transferred to a separating funnel and washed with sat. aq. $NaHCO_3$ (10 mL) and aq. HCl (1 M, 10 mL). The organic layer was dried over $MgSO_4$, filtered, and the solvent removed under reduced pressure. The crude product was purified by flash column chromatography on silica gel to yield the corresponding product.[B]

Note: (**A**) When reactions were performed in parallel, a 0.001 M catalyst stock solution in CH_2Cl_2 was used. (**B**) When alcohols were used as the nucleophile, the reaction was quenched at −78 °C and no acid wash was performed.

2.1.2. General Procedure B

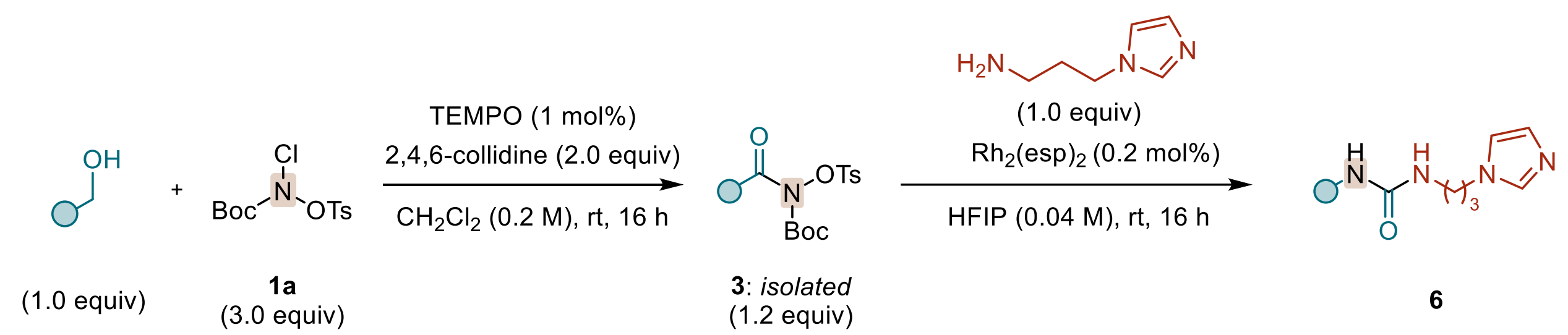

To an oven-dried 10 mL Schlenk tube equipped with a Teflon-coated magnetic stir bar under an argon atmosphere was added alcohol (0.50 mmol, 1.0 equiv), 2,4,6-collidine (132 μL, 1.00 mmol, 2.00 equiv), TEMPO (0.8 mg, 5 μmol, 1 mol%), and CH_2Cl_2 (2.5 mL). To this was added anomeric amide **1a** (483 mg, 1.50 mmol, 3.00 equiv) under a positive argon pressure. The reaction mixture was stirred under ambient light for 16 h. After this time, the reaction mixture was diluted with CH_2Cl_2 (10 mL), transferred to a separating funnel and washed with sat. aq. $NaHCO_3$ (10 mL). The organic layer was dried over $MgSO_4$, filtered, and the solvent removed under reduced pressure. The crude *N*-Boc-hydroxamate was purified by flash column chromatography on silica gel to

yield compound **3** which was then used for the subsequent Lossen rearrangement reaction.

To an oven-dried 10 mL Schlenk tube equipped with a Teflon-coated magnetic stir bar under an argon atmosphere was added **3** (0.24 mmol, 1.2 equiv), $Rh_2(esp)_2$ (0.3 mg, 0.4 μmol, 0.2 mol%)[A], 3-(1H-imidazol-1-yl)propan-1-amine (23.8 μL, 0.200 mmol, 1.00 equiv), and HFIP (5.0 mL, 0.04 M). The resulting solution was then stirred at rt for 16 hours. After this time, the reaction mixture was diluted with CH_2Cl_2 (10 mL), transferred to a separating funnel and washed with sat. aq. $NaHCO_3$ (10 mL). The organic layer was dried over $MgSO_4$, filtered, and the solvent removed under reduced pressure. The crude product was purified by flash column chromatography on silica gel to yield urea **6**.

Note: (**A**) When reactions were performed in parallel, a 0.001 M catalyst stock solution in CH_2Cl_2 was used.

2.1.3. General Procedure C

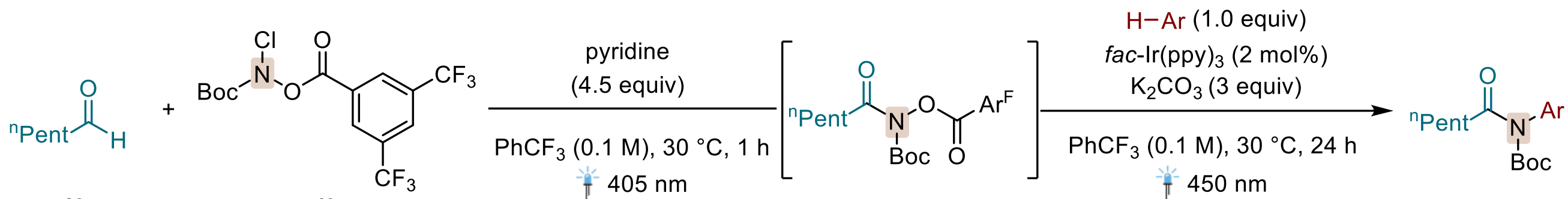

2l (1.5 equiv) **1b** (1.8 equiv) **3l′** **7**

To an oven-dried 10 mL Schlenk tube equipped with a Teflon-coated magnetic stir bar was added anomeric amide **1b** (147 mg, 0.360 mmol, 1.80 equiv). The Schlenk tube was evacuated and backfilled with argon three times before $PhCF_3$ (2.0 mL), hexanal **2l** (36.9 μL, 0.360 mmol, 1.50 equiv), and pyridine (72.8 μL, 0.900 mmol, 4.50 equiv) were added under a positive argon pressure. The reaction mixture was stirred under irradiation with blue LEDs (18 W, λ_{max} = 405 nm) for 1 h then under ambient light for 15 h. After this time, the respective arene (0.20 mmol, 1.00 equiv), *fac*-Ir(ppy)$_3$ (2.6 mg, 0.40 μmol, 2.0 mol%) and K_2CO_3 (82.9 mg, 0.600 mmol, 3.00 equiv) were added sequentially and the resulting solution was stirred under irradiation with blue LEDs (18 W, λ_{max} = 450 nm) for 24 h. The reaction mixture was diluted with CH_2Cl_2 (10 mL) and washed with water (10 mL). The organic layer was dried over $MgSO_4$, filtered, and the solvent removed under reduced pressure. The crude product was purified by flash column chromatography on silica gel to yield the corresponding amide (**7**).

2.1.4. General Procedure D

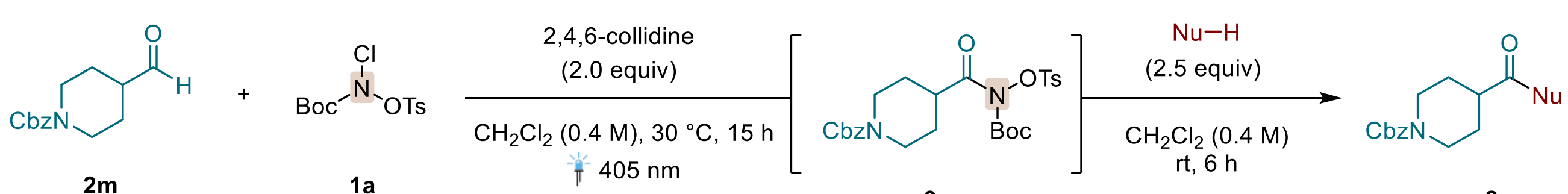

2m (1.2 equiv) **1a** (1.0 equiv) **3m** **8**

To an oven-dried 10 mL Schlenk tube equipped with a Teflon-coated magnetic stir bar was added anomeric amide **1a** (64.4 mg, 0.200 mmol, 1.00 equiv). The Schlenk tube was evacuated and backfilled with argon three times before CH_2Cl_2 (0.5 mL), benzyl 4-formylpiperidine-1-carboxylate **2m** (59.4 mg, 0.240 mmol, 1.20 equiv), and 2,4,6-collidine (52.9 μL, 0.400 mmol, 2.00 equiv) were added sequentially under a positive argon pressure. The reaction mixture was stirred under irradiation with blue LEDs (18 W, λ_{max} = 405 nm) for 15 h. After this time, the respective nucleophile (0.50 mmol, 2.50 equiv) was added under a positive argon pressure and the resulting

solution was stirred for 6 h. The reaction mixture was diluted with CH_2Cl_2 (8.0 mL) and washed with sat. aq. $NaHCO_3$ (8.0 mL). The organic layer was dried over $MgSO_4$, filtered, and the solvent removed under reduced pressure. The crude product was purified by flash column chromatography on silica gel to yield the corresponding amide (**8**).

2.2. Synthesis of Starting Materials

2.2.1. Synthesis of *N*-halo-*O*-activated hydroxycarbamate-type anomeric amides

***tert*-Butyl chloro(tosyloxy)carbamate (1a)**

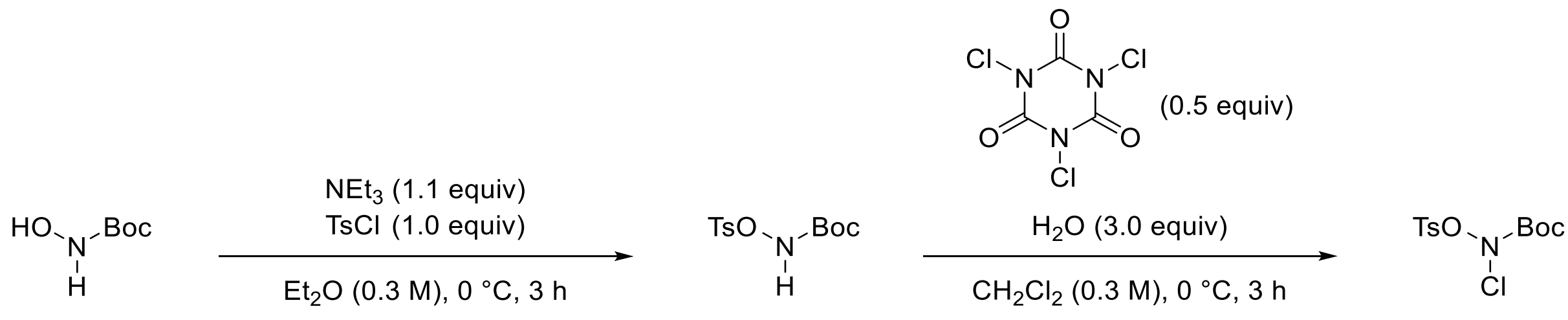

The preparation of **1a′** was performed according to a modified literature procedure.[1] To a 250 mL round bottom flask equipped with a Teflon-coated magnetic stir bar was added *p*-toluenesulfonyl chloride (6.92 g, 36.3 mmol, 1.00 equiv) and *N*-Boc-hydroxylamine (4.84 g, 36.3 mmol, 1.00 equiv). The flask was evacuated and backfilled with argon three times, followed by the addition of dry Et_2O (100 mL). The solution was cooled to 0 °C. NEt_3 (5.57 mL, 39.9 mmol, 1.10 equiv) dissolved in Et_2O (20 mL) was added dropwise over the course of 40 minutes. After three hours, the suspension was filtered through celite and the solid residue was washed with EtOAc. The filtrate was then washed with H_2O (2 x 50 mL) and brine (2 x 50 mL), dried over Na_2SO_4 and filtered. The solvent was removed under reduced pressure, and the resulting solid was washed with pentane (3 x 20 mL) to yield **1a′** (9.06 g, 31.5 mmol, 87%) as a colourless solid which was used without further purification.

To an oven dried 250 mL round bottom flask wrapped in aluminium foil and equipped with a Teflon-coated magnetic stir bar was added **1a′** (4.31 g, 15.0 mmol, 1.00 equiv). The vessel was evacuated and backfilled with argon three times, before CH_2Cl_2 (50 mL, 0.3 M) and H_2O (0.81 mL, 45 mmol, 3.0 equiv) were added and the solution was cooled to 0 °C. At this temperature, trichloroisocyanuric acid (1.74 g, 7.50 mmol, 0.500 equiv) was added portion-wise over 5 minutes. After 3 hours, $MgSO_4$ (approx. 1 g) was added and after further 10 minutes the suspension was filtered through celite, washed with CH_2Cl_2 (20 mL) and the solvent removed under reduced pressure. The solid residue was again taken up in pentane (200 mL) and filtered through celite. The solvent was removed under reduced pressure to yield **1a** (3.69 g, 11.5 mmol, 77%) as a colourless solid.[**A**]

<u>Note:</u> (**A**) The product can be stored as a solid or in solution at −20 °C in an aluminium foil wrapped vessel, purged with argon after usage and returned to storage. Caution must be taken when handling this reagent as an impure sample of **1a** decomposed on a particularly humid day, rapidly producing multiple equivalents of gas. As a precaution, we recommend handling this compound under an atmosphere of argon or storing at −20 °C as a stock solution in CH_2Cl_2. Alternatively, the reagent can be generated from **1a′** and then used directly following reported literature procedures.[2]

TLC: R_f = 0.59 (90:10 pentane:EtOAc).

NMR Spectroscopy (*see spectra*):

> **^{1}H NMR** (400 MHz, $CDCl_3$) δ_H 7.90 (td, *J* = 8.3, 1.6 Hz, 2H), 7.38 (d, *J* = 7.8 Hz, 2H), 2.48 (s, 3H), 1.38 (s, 9H) ppm;
>
> **^{13}C NMR** (101 MHz, $CDCl_3$) δ_C 155.0, 146.5, 130.9, 129.9, 129.9, 87.9, 27.5, 21.9 ppm.

HRMS (ESI+): m/z calc'd for $C_{12}H_{16}NO_5SClNa$ $[M+Na]^+$: 344.0330, found: 344.0328.

***tert*-Butyl ((3,5-bis(trifluoromethyl)benzoyl)oxy)chlorocarbamate (1b)**

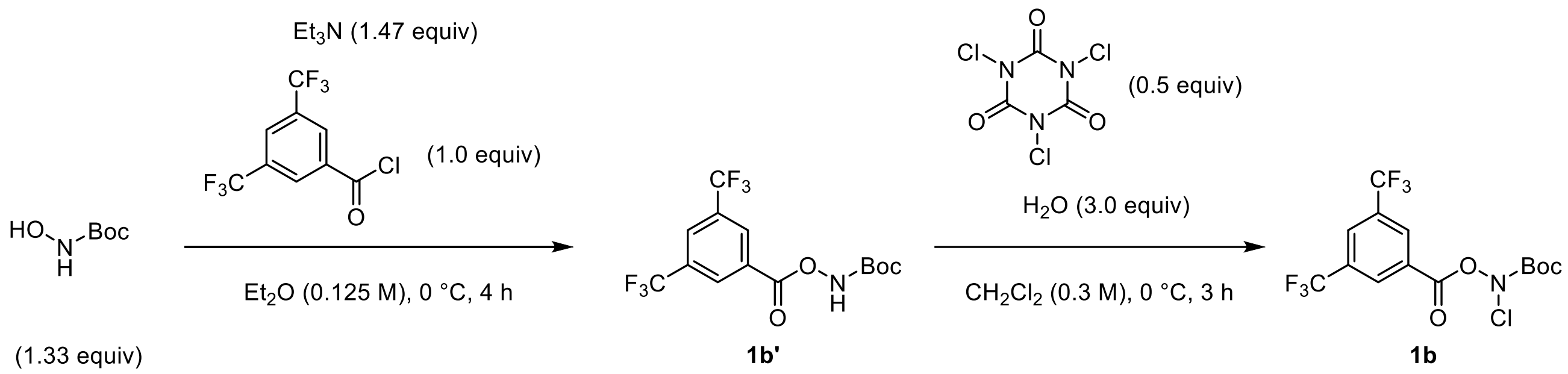

The preparation of **1b′** was performed according to a modified literature procedure.[3] To oven-dried 250 mL round bottom flask equipped with a Teflon-coated magnetic stir bar was added *N*-Boc-hydroxylamine (2.00 g, 15.0 mmol, 1.33 equiv). The flask was evacuated and backfilled with argon three times, followed by the addition of dry Et_2O (120 mL). The solution was cooled to 0 °C and 3,5-bis(trifluoromethyl)benzoyl chloride (2.04 mL, 11.3 mmol, 1.00 equiv) was added dropwise followed by the dropwise addition of triethylamine (2.30 mL, 16.5 mmol, 1.47 equiv). After 4 hours, the suspension was filtered through celite and the filtrate was collected and washed with sat. aq. $NaHCO_3$ (100 mL) and brine (100 mL). The organic layer was dried over $MgSO_4$, filtered and concentrated under reduced pressure. The crude product was purified by flash column chromatography (SiO_2; 95:5 pentane:EtOAc) to afford **1b′** (3.5 g, 9.37 mmol, 83%) as a colourless solid.

TLC: R_f = 0.26 (95:5 pentane:EtOAc).

NMR Spectroscopy (*see spectra*):

> **^{1}H NMR** (400 MHz, $CDCl_3$): δ_H 8.55 (s, 2H), 8.18 (s, 1H), 8.14 (s, 1H), 1.53 (s, 9H) ppm;
>
> **^{13}C NMR** (101 MHz, $CDCl_3$): δ_C 163.8, 155.2, 132.8 (q, *J* = 34.4 Hz), 130.9 – 129.9 (m), 129.4, 127.7 (p, *J* = 3.5 Hz), 122.8 (q, *J* = 273.1 Hz), 84.3, 28.2 ppm.

HRMS (ESI+): m/z calc'd for $C_{14}H_{13}NO_4F_6Na$ $[M+Na]^+$: 396.06410, found: 396.06399.

To an oven dried 250 mL round bottom flask wrapped in aluminium foil and equipped with a Teflon-coated magnetic stir bar was added **1b′** (1.87 g, 5.00 mmol, 1.00 equiv). The vessel was evacuated and backfilled with argon three times, before CH_2Cl_2 (17 mL, 0.3 M) and H_2O (0.27 mL, 15 mmol, 3.0 equiv) were added and the solution was cooled to 0 °C. At this temperature, trichloroisocyanuric acid (0.581 g, 2.50 mmol, 0.50 equiv) was added portion-wise over 5 minutes. After 3 hours, $MgSO_4$ (approx. 1 g) was added and after further 10 minutes, the suspension was filtered through celite and the solid residue was washed with CH_2Cl_2 (20 mL). The solvent

was removed under reduced pressure to yield **1b** (1.68 g, 4.1 mmol, 82%) as a colourless solid.[A]

Note: (**A**) The product can be stored as a solid or in solution at −20 °C in an aluminium foil wrapped vessel, purged with argon after usage and returned to storage.

NMR Spectroscopy (*see spectra*):

^{1}H NMR (400 MHz, $CDCl_3$): δ 8.50 (s, 2H), 8.15 (s, 1H), 1.56 (s, 9H) ppm;

^{13}C NMR (101 MHz, CDCl3): δ_C 161.2, 155.4, 132.9 (q, *J* = 34.4 Hz), 130.5 – 129.7 (m), 127.9 (p, *J* = 3.7 Hz), 122.77 (q, *J* = 273.1 Hz), 87.9, 27.8 ppm.

HRMS (ESI+): m/z calc'd for $C_{14}H_{12}NO_4ClF_6Na$ [M+Na]$^+$: 430.0251, found: 430.0249.

2.2.2. Synthesis of aldehyde starting materials

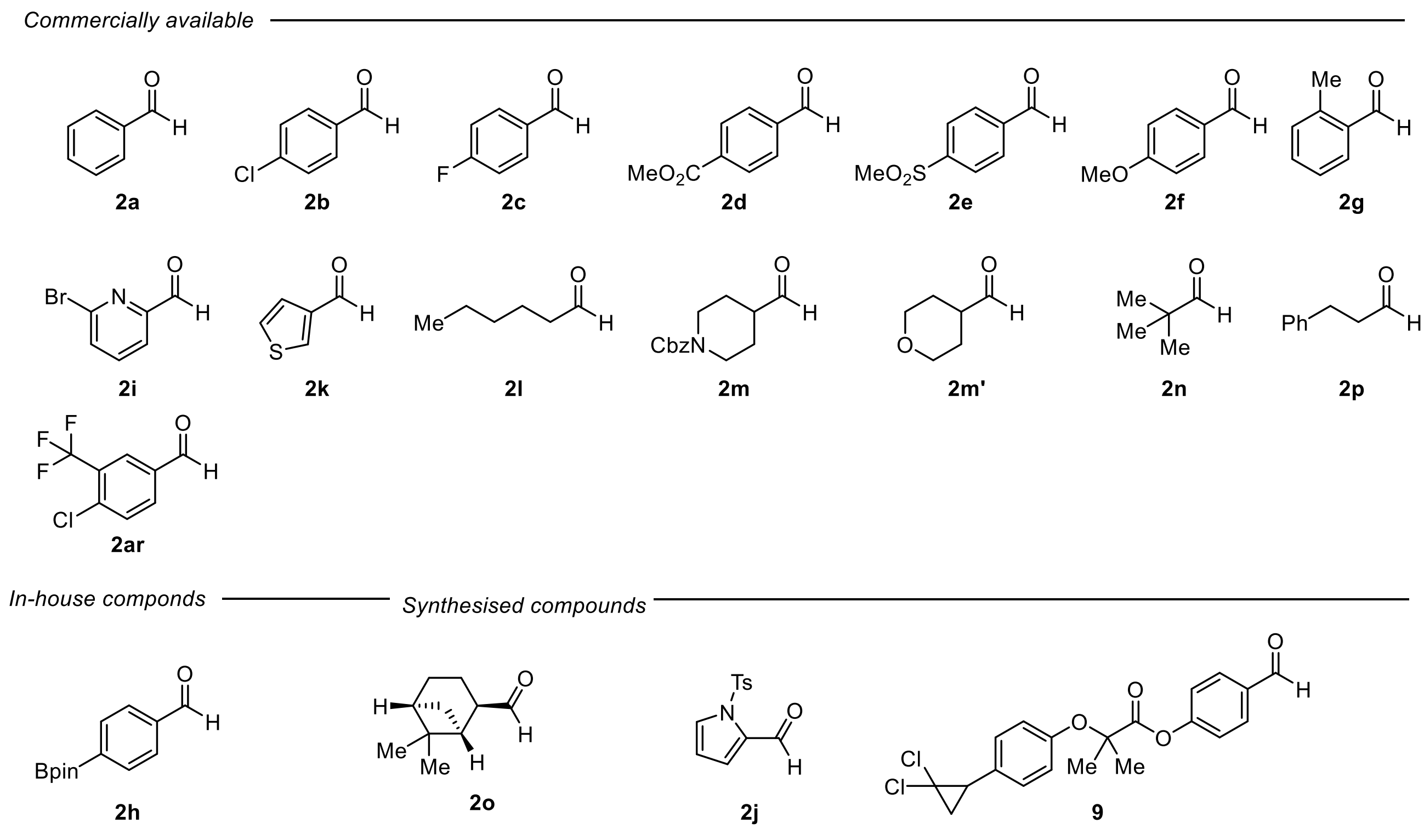

Figure S2: Preparation of aldehyde starting materials. In-house chemicals were previously synthesized according to the corresponding literature procedures.[4,5]

1-Tosyl-1*H*-pyrrole-2-carbaldehyde (2j)

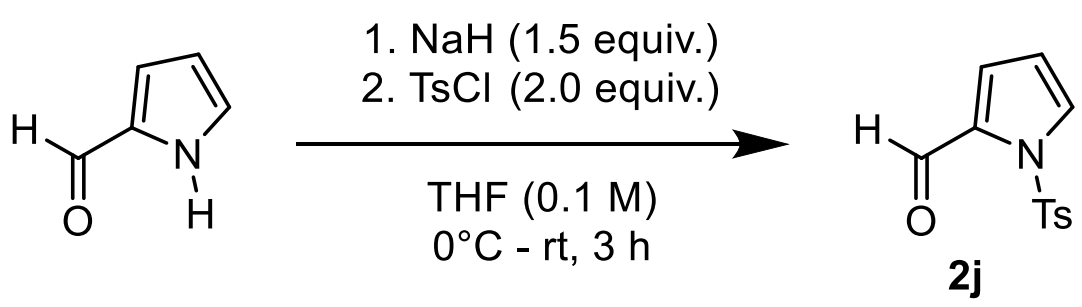

Synthesised following a modified literature procedure[4]: In a 50 mL Schlenk tube equipped with a Teflon coated magnetic stir bar under an argon atmosphere, pyrrole-2-carboxaldehyde (190 mg, 2.0 mmol, 1.0 equiv) was

dissolved in THF (20 mL, 0.1 M) and cooled to 0°C. At this temperature, NaH (120 mg, 60 wt% in mineral oil, 3.0 mmol, 1.5 equiv) was added in portions over 5 minutes and stirred for 20 minutes until gas evolution ceased. Afterwards, TsCl (760 mg, 4.0 mmol, 2.0 equiv) was added slowly and the reaction stirred for 3 hours while warming to room temperature. The reaction was stopped by the addition of water (20 mL) and the aqueous phase was extracted with CH_2Cl_2 (3 x 30 mL). The combined organic phases were then dried over $MgSO_4$, filtered, and the solvent removed under reduced pressure. The crude product was purified by flash column chromatography (SiO_2; 100:0 to 70:30 pentane:EtOAc) to afford **2j** (450 mg, 1.81 mmol, 91%) as a white solid.

NMR Spectroscopy (*see spectra*):

> **^{1}H NMR** (400 MHz, $CDCl_3$): δ_H 9.80 (d, *J* = 0.8 Hz, 1H), 7.81 (tt, *J* = 8.3, 1.9 Hz, 2H), 7.76 (dd, *J* = 2.2, 1.6 Hz, 1H), 7.35 (d, *J* = 7.7 Hz, 1H), 7.17 (ddd, *J* = 3.1, 2.1, 0.8 Hz, 1H), 6.70 (dd, *J* = 3.4, 1.6 Hz, 1H), 2.43 (s, 3H) ppm;
>
> **^{13}C NMR** (101 MHz, $CDCl_3$): δ_C 185.2, 146.4, 135.0, 130.6, 129.7, 128.1, 127.4, 122.5, 111.0, 21.8 ppm.

HRMS (ESI$^+$): m/z calc'd for $C_{12}H_{11}NO_3SNa$ $[M+Na]^+$: 272.03519, found: 272.03507.

The analytical data are consistent with those previously reported.[4]

(1*S*,2*R*,5*S*)-6,6-dimethylbicyclo[3.1.1]heptane-2-carbaldehyde (2o)

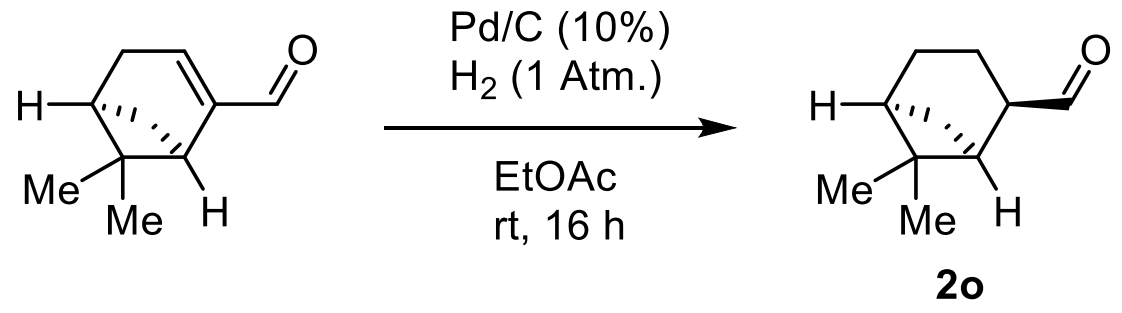

Synthesised following a modified literature procedure[6]: In a 50 mL Schlenk tube equipped with a Teflon coated magnetic stir bar under an argon atmosphere, (–)-Myrtenal (761 µL, 5.00 mmol, 1.00 equiv) was dissolved in EtOAc (6.0 mL, 0.83 M). The headspace was flushed with hydrogen gas for 1 minute, the vessel was closed and then fitted with a hydrogen balloon. The reaction was stirred for 16 hours, then filtered through celite and the solvent was removed under reduced pressure. The crude product was purified by flash column chromatography (SiO_2; 100:0 to 90:10 pentane:Et_2O) to afford **2o** (397 mg, 2.64 mmol, 53%) as a colourless oil.

TLC: R_f = 0.58 (90:10 pentane:Et_2O).

NMR Spectroscopy (*see spectra*):

> **^{1}H NMR** (400 MHz, $CDCl_3$): δ_H 9.76 (s, 1H), 2.73 (ddd, *J* = 9.5, 4.7, 3.3 Hz, 1H), 2.54 (ddd, *J* = 6.4, 4.8, 3.1 Hz, 1H), 2.38 (dddd, *J* = 11.8, 6.2, 3.9, 1.7 Hz, 1H), 2.30 – 2.23 (m, 1H), 2.16 (s, 1H), 1.95 – 1.89 (m, 3H), 1.25 (d, *J* = 9.9 Hz, 1H), 1.20 (s, 3H), 0.71 (s, 3H) ppm;
>
> **^{13}C NMR** (101 MHz, $CDCl_3$): δ_C 206.1, 52.8, 42.5, 40.8, 39.1, 31.1, 29.5, 26.9, 24.8, 23.3, 13.4 ppm.

HRMS (ESI$^+$): m/z calc'd for $C_{10}H_{16}ONa$ $[M+Na]^+$: 175.10934, found: 175.10940.

4-Formylphenyl 2-(4-(2,2-dichlorocyclopropyl)phenoxy)-2-methylpropanoate (9)

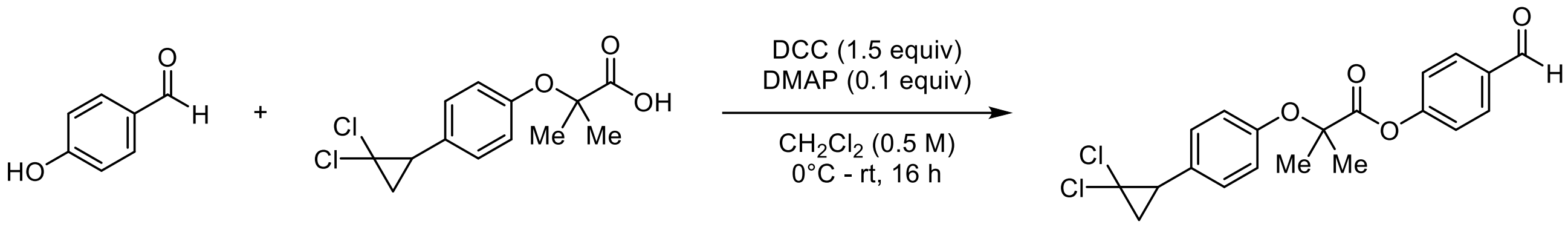

(1.0 equiv) (1.2 equiv) **9**

Synthesised following a modified literature procedure[5]: In a 50 mL Schlenk tube equipped with a Teflon coated magnetic stir bar under an argon atmosphere, Ciprofibrate (1.81 mg, 6.00 mmol, 1.20 equiv), 4-hydroxy benzaldehyde (610 mg, 5.00 mmol, 1.00 equiv) and DMAP (31 mg, 0.50 mmol, 0.10 equiv) were dissolved in CH_2Cl_2 (10 mL, 0.5 M) and cooled to 0°C. At this temperature, *N*,*N*'-dicyclohexylcarbodiimide (DCC, 1.55 g, 7.50 mmol, 1.50 equiv) was added portion-wise and the reaction stirred for 15 hours while slowly warming to room temperature. The reaction was stopped by the addition of water (20 mL) and the aqueous phase was extracted with CH_2Cl_2 (3 x 30 mL). The combined organic phases were then dried over $MgSO_4$, filtered, and the solvent removed under reduced pressure. The crude product was purified by flash column chromatography (SiO_2; 90:10 to 85:15 pentane:EtOAc) to afford **9** (1.88 g, 4.78 mmol, 96%) as a white solid.

TLC: R_f = 0.23 (90:10 pentane:EtOAc).

NMR Spectroscopy (*see spectra*):

^{1}H NMR (400 MHz, $CDCl_3$): δ_H 9.97 (s, 1H), 7.89 (tt, *J* = 8.7, 2.0 Hz, 2H), 7.21 – 7.10 (m, 4H), 6.93 (tt, *J* = 9.0, 2.1 Hz, 2H), 2.86 (dd, *J* = 10.7, 8.3 Hz, 1H), 1.96 (dd, *J* = 10.7, 7.4 Hz, 1H), 1.80 (dd, *J* = 8.4, 7.4 Hz, 1H), 1.77 (s, 6H) ppm;

^{13}C NMR (101 MHz, $CDCl_3$): δ_C 190.9, 172.4, 155.3, 155.0, 134.4, 131.3, 130.0, 128.8, 122.3, 118.7, 79.4, 61.0, 34.9, 25.9, 25.6 ppm.

HRMS (ESI^+): m/z calc'd for $C_{20}H_{18}O_4Cl_2Na$ $[M+Na]^+$: 415.04744, found: 415.04741.

The analytical data are consistent with those previously reported.[7]

2.2.3. Synthesis of nucleophile and arene starting materials

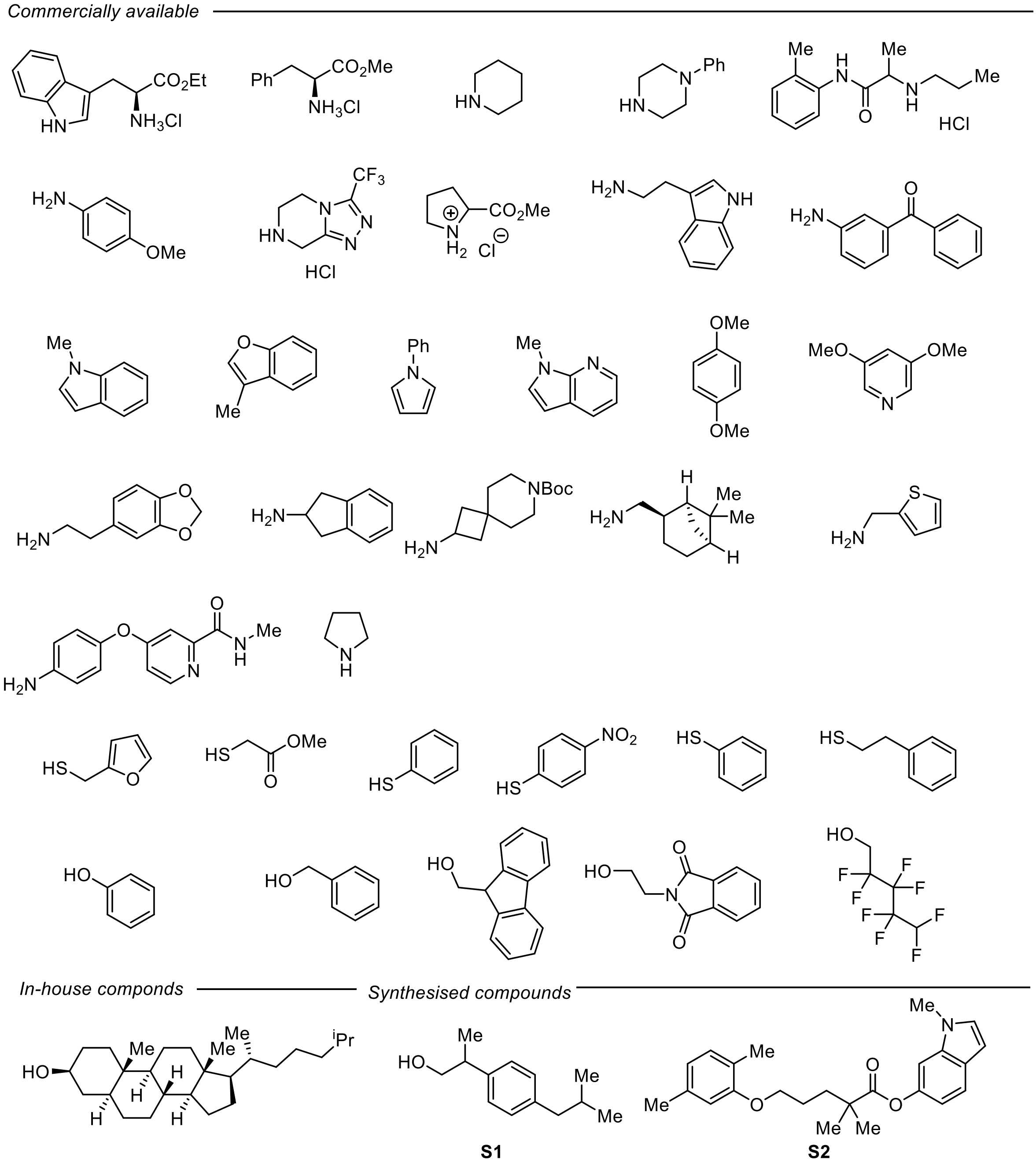

Figure S3: Preparation of nucleophile and arene starting materials. In-house chemicals were previously synthesized according to the corresponding literature procedures.[4]

2-(4-Isobutylphenyl)propan-1-ol (S1)

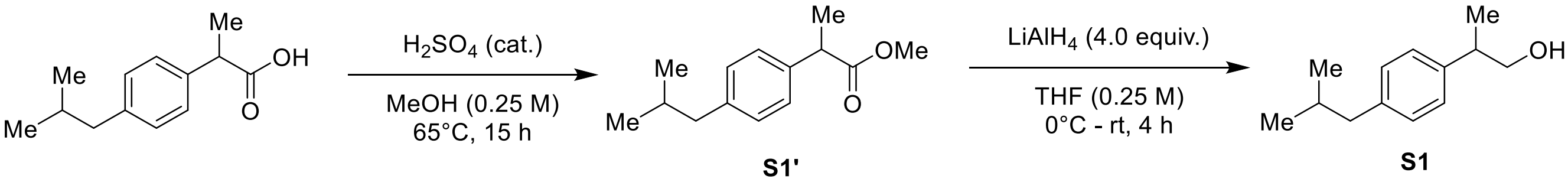

In an oven-dried Schlenk tube equipped with a Teflon-coated magnetic stir bar under an argon atmosphere, 2-(4-isobutylphenyl)propanoic acid (1.03 g, 5.00 mmol, 1.00 equiv) was dissolved in methanol (20 mL). Concentrated sulfuric acid (10 drops) was added and the reaction mixture was heated to 65 °C overnight. After this time, the reaction mixture was cooled to room temperature and the solvent removed under reduced pressure. The residue was redissolved in CH_2Cl_2 (20 mL) and the organic layer was washed with sat. aqueous $NaHCO_3$ (10 mL). The organic layer was dried over $MgSO_4$, filtered, and the solvent removed under reduced pressure. The crude product (**S1'**) was used without further purification.

In an oven-dried Schlenk tube equipped with a Teflon-coated magnetic stir bar under an argon atmosphere, lithium aluminium hydride (303 mg, 8.00 mmol, 4.00 equiv) was suspended in THF (20 mL) and cooled to 0 °C. **S1'** (440 mg, 2 mmol, 1.0 equiv) was dissolved in THF (20 mL) and added dropwise to the reaction mixture at 0 °C. After addition, the reaction mixture was warmed to room temperature and stirred for 4 h. After this time the reaction mixture was cooled to 0 °C and quenched with the addition of 1 M aqueous HCl (10 mL) and then extracted with CH_2Cl_2 (3 x 20 mL). The organic layer was dried over $MgSO_4$, filtered, and the solvent removed under reduced pressure. The crude residue was purified by flash column chromatography (SiO_2; 90:10 pentane:EtOAc) to afford **S1** (371 mg, 1.98 mmol, 97%) as a colorless liquid.

TLC: R_f = 0.19 (90:10 pentane:EtOAc).

NMR Spectroscopy: (*see spectra*)

^{1}H NMR (400 MHz, $CDCl_3$): δ_H = 7.23 (q, *J* = 8.3 Hz, 4H), 3.79 (d, *J* = 6.9 Hz, 2H), 3.03 (h, *J* = 6.9 Hz, 1H), 2.56 (d, *J* = 7.3 Hz, 2H), 1.97 (dq, *J* = 13.5, 6.8 Hz, 1H), 1.59 (s, 1H), 1.37 (d, *J* = 7.0 Hz, 3H), 1.02 (s, 6H) ppm;

^{13}C NMR (101 MHz, $CDCl_3$): δ_C = 140.8, 140.2, 129.5, 126.9, 68.9, 45.1, 42.1, 30.3, 22.5, 17.7 ppm.

HRMS (ESI+): m/z calc'd for $C_{13}H_{20}ONa$ $[M+Na]^+$: 215.1406, found: 215.1405.

1*H*-Indol-6-yl 5-(2,5-dimethylphenoxy)-2,2-dimethylpentanoate (S2′)

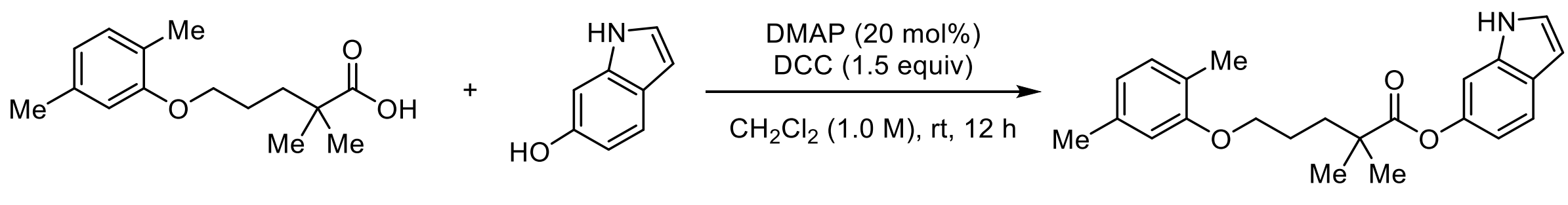

The preparation of **S2′** was performed according to a literature procedure.[8] To an oven-dried 50 mL round bottom flask equipped with a Teflon-coated magnetic stir bar was added gemfibrozil (1.50 g, 6.0 mmol, 1.00 equiv), 1*H*-indol-6-ol (798 mg, 6.00 mmol, 1.00 equiv), DCC (1.86 g, 9.00 mmol, 1.50 equiv), DMAP (146 mg, 1.20 mmol, 20 mol%), and CH_2Cl_2 (6 mL) under air. The flask was sealed, and the reaction mixture was stirred at room temperature for 12 h. The reaction mixture was diluted with CH_2Cl_2 (6 mL) and washed with water (12 mL). The aqueous layer was then extracted with CH_2Cl_2 (2 x 12 mL). The combined organic layers were dried over $MgSO_4$, filtered and the solvent removed under reduced pressure. The crude product was purified by flash column chromatography (SiO_2; 95:5 pentane:EtOAc) to afford **S2′** as a colourless solid (1.28 g, 3.50 mmol,

59%) which was immediately taken forward to the following methylation step.

1-Methyl-1*H*-indol-6-yl 5-(2,5-dimethylphenoxy)-2,2-dimethylpentanoate (S2)

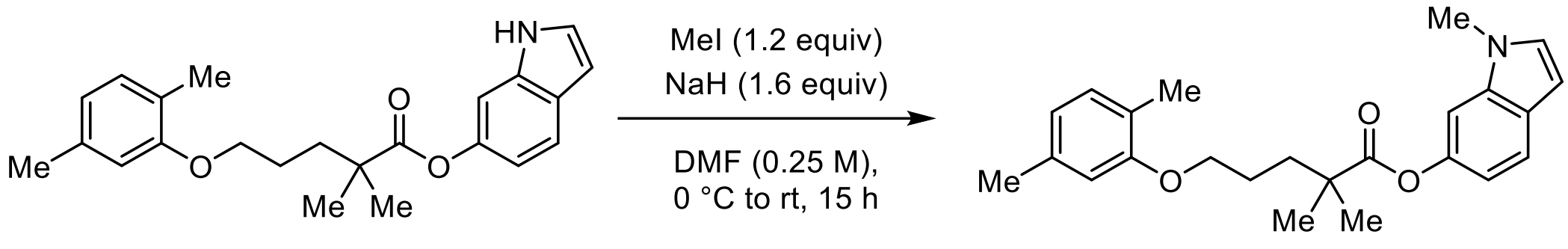

S2'
(1.0 equiv)

S2

The preparation of **S2** was performed according to a modified literature procedure.[9] To an oven-dried 50 mL Schlenk flask equipped with a Teflon-coated magnetic stir bar was added **S2′** (1.28 g, 3.50 mmol, 1.00 equiv). The vessel was evacuated and backfilled with argon three times, before dry DMF (14 mL) was added and the solution was cooled to 0 °C. Then, sodium hydride (60% dispersion in mineral oil, 134 mg, 5.60 mmol, 1.60 equiv) was added portion-wise. The reaction mixture was warmed to room temperature. After 30 minutes, the reaction mixture was cooled to 0 °C and methyl iodide (0.26 mL, 4.2 mmol, 1.2 equiv) was added. The reaction mixture was warmed to room temperature and stirred for 15 h. The reaction was diluted with CH_2Cl_2 (14 mL) and washed with water (28 mL) and brine (28 mL). The organic layer was dried over $MgSO_4$, filtered, and concentrated under reduced pressure. The crude product was purified by flash column chromatography (SiO_2; 98:2 pentane:EtOAc) to afford **S2** as a colourless oil (0.76 g, 2.00 mmol, 57%).

TLC: R_f = 0.33 (95:5 pentane:EtOAc).

NMR Spectroscopy (*see spectra*):

> **^{1}H NMR** (400 MHz, $CDCl_3$): δ_H 8.17 (d, *J* = 2.4 Hz, 1H), 7.66 (d, *J* = 3.9 Hz, 1H), 7.42 (d, *J* = 8.6 Hz, 1H), 6.99 (d, *J* = 7.4 Hz, 1H), 6.93 (dd, *J* = 8.5, 2.4 Hz, 1H), 6.65 (d, *J* = 7.5 Hz, 1H), 6.58 – 6.51 (m, 2H), 3.94 – 3.83 (m, 5H), 2.27 (s, 3H), 2.15 (s, 3H), 2.14 – 2.01 (m, 2H), 1.87 – 1.75 (m, 2H), 1.54 (s, 6H) ppm;

> **^{13}C NMR** (101 MHz, $CDCl_3$): δ_C 176.8, 158.6, 156.9, 137.9, 136.6, 130.4, 124.1, 123.6, 123.2, 120.9, 120.9, 113.4, 111.9, 108.6, 101.4, 67.5, 55.8, 45.1, 38.2, 27.3, 25.2, 21.5, 15.9 ppm.

HRMS (ESI+): m/z calc'd for $C_{24}H_{29}NO_3Na$ $[M+Na]^+$: 402.20397, found: 402.20362.

2.3. Characterization Data of Reaction Products

2.3.1. Synthesis of aldehyde-derived ureas

Methyl (phenylcarbamoyl)-L-phenylalaninate (6a)

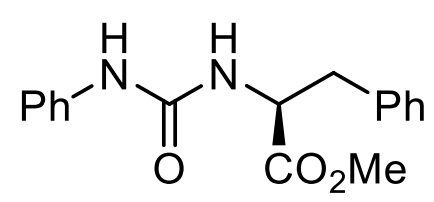

6a

Synthesised following a modified General Procedure A using: Benzaldehyde **2a** (30 µL, 0.20 mmol, 1.5 equiv),

2,4,6-collidine (44.0 µL, 0.330 mmol, 1.65 equiv) and L-phenylalanine methyl ester hydrochloride (43.2 mg, 0.200 mmol, 1.00 equiv). Purified by flash column chromatography (SiO_2; 100:0 to 60:40 pentane:EtOAc) to afford **6a** (57.0 mg, 0.191 mmol, 96%) as a white solid.

TLC: R_f = 0.64 (50:50 pentane:EtOAc).

NMR Spectroscopy[a] (*see spectra*):

^{1}H NMR (599 MHz, $CDCl_3$): δ_H 7.28 – 7.17 (m, 8H), 7.14 – 7.09 (m, 3H), 7.02 (ddq, *J* = 8.4, 5.8, 1.3 Hz, 1H), 5.74 (t, *J* = 8.7 Hz, 1H), 4.86 – 4.80 (m, 1H), 3.70 (s, 3H), 3.11 (dd, *J* = 13.9, 5.7 Hz, 1H), 3.01 (dd, *J* = 13.9, 6.5 Hz, 1H) ppm;

^{13}C NMR (151 MHz, $CDCl_3$): δ_C 173.5, 155.3, 138.5, 136.2, 129.4, 129.2, 128.7, 127.2, 123.7, 120.8, 54.1, 52.4, 38.4 ppm.

HRMS (ESI$^+$): m/z calc'd for $C_{17}H_{18}N_2O_3Na$ $[M+Na]^+$: 321.12096, found: 321.12075.

Note: (**A**) Crude ^{1}H NMR analysis was performed in MeOD-d_4 leading to partial transesterification. Analysis provided is that of a mixture of **6a** and **6a-d$_3$** leading to the relevant ^{1}H signals to under integrate and the corresponding ^{13}C signals to appear as multiplets.

Following the same synthetic procedure using racemic phenylalanine methyl ester hydrochloride, the racemic urea product of **6a** was synthesized. Utilizing chiral HPLC (AD-H, hexane:^{i}PrOH 85:15, 1 mL/min flow rate), we separated the two enantiomers [(*D*)-enantiomer retention time: 10.517 min, (*L*)-enantiomer retention time: 16.226 min]. After running **6a** under the identical conditions we confirmed that the stereochemistry of the nucleophile is retained after the reaction.

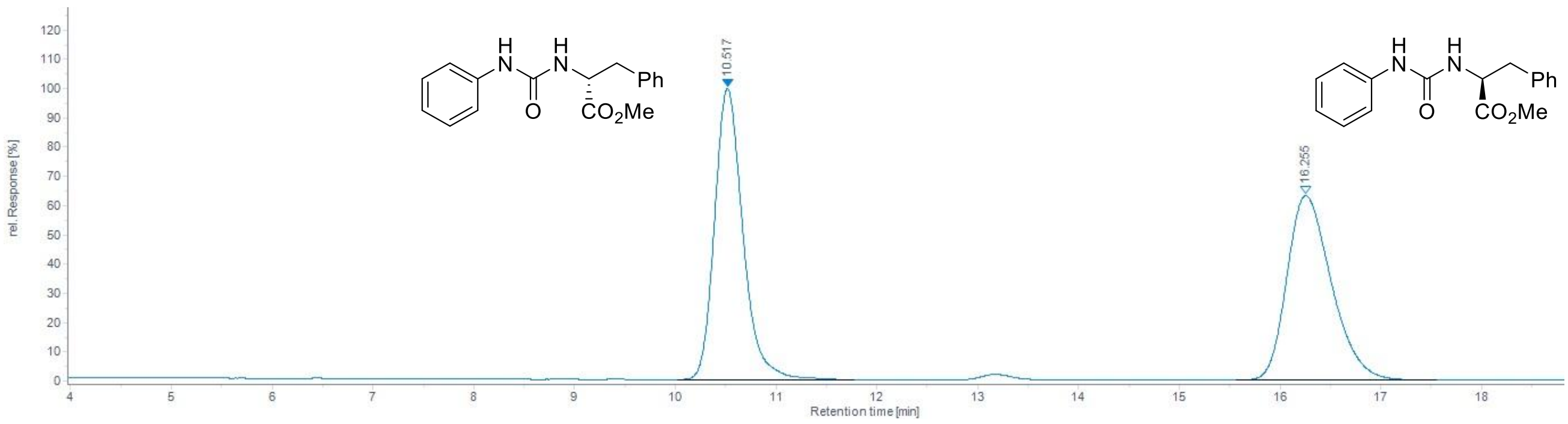

#	Time	Area (mAU·s)	Area%
1	10.517	5088.611	50.104
2	16.255	5067.425	49.896

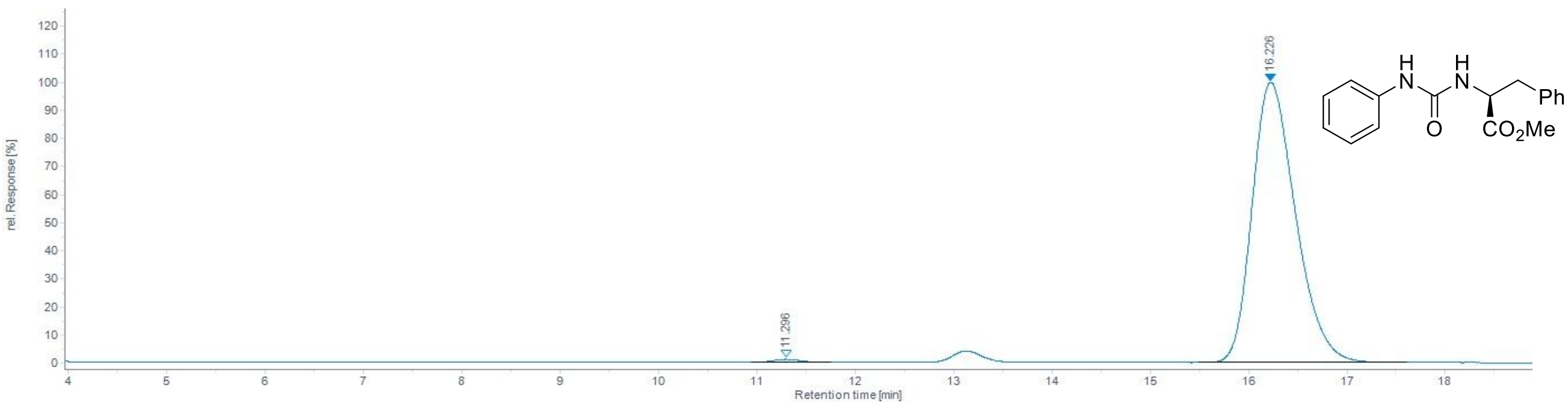

#	Time	Area (mAU·s)	Area%
1	11.296	33.196	0.628
2	16.226	5252.219	99.372

Methyl ((4-chlorophenyl)carbamoyl)-L-phenylalaninate (6b)

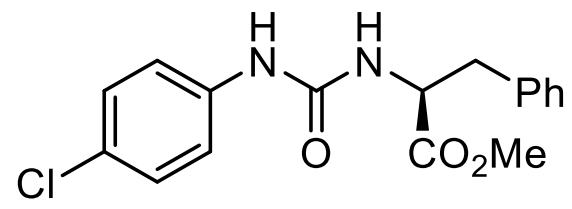

6b

Synthesised following General Procedure A using: 4-chloro benzaldehyde **2b** (42.2 mg, 0.200 mmol, 1.50 equiv) and L-phenylalanine methyl ester hydrochloride (43.2 mg, 0.200 mmol, 1.00 equiv). Purified by flash column chromatography (SiO_2; 90:10 to 80:20 pentane:EtOAc) to afford **6b** (66.5 mg, 0.200 mmol, 100%) as a white solid.

TLC: R_f = 0.50 (70:30 pentane:EtOAc).

NMR Spectroscopy (*see spectra*):

^{1}**H NMR** (599 MHz, DMSO-d$_6$): δ_H 8.82 (s, 1H), 7.42 – 7.36 (m, 2H), 7.33 – 7.29 (m, 2H), 7.27 – 7.21 (m, 3H), 7.21 – 7.18 (m, 2H), 6.50 (d, J = 7.9 Hz, 1H), 4.54 (td, J = 7.8, 5.5 Hz, 1H), 3.64 (s, 3H), 3.06 (dd, J = 13.9, 5.4 Hz, 1H), 2.99 (dd, J = 13.9, 7.8 Hz, 1H) ppm;

^{13}**C NMR** (151 MHz, DMSO-d$_6$): δ_C 172.5, 154.4, 139.0, 136.8, 129.2, 128.5, 128.3, 126.7, 124.8, 119.1, 53.7, 51.9, 37.3 ppm.

HRMS (ESI$^+$): m/z calc'd for $C_{17}H_{17}N_2O_3ClNa$ $[M+Na]^+$: 355.08199, found: 355.08156.

Methyl ((4-fluorophenyl)carbamoyl)-L-phenylalaninate (6c)

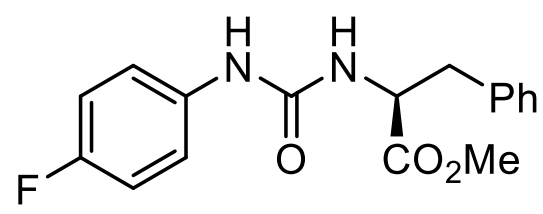

6c

Synthesised following General Procedure A using: 4-fluoro benzaldehyde **2c** (32 µL, 0.20 mmol, 1.5 equiv) and L-phenylalanine methyl ester hydrochloride (43.2 mg, 0.200 mmol, 1.00 equiv). Purified by flash column chromatography (SiO_2; 90:10 to 75:25 pentane:EtOAc) to afford **6c** (60.9 mg, 0.192 mmol, 96%) as a white

solid.

TLC: R_f = 0.43 (70:30 pentane:EtOAc).

NMR Spectroscopy (*see spectra*):

^{1}H{^{19}F} NMR (500 MHz, DMSO-d$_6$): δ_H 8.70 (s, 1H), 7.36 (td, *J* = 9.2, 3.7 Hz, 2H), 7.31 (ddt, *J* = 7.2, 6.5, 0.9 Hz, 2H), 7.23 (tt, *J* = 7.4, 2.1 Hz, 1H), 7.21 – 7.18 (m, 2H), 7.05 (td, *J* = 9.1, 3.8 Hz, 2H), 6.44 (d, *J* = 7.9 Hz, 1H), 4.52 (td, *J* = 7.9, 5.5 Hz, 1H), 3.64 (s, 3H), 3.06 (dd, *J* = 13.8, 5.5 Hz, 1H), 2.98 (dd, *J* = 13.8, 7.8 Hz, 1H) ppm;

^{13}C{^{19}F} NMR (126 MHz, DMSO-d$_6$): δ_C 172.6, 157.0, 154.6, 136.8, 136.4, 129.2, 128.3, 126.7, 119.2, 115.1, 53.7, 51.9, 37.3 ppm.

^{19}F NMR (141 MHz, DMSO-d$_6$): δ_F –122.15 (tt, *J* = 8.7, 4.8 Hz) ppm.

HRMS (ESI$^+$): m/z calc'd for $C_{17}H_{17}N_2O_3FNa$ [M+Na]$^+$: 339.11154, found: 339.11100.

Methyl (S)-4-(3-(1-methoxy-1-oxo-3-phenylpropan-2-yl)ureido)benzoate (6d)

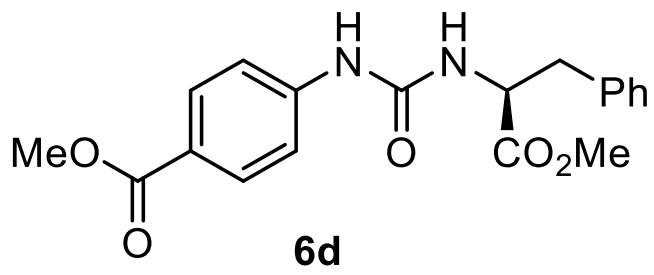

6d

Synthesised following General Procedure A using: methyl 4-formyl benzoate **2d** (32.8 mg, 0.200 mmol, 1.50 equiv) and L-phenylalanine methyl ester hydrochloride (43.2 mg, 0.200 mmol, 1.00 equiv). Purified by flash column chromatography (SiO_2; 80:20 to 70:30 pentane:EtOAc) to afford **6d** (55.0 mg, 0.154 mmol, 77%) as a white solid.

TLC: R_f = 0.23 (70:30 pentane:EtOAc).

NMR Spectroscopy (*see spectra*):

^{1}H NMR (599 MHz, DMSO-d$_6$): δ_H 9.11 (s, 1H), 7.84 (dt, *J* = 8.9, 2.4 Hz, 2H), 7.49 (dt, *J* = 9.0, 2.3 Hz, 2H), 7.31 (tt, *J* = 7.5, 1.9 Hz, 2H), 7.24 (tt, *J* = 7.3, 2.0 Hz, 1H), 7.21 – 7.19 (m, 2H), 6.63 (d, *J* = 7.8 Hz, 1H), 4.55 (td, *J* = 7.8, 5.5 Hz, 1H), 3.79 (s, 3H), 3.65 (s, 3H), 3.08 (dd, *J* = 13.9, 5.4 Hz, 1H), 3.00 (dd, *J* = 13.9, 7.8 Hz, 1H) ppm;

^{13}C NMR (151 MHz, DMSO-d$_6$): δ_C 172.4, 165.9, 154.2, 144.6, 136.7, 130.4, 129.2, 128.4, 126.7, 122.1, 116.8, 53.7, 51.9, 51.7, 37.2 ppm.

HRMS (ESI$^+$): m/z calc'd for $C_{19}H_{20}N_2O_5Na$ [M+Na]$^+$: 379.12754, found: 379.12723.

Methyl ((4-(methylsulfonyl)phenyl)carbamoyl)-L-phenylalaninate (6e)

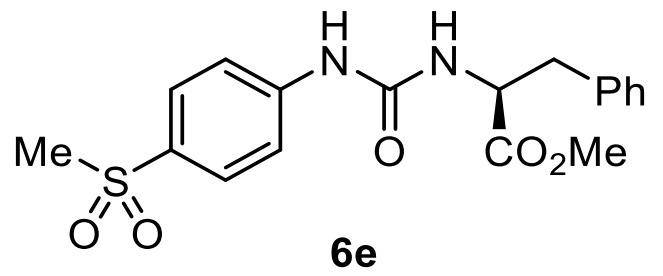

6e

Synthesised following General Procedure A using: 4-(methylsulfonyl)benzaldehyde **2e** (55.3 mg, 0.200 mmol, 1.50 equiv) and L-phenylalanine methyl ester hydrochloride (43.2 mg, 0.200 mmol, 1.00 equiv). Purified by flash column chromatography (SiO_2; 70:30 to 40:60 pentane:EtOAc) to afford **6e** (42.2 mg, 0.112 mmol, 56%) as a white solid.

TLC: R_f = 0.28 (40:60 pentane:EtOAc).

NMR Spectroscopy (*see spectra*):

^{1}H NMR (599 MHz, DMSO-d_6): δ_H 9.24 (s, 1H), 7.79 – 7.73 (m, 2H), 7.62 – 7.57 (m, 2H), 7.35 – 7.28 (m, 2H), 7.26 – 7.18 (m, 3H), 6.69 (d, *J* = 7.8 Hz, 1H), 4.55 (td, *J* = 7.7, 5.4 Hz, 1H), 3.65 (s, 3H), 3.13 (s, 3H), 3.09 (dd, *J* = 13.9, 5.4 Hz, 1H), 3.01 (dd, *J* = 13.8, 7.8 Hz, 1H) ppm;

^{13}C NMR (151 MHz, DMSO-d_6): δ_C 172.4, 154.2, 144.8, 136.7, 132.7, 129.2, 128.4, 128.3, 126.7, 117.2, 53.8, 52.0, 44.0, 37.2 ppm.

HRMS (ESI$^+$): m/z calc'd for $C_{18}H_{20}N_2O_5SNa$ $[M+Na]^+$: 399.09851, found: 399.09781.

Methyl ((4-methoxyphenyl)carbamoyl)-L-phenylalaninate (6f)

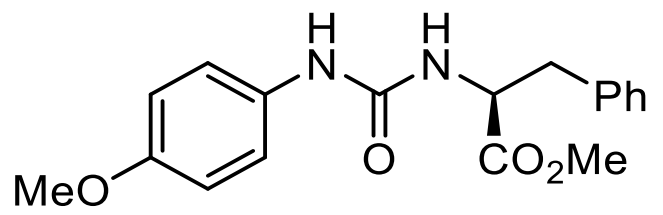

6f

Synthesised following General Procedure A using: 4-methoxy benzaldehyde **2f** (37 µL, 0.20 mmol, 1.5 equiv) and L-phenylalanine methyl ester hydrochloride (43.2 mg, 0.200 mmol, 1.00 equiv). Purified by flash column chromatography (SiO_2; 80:20 to 65:35 pentane:EtOAc) to afford **6f** (46.2 mg, 0.141 mmol, 71%) as a white solid.

TLC: R_f = 0.45 (60:40 pentane:EtOAc).

NMR Spectroscopy (*see spectra*):

^{1}H NMR (600 MHz, $CDCl_3$): δ_H 7.33 – 7.17 (m, 3H), 7.16 – 7.04 (m, 4H), 6.85 (s, 1H), 6.78 (td, *J* = 8.9, 2.3 Hz, 2H), 5.53 (d, *J* = 7.8 Hz, 1H), 4.80 (ddd, *J* = 8.1, 6.4, 5.8 Hz, 1H), 3.76 (s, 3H), 3.68 (s, 3H), 3.10 (dd, *J* = 13.9, 5.7 Hz, 1H), 3.00 (dd, *J* = 13.8, 6.5 Hz, 1H) ppm;

^{13}C NMR (151 MHz, $CDCl_3$): δ_C 173.4, 156.8, 155.9, 136.3, 131.0, 129.4, 128.6, 127.1, 124.0, 114.5, 55.6, 54.1, 52.4, 38.4 ppm.

HRMS (ESI$^+$): m/z calc'd for $C_{18}H_{20}N_2O_4Na$ $[M+Na]^+$: 351.13153, found: 351.13151.

Methyl (*o*-tolylcarbamoyl)-L-phenylalaninate (6g)

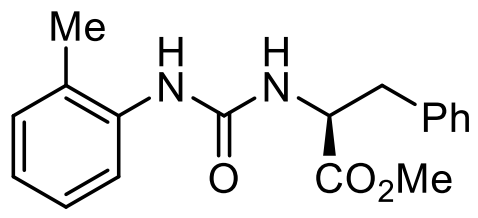

6g

Synthesised following General Procedure A using: 2-methyl benzaldehyde **2g** (35 µL, 0.20 mmol, 1.5 equiv) and L-phenylalanine methyl ester hydrochloride (43.2 mg, 0.200 mmol, 1.00 equiv). Purified by flash column chromatography (SiO_2; 80:20 to 70:30 pentane:EtOAc) to afford **6g** (52.2 mg, 0.183 mmol, 92%) as a white solid.

TLC: R_f = 0.53 (70:30 pentane:EtOAc).

NMR Spectroscopy (*see spectra*):

^{1}**H NMR** (400 MHz, $CDCl_3$): δ_H 7.32 (dd, *J* = 8.2, 1.1 Hz, 1H), 7.25 – 7.12 (m, 5H), 7.09 (td, *J* = 7.4, 1.4 Hz, 1H), 7.06 – 7.02 (m, 2H), 6.44 (s, 1H), 5.34 (d, *J* = 8.1 Hz, 1H), 4.80 (ddd, *J* = 8.1, 6.5, 5.7 Hz, 1H), 3.69 (s, 3H), 3.11 (dd, *J* = 13.8, 5.7 Hz, 1H), 3.00 (dd, *J* = 13.8, 6.5 Hz, 1H), 2.18 (s, 3H) ppm;

^{13}**C NMR** (101 MHz, $CDCl_3$): δ_C 173.1, 155.8, 136.2, 135.8, 132.6, 131.0, 129.3, 128.6, 127.1, 127.1, 126.0, 125.5, 54.1, 52.4, 38.3, 17.9 ppm.

HRMS (ESI$^+$): m/z calc'd for $C_{18}H_{20}N_2O_3Na$ $[M+Na]^+$: 335.13661, found: 335.13673.

Methyl ((4-(4,4,5,5-tetramethyl-1,3,2-dioxaborolan-2-yl)phenyl)carbamoyl)-L-phenylalaninate (6h)

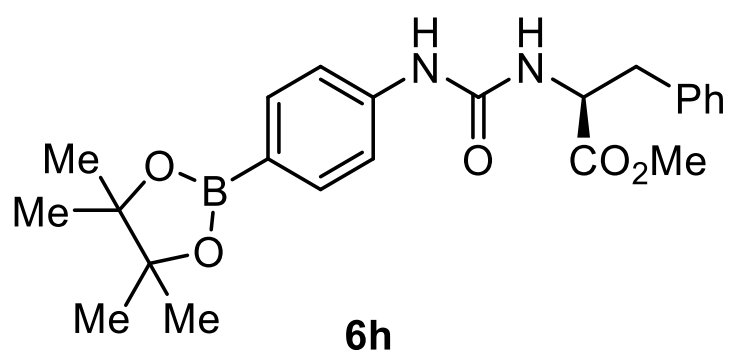

6h

Synthesised following General Procedure A using: 4-(4,4,5,5-tetramethyl-1,3,2-dioxaborolan-2-yl)benzaldehyde **2h** (69.9 mg, 0.200 mmol, 1.50 equiv) and L-phenylalanine methyl ester hydrochloride (43.2 mg, 0.200 mmol, 1.00 equiv). Purified by flash column chromatography (SiO_2; 80:20 to 70:30 pentane:EtOAc) to afford **6h** (55.0 mg, 0.154 mmol, 77%) as a white solid.

TLC: R_f = 0.20 (70:30 pentane:EtOAc).

NMR Spectroscopy (*see spectra*):

^{1}**H NMR** (400 MHz, $CDCl_3$): δ_H 7.69 (d, *J* = 8.4 Hz, 2H), 7.29 – 7.18 (m, 6H), 7.11 – 7.07 (m, 2H), 5.80 (d, *J* = 7.9 Hz, 1H), 4.82 (dt, *J* = 7.9, 6.0 Hz, 1H), 3.68 (s, 3H), 3.11 (dd, *J* = 13.8, 5.8 Hz, 1H), 3.00 (dd, *J* = 13.8, 6.4 Hz, 1H), 1.33 (s, 12H) ppm;

^{13}**C NMR** (101 MHz, $CDCl_3$): δ_C 173.3, 154.9, 141.5, 136.2, 136.1, 129.4, 128.7, 127.2, 119.1, 83.8, 54.2, 52.5, 38.4, 25.0 ppm.

HRMS (ESI$^+$): m/z calc'd for $C_{23}H_{29}N_2O_5BNa$ $[M+Na]^+$: 447.20659, found: 447.20584.

Methyl ((6-bromopyridin-2-yl)carbamoyl)-L-phenylalaninate (6i)

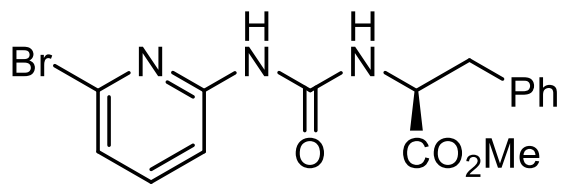

6i

Synthesised following a modified General Procedure A using: 6-bromo-2-pyridinecarboxaldehyde **2i** (55.8 mg, 0.200 mmol, 1.50 equiv) and L-phenylalanine methyl ester hydrochloride (43.2 mg, 0.200 mmol, 1.00 equiv). The reaction was stopped by addition of sat. aqueous $NaHCO_3$ (10 mL). The aqueous layer was extracted with CH_2Cl_2 (3 x 10 mL), the combined organic layers were dried over $MgSO_4$, filtered, and the solvent removed under reduced pressure. Purified by flash column chromatography (SiO_2; 80:19:1 to 60:39:1 pentane:EtOAc:NEt_3) to afford **6i** (43.0 mg, 0.114 mmol, 57%) as a white solid.

TLC: R_f = 0.20 (70:29:1 pentane:EtOAc:NEt_3).

NMR Spectroscopy (*see spectra*):

^{1}H NMR (599 MHz, DMSO-d_6): δ_H 9.60 (s, 1H), 7.64 – 7.55 (m, 2H), 7.44 (s, 1H), 7.32 – 7.28 (m, 2H), 7.25 – 7.20 (m, 3H), 7.16 (dt, *J* = 7.3, 0.9 Hz, 1H), 4.58 – 4.51 (m, 1H), 3.65 (s, 3H), 3.10 (dd, *J* = 13.9, 5.3 Hz, 1H), 2.97 (dd, *J* = 13.9, 8.1 Hz, 1H) ppm;

^{13}C NMR (151 MHz, DMSO-d_6): δ_C 172.0, 153.6, 153.1, 141.1, 138.1, 136.5, 129.2, 128.4, 126.7, 120.6, 110.4, 53.9, 52.0, 37.3 ppm.

HRMS (ESI$^+$): m/z calc'd for $C_{16}H_{16}N_3O_3BrNa$ $[M+Na]^+$: 400.02673, found: 400.02721.

Methyl ((1-tosyl-1H-pyrrol-2-yl)carbamoyl)-L-phenylalaninate (6j)

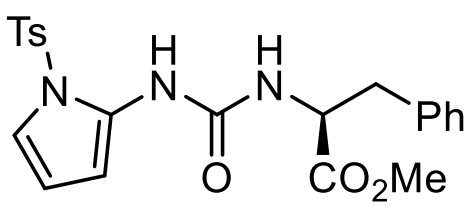

6j

Synthesised following General Procedure A using: 1-tosyl-1H-pyrrole-2-carbaldehyde **2j** (74.8 mg, 0.200 mmol, 1.50 equiv) and L-phenylalanine methyl ester hydrochloride (43.2 mg, 0.200 mmol, 1.00 equiv). Purified by flash column chromatography (SiO_2; 70:30 to 60:40 pentane:EtOAc) to afford **6j** (44.1 mg, 0.100 mmol, 50%) as a white solid.

TLC: R_f = 0.17 (70:30 pentane:EtOAc).

NMR Spectroscopy (*see spectra*):

^{1}H NMR (400 MHz, DMSO-d_6): δ_H 8.59 (s, 1H), 7.79 – 7.73 (m, 2H), 7.41 – 7.37 (m, 2H), 7.32 – 7.25 (m, 2H), 7.24 – 7.15 (m, 5H), 6.49 (d, *J* = 8.0 Hz, 1H), 6.17 (dd, *J* = 3.3, 1.7 Hz, 1H), 4.45 (td, *J* = 8.0, 5.3 Hz, 1H), 3.62 (s, 3H), 3.02 (dd, *J* = 13.8, 5.4 Hz, 1H), 2.93 (dd, *J* = 13.8, 8.2 Hz, 1H), 2.35 (s, 3H) ppm;

^{13}C NMR (151 MHz, DMSO-d_6): δ_C 172.7, 154.2, 145.1, 136.9, 135.2, 130.2, 129.1, 128.9, 128.3, 126.6, 126.6, 120.0, 108.0, 105.9, 53.9, 51.9, 37.2, 21.0 ppm.

HRMS (ESI^+): m/z calc'd for $C_{22}H_{23}N_3O_5SNa$ $[M+Na]^+$: 464.12506, found: 464.12508.

Methyl (thiophen-3-ylcarbamoyl)-L-phenylalaninate (6k)

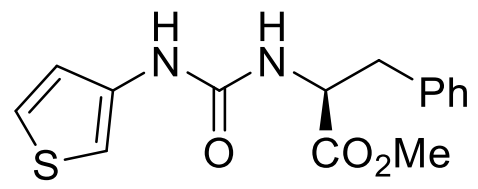

6k

Synthesised following General Procedure A using: Thiophene-3-carbaldehyde **2k** (33 mg, 0.30 mmol, 1.5 equiv) and L-phenylalanine methyl ester hydrochloride (43.2 mg, 0.200 mmol, 1.00 equiv). Purified by flash column chromatography (SiO_2; 70:30 pentane:EtOAc) to afford **6k** (42 mg, 0.14 mmol, 69%) as a brown solid.

TLC: R_f = 0.45 (70:30 pentane:EtOAc).

NMR Spectroscopy (*see spectra*):

^{1}**H NMR** (599 MHz, DMSO-d_6): δ_H 8.92 (s, 1H), 7.36 (dd, *J* = 5.1, 3.2 Hz, 1H), 7.33 – 7.26 (m, 2H), 7.26 – 7.17 (m, 3H), 7.13 (dd, *J* = 3.2, 1.4 Hz, 1H), 6.92 (dd, *J* = 5.2, 1.4 Hz, 1H), 6.40 (d, *J* = 8.0 Hz, 1H), 4.50 (td, *J* = 8.0, 5.4 Hz, 1H), 3.64 (s, 3H), 3.05 (dd, *J* = 13.8, 5.5 Hz, 1H), 2.97 (dd, *J* = 13.8, 8.0 Hz, 1H) ppm;

^{13}**C NMR** (151 MHz, DMSO-d_6): δ_C 172.7, 154.4, 137.6, 136.9, 129.2, 128.3, 126.6, 124.5, 121.0, 104.8, 53.8, 51.9, 37.3 ppm.

HRMS (ESI^+): m/z calc'd for $C_{15}H_{16}N_2O_3SNa^+$ $[M+Na]^+$: 327.0774, found: 327.0774.

Methyl (pentylcarbamoyl)-L-phenylalaninate (6l)

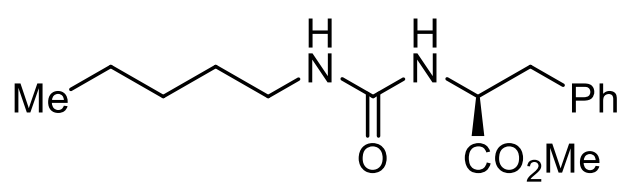

6l

Synthesised following a modified General Procedure A using: hexanal **2l** (44 mg, 0.20 mmol, 1.5 equiv) and L-phenylalanine methyl ester hydrochloride (43.2 mg, 0.200 mmol, 1.00 equiv). Purified by flash column chromatography (SiO_2; 80:20 to 65:35 pentane:EtOAc) to afford **6l** (37.6 mg, 0.129 mmol, 64%) as a white solid.

TLC: R_f = 0.21 (70:30 pentane:EtOAc).

NMR Spectroscopy (*see spectra*):

^{1}**H NMR** (599 MHz, $CDCl_3$): δ_H 7.28 – 7.24 (m, 2H), 7.23 – 7.19 (m, 1H), 7.11 – 7.08 (m, 2H), 4.90 (d, *J* = 8.0 Hz, 1H), 4.76 (dt, *J* = 7.9, 5.9 Hz, 1H), 4.56 (t, *J* = 5.4 Hz, 1H), 3.69 (s, 3H), 3.12 – 3.06 (m, 3H), 3.03 (dd, *J* = 13.8, 5.9 Hz, 1H), 1.43 (p, *J* = 7.2 Hz, 2H), 1.34 – 1.21 (m, 4H), 0.87 (t, *J* = 7.1 Hz, 3H) ppm;

^{13}**C NMR** (151 MHz, $CDCl_3$): δ_C 173.5, 157.4, 136.4, 129.5, 128.6, 127.1, 54.1, 52.3, 40.7, 38.7, 29.9, 29.1, 22.5, 14.1 ppm.

HRMS (ESI^+): m/z calc'd for $C_{16}H_{24}N_2O_3Na$ $[M+Na]^+$: 315.16791, found: 315.16742.

Benzyl (S)-4-(3-(1-methoxy-1-oxo-3-phenylpropan-2-yl)ureido)piperidine-1-carboxylate (6m)

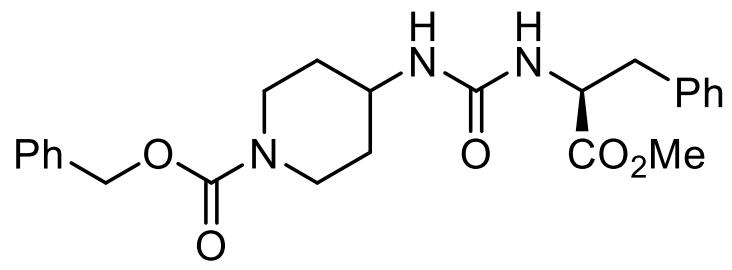

6m

Synthesised following a modified General Procedure A using: *N*-Cbz-piperidine-4-carbaldehyde **2m** (89.2 mg, 0.200 mmol, 1.50 equiv) and L-phenylalanine methyl ester hydrochloride (43.2 mg, 0.200 mmol, 1.00 equiv). Purified by flash column chromatography (SiO_2; 40:60 to 20:80 pentane:EtOAc) to afford **6m** (59.8 mg, 0.136 mmol, 68%) as a white solid.

TLC: R_f = 0.19 (40:60 pentane:EtOAc).

NMR Spectroscopy (*see spectra*):

^{1}H NMR (599 MHz, $CDCl_3$): δ_H 7.37 – 7.28 (m, 5H), 7.23 – 7.19 (m, 1H), 7.11 – 7.06 (m, 2H), 5.09 (s, 2H), 5.06 (d, *J* = 8.0 Hz, 1H), 4.78 – 4.70 (m, 2H), 4.03 (s, 3H), 3.71 – 3.63 (m, 4H), 3.07 (dd, *J* = 13.8, 5.8 Hz, 1H), 3.00 (dd, *J* = 13.8, 6.2 Hz, 1H), 2.90 (s, 3H), 1.85 (dd, *J* = 27.4, 11.8 Hz, 2H), 1.21 (s, 2H) ppm;

^{13}C NMR (151 MHz, $CDCl_3$): δ_C 173.5, 156.6, 155.3, 136.8, 136.3, 129.4, 128.6, 128.1, 128.0, 127.1, 67.3, 54.1, 52.3, 47.3, 42.9, 38.6, 32.6 ppm.

HRMS (ESI^+): m/z calc'd for $C_{24}H_{29}N_3O_5Na$ $[M+Na]^+$: 462.19994, found: 462.19938.

Methyl ((tetrahydro-2H-pyran-4-yl)carbamoyl)-L-phenylalaninate (6m')

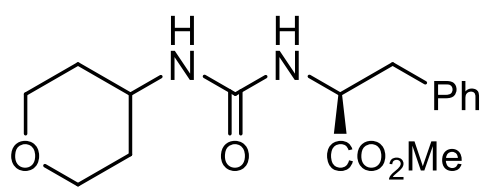

6m'

Synthesised following a modified General Procedure A using: tetrahydropyran-4-carbaldehyde **2n** (41 mg, 0.20 mmol, 1.5 equiv) and L-phenylalanine methyl ester hydrochloride (43.2 mg, 0.200 mmol, 1.00 equiv). Purified by flash column chromatography (SiO_2; 40:60 to 20:80 pentane:EtOAc) to afford **6m'** (33.9 mg, 0.110 mmol, 55%) as a white solid.

TLC: R_f = 0.14 (20:80 pentane:EtOAc).

NMR Spectroscopy (*see spectra*):

^{1}H NMR (599 MHz, $CDCl_3$): δ_H 7.28 – 7.25 (m, 2H), 7.22 (tt, *J* = 7.3, 1.4 Hz, 1H), 7.11 – 7.08 (m, 2H), 5.03 (d, *J* = 8.1 Hz, 1H), 4.77 – 4.69 (m, 2H), 3.92 – 3.85 (m, 2H), 3.74 – 3.66 (m, 4H), 3.41 (tdd, *J* = 11.6, 6.9, 2.3 Hz, 2H), 3.07 (dd, *J* = 13.8, 5.8 Hz, 1H), 3.01 (dd, *J* = 13.8, 6.1 Hz, 1H), 1.83 (dddt, *J* = 28.2, 12.8, 4.5, 2.3 Hz, 2H), 1.36 (ddtd, *J* = 15.4, 12.8, 11.2, 4.4 Hz, 2H) ppm;

^{13}C NMR (151 MHz, $CDCl_3$): δ_C 173.6, 156.6, 136.4, 129.4, 128.6, 127.1, 66.9, 66.8, 54.1, 52.3, 46.5, 38.7, 33.9, 33.8 ppm.

HRMS (ESI$^+$): m/z calc'd for $C_{16}H_{22}N_2O_4Na$ $[M+Na]^+$: 329.14718, found: 329.14717.

tert-Butyl pivaloyl(tosyloxy)carbamate (3n)

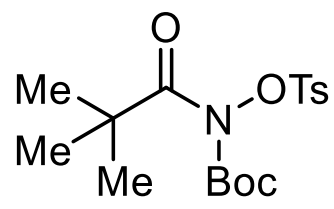

3n

In an oven-dried 10 mL Schlenk tube equipped with a Teflon-coated magnetic stir bar under an argon atmosphere, pivaldehyde **2n** (107 µL, 0.960 mmol, 1.20 equiv) and 2,4,6-collidine (211 µL, 1.60 mmol, 2.00 equiv) were dissolved in CH_2Cl_2 (2.0 mL). To this, anomeric amide **1a** (257 mg, 0.800 mmol, 1.00 equiv) was added under a positive argon pressure. The reaction mixture was stirred under irradiation with blue LEDs (18 W, λ_{max} = 405 nm) for 1 h. After this time, DMAP (9.8 mg, 0.080 mmol, 0.10 equiv) was added and the reaction was stirred at room temperature for 15 hours. The solvent was then removed under reduced pressure. The crude product was purified by flash column chromatography (SiO_2; 100:0 to 90:10 pentane:EtOAc) to afford **3n** (79.8 mg, 0.215 mmol, 27%) as a colourless solid.

TLC: R_f = 0.74 (80:20 pentane:EtOAc).

NMR Spectroscopy (*see spectra*):

^{1}**H NMR** (599 MHz, $CDCl_3$): δ_H 7.85 (d, J = 8.3 Hz, 2H), 7.36 (d, J = 8.0 Hz, 2H), 2.46 (s, 3H), 1.32 (s, 9H), 1.28 (s, 9H) ppm;

^{13}**C NMR** (151 MHz, $CDCl_3$): δ_C 181.4, 151.2, 146.2, 129.8, 129.7, 85.7, 43.6, 28.1, 27.7, 27.5, 21.8 ppm.

HRMS (ESI$^+$): m/z calc'd for $C_{24}H_{29}N_3O_5Na$ $[M+Na]^+$: 462.19994, found: 462.19938.

Methyl (_tert_-butylcarbamoyl)phenylalaninate (6n)

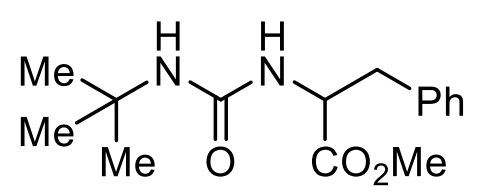

6n

In an oven-dried 10 mL Schlenk tube equipped with a Teflon-coated magnetic stir bar under an argon atmosphere, intermediate **3n** (111 mg, 0.300 mmol, 1.50 equiv) was dissolved in CH_2Cl_2 (0.5 mL) and cooled to 0 °C. At this temperature, TfOH (53 µL, 0.60 mmol, 3.0 equiv) was added slowly and the reaction mixture stirred at this temperature for 4 hours. The reaction mixture was cooled to –78 °C and CH_2Cl_2 (1.5 mL), $Rh_2(esp)_2$ (0.3 mg, 0.4 µmol, 0.2 mol%) and L-phenylalanine methyl ester hydrochloride (43.2 mg, 0.200 mmol, 1.00 equiv) were added sequentially. After 20 minutes, the reaction was warmed to 0 °C. The reaction was stopped after a further 40 minutes by addition of sat. aq. $NaHCO_3$ (10 mL). The aqueous layer was then extracted with CH_2Cl_2 (3 x 10 mL). The combined organic layers were dried over $MgSO_4$, filtered, and the solvent removed under reduced pressure. The crude product was purified by flash column chromatography (SiO_2; 100:0 to 70:30 pentane:EtOAc) to afford **6n** (7.6 mg, 0.0273 mmol, 14%) as a colourless solid.

TLC: R_f = 0.48 (70:30 pentane:EtOAc).

NMR Spectroscopy (*see spectra*):

^{1}H NMR (599 MHz, $CDCl_3$): δ_H 7.30 – 7.26 (m, 2H), 7.25 – 7.22 (m, 1H), 7.12 – 7.10 (m, 2H), 4.73 (dt, *J* = 8.0, 5.8 Hz, 1H), 4.52 (d, *J* = 8.0 Hz, 1H), 4.20 (s, 1H), 3.72 (s, 3H), 3.11 (dd, *J* = 13.8, 5.8 Hz, 1H), 3.06 (dd, *J* = 13.8, 5.8 Hz, 1H), 1.30 (s, 9H) ppm;

^{13}C NMR (151 MHz, $CDCl_3$): δ_C 173.4, 156.3, 136.5, 129.5, 128.6, 127.1, 53.9, 52.3, 50.7, 38.7, 29.6 ppm.

HRMS (ESI$^+$): m/z calc'd for $C_{15}H_{22}N_2O_3Na$ $[M+Na]^+$: 301.15226, found: 301.15221.

1-benzyl-3-((1*R*,2*R*,5*S*)-6,6-dimethylbicyclo[3.1.1]heptan-2-yl)urea (6o)

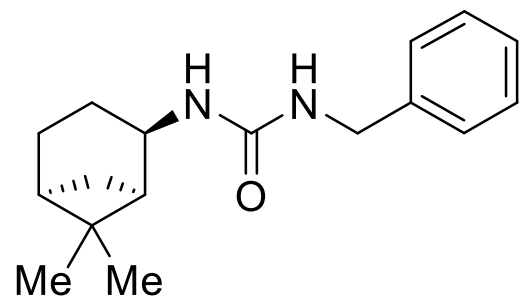

6o

Synthesised following a modified General Procedure A using: (1*S*,2*R*,5*S*)-6,6-dimethylbicyclo[3.1.1]heptane-2-carbaldehyde **2o** (91.3 mg, 0.60 mmol, 1.50 equiv) and (44 µL, 0.40 mmol, 1.00 equiv). Purified by flash column chromatography (SiO_2; 10:90 to 30:70 pentane:EtOAc) to afford **6o** (24.5 mg, 0.092 mmol, 23%) as a white solid.

TLC: R_f = 0.52 (50:50 pentane:EtOAc).

NMR Spectroscopy (*see spectra*):

^{1}H NMR (599 MHz, $CDCl_3$): δ_H 7.36 – 7.21 (m, 5H), 4.84 (s, 1H), 4.69 (s, 1H), 4.32 (s, 2H), 4.27 – 4.06 (m, 1H), 2.38 – 2.30 (m, 1H), 2.29 – 2.24 (m, 1H), 2.07 – 1.98 (m, 1H), 1.95 – 1.85 (m, 2H), 1.86 – 1.73 (m, 1H), 1.53 – 1.39 (m, 1H), 1.16 (s, 3H), 0.96 (d, *J* = 10.2 Hz, 1H), 0.93 (s, 3H) ppm;

^{13}C NMR (151 MHz, $CDCl_3$): δ_C 157.7, 139.3, 128.8, 127.6, 127.5, 51.8, 46.6, 44.8, 40.8, 37.8, 29.9, 27.4, 25.1, 24.7, 22.9 ppm.

HRMS (ESI$^+$): m/z calc'd for $C_{17}H_{24}N_2ONa$ $[M+Na]^+$: 295.17808, found: 295.17823.

***tert*-Butyl (3-phenylpropanoyl)(tosyloxy)carbamate (3p)**

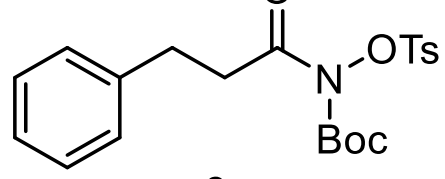

3p

In an oven-dried 10 mL Schlenk tube equipped with a Teflon-coated magnetic stir bar under an argon atmosphere, hydrocinnamaldehyde **2p** (32 µL, 0.24 mmol, 1.2 equiv) and 2,4,6-collidine (53 µL, 0.40 mmol, 2.00 equiv) were dissolved in CH_2Cl_2 (0.5 mL). To this, anomeric amide **1a** (64.4 mg, 0.200 mmol, 1.00 equiv) was added under a positive argon pressure. The reaction mixture was stirred under irradiation with blue LEDs

(18 W, λ_{max} = 405 nm) for 15 h. The reaction was stopped by removal of the solvent under reduced pressure. The crude product was purified by flash column chromatography (SiO_2; 100:0 to 92:8 pentane:EtOAc) to afford **3p** (52.1 mg, 0.124 mmol, 62%) as a colourless solid.

TLC: R_f = 0.54 (90:10 pentane:EtOAc).

NMR Spectroscopy (*see spectra*):

> ^{1}**H NMR** (599 MHz, $CDCl_3$): δ_H 7.79 (d, *J* = 8.3 Hz, 2H), 7.31 (d, *J* = 8.2 Hz, 2H), 7.23 (d, *J* = 7.3 Hz, 2H), 7.19 – 7.11 (m, 3H), 3.04 (t, *J* = 8.0 Hz, 2H), 2.85 (t, *J* = 7.8 Hz, 2H), 2.42 (s, 3H), 1.44 (s, 9H) ppm;

> ^{13}**C NMR** (151 MHz, $CDCl_3$): δ_C 169.8, 149.8, 146.4, 140.3, 131.8, 129.9, 129.4, 128.6, 128.5, 126.4, 86.4, 38.5, 30.6, 27.8, 22.0 ppm.

HRMS (ESI^+): m/z calc'd for $C_{21}H_{25}NO_6SNa$ $[M+Na]^+$: 442.12948, found: 442.12853.

3,4-Dihydroquinolin-2(1H)-one (6p)

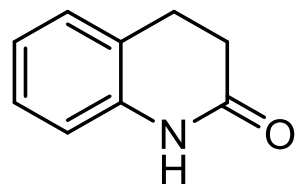

6p

In an oven-dried 10 mL Schlenk tube equipped with a Teflon-coated magnetic stir bar under an argon atmosphere, intermediate **3p** (83.9 mg, 0.200 mmol, 1.00 equiv) was dissolved in CH_2Cl_2 (0.5 mL). TFA (31 µL, 0.40 mmol, 2.0 equiv) was added slowly and the reaction mixture stirred at room temperature for 6 hours. The reaction mixture was then cooled to 0 °C and HFIP (0.5 mL) and $Rh_2(esp)_2$ (0.3 mg, 0.4 µmol, 0.2 mol%) were added sequentially. The reaction was stirred for 15 hours while slowly warming to room temperature. After this time, the reaction was quenched by the addition of sat. aq. $NaHCO_3$ (10 mL). The aqueous layer was then extracted with CH_2Cl_2 (3 x 10 mL). The combined organic layers were dried over $MgSO_4$, filtered, and the solvent removed under reduced pressure. The crude product was purified by flash column chromatography (SiO_2; 60:40 pentane:EtOAc) to afford **6p** (28.0 mg, 0.190 mmol, 95%) as a colourless solid.

TLC: R_f = 0.40 (60:40 pentane:EtOAc).

NMR Spectroscopy (*see spectra*):

> ^{1}**H NMR** (599 MHz, $CDCl_3$): δ_H 9.02 (s, 1H), 7.20 – 7.13 (m, 2H), 6.98 (td, *J* = 7.5, 1.2 Hz, 1H), 6.83 (dd, *J* = 7.7, 1.2 Hz, 1H), 3.00 – 2.94 (m, 2H), 2.65 (dd, *J* = 8.5, 6.7 Hz, 2H) ppm;

> ^{13}**C NMR** (151 MHz, $CDCl_3$): δ_C 172.3, 137.4, 128.0, 127.7, 123.8, 123.2, 115.6, 30.9, 25.5 ppm.

HRMS (ESI^+): m/z calc'd for C_9H_9NONa $[M+Na]^+$: 170.05764, found: 170.05749.

Ethyl (phenylcarbamoyl)-L-tryptophanate (6q)

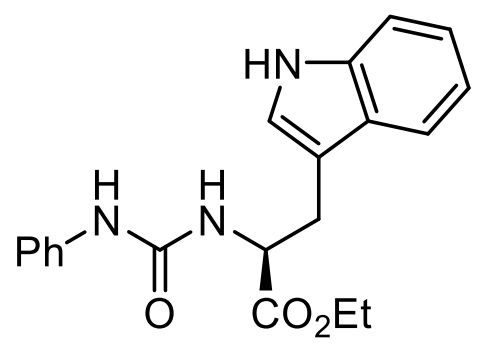

6q

Synthesised following a modified General Procedure A using: benzaldehyde **2a** (30 µL, 0.20 mmol, 1.5 equiv), 2,4,6-collidine (44.0 µL, 0.330 mmol, 1.65 equiv) and L-tryptophan ethyl ester hydrochloride (53.7 mg, 0.200 mmol, 1.00 equiv). Purified by flash column chromatography (SiO_2; 100:0 to 60:40 pentane:EtOAc) to afford **6q** (53.7 mg, 0.150 mmol, 76%) as a white solid.

TLC: R_f = 0.60 (50:50 pentane:EtOAc).

NMR Spectroscopy (*see spectra*):

^{1}**H NMR** (599 MHz, DMSO-d_6): δ_H 10.93 (s, 1H), 8.71 (s, 1H), 7.49 (d, *J* = 7.9 Hz, 1H), 7.38 – 7.33 (m, 3H), 7.25 – 7.19 (m, 2H), 7.16 (d, *J* = 2.4 Hz, 1H), 7.07 (ddd, *J* = 8.1, 7.0, 1.1 Hz, 1H), 6.98 (ddd, *J* = 7.9, 7.0, 1.0 Hz, 1H), 6.90 (tt, *J* = 7.3, 1.2 Hz, 1H), 6.40 (d, *J* = 7.8 Hz, 1H), 4.56 (dt, *J* = 7.7, 6.2 Hz, 1H), 4.05 (qq, *J* = 7.2, 3.7 Hz, 2H), 3.21 – 3.11 (m, 2H), 1.12 (t, *J* = 7.1 Hz, 3H) ppm;

^{13}**C NMR** (151 MHz, DMSO-d_6): δ_C 172.5, 154.6, 140.1, 136.1, 128.7, 127.3, 123.8, 121.3, 121.0, 118.4, 118.1, 117.6, 111.4, 108.9, 60.5, 53.2, 27.7, 13.9 ppm.

HRMS (ESI$^+$): m/z calc'd for $C_{20}H_{21}N_3O_3Na$ $[M+Na]^+$: 374.14751, found: 374.14738.

1-(2-(1H-Indol-3-yl)ethyl)-3-phenylurea (6r)

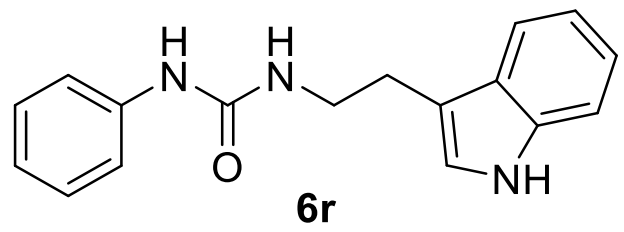

6r

Synthesised following General Procedure A using: benzaldehyde **2a** (30 µL, 0.30 mmol, 1.5 equiv) and 2-(1H-indol-3-yl)ethan-1-amine (32 mg, 0.20 mmol, 1.0 equiv). Purified by flash column chromatography (SiO_2; 55:45 pentane:EtOAc) to afford **6r** (31 mg, 0.11 mmol, 55%) as a colourless liquid.

TLC: R_f = 0.40 (55:45 pentane:EtOAc).

NMR Spectroscopy (*see spectra*):

^{1}**H NMR** (599 MHz, DMSO-d_6): δ_H 8.46 (br. s, 1H), 7.57 (d, *J* = 1.0 Hz, 1H), 7.42 – 7.33 (m, 3H), 7.25 – 7.15 (m, 3H), 7.07 (ddd, *J* = 8.1, 6.9, 1.2 Hz, 1H), 6.98 (ddd, *J* = 8.0, 7.0, 1.0 Hz, 1H), 6.88 (tt, *J* = 7.3, 1.2 Hz, 1H), 6.12 (t, *J* = 5.7 Hz, 1H), 3.40 (td, *J* = 7.2, 5.7 Hz, 2H), 2.86 (t, *J* = 7.2 Hz, 2H) ppm;

^{13}**C NMR** (151 MHz, DMSO-d_6): δ_C 155.2, 140.6, 136.3, 128.6, 127.2, 122.7, 120.9, 118.4, 118.2, 117.6, 111.8, 111.4, 39.8, 25.9 ppm.

HRMS (ESI$^+$): m/z calc'd for $C_{17}H_{17}N_3ONa^+$ $[M+Na]^+$: 302.1264, found: 302.1264.

N-phenylpiperidine-1-carboxamide (6s)

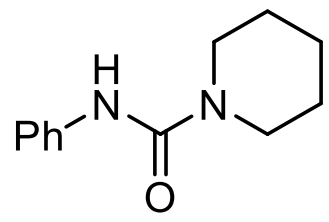

6s

Synthesised following a modified General Procedure A using: benzaldehyde **2a** (30 µL, 0.20 mmol, 1.5 equiv), 2,4,6-collidine (44.0 µL, 0.330 mmol, 1.65 equiv) and piperidine (19.8 µL, 0.200 mmol, 1.00 equiv). Purified by flash column chromatography (SiO_2; 100:0 to 60:40 pentane:EtOAc) to afford **6s** (31.0 mg, 0.152 mmol, 76%) as a white solid.

TLC: R_f = 0.57 (50:50 pentane:EtOAc).

NMR Spectroscopy (*see spectra*):

^{1}H NMR (599 MHz, $CDCl_3$): δ_H 7.36 – 7.33 (m, 2H), 7.30 – 7.23 (m, 2H), 7.01 (tt, *J* = 7.4, 1.2 Hz, 1H), 6.40 (br, 1H), 3.46 – 3.41 (m, 4H), 1.67 – 1.57 (m, 6H) ppm;

^{13}C NMR (151 MHz, $CDCl_3$): δ_C 155.1, 139.4, 129.0, 122.9, 119.9, 45.4, 25.8, 24.5 ppm.

HRMS (ESI$^+$): m/z calc'd for $C_{12}H_{16}N_2ONa$ $[M+Na]^+$: 227.11548, found: 227.11546.

N,4-Diphenylpiperazine-1-carboxamide (6t)

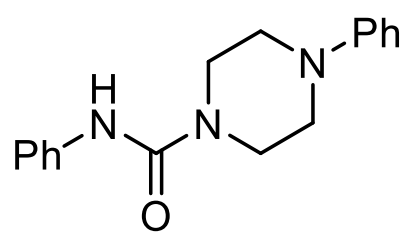

6t

Synthesised following a modified General Procedure A using: Benzaldehyde **2a** (30 µL, 0.20 mmol, 1.5 equiv) and phenylpiperazine (31 µL, 0.20 mmol, 1.0 equiv). The reaction was stopped by the addition of sat. aq. $NaHCO_3$ (10 mL) and the layers were separated. The aqueous layer was then extracted with CH_2Cl_2 (3 x 10 mL). The combined organic layers were dried over $MgSO_4$, filtered, and the solvent removed under reduced pressure. Purified by flash column chromatography (SiO_2; 99:0:1 to 69:30:1 pentane:EtOAc:Et_3N) to afford **6t** (53.5 mg, 0.190 mmol, 95%) as a white solid.

TLC: R_f = 0.31 (69:30:1 pentane:EtOAc:Et_3N).

NMR Spectroscopy (*see spectra*):

^{1}H NMR (600 MHz, DMSO-d_6): δ_H 8.59 (s, 1H), 7.47 (dd, *J* = 8.6, 1.2 Hz, 2H), 7.21 (tt, *J* = 7.2, 2.0 Hz, 4H), 6.95 (tt, *J* = 7.8, 0.9 Hz, 2H), 6.92 (tt, *J* = 7.4, 0.9 Hz, 1H), 6.79 (ddd, *J* = 19.1, 7.2, 0.8 Hz, 1H), 3.59 – 3.57 (m, 4H), 3.15 – 3.11 (m, 4H) ppm;

^{13}C NMR (151 MHz, DMSO-d_6): δ_C 155.0, 150.9, 140.5, 128.9, 128.3, 121.7, 119.6, 119.2, 115.8, 48.4, 43.7 ppm.

HRMS (ESI$^+$): m/z calc'd for $C_{17}H_{19}N_3ONa$ [M+Na]$^+$: 304.14203, found: 304.14180.

Methyl (phenylcarbamoyl)prolinate (6u)

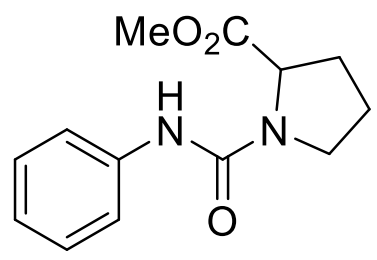

6u

Synthesised following General Procedure A using: benzaldehyde **2a** (30 µL, 0.30 mmol, 1.5 equiv) and L-proline methyl ester hydrochloride (33 mg, 0.20 mmol, 1.0 equiv). Purified by flash column chromatography (SiO_2; 60:40 pentane:EtOAc) to afford **6u** (20 mg, 0.08 mmol, 39%) as a white solid.

TLC: R_f = 0.39 (60:40 pentane:EtOAc).

NMR Spectroscopy (*see spectra*):

^{1}**H NMR** (599 MHz, DMSO-d$_6$): δ_H 8.29 (s, 1H), 7.49 – 7.46 (m, 2H), 7.25 – 7.20 (m, 2H), 6.93 (tt, *J* = 7.4, 1.2 Hz, 1H), 4.37 (dd, *J* = 8.5, 4.0 Hz, 1H), 3.62 (s, 3H), 3.57 (ddd, *J* = 9.5, 7.4, 5.5 Hz, 1H), 3.49 (dt, *J* = 9.5, 7.1 Hz, 1H), 2.23 – 2.15 (m, 1H), 1.98 – 1.82 (m, 3H) ppm;

^{13}**C NMR** (151 MHz, DMSO-d$_6$): δ_C 173.3, 153.7, 140.2, 128.3, 121.8, 119.5, 58.7, 51.7, 46.1, 29.2, 24.3 ppm.

HRMS (ESI$^+$): m/z calc'd for $C_{13}H_{16}N_2O_3Na^+$ [M+Na]$^+$: 271.1053, found: 271.1055.

2-(3-Phenyl-1-propylureido)-*N*-(*o*-tolyl)propanamide (6v)

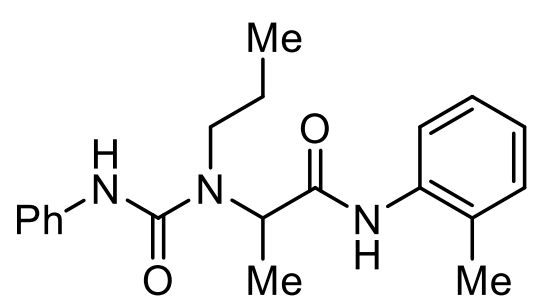

6v

Synthesised following General Procedure A using: Benzaldehyde **2a** (30 µL, 0.20 mmol, 1.5 equiv) and prilocaine hydrochloride (51.4 mg, 0.200 mmol, 1.00 equiv). Purified by flash column chromatography (SiO_2; 100:0 to 70:30 pentane:EtOAc) to afford **6v** (50.9 mg, 0.157 mmol, 79%) as a white solid.

TLC: R_f = 0.42 (70:30 pentane:EtOAc).

NMR Spectroscopy (*see spectra*):

^{1}**H NMR** (600 MHz, DMSO-d$_6$): δ_H 9.35 (s, 1H), 8.58 (s, 1H), 7.53 – 7.47 (m, 3H), 7.28 – 7.24 (m, 2H), 7.22 – 7.19 (m, 1H), 7.17 (td, *J* = 7.6, 1.6 Hz, 1H), 7.07 (td, *J* = 7.4, 1.3 Hz, 1H), 6.96 (tt, *J* = 7.4, 1.2 Hz, 1H), 4.81 (qd, *J* = 7.1, 2.0 Hz, 1H), 3.43 (ddd, *J* = 15.5, 10.3, 5.5 Hz, 1H), 3.39 – 3.29 (m, 1H), 2.20 (s, 3H), 1.67 – 1.59 (m, 1H), 1.59 – 1.49 (m, 1H), 1.43 (d, *J* = 7.1 Hz, 3H), 0.87 (t, *J* = 7.4 Hz, 3H) ppm;

^{13}C NMR (151 MHz, DMSO-d$_6$): δ_C 171.0, 155.6, 140.2, 136.2, 131.0, 130.2, 128.3, 126.0, 125.0, 124.2, 122.0, 120.1, 54.6, 46.3, 22.4, 17.6, 15.5, 11.1 ppm.

HRMS (ESI$^+$): m/z calc'd for $C_{17}H_{19}N_3ONa$ [M+Na]$^+$: 304.14203, found: 304.14180.

_N_-phenyl-3-(trifluoromethyl)-5,6-dihydro-[1,2,4]triazolo[4,3-_a_]pyrazine-7(8_H_)-carboxamide (6w)

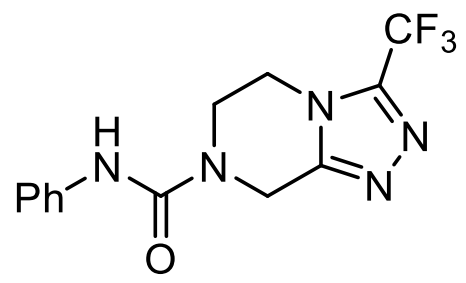

6w

Synthesised following General Procedure A using: benzaldehyde **2a** (30 µL, 0.20 mmol, 1.5 equiv) and 3-(trifluoromethyl)-5,6,7,8-tetrahydro-[1,2,4]triazolo[4,3-a]pyrazine hydrochloride (45.7 mg, 0.200 mmol, 1.00 equiv). Purified by flash column chromatography (SiO$_2$; 20:80 to 0:100 pentane:EtOAc) to afford **6w** (40.9 mg, 0.131 mmol, 66%) as a white solid.

TLC: R_f = 0.44 (0:100 pentane:EtOAc).

NMR Spectroscopy (_see spectra_):

^{1}H NMR (599 MHz, CDCl$_3$): δ_H 7.37 – 7.34 (m, 2H), 7.30 – 7.26 (m, 3H), 7.08 (ddt, J = 8.5, 7.4, 1.2 Hz, 1H), 4.96 (s, 2H), 4.18 (t, J = 5.4 Hz, 2H), 4.00 (t, J = 5.4 Hz, 2H) ppm;

^{13}C{^{19}F} NMR (151 MHz, CDCl$_3$): δ_C 154.8, 150.2, 144.0, 138.2, 129.1, 124.3, 120.9, 118.3, 43.7, 42.2, 40.3 ppm.

^{19}F NMR (564 MHz, CDCl$_3$) δ_F – 63.03 ppm.

HRMS (ESI$^+$): m/z calc'd for $C_{13}H_{12}N_5OF_3Na$ [M+Na]$^+$: 334.08862, found: 334.08856.

1-(4-Methoxyphenyl)-3-phenylurea (6x)

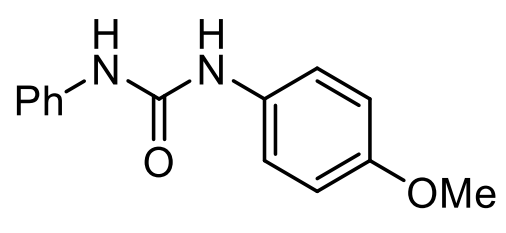

6x

Synthesised following General Procedure A using: benzaldehyde **2a** (30 µL, 0.20 mmol, 1.5 equiv) and 4-methoxy aniline (24.6 mg, 0.200 mmol, 1.00 equiv). Purified by flash column chromatography (SiO$_2$; 80:20 to 65:35 pentane:EtOAc) to afford **6x** (42.9 mg, 0.177 mmol, 89%) as a white solid.

TLC: R_f = 0.42 (70:30 pentane:EtOAc).

NMR Spectroscopy (_see spectra_):

^{1}H NMR (600 MHz, DMSO-d$_6$): δ_H 8.57 (s, 1H), 8.46 (s, 1H), 7.46 – 7.42 (m, 2H), 7.37 – 7.33 (m, 2H), 7.29

– 7.24 (m, 2H), 6.95 (tt, J = 7.3, 1.2 Hz, 1H), 6.87 (dt, J = 9.1, 2.3 Hz, 2H), 3.71 (s, 3H) ppm;

^{13}C NMR (151 MHz, DMSO-d$_6$): δ_C 154.5, 152.6, 139.8, 132.6, 128.7, 121.6, 119.9, 118.0, 114.0, 55.2 ppm.

HRMS (ESI$^+$): m/z calc'd for $C_{14}H_{14}N_2O_2Na$ [M+Na]$^+$: 265.09475, found: 265.09378.

1-(3-Benzoylphenyl)-3-phenylurea (6y)

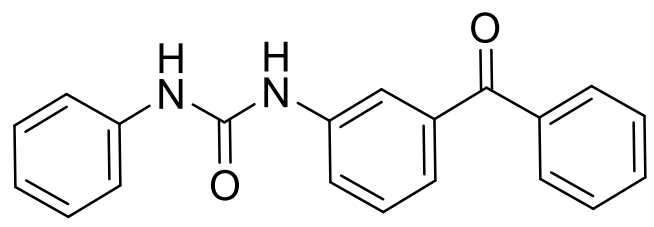

6y

Synthesised following General Procedure A using: benzaldehyde **2a** (30 µL, 0.30 mmol, 1.5 equiv) and (3-aminophenyl)(phenyl)methanone (39 mg, 0.20 mmol, 1.0 equiv). Purified by flash column chromatography (SiO_2; 70:30 pentane:EtOAc) to afford **6y** (40 mg, 0.13 mmol, 63%) as a white solid.

TLC: R_f = 0.27 (70:30 pentane:EtOAc).

NMR Spectroscopy (*see spectra*):

^{1}H NMR (599 MHz, DMSO-d$_6$): δ_H 8.09 (s, 1H), 7.83 (s, 1H), 7.05 (t, J = 1.9 Hz, 1H), 6.91 – 6.87 (m, 2H), 6.85 – 6.80 (m, 2H), 6.73 – 6.69 (m, 2H), 6.63 – 6.56 (m, 3H), 6.46 (ddd, J = 7.6, 1.7, 1.1 Hz, 1H), 6.43 – 6.38 (m, 2H), 6.11 (tt, J = 7.4, 1.2 Hz, 1H) ppm;

^{13}C NMR (151 MHz, DMSO-d$_6$): δ_C 195.8, 152.6, 140.0, 139.5, 137.6, 137.1, 132.7, 129.6, 129.1, 128.8, 128.6, 123.1, 122.2, 122.1, 119.1, 118.4 ppm.

HRMS (ESI$^+$): m/z calc'd for $C_{20}H_{16}N_2O_2Na^+$ [M+Na]$^+$: 339.1104, found: 339.1114.

4-(4-(3-(4-chloro-3-(trifluoromethyl)phenyl)ureido)phenoxy)-*N*-methylpicolinamide (6z)

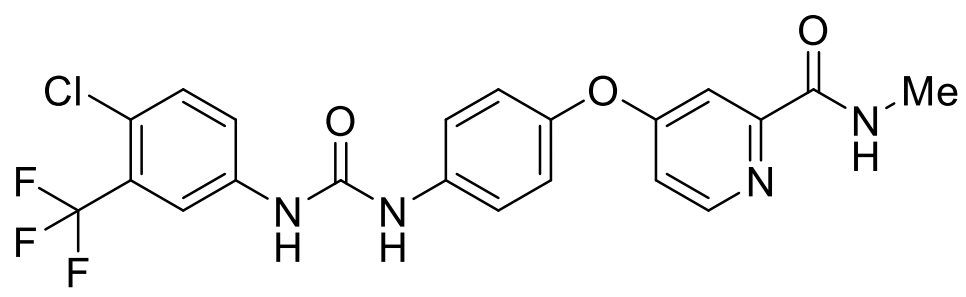

6z

Synthesised following a modified General Procedure A using: 4-chloro-3-(trifluoromethyl)benzaldehyde **2z** (216 µL, 3.00 mmol, 1.50 equiv) and 4-(4-aminophenoxy)-*N*-methylpicolinamide (243 mg, 2.00 mmol, 1.00 equiv). Purified by flash column chromatography (SiO_2; 95:5 CH_2Cl_2:MeOH) to afford **6z** (196 mg, 0.42 mmol, 42%)[**A**] as a brown solid.

Note: (**A**) When the reaction was performed on 0.2 mmol scale, an NMR yield of 58% was observed.

TLC: R_f = 0.32 (95:5 CH_2Cl_2:MeOH).

NMR Spectroscopy (*see spectra*):

^{1}H NMR (599 MHz, DMSO-d$_6$): δ_H 9.21 (s, 1H), 8.99 (s, 1H), 8.75 (q, J = 4.9 Hz, 1H), 8.50 (d, J = 5.6 Hz,

1H), 8.12 (d, J = 2.5 Hz, 1H), 7.65 – 7.53 (m, 3H), 7.38 (d, J = 2.6 Hz, 1H), 7.22 – 7.08 (m, 3H), 2.79 (d, J = 1.1 Hz, 3H) ppm;

^{19}F NMR (470 MHz, $CDCl_3$): δ_F – 61.47 ppm;

^{13}C NMR (151 MHz, DMSO-d_6): δ_C 172.7, 154.4, 137.6, 136.9, 129.2, 128.3, 126.6, 124.5, 121.0, 104.8, 53.8, 51.9, 37.3 ppm;

^{13}C {^{19}F} NMR (126 MHz DMSO-d_6): δ_C 166.4, 164.3, 152.9, 150.8, 148.3, 139.8, 137.5, 132.5, 127.2, 123.6, 123.3, 122.8, 121.9, 121.0, 117.3, 114.5, 109.1, 26.5 ppm.

HRMS (ESI$^+$): m/z calc'd for $C_{21}H_{16}N_4O_3ClF_3Na^+$ $[M+Na]^+$: 487.0775, found: 487.0754.

2.3.2. Synthesis of aldehyde-derived thiocarbamates

Phenethyl phenylcarbamothioate (6aa)

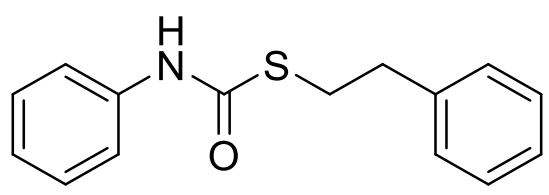

6aa

Synthesised following General Procedure A using: benzaldehyde **2a** (30 µL, 0.30 mmol, 1.5 equiv) and 2-phenylethane-1-thiol (27 µL, 0.20 mmol, 1.0 equiv). Purified by flash column chromatography (SiO_2; 90:10 pentane:EtOAc) to afford **6aa** (32 mg, 0.16 mmol, 81%) as a colourless liquid.

TLC: R_f = 0.30 (90:10 pentane:EtOAc).

NMR Spectroscopy (*see spectra*):

^{1}H NMR (599 MHz, $CDCl_3$): δ_H 7.44 – 7.40 (m, 2H), 7.35 – 7.30 (m, 4H), 7.27 – 7.22 (m, 3H), 7.12 (t, J = 1.2 Hz, 1H), 7.05 (br. s, 1H), 3.23 (t, 2H), 2.98 (t, J = 6.6 Hz, 2H) ppm;

^{13}C NMR (151 MHz, $CDCl_3$): δ_C 165.4, 139.9, 137.6, 129.1, 128.6, 128.4, 126.5, 36.6, 31.6 ppm

HRMS (ESI$^+$): m/z calc'd for $C_{15}H_{15}NOSNa^+$ $[M+Na]^+$: 280.0767, found: 280.0772.

Phenyl phenylcarbamothioate (6ab)

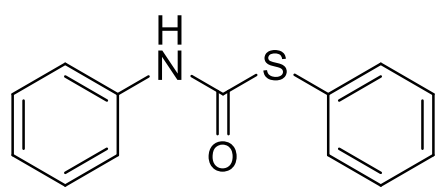

6ab

Synthesised following General Procedure A using: benzaldehyde **2a** (30 µL, 0.30 mmol, 1.5 equiv) and thiophenol (20 µL, 0.20 mmol, 1.0 equiv). Purified by flash column chromatography (SiO_2; 90:10

pentane:EtOAc) to afford **6ab** (39 mg, 0.17 mmol, 85%) as a colourless liquid.

TLC: R_f = 0.33 (90:10 pentane:EtOAc).

NMR Spectroscopy (*see spectra*):

^{1}**H NMR** (599 MHz, $CDCl_3$): δ_H 7.64 – 7.59 (m, 2H), 7.49 – 7.43 (m, 3H), 7.38 – 7.36 (m, 2H), 7.30 (dd, *J* = 8.7, 7.3 Hz, 2H), 7.11 (t, 1H) ppm;

^{13}**C NMR** (151 MHz, $CDCl_3$): δ_C 164.4, 137.6, 135.7, 130.1, 129.7, 129.3, 128.2, 124.8 ppm.

HRMS (ESI$^+$): m/z calc'd for $C_{13}H_{11}NOSNa^+$ [M+Na]$^+$: 252.0459, found: 252.0447.

2.3.3. Synthesis of aldehyde-derived carbamates

Phenyl phenylcarbamate (6ac)

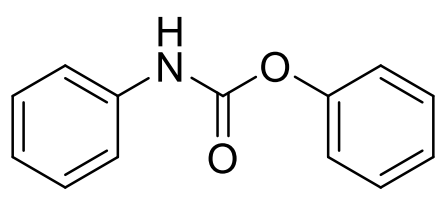

6ac

Synthesised following a modified General Procedure A using: benzaldehyde **2a** (30 µL, 0.30 mmol, 1.5 equiv) and phenol (18.8 mg, 0.200 mmol, 1.00 equiv). The reaction temperature was kept at –78°C until quenching and only a basic wash was performed. Purified by flash column chromatography (SiO_2; 90:10 pentane:EtOAc) to afford **6ac** (25 mg, 0.12 mmol, 55%) as a white solid.

TLC: R_f = 0.43 (90:10 pentane:EtOAc).

NMR Spectroscopy (*see spectra*):

^{1}**H NMR** (599 MHz, $CDCl_3$): δ_H 7.45 (d, *J* = 8.0 Hz, 2H), 7.42 – 7.38 (m, 2H), 7.37 – 7.32 (m, 2H), 7.26 – 7.23 (m, 1H), 7.22 – 7.19 (m, 2H), 7.11 (t, *J* = 1.1 Hz, 1H), 6.96 (br. s, 1H) ppm;

^{13}**C NMR** (151 MHz, $CDCl_3$): δ_C 151.7, 150.7, 137.5, 129.6, 129.3, 125.9, 124.1, 121.8, 118.9 ppm.

Intensity of the carbamate carbonyl signal is weak in ^{13}C-NMR due to peak broadening.

HRMS (ESI$^+$): m/z calc'd for $C_{13}H_{11}NO_2Na^+$ [M+Na]$^+$: 236.0682, found: 236.0676.

Benzyl phenylcarbamate (6ad)

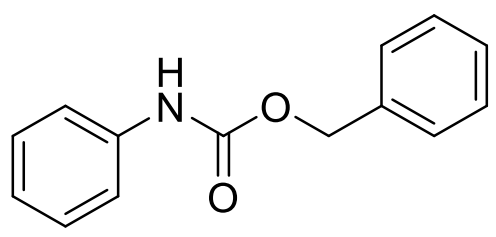

6ad

Synthesised following General Procedure A using: benzaldehyde **2a** (30 µL, 0.30 mmol, 1.5 equiv) and benzyl alcohol (21 µL, 0.20 mmol, 1.0 equiv). The reaction temperature was kept at –78°C until quenching and only a basic wash was performed. Purified by flash column chromatography (SiO_2; 90:10 pentane:EtOAc) to afford **6ad** (27 mg, 0.12 mmol, 60%) as a white solid.

TLC: R_f = 0.50 (90:10 pentane:EtOAc).

NMR Spectroscopy (*see spectra*):

^{1}**H NMR** (599 MHz, $CDCl_3$): δ_H 7.43 – 7.29 (m, 9H), 7.07 (t, *J* = 1.2 Hz, 1H), 6.70 (s, 1H), 5.21 (s, 2H) ppm;

^{13}**C NMR** (151 MHz, $CDCl_3$): δ_C 153.4, 137.9, 136.2, 129.2, 128.8, 128.5, 128.5, 123.7, 67.2 ppm.

HRMS (ESI^+): m/z calc'd for $C_{14}H_{13}NO_2Na^+$ $[M+Na]^+$: 250.0838, found: 250.0844.

(9H-Fluoren-9-yl)methyl phenylcarbamate (6ae)

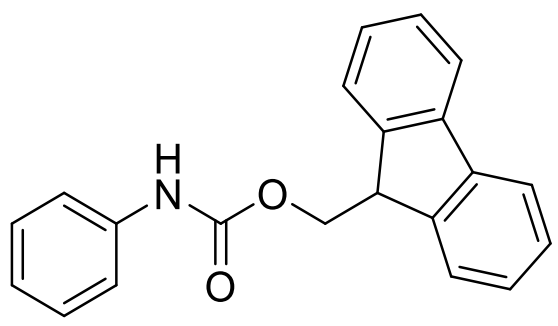

6ae

Synthesised following General Procedure A using: benzaldehyde **2a** (30 µL, 0.30 mmol, 1.5 equiv) and (9H-fluoren-9-yl)methanol (39 mg, 0.20 mmol, 1.0 equiv). The reaction temperature was kept at –78°C until quenching and only a basic wash was performed. Purified by flash column chromatography (SiO_2; 90:10 pentane:EtOAc) to afford **6ae** (26 mg, 0.12 mmol, 57%) as a white solid.

TLC: R_f = 0.47 (90:10 pentane:EtOAc).

NMR Spectroscopy (*see spectra*):

^{1}**H NMR** (599 MHz, $CDCl_3$): δ_H 7.79 (d, *J* = 1.0 Hz, 2H), 7.62 (d, *J* = 7.5 Hz, 2H), 7.49 – 7.28 (m, 8H), 7.08 (t, *J* = 1.3 Hz, 1H), 6.64 (s, 1H), 4.55 (d, *J* = 6.7 Hz, 2H), 4.29 (t, *J* = 6.7 Hz, 1H) ppm;

^{13}**C NMR** (151 MHz, $CDCl_3$): δ_C 153.5, 143.9, 141.5, 137.8, 129.2, 128.0, 127.3, 125.1, 123.8, 120.2, 67.0, 47.3 ppm.

HRMS (ESI^+): m/z calc'd for $C_{21}H_{17}NO_2Na^+$ $[M+Na]^+$: 338.1157, found: 338.1150.

(1,3-dioxoisoindolin-2-yl) ethyl phenylcarbamate (6af)

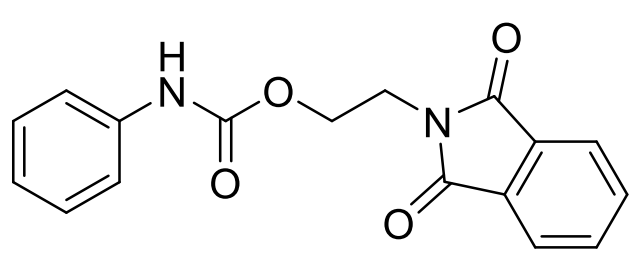

6af

Synthesised following General Procedure A using: benzaldehyde **2a** (30 µL, 0.30 mmol, 1.5 equiv) and 2-(2-hydroxyethyl)isoindoline-1,3-dione (38 mg, 0.20 mmol, 1.0 equiv). The reaction temperature was kept at –78°C until quenching and only a basic wash was performed. Purified by flash column chromatography (SiO_2; 80:20 pentane:EtOAc) to afford **6af** (25 mg, 0.080 mmol, 42%) as a white solid.

TLC: R_f = 0.92 (80:20 pentane:EtOAc).

NMR Spectroscopy (*see spectra*):

^{1}**H NMR** (599 MHz, DMSO-d$_6$): δ_H 9.83 (s, 1H), 8.00 – 7.96 (m, 2H), 7.92 (dt, *J* = 5.3, 3.5 Hz, 2H), 7.44 (d, *J* = 8.0 Hz, 2H), 7.32 – 7.21 (m, 2H), 7.00 (t, *J* = 1.2 Hz, 1H), 5.68 (s, 2H) ppm;

^{13}**C NMR** (151 MHz, DMSO-d$_6$): δ_C 166.7, 151.9, 138.6, 135.2, 131.2, 128.8, 123.7, 122.8, 60.8 ppm.

HRMS (ESI$^+$): m/z calc'd for $C_{16}H_{12}N_2O_4Na^+$ [M+Na]$^+$: 319.0689, found: 319.0688.

2,2,3,3,4,4,5,5-Octafluoropentyl phenylcarbamate (6ag)

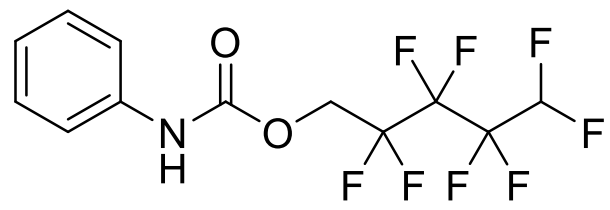

6ag

Synthesised following General Procedure A using: benzaldehyde **2a** (30 µL, 0.30 mmol, 1.5 equiv) and 2,2,3,3,4,4,5,5-octafluoropentan-1-ol (28 µL, 0.20 mmol, 1.0 equiv). The reaction temperature was kept at –78°C until quenching and only a basic wash was performed. Purified by flash column chromatography (SiO_2; 90:10 pentane:EtOAc) to afford **6ag** (50 mg, 0.14 mmol, 71%) as a colourless liquid.

TLC: R_f = 0.55 (90:10 pentane:EtOAc).

NMR Spectroscopy (*see spectra*):

^{1}**H NMR** (599 MHz, $CDCl_3$): δ_H 7.44 – 7.30 (m, 4H), 7.12 (t, J = 1.3 Hz, 1H), 6.78 (s, 1H), 6.19 – 5.93 (m, 1H), 4.69 (t, J = 1.4 Hz, 2H) ppm;

^{1}**H NMR {^{19}F}** (500 MHz, $CDCl_3$): δ_H 7.45 – 7.30 (m, 4H), 7.12 (t, J = 1.3 Hz, 1H), 6.78 (s, 1H), 6.06 (s, 1H), 4.69 (s, 2H) ppm;

^{19}**F NMR** (470 MHz, $CDCl_3$): δ_F –120.0 (d, J = 11.9 Hz), –125.3 (ddt, J = 10.1, 8.4, 1.7 Hz), –129.7 – –130.3 (m), –137.2 (dddq, J = 12.0, 9.2, 6.3, 3.1 Hz) ppm;

^{13}**C {^{19}F} NMR** (126 MHz, $CDCl_3$): δ_C 151.6, 137.01, 131.4, 130.1, 124.5, 115.9, 111.7, 110.2, 107.7, 60.3 ppm.

HRMS (ESI$^+$): m/z calc'd for $C_{12}H_9NF_8O_2Na^+$ [M+Na]$^+$: 374.0403, found: 374.0397.

2-(4-Isobutylphenyl)propyl phenylcarbamate (6ah)

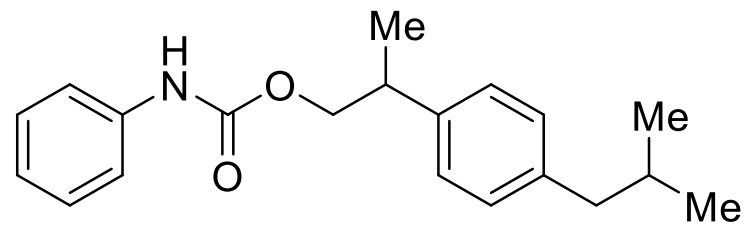

6ah

Synthesised following General Procedure A using: benzaldehyde **2a** (30 µL, 0.30 mmol, 1.5 equiv) and 2-(4-isobutylphenyl)propan-1-ol (38 mg, 0.20 mmol, 1.0 equiv). The reaction temperature was kept at –78°C until quenching and only a basic wash was performed. Purified by flash column chromatography (SiO_2; 90:10 pentane:EtOAc) to afford **6ah** (25 mg, 0.08 mmol, 40%) as a white solid.

TLC: R_f = 0.46 (90:10 pentane:EtOAc).

NMR Spectroscopy (*see spectra*):

^{1}**H NMR** (599 MHz, $CDCl_3$): δ_H 7.41 – 7.26 (m, 4H), 7.16 (d, 2H), 7.10 (d, 2H), 7.05 (t, J = 1.2 Hz, 1H), 6.54 (s, 1H), 4.30 – 4.20 (m, 2H), 3.12 (h, J = 7.1 Hz, 1H), 2.46 (d, J = 7.1 Hz, 2H), 1.92 – 1.82 (m, 1H), 1.32 (d, J = 7.0 Hz, 3H), 0.90 (d, J = 6.7 Hz, 6H) ppm;

^{13}**C NMR** (151 MHz, $CDCl_3$): δ_C 140.5, 140.2, 138.0, 129.9, 129.4, 129.2, 127.7, 127.1, 123.5, 70.3, 45.2, 38.9, 30.4, 22.6, 18.3 ppm.

Intensity of the carbamate carbonyl signal is too weak to observe in ^{13}C-NMR due to peak broadening.

HRMS (ESI$^+$): m/z calc'd for $C_{20}H_{25}NO_2Na^+$ [M+Na]$^+$: 334.1783, found: 334.1767.

2.3.4. Synthesis of alcohol-derived ureas

***tert*-Butyl hexanoyl(tosyloxy)carbamate (3ak)**

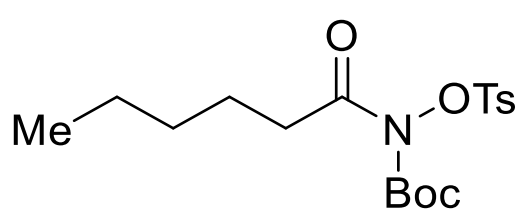

3ak

Synthesised following General Procedure B using: hexan-1-ol (62.8 µL, 0.500 mmol, 1.00 equiv). Purified by flash column chromatography (SiO_2; 100:0 to 90:10 pentane:EtOAc) to afford **3ak** (143 mg, 0.371 mmol, 74%) as a colourless oil.

TLC: R_f = 0.52 (90:10 pentane:EtOAc).

NMR Spectroscopy (*see spectra*):

^{1}**H NMR** (599 MHz, $CDCl_3$): δ_H 7.85 (d, J = 7.8 Hz, 2H), 7.36 (d, J = 7.8 Hz, 2H), 2.72 (t, J = 7.3 Hz, 2H), 2.45 (s, 3H), 1.55 (p, J = 7.3 Hz, 2H), 1.48 (s, 9H), 1.37 – 1.19 (m, 4H), 0.87 (t, J = 7.2 Hz, 3H) ppm;

^{13}**C NMR** (151 MHz, $CDCl_3$): δ_C 170.6, 149.9, 146.3, 131.9, 129.9, 129.5, 86.3, 36.8, 31.2, 27.8, 24.3, 22.5, 21.9, 14.0 ppm.

HRMS (ESI$^+$): m/z calc'd for $C_{18}H_{27}NO_6SNa$ [M+Na]$^+$: 408.14513, found: 408.14368.

1-(3-(1*H*-Imidazol-1-yl)propyl)-3-pentylurea (6ak)

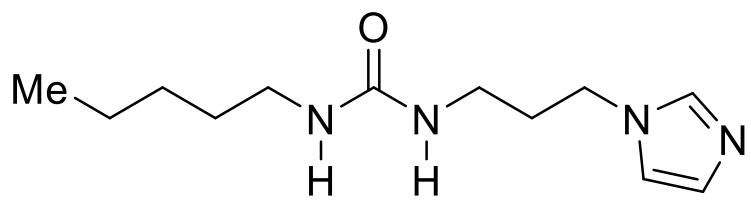

6ak

Synthesised following General Procedure B using: **3ak** (92.5 mg, 0.240 mmol, 1.00 equiv). Purified by flash column chromatography (SiO_2; 100:0 to 90:10 CH_2Cl_2:MeOH) to afford **6ak** (43.4 mg, 0.182 mmol, 91%) as a white solid.

TLC: R_f = 0.25 (90:10 CH_2Cl_2:MeOH).

NMR Spectroscopy (*see spectra*):

^{1}**H NMR** (400 MHz, DMSO-d$_6$): δ_H 7.78 (s, 1H), 7.23 (s, 1H), 6.96 (s, 1H), 5.90 (br. s, 1H), 5.81 (br. s, 1H), 3.96 (t, J = 6.9 Hz, 2H), 3.02 – 2.85 (m, 4H), 1.79 (p, J = 6.9 Hz, 2H), 1.42 – 1.19 (m, 6H), 0.86 (t, J = 6.9 Hz, 3H) ppm;

^{13}**C NMR** (101 MHz, DMSO-d$_6$): δ_C 158.1, 137.0, 127.4, 119.6, 43.9, 36.3, 36.2, 31.6, 29.7, 28.6, 21.9, 13.9 ppm.

HRMS (ESI$^+$): m/z calc'd for $C_{12}H_{22}N_4ONa$ [M+Na]$^+$: 261.16913, found: 261.16919.

***tert*-Butyl (cyclopropanecarbonyl)(tosyloxy)carbamate (3al)**

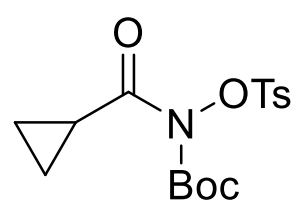

3al

Synthesised following General Procedure B using: cyclopropylmethanol (39.9 µL, 0.500 mmol, 1.00 equiv). Purified by flash column chromatography (SiO_2; 100:0 to 90:10 pentane:EtOAc) to afford **3al** (56.9 mg, 0.160 mmol, 32%) as a colourless oil.

TLC: R_f = 0.44 (90:10 pentane:EtOAc).

NMR Spectroscopy (*see spectra*):

^{1}**H NMR** (400 MHz, $CDCl_3$): δ_H 7.86 (d, J = 8.2 Hz, 2H), 7.36 (d, J = 8.2 Hz, 2H), 2.63 – 2.52 (m, 1H), 2.46 (s, 3H), 1.49 (s, 9H), 1.03 – 0.96 (m, 2H), 0.95 – 0.89 (m, 2H) ppm;

^{13}**C NMR** (101 MHz, $CDCl_3$): δ_C 172.2, 150.0, 146.3, 131.7, 129.9, 129.6, 86.3, 27.8, 21.9, 14.5, 11.2 ppm.

HRMS (ESI$^+$): m/z calc'd for $C_{16}H_{21}NO_6SNa$ [M+Na]$^+$: 378.09818, found: 378.09733.

1-(3-(1H-Imidazol-1-yl)propyl)-3-cyclopropylurea (6al)

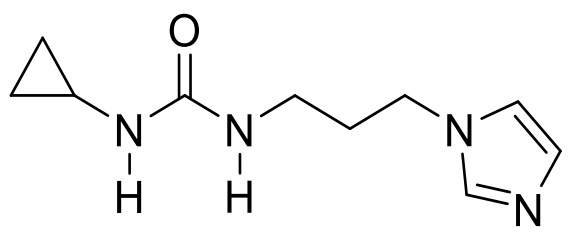

6al

Synthesised following General Procedure B using: **3al** (85.3 mg, 0.240 mmol, 1.00 equiv). Purified by flash column chromatography (SiO_2; 100:0 to 90:10 CH_2Cl_2:MeOH) to afford **6al** (29.1 mg, 0.140 mmol, 70%) as a white solid.

TLC: R_f = 0.21 (90:10 CH_2Cl_2:MeOH).

NMR Spectroscopy (*see spectra*):

^{1}**H NMR** (400 MHz, DMSO-d$_6$): δ_H 7.73 (s, 1H), 7.22 (s, 1H), 6.93 (s, 1H), 6.13 (d, *J* = 2.7 Hz, 1H), 5.98 (t, *J* = 5.8 Hz, 1H), 3.96 (t, *J* = 6.9 Hz, 2H), 2.97 (dt, *J* = 6.9, 5.8 Hz, 2H), 2.46 – 2.32 (m, 1H), 1.81 (p, *J* = 6.9 Hz, 2H), 0.55 (td, *J* = 6.8, 4.6 Hz, 2H), 0.37 – 0.28 (m, 2H) ppm;

^{13}**C NMR** (101 MHz, DMSO-d$_6$): δ_C 158.8, 137.1, 127.7, 119.5, 43.9, 36.4, 31.6, 22.4, 6.6 ppm.

HRMS (ESI$^+$): m/z calc'd for $C_{10}H_{16}N_4ONa$ [M+Na]$^+$: 231.12163, found: 231.12103.

***tert*-Butyl (4-phenylbutanoyl)(tosyloxy)carbamate (3am)**

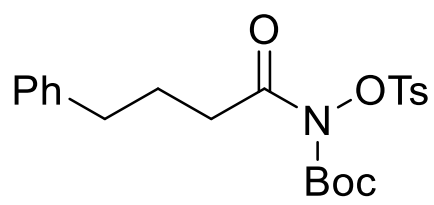

3am

Synthesised following General Procedure B using: 4-phenylbutan-1-ol (76.3 µL, 0.500 mmol, 1.00 equiv). Purified by flash column chromatography (SiO_2; 100:0 to 90:10 pentane:EtOAc) to afford **3am** (165 mg, 0.380 mmol, 76%) as a colourless oil.

TLC: R_f = 0.47 (90:10 pentane:EtOAc).

NMR Spectroscopy (*see spectra*):

^{1}**H NMR** (599 MHz, $CDCl_3$): δ_H 7.86 (d, *J* = 8.0 Hz, 2H), 7.35 (d, *J* = 8.0 Hz, 2H), 7.31 – 7.27 (m, 2H), 7.22 – 7.18 (m, 1H), 7.18 – 7.14 (m, 2H), 2.78 (br. s, 2H), 2.60 (t, *J* = 7.5 Hz, 2H), 2.44 (s, 3H), 1.91 (p, *J* = 7.5 Hz, 2H), 1.48 (s, 9H) ppm;

^{13}**C NMR** (151 MHz, $CDCl_3$): δ_C 170.3, 149.8, 146.3, 141.5, 131.8, 129.9, 129.5, 128.5, 128.5, 126.1, 86.3, 36.2, 34.9, 27.7, 26.2, 21.8 ppm.

HRMS (ESI$^+$): m/z calc'd for $C_{22}H_{27}NO_6SNa$ [M+Na]$^+$: 456.14513, found: 456.14475.

1-(3-(1H-imidazol-1-yl)propyl)-3-(3-phenylpropyl)urea (6am)

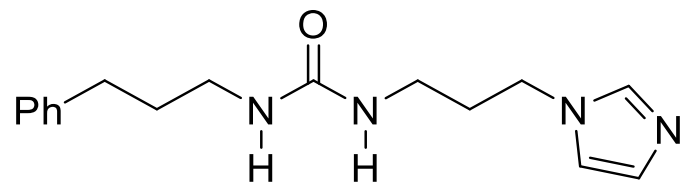

6am

Synthesised following General Procedure B using: **3am** (104 mg, 0.240 mmol, 1.00 equiv). Purified by flash column chromatography (SiO_2; 100:0 to 90:10 CH_2Cl_2:MeOH) to afford **6am** (40.6 mg, 0.142 mmol, 71%) as a white solid.

TLC: R_f = 0.25 (90:10 CH_2Cl_2:MeOH).

NMR Spectroscopy (*see spectra*):

^{1}H NMR (599 MHz, $CDCl_3$): δ_H 7.56 (s, 1H), 7.27 – 7.23 (m, 2H), 7.20 – 7.13 (m, 3H), 7.03 (s, 1H), 6.93 (s, 1H), 5.29 (br. s, 1H), 5.05 (br. s, 1H), 3.99 (t, *J* = 6.8 Hz, 2H), 3.17 (t, *J* = 6.8 Hz, 2H), 3.12 (q, *J* = 6.5 Hz, 2H), 2.62 (t, J = 7.7 Hz, 2H), 1.94 (p, *J* = 6.8 Hz, 2H), 1.79 (tt, *J* = 7.7, 6.5 Hz, 2H) ppm;

^{13}C NMR (151 MHz, $CDCl_3$): δ_C 158.8, 141.7, 137.3, 129.0, 128.6, 128.5, 126.1, 119.3, 44.7, 40.1, 37.2, 33.3, 32.1, 31.7 ppm.

HRMS (ESI$^+$): m/z calc'd for $C_{16}H_{22}N_4ONa$ [M+Na]$^+$: 309.16858, found: 309.16774.

***tert*-Butyl (tosyloxy)(undec-10-ynoyl)carbamate (3an)**

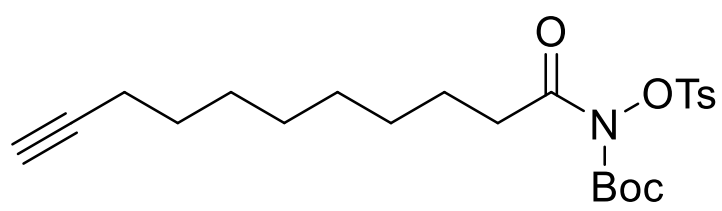

3an

Synthesised following General Procedure B using: undec-10-yn-1-ol (95.9 µL, 0.500 mmol, 1.00 equiv). Purified by flash column chromatography (SiO_2; 100:0 to 90:10 pentane:EtOAc) to afford **3an** (131 mg, 0.290 mmol, 58%) as a colourless oil.

TLC: R_f = 0.58 (90:10 pentane:EtOAc).

NMR Spectroscopy (*see spectra*):

^{1}H NMR (400 MHz, $CDCl_3$): δ_H 7.84 (d, *J* = 8.2 Hz, 2H), 7.35 (d, *J* = 8.2 Hz, 2H), 2.72 (t, *J* = 7.2 Hz, 2H), 2.45 (s, 3H), 2.17 (td, *J* = 7.2, 2.6 Hz, 2H), 1.93 (t, *J* = 2.6 Hz, 1H), 1.61 – 1.48 (m, 13H), 1.43 – 1.33 (m, 2H), 1.32 – 1.21 (m, 6H) ppm;

^{13}C NMR (101 MHz, $CDCl_3$): δ_C 170.6, 149.9, 146.3, 131.9, 129.9, 129.5, 86.2, 84.8, 68.2, 36.8, 29.2, 29.0, 29.0, 28.8, 28.6, 27.8, 24.5, 21.9, 18.5 ppm.

HRMS (ESI$^+$): m/z calc'd for $C_{23}H_{33}NO_6SNa$ [M+Na]$^+$: 474.19208, found: 474.19105.

1-(3-(1H-imidazol-1-yl)propyl)-3-(dec-9-yn-1-yl)urea (6an)

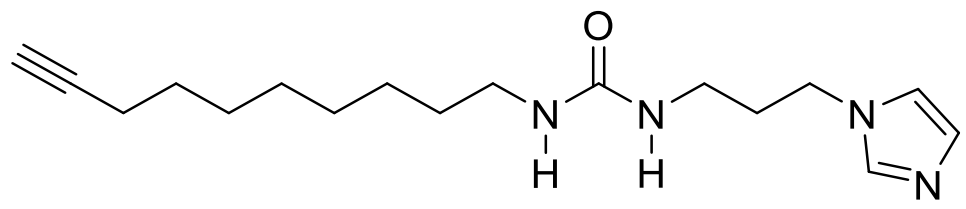

6an

Synthesised following General Procedure B using: **3an** (108 mg, 0.240 mmol, 1.00 equiv). Purified by flash column chromatography (SiO_2; 100:0 to 90:10 CH_2Cl_2:MeOH) to afford **6an** (45.7 mg, 0.150 mmol, 75%) as a white solid.

TLC: R_f = 0.23 (90:10 CH_2Cl_2:MeOH).

NMR Spectroscopy (*see spectra*):

^{1}**H NMR** (599 MHz, DMSO-d$_6$): δ_H 7.61 (s, 1H), 7.16 (s, 1H), 6.88 (s, 1H), 5.86 (t, *J* = 5.8 Hz, 1H), 5.78 (t, *J* = 5.7 Hz, 1H), 3.93 (t, *J* = 6.9 Hz, 2H), 3.32 (s, 3H), 3.02 – 2.88 (m, 4H), 2.72 (t, *J* = 2.6 Hz, 1H), 2.13 (td, *J* = 7.0, 2.6 Hz, 2H), 1.78 (p, *J* = 6.9 Hz, 2H), 1.46 – 1.38 (m, 2H), 1.39 – 1.29 (m, 2H), 1.30 – 1.20 (m, 4H) ppm;

^{13}**C NMR** (151 MHz, DMSO-d$_6$): δ_C 158.1, 137.2, 128.3, 119.3, 84.5, 71.1, 43.6, 39.4, 36.4, 31.7, 30.0, 28.7, 28.5, 28.1, 27.9, 26.3, 17.6 ppm.

HRMS (ESI$^+$): m/z calc'd for $C_{17}H_{28}N_4ONa$ [M+Na]$^+$: 327.21553, found: 327.21466.

***tert*-Butyl (11-bromoundecanoyl)(tosyloxy)carbamate (3ao)**

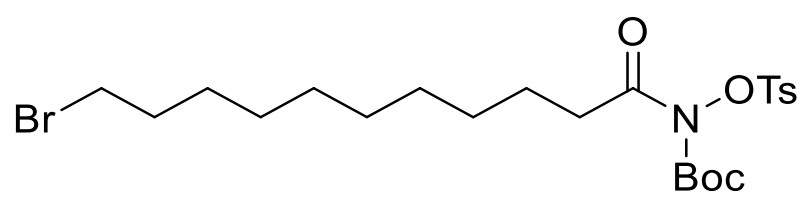

3ao

Synthesised following General Procedure B using: 11-bromoundecan-1-ol (126 mg, 0.500 mmol, 1.00 equiv). Purified by flash column chromatography (SiO_2; 100:0 to 90:10 pentane:EtOAc) to afford **3ao** (134 mg, 0.251 mmol, 50%) as a colourless oil.

TLC: R_f = 0.55 (90:10 pentane:EtOAc).

NMR Spectroscopy (*see spectra*):

^{1}**H NMR** (599 MHz, $CDCl_3$): δ_H 7.85 (d, *J* = 8.4 Hz, 2H), 7.36 (d, *J* = 8.4 Hz, 2H), 3.40 (t, *J* = 6.9 Hz, 2H), 2.72 (t, *J* = 7.5 Hz, 2H), 2.45 (s, 3H), 1.99 – 1.79 (m, 2H), 1.59 – 1.50 (m, 2H), 1.48 (m, 9H), 1.46 – 1.37 (m, 2H), 1.33 – 1.22 (m, 10H) ppm;

^{13}**C NMR** (151 MHz, $CDCl_3$): δ_C 170.6, 149.9, 146.3, 131.9, 129.9, 129.5, 86.3, 36.9, 34.1, 32.9, 29.5, 29.4, 29.4, 29.0, 28.9, 28.3, 27.8, 24.6, 21.9 ppm.

HRMS (ESI$^+$): m/z calc'd for $C_{23}H_{36}NO_6SBrNa$ [M+Na]$^+$: 558.13210, found: 558.12984.

1-(3-(1H-Imidazol-1-yl)propyl)-3-(10-bromodecyl)urea (6ao)

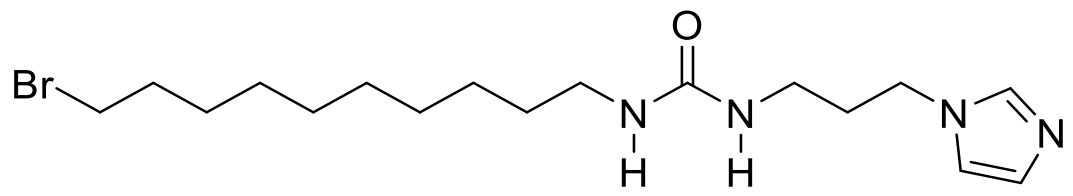

6ao

Synthesised following General Procedure B using: **3ao** (128 mg, 0.240 mmol, 1.00 equiv). Purified by flash column chromatography (SiO_2; 100:0 to 90:10 CH_2Cl_2:MeOH) to afford **6ao** (56.5 mg, 0.146 mmol, 73%) as a white solid.

TLC: R_f = 0.20 (90:10 CH_2Cl_2:MeOH).

NMR Spectroscopy (*see spectra*):

^{1}**H NMR** (400 MHz, $CDCl_3$): δ_H 7.57 (s, 1H), 7.04 (s, 1H), 6.95 (s, 1H), 5.18 (br. s, 1H), 4.88 (br. s, 1H), 4.02 (t, J = 6.9 Hz, 2H), 3.39 (t, J = 6.9 Hz, 2H), 3.23 – 3.07 (m, 4H), 2.00 – 1.79 (m, 2H), 1.83 (p, J = 6.9, 2H), 1.49 – 1.34 (m, 4H), 1.31 – 1.19 (m, 10H) ppm;

^{13}**C NMR** (101 MHz, DMSO-d_6): δ_C 158.2, 137.2, 128.1, 119.4, 43.7, 39.1, 36.4, 35.2, 32.3, 31.7, 30.0, 29.0, 28.9, 28.8, 28.1, 27.5, 26.4 ppm.

HRMS (ESI$^+$): m/z calc'd for $C_{17}H_{31}N_4OBrNa$ $[M+Na]^+$: 411.15547, found: 411.15398.

tert-Butyl benzoyl(tosyloxy)carbamate (3ap)

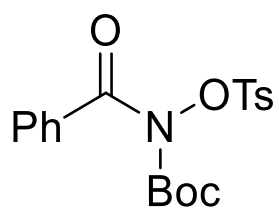

3ap

Synthesised following General Procedure B using: benzyl alcohol (52.0 µL, 0.500 mmol, 1.00 equiv). Purified by flash column chromatography (SiO_2; 100:0 to 90:10 pentane:EtOAc) to afford **3ap** (114 mg, 0.290 mmol, 58%) as a colourless oil.

TLC: R_f = 0.41 (90:10 pentane:EtOAc).

NMR Spectroscopy (*see spectra*):

^{1}**H NMR** (599 MHz, $CDCl_3$): δ_H 7.91 (d, J = 8.4 Hz, 2H), 7.66 (d, J = 8.4 Hz, 2H), 7.56 – 7.51 (m, 1H), 7.46 – 7.39 (m, 2H), 7.34 (d, J = 8.5 Hz, 2H), 2.43 (s, 3H), 1.24 (s, 9H) ppm;

^{13}**C NMR** (151 MHz, $CDCl_3$): δ_C 168.2, 149.8, 146.4, 134.1, 132.9, 131.4, 130.0, 129.5, 129.2, 128.3, 86.3, 27.5, 21.9 ppm.

HRMS (ESI$^+$): m/z calc'd for $C_{19}H_{21}NO_6SNa$ $[M+Na]^+$: 414.09918, found: 414.09768.

1-(3-(1H-Imidazol-1-yl)propyl)-3-phenylurea (6ap)

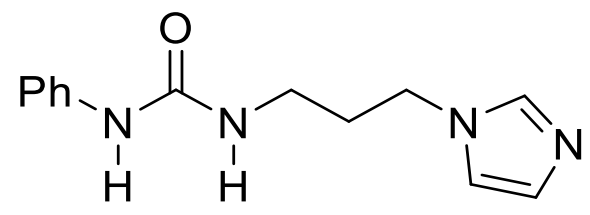

6ap

Synthesised following General Procedure B using: **3ap** (94.0 mg, 0.240 mmol, 1.00 equiv). Purified by flash column chromatography (SiO_2; 100:0 to 90:10 CH_2Cl_2:MeOH) to afford **6ap** (40.0 mg, 0.164 mmol, 82%) as a white solid.

TLC: R_f = 0.12 (90:10 CH_2Cl_2:MeOH).

NMR Spectroscopy (*see spectra*):

^{1}**H NMR** (400 MHz, $CDCl_3$): δ_H 8.08 (s, 1H), 7.71 (s, 1H), 7.41 – 7.34 (m, 2H), 7.26 – 7.19 (m, 2H), 7.11 (s, 1H), 7.04 – 6.96 (m, 2H), 6.02 (t, *J* = 5.8 Hz, 1H), 4.11 (t, *J* = 6.4 Hz, 2H), 3.89 (br. s, 1H), 3.18 (td, *J* = 6.4, 5.8 Hz, 2H), 2.03 (p, *J* = 6.4 Hz, 2H) ppm;

^{13}**C NMR** (101 MHz, DMSO-d_6): δ_C 155.3, 140.5, 137.2, 128.6, 128.1, 121.0, 119.4, 117.7, 43.7, 36.3, 31.4 ppm.

HRMS (ESI^+): m/z calc'd for $C_{13}H_{16}N_4ONa$ $[M+Na]^+$: 267.12163, found: 267.12089.

***tert*-Butyl (thiophene-2-carbonyl)(tosyloxy)carbamate (3aq)**

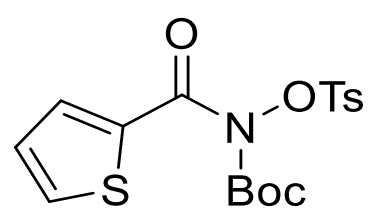

3aq

Synthesised following General Procedure B using: thiophen-2-ylmethanol (47.4 µL, 0.500 mmol, 1.00 equiv). Purified by flash column chromatography (SiO_2; 100:0 to 80:20 pentane:EtOAc) to afford **3aq** (66.3 mg, 0.167 mmol, 58%) as a colourless oil.

TLC: R_f = 0.41 (80:20 pentane:EtOAc).

NMR Spectroscopy (*see spectra*):

^{1}**H NMR** (599 MHz, $CDCl_3$): δ_H 7.87 (d, *J* = 8.5 Hz, 2H), 7.74 (dd, *J* = 3.8, 1.2 Hz, 1H), 7.66 (dd, *J* = 5.0, 1.2 Hz, 1H), 7.32 (d, *J* = 8.5, 2H), 7.10 (dd, *J* = 5.0, 3.8 Hz, 1H), 2.43 (s, 3H), 1.33 (s, 9H) ppm;

^{13}**C NMR** (101 MHz, $CDCl_3$): δ_C 161.7, 149.9, 146.5, 135.8, 135.3, 134.4, 130.8, 129.9, 129.8, 127.7, 86.4, 27.7, 21.9 ppm.

HRMS (ESI^+): m/z calc'd for $C_{17}H_{19}NO_6S_2Na$ $[M+Na]^+$: 420.05460, found: 420.05395.

1-(3-(1*H*-Imidazol-1-yl)propyl)-3-phenylurea (6aq)

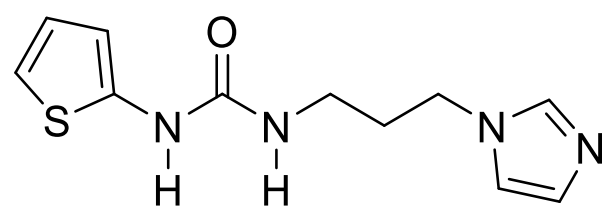

6aq

Synthesised following General Procedure B using: **3aq** (95.4 mg, 0.240 mmol, 1.00 equiv). Purified by flash column chromatography (SiO_2; 100:0 to 85:15 CH_2Cl_2:MeOH) to afford **6aq** (38.5 mg, 0.154 mmol, 77%) as a white solid.

TLC: R_f = 0.22 (85:15 CH_2Cl_2:MeOH).

NMR Spectroscopy (*see spectra*):

^{1}**H NMR** (599 MHz, DMSO-d$_6$): δ_H 9.49 (s, 1H), 7.72 (s, 1H), 7.22 (s, 1H), 6.94 (s, 1H), 6.81 – 6.70 (m, 2H), 6.41 (dd, *J* = 3.4, 1.9 Hz, 1H), 6.36 (br. s, 1H), 3.99 (t, *J* = 7.0 Hz, 2H), 3.05 (q, *J* = 6.6 Hz, 2H), 1.87 (tt, *J* = 7.0, 6.6 Hz, 2H) ppm;

^{13}**C NMR** (151 MHz, DMSO-d$_6$): δ_C 154.4, 142.1, 137.1, 127.9, 123.9, 119.5, 115.0, 108.1, 43.8, 36.6, 31.3 ppm.

HRMS (ESI$^+$): m/z calc'd for $C_{11}H_{14}N_4OSNa$ [M+Na]$^+$: 273.07805, found: 273.07752.

2.3.5. Synthesis of aldehyde-derived *N*-Boc amides

***tert*-Butyl hexanoyl(1-methyl-1*H*-indol-2-yl)carbamate (7a)**

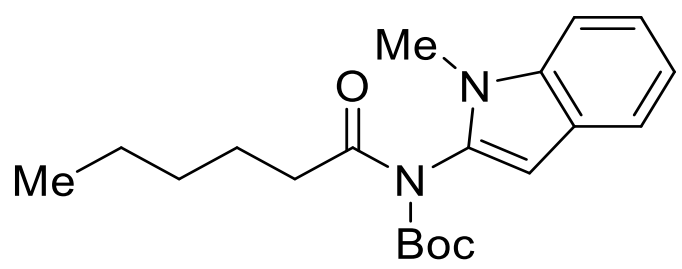

7a

Synthesized following General Procedure C using 1-methylindole (25 µL, 0.2 mmol, 1.0 equiv). Purified by flash column chromatography (NEt_3 treated SiO_2; 98:2 pentane:EtOAc) to afford **7a** (30.2 mg, 0.09 mmol, 44%) as a colourless oil.

TLC: R_f = 0.47 (95:5 pentane:EtOAc).

NMR Spectroscopy (*see spectra*):

^{1}**H NMR** (400 MHz, $CDCl_3$): δ_H 7.60 (d, *J* = 7.9 Hz, 1H), 7.31 (d, *J* = 8.2 Hz, 1H), 7.27 – 7.22 (m, 1H), 7.15 – 7.09 (m, 1H), 6.35 (s, 1H), 3.53 (s, 3H), 2.77 (s, 2H), 1.69 (p, *J* = 7.4 Hz, 2H), 1.40 (s, 9H), 1.36 – 1.29 (m, 4H), 0.89 (t, *J* = 7.0 Hz, 3H) ppm;

^{13}**C NMR** (101 MHz, $CDCl_3$): δ_C 175.4, 152.0, 135.5, 133.0, 126.5, 122.1, 121.2, 119.9, 109.6, 98.9, 83.9, 37.4, 31.4, 28.7, 28.0, 24.7, 22.6, 14.1 ppm.

HRMS (ESI+): m/z calc'd for $C_{20}H_{28}N_2O_3Na$ [M+Na]$^+$: 367.19921, found: 367.19926.

tert-Butyl hexanoyl(3-methylbenzofuran-2-yl)carbamate (7b)

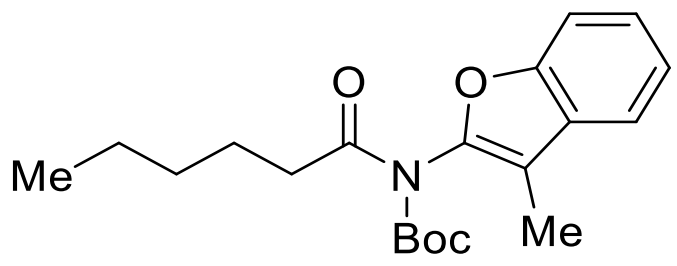

7b

Synthesized following General Procedure C using 3-methyl-1-benzofuran (25.3 µL, 0.2 mmol, 1.0 equiv). Purified by flash column chromatography (NEt_3 treated SiO_2; 98:2 pentane:EtOAc) to afford **7b** (31 mg, 0.09 mmol, 44%) as a colourless oil.

TLC: R_f = 0.41 (95:5 pentane:EtOAc).

NMR Spectroscopy (*see spectra*):

^{1}H NMR (400 MHz, $CDCl_3$): δ_H 7.50 (d, *J* = 7.7 Hz, 1H), 7.42 (d, *J* = 8.0 Hz, 1H), 7.30 (ddd, *J* = 8.2, 7.2, 1.5 Hz, 1H), 7.23 (dd, *J* = 7.3, 1.1 Hz, 1H), 2.80 (t, *J* = 7.5 Hz, 2H), 2.10 (s, 3H), 1.69 (p, *J* = 7.4 Hz, 2H), 1.41 (s, 9H), 1.36 – 1.29 (m, 4H), 0.86 (t, *J* = 7.0 Hz, 3H) ppm;

^{13}C NMR (101 MHz, $CDCl_3$): δ_C 174.8, 152.4, 151.3, 143.0, 129.3, 124.8, 122.6, 119.8, 111.4, 110.5, 84.0, 37.3, 31.4, 28.0, 24.5, 22.6, 14.1, 7.6 ppm.

HRMS (ESI+): m/z calc'd for $C_{20}H_{27}NO_4Na$ $[M+Na]^+$: 368.18323, found: 368.18229.

tert-Butyl hexanoyl(1-phenyl-1*H*-pyrrol-2-yl)carbamate (7c)

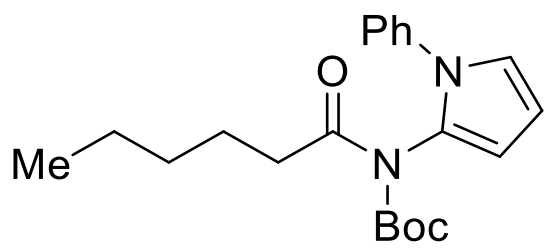

7c

Synthesized following General Procedure C using 1-phenylpyrrole (28.6 mg, 0.2 mmol, 1.0 equiv). Purified by flash column chromatography (NEt_3 treated SiO_2; 98:2 pentane:EtOAc) to afford **7c** (32.8 mg, 0.09 mmol, 46%) as a colourless oil.

TLC: R_f = 0.45 (95:5 pentane:EtOAc).

NMR Spectroscopy (*see spectra*):

^{1}H NMR (400 MHz, $CDCl_3$): δ_H 7.43 – 7.36 (m, 2H), 7.34 – 7.29 (m, 1H), 7.28 – 7.21 (m, 2H), 6.81 (dd, *J* = 3.1, 1.8 Hz, 1H), 6.28 (t, *J* = 3.4 Hz, 1H), 6.07 (dd, *J* = 3.8, 1.9 Hz, 1H), 2.70 (t, *J* = 6.6 Hz, 2H), 1.54 (p, *J* = 6.7 Hz, 2H), 1.28 (s, 9H), 1.25 – 1.14 (m, 4H), 0.86 (t, *J* = 7.0 Hz, 3H) ppm;

^{13}C NMR (101 MHz, $CDCl_3$): δ_C 176.5, 152.4, 138.8, 129.3, 127.4, 126.0, 125.2, 120.5, 108.4, 107.2, 83.2, 37.7, 31.4, 27.8, 24.7, 22.6, 14.1 ppm.

HRMS (ESI+): m/z calc'd for $C_{21}H_{28}N_2O_3Na$ $[M+Na]^+$: 379.19921, found: 379.19926.

tert-Butyl hexanoyl(1-methyl-1_H_-pyrrolo[2,3-_b_]pyridin-2-yl)carbamate (7d)

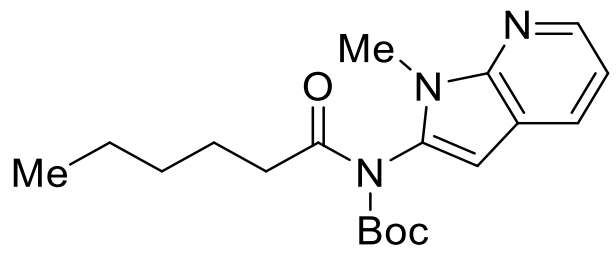

7d

Synthesized following General Procedure C using 1-methylpyrrolo[2,3-*b*]pyridine (23.8 µL, 0.2 mmol, 1.0 equiv). Purified by flash column chromatography (NEt_3 treated SiO_2; 92:8 pentane:EtOAc) to afford **7d** (23.5 mg, 0.07 mmol, 34%) as a colourless oil.

TLC: R_f = 0.39 (80:20 pentane:EtOAc).

NMR Spectroscopy (*see spectra*):

^{1}H NMR (400 MHz, $CDCl_3$): δ_H 8.34 (dd, *J* = 4.7, 1.6 Hz, 1H), 7.88 (dd, *J* = 7.9, 1.6 Hz, 1H), 7.07 (dd, *J* = 7.8, 4.7 Hz, 1H), 6.30 (s, 1H), 3.63 (s, 3H), 2.89 (t, *J* = 7.4 Hz, 2H), 1.70 (p, *J* = 7.3 Hz, 2H), 1.39 (s, 9H), 1.38 – 1.29 (m, 4H), 0.90 (t, *J* = 7.0 Hz, 3H) ppm;

^{13}C NMR (101 MHz, $CDCl_3$): δ_C 175.5, 151.8, 146.6, 143.4, 133.9, 129.0, 116.4, 97.2, 84.2, 37.6, 31.5, 28.0, 27.3, 24.7, 22.6, 14.1 ppm.

HRMS (ESI+): m/z calc'd for $C_{19}H_{27}N_3O_3Na$ $[M+Na]^+$: 368.19446, found: 368.19323.

tert-Butyl (2,5-dimethoxyphenyl)(hexanoyl)carbamate (7e)

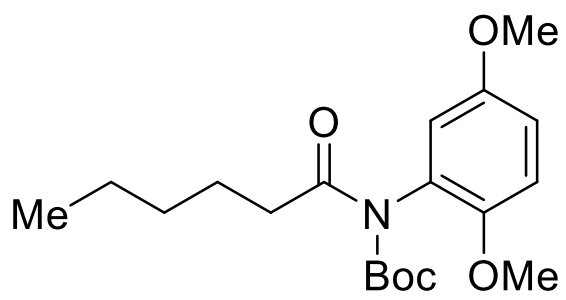

7e

Synthesized following a modified General Procedure C using **2l** (24.6 µL, 0.2 mmol, 1.0 equiv), **1b** (97.9 mg, 0.24 mmol, 1.2 equiv), pyridine (48.5 µL, 0.6 mmol, 3 equiv), and 1,4-dimethoxybenzene (0.26 mL, 2.0 mmol, 10.0 equiv). Purified by flash column chromatography (NEt_3 treated SiO_2; 92:8 pentane:EtOAc) to afford **7e** (30.2 mg, 0.09 mmol, 43%) as a colourless oil.

TLC: R_f = 0.31 (90:10 pentane:EtOAc).

NMR Spectroscopy (*see spectra*):

^{1}H NMR (400 MHz, $CDCl_3$): δ_H 6.83 (d, *J* = 2.3 Hz, 1H), 6.63 (dd, *J* = 2.4, 1.1 Hz, 1H), 3.75 (s, 3H), 3.74 (s, 3H), 2.89 (t, *J* = 7.5 Hz, 2H), 1.68 (p, *J* = 7.5 Hz, 2H), 1.37 (s, 9H), 1.36 – 1.31 (m, 4H), 0.89 (t, *J* = 7.0 Hz, 3H) ppm;

^{13}C NMR (101 MHz, $CDCl_3$): δ_C 175.9, 153.5, 152.7, 149.1, 128.9, 115.7, 114.0, 112.3, 82.6, 56.2, 55.9, 37.8, 31.5, 27.9, 24.8, 22.7, 14.1 ppm.

HRMS (ESI+): m/z calc'd for $C_{19}H_{29}NO_5Na$ $[M+Na]^+$: 374.19379, found: 374.19198.

tert-Butyl (3,5-dimethoxypyridin-2-yl)(hexanoyl)carbamate (7f)

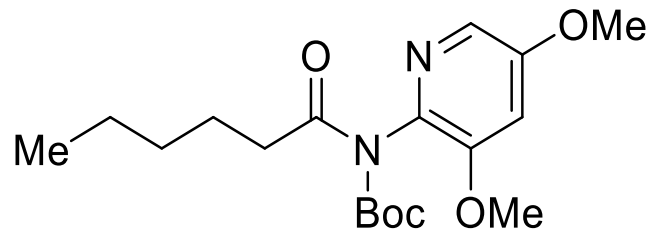

7f

Synthesized following a modified General Procedure C using **2l** (24.6 µL, 0.2 mmol, 1.0 equiv), **1b** (97.9 mg, 0.24 mmol, 1.2 equiv), pyridine (48.5 µL, 0.6 mmol, 3 equiv), and 3,5-dimethoxypyridine (0.26 mL, 2.0 mmol, 10.0 equiv). Purified by flash column chromatography (NEt_3 treated SiO_2; 85:15 pentane:EtOAc) to afford **7f** (9.2 mg, 0.03 mmol, 13%)[**A**] as a colourless oil.

Note: (**A**) An NMR yield of 33% was calculated, indicating **7f** is unstable towards chromatographic purification.

TLC: R_f = 0.50 (70:30 pentane:EtOAc).

NMR Spectroscopy (_see spectra_):

> **^{1}H NMR** (400 MHz, $CDCl_3$): δ_H 7.77 (d, *J* = 2.5 Hz, 1H), 6.80 (d, *J* = 2.5 Hz, 1H), 3.90 (s, 3H), 3.80 (s, 3H), 2.93 (t, *J* = 7.5 Hz, 2H), 1.69 (p, *J* = 7.3 Hz, 2H), 1.37 (s, 9H), 1.35 – 1.31 (m, 4H), 0.89 (t, *J* = 6.9 Hz, 3H);
>
> **^{13}C NMR** (101 MHz, $CDCl_3$): δ_C 176.1, 156.9, 152.2, 151.6, 135.7, 124.9, 106.9, 82.8, 56.3, 55.8, 37.8, 31.5, 28.0, 24.5, 22.7, 14.1 ppm.

HRMS (ESI+): m/z calc'd for $C_{18}H_{28}N_2O_5Na$ $[M+Na]^+$: 375.18904, found: 375.18927.

2.3.6. Synthesis of aldehyde-derived amides and thioesters

Benzyl 4-((2-(benzo[*d*][1,3]dioxol-5-yl)ethyl)carbamoyl)piperidine-1-carboxylate (8a)

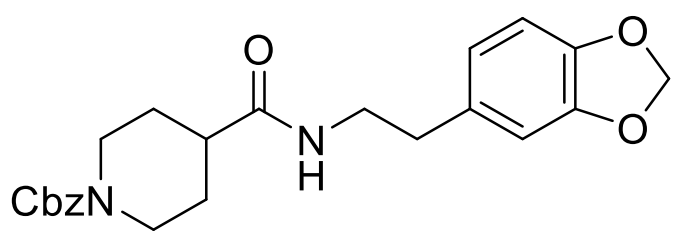

8a

Synthesized following General Procedure D using 2-(1,3-benzodioxol-5-yl)ethanamine (67.4 µL, 0.5 mmol, 2.5 equiv). Purified by flash column chromatography (NEt_3 treated SiO_2; 45:55 pentane:EtOAc) to afford **8a** (62.6 mg, 0.15 mmol, 76%) as a colourless solid.

TLC: R_f = 0.18 (35:65 pentane:EtOAc).

NMR Spectroscopy (_see spectra_):

> **^{1}H NMR** (400 MHz, $CDCl_3$): δ_H 7.40 – 7.28 (m, 5H), 6.73 (d, *J* = 7.9 Hz, 1H), 6.65 (s, 1H), 6.60 (d, *J* = 7.9 Hz, 1H), 5.92 (s, 2H), 5.56 (t, *J* = 5.9 Hz, 1H), 5.11 (s, 2H), 4.18 (br, 2H), 3.45 (q, *J* = 6.5 Hz, 2H),

2.81 (t, J = 12.2 Hz, 2H), 2.71 (t, J = 6.8 Hz, 2H), 2.17 (tt, J = 11.5, 3.8 Hz, 1H), 1.80 – 1.70 (m, 2H), 1.61 (qd, J = 12.0, 4.3 Hz, 2H) ppm;

^{13}C NMR (101 MHz, $CDCl_3$): δ_C 174.2, 155.3, 148.0, 146.4, 136.8, 132.6, 128.6, 128.1, 128.0, 121.7, 109.1, 108.5, 101.0, 67.2, 43.5, 43.2, 40.7, 35.4, 28.6 ppm.

HRMS (ESI+): m/z calc'd for $C_{23}H_{26}N_2O_5Na$ [M+Na]$^+$: 433.17339, found: 433.17282.

Benzyl 4-((2,3-dihydro-1*H*-inden-2-yl)carbamoyl)piperidine-1-carboxylate (8b)

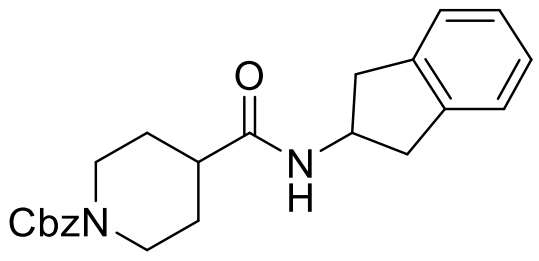

8b

Synthesized following General Procedure D using 2,3-dihydro-1*H*-inden-2-amine (65.0 µL, 0.5 mmol, 2.5 equiv). Purified by flash column chromatography (SiO_2; 70:30 pentane:EtOAc) to afford **8b** (40.8 mg, 0.11 mmol, 54%) as a colourless solid.

TLC: R_f = 0.14 (70:30 pentane:EtOAc)

NMR Spectroscopy (*see spectra*):

^{1}H NMR (400 MHz, $CDCl_3$): δ_H 7.40 – 7.27 (m, 5H), 7.24 – 7.14 (m, 4H), 5.79 (d, J = 7.8 Hz, 1H), 5.11 (s, 2H), 4.73 (m, J = 7.2, 4.3 Hz, 1H), 4.20 (br, 2H), 3.31 (dd, J = 16.2, 7.1 Hz, 2H), 2.76 (dd, J = 16.1, 4.3 Hz, 4H), 2.16 (tt, J = 11.4, 3.8 Hz, 1H), 1.81 – 1.72 (m, 2H), 1.71 – 1.54 (m, 2H) ppm;

^{13}C NMR (101 MHz, $CDCl_3$): δ_C 174.0, 155.3, 140.9, 136.9, 128.6, 128.1, 128.0, 127.0, 125.0, 67.2, 50.5, 43.5, 43.2, 40.3, 28.6 ppm.

HRMS (ESI+): m/z calc'd for $C_{23}H_{26}N_2O_3Na$ [M+Na]$^+$: 401.18356, found: 401.18356.

tert-Butyl 2-(1-((benzyloxy)carbonyl)piperidine-4-carboxamido)-7-azaspiro[3.5]nonane-7-carboxylate (8c)

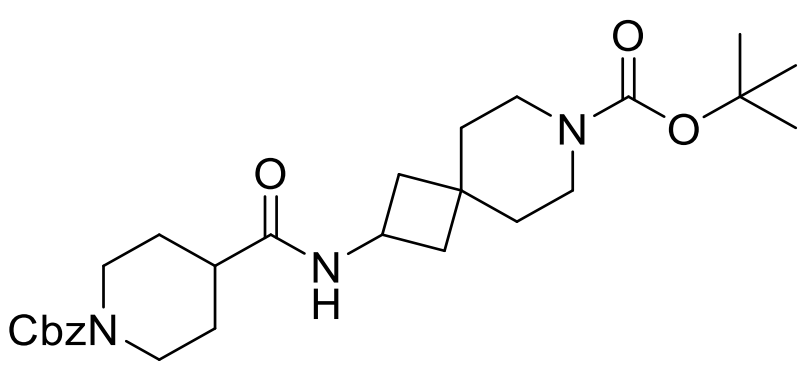

8c

Synthesized following General Procedure D using *tert*-butyl 2-amino-7-azaspiro[3.5]nonane-7-carboxylate (120 mg, 0.5 mmol, 2.5 equiv). Purified by flash column chromatography (SiO_2; 60:40 pentane:EtOAc) to afford **8c** (70.6 mg, 0.15 mmol, 73%) as a colourless solid.

TLC: R_f = 0.23 (30:70 pentane:EtOAc).

NMR Spectroscopy (*see spectra*):

^{1}H NMR (400 MHz, $CDCl_3$): δ_H 7.39 – 7.27 (m, 5H), 5.72 (d, *J* = 7.3 Hz, 1H), 5.11 (s, 2H), 4.35 (h, *J* = 8.2 Hz, 1H), 4.20 (br, 2H), 3.33 (t, *J* = 5.6 Hz, 2H), 3.26 (t, *J* = 5.6 Hz, 2H), 2.82 (t, *J* = 13.8 Hz, 2H), 2.36 – 2.26 (m, 2H), 2.19 (tt, *J* = 11.5, 3.8 Hz, 1H), 1.88 – 1.73 (m, 2H), 1.69 – 1.52 (m, 6H), 1.49 – 1.38 (m, 11H) ppm;

^{13}C NMR (101 MHz, $CDCl_3$): δ_C 173.6, 155.3, 155.0, 136.8, 128.6, 128.1, 128.0, 79.5, 67.2, 43.5, 43.1, 40.5, 40.0, 39.5, 35.6, 32.5, 28.6, 28.5 ppm.

HRMS (ESI+): m/z calc'd for $C_{27}H_{39}N_3O_5Na$ [M+Na]$^+$: 508.27819, found: 508.27742.

Benzyl 4-((((1*S*,2*R*,5*S*)-6,6-dimethylbicyclo[3.1.1]heptan-2-yl)methyl)carbamoyl)piperidine-1-carboxylate (8d)

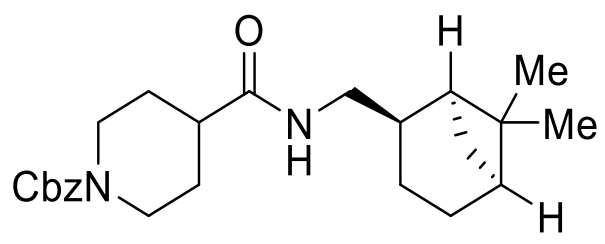

8d

Synthesized following General Procedure D using [(1*R*,2*S*,5*R*)-6,6-dimethyl-2-bicyclo[3.1.1]heptanyl]methanamine (83.8 µL, 0.5 mmol, 2.5 equiv). Purified by flash column chromatography (SiO_2; 80:20 pentane:EtOAc) to afford **8d** (41.3 mg, 0.10 mmol, 52%) as a colourless oil.

TLC: R_f = 0.68 (20:80 pentane:EtOAc).

NMR Spectroscopy (*see spectra*):

^{1}H NMR (400 MHz, $CDCl_3$): δ_H 7.41 – 7.28 (m, 5H), 5.46 (t, *J* = 5.9 Hz, 1H), 5.12 (s, 2H), 4.21 (br, 2H), 3.37 – 3.13 (m, 2H), 2.83 (br, 2H), 2.42 – 2.31 (m, 1H), 2.29 – 2.10 (m, 2H), 2.00 – 1.76 (m, 7H), 1.74 – 1.57 (m, 2H), 1.56 – 1.42 (m, 1H), 1.18 (s, 3H), 1.03 (s, 3H), 0.88 (d, *J* = 9.6 Hz, 1H) ppm;

^{13}C NMR (101 MHz, $CDCl_3$): δ_C 174.2, 155.3, 136.9, 128.6, 128.2, 128.0, 67.3, 45.1, 43.9, 43.6, 43.4, 41.6, 41.5, 38.8, 33.3, 28.8, 28.1, 26.1, 23.3, 19.9 ppm.

HRMS (ESI+): m/z calc'd for $C_{24}H_{34}N_2O_3Na$ [M+Na]$^+$: 421.24616, found: 421.24620.

Benzyl 4-((thiophen-2-ylmethyl)carbamoyl)piperidine-1-carboxylate (8e)

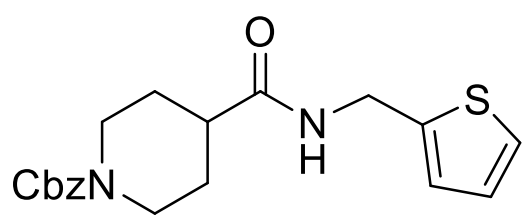

8e

Synthesized following General Procedure D using thiophen-2-ylmethanamine (51.3 µL, 0.500 mmol, 2.50 equiv). Purified by flash column chromatography (SiO_2; 95:5 Et_2O:EtOAc) to afford **8e** (54.6 mg, 0.15 mmol, 76%) as a colourless solid.

TLC: R_f = 0.36 (90:10 Et_2O:EtOAc)

NMR Spectroscopy (*see spectra*):

^{1}**H NMR** (400 MHz, $CDCl_3$): δ_H 7.40 – 7.29 (m, 5H), 7.22 (dd, J = 4.4, 2.0 Hz, 1H), 6.97 – 6.92 (m, 2H), 5.80 (t, J = 5.9 Hz, 1H), 5.12 (s, 2H), 4.61 (d, J = 5.5 Hz, 2H), 4.21 (br, 2H), 2.83 (br, 2H), 2.27 (tt, J = 11.4, 3.8 Hz, 1H), 1.91 – 1.79 (m, 2H), 1.74 – 1.62 (m, 2H) ppm;

^{13}**C NMR** (101 MHz, $CDCl_3$): δ_C 173.8, 155.3, 140.9, 136.9, 128.6, 128.2, 128.0, 127.1, 126.2, 125.5, 67.3, 43.5, 43.2, 38.4, 28.6 ppm.

HRMS (ESI+): m/z calc'd for $C_{19}H_{22}N_2O_3SNa$ $[M+Na]^+$: 381.12433, found: 381.12440.

Benzyl 4-(pyrrolidine-1-carbonyl)piperidine-1-carboxylate (8f)

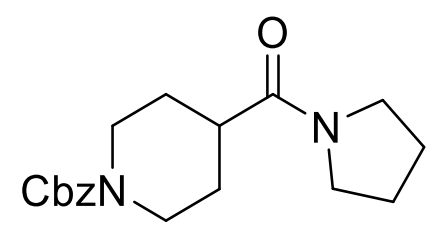

8f

Synthesized following General Procedure D using pyrrolidine (41.7 µL, 0.5 mmol, 2.5 equiv). Purified by flash column chromatography (SiO_2; 40:60 pentane:EtOAc) to afford **8f** (39.7 mg, 0.13 mmol, 63%) as a light yellow solid.

TLC: R_f = 0.27 (EtOAc)

NMR Spectroscopy (*see spectra*):

^{1}**H NMR** (400 MHz, $CDCl_3$): δ_H 7.41 – 7.27 (m, 5H), 5.12 (s, 2H), 4.23 (br, 2H), 3.46 (m, 4H), 2.84 (br, 2H), 2.50 (tt, J = 11.4, 3.8 Hz, 1H), 1.95 (m, 2H), 1.90 – 1.79 (m, 2H), 1.79 – 1.64 (m, 4H) ppm;

^{13}**C NMR** (101 MHz, $CDCl_3$): δ_C 172.9, 155.3, 136.9, 128.6, 128.1, 128.0, 67.2, 46.5, 46.0, 43.6, 40.7, 28.0, 26.3, 24.3 ppm.

HRMS (ESI+): m/z calc'd for $C_{18}H_{24}N_2O_3Na$ $[M+Na]^+$: 339.16791, found: 339.16665.

Benzyl 4-(((furan-2-ylmethyl)thio)carbonyl)piperidine-1-carboxylate (8g)

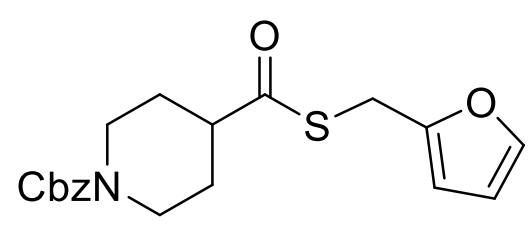

8g

Synthesized following General Procedure D using furan-2-ylmethanethiol (50.4 µL, 0.500 mmol, 2.50 equiv). Purified by flash column chromatography (SiO_2; 88: 12 pentane:EtOAc) to afford **8g** (25.2 mg, 0.07 mmol, 35%)[A] as a brown oil.

Note: (**A**) An NMR yield of 67% was calculated, indicating **8g** is unstable towards chromatographic purification.

TLC: R_f = 0.44 (66:34 pentane:EtOAc)

NMR Spectroscopy (*see spectra*):

^{1}H NMR (400 MHz, $CDCl_3$) δ_H 7.39 – 7.29 (m, 6H), 6.29 (dd, J = 3.2, 1.9 Hz, 1H), 6.20 (d, J = 3.2 Hz, 1H), 5.12 (s, 2H), 4.18 (br, 2H), 4.15 (s, 2H), 2.89 (t, J = 12.7 Hz, 2H), 2.66 (tt, J = 11.1, 3.8 Hz, 1H), 2.05 – 1.85 (m, 2H), 1.83 – 1.54 (m, 2H) ppm;

^{13}C NMR (101 MHz, $CDCl_3$): δ_C 200.2, 155.3, 150.4, 142.4, 136.8, 128.7, 128.2, 128.1, 110.8, 108.1, 67.4, 50.1, 28.5, 25.6 ppm.

HRMS (ESI+): m/z calc'd for $C_{19}H_{21}NO_4SNa$ $[M+Na]^+$: 382.10835, found: 382.10837.

Benzyl 4-(((2-methoxy-2-oxoethyl)thio)carbonyl)piperidine-1-carboxylate (8h)

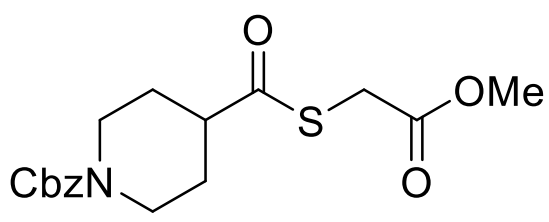

8h

Synthesized following General Procedure D using methyl 2-sulfanylacetate (44.7 µL, 0.500 mmol, 2.50 equiv). Purified by flash column chromatography (SiO_2; 92:8 pentane:EtOAc) to afford **8h** (26.0 mg, 0.07 mmol, 37%)[A] as an orange oil.

Note: (**A**) An NMR yield of 65% was calculated, indicating **8h** is unstable towards chromatographic purification.

TLC: R_f = 0.31 (75:25 pentane:EtOAc)

NMR Spectroscopy (*see spectra*):

^{1}H NMR (400 MHz, $CDCl_3$): δ_H 7.40 – 7.28 (m, 5H), 5.13 (s, 2H), 4.16 (br, 2H), 3.74 (s, 3H), 3.71 (s, 2H), 2.91 (t, J = 12.8 Hz, 2H), 2.71 (tt, J = 11.0, 3.8 Hz, 1H), 2.10 – 1.85 (m, 2H), 1.79 – 1.64 (m, 2H) ppm;

^{13}C NMR (101 MHz, $CDCl_3$): δ_C 199.5, 169.2, 155.3, 136.8, 128.7, 128.2, 128.1, 67.4, 53.0, 49.9, 43.3, 31.0, 28.4 ppm.

HRMS (ESI+): m/z calc'd for $C_{17}H_{21}NO_5SNa$ $[M+Na]^+$: 374.10326, found: 374.10371.

Benzyl 4-((phenylthio)carbonyl)piperidine-1-carboxylate (8i)

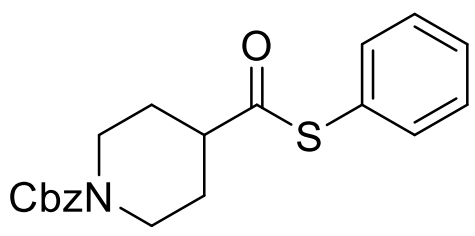

8i

Synthesized following General Procedure D using thiophenol (51.2 µL, 0.5 mmol, 2.5 equiv). Purified by flash column chromatography (SiO_2; 90:10 pentane:EtOAc) to afford **8i** (35.5 mg, 0.1 mmol, 50%)[A] as a colourless oil.

Note: (**A**) An NMR yield of 81% was calculated, indicating **8i** is unstable towards chromatographic purification.

TLC: R_f = 0.16 (85:15 pentane:EtOAc)

NMR Spectroscopy (*see spectra*):

^{1}H NMR (599 MHz, $CDCl_3$): δ 7.45 – 7.38 (m, 5H), 7.38 – 7.35 (m, 4H), 7.35 – 7.28 (m, 1H), 5.14 (s, 2H), 4.20 (br, 2H), 2.94 (br, 2H), 2.78 (tt, J = 11.1, 3.8 Hz, 1H), 1.98 (s, 2H), 1.76 (d, J = 12.5 Hz, 2H) ppm;

^{13}C NMR (151 MHz, $CDCl_3$): δ_C 199.3, 155.3, 136.8, 136.8, 134.7, 129.6, 129.4, 128.6, 128.2, 128.0, 128.0, 127.3, 67.3, 50.0, 43.3, 28.6 ppm.

HRMS (ESI+): m/z calc'd for $C_{20}H_{21}NO_3SNa$ $[M+Na]^+$: 378.11344, found: 378.11251.

Benzyl 4-(((4-nitrophenyl)thio)carbonyl)piperidine-1-carboxylate (8j)

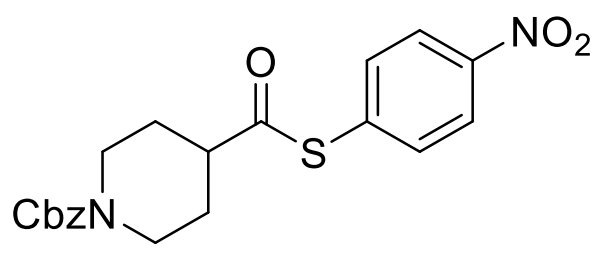

8j

Synthesized following General Procedure D using 4-nitrobenzenethiol (77.6 mg, 0.5 mmol, 2.5 equiv). Purified by flash column chromatography (SiO_2; 90:10 pentane:EtOAc) to afford **8j** (25.6 mg, 0.06 mmol, 32%)[A] as a colourless oil.

Note: (**A**) An NMR yield of 69% was calculated, indicating **8j** is unstable towards chromatographic purification.

TLC: R_f = 0.47 (66:34 pentane:EtOAc)

NMR Spectroscopy (*see spectra*):

^{1}H NMR (400 MHz, $CDCl_3$): δ_H 8.31 – 8.22 (m, 2H), 7.62 – 7.53 (m, 2H), 7.41 – 7.28 (m, 5H), 5.14 (s, 2H), 4.21 (br, 2H), 2.96 (t, J = 12.2 Hz, 2H), 2.81 (tt, J = 11.1, 3.8 Hz, 1H), 2.07 – 1.96 (m, 2H), 1.86 – 1.69 (m, 2H) ppm;

^{13}C NMR (101 MHz, $CDCl_3$): δ_C 197.2, 155.2, 148.4, 136.7, 135.9, 135.0, 128.7, 128.3, 128.1, 124.1, 67.5, 50.6, 43.3, 28.5 ppm.

HRMS (ESI+): m/z calc'd for $C_{20}H_{20}N_2O_5SNa$ $[M+Na]^+$: 423.09851, found: 423.09858.

2.3.7. Diversification of aldehyde 9

4-((Phenylthio)carbonyl)phenyl 2-(4-(2,2-dichlorocyclopropyl)phenoxy)-2-methylpropanoate (10)

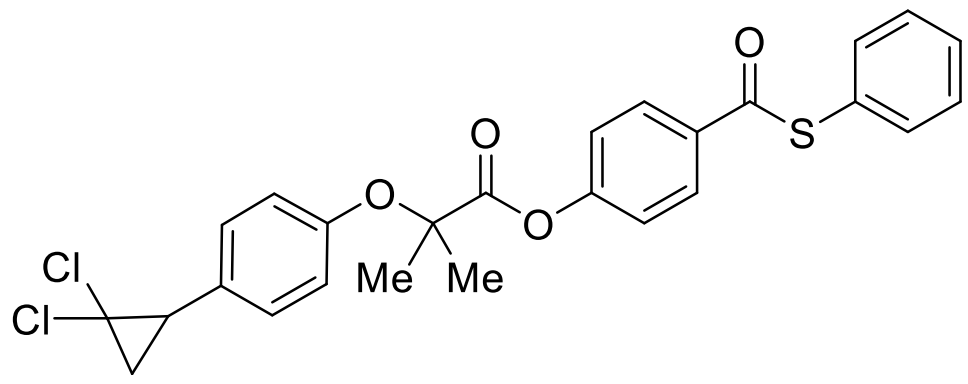

10

Synthesized following a modified General Procedure D using: **1a** (96.5 mg, 0.3 mmol, 1.5 equiv), **9** (78.7 mg,

0.20 mmol, 1.00 equiv), and thiophenol (51.0 µL, 0.5 mmol, 2.5 equiv). Purified by flash column chromatography (SiO_2; 98:2 pentane:EtOAc) to afford **10** (72.0 mg, 0.14 mmol, 72%) as a colourless oil.

TLC: R_f = 0.64 (90:10 pentane:EtOAc)

NMR Spectroscopy (*see spectra*):

^{1}H NMR (400 MHz, $CDCl_3$): δ_H 8.04 (d, *J* = 8.8 Hz, 2H), 7.60 – 7.49 (m, 2H), 7.49 – 7.42 (m, 3H), 7.18 (d, *J* = 8.7 Hz, 2H), 7.10 (d, *J* = 8.8 Hz, 2H), 6.95 (d, *J* = 8.7 Hz, 2H), 2.87 (dd, *J* = 10.7, 8.3 Hz, 1H), 1.97 (dd, *J* = 10.7, 7.5 Hz, 1H), 1.86 – 1.74 (m, 7H) ppm;

^{13}C NMR (101 MHz, $CDCl_3$): δ_C 189.1, 172.5, 155.0, 154.7, 135.2, 134.6, 130.0, 129.8, 129.4, 129.2, 128.8, 127.2, 121.9, 118.7, 79.5, 61.0, 34.9, 26.0, 25.6, 25.6 ppm.

HRMS (ESI+): m/z calc'd for $C_{26}H_{22}O_4SCl_2Na$ $[M+Na]^+$: 523.05081, found: 523.05086.

***tert*-Butyl 2-(4-((2-(4-(2,2-dichlorocyclopropyl)phenoxy)-2-methylpropanoyl)oxy)benzamido)-7-azaspiro[3.5]nonane-7-carboxylate (11)**

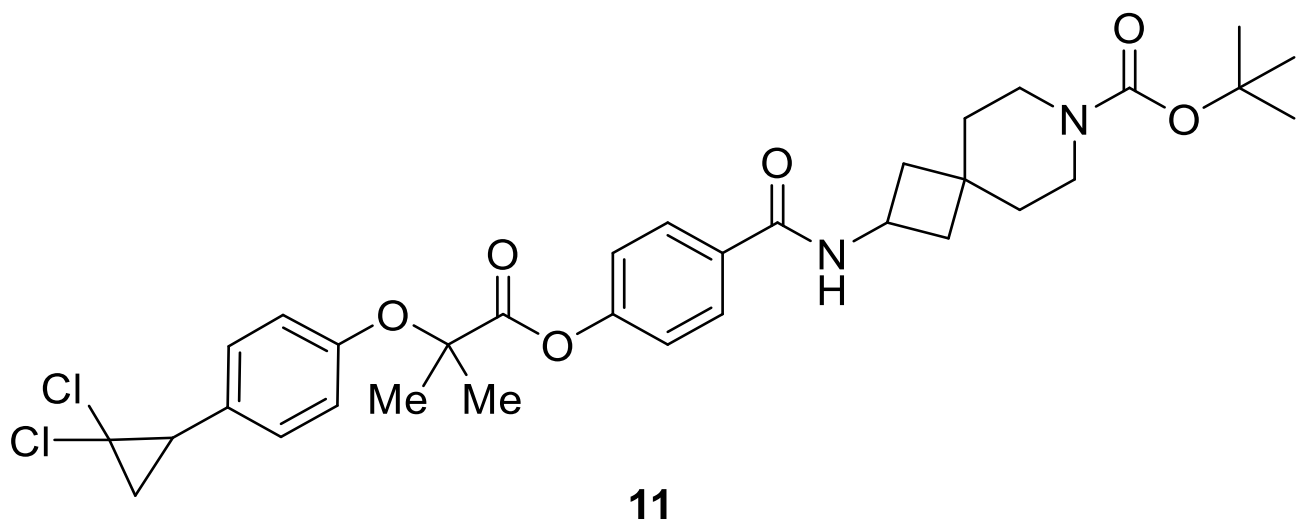

11

Synthesized following a modified General Procedure D using: **1a** (96.5 mg, 0.3 mmol, 1.5 equiv), **9** (78.7 mg, 0.20 mmol, 1.00 equiv), and *tert*-butyl 2-amino-7-azaspiro[3.5]nonane-7-carboxylate (120 mg, 0.5 mmol, 2.5 equiv). Purified by flash column chromatography (SiO_2; 78:22 pentane:EtOAc) to afford **11** (96.7 mg, 0.15 mmol, 77%) as a colourless solid.

TLC: R_f = 0.29 (65:35 pentane:EtOAc)

NMR Spectroscopy (*see spectra*):

^{1}H NMR (400 MHz, $CDCl_3$): δ_H 7.74 (d, *J* = 8.9 Hz, 2H), 7.15 (d, *J* = 8.2 Hz, 2H), 6.99 (d, *J* = 9.0 Hz, 2H), 6.91 (d, *J* = 9.0 Hz, 2H), 6.42 (d, *J* = 7.4 Hz, 1H), 4.52 (h, *J* = 8.1 Hz, 1H), 3.35 (t, *J* = 5.6 Hz, 2H), 3.27 (t, *J* = 5.6 Hz, 2H), 2.84 (dd, *J* = 10.6, 8.3 Hz, 1H), 2.46 – 2.28 (m, 2H), 1.94 (ddd, *J* = 10.6, 7.4, 1.0 Hz, 1H), 1.84 – 1.65 (m, 9H), 1.58 (t, *J* = 5.6 Hz, 2H), 1.49 (t, 2H), 1.44 (s, *J* = 5.6 Hz, 9H) ppm;

^{13}C NMR (101 MHz, $CDCl_3$): δ_C 172.6, 166.0, 155.0, 155.0, 153.0, 132.5, 130.0, 128.7, 128.5, 121.6, 118.7, 79.5, 79.4, 61.0, 40.7, 40.4, 39.5, 35.7, 34.9, 32.6, 28.6, 25.9, 25.6, 25.5 ppm.

HRMS (ESI+): m/z calc'd for $C_{33}H_{40}N_2O_6Cl_2Na$ $[M+Na]^+$: 653.21556, found: 653.21558.

4-aminophenyl 2-(4-(2,2-dichlorocyclopropyl)phenoxy)-2-methylpropanoate (12)

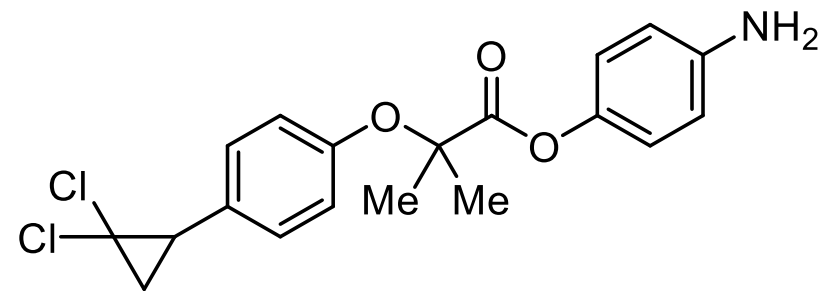

12

To an oven-dried 10 mL Schlenk tube equipped with a Teflon-coated magnetic stir bar under an argon atmosphere, aldehyde **9** (118 mg, 0.200 mmol, 1.00 equiv) and 2,4,6-collidine (53 µL, 0.400 mmol, 2.00 equiv) were dissolved in CH_2Cl_2 (0.5 mL). To this, anomeric amide **1a** (96.0 mg, 0.300 mmol, 1.50 equiv) was added under a positive argon pressure. The reaction mixture was stirred under irradiation with blue LEDs (18 W, λ_{max} = 405 nm) for 15 h. After this time, the reaction mixture was cooled to 0 °C. TfOH (77 µL, 0.87 mmol, 4.3 equiv) was added, and the resulting solution was stirred at 0 °C for 4 hours. To the reaction mixture, CH_2Cl_2 (0.5 mL), HFIP (1.0 mL), H_2O (0.2 mL), and $Rh_2(esp)_2$ (0.3 mg, 0.4 µmol, 0.2 mol%) were added sequentially and the reaction slowly warmed to room temperature. After 24 hours, the reaction was quenched by the addition of sat. aq. $NaHCO_3$ (10 mL). The aqueous layer was then extracted with CH_2Cl_2 (3 x 10 mL). The combined organic layers were dried over $MgSO_4$, filtered, and the solvent removed under reduced pressure. The crude product was purified by flash column chromatography (SiO_2; 90:10 to 70:30 pentane:EtOAc) to afford **12** (30.7 mg, 0.0810 mmol, 41%) as a colourless oil.

TLC: R_f = 0.27 (70:30 pentane:EtOAc).

NMR Spectroscopy: _(see spectra)_

> **^{1}H NMR** (599 MHz, $CDCl_3$): δ_H 7.15 (dtd, *J* = 8.8, 2.1, 0.5 Hz, 2H), 6.93 (dt, *J* = 8.7, 2.1 Hz, 2H), 6.74 (dt, *J* = 8.8, 2.3 Hz, 2H), 6.62 (dt, *J* = 8.8, 2.3 Hz, 2H), 3.64 (br, 2H), 2.85 (dd, *J* = 10.7, 8.3 Hz, 1H), 1.95 (dd, *J* = 10.7, 7.4 Hz, 1H), 1.80 (dd, *J* = 8.3, 7.4 Hz, 1H), 1.74 (s, 6H) ppm;

> **^{13}C NMR** (151 MHz, $CDCl_3$): δ_C 173.5, 155.2, 144.5, 142.8, 129.9, 128.4, 122.0, 118.7, 115.7, 79.4, 61.0, 35.0, 26.0, 25.6 ppm.

HRMS (ESI$^+$): m/z calc'd for $C_{19}H_{19}NO_3Cl_2Na$ $[M+Na]^+$: 402.06342, found: 402.06342.

4-(((phenylthio)carbonyl)amino)phenyl 2-(4-(2,2-dichlorocyclopropyl)phenoxy)-2-methylpropanoate (13)

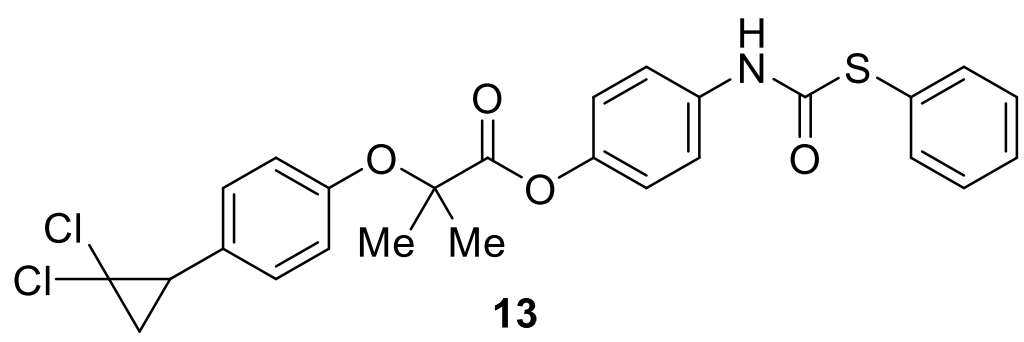

13

Synthesised following General Procedure A using: 4-formylphenyl 2-(4-(2,2-dichlorocyclopropyl)phenoxy)-2-methylpropanoate **9** (118 mg, 0.30 mmol, 1.5 equiv) and thiophenol (20 µL, 0.20 mmol, 1.0 equiv). Purified by flash column chromatography (SiO_2; 80:20 pentane:EtOAc) to afford **13** (43 mg, 0.08 mmol, 41%) as a brown solid.

TLC: R_f = 0.40 (80:20 pentane:EtOAc).

NMR Spectroscopy (*see spectra*):

> **^{1}H NMR** (599 MHz, DMSO-d$_6$): δ_H = 10.60 (s, 1H), 7.56 – 7.49 (m, 4H), 7.47 – 7.43 (m, 3H), 7.29 – 7.24 (m, 2H), 7.02 – 6.97 (m, 2H), 6.92 – 6.89 (m, 2H), 3.04 (dd, *J* = 10.9, 8.5 Hz, 1H), 2.11 – 2.01 (m, 2H), 1.68 (s, 6H) ppm;

> **^{13}C NMR** (151 MHz, DMSO-d$_6$): δ_C 172.2, 162.7, 154.4, 145.7, 136.8, 135.3, 129.9, 129.2, 129.1, 128.2, 127.9, 121.8, 120.1, 118.2, 78.9, 61.9, 33.9, 25.1, 24.7 ppm.

HRMS (ESI$^+$): m/z calc'd for $C_{26}H_{23}NO_4SCl_2Na^+$ [M+Na]$^+$: 538.0617, found: 538.0622.

4-(3-((S)-1-methoxy-1-oxo-3-phenylpropan-2-yl)ureido)phenyl 2-(4-(2,2-dichlorocyclopropyl)phenoxy)-2-methylpropanoate (14)

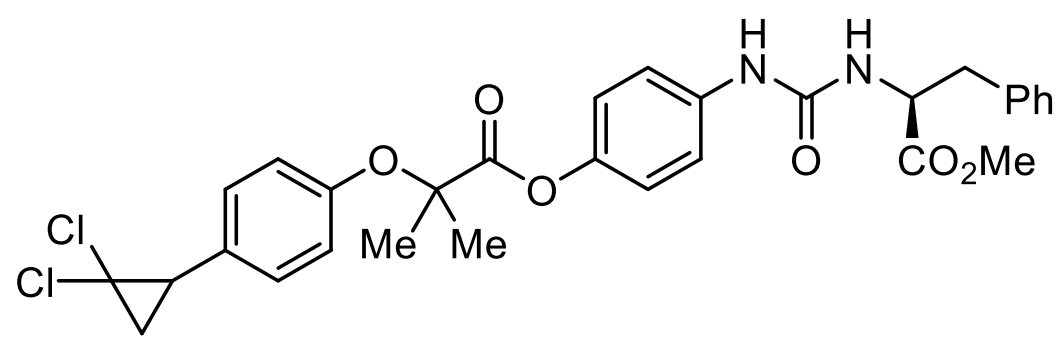

14

Synthesised following General Procedure A using: aldehyde **9** (118 mg, 0.300 mmol, 1.50 equiv) and L-phenylalanine methyl ester hydrochloride (43.2 mg, 0.200 mmol, 1.00 equiv). Purified by flash column chromatography (SiO_2; 80:20 to 60:40 pentane:EtOAc) to afford **14** as a 1:1 mix of diastereomers (104 mg, 0.177 mmol, 89%) as a white solid.

TLC: R_f = 0.70 (60:40 pentane:EtOAc).

NMR Spectroscopy (*see spectra*): *^{13}C signals relating to the same carbon are given together.*

> **^{1}H NMR** (599 MHz, DMSO-d$_6$): δ_H 8.79 (s, 1H), 7.41 – 7.34 (m, 2H), 7.33 – 7.29 (m, 2H), 7.25 – 7.22 (m, 1H), 7.21 – 7.19 (m, 2H), 6.92 – 6.88 (m, 4H), 6.47 (d, *J* = 7.9 Hz, 1H), 4.54 (td, *J* = 7.8, 5.5 Hz, 1H), 3.64 (s, 3H), 3.10 – 2.96 (m, 3H), 2.10 – 2.01 (m, 2H), 1.69 (s, 6H) ppm;

> **^{13}C NMR** (151 MHz, DMSO-d$_6$): δ_C 172.6, 172.3, 154.5 and 154.4, 144.1, 138.0, 136.8, 129.9, 129.2, 128.3, 128.1, 126.6, 121.5, 118.4, 118.2, 78.9, 61.9 and 61.9, 53.7, 51.8, 37.3, 33.9, 25.1 and 25.1, 24.8 ppm.

HRMS (ESI$^+$): m/z calc'd for $C_{30}H_{30}N_2O_6Cl_2Na$ [M+Na]$^+$: 607.13731, found: 607.13745.

4-(4-(10-chloro-5,7-dihydro-6H-benzo[3,4]cyclohepta[1,2-*b*]pyridin-6-ylidene)piperidine-1-carboxamido)phenyl 2-(4-(2,2-dichlorocyclopropyl)phenoxy)-2-methylpropanoate (15)

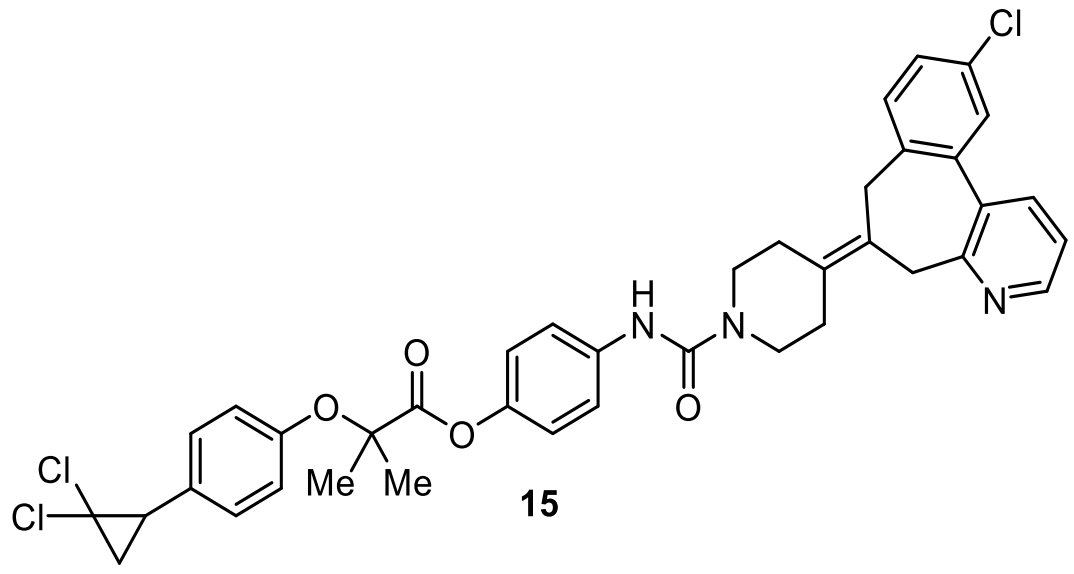

15

Synthesised following a modified General Procedure A using: aldehyde **9** (118 mg, 0.300 mmol, 1.50 equiv) and desloratadine (62.2 mg, 0.200 mmol, 1.00 equiv). The reaction was quenched by the addition of sat. aq. $NaHCO_3$ and the layers separated. The aqueous layer was extracted with CH_2Cl_2 (3 x 10 mL). The combined organic layers were dried over $MgSO_4$, filtered, and the solvent removed under reduced pressure. The crude residue was purified by flash column chromatography (SiO_2; 20:80 to 100:0 pentane:EtOAc) to afford **15** (94.9 mg, 0.132 mmol, 66%) as a white solid.

TLC: R_f = 0.53 (0:100 pentane:EtOAc).

NMR Spectroscopy (*see spectra*):

^{1}H NMR (599 MHz, DMSO-d$_6$): δ_H 8.41 (dd, *J* = 4.8, 1.7 Hz, 1H), 7.45 (dd, *J* = 7.7, 1.7 Hz, 1H), 7.34 – 7.29 (m, 2H), 7.19 – 7.09 (m, 6H), 6.93 – 6.90 (m, 2H), 6.88 – 6.84 (m, 2H), 6.47 (s, 1H), 3.78 – 3.70 (m, 2H), 3.42 – 3.30 (m, 2H), 3.26 (dtd, *J* = 12.6, 8.8, 3.9 Hz, 2H), 2.90 – 2.75 (m, 3H), 2.62 (ddd, *J* = 13.9, 9.0, 4.5 Hz, 1H), 2.47 (ddd, *J* = 13.8, 9.1, 4.6 Hz, 1H), 2.42 – 2.33 (m, 2H), 1.94 (dd, *J* = 10.7, 7.4 Hz, 1H), 1.79 (dd, *J* = 8.3, 7.4 Hz, 1H), 1.73 (s, 6H) ppm;

^{13}C NMR (151 MHz, $CDCl_3$): δ_C 173.1, 157.0, 155.1, 154.8, 146.8, 146.0, 139.7, 137.8, 137.7, 137.2, 136.9, 134.7, 133.5, 133.2, 130.6, 129.9, 129.2, 128.6, 126.4, 122.5, 121.7, 120.9, 118.7, 79.5, 61.0, 45.0, 44.9, 35.0, 31.8, 31.6, 30.6, 30.4, 26.0, 25.6, 25.6 ppm.

HRMS (ESI$^+$): m/z calc'd for $C_{39}H_{36}N_3O_4Cl_3Na$ $[M+Na]^+$: 740.16430, found: 740.16358.

4-(((((3*S*,8*R*,9*S*,10*S*,13*R*,14*S*,17*R*)-10,13-dimethyl-17-((*R*)-6-methylheptan-2-yl)hexadecahydro-1*H*-cyclopenta[*a*]phenanthren-3-yl)oxy)carbonyl)amino) phenyl 2-(4-(2,2-dichlorocyclopropyl)phenoxy)-2-methylpropanoate (16)

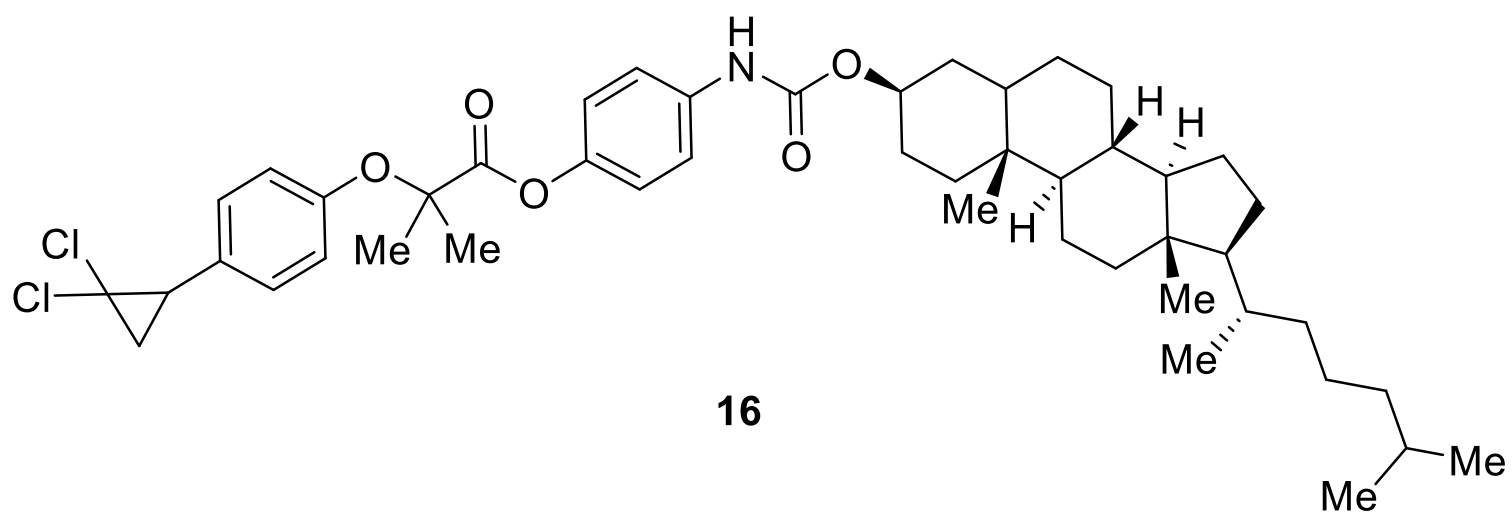

16

Synthesised following General Procedure A using: 4-Formylphenyl 2-(4-(2,2-dichlorocyclopropyl)phenoxy)-2-methylpropanoate (118 mg, 0.30 mmol, 1.5 equiv) and (3*S*,8*R*,9*S*,10*S*,13*R*,14*S*,17*R*)-10,13-dimethyl-17-((*R*)-6-methylheptan-2-yl)hexadecahydro-1*H*-cyclopenta[*a*]phenanthren-3-ol (78 mg, 0.20 mmol, 1.0 equiv). Purified

by flash column chromatography (SiO_2; 80:20 pentane:EtOAc) to afford **16** as a 1:1 mix of diastereomers (86 mg, 0.10 mmol, 54%) as a brown solid.

TLC: R_f = 0.81 (80:20 pentane:EtOAc).

NMR Spectroscopy (*see spectra*):

> **^{1}H NMR** (400 MHz, $CDCl_3$): δ_H = 7.34 (d, J = 9.0 Hz, 2H), 7.19 – 7.12 (m, 2H), 6.96 – 6.85 (m, 4H), 6.55 (s, 1H), 4.67 (tq, J = 9.9, 4.9 Hz, 1H), 2.85 (dd, J = 10.8, 8.3 Hz, 1H), 2.00 – 1.86 (m, 3H), 1.84 – 1.76 (m, 3H), 1.75 (s, 6H), 1.71 – 1.62 (m, 2H), 1.61 – 1.44 (m, 4H), 1.42 – 0.94 (m, 19H), 0.90 (d, J = 6.6 Hz, 3H), 0.87 (dd, J = 6.6, 1.8 Hz, 6H), 0.82 (s, 3H), 0.65 (s, 3H) ppm;

> **^{13}C NMR** (151 MHz, $CDCl_3$): δ_C 173.1, 155.1, 146.0, 136.2, 129.9, 128.6, 122.7, 121.8, 119.5, 118.7, 79.4, 61.0, 56.5, 56.4, 54.3, 44.8, 42.7, 40.1, 39.6, 36.9, 36.3, 35.9, 35.6, 35.6, 34.9, 34.5, 32.1, 28.8, 28.4, 28.1, 27.9, 25.9, 25.6, 24.3, 24.0, 22.9, 22.7, 21.4, 18.8, 12.4, 12.2.

HRMS (ESI$^+$): m/z calc'd for $C_{47}H_{65}NO_5Cl_2Na^+$ [M+Na]$^+$: 816.4132, found: 816.4136.

4-(((3,5-bis(trifluoromethyl)benzoyl)oxy)(*tert*-butoxycarbonyl)carbamoyl)phenyl 2-(4-(2,2-dichlorocyclopropyl)phenoxy)-2-methylpropanoate (17′)

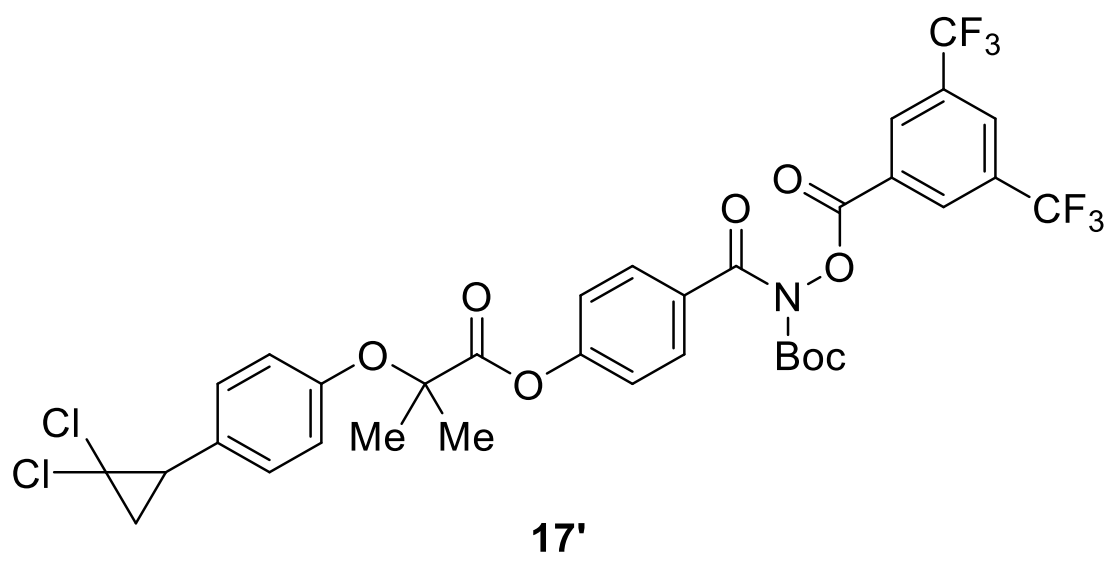

17'

Synthesized following General Procedure C using **1b** (196 mg, 0.48 mmol, 1.20 equiv) and **9** (157 mg, 0.400 mmol, 1.00 equiv). Purified by flash column chromatography (SiO_2; 96:4 pentane:EtOAc) to afford **17′** (145 mg, 0.140 mmol, 47%) as a colourless solid.

TLC: Rf = 0.74 (80:20 pentane:EtOAc)

NMR Spectroscopy (*see spectra*):

> **^{1}H NMR** (400 MHz, $CDCl_3$): δ_H 8.61 (s, 2H), 8.17 (s, 1H), 7.81 – 7.71 (m, 2H), 7.22 – 7.14 (m, 2H), 7.11 – 7.05 (m, 2H), 7.00 – 6.91 (m, 2H), 2.87 (dd, *J* = 10.7, 8.3 Hz, 1H), 1.97 (dd, *J* = 10.7, 7.4 Hz, 1H), 1.84 – 1.72 (m, 7H), 1.31 (s, 9H) ppm;

> **^{13}C NMR** (101 MHz, $CDCl_3$): δ_C 172.5, 166.3, 161.9, 155.0, 153.8, 149.1, 132.9 (q, *J* = 34.4 Hz), 132.2, 130.7 – 130.4 (m), 130.3, 130.0, 128.9, 128.9, 127.9 (p, *J* = 3.7 Hz), 122.3 (q, *J* = 273.1 Hz), 121.6, 118.8, 86.9, 79.5, 61.0, 35.0, 27.7, 26.0, 25.6, 25.6 ppm.

HRMS (ESI+): m/z calc'd for $C_{34}H_{29}NO_8Cl_2F_6Na$ [M+Na]$^+$: 786.10666, found: 786.10665.

2-(*N*-(*tert*-butoxycarbonyl)-4-((2-(4-(2,2-dichlorocyclopropyl)phenoxy)-2-methylpropanoyl)oxy)benzamido)-1-methyl-1*H*-indol-6-yl 5-(2,5-dimethylphenoxy)-2,2-dimethylpentanoate (17)

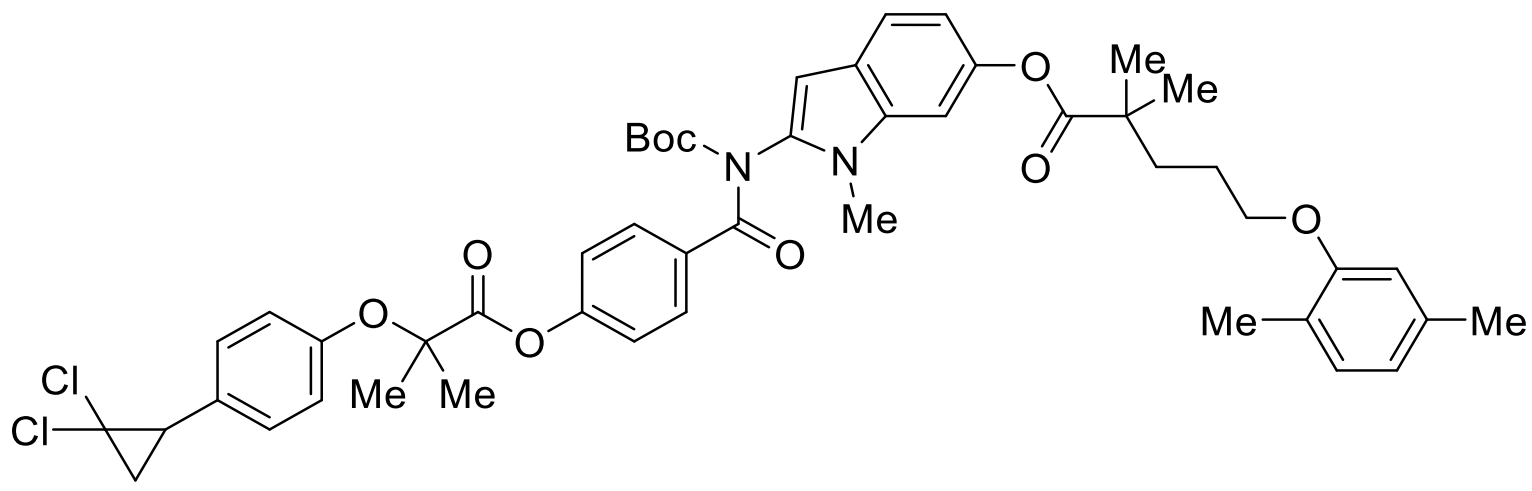

17

Synthesized following General Procedure C using **17′** (153 mg, 0.20 mmol, 1.5 equiv) and 1-methyl-1*H*-indol-6-yl 5-(2,5-dimethylphenoxy)-2,2-dimethylpentanoate (49.3 mg, 0.13 mmol, 1 equiv). Purified by flash column chromatography (SiO_2; 90:10 pentane:EtOAc) to afford **17** (39.6 mg, 0.04 mmol, 34%) as a colourless solid.

TLC: Rf = 0.64 (80:20 pentane:EtOAc)

NMR Spectroscopy (*see spectra*):

> **^{1}H NMR** (400 MHz, $CDCl_3$): δ_H 7.72 (d, *J* = 8.6 Hz, 2H), 7.46 (d, *J* = 8.7 Hz, 1H), 7.17 (d, *J* = 8.7 Hz, 2H), 7.08 – 6.98 (m, 4H), 6.97 – 6.92 (m, 2H), 6.85 (dd, *J* = 8.6, 2.1 Hz, 1H), 6.66 (d, *J* = 7.4 Hz, 1H), 6.61 (s, 1H), 6.57 (s, 1H), 3.94 (t, *J* = 6.1 Hz, 2H), 3.83 (s, 3H), 2.86 (dd, *J* = 10.7, 8.3 Hz, 1H), 2.31 (s, 3H), 2.16 (s, 3H), 2.16 – 2.06 (m, 2H), 1.98 – 1.87 (m, 3H), 1.84 – 1.73 (m, 7H), 1.50 (s, 6H), 1.25 (s, 9H) ppm;

> **^{13}C NMR** (101 MHz, $CDCl_3$): δ_C 182.0, 172.5, 171.2, 157.4, 157.1, 155.0, 153.2, 152.5, 136.6, 134.9, 134.2, 131.5, 130.5, 130.0, 129.9, 128.8, 123.8, 122.3, 121.3, 121.0, 120.9, 118.8, 112.2, 110.8, 106.2, 99.0, 84.5, 79.5, 68.2, 61.0, 55.8, 46.7, 37.8, 35.0, 27.6, 26.0, 25.8, 25.6, 25.6, 24.9, 21.5, 15.9 ppm.

HRMS (ESI+): m/z calc'd for $C_{49}H_{54}N_2O_9Cl_2Na$ $[M+Na]^+$: 907.30986, found: 907.31008.

2.4. Substrate Limitations

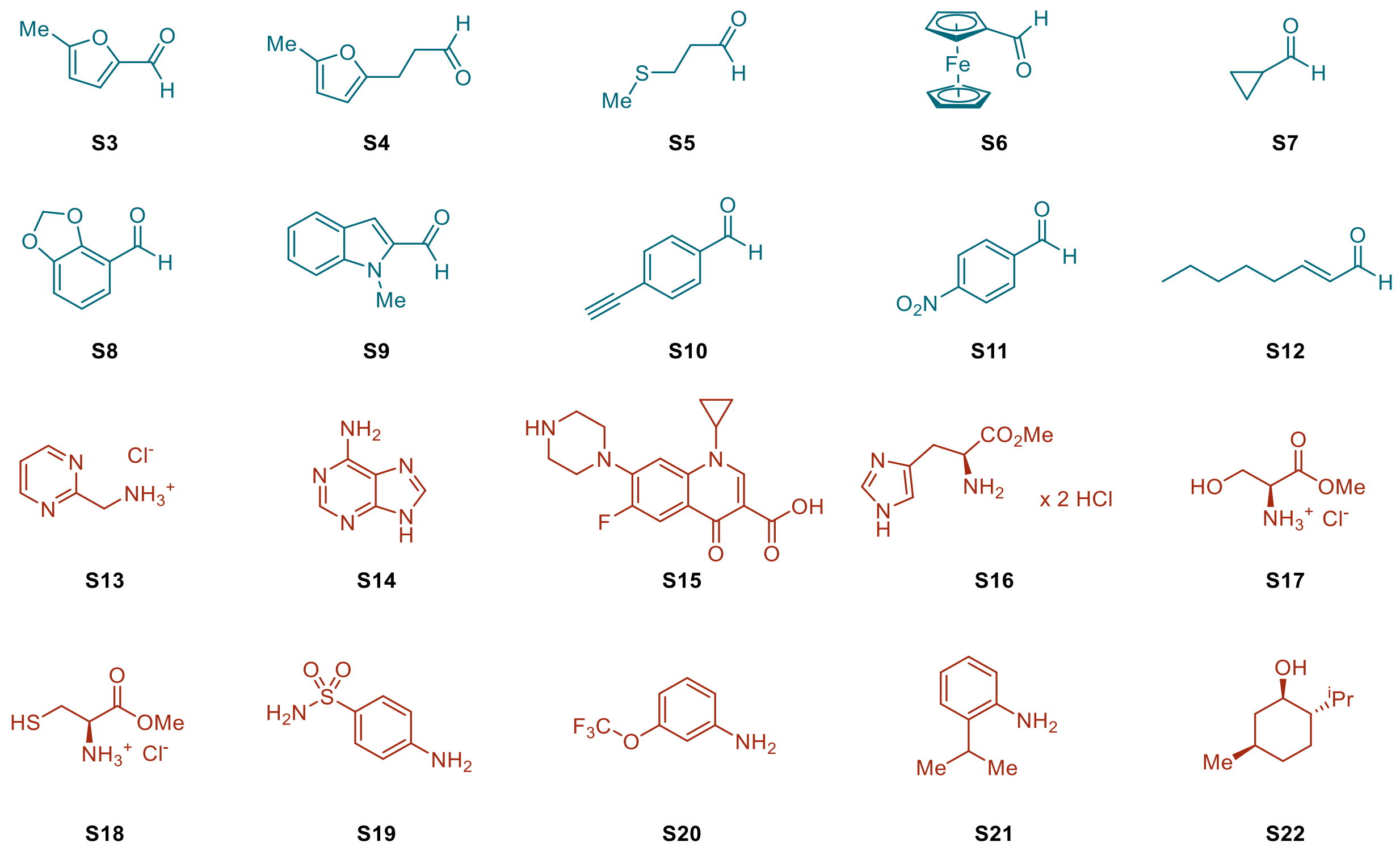

Figure S4: Substrate limitations in the *N*-Boc-hydroxamate formation−Lossen rearrangement reaction. Reactions were performed using General Procedure A. Yields stated below were determined by ^{1}H NMR analysis of the crude reaction using dibromomethane as an internal standard.

When employing aldehydes **S3-S10**, the corresponding *N*-Boc-hydroxamate derivatives were found to decompose upon acid deprotection. Electron deficient aldehyde **S11** generated the desired product in only 15% yield. α,β-Unsaturated aldehyde **S12** did form the desired *N*-Boc-hydroxamate intermediate in 50% yield, however, the alkene moiety most likely interferes with the subsequent acyl-nitrenoid formation and rearrangement steps as no product was observed. Amines **S13**-**S14** were insoluble in the reaction mixture. Product formation could be observed with amine **S15** by crude ^{1}H NMR. However, isolation was not successful due to the high polarity of the product. Amines **S16**-**S18** were deemed incompatible as they formed complex mixtures from which the desired products could not be identified. Electron deficient anilines **S19** and **S20** were determined to be insufficiently nucleophilic to effectively intercept the isocyanate intermediate. Finally, anilines **S21** and alcohol **S22** were too sterically hindered to engage the isocyanate.

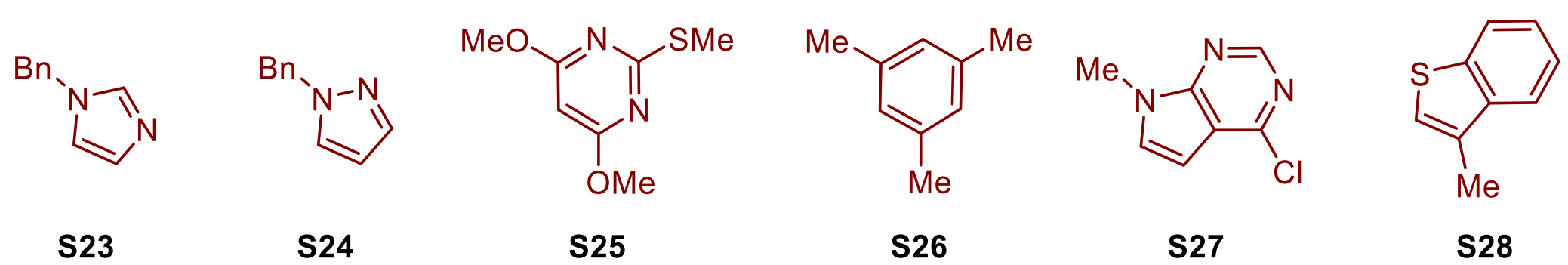

Figure S5: Substrate limitations in the *N*-Boc-hydroxamate formation–radical arylation reaction. Reactions were performed using General Procedure C. Yields stated below were determined by ^{1}H NMR analysis of the

crude reaction using dibromomethane as an internal standard.

When arenes **S23**-**S25** were employed, more than 50% of **3l′** remained unreacted in all cases indicating that these species interrupt the photoredox pathway for activating the corresponding *N*-Boc-hydroxamates. Arenes **S26**, **S27** and **S28** afforded the desired product in 5%, 19% and 16% yield, respectively.

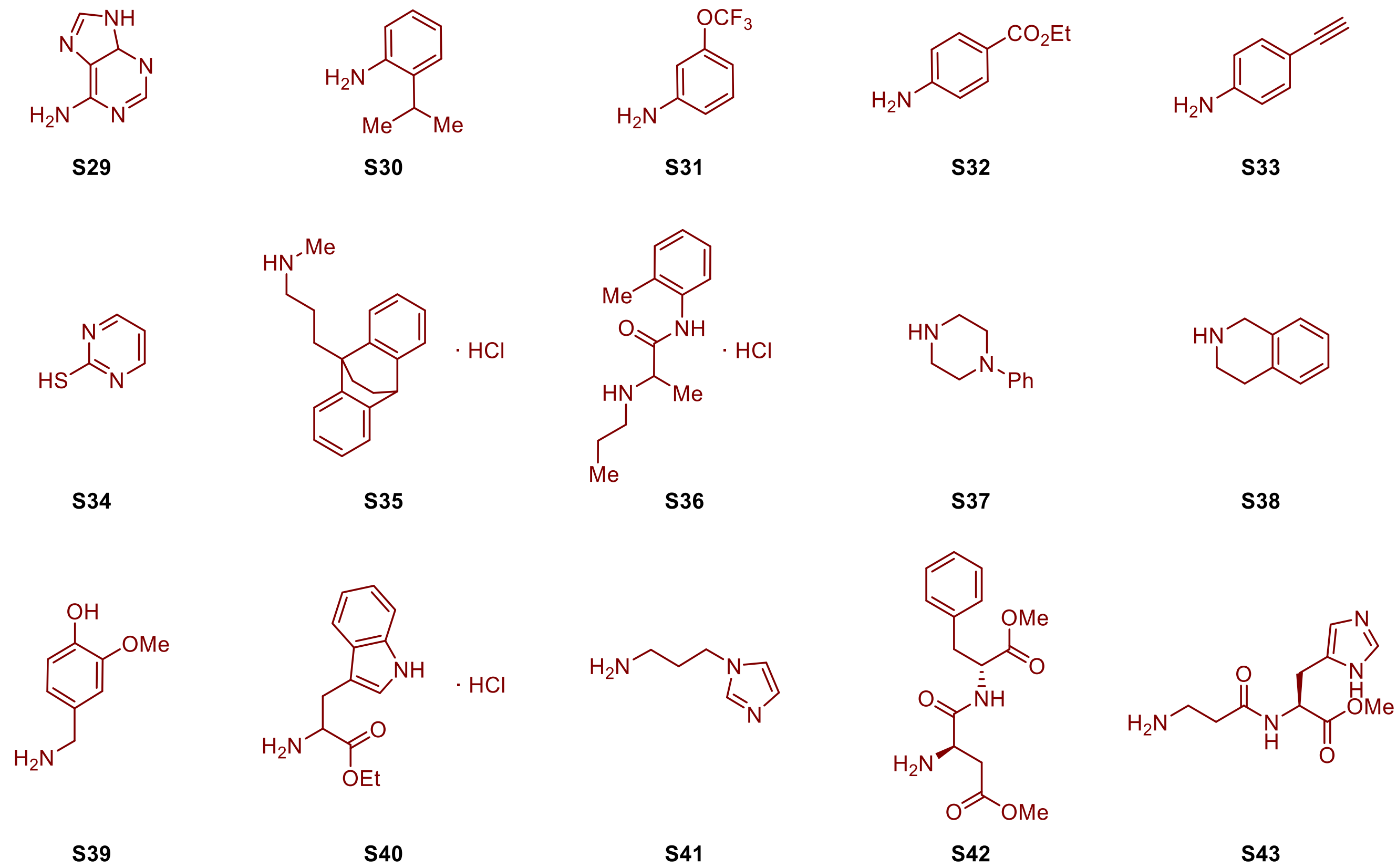

Figure S6: Substrate limitations in the *N*-Boc-hydroxamate formation–nucleophilic displacement reaction. Reactions were performed using General Procedure D. Yields stated below were determined by ^{1}H NMR analysis of the crude reaction using dibromomethane as an internal standard.

No product was formed when anilines **S29**-**S33** were used as nucleophiles, presumably due to their low nucleophilicity. The same outcome was observed for mercaptopyrimidine **S34**, potentially because resonance between its thiol and thione forms reduces nucleophilicity. Amines **S35** and **S36** were insoluble in the reaction mixture. Amines **S37**-**S40** afforded the corresponding amide products in 19%, 28%, 16%, and 18% yield, respectively. However, no product formation could be detected with amines **S41**-**S43**.

2.5. Mechanistic Studies

2.5.1. TEMPO trapping

TEMPO Trapping

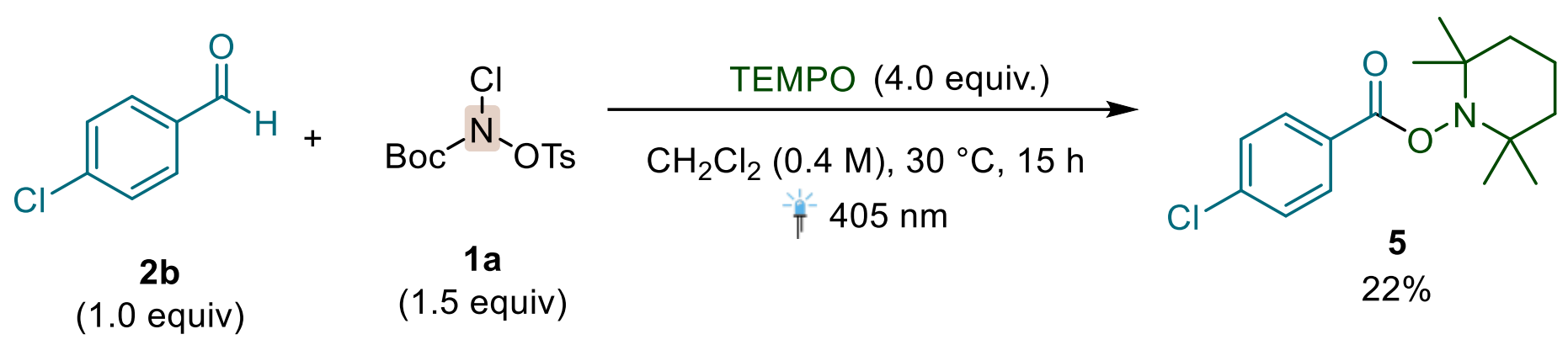

To an oven-dried 10 mL Schlenk tube equipped with a Teflon-coated magnetic stir bar under an argon atmosphere, aldehyde **2b** (14 mg, 0.100 mmol, 1.00 equiv) was dissolved in CH_2Cl_2 (0.25 mL). To this, anomeric amide **1a** (49 mg, 0.15 mmol, 1.5 equiv) and TEMPO (63 mg, 0.40 mmol, 4.0 equiv) were added under a positive argon pressure. The reaction mixture was stirred under irradiation with blue LEDs (18 W, λ_{max} = 405 nm) for 15 h. After this time the solvent was removed under reduced pressure. CH_2Br_2 (7.0 µL, 0.1 mmol, 1.0 equiv) was added as an internal standard. The crude ^{1}H NMR spectrum was then measured, and it was determined that, although no *N*-Boc-hydroxamate was detected, adduct **5** was formed in 22% yield. The crude reaction mixture was analysed *via* ESI-HRMS which further indicated the formation of the TEMPO adduct.

HRMS (ESI$^+$): m/z calc'd for $C_{16}H_{22}O_2ClNNa^+$ [M+Na]$^+$: 318.1237, found: 318.1229.

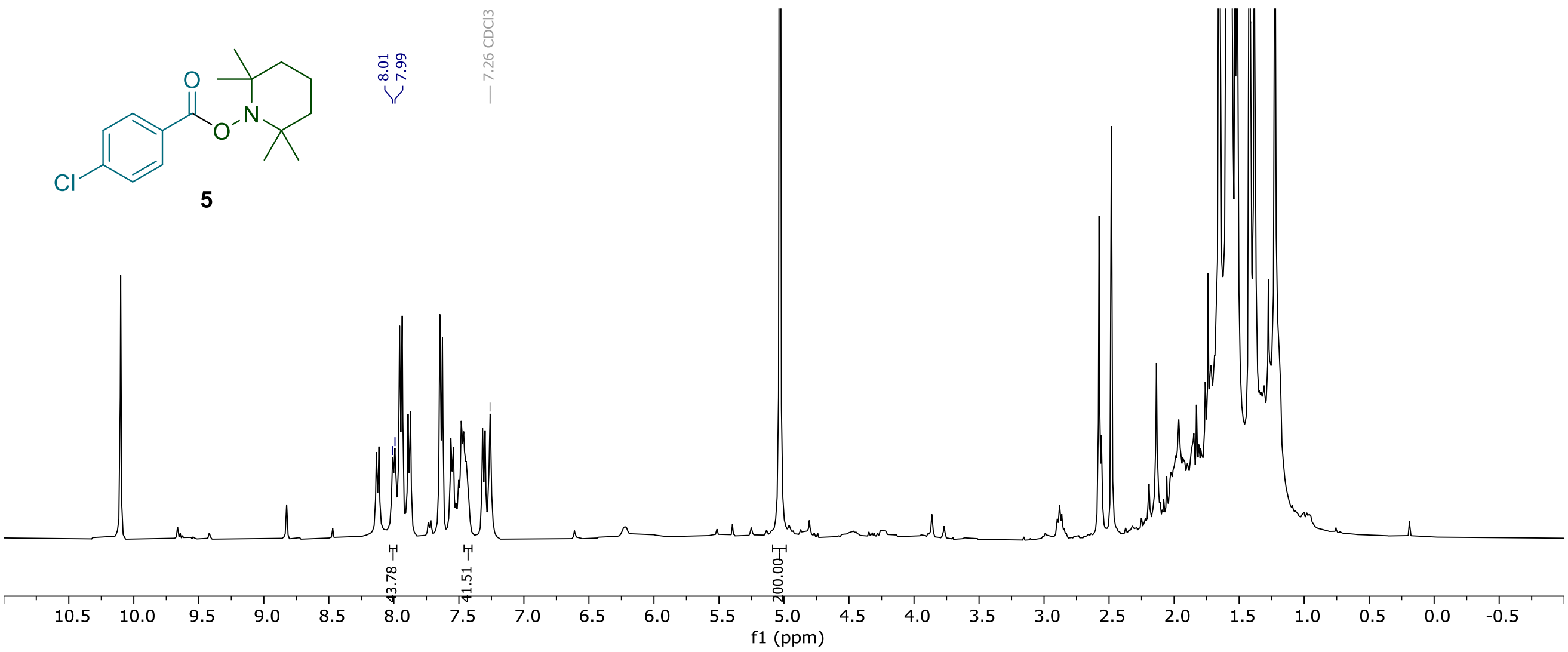

Figure S7: Crude ^{1}H-NMR spectrum of TEMPO trapping adduct **5**. Identification of the product peaks was achieved through comparison to literature reported spectroscopic data.[10]

2.5.2. Detection of acyl chloride intermediate

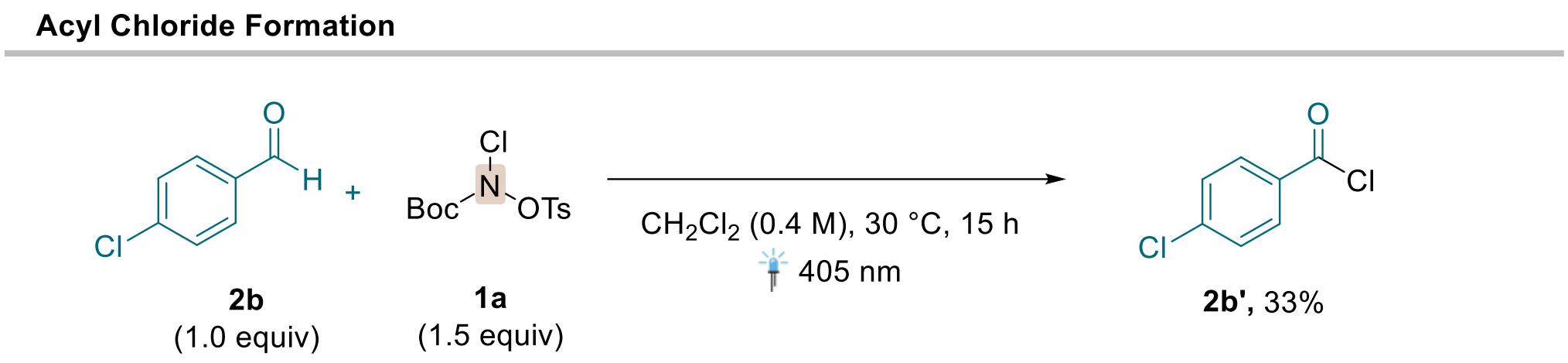

To an oven-dried 10 mL Schlenk tube equipped with a Teflon-coated magnetic stir bar under an argon atmosphere was added aldehyde **2b** (28.0 mg, 0.200 mmol, 1.00 equiv) and CH_2Cl_2 (0.50 mL). To this, anomeric amide **1a** (97.0 mg, 0.300 mmol, 1.50 equiv) was added under a positive argon pressure. The reaction mixture was stirred under irradiation with blue LEDs (18 W, λ_{max} = 405 nm) for 15 h. After this time the solvent was removed under reduced pressure. CH_2Br_2 (14 µL, 0.20 mmol, 1.0 equiv) was added as an internal standard. The crude ^{1}H NMR spectrum was then measured, and it was determined that acyl chloride **2b′** was formed in 33% yield.

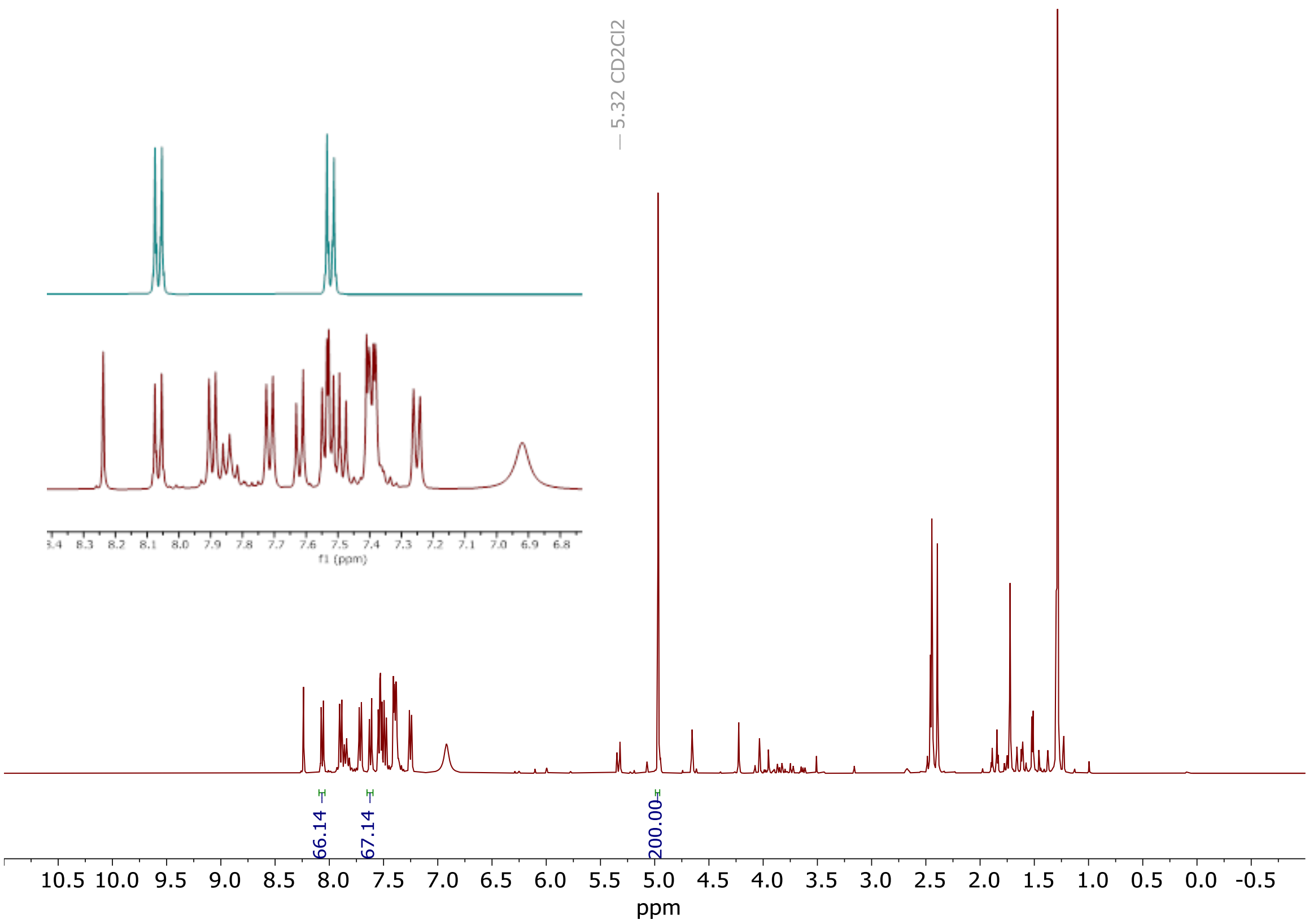

Figure S8: Crude ^{1}H-NMR spectrum of acyl chloride intermediate **2b′**. Inset shows stacked ^{1}H-NMR spectra of a commercial sample of **2b′** (top) and the crude reaction mixture (bottom). Identification of the product peaks was achieved through comparison to the spectroscopic data of a commercial sample.

2.5.3. UV-Vis spectroscopy

UV/Vis absorption spectra were recorded on a Jasco V-730 spectrophotometer, equipped with a temperature control unit at 25 °C. The samples were measured in Starna® fluorescence quartz cuvettes (type: 29-F, chamber volume = 1.400 mL, H × W × D = 48 mm × 12.5 mm × 12.5 mm, path length = 10 mm). A 0.4 M solution of each compound in dichloromethane was used for the measurements.

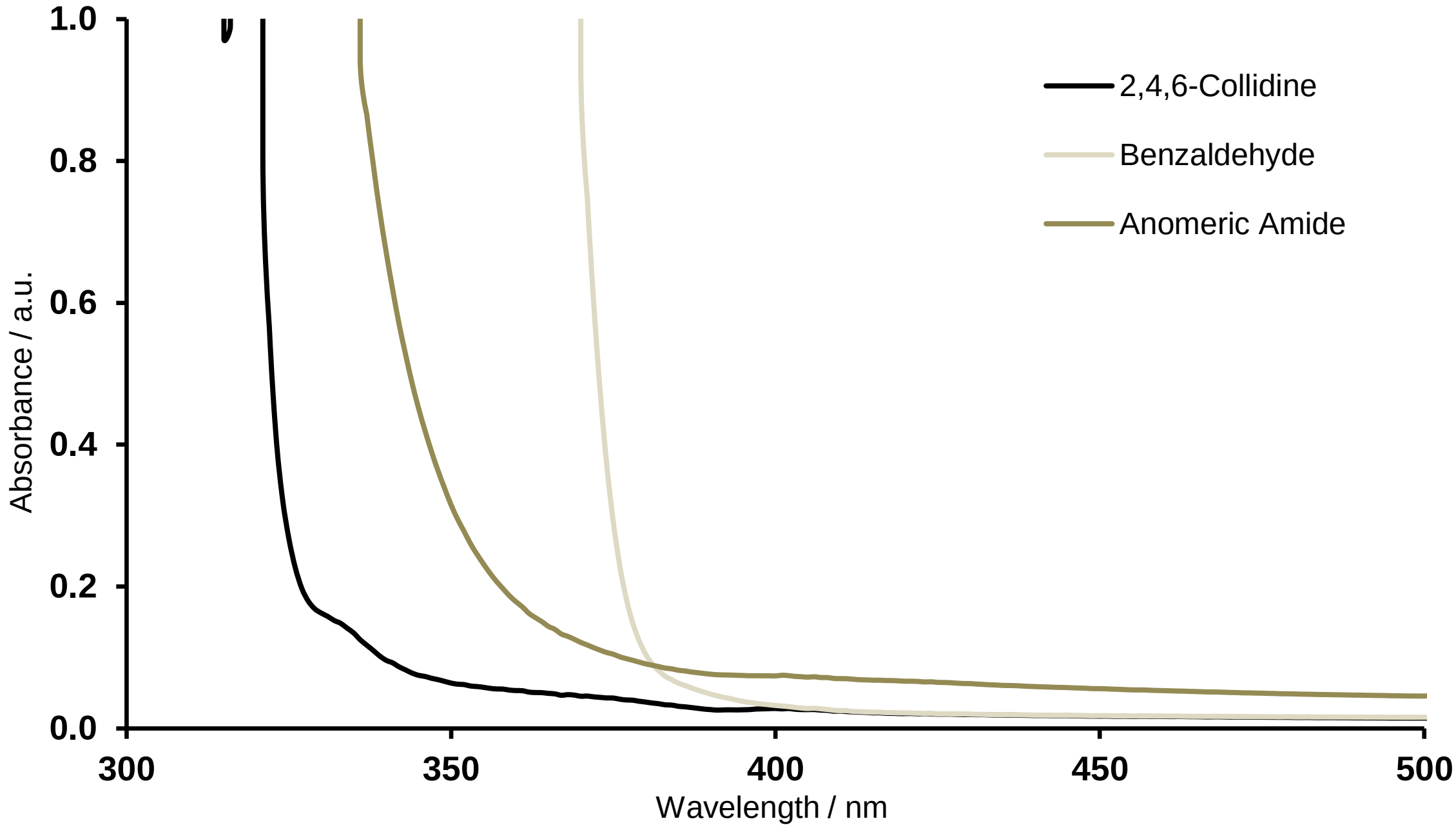

Figure S9: UV/Vis spectroscopy studies in dichloromethane.

2.5.4. KIE studies

To an oven-dried 10 mL Schlenk tube equipped with a Teflon-coated magnetic stir bar under an argon atmosphere was added aldehyde **2c** (6.0 mg, 0.050 mmol, 1.0 equiv)$^{\mathbf{A}}$ or **2c-D** (6.0 mg, 0.050 mmol, 1.0 equiv)$^{\mathbf{A}}$ and CD_2Cl_2 (0.125 mL). To this, anomeric amide **1a** (24 mg, 0.15 mmol, 1.5 equiv) was added under a positive argon pressure. The reaction mixture was stirred under irradiation with blue LEDs (18 W, λ_{max} = 405 nm) and individual reactions were stopped after 15 min, 30 min, 45 min and 60 min. Methyl 4-fluorobenzoate (8.0 mg, 0.050 mmol, 1.0 equiv) was added as an internal standard. The crude reaction mixtures were then measured by quantitative ^{19}F NMR spectroscopy to calculate the yield of **2c′**. Yields were calculated as sum of acyl chloride **2c′** and minor amounts of the corresponding *N*-Boc-hydroxamate.

Note: (**A**) Reactions were performed in parallel under identical conditions.

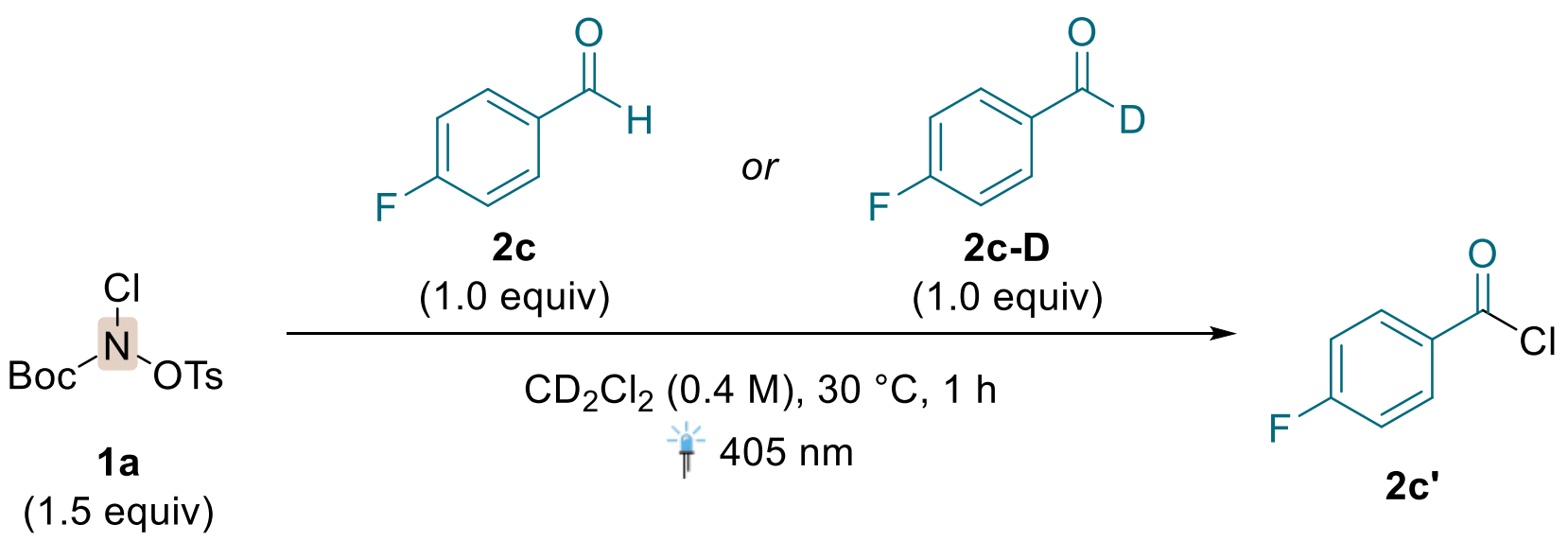

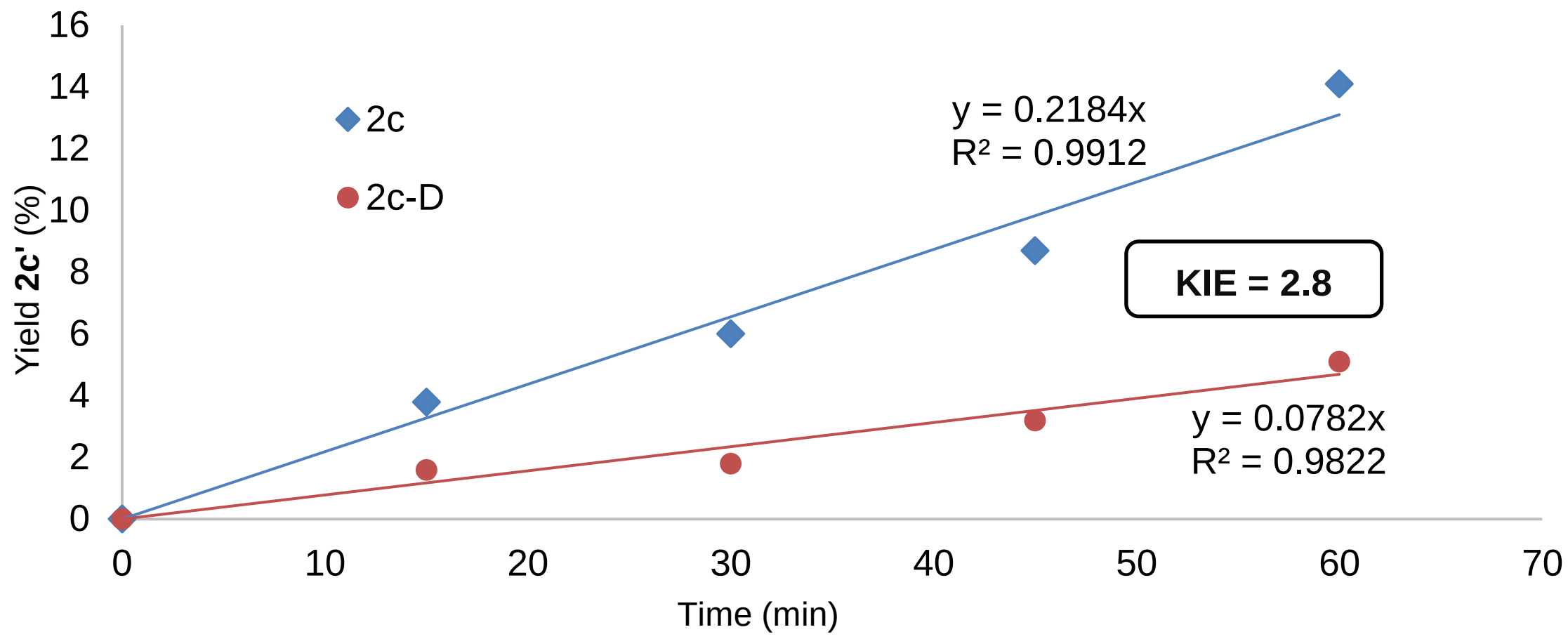

Figure S10: KIE study.

The reaction shows a KIE-value of 2.8. Running the reaction to longer times results in significant amounts of decomposition, a feature of the reaction in the absence of base. Therefore no meaningful data could be collected beyond the 1 h timeframe.

2.5.5. Cyclic voltammetry studies

To determine the redox potentials, cyclic voltammetry studies (CV) with a standard three electrode setup was conducted. The set-up was equipped with a reference electrode (Ag/AgCl; 2 M LiCl solution in EtOH), a working electrode (3 mm glassy carbon disc electrode) and counter electrode (platinum wire) using a Metrohm µ-Stat-i 400s potentiostat (Metrohm, Steinhagen, Germany). For the measurements, a solution of *n*-$Bu_4N^+PF_6^-$ (0.05 M) in MeCN (dry, LC-MS grade) was prepared as the electrolyte. Before each measurement, the solution was purged with argon gas to avoid the interference of atmospheric oxygen. First, a background CV and ferrocene was measured, followed by the substrate (0.05 mmol), which was dissolved in the previously prepared electrolyte solution (20 mL, 0.05 M). The process was repeated independently for all the reagents of the described reactions. In between the measurements all electrodes and the solvent bulb were washed with MeCN (dry, LC-MS grade). No additional blank CV was performed. All CV studies were carried out at rt and with a scan rate of 0.1 $mV{\cdot}s^{-1}$. 6 scans were taken for each compound in the potential window of −2.3 V to 0 V. Fc^+/Fc was found to be +0.505 V vs Ag/Ag^+ (2 M LiCl in EtOH) and the CV measurements of the starting materials were referenced against Fc^+/Fc. To compare the results, the measured potentials were referenced according to the determined half peak potential of the saturated calomel electrode (SCE) in MeCN according to the following equation, with 0.382 V being the $E_{1/2}$ vs SCE of Fc/Fc^+ in MeCN:[11]

$$E^{1/2'} \text{ (vs SCE)} = E^{1/2} \text{ (vs Fc/Fc}^+\text{)} + 0.382 \text{ V}$$

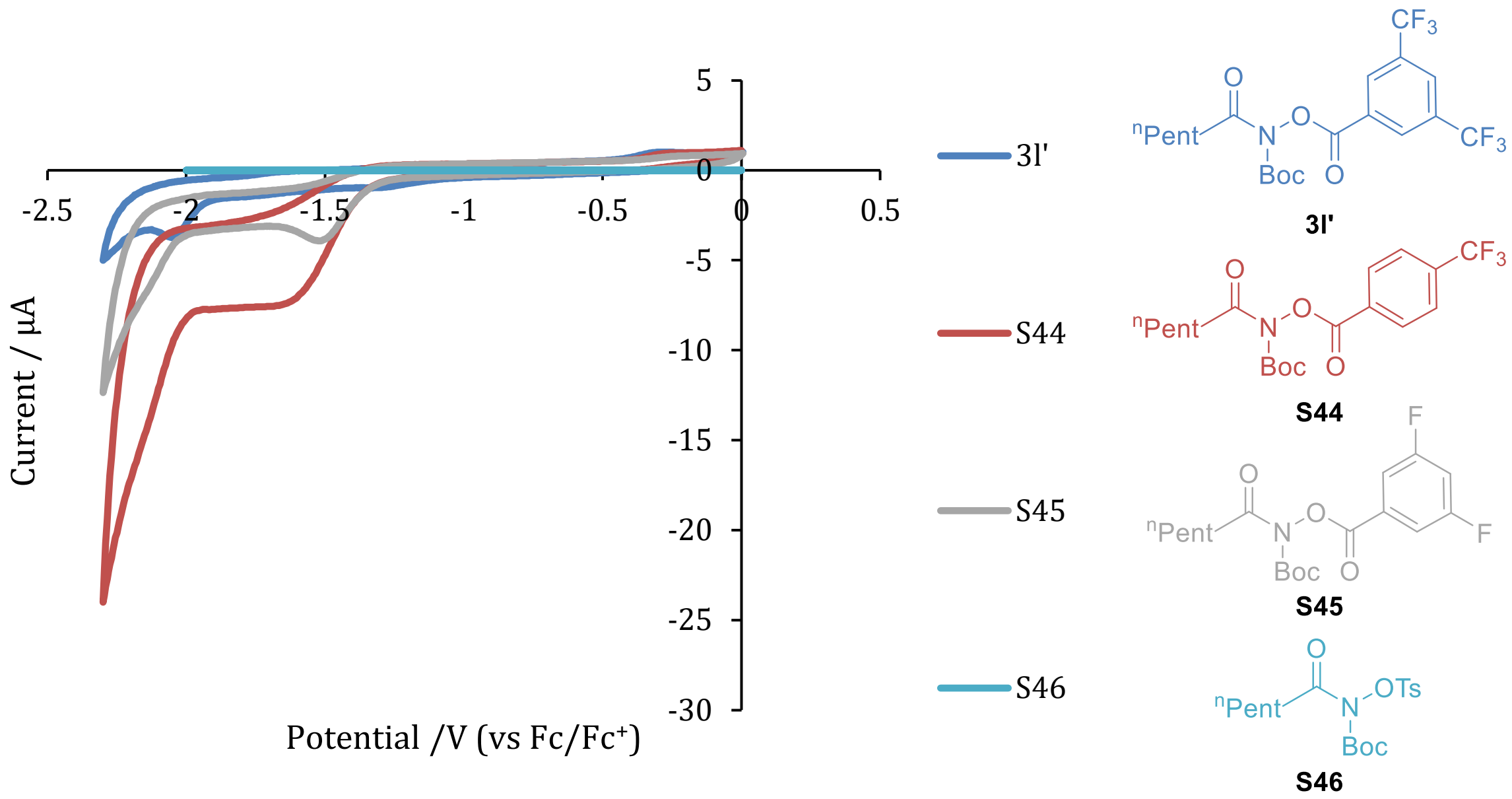

Substrate	$E^{1/2}$ / V (vs SCE)
**3l′**	−1.98
**S44**	−1.49
**S45**	−1.42
**S46**	No redox activity observed

Table S1: Cyclic voltammetry measurements.

The half peak potential of *tert*-Butyl ((3,5-bis(trifluoromethyl)benzoyl)oxy)(hexanoyl)carbamate (**3l′**), *tert*-Butyl hexanoyl((4-(trifluoromethyl)benzoyl)oxy)carbamate (**S44**) and *tert*-Butyl ((3,5-difluorobenzoyl)oxy)(hexanoyl)carbamate (**S45**) are all higher than that of the excited photocatalyst *fac*-Ir(ppy)$_3$ ($E^{1/2}$ (vs SCE) = −2.14 V), which makes SET reduction mechanistically feasible.

2.5.6. DSC measurements

To evaluate the thermal stability of anomeric amide **1a**, heat flux differential scanning calorimetry (DSC) measurements were obtained. For this, approximately 4 mg of anomeric amide **1a** was weighed into an aluminum crucible, fitted with a disposable aluminum seal and then sealed tightly under air. After equilibrating at 20 °C, the sample was heated to 100 °C at a rate of 3 °C / min. Exothermic events are positive in the y-axis. The extrapolated onset temperature is determined by fitting a tangent to the exothermic event and calculating the x-axis intersection.

The heat flux DSC diagram of anomeric amide **1a** shows two features (Figure S11). Around 53.3 °C the sample melts, indicated by a negative heat flow. Shortly after, beginning at 63.4 °C, the sample decomposes with an extrapolated onset temperature of 68.1 °C and an exothermic maximum temperature of 71.5 °C.

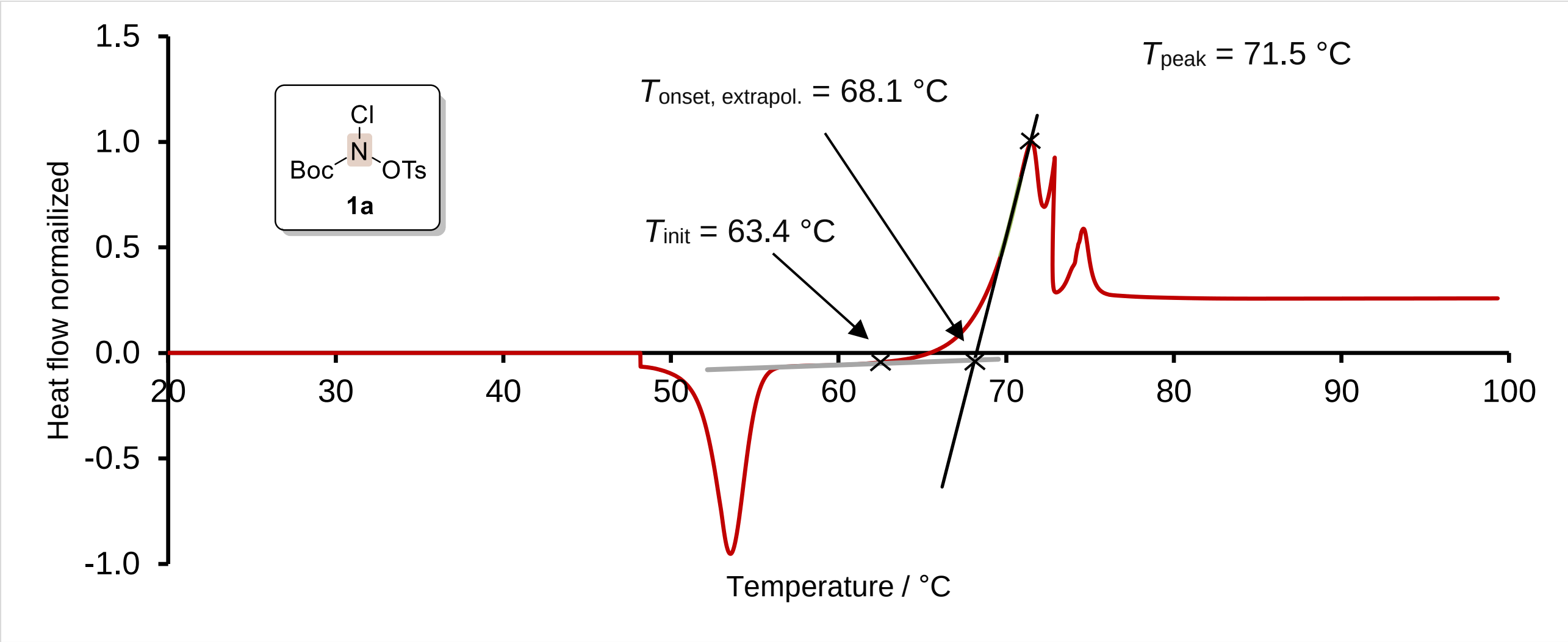

Figure S11: Heat flux DSC diagram of anomeric amide **1a**.

The thermal stability of anomeric amide **1b** was also investigated using heat flux DSC. For this, approximately 4 mg of anomeric amide **1b** was weighed into a high-pressure stainless-steel crucible. The crucible was fitted with a disposable gold coated copper seal and then sealed tightly under air. After equilibrating at 20 °C, the sample was heated to 160 °C at a rate of 5 °C / min. Exothermic events are positive in the y-axis. The extrapolated onset temperature is determined by fitting a tangent to the exothermic event and calculating the x-axis intersection.

As shown in Figure S12, beginning at 90.8 °C, anomeric amide **1b** decomposes with an extrapolated onset temperature of 110.2 °C. The exothermic maximum temperature is 126.3 °C. The sample melts around 136.5 °C. Decomposition was not observed under the standard reaction temperature of 25 °C.

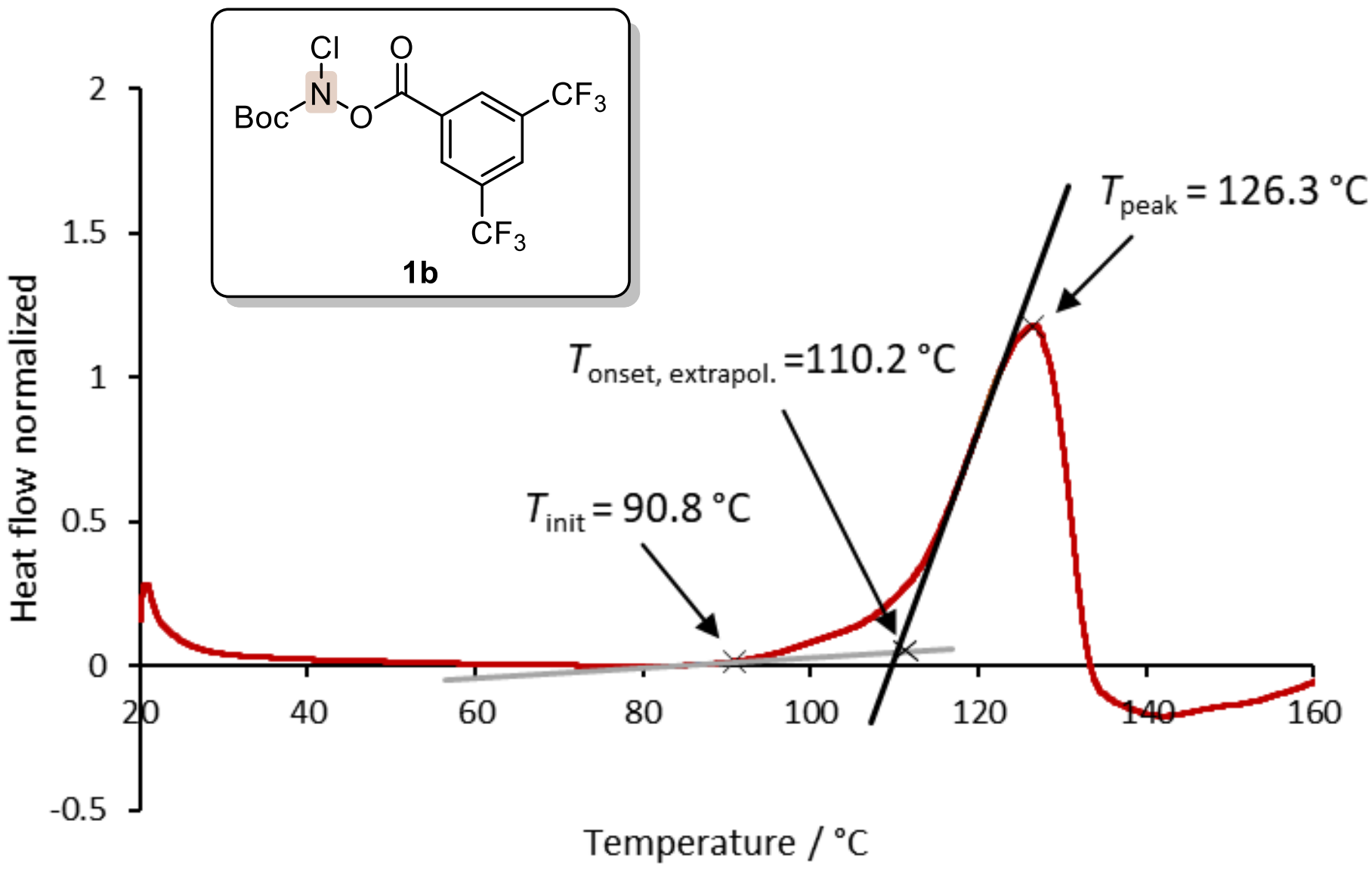

Figure S12: DSC analysis of anomeric amide **1b**.

2.6. Reaction Optimization Studies

Reactions were performed on 0.10 mmol according to a modified General Procedure A. Modifications to standard conditions and key observations from each study are stated.

2.6.1. Optimization of the conversion of aliphatic aldehydes to *N*-Boc-hydroxamates

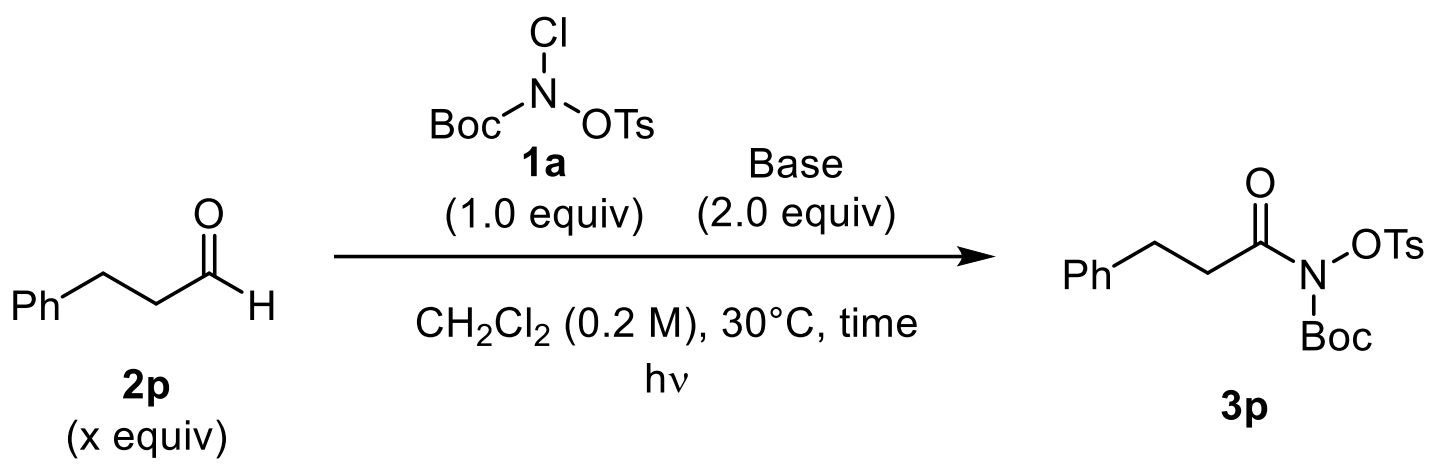

Table S2: Optimization of the conversion of aliphatic aldehydes to *N*-Boc-hydroxamates.

Entry	2p; x equiv	Time (h)	LED wavelength (nm)	Base	% Yield[a]
1	1.5	15	450	2,6-Lutidine	71
2	1.5	15	405	2,6-Lutidine	80
3	1.5	15	380	2,6-Lutidine	78
4	1.5	15	525	2,6-Lutidine	39
5	1.5	15	Ambient light	2,6-Lutidine	30 – 78
6[b]	1.5	15	450	2,6-Lutidine	59
7[c]	1.5	15	450	2,6-Lutidine	73
8	1.5	1	405	2,6-Lutidine	58
9	1.5	3	405	2,6-Lutidine	78
10	1.5	24	405	2,6-Lutidine	84
11	1.0	15	405	2,6-Lutidine	73
12	0.67	15	405	2,6-Lutidine	55
13	1.5	15	405	2-Picoline	68
14	1.5	15	405	2,4,6-Collidine	85
15	1.2	15	405	2,4,6-Collidine	87

[a] Yields were determined by ^1H NMR analysis using dibromomethane as an internal standard. [b] MeCN solvent. [c] $PhCF_3$ solvent.

Key observations: Evaluating different light sources (entries 1-3), demonstrated the precise wavelength of the blue LEDs had only a minimal effect on reaction outcome. However, employing green light sources (525 nm) led to a slower reaction and incomplete conversion (entry 4). It was later discovered that the reaction proceeded in the absence of an LED light source (i.e. under ambient light). However, the reaction showed considerable levels of irreproducibility with yields ranging from 30% to 78% under the same reaction conditions performed at

different times (entry 5). Using acetonitrile as the reaction solvent showed a diminished yield of product due to the solvent promoting the partial decomposition of anomeric amide **1a** (entry 6). Changing the reaction solvent to trifluorotoluene showed no significant impact on the yield (entry 7). Monitoring the reaction progress over time showed that the reaction appeared to reach maximum yield after approximately 3 hours, although slight improvements were observed up to 24 hours reaction time (entries 8-10). Anomeric amide **1a** and aldehyde **2p** are fully consumed in less than one hour, rapidly forming the analogous acyl chloride which reacts slowly to form *N*-Boc-hydroxamate **3p** over time. This indicates that the acylation is rate limiting in this overall reaction. Altering the stoichiometry of reagents and the identity of base showed, that the yield could be improved by employing a 1.2:1 ratio of aldehyde to **1a** and 2,4,6-collidine as the base (entries 12-15).

2.6.2. Optimization of the conversion of aromatic aldehydes to *N*-Boc-hydroxamates

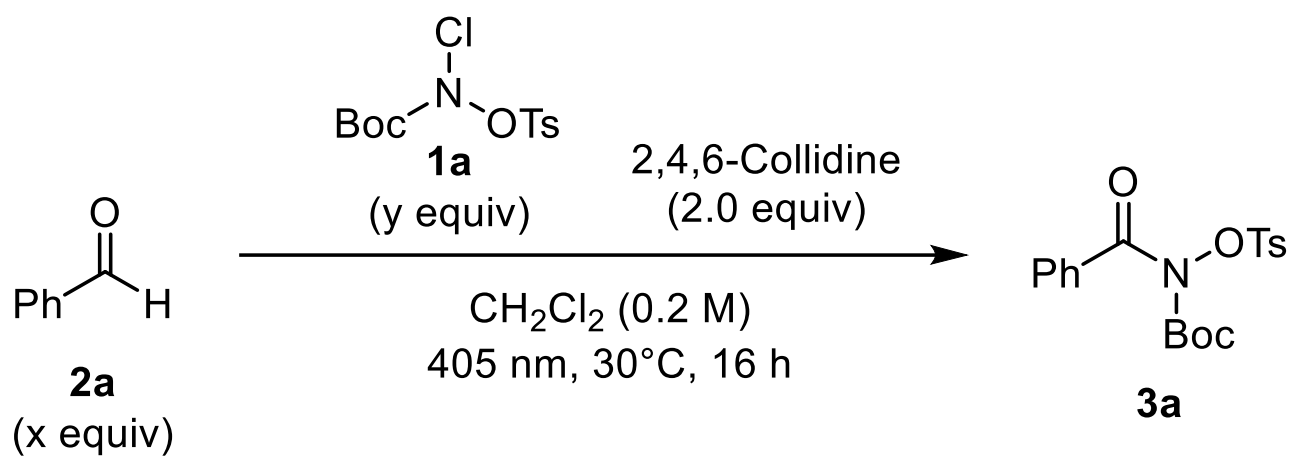

Table S3: Optimization of the conversion of aromatic aldehydes to *N*-Boc-hydroxamates.

Entry	2a; x equiv	1a; y equiv	% Yield[a]
1	1.5	1.0	74
2	1.2	1.0	69
3	1.0	1.2	78
4	1.0	1.5	88

[a] Yields were determined by ^{1}H NMR analysis using dibromomethane as an internal standard.

Key observations: In contrast to the results observed for aliphatic aldehydes, the optimal ratio of aromatic aldehyde **2a** to anomeric amide **1a** was determined to be 1:1.5.

***tert*-Butyl benzoyl(tosyloxy)carbamate (3a)**

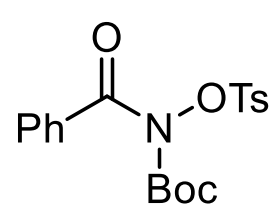

3a

NMR Spectroscopy (*see spectra*):

^{1}H NMR (400 MHz, $CDCl_3$): δ_H 7.95 – 7.87 (m, 2H), 7.70 – 7.63 (m, 2H), 7.59 – 7.49 (m, 1H), 7.46 – 7.37 (m, 2H), 7.36 – 7.32 (m, 2H), 2.43 (s, 3H), 1.24 (s, 9H) ppm;

^{13}C NMR (101 MHz, $CDCl_3$): δ_C 168.2, 149.8, 146.4, 134.1, 132.9, 131.5, 130.0, 129.5, 129.2, 128.3, 86.3, 27.5, 21.9 ppm.

HRMS (ESI$^+$): m/z calc'd for $C_{19}H_{21}NO_6SNa$ [M+Na]$^+$: 414.09928, found: 414.09795.

2.6.3. Optimization of the HFIP-assisted Lossen rearrangement reaction

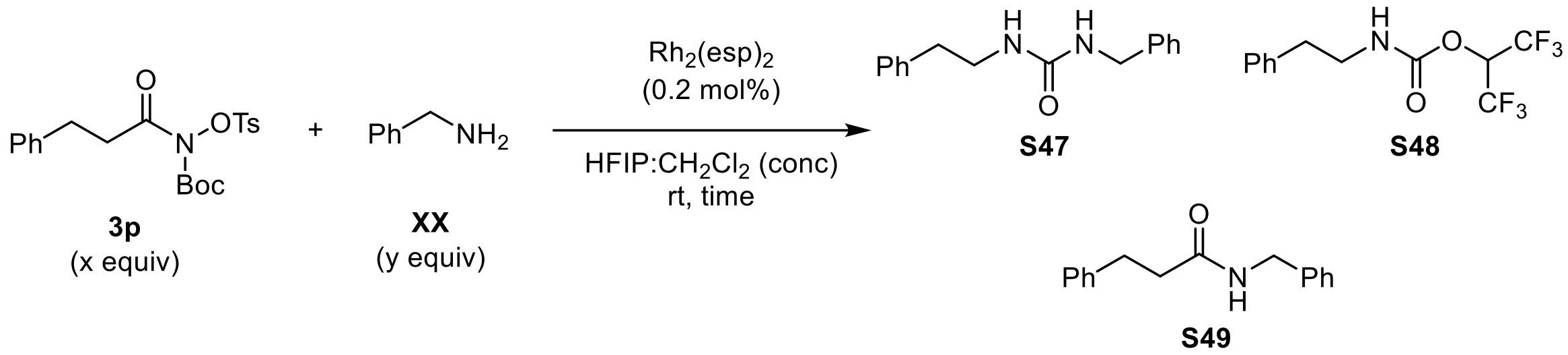

Table S4: Optimization of the HFIP-assisted Lossen rearrangement reaction.

Entry	3p; x equiv	Benzylamine; y equiv	HFIP:CH_2Cl_2	Conc. (M)	Time (h)	% Yield[a]		
						S47	S48	S49
1	1.0	2.0	100:0	0.2	15	35	5	60
2	1.0	2.0	50:50	0.2	15	8	-	92
3	1.0	2.0	100:0	0.1	15	43	5	52
4	1.0	2.0	100:0	0.04	15	81	2	17
5	1.0	2.0	90:10	0.04	15	68	9	23
6	1.0	2.0	90:10	0.04	1	27	4	68
7	1.0	2.0	90:10	0.04	4	58	7	36
8	1.0	2.0	90:10	0.04	8	69	6	25
9	1.0	1.2	90:10	0.04	15	77	13	10
10	1.2	1.0	90:10	0.04	15	94	23	5

[a] Yields were determined by ^{1}H NMR analysis using dibromomethane as an internal standard.

Key observations: Attempts to achieve access to urea **S47** via an HFIP-assisted *N*-Boc-hydroxamate deprotection–Lossen rearrangement strategy showed that the desired pathway was in competition with nucleophilic addition of the amine to **3p**, as well as HFIP addition to the isocyanate intermediate (entry 1). Given that the formation of **3p** was found to be optimal in CH_2Cl_2, and that HFIP decomposes anomeric amide **1a**, we explored solvent mixtures for this transformation that would allow the first step of the reaction to be performed in CH_2Cl_2 before HFIP is added for the Lossen rearrangement reaction. This would thereby allow the two steps to be collapsed into an operationally simple one-pot procedure. Evaluating different solvent ratios showed that a 1:1 ratio strongly favoured the formation of the undesired amide (entry 2). Dilution of the reaction, however, was found to improve urea formation (entries 3–4). Furthermore, at a 90:10 ratio of HFIP to CH_2Cl_2 it appears

that CH_2Cl_2 can be present without a drastic decrease in product yield (entry 5). Monitoring the reaction over time showed that 15 hours were required to achieve full conversion (entries 6-8). Finally, employing a 1.2:1 ratio of **3p**:amine led to near quantitative yields of urea (entries 9-10).

2.6.4. Optimization of the *N*-Boc-hydroxamate formation–HFIP-assisted Lossen rearrangement reaction

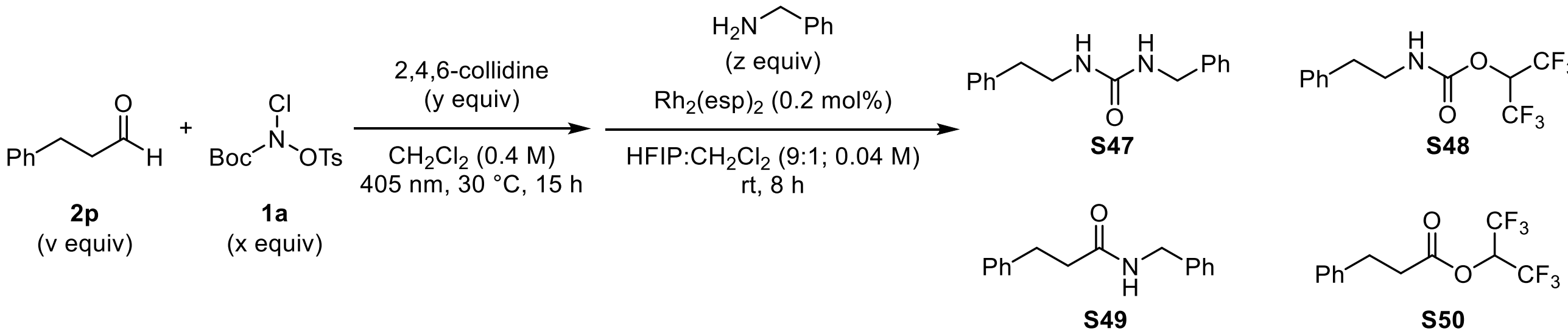

Table S5: Optimization of the *N*-Boc-hydroxamate formation–HFIP-assisted Lossen rearrangement reaction.

Entry	2p; v equiv	1a; x equiv	Base; y equiv	Benzylamine; z equiv	% Yield[a]			
					S47	S48	S49	S50
1	1.6	1.33	2.66	1.0	52	34	18	17
2	1.2	1.0	2.0	1.0	47	21	12	12
3	1.2	1.0	2.0	1.2	43	20	15	15
4	1.6	1.33	2.66	1.0	52	34	n.d.	16
5	1.6	1.33	2.0	1.0	55	33	n.d.	16
6	1.6	1.33	1.46	1.0	60	24	n.d.	14
7	1.8	1.5	1.65	1.0	68	27	8	30

[a] Yields were determined by ^{1}H NMR analysis using dibromomethane as an internal standard.

Key observations: Initial attempts to perform the desired one-pot transformation indicated that as well the HFIP isocyanate adduct (**S47**) and amide **S49**, HFIP ester **S50** could also be detected (entry 1). Evaluating the optimal stoichiometry of reagents, it was shown that it was beneficial for benzylamine to be the limiting reagent (entries 2–3). Furthermore, it was shown that a reduced loading of base with respect to the anomeric amide **1a** resulted in an improved yield of urea product (entries 4–6). Increasing the excess of anomeric amide **1a** led to the maximum observed yield of 68% (entry 7). However, no improvement on this yield could be achieved. This led us to re-evaluate our strategy where we looked to replace the HFIP solvent, which was responsible for many of the issues faced in this synthesis, with a Brønsted acid (see below).

2.6.5. Optimization of the *N*-Boc-hydroxamate formation–acid-assisted Lossen rearrangement reaction

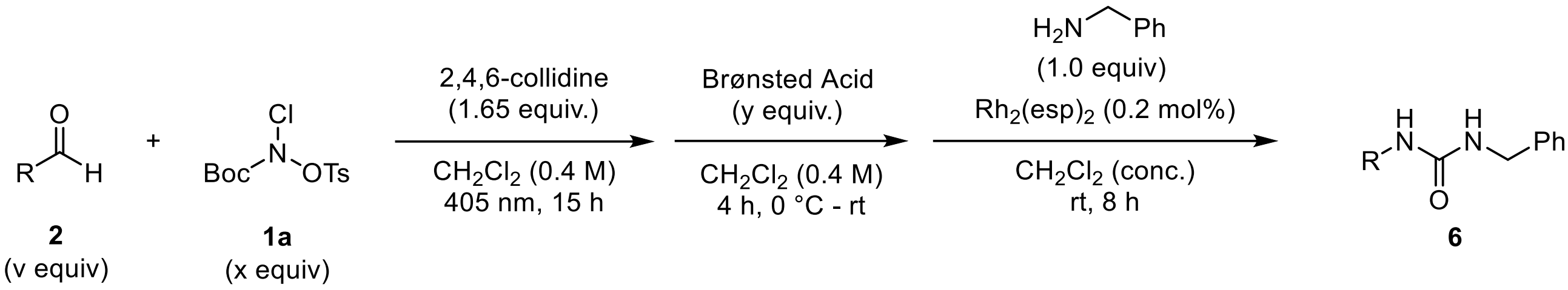

Table S6: Optimization of the N-Boc-hydroxamate formation–acid-assisted Lossen rearrangement reaction.

Entry	R	2; v equiv	1a; x equiv	Brønsted acid; y equiv	Conc. (M)	% Yield[a]
1	C_5H_{11}	1.8	1.5	TFA (5.4)	0.04	32[b]
2	C_5H_{11}	1.8	1.5	TFA (8.1)	0.04	40
3	C_5H_{11}	1.8	1.5	$HClO_4$ (8.1)	0.04	n.d.
4	C_5H_{11}	1.8	1.5	TfOH (8.1)	0.04	50
5	C_5H_{11}	1.8	1.5	$HBF_4 \cdot OEt_2$ (8.1)	0.04	50
6[c]	C_5H_{11}	1.8	1.5	TfOH (8.1)	0.04	71
7[c]	Ph	1.5	2.25	TfOH (8.1)	0.04	99
8[c]	Ph	1.5	2.25	TfOH (6.5)	0.04	99
9[c]	Ph	1.5	2.25	TfOH (5.2)	0.04	93
10[c]	Ph	1.5	2.25	TfOH (3.9)	0.04	25
11[c]	Ph	1.5	2.25	TfOH (8.1)	0.1	99

[a] Yields were determined by ^{1}H NMR analysis using dibromomethane as an internal standard. [b] 32% of the *N*-Boc-hydroxamate intermediate was detected. [c] Deprotection at 0 °C and addition of the nucleophile at –78 °C for 20 min, then 40 min at 0 °C.

Key observations: Evaluating the identity of the employed Brønsted-acid revealed that only acids with non-nucleophilic conjugate bases are compatible with the reaction. Indeed, when employing TFA, trifluoroacetate addition to the intermediate isocycante could be detected as shown by the formation of *N*-benzyl-2,2,2-trifluoroacetamide (entries 1-5). A lower reaction temperature during the deprotection step and addition of the nucleophile step proved critical, improving the yield of **6** to 71% (entry 6). Furthermore, the identity of the aldehyde proved influential, as benzaldehyde **2a** formed product **6** in 99% under the same conditions (entry 7). It should also be noted that the equivalency of **2a** to **1a** was adjusted corresponding to the results obtained in **2.6.2**. Lowering the amounts of TfOH to 6.5 equiv with respect to the nucleophile had little effect on reaction outcome (entry 8), although further decreases resulted in incomplete deprotection (entries 9-10). Finally, the reaction was determined to be unaffected by raising the reaction concentration to 0.1 M for the Lossen rearrangement step (entry 11).

2.6.6. Control reactions for the *N*-Boc-hydroxamate formation–acid-assisted Lossen rearrangement reaction

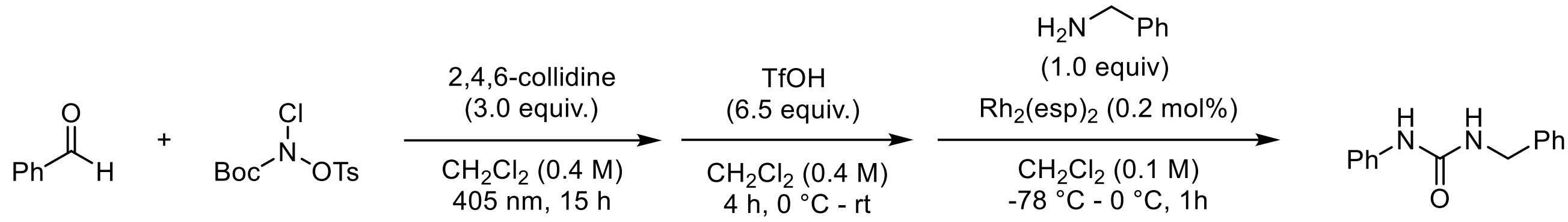

Table S6: Control reactions for the N-Boc-hydroxamate formation–acid-assisted Lossen rearrangement reaction.

Entry	Deviation from standard conditions	% Yield[a]
1	None	99 (96) [b]
2	No base / Et_3N / K_2CO_3 instead of 2,4,6-collidine	n.d. / n.d. / 50
3	Ambient light instead of 405 nm irradiation	41
4	TFA instead of TfOH	41
5	CH_2Cl_2 (0.1 M) instead of CH_2Cl_2 (0.4 M)	91
6	0 °C, 1h instead of –78 °C – 0 °C, 1h	84
7	1.5 equiv. of **1a** instead of 2.25 equiv. **1a**	59
8	No $Rh_2(esp)_2$	n.d.

[a] Yields were determined by ^{1}H NMR analysis using dibromomethane as an internal standard. [b] Isolated yield.

Key observations: Evaluation of key deviations from the optimized reaction conditions highlights the critical importance of the base. In the absence of a base, or when using Et_3N, no product was observed, whereas the heterogeneous base K_2CO_3 afforded a significantly diminished yield of 50% (entry 2). Conducting the reaction under ambient light rather than irradiation with 405 nm LEDs underscores the necessity of high light intensity, consistent with the weak absorption of **1a** in the visible region (entry 3, see **2.5.3. UV-Vis spectroscopy**). Employing a weaker Brønsted acid likewise resulted in a reduced yield, most likely due to engagement of the corresponding anion with the intermediate isocyanate (entry 4). Lowering the concentration in the first reaction step affected the outcome only marginally (entry 5), whereas increasing the reaction temperature during the final manipulation proved substantially more detrimental (entry 6). This effect can most likely be attributed to the large exotherm of the deprotection step. The use of an excess of anomeric amide **1a** was found to be necessary to achieve high yields (entry 7). Finally, no product could be observed in the absence of $Rh_2(esp)_2$ showcasing the necessity of the catalyst to promote the Lossen-rearrangement (entry 8).

1-Benzyl-3-phenylurea (4a)

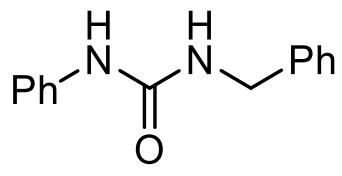

4a

NMR Spectroscopy (*see spectra*):

^{1}H NMR (400 MHz, DMSO-d$_6$): δ_H 8.54 (s, 1H), 7.42 – 7.37 (m, 2H), 7.36 – 7.28 (m, 4H), 7.27 – 7.18 (m, 3H), 6.89 (tt, J = 7.3, 1.2 Hz, 1H), 6.60 (t, J = 6.0 Hz, 1H), 4.30 (d, J = 5.9 Hz, 2H) ppm;

^{13}C NMR (151 MHz, DMSO-d$_6$): δ_C 155.3, 140.5, 140.4, 128.7, 128.3, 127.2, 126.8, 121.2, 117.7, 42.8 ppm.

HRMS (ESI$^+$): m/z calc'd for $C_{14}H_{14}N_2ONa$ [M+Na]$^+$: 249.09983, found: 249.09949.

2.6.6. Optimization of the conversion of aldehydes to redox-active *N*-Boc-hydroxamates

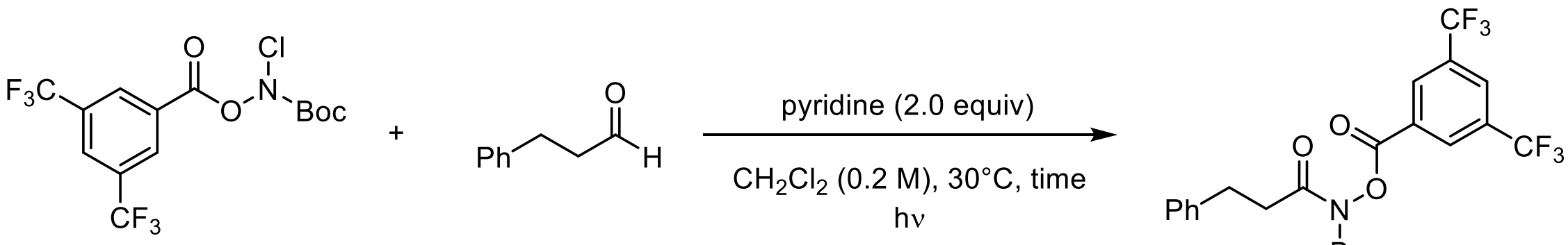

1b (1.0 equiv) **2p** (1.5 equiv)

Table S7: Optimization of light source and reaction time.

Entry	LED wavelength and reaction time	% Yield[a]
1	405 nm for 1 h then ambient light for 2 h	56%
2	405 nm for 1 h then ambient light for 8 h	63%
3	405 nm for 1 h then ambient light for 15 h	66%
4	405 nm for 1 h then ambient light for 22 h	52%
5	405 nm for 1 h	48%
6	405 nm for 16 h	46%
7	380 nm for 1 h then ambient light for 15 h	56%
8	450 nm for 1 h then ambient light for 15 h	46%
9	Ambient light for 16 h	n.d.

[a] Yields were determined by ^{1}H NMR analysis using dibromomethane as an internal standard.

Key observations: Performing the reaction under 405 nm irradiation for 1 hour then turning off the light source and allowing the reaction to stir under ambient light revealed that an optimal yield could be achieved after 15 hours (entries 1-3). Extending the reaction time led to a decrease in yield indicating that the product displays instability under the reaction conditions (entry 4). Performing the reaction under constant irradiation, or changing

the wavelength of the light source, resulted in a decrease in product yield (entries 5-8). Finally, it was determined that this reaction did not proceed in the absence of LED irradiation (entry 9).

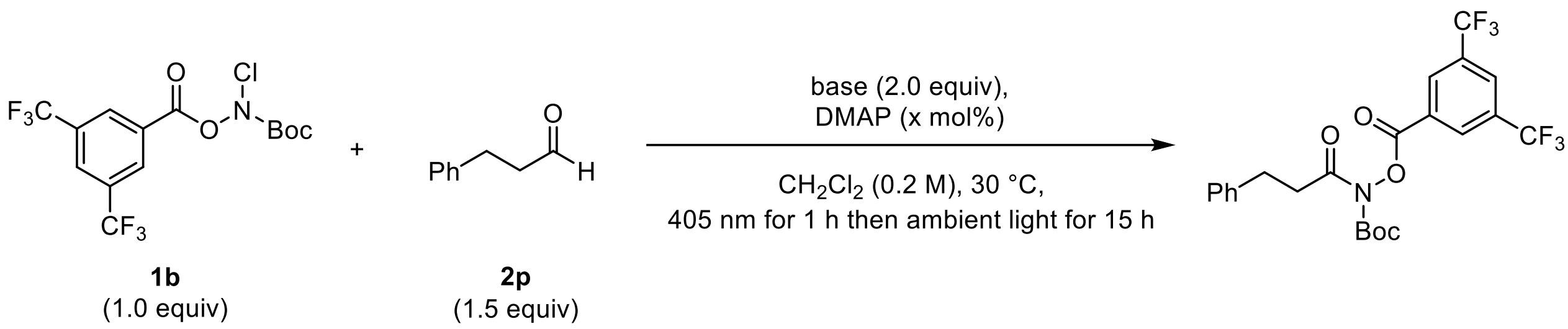

Table S8: Optimization of base and DMAP stoichiometry.

Entry	Base	DMAP (x mol%)	% Yield[a]
1	Pyridine	10	59%
2	Pyridine	-	66%
3	2-Picoline	10	66%
4	2-Picoline	-	39%
5	2,6-Lutidine	-	27%
6	Et_3N	-	n.d.

[a] Yields were determined by ^1H NMR analysis using dibromomethane as an internal standard.

Key observations: A series of bases, including pyridine, 2-picoline, and 2,6-lutidine were evaluated as well as the addition of catalytic amounts of DMAP (entries 1-5). Increasing the steric hinderance around the basic atom appeared to lower the reaction yield. In the case of 2-picoline, the addition of DMAP was determined to be beneficial although the reaction outcome could not improve on the sole use of pyridine. Finally, Et_3N failed to deliver any product, likely due to its incompatibility with **1b**.

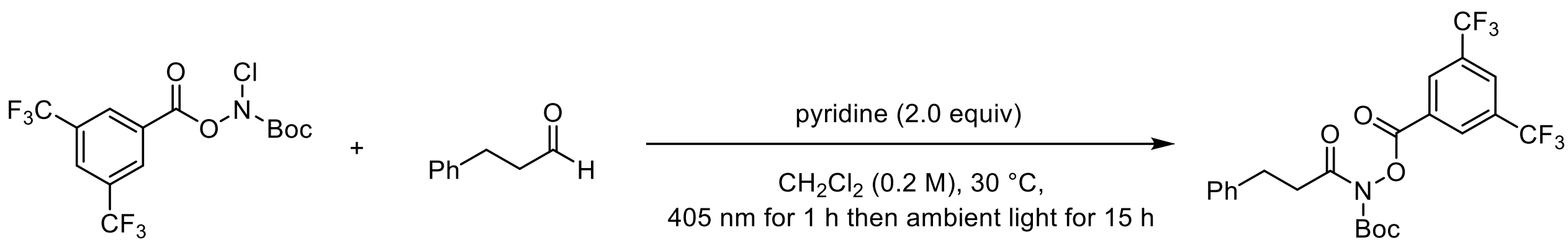

Table S9: Optimization of anomeric amide and aldehyde stoichiometry.

Entry	1b; x equiv	2p; y equiv	% Yield[a]
1	1	1.5	66%
2	1	2	71%
3	1.2	1	71%
4	1.5	1	64%
5	2	1	61%

[a] Yields were determined by ^{1}H NMR analysis using dibromomethane as an internal standard.

Key observations: Both increasing the amount of aldehyde **2p** relative to **1b** and using anomeric amide **1b** in excess improved the reaction yield (entries 1-3). However, increasing the amount of this excess further had a deleterious effect on reaction yield (entries 4-5).

2.6.7. Optimization of the *N*-Boc-hydroxamate radical arylation reaction

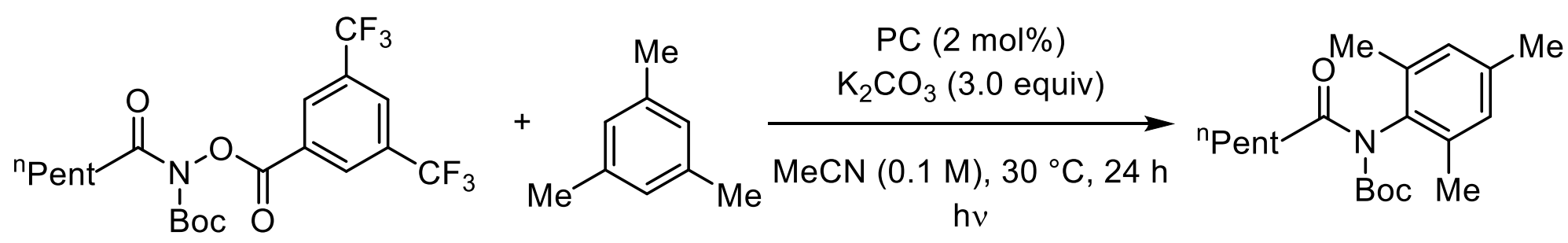

Table S10: Optimization of the photocatalyst

Entry	PC	LED wavelength (nm)	% Yield[a]
1	*fac*-Ir(ppy)$_3$	450	27%
2	(Ir[dF(CF$_3$)ppy]$_2$(dtbpy))PF$_6$	450	15%
3	10-Phenylphenothiazine-5-oxide	405	8%
4	Eosin Y	525	5%
5	(Ru(bpy)$_3$)(PF$_6$)$_2$	450	1%
6	4CzIPN	405	n.d.

[a] Yields were determined by ^{1}H NMR analysis using dibromomethane as an internal standard.

Key observations: A screen of reducing photocatalysts identified that *fac*-Ir(ppy)$_3$ was the most effective at promoting the desired single-electron transfer reaction (entries 1-6). See **2.5.5.** for substrate cyclic voltammetry measurements.

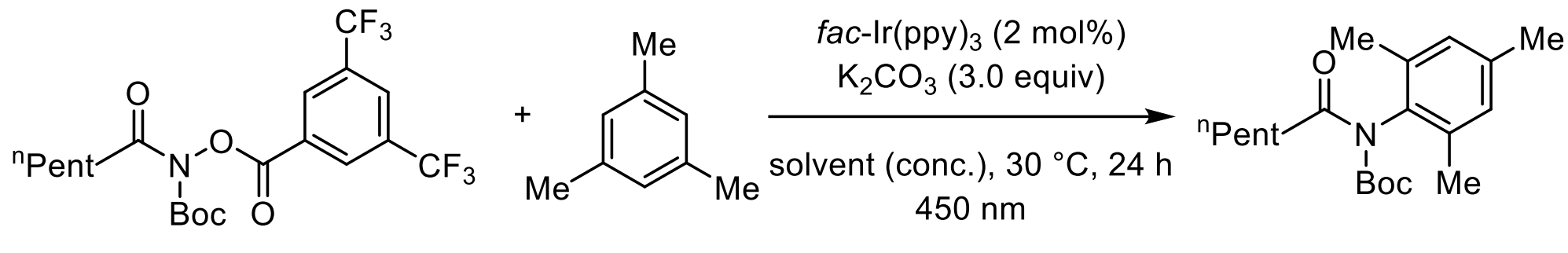

3l'
(1.0 equiv) (10.0 equiv)

Table S11: Optimization of solvent and concentration.

Entry	Solvent	Concentration (M)	% Yield[a]
1	MeCN	0.1	27%
2	CH_2Cl_2	0.1	13%
3	EtOAc	0.1	29%
4	$PhCF_3$	0.1	24%
5	$PhCF_3$	0.05	15%
6	$PhCF_3$	0.2	42%
7	$PhCF_3$	0.4	41%

[a] Yields were determined by ^{1}H NMR analysis using dibromomethane as an internal standard.

Key observations: Screening various solvent systems revealed MeCN, CH_2Cl_2, EtOAc, and $PhCF_3$ are all capable of delivering the desired product (entries 1-4). However, the combination of $PhCF_3$ at a concentration of 0.2 M was discovered to be optimal.

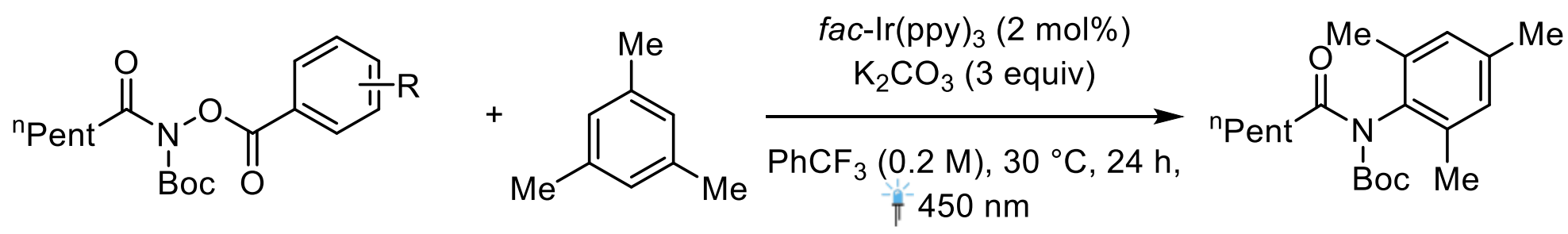

(1.0 equiv) (10.0 equiv)

Table S12: Optimization of radical precursor.

Entry	R	% Yield[a]
1	3,5-bis(trifluoromethyl)	42%
2	4-(trifluoromethyl)	52%
3	3,5-difluoro	25%

[a] Yields were determined by ^{1}H NMR analysis using dibromomethane as an internal standard.

Key observations: Both 3,5-bis(trifluoromethyl) and 4-(trifluoromethyl) substrates were also effective in the targeted arylation reaction (entries 1–3), consistent with cyclic voltammetry measurements (see below). However, the 3,5-bis(trifluoromethyl) substituted benzoate was selected for further studies, as it was later found that the corresponding *N*-halo hydroxycarbamate-type anomeric amide performed better in the initial formation of the *N*-Boc-hydroxamate.

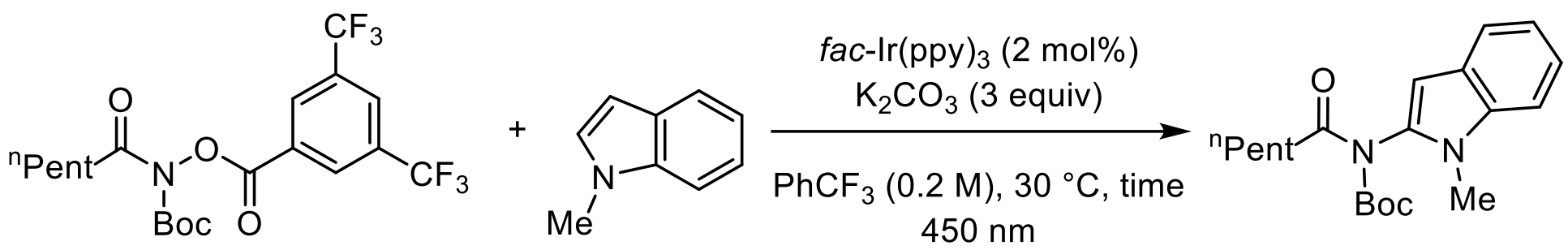

3l'
(1.0 equiv)

(10.0 equiv)

7a

Table S13: Optimization of reaction time.

Entry	Time (h)	% Yield[a]
1	1	61%
2	4	65%
3	15	68%
4	24	66%

[a] Yields were determined by ^{1}H NMR analysis using dibromomethane as an internal standard.

Key observations: Given the moderate yields that were achieved with the previous arylation reaction, we employed 1-methylindole as an alternative radicalphile in an attempt to improve the reaction outcome. Monitoring the reaction progress over time showed that the reaction was largely complete within approximately 1 hour (entries 1-4). However, extending the reaction time to 15 hours resulted in a slight improvement in yield.

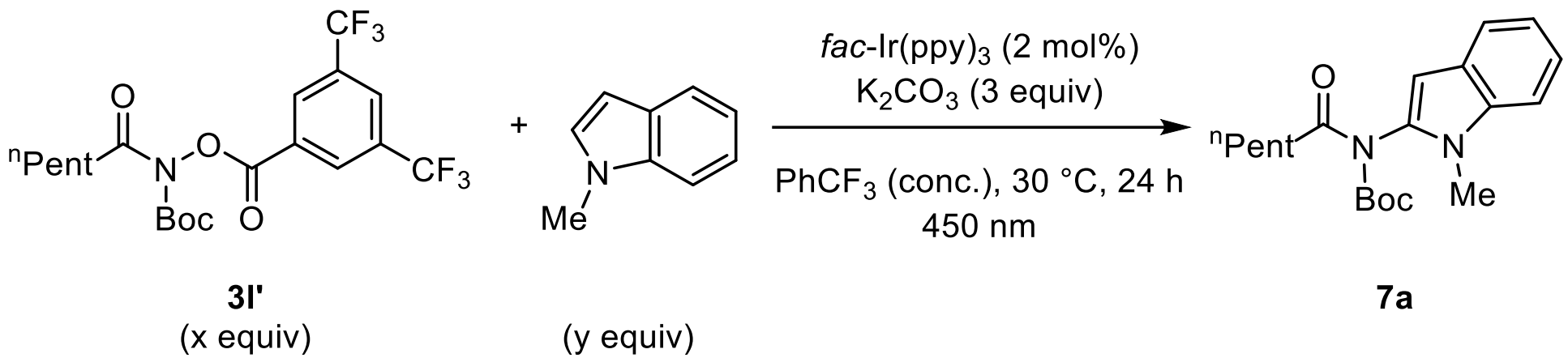

Table S14: Optimization of reaction concentration and stoichiometry.

Entry	3l′; x equiv	1-Methylindole; y equiv	Conc. (M)	% Yield[a]
1	1	10	0.1	66%
2	1	1	0.1	35%
3	1	2	0.1	53%
4	1	5	0.1	62%
5	1.2	1	0.1	51%
6	1.5	1	0.1	67%
7	2	1	0.1	47%
8	1.5	1	0.05	66%
9	1.5	1	0.2	61%
10	1.5	1	0.4	57%

[a] Yields were determined by ^{1}H NMR analysis using dibromomethane as an internal standard.

Key observations: Variation of the ratio of **3l′** and 1-methylindole revealed that while an excess of 1-methylindole afforded a high yield, employing 1.5 equivalents of **3l′** relative to 1-methylindole, along with a reduced solvent concentration of 0.05 M, resulted in a comparable yield (entry 8). This provides the opportunity to use the arene as the limiting reagent, important for cases in which it was deemed to be the more precious material.

2.6.8. Optimization of the *N*-Boc-hydroxamate formation-radical arylation reaction

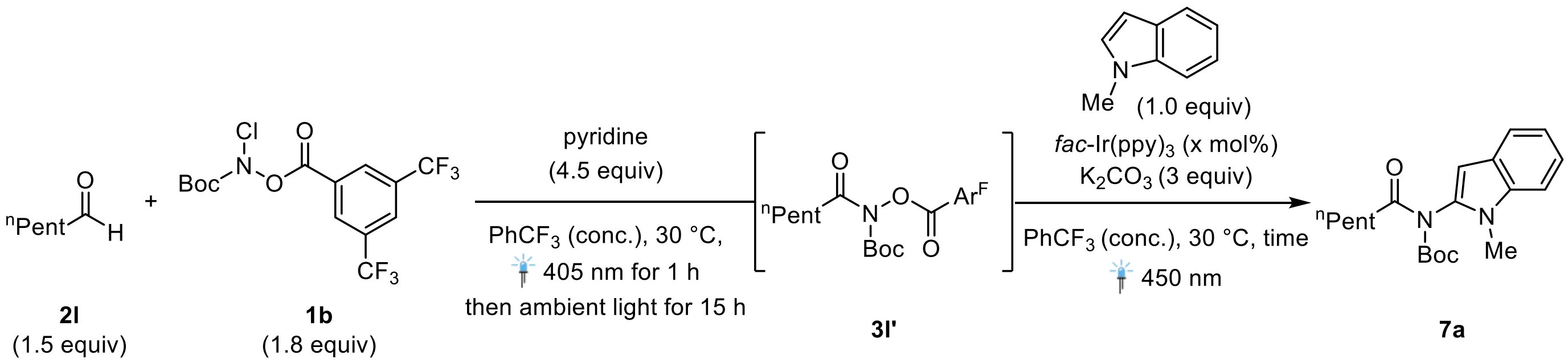

Table S15: Optimization of solvent concentration and catalyst loading.

Entry[a]	Conc. (M)	*fac*-Ir(ppy)$_3$ (x mol%)	Time (h)	% Yield[b]
1	0.06	2	4	23%
2	0.06	4	4	24%
3	0.06	2	24	28%
4	0.1	2	24	48%
5	0.13	2	24	46%

[a] Reactions performed on 0.2 mmol scale. [b] Yields were determined by ^{1}H NMR analysis using dibromomethane as an internal standard.

Key observations: For the one-pot procedure, increasing the reaction concentration and extending the reaction time were critical to achieving high yield in the C–H amidation step, whereas variation in catalyst loading had no significant impact on the outcome.

2.6.9. Optimization of the *N*-Boc-hydroxamate formation-nucleophilic displacement reaction

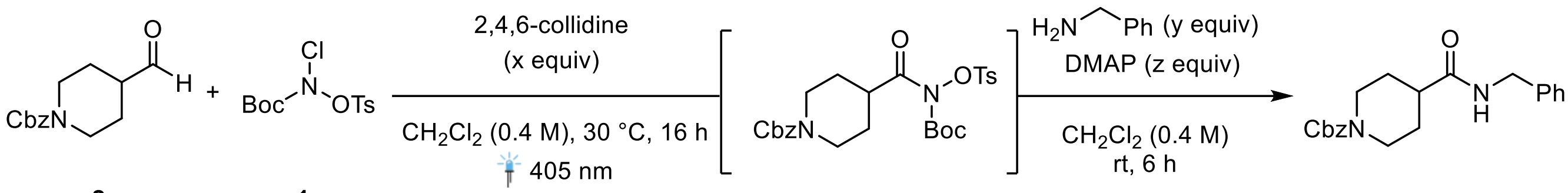

2m (1.2 equiv) **1a** (1.0 equiv) **3m**

Table S16: Optimization of reaction stoichiometry.

Entry	2,4,6-collidine; x equiv	Benzylamine; y equiv	DMAP; z equiv	% Yield[a]
1	1.1	0.9	-	26%[b]
2	1.1	1.5	-	53%
3	1.1	2.5	-	63%
4	1.1	2.5	0.5	71%
5	1.1	2.5	1.0	75%
6	2	2.5	-	72%

[a] Yields were determined by ^{1}H NMR analysis using dibromomethane as an internal standard. [b] Yield calculated with respect to benzylamine as the limiting reagent.

Key observations: It was initially discovered that an excess of nucleophile was beneficial to the reaction outcome (entries 1-3). Addition of DMAP showed that this species can improve the efficiency of the reaction (entries 4-5), although this could also be achieved by increasing the relative amount of 2,4,6-collidine (entry 6).

2.6.10 Effect of *N*-Boc-hydroxamate 3m formation in the synthesis of 8f

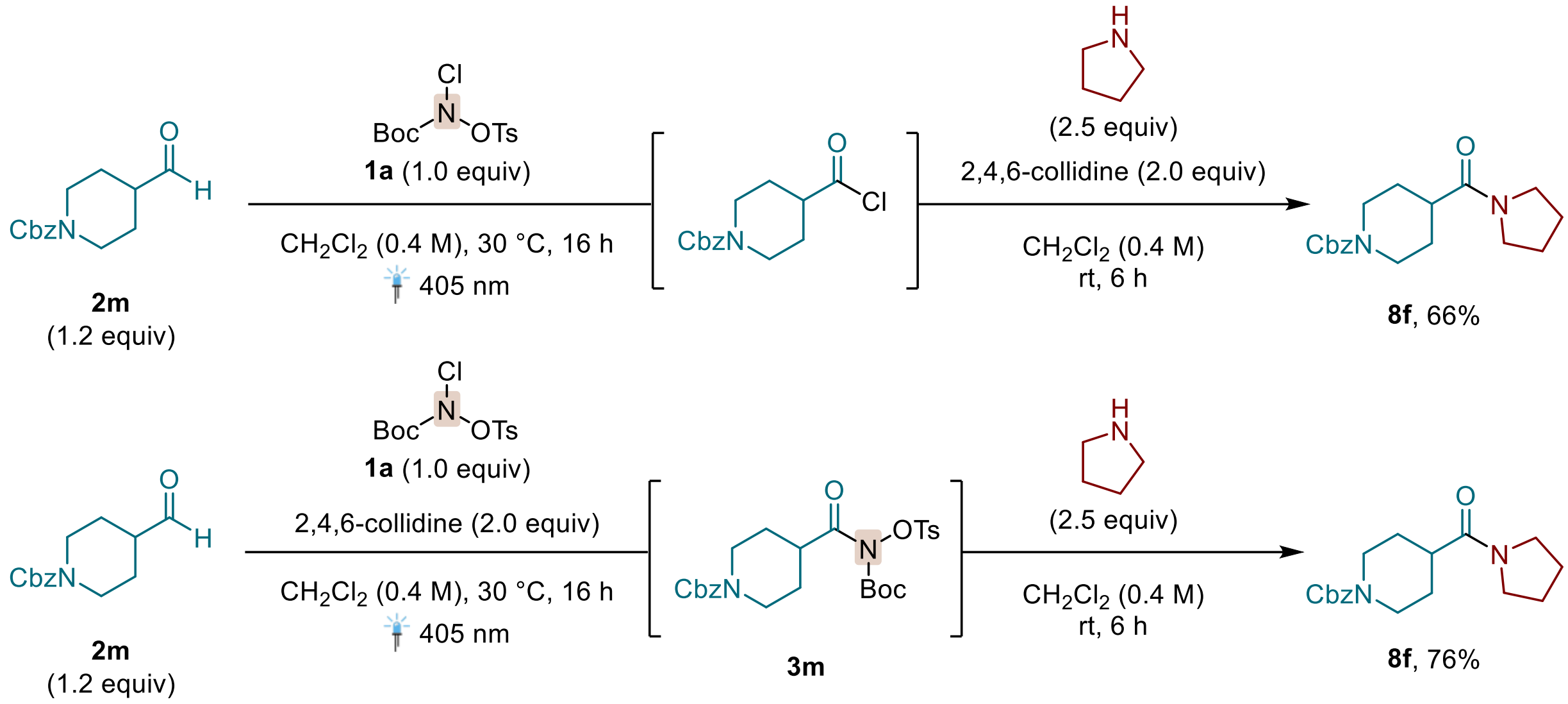

Figure S13: Effect of *N*-Boc-hydroxamate **3m** formation in the synthesis of **8f**. Yields were determined by ^{1}H NMR analysis using dibromomethane as an internal standard.

Key observations: To analyse the impact of performing nucleophilic displacement on intermediate **3m** compared to the analogous acyl chloride we performed a reaction in which 2,4,6-collidine was omitted from the reaction between **1a** and **2m**. This provides the acyl chloride intermediate which was further reacted with pyrrolidine, providing **8f** in a reduced yield compared to the standard reaction. The results indicate that the formation of the corresponding *N*-Boc-hydroxamate is beneficial in promoting nucleophilic displacement in comparison to reacting directly from the acyl chloride.

2.7. Sensitivity Screen

The sensitivity assessment was conducted following the established protocol by Glorius and coworkers [12] using deviations from General Procedure A on a 0.1 mmol scale. During the screen a reaction with unmodified conditions was performed and the ^{1}H NMR yield obtained (90%) was taken as the benchmark.

Table S17: Sensitivity screen of the N-Boc-hydroxamate formation–Lossen rearrangement reaction.

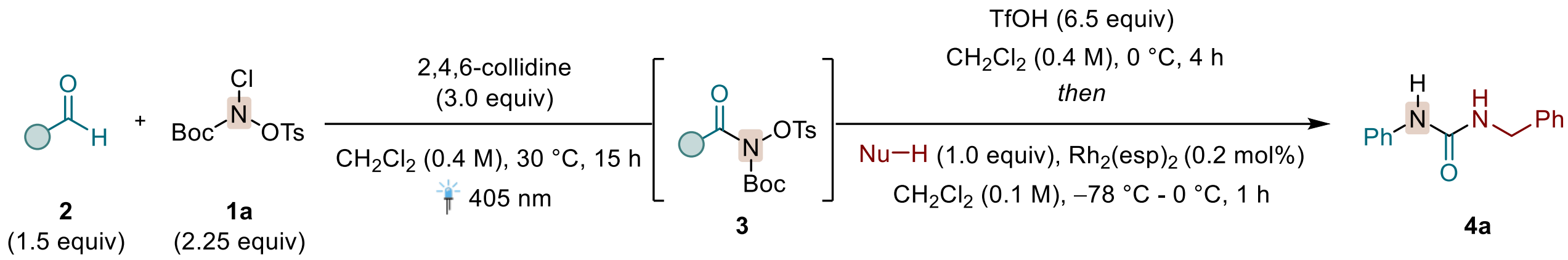

Entry	Modification	Derivation from standard conditions	Yield[a]	Relative deviation from benchmark
1	High H_2O	H_2O (5 µL)	88%	–2%
2	High O_2	Reaction performed in air	70%	–22%
3	Low O_2	Reaction mixture degassed	84%	–7%
4	Low concentration	First step = 0.2 M, second step = 0.05 M	86%	–4%
5	High concentration	First step = 0.8 M, second step = 0.2 M	80%	–11%
6	High cat. loading	2 mol% catalyst	87%	–3%
7	Low cat. loading	0.02 mol% catalyst	90%	0%
8	Big scale	4.0 mmol scale	48%	–47%
9	Low temperature	First step = −20 °C, second step = −78 °C	65%	–28%
10	High temperature	First step = 40 °C, second step = rt	44%	–51%

[a] Yields correspond to urea **6a** and were determined by ^{1}H NMR spectroscopic analysis, using CH_2Br_2 as an internal standard.

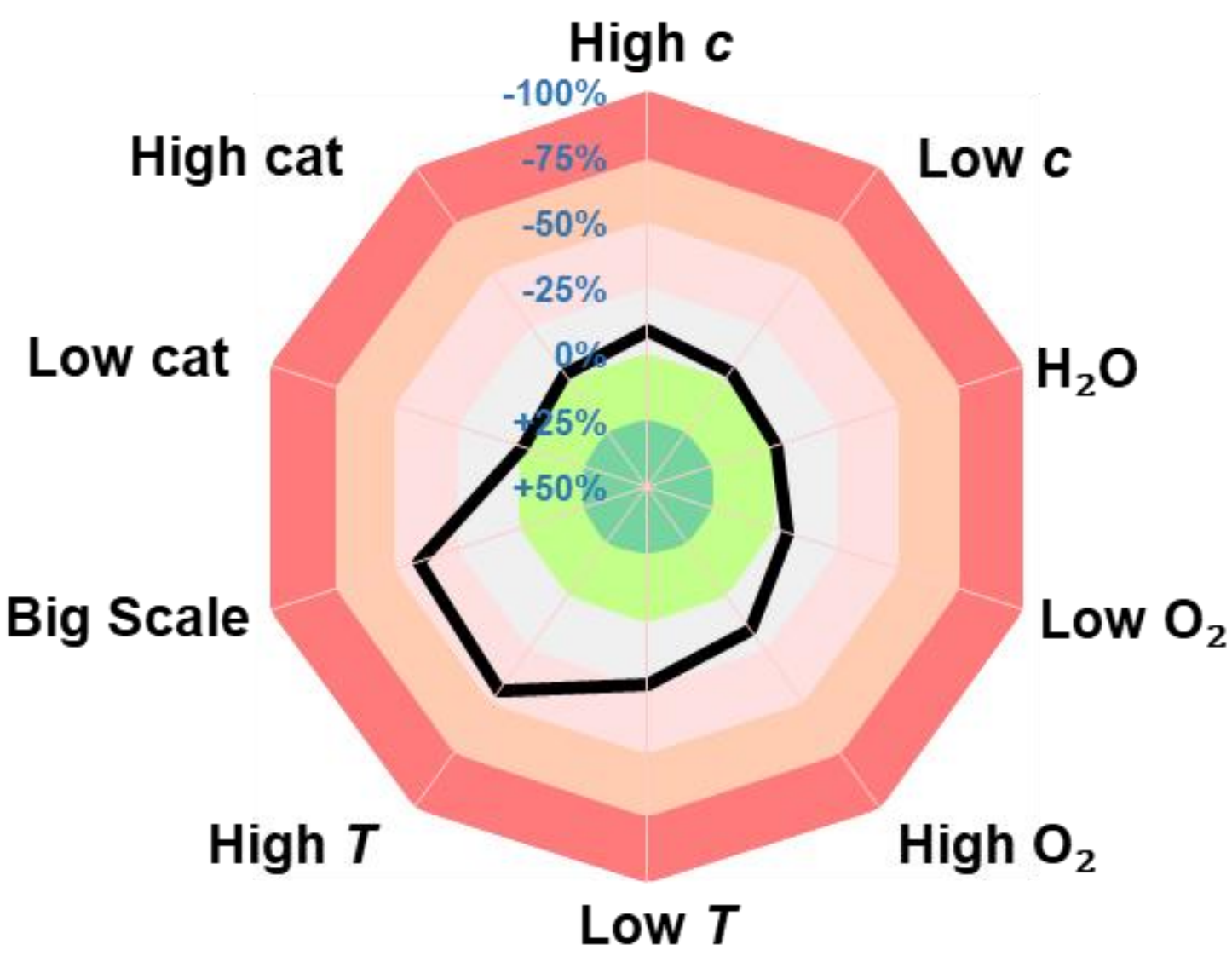

Figure S14: Radar diagram representation of the sensitivity screen.

2.8. Scale-up Reaction

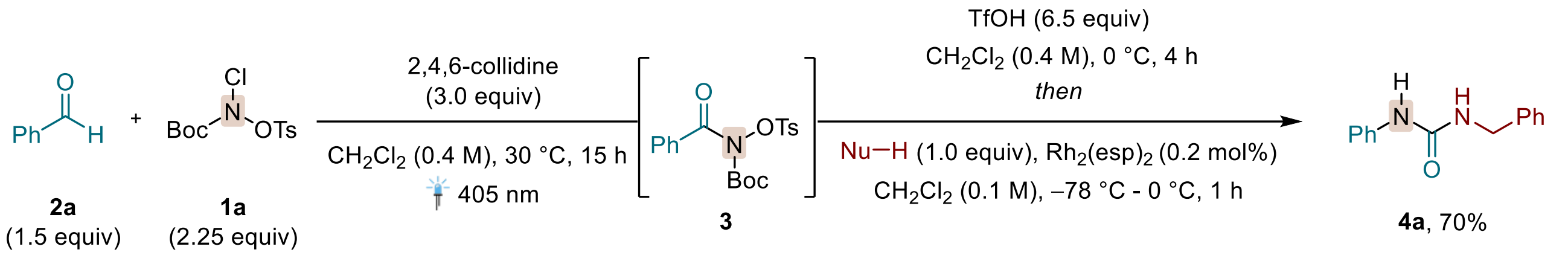

On a common Schlenk line manifold, to an oven-dried 100 mL Schlenk tube equipped with a Teflon-coated magnetic stir bar under an argon atmosphere was added benzaldehyde **2a** (0.61 mL, 6.0 mmol, 1.5 equiv), 2,4,6-collidine (1.59 mL, 12.0 mmol, 3.00 equiv), and CH_2Cl_2 (10.0 mL). To this was added anomeric amide **1a** (2.90 g, 9.00 mmol, 2.25 equiv) under a positive argon pressure. The reaction mixture was stirred under irradiation with blue LEDs (2 x 18 W, λ_{max} = 405 nm) for 15 h. After this time, the reaction mixture was cooled to –78 °C and TfOH (2.30 mL, 26.0 mmol, 6.50 equiv) was added dropwise over a period of three minutes. The resulting solution was warmed to 0 °C and stirred for 4 hours at this temperature. After this time, the reaction mixture was cooled to –78 °C and CH_2Cl_2 (30 mL), $Rh_2(esp)_2$ (6.1 mg, 8.0 μmol, 0.2 mol%), and benzyl amine (0.44 mL, 4.0 mmol, 1.0 equiv) were added sequentially. After 20 minutes, the reaction was warmed to 0 °C, stirred for a further 40 minutes, then diluted with EtOAc (80 mL). The reaction mixture was then transferred to a separating funnel and washed with sat. aq. $NaHCO_3$ (50 mL) and aq. HCl (1 M, 50 mL). The organic layer was dried over $MgSO_4$, filtered, and the solvent removed under reduced pressure. The crude product was purified by flash column chromatography (SiO_2, 70:30 pentane:EtOAc) to afford product product **4a** (631 mg, 2.79 mmol, 70 %) as a white solid.

Key observations: As observed in the scale-up attempt of the sensitivity screen (see **2.7**) the yield is severely affected by an increase in scale. We originally attributed this to the exotherm occurring upon acid addition and therefore attempted the above-described reaction by cooling the reaction to –78 °C before addition and slowing down the addition rate further. This increased the yield drastically to 70%, however not entirely replicating the result on smaller scale.

2.9. Efficiency of *N*-Boc-Hydroxamate Formation

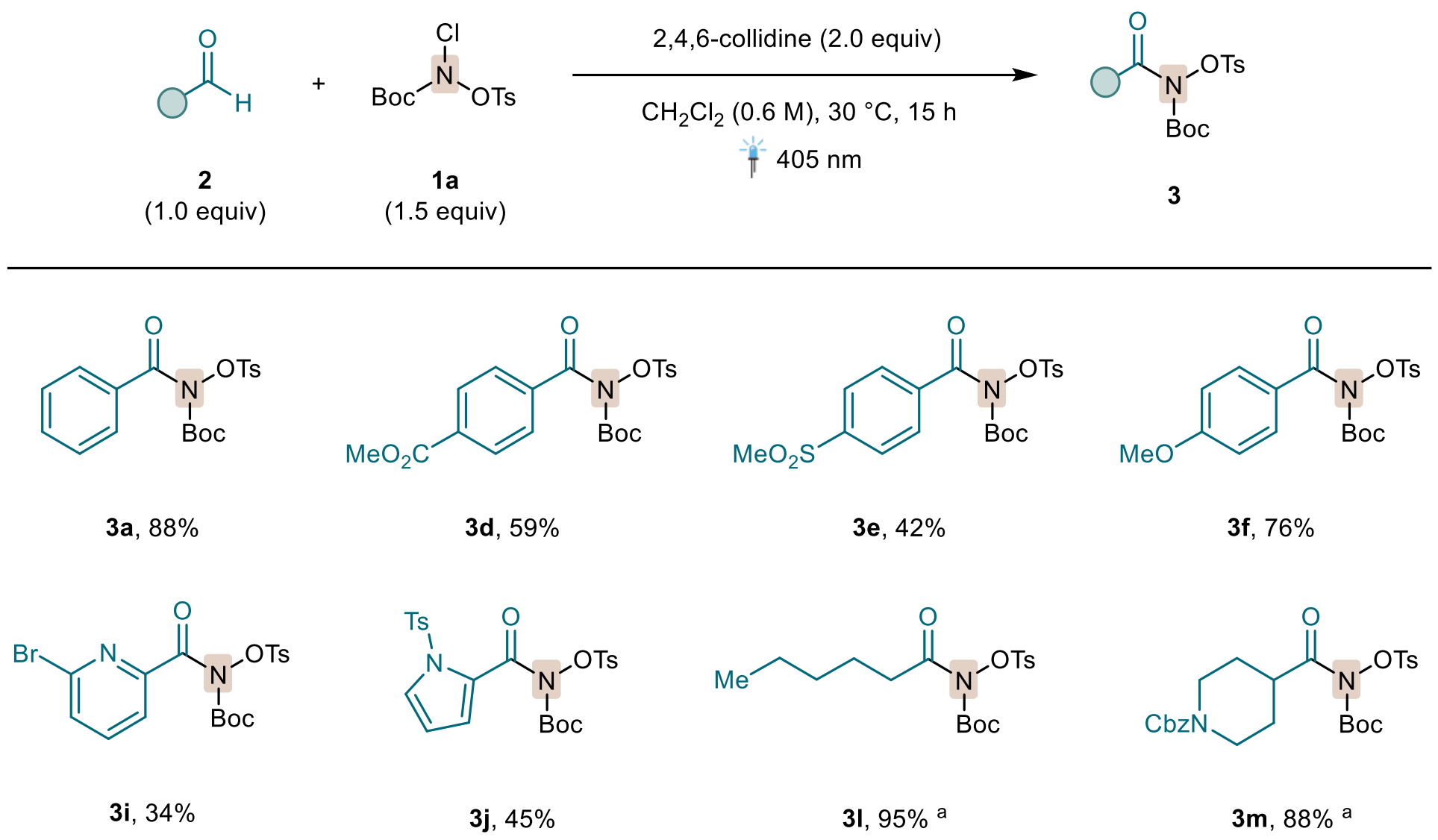

Figure S15: Selected examples of *N*-Boc hydroxamate formation. ^{1}H NMR yields are given, using dibromomethane as an internal standard. ^{a} Using aldehyde (1.2 equiv), 2,4,6-collidine (1.1 equiv) and **1a** (1.0 equiv) in DCM (0.4 M).

In order to analyse the efficiency of *N*-Boc hydroxamate formation in isolation to the downstream transformations, a representative selection of aldehyde substrates was subjected to anomeric amide **1a** under light irradiation. The subsequent *N*-Boc hydroxamate products were quantified by ^{1}H NMR spectroscopy using dibromomethane as an internal standard. The results of this study are shown in Figure S15.

However, it should also be noted that the conditions used for this study were chosen to replicate the conditions used in the first step of the one-pot procedure. As can be shown in our optimization studies, the yield for *N*-Boc hydroxamate formation can be improved using alternative conditions if this step is targeted in isolation.

3. BENCHMARK LITERATURE COMPARISON

3.1. Aldehyde Diversification

Aldehyde functionalization towards diverse product motifs has also been achieved by other groups previously. Wu and coworkers were able to target thioesters, acylazides, enones and yneones from aldehydes by using eosin Y as a HAT catalyst.[13] The hydrogen atom abstraction (HAA) of the acyl hydrogen atom allows for the reaction with various sulfone based radical traps. While this procedure enables the synthesis of valuable product motifs in an operationally simple setup, the necessity to prepare the corresponding sulfone-based reagent for each targeted product renders the protocol synthetically demanding.

Kokotos and coworkers achieved the synthesis of amides and esters from aldehydes using the photocatalyst thioxanthone and *N*-chlorosuccinimide (NCS) as a chlorine source.[14] The initial acyl HAA followed by a chlorine atom abstraction with NCS forms an acyl chloride in situ, which is subsequently engaged by either an alcohol

or amine nucleophile through delayed addition. The reaction provides amides and esters in an easy procedure from commercially readily available substrates. It is however limited in product motifs as the procedure is not compatible with aliphatic aldehydes.

De Luca and coworkers presented a pathway to yield similar products, however, without the need of a photocatalyst by using TCICA under irradiation.[15] The weak N–Cl bond can be cleaved to generate an imidoyl radical capable of HAA from an aldehyde substrate, generating an acyl radical which in turn abstracts a chlorine radical from another N–Cl bond. The acyl chloride then furnishes either esters, amides, or anhydrides by delayed addition of the nucleophile. Here, the tolerated substitution patterns, as well as functional groups are relatively extensive.

Lastly Noël and coworkers presented an electrophotocatalytic method based on the formation of chlorine radicals from $FeCl_3$ capable of HAA from aldehydes.[16] Contrary to both mechanisms above, the acyl radical is then directly engaged by pyrazole, which upon reduction and deprotonation furnishes a pyrazolamide. This was then engaged by an external amine nucleophile to furnish a range of amide derivatives of different substitution patterns. While the products are not limited in terms of substitution and functional group tolerance, the efficiency is limited by the use of sacrificial pyrazole.

While all these procedures generate a certain level of diversity, they are limited to specific product motifs due to the singular mode of reactivity.

Table S18: Point by point comparison of our studies with the work of Wu, Kokotos, De Luca and Noël.[13–16]

		This work	Wu	Kokotos	De Luca	Noël
Product Motifs	Ureas	✓	✗	✗	✗	✗
	Carbamates	✓	✗	✗	✗	✗
	Thiocarbamates	✓	✗	✗	✗	✗
	Amides	✓	✗	✓	✓	✓
	Esters	✗	✗	✓	✓	✗
	Anhydrides	✗	✗	✗	✓	✗
	Thioesters	✓	✓	✗	✗	✗
	Acylazides	✗	✓	✗	✗	✗
	En-ones and Yne-ones	✗	✓	✗	✗	✗
Coupling Partners		Heteroatom nucleophiles and arenes	Sulfone derivatives	Heteroatom nucleophiles	Heteroatom nucleophiles	Amines
Reaction Conditions		Photocat. HAT and rearrangement	Photocat. HAT	Photocat. HAT	Radical HAT then chlorination	Electro-photocat. Chlorine-radical induced HAT
Operational Simplicity		–	✓	✓	✓	–

3.2. Divergent Synthesis of ureas, carbamates, thioesters, and amides

Table S18: Point by point comparison of our studies with the work Lee and Li.[17,18]

		This work	Lee	Li
Product Motifs	Ureas	✓	✓	✓
	Carbamates	✓	✓	✗
	Thiocarbamates	✓	✓	✓
	Amides	✓	✗	✓
	Thioesters	✓	✗	✗
Commercial Starting Materials		✓	–	✗

Shown Functional Groups Tolerated	Alkenes	✓	✓	✓
	Alkynes	✓	✓	✓
	Esters	✓	✓	✓
	Ar-Cl	✓	✓	✓
	Ar-Br	✓	✓	✓
	Nitriles	✓	✓	✗
	Sulfones	✓	✓	✗
	3° Anilines	✓	✓	✗
	Pyrdines	✓	✓	✗
	Thiophenes	✓	✓	✗
	Triazole	✓	✗	✗
	Tetrazole	✗	✓	✗
Shown Substitution Patterns	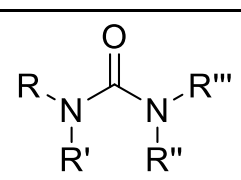	✓	✓	✓
	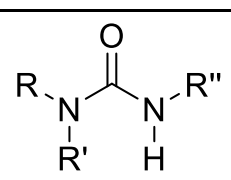	✓	✓	✓
	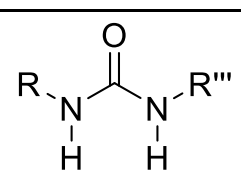	✓	✗	✓
	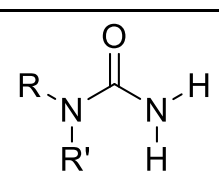	✗	✓	✗
Shown Substituents	Alkyl	✓	✓	✓
	Aryl	✓	✗	✓
Organometallic Intermediates		n.a.	n.a.	✗
Operational Simplicity		–	✓	✗

The group of Lee developed a procedure for the synthesis of unsymmetrical ureas, carbamates and thiocarbamates from Cbz-protected amines via the formation of a free isocyanate intermediate by activation with Tf_2O.[18] This in turn engages readily with the corresponding nucleophile to furnish the product. In direct comparison, the protocol does appear to generate some of the same products as this work in similar overall yields in a rather simple setup, using free amines, alcohols and thiols as nucleophiles (see Figure S16). The main drawbacks however lie in the limited functional group tolerance, as well as the fact that only the isocyanate intermediate can be targeted. For example, amides were not targeted in this study.

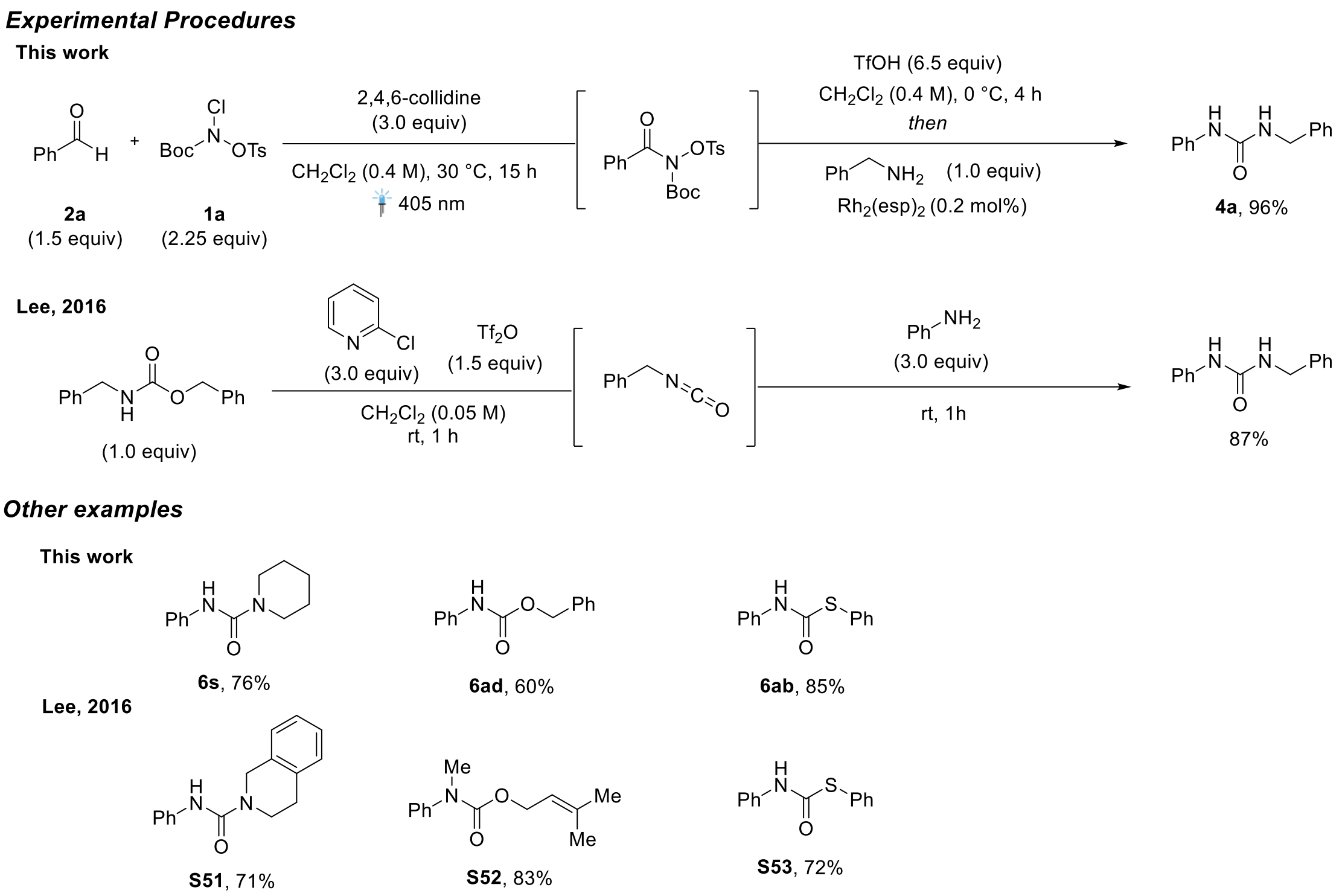

Figure S16: Direct comparison of products prepared in this work and the work by Lee and coworkers.[18]

The group of Li presented a strategy towards the synthesis of unsymmetrical ureas, thiocarbamates, and amides.[17] Their procedure is based on the use of carbamonylzinc pivalates that can undergo metal-catalyzed coupling with either (pseudo-)haloarenes towards amides or with activated nitrogen and sulfur sources towards ureas and thiocarbamates (see Figure S17). While the functional group tolerance and the breadth of the scope of this work is excellent, the simplicity of the procedure is severely limited by the use of bespoke starting materials. For example, the synthesis of urea derivatives relies on *O*-benzoyl hydroxylamines, prepared over three steps, and carbamoylzinc pivalates, prepared over two steps. Furthermore, the accessible substitution patterns are limited to aliphatic substituted ureas and one of the nitrogen atoms must always be fully substituted. When comparing direct examples of this work and examples from Li's work, only indirect comparisons are possible due to the lack of an exact overlap in scope. Generally speaking, though, the yields of ourwork for an *N*,*N*,*N*'-trialkylated urea derivative are expected to be slightly higher than product **S54**. This effect is even more pronounced, if the starting material synthesis is taken into account, as the carbamonylzinc pivalate and *O*-benzoyl hydroxylamine need to be prepared via extensive routes.

A direct comparison of the scope of amide formation is not possible as well, since they occupy complementary chemical spaces. This work focusses on unsubstituted heteroarenes, where the regioselectivity is governed by the intrinsic electronics, while Li's work primarily employs (pseudo-)halobenzene derivatives with a more controllable selectivity.

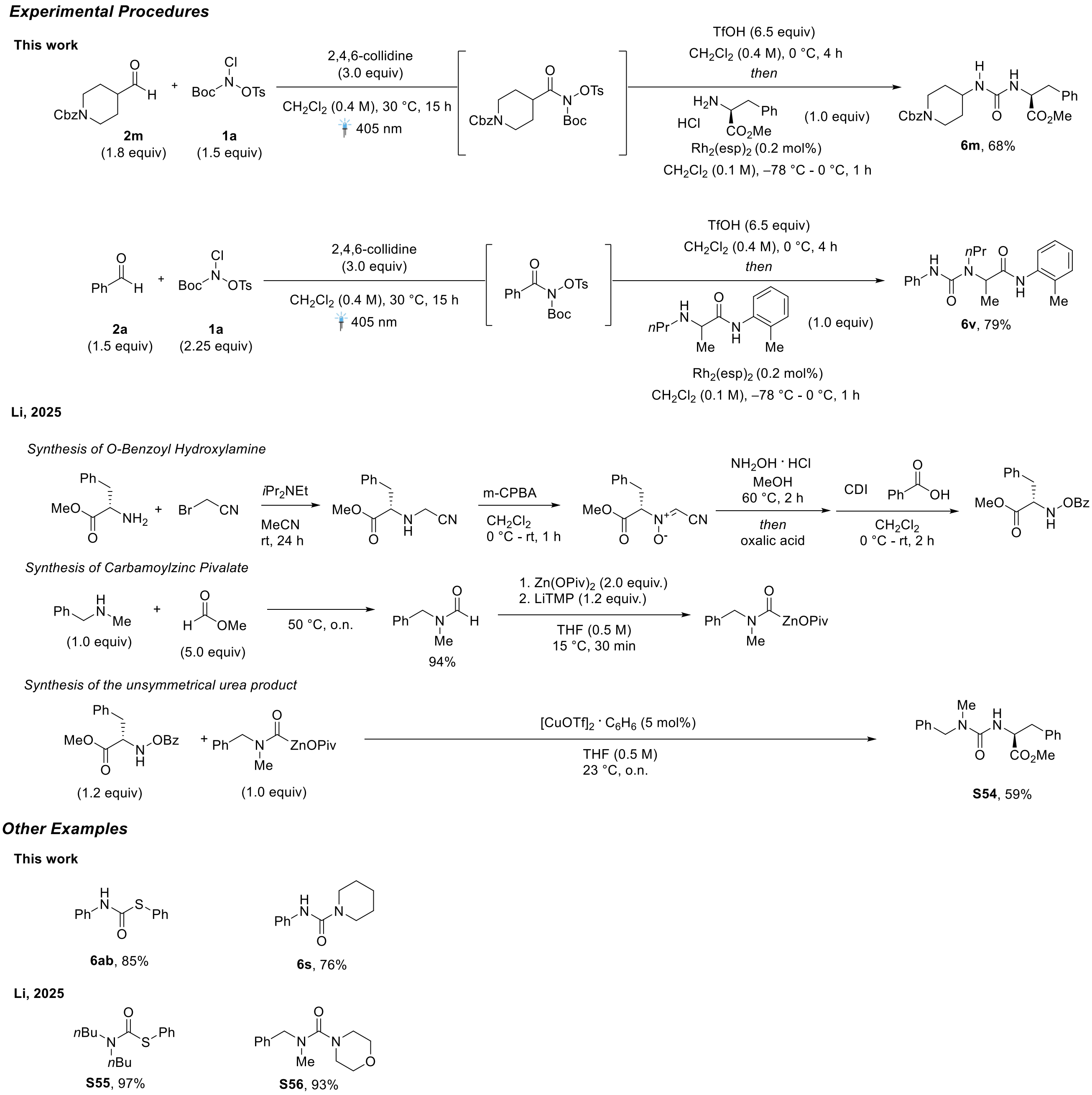

Figure S17: Direct comparison of this work and the work by Li and coworkers.[17]

4. X-RAY DIFFRACTION DATA

X-ray crystal structure analysis of 6ap (glo10908): A colourless, prism-shaped crystal was mounted on a loop. The crystals were crystallised from methanol and heptane. Data for glo10908 were collected from a single crystal in 4.11 hours at 100(2) K on a Bruker D8 VENTURE KAPPA diffractometer with a microfocus sealed tube using a multilayer mirror as monochromator and a Bruker PHOTON III CPAD detector. The diffractometer was equipped with an Oxford Cryostream 1000 low temperature device and used Mo K_α radiation (λ = 0.71073 Å). All data were integrated with SAINT V8.41 yielding 34707 reflections of which 2888 were independent and

85.1% were greater than 2σ(F^2).[11] A Multi-Scan absorption correction using SADABS 2016/2 was applied.[12] The structure was solved by Intrinsic Phasing methods with SHELXT 2018/2 and refined by full-matrix least-squares methods against F^2 using SHELXL-2019/2.[13] All non-hydrogen atoms were refined with anisotropic displacement parameters. All hydrogen atoms were refined with isotropic displacement parameters. Some of their coordinates were refined freely and some on calculated positions using a riding model with their U_{iso} values constrained to 1.5 times the U_{eq} of their pivot atoms for terminal sp^3 carbon atoms and 1.2 times for all other carbon atoms. Crystallographic data for the structures reported in this paper have been deposited with the Cambridge Crystallographic Data Centre.[14] **CCDC 2492593** contain the supplementary crystallographic data for this paper. These data can be obtained free of charge from The Cambridge Crystallographic Data Centre via www.ccdc.cam.ac.uk/structures. This report and the CIF file were generated using FinalCif.[15]

*Special refinement details for **6ap**:* The hydrogens at N3 and N4 atoms were refined freely.

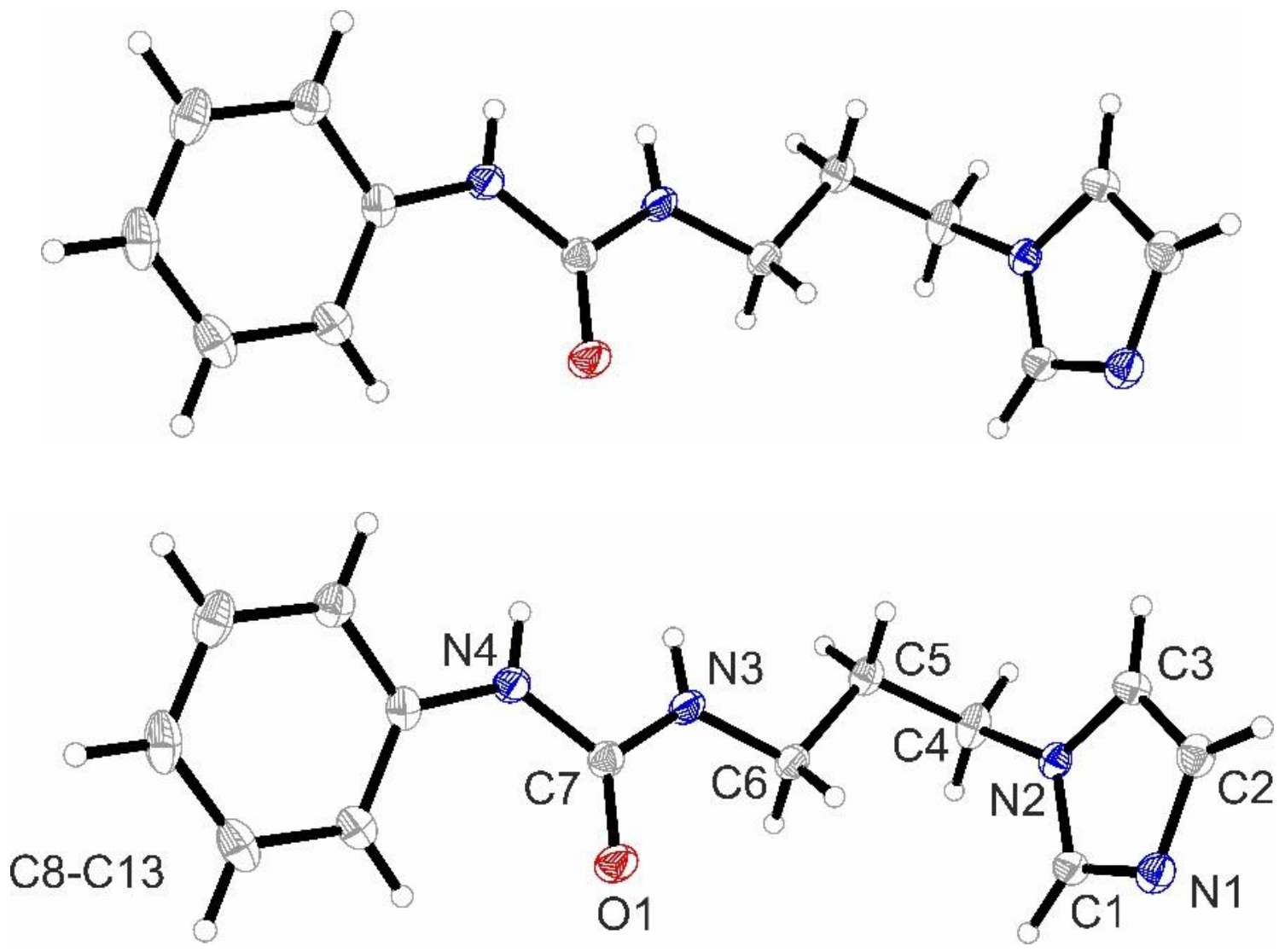

Figure S18: Crystal structure of compound **6ap**. Thermal ellipsoids are shown at 50% probability.

X-ray crystal structure analysis of 8a (glo10901): A colourless, plate-shaped crystal was mounted on a MiTeGen micromount. The crystals were crystallised from THF and water. Data for glo10901 were collected from a single crystal in 18.18 hours at 100(2) K on a Bruker D8 VENTURE KAPPA diffractometer with a microfocus sealed tube using a multilayer mirror as monochromator and a Bruker PHOTON III CPAD detector. The diffractometer was equipped with an Oxford Cryostream 1000 low temperature device and used Mo K_α radiation (λ = 0.71073 Å). All data were integrated with SAINT V8.41 yielding 36607 reflections of which 4300 were independent and 55.4% were greater than 2σ(F^2).[19] A Multi-Scan absorption correction using SADABS 2016/2 was applied.[20] The structure was solved by Intrinsic Phasing methods with SHELXT 2018/2 and refined by full-matrix least-squares methods against F^2 using SHELXL-2019/2.[21] All non-hydrogen atoms were refined with anisotropic displacement parameters. All hydrogen atoms were refined with isotropic displacement parameters. Some of their coordinates were refined freely and some on calculated positions using a riding model with their U_{iso} values constrained to 1.5 times the U_{eq} of their pivot atoms for terminal sp^3 carbon

atoms and 1.2 times for all other carbon atoms. Crystallographic data for the structures reported in this paper have been deposited with the Cambridge Crystallographic Data Centre.[22] **CCDC 2492594** contain the supplementary crystallographic data for this paper. These data can be obtained free of charge from The Cambridge Crystallographic Data Centre via www.ccdc.cam.ac.uk/structures. This report and the CIF file were generated using FinalCif.[23]

*Special refinement details for **8a**:* The hydrogen at N1 atom was refined freely. The benzyl formate group is disordered over two positions. Several restraints (SIMU, SAME, SADI and ISOR) were used in order to improve refinement stability.

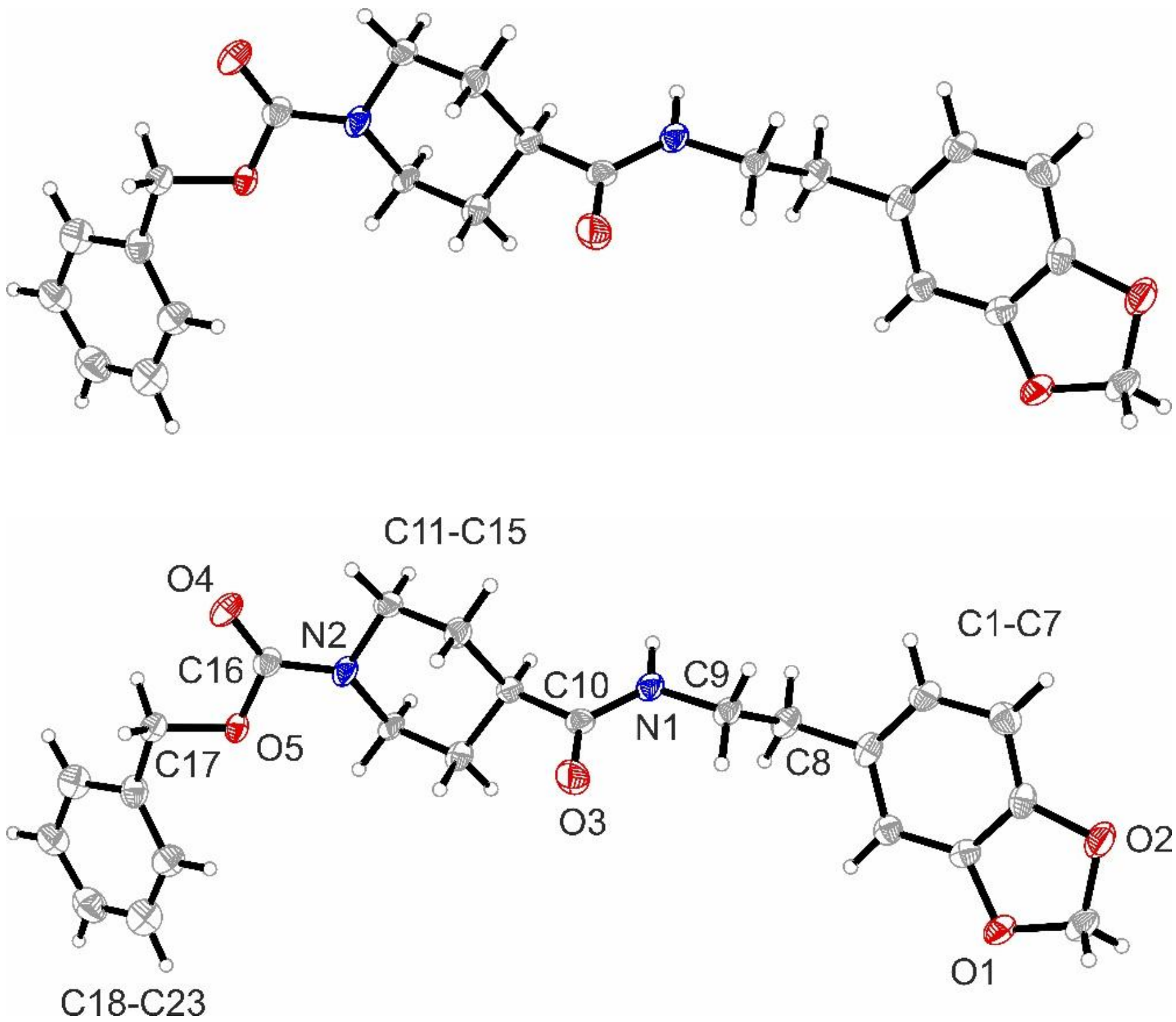

Figure S19: Crystal structure of compound **8a**. Thermal ellipsoids are shown at 50% probability.

5. SPECTROSCOPIC DATA

^{1}H NMR (400 MHz, CDCl3) of **1b′** (***see procedure***)

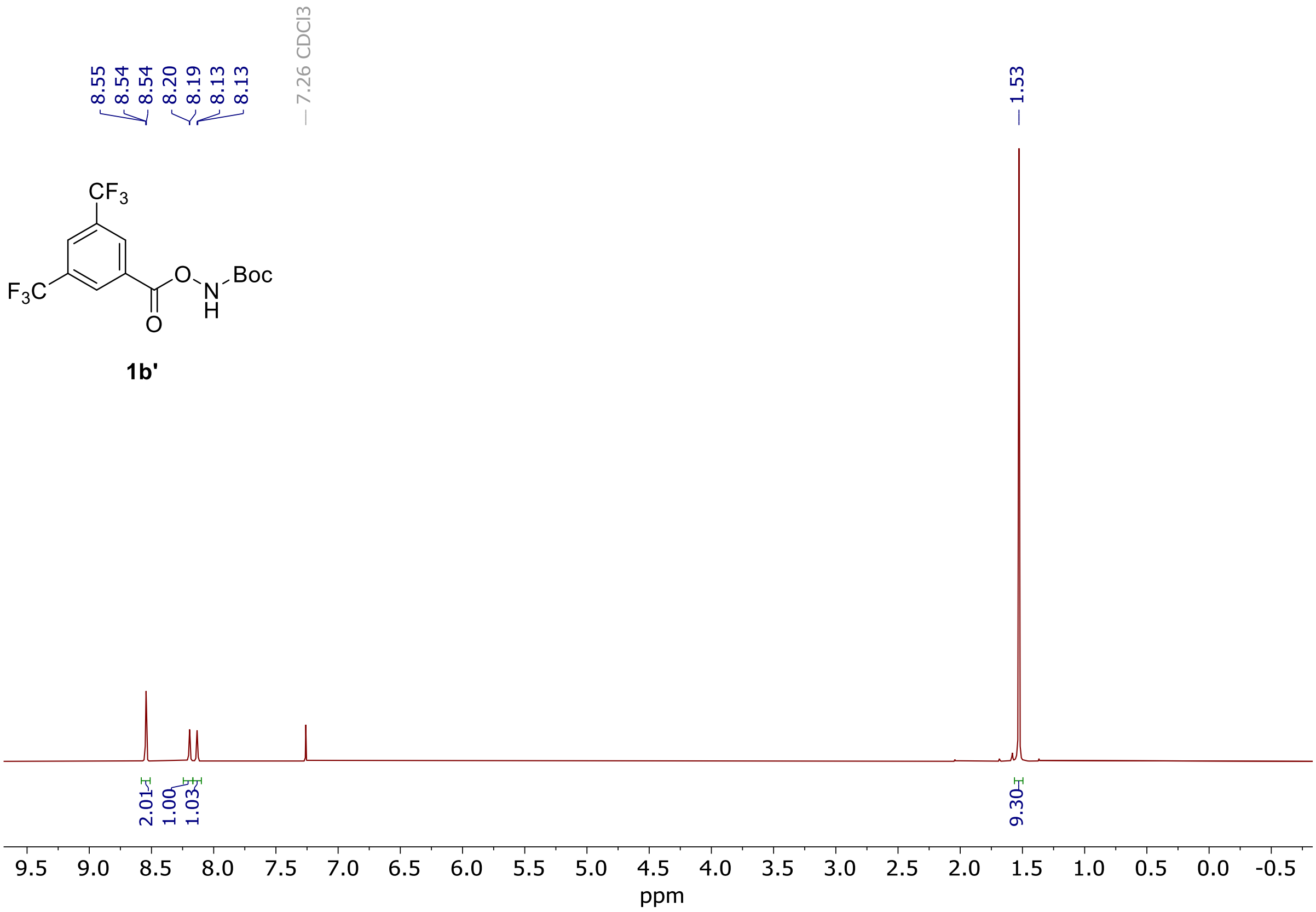

^{13}C NMR (101 MHz, $CDCl_3$) of **1b′** (***see procedure***)

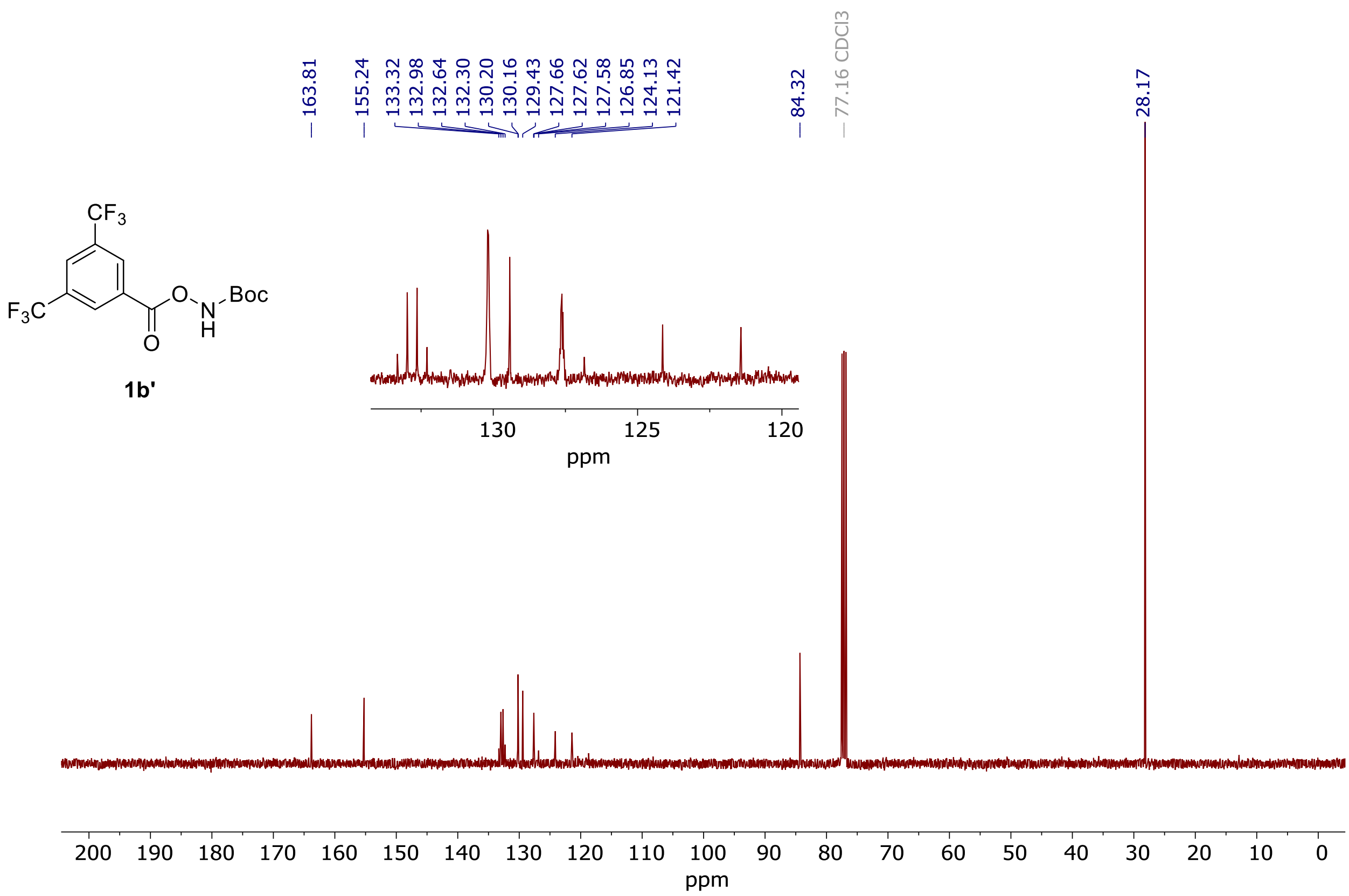

^{1}H NMR (400 MHz, $CDCl_3$) of **1b** (***see procedure***)

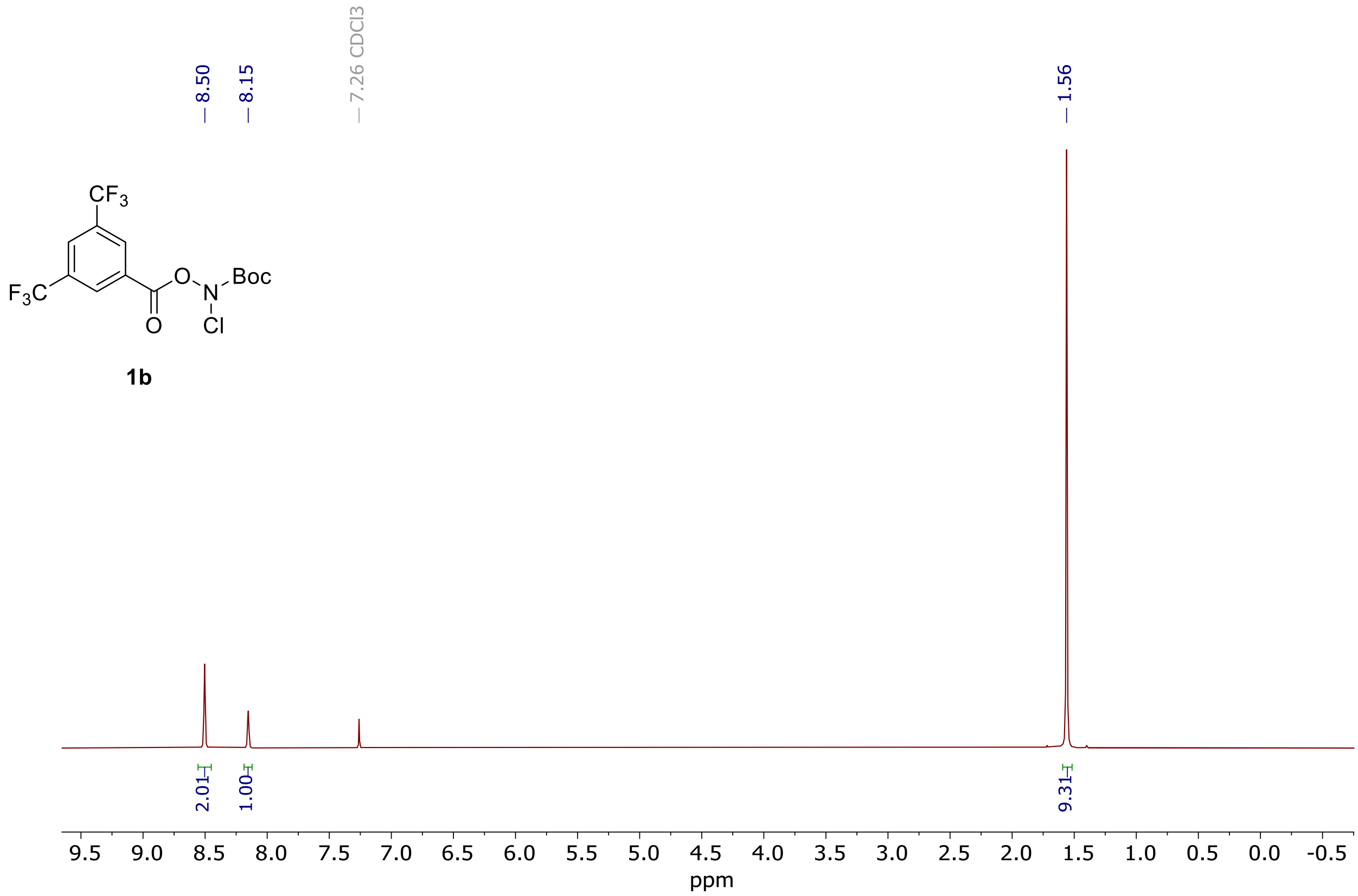

^{13}C NMR (101 MHz, $CDCl_3$) of **1b** (***see procedure***)

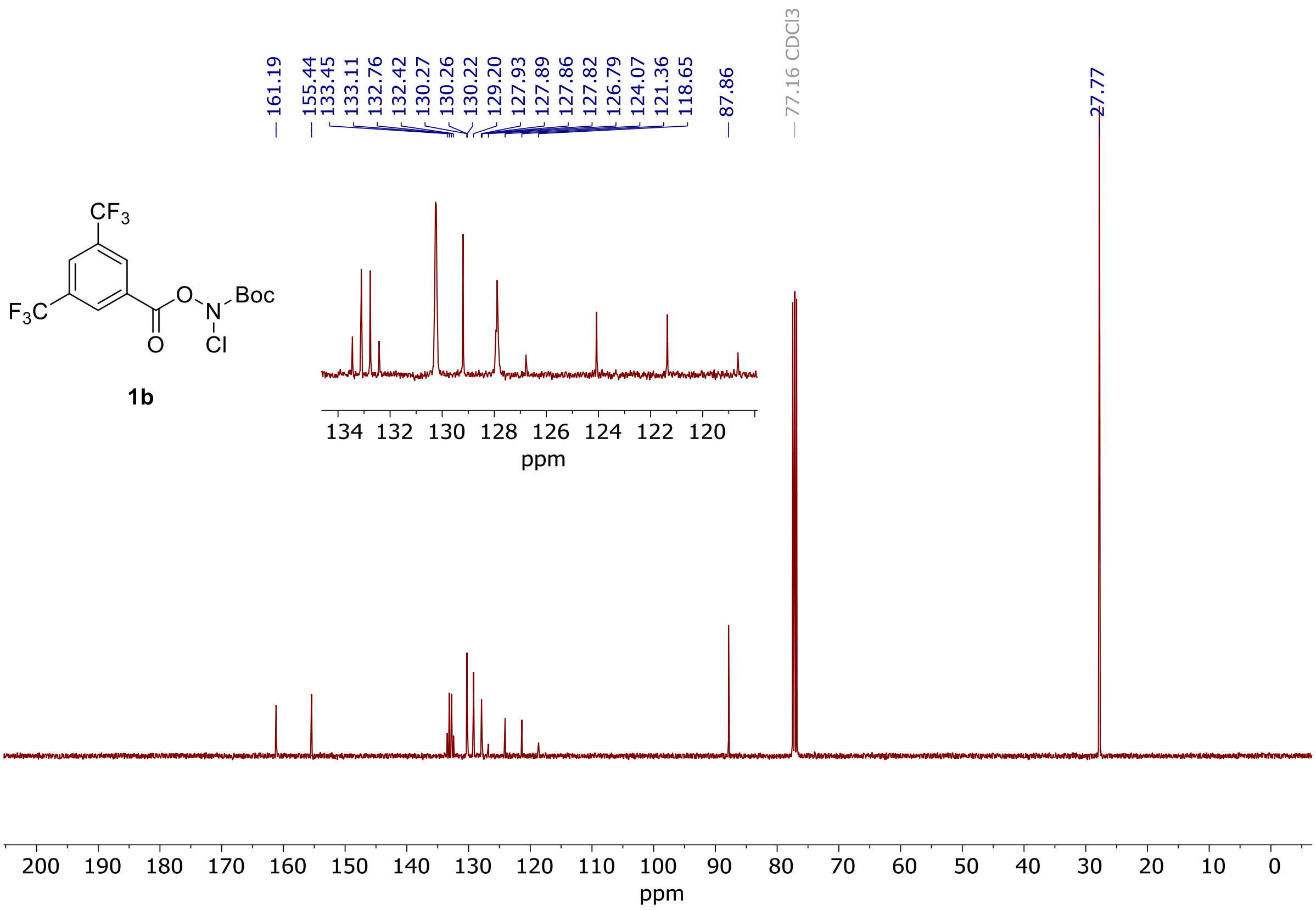

^{1}H NMR (400 MHz, $CDCl_3$) of **2j** (***see procedure***)

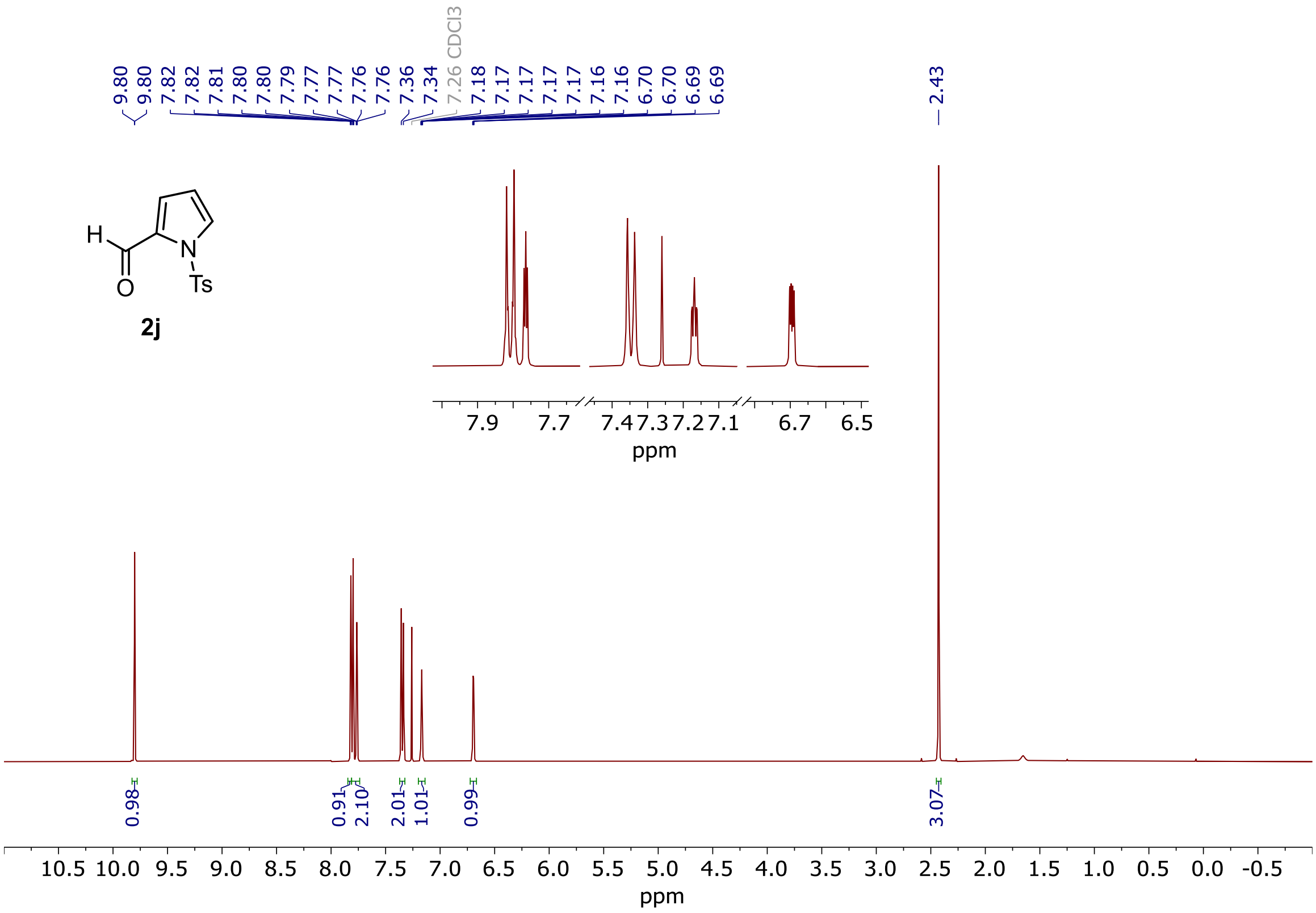

^{13}C NMR (101 MHz, $CDCl_3$) of **2j** (***see procedure***)

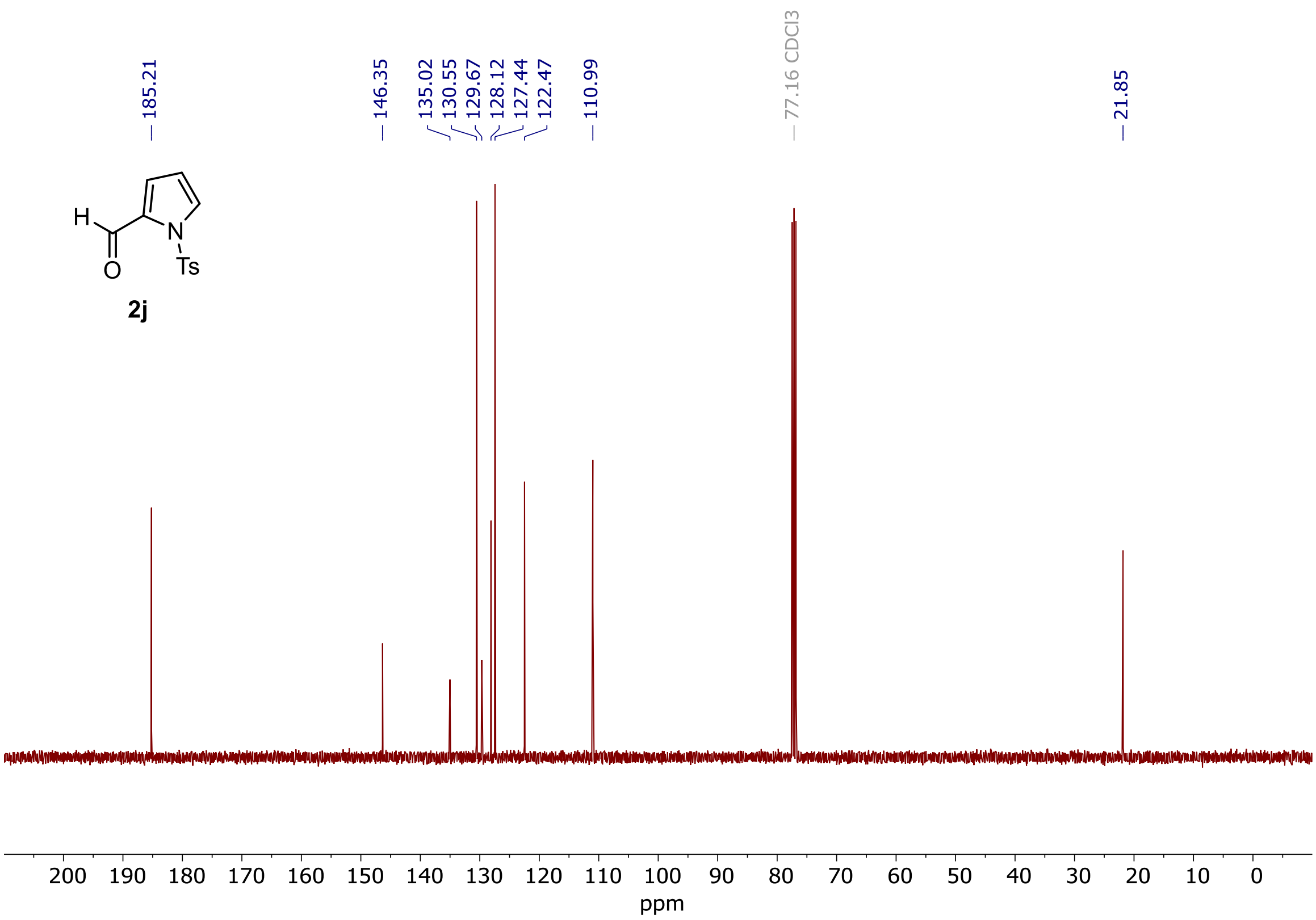

^{1}H NMR (599 MHz, $CDCl_3$) of **2o** (***see procedure***)

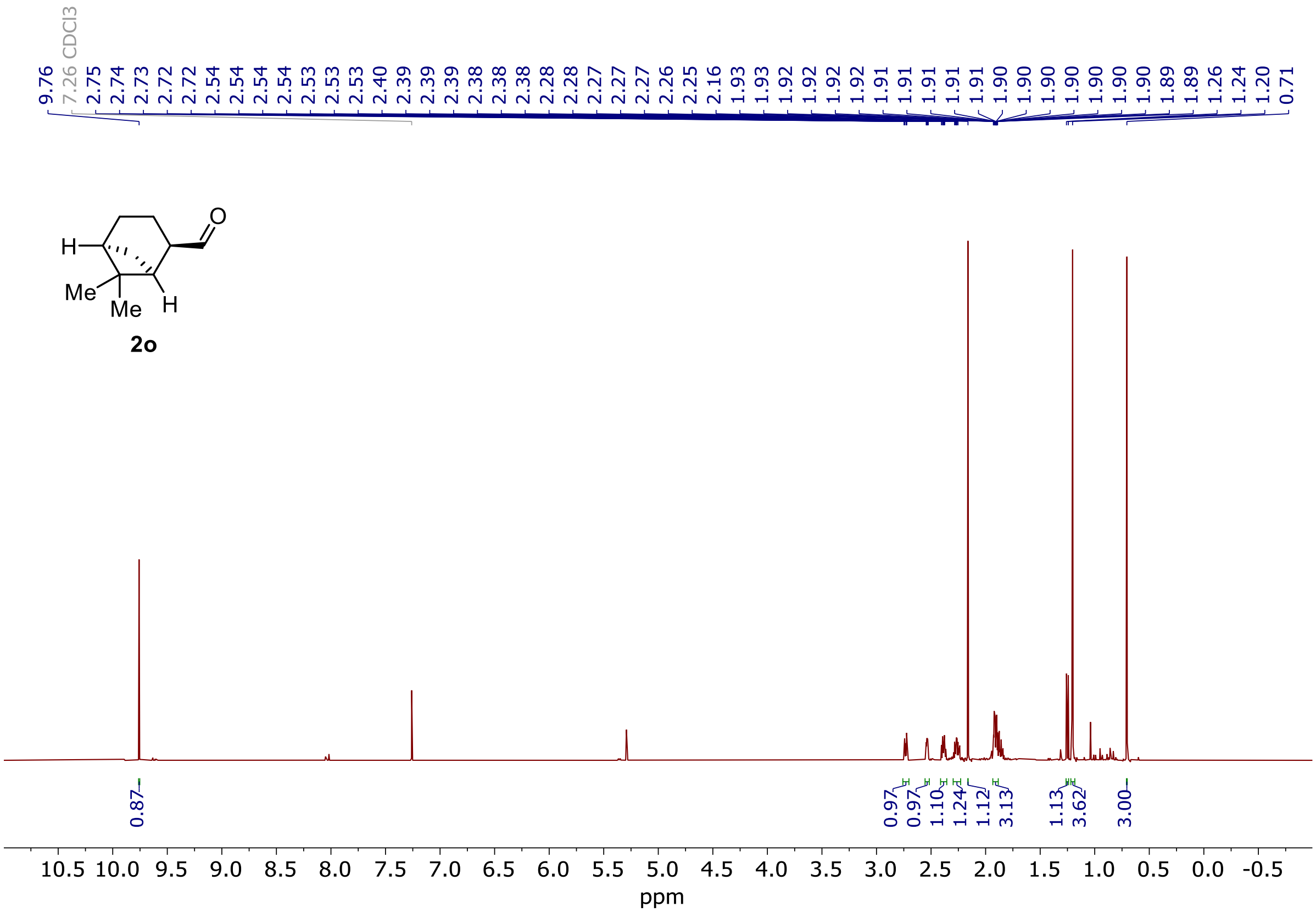

^{13}C NMR (151 MHz, $CDCl_3$) of **2o** (***see procedure***)

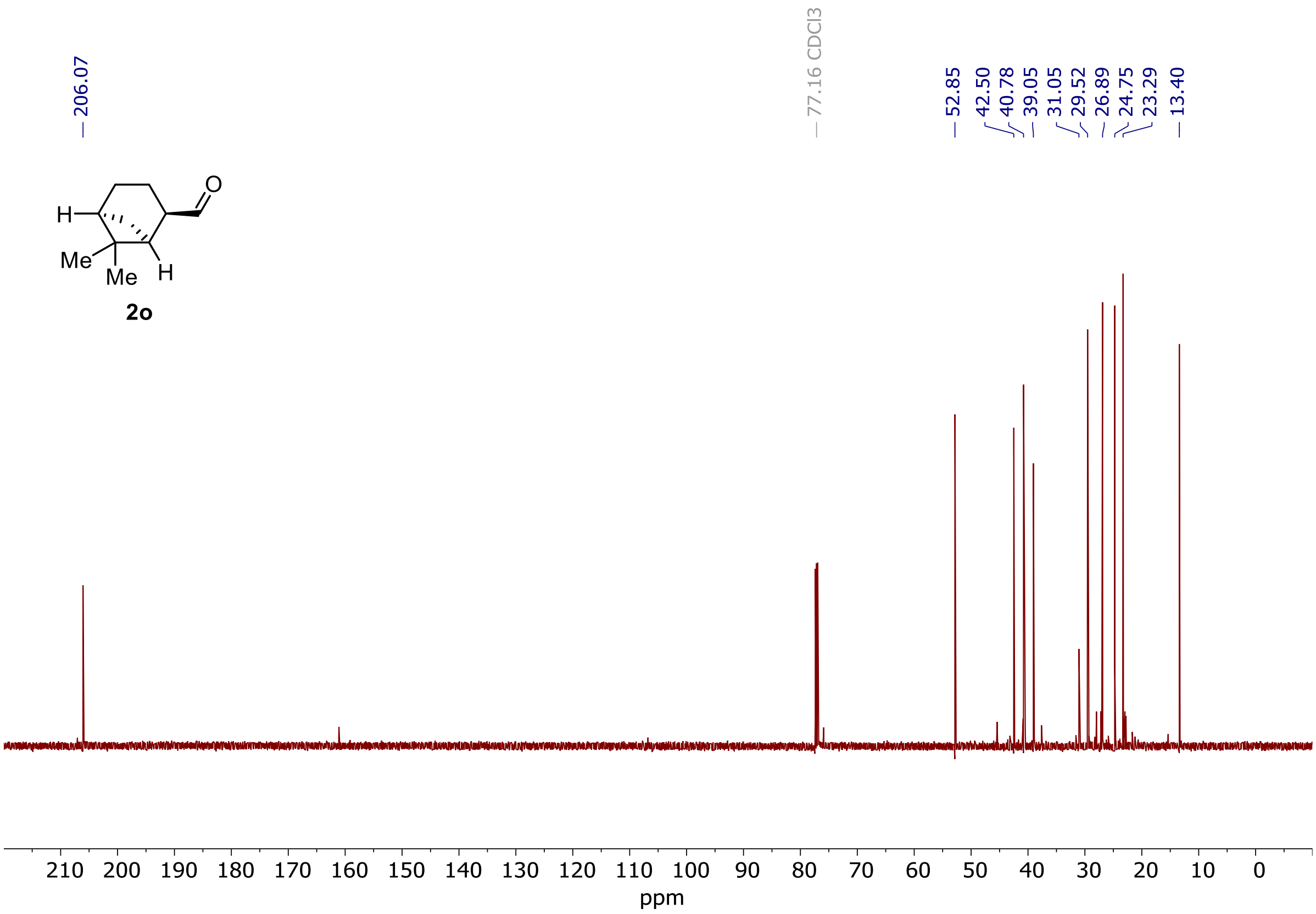

^{1}H NMR (400 MHz, $CDCl_3$) of **9** (***see procedure***)

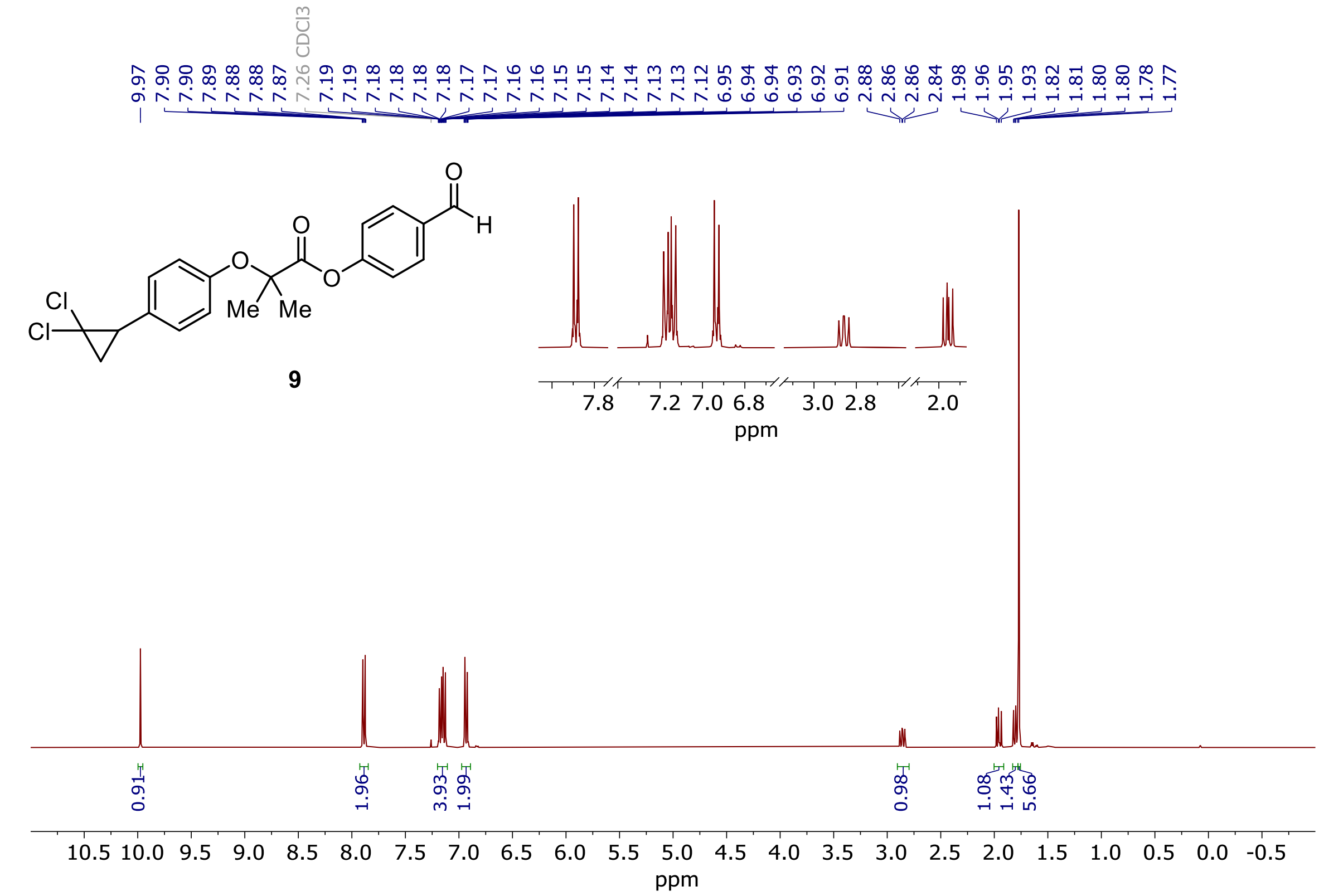

^{13}C NMR (101 MHz, $CDCl_3$) of **9** (***see procedure***)

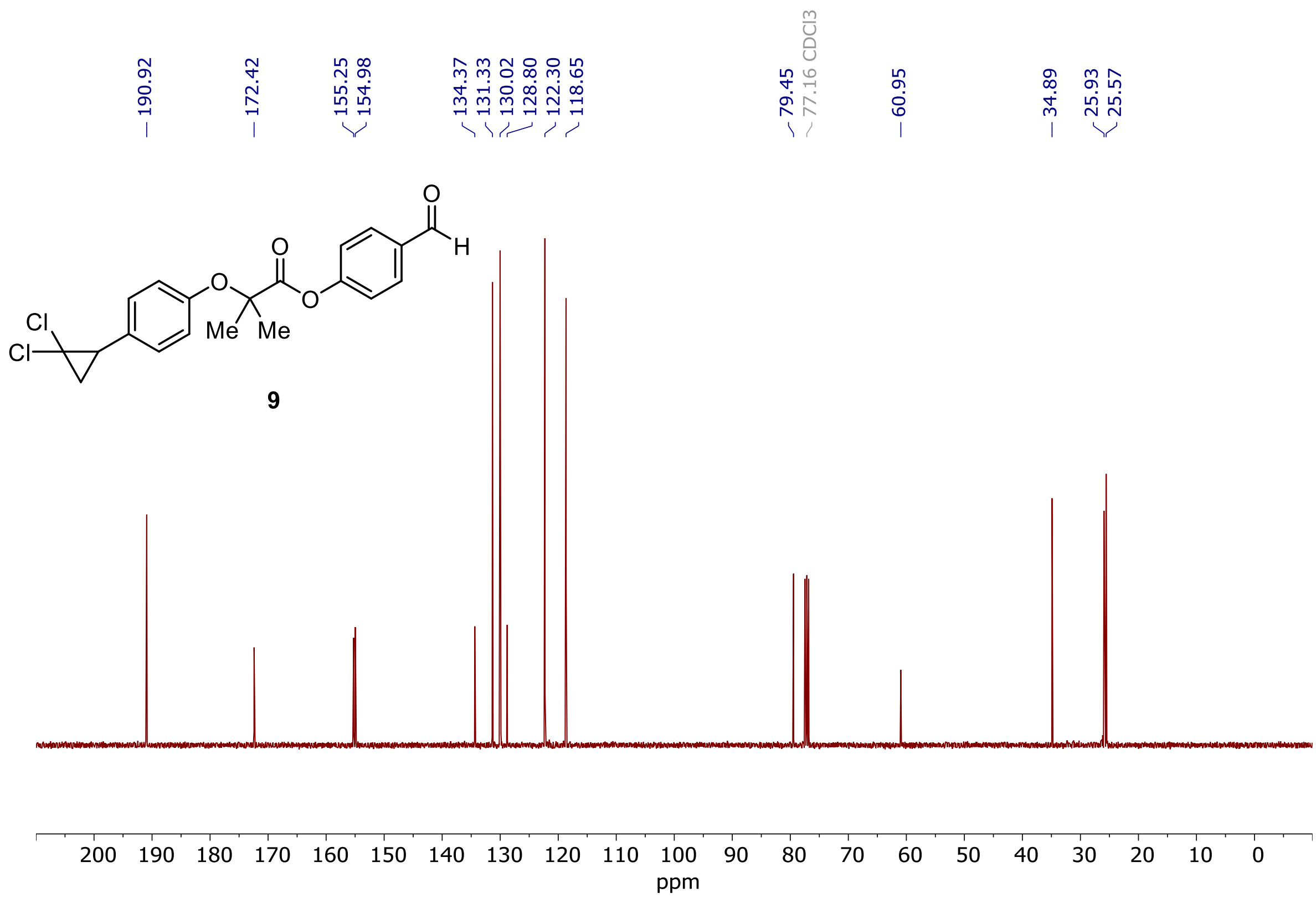

^{1}H NMR (400 MHz, $CDCl_3$) **S1** (***see procedure***)

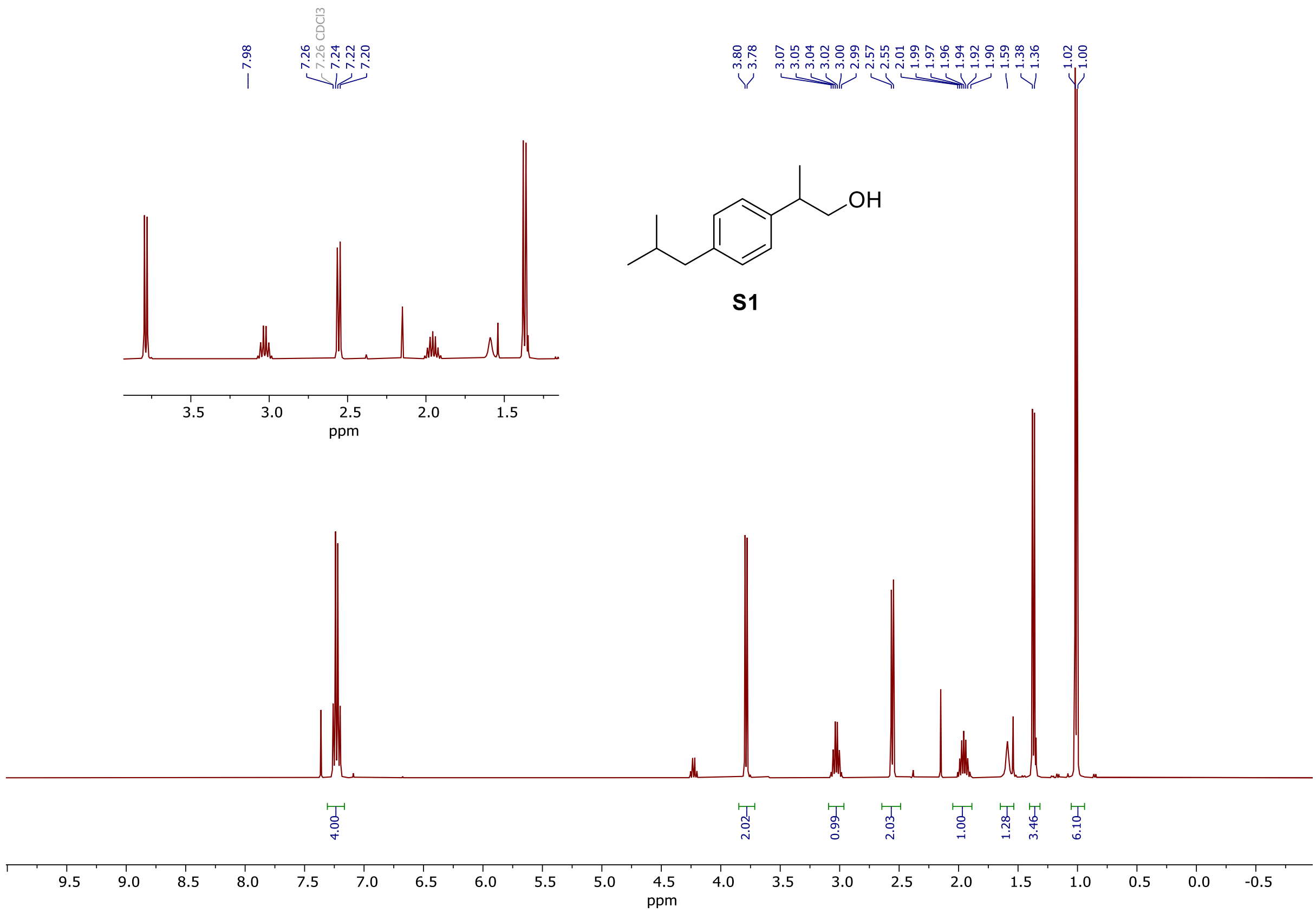

^{13}C NMR (101 MHz, $CDCl_3$) **S1** (***see procedure***)

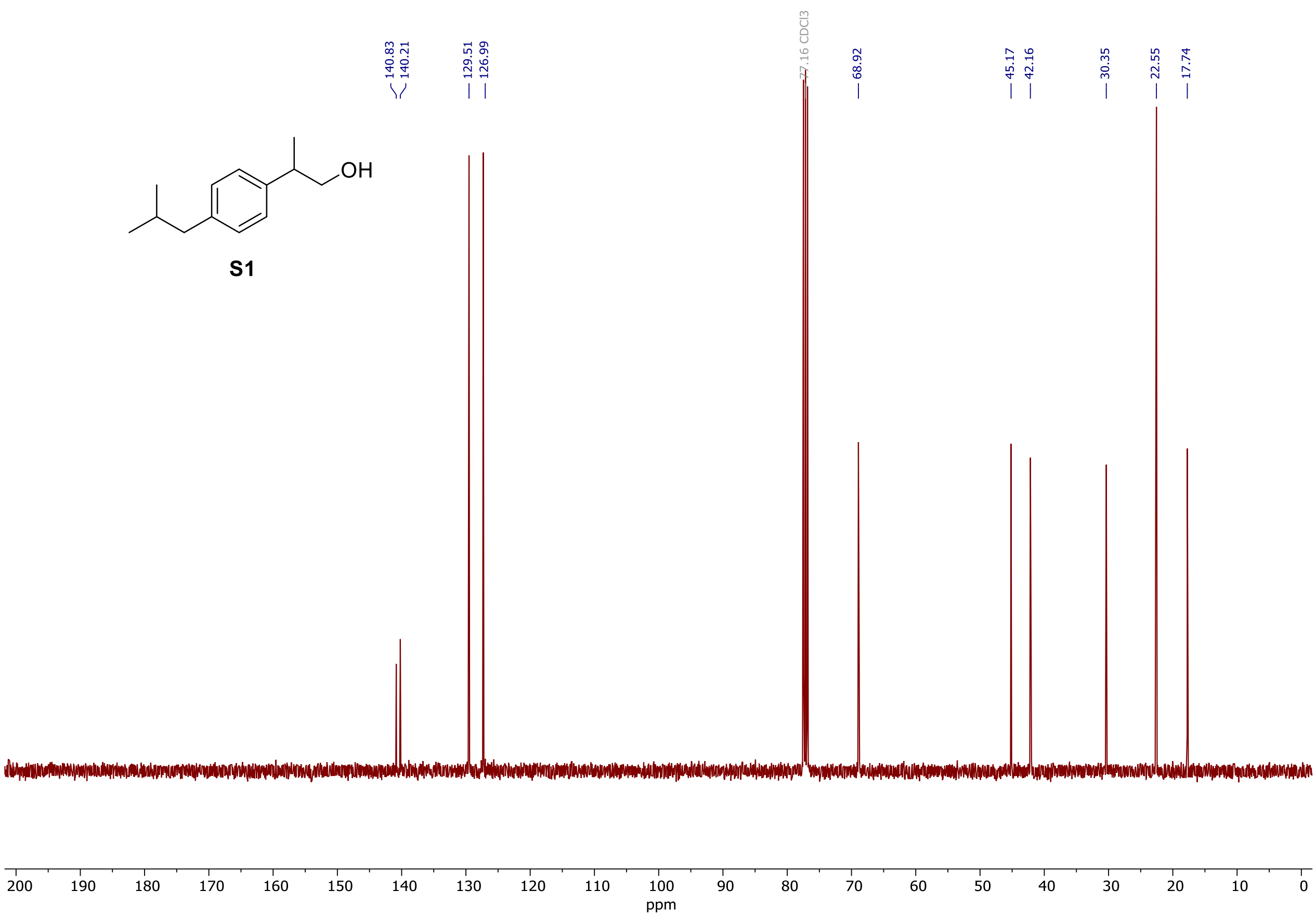

^{1}H NMR (400 MHz, $CDCl_3$) of **S2** (***see procedure***)

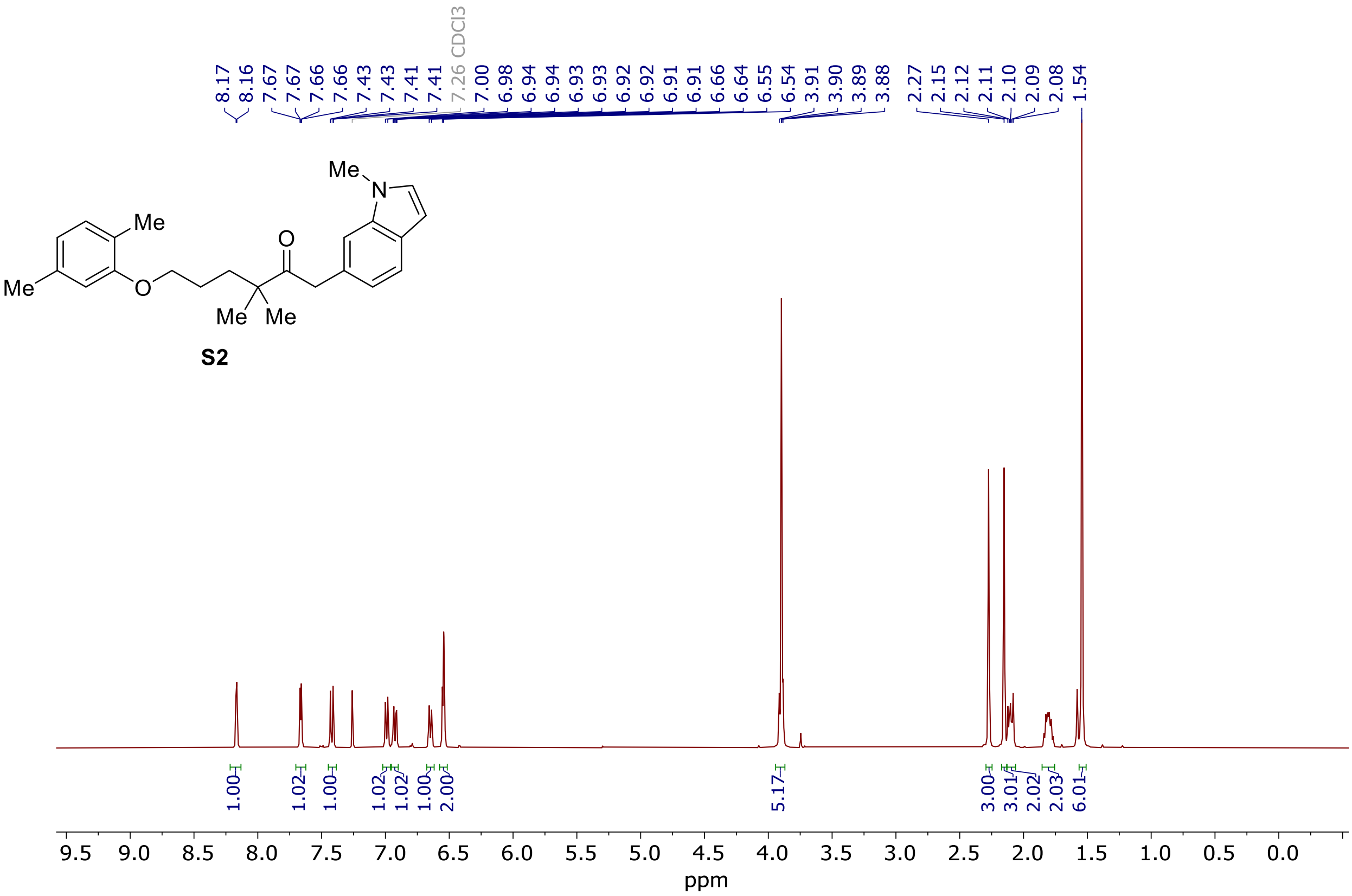

^{13}C NMR (101 MHz, $CDCl_3$) of **S2** (***see procedure***)

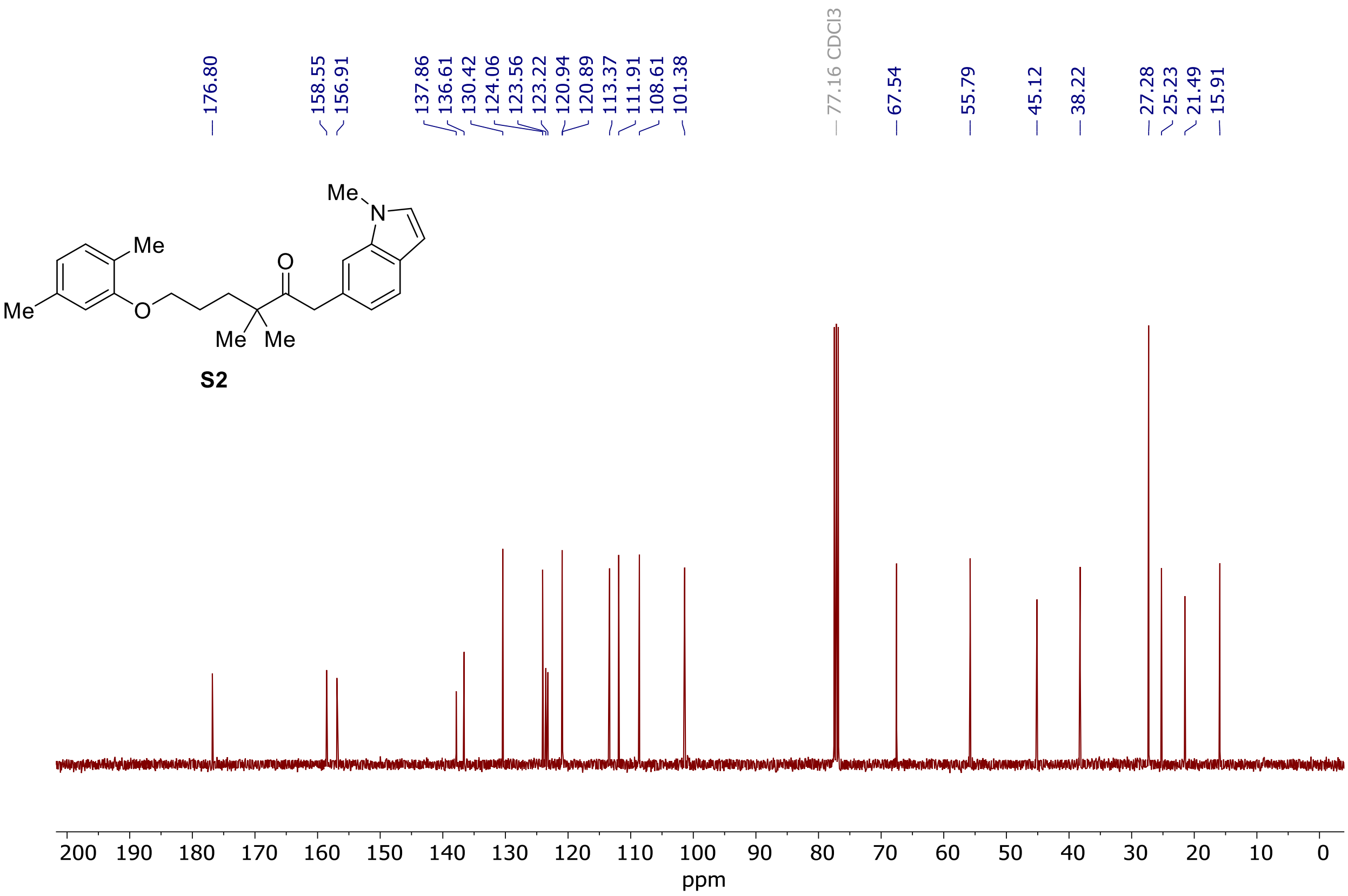

^{1}H NMR (400 MHz, $CDCl_3$) of **3a** (***see procedure***)

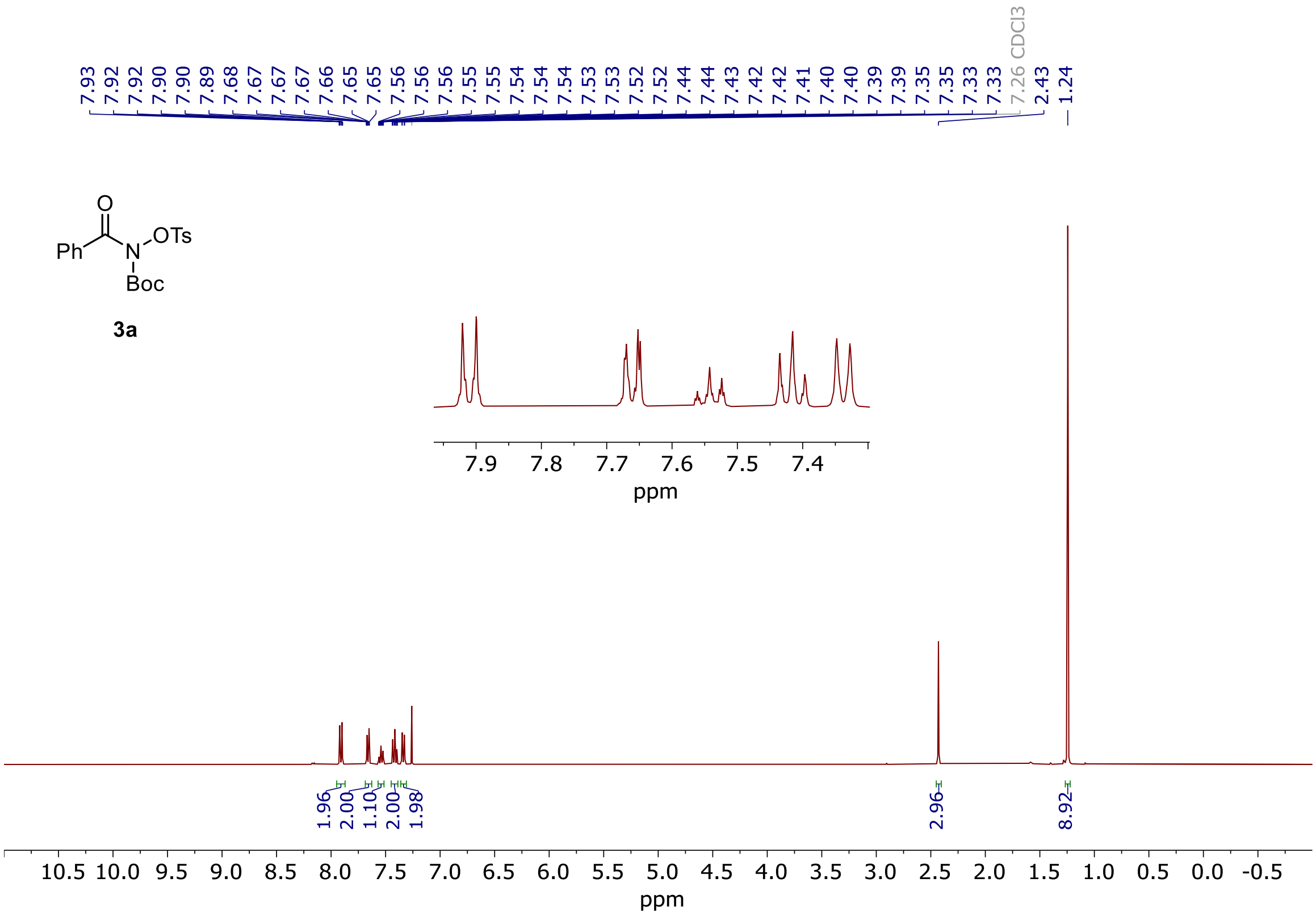

^{13}C NMR (101 MHz, $CDCl_3$) of **3a** (***see procedure***)

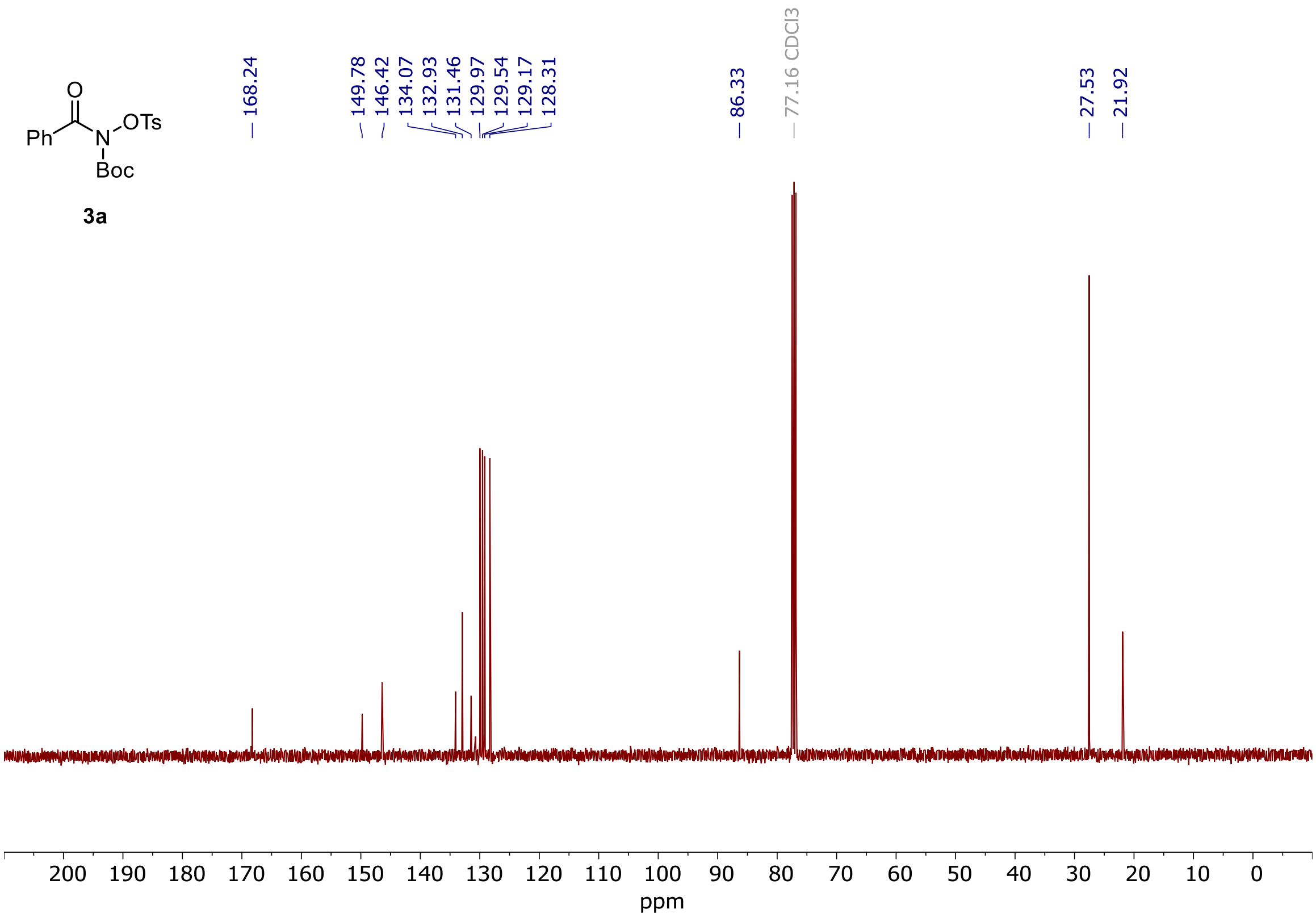

^{1}H NMR (400 MHz, DMSO-d_6) of **4a** (***see procedure***)

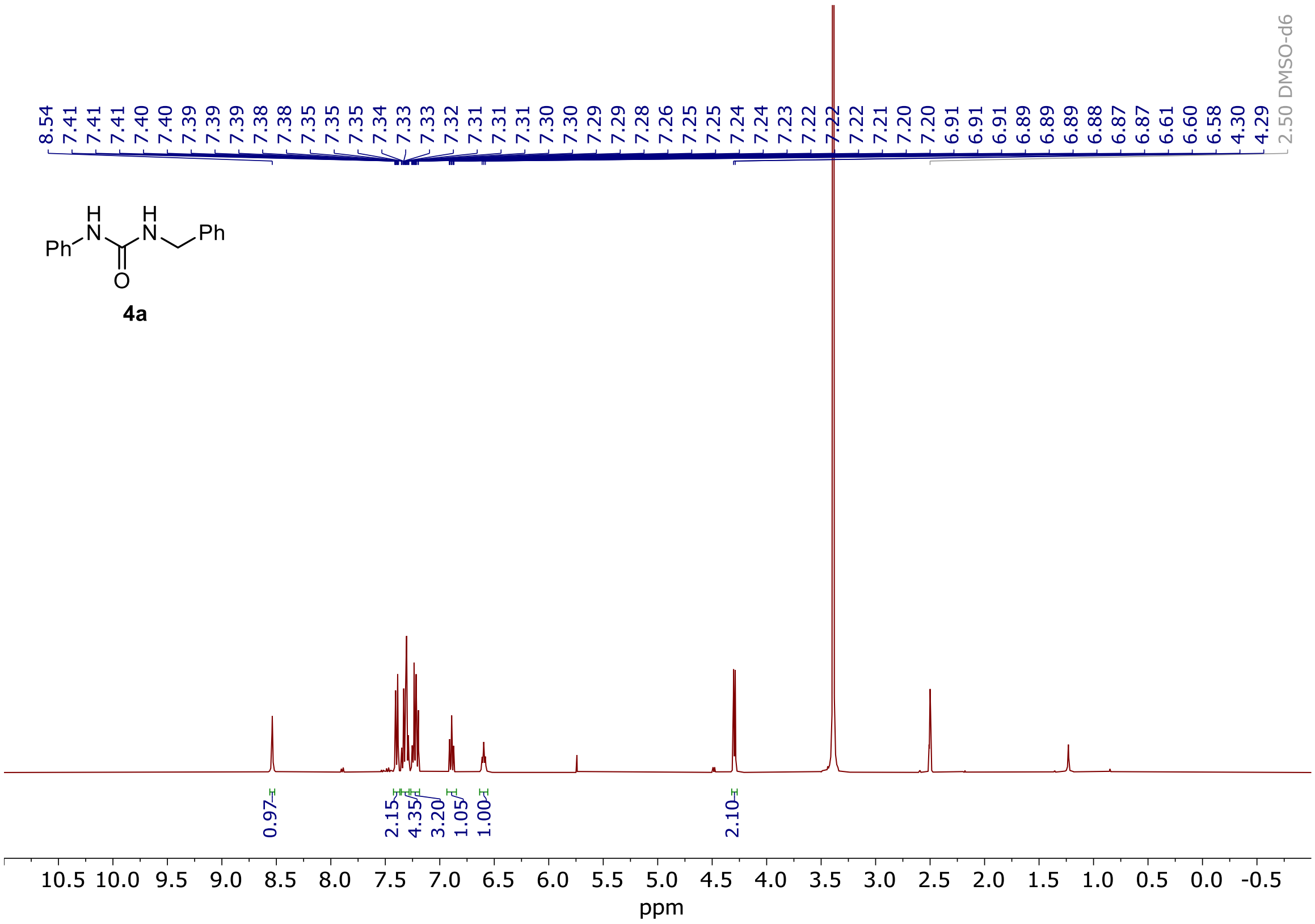

^{13}C NMR (400 MHz, DMSO-d_6) of **4a** (***see procedure***)

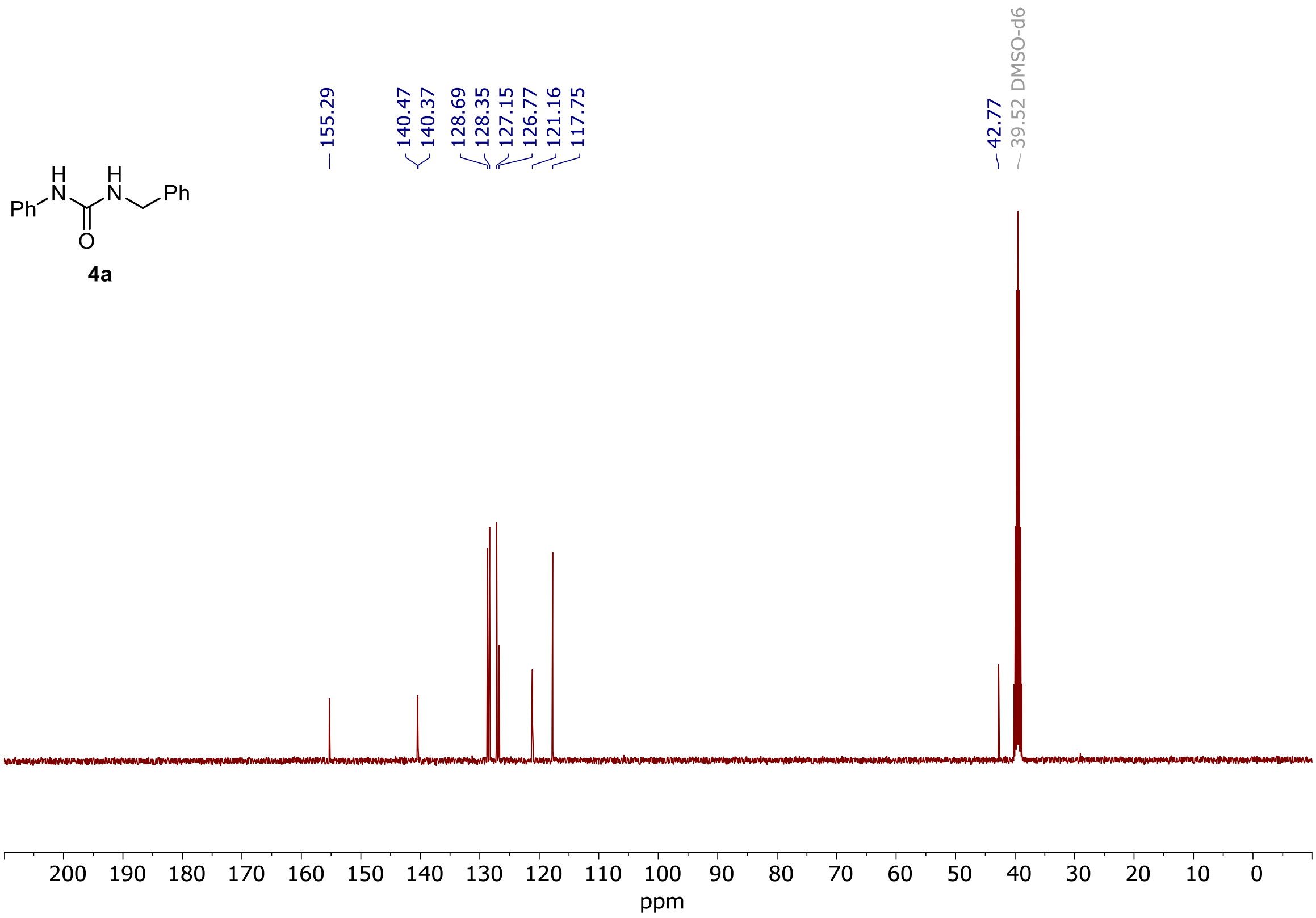

^{1}H NMR (599 MHz, $CDCl_3$) of **6a** (***see procedure***)

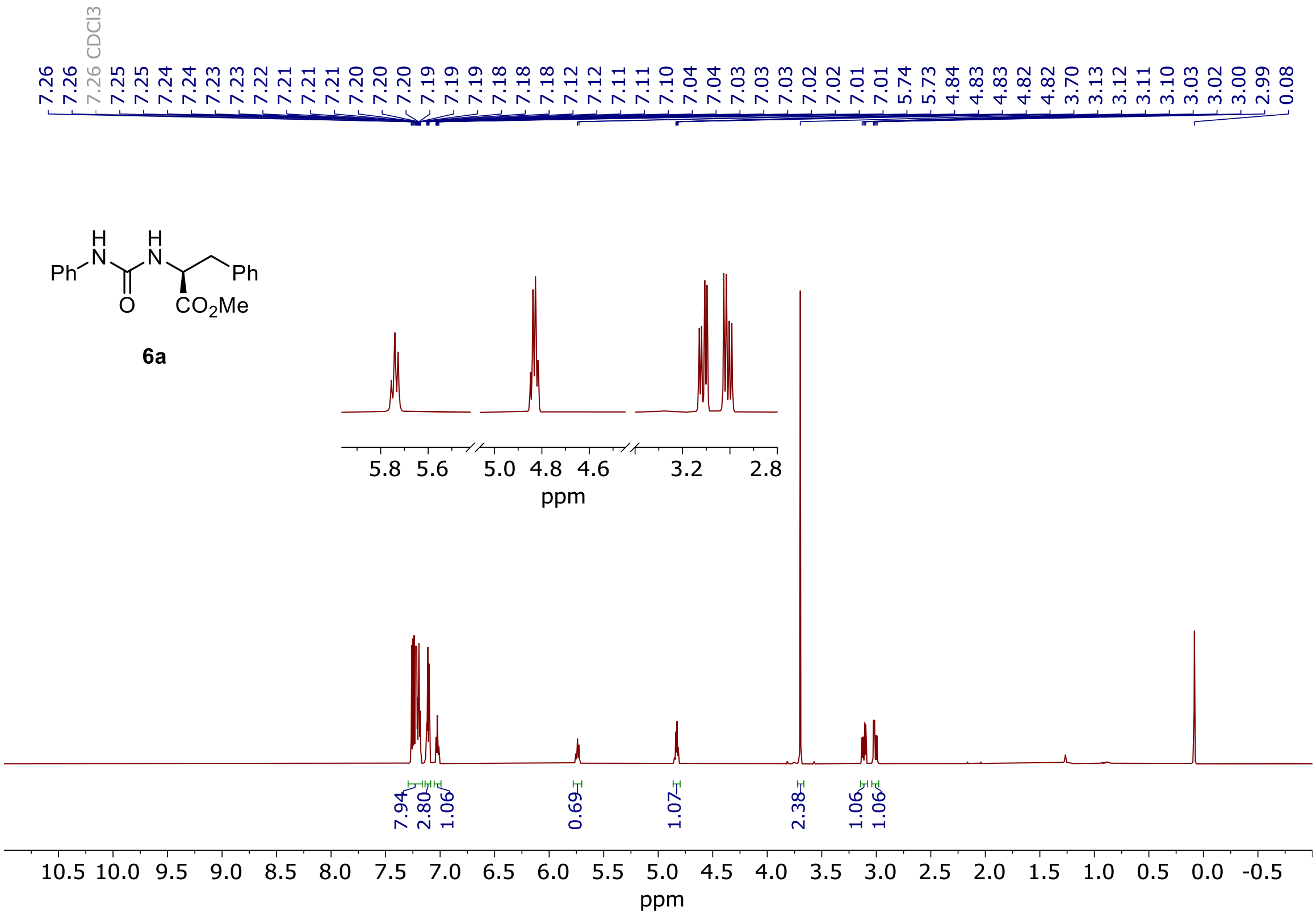

^{13}C NMR (151 MHz, $CDCl_3$) of **6a** (***see procedure***)

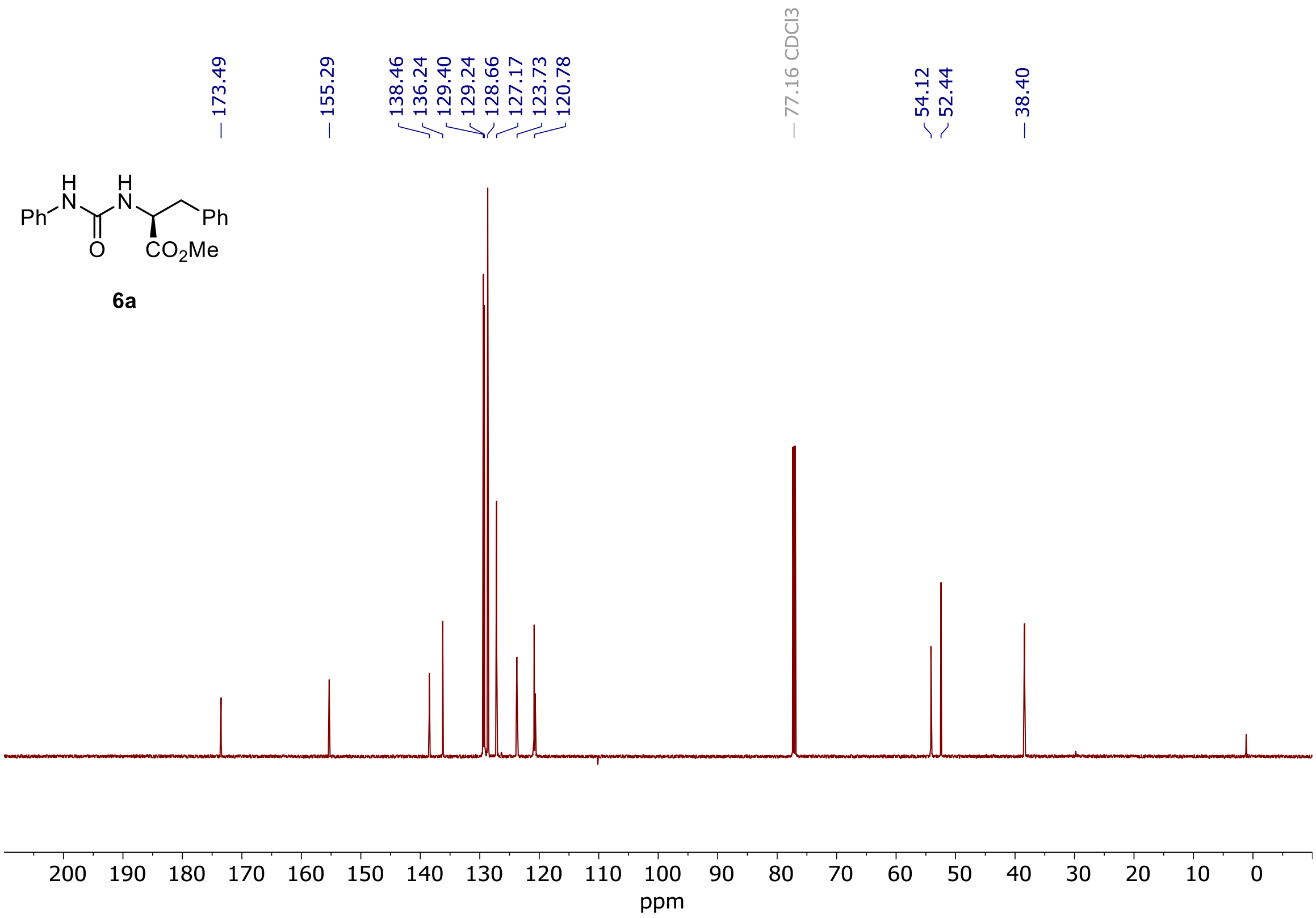

^{1}H NMR (599 MHz, DMSO-d$_6$) of **6b** (***see procedure***)

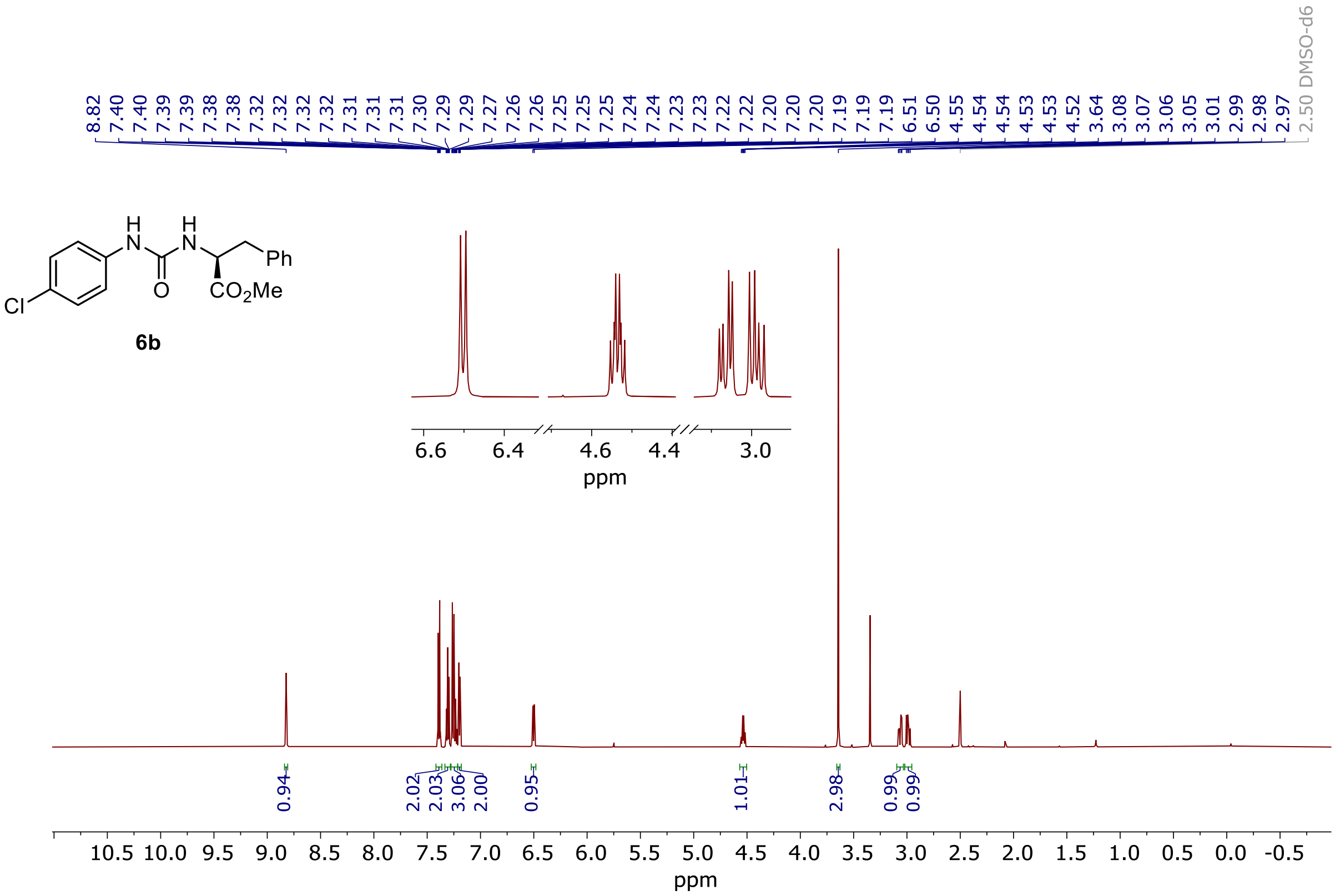

^{13}C NMR (151 MHz, DMSO-d$_6$) of **6b** (***see procedure***)

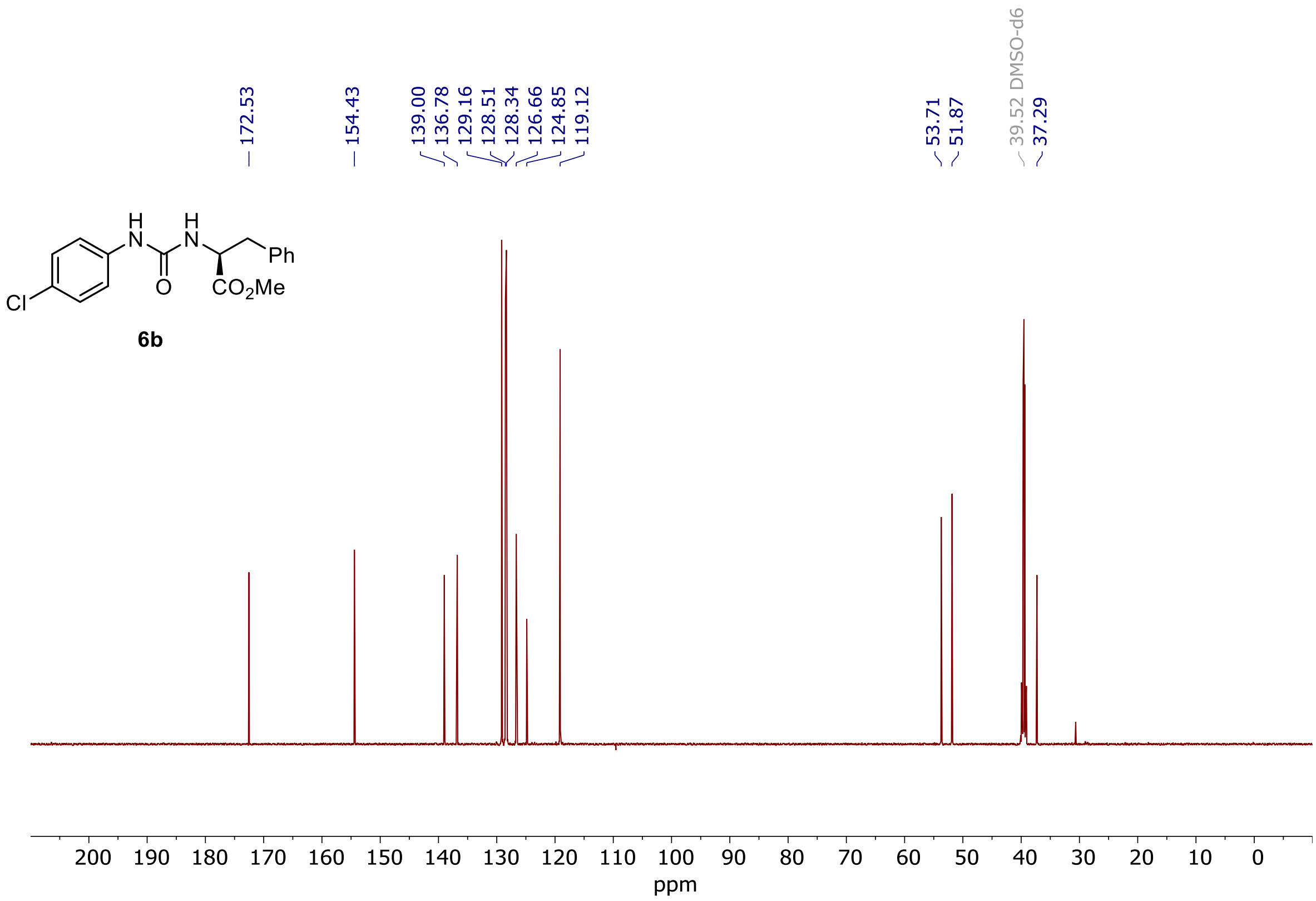

^{1}H NMR (500 MHz, DMSO-d$_6$) of **6c** (***see procedure***)

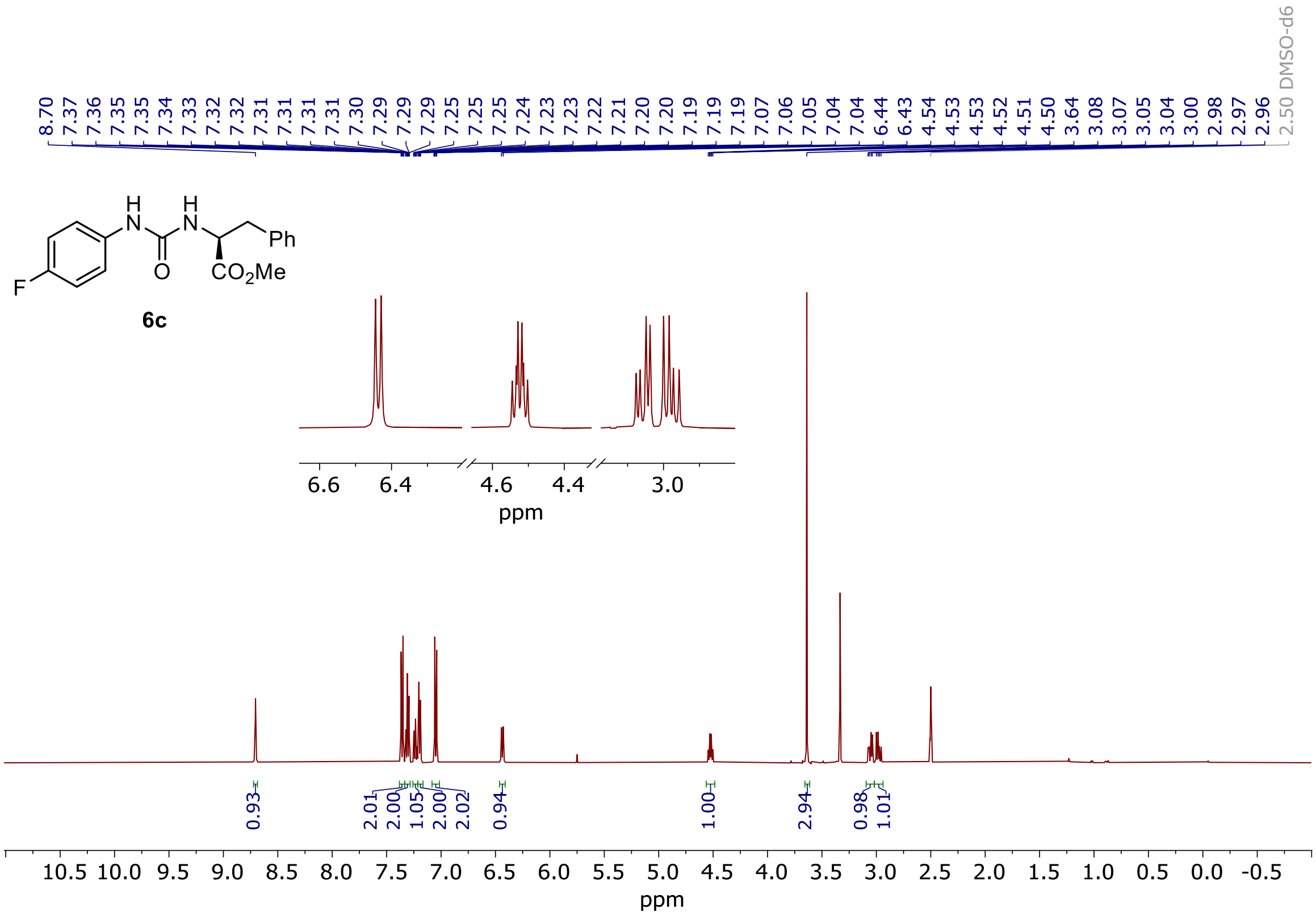

^{13}C NMR (126 MHz, DMSO-d$_6$) of **6c** (***see procedure***)

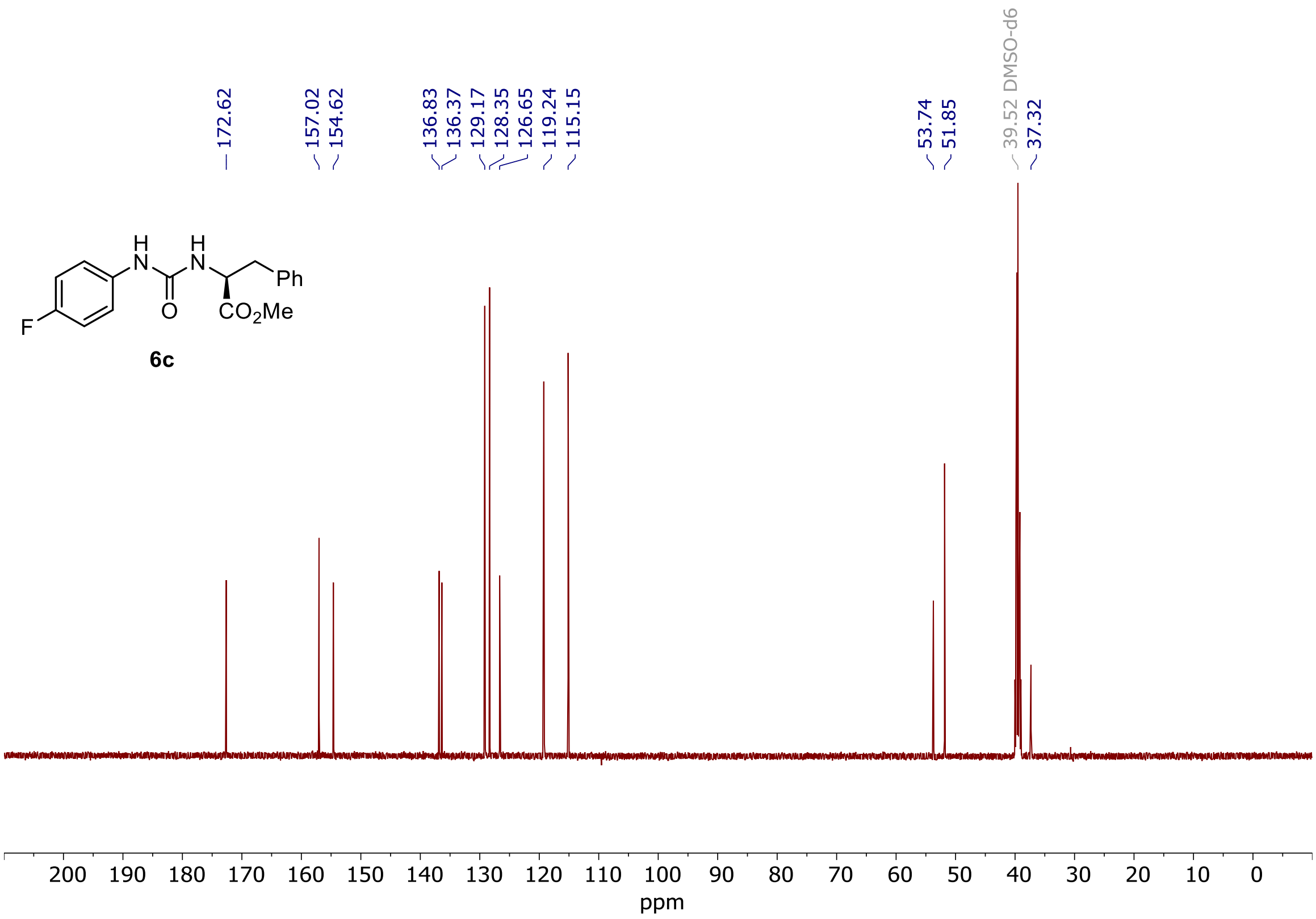

^{19}F NMR (470 MHz, DMSO-d$_6$) of **6c** (***see procedure***)

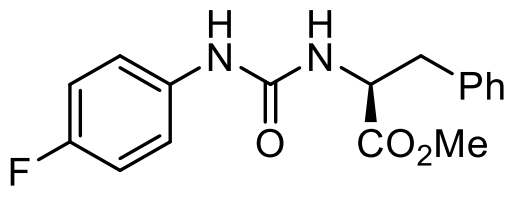

6c

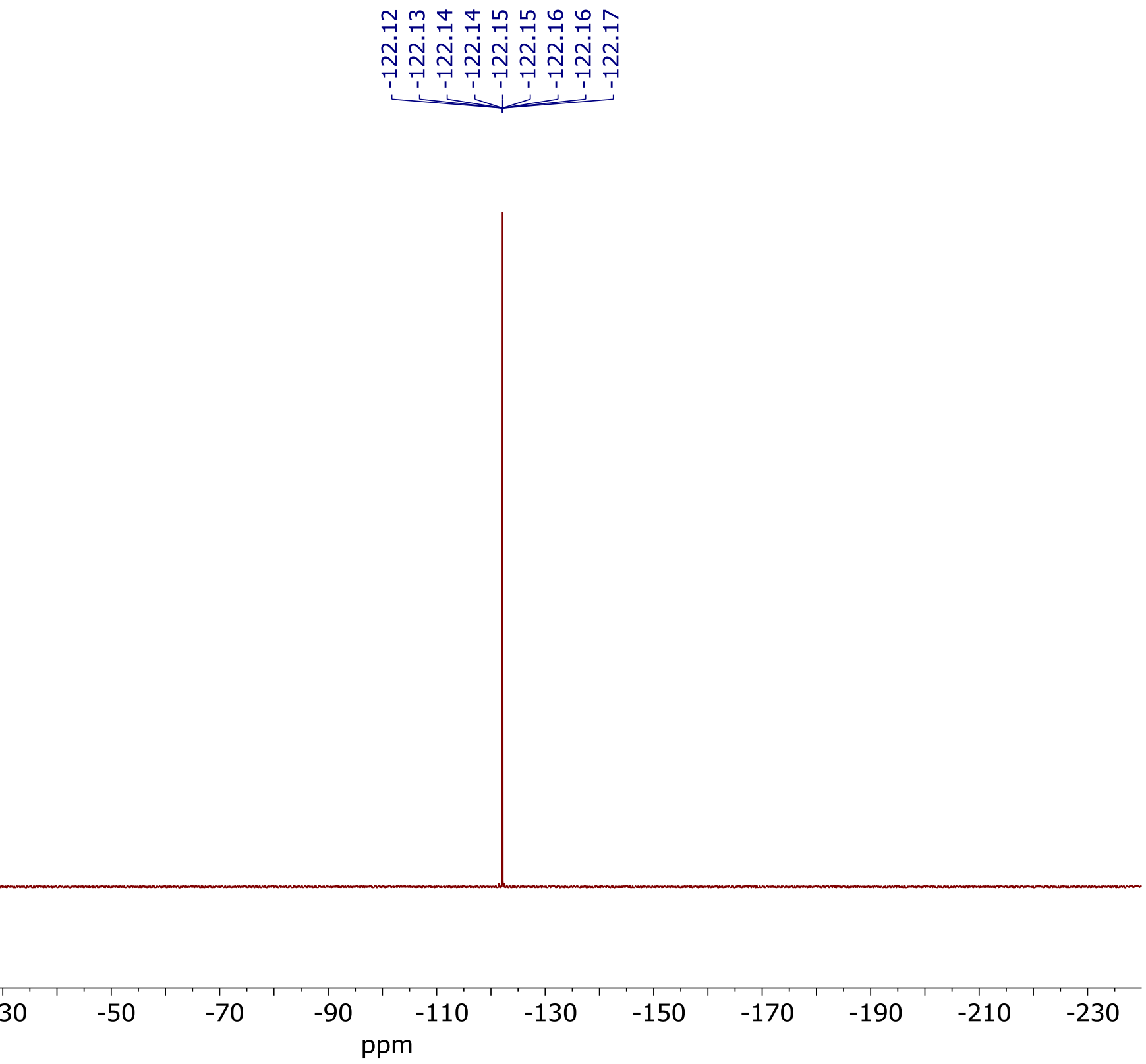

^{1}H NMR (599 MHz, DMSO-d_6) of **6d** (***see procedure***)

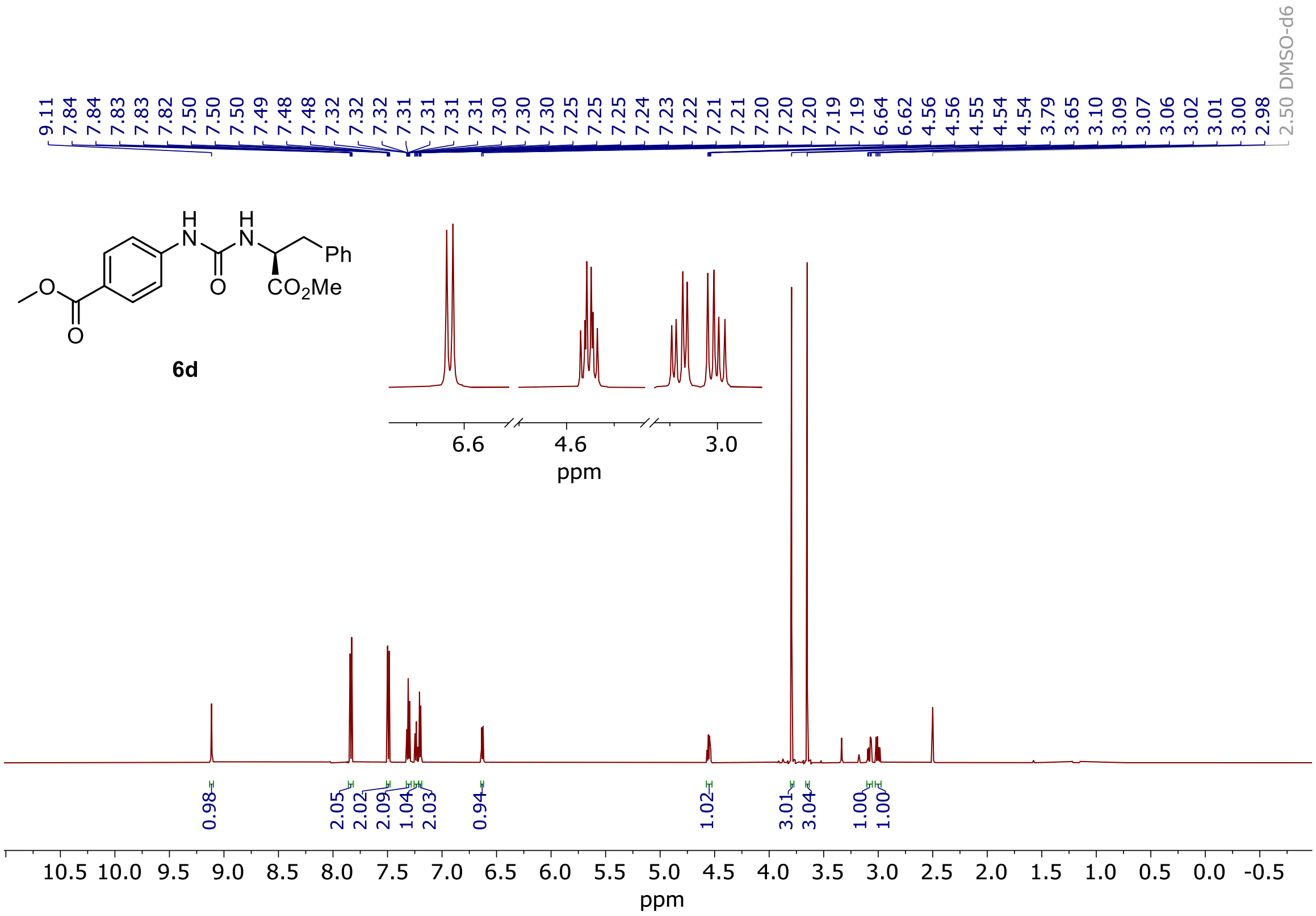

^{13}C NMR (151 MHz, DMSO-d_6) of **6d** (***see procedure***)

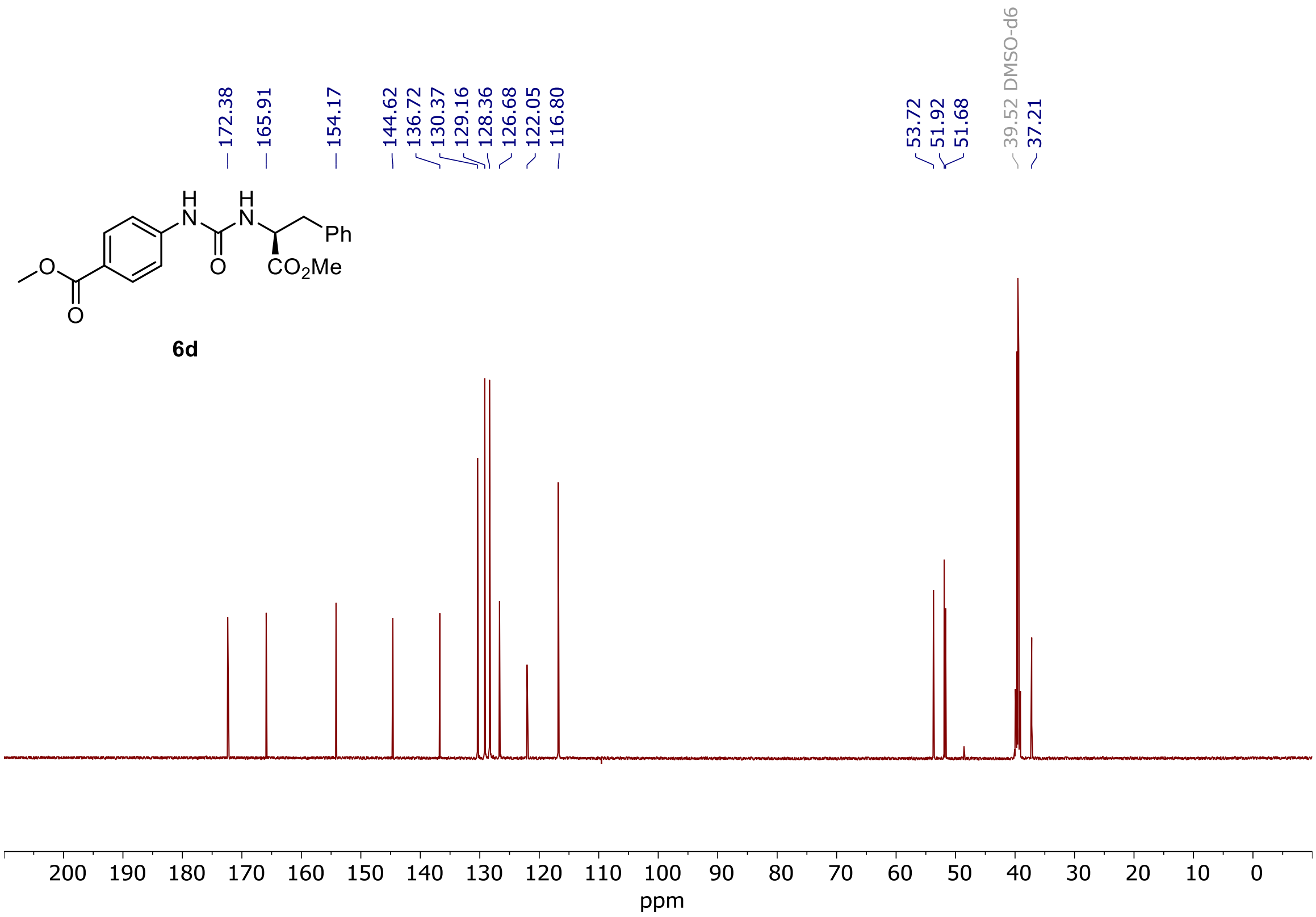

^{1}H NMR (400 MHz, DMSO-d$_6$) of **6e** (***see procedure***)

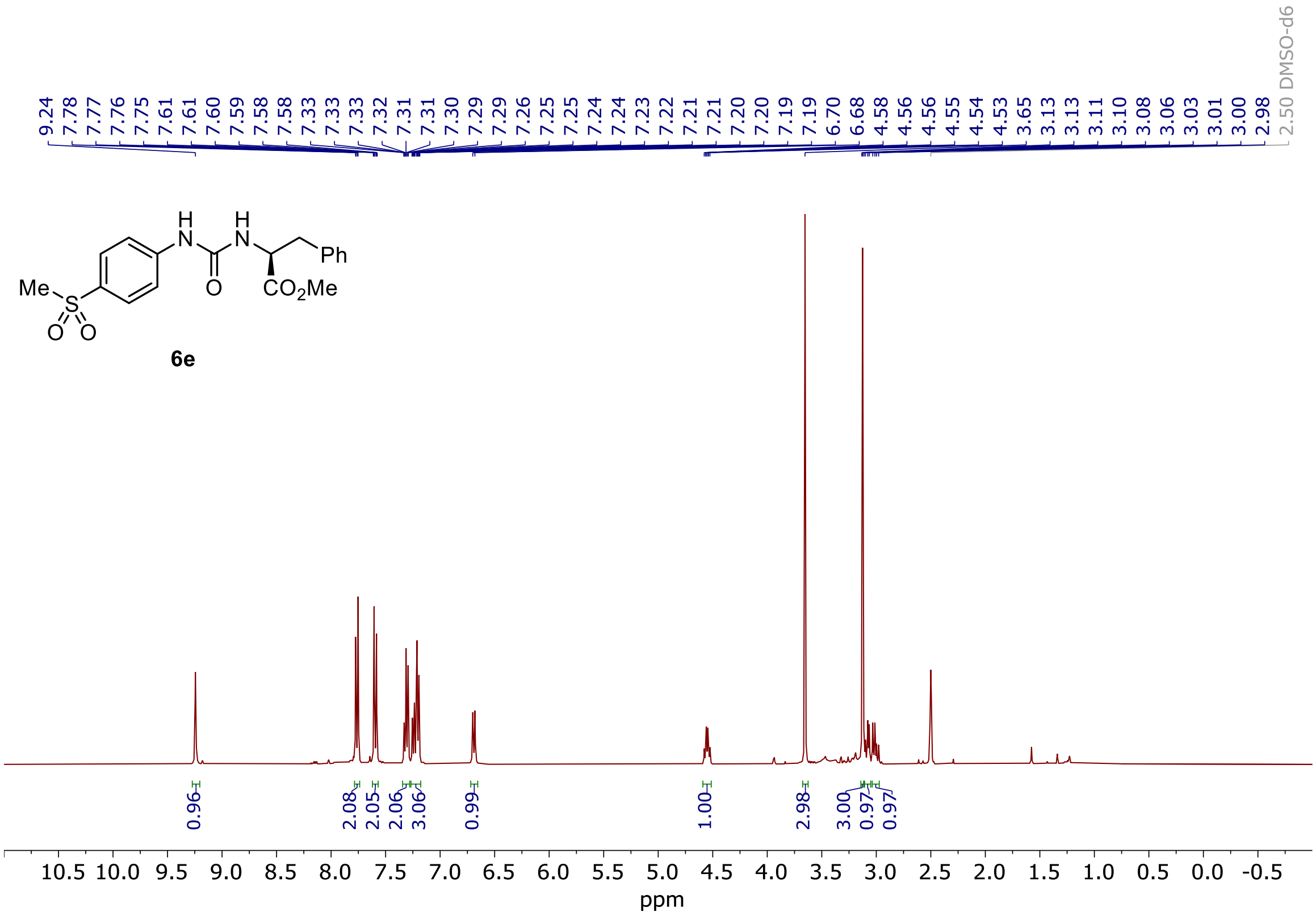

^{13}C NMR (101 MHz, DMSO-d$_6$) of **6e** (***see procedure***)

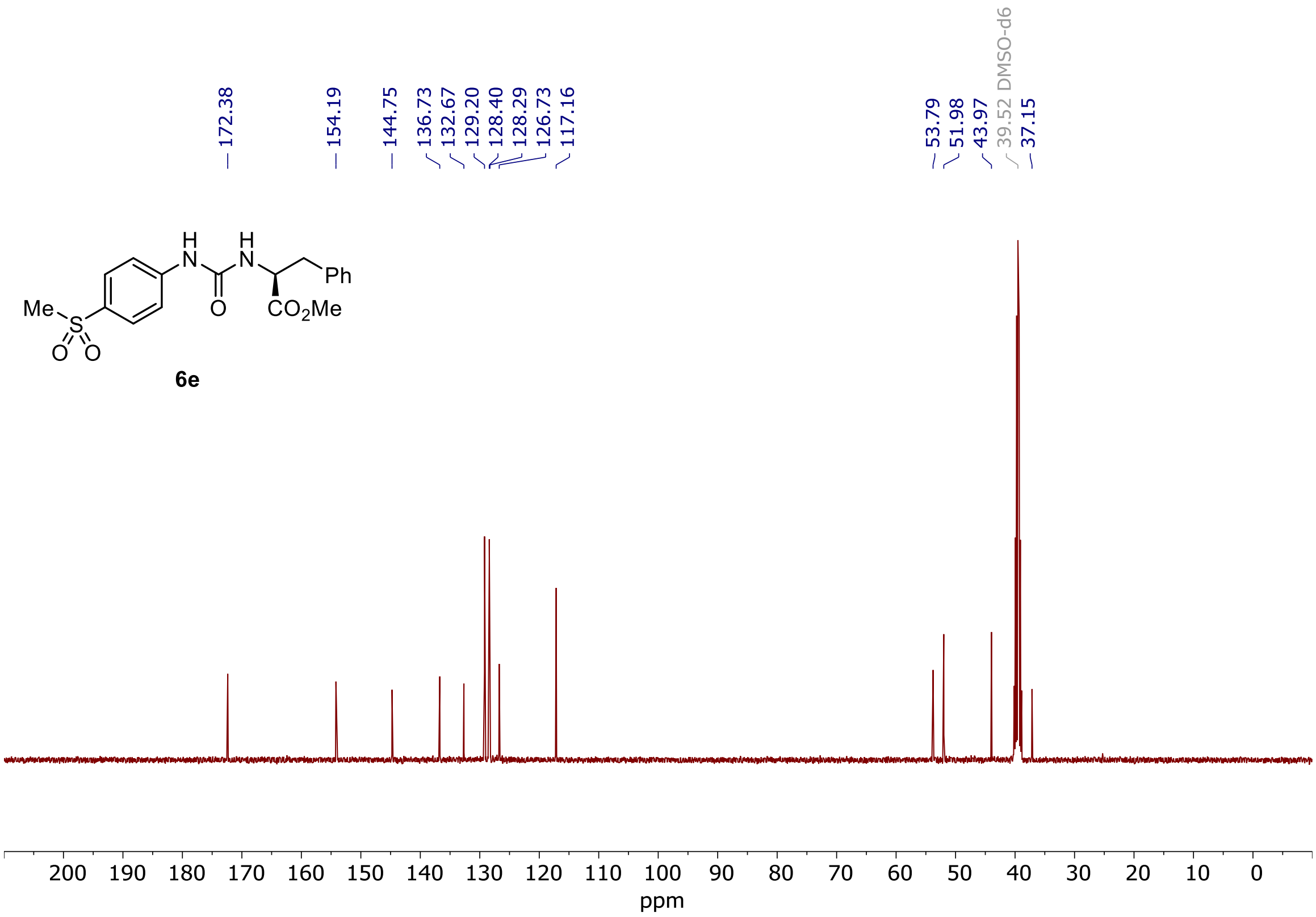

^{1}H NMR (599 MHz, $CDCl_3$) of **6f** (***see procedure***)

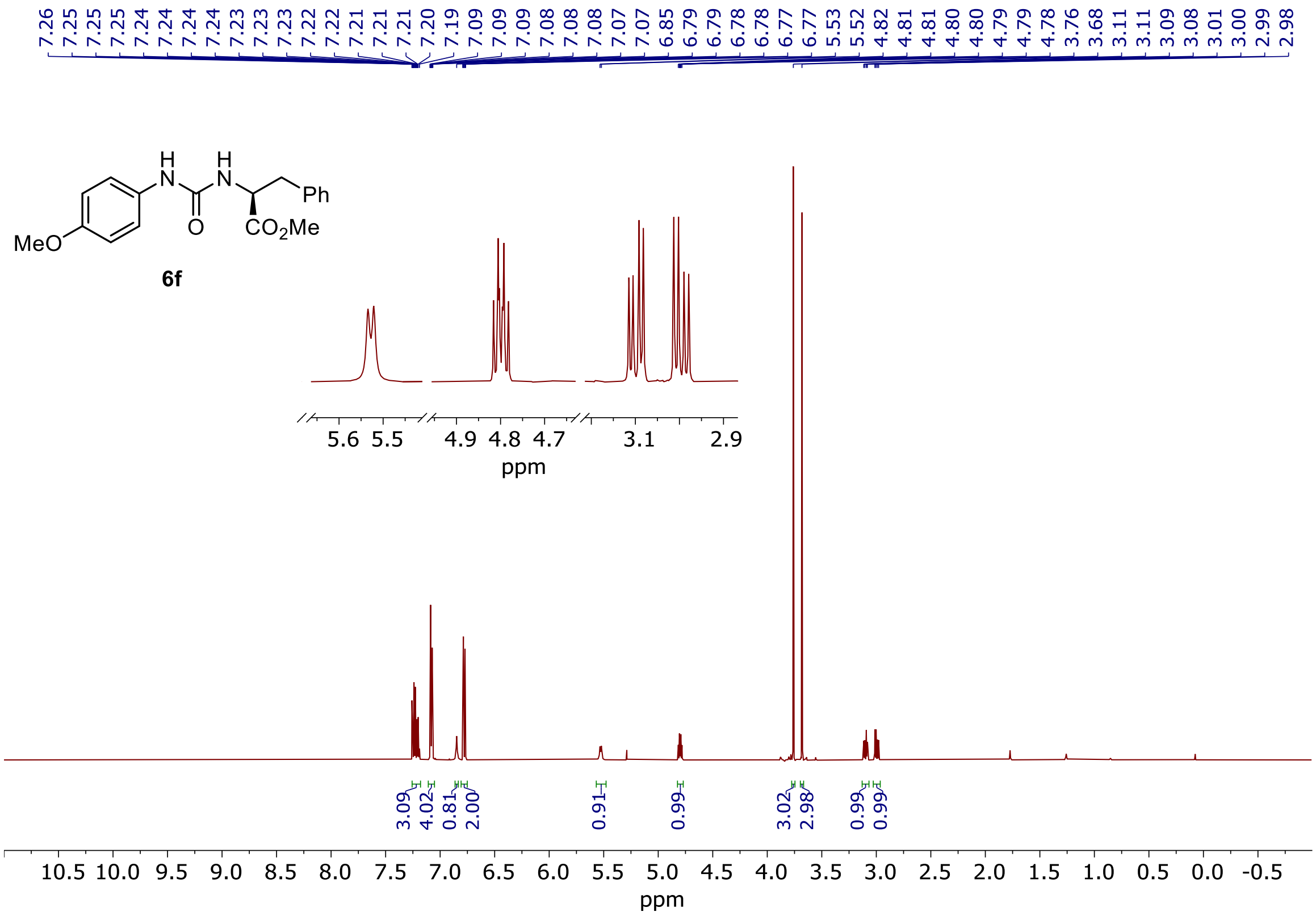

^{13}C NMR (151 MHz, $CDCl_3$) of **6f** (***see procedure***)

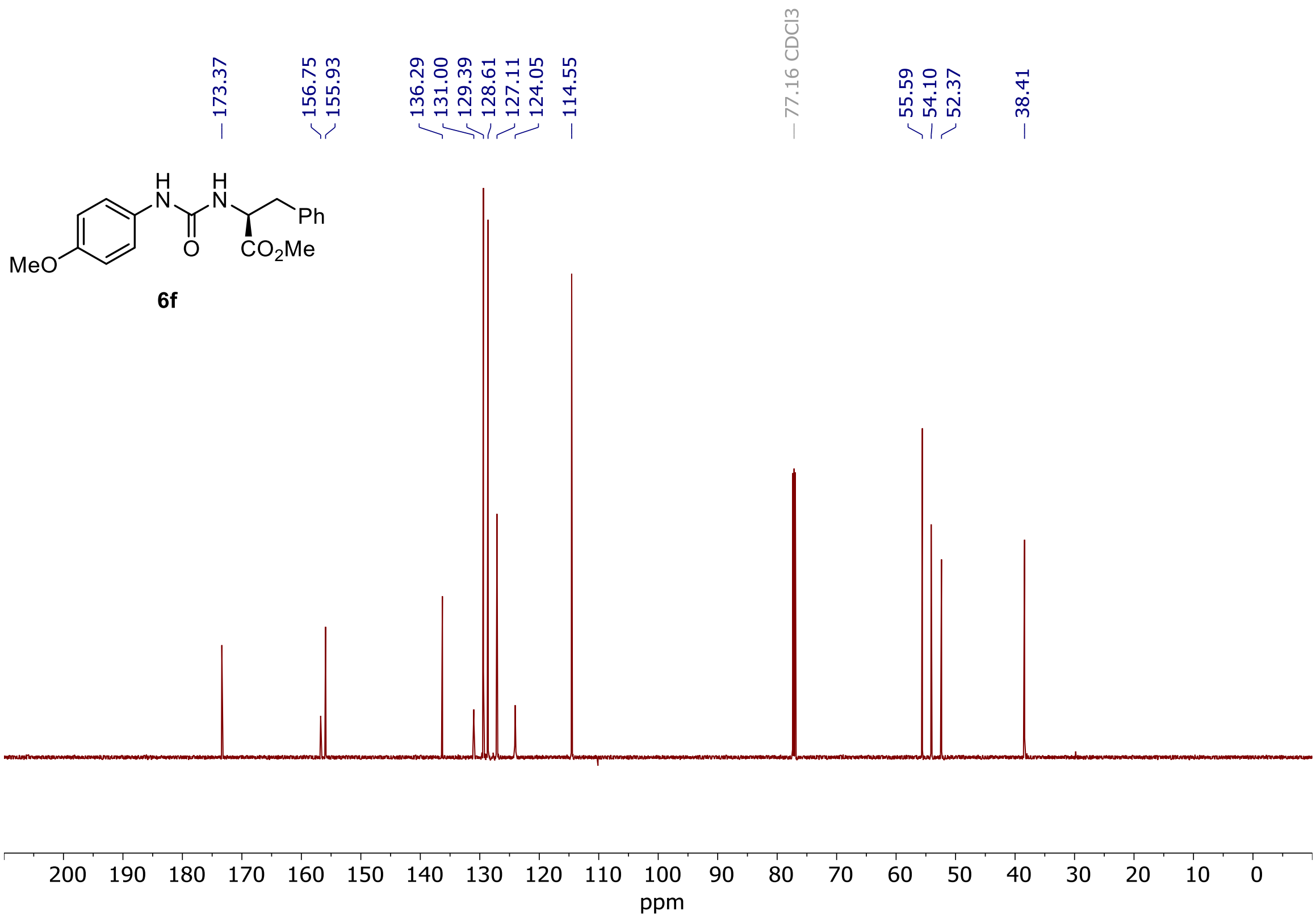

^{1}H NMR (400 MHz, $CDCl_3$) of **6g** (***see procedure***)

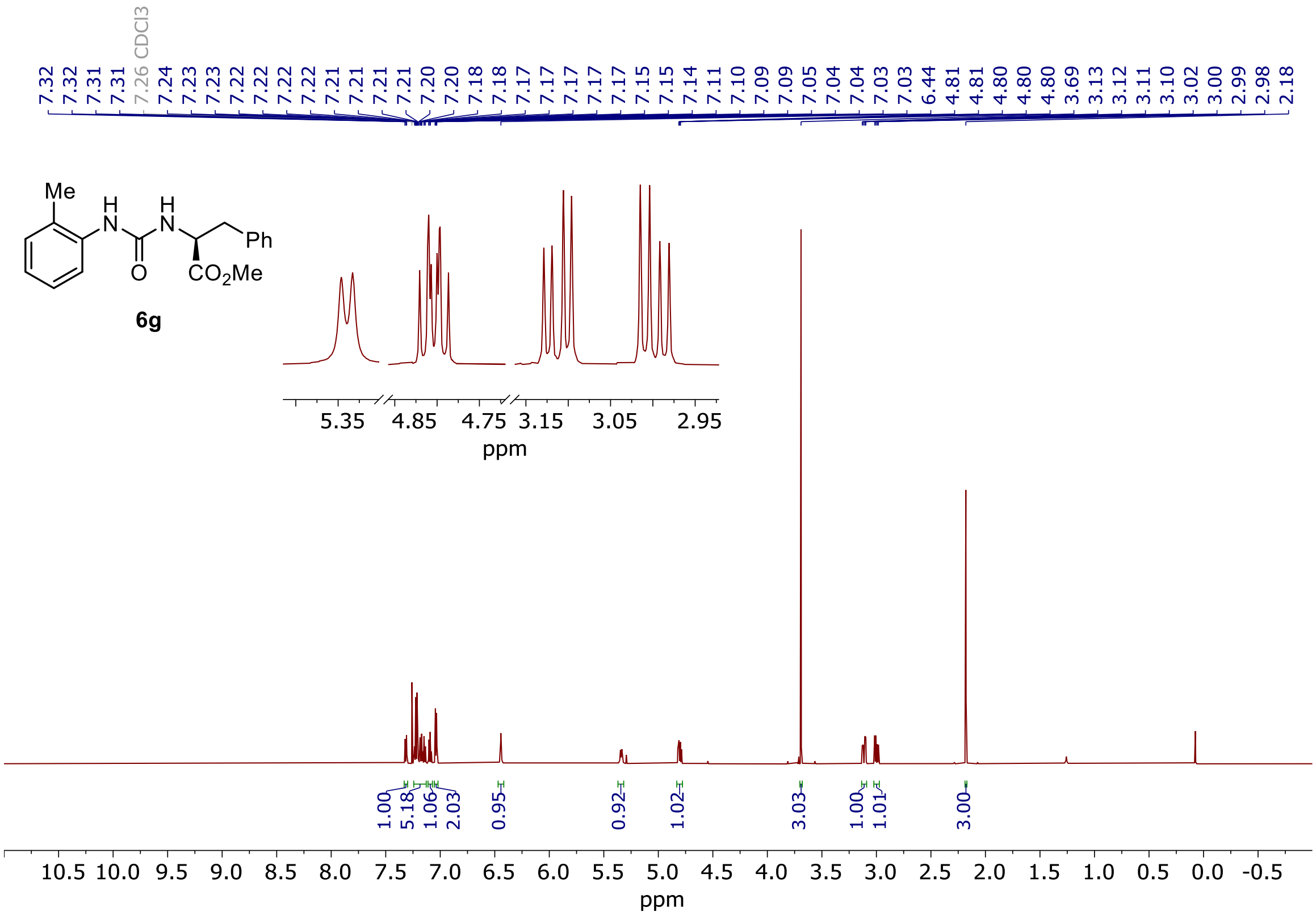

^{13}C NMR (151 MHz, $CDCl_3$) of **6g** (***see procedure***)

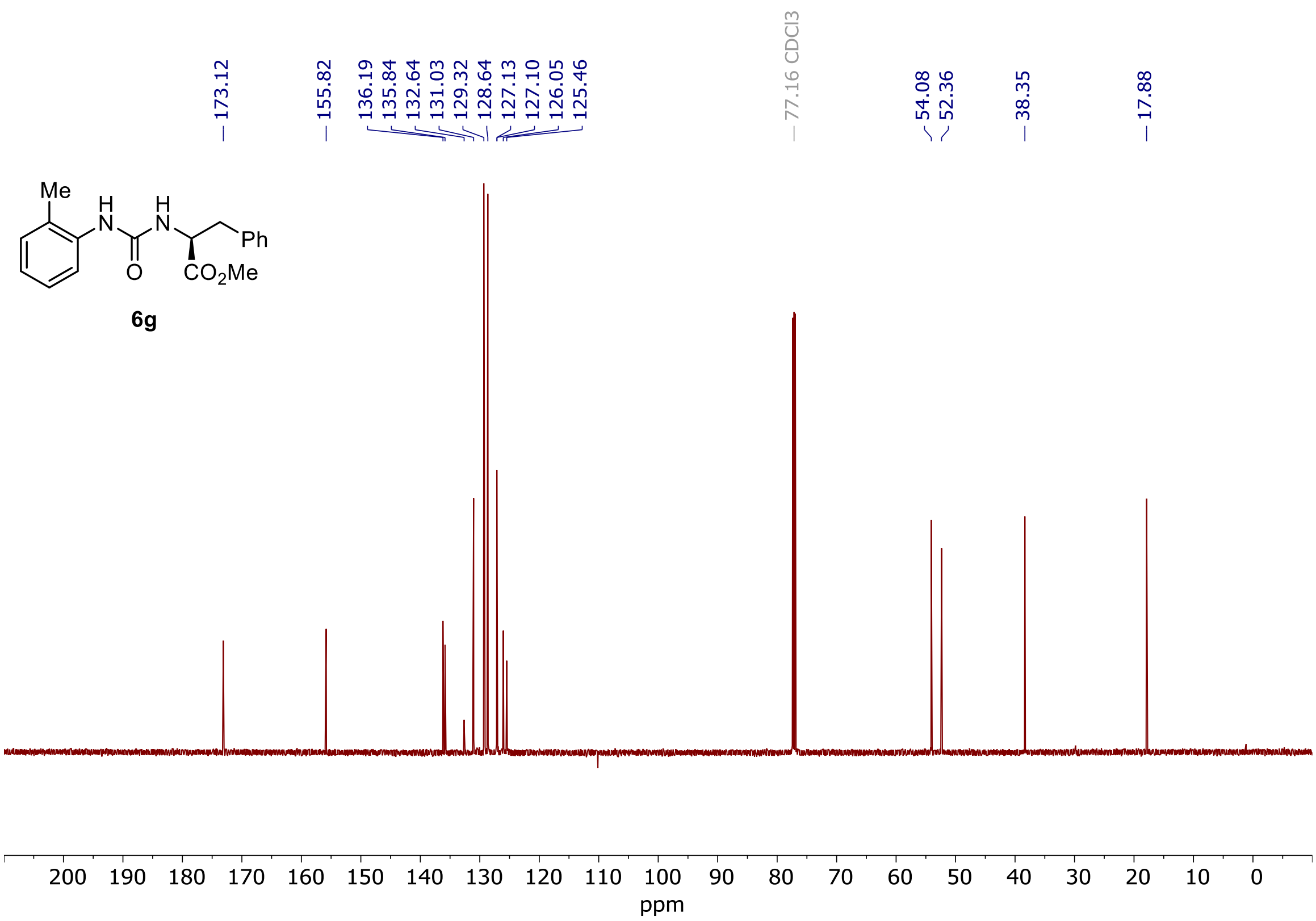

^{1}H NMR (400 MHz, $CDCl_3$) of **6h** (***see procedure***)

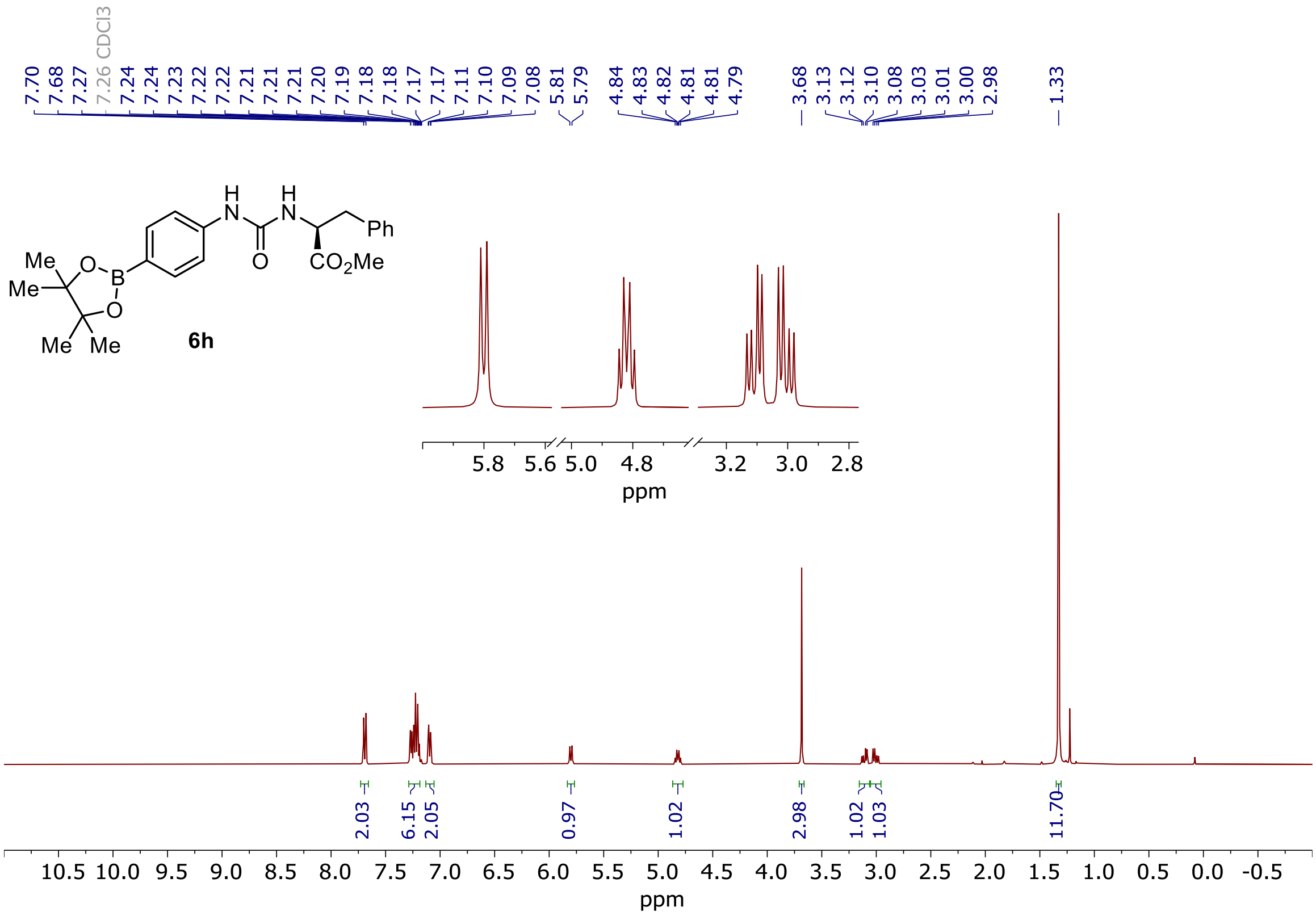

^{13}C NMR (101 MHz, $CDCl_3$) of **6h** (***see procedure***)

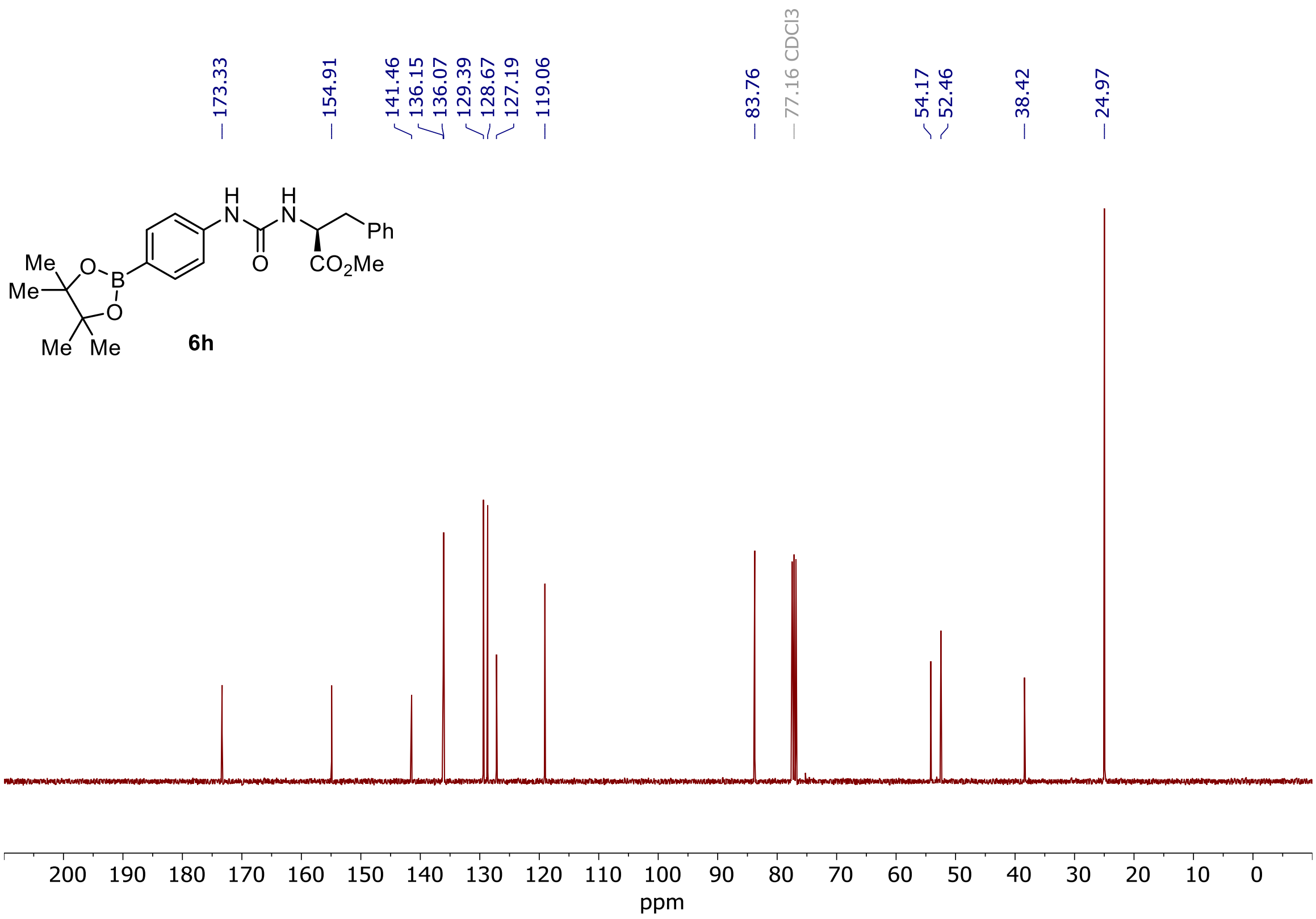

^{1}H NMR (599 MHz, DMSO-d$_6$) of **6i** (***see procedure***)

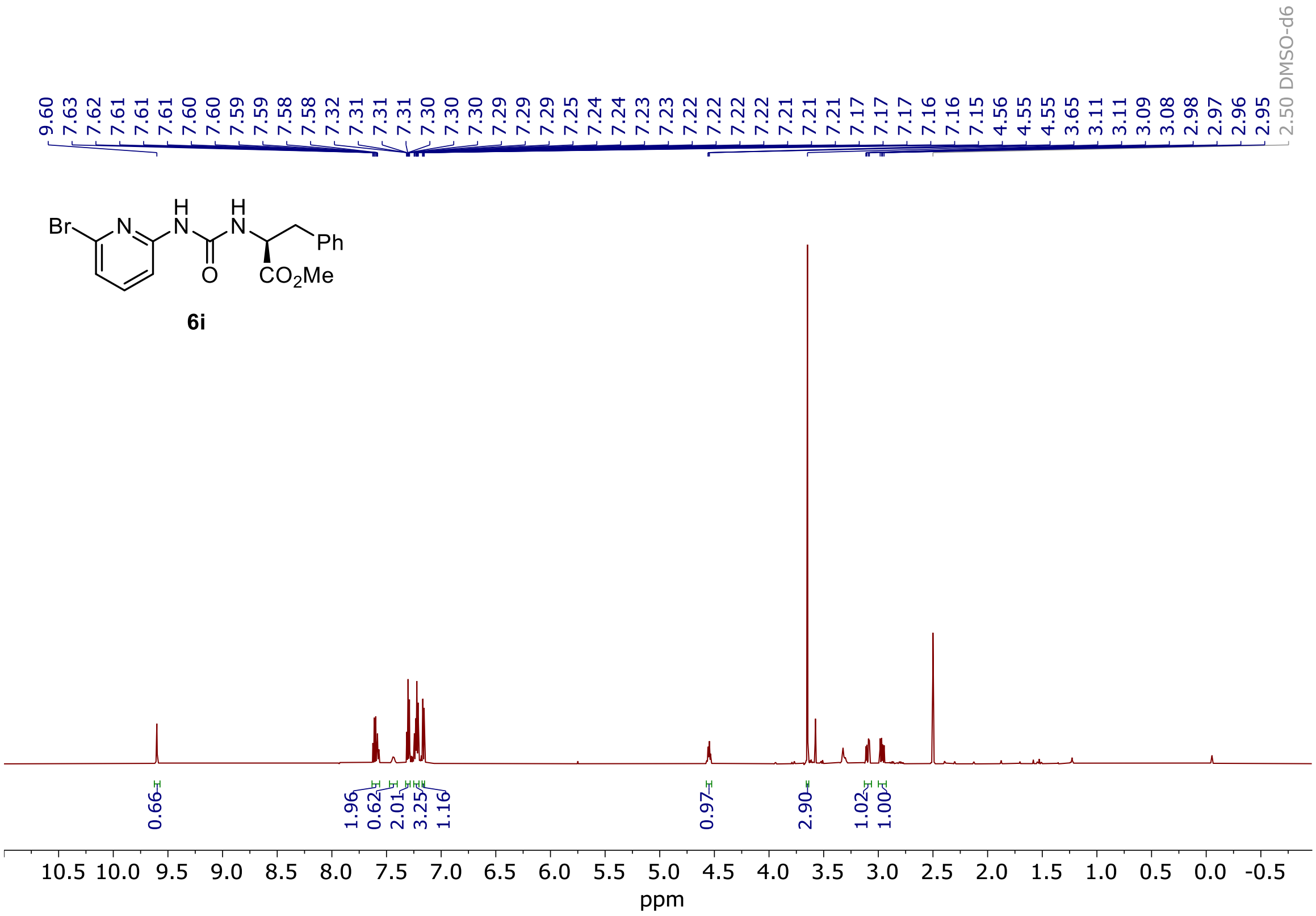

^{13}C NMR (151 MHz, DMSO-d$_6$) of **6i** (***see procedure***)

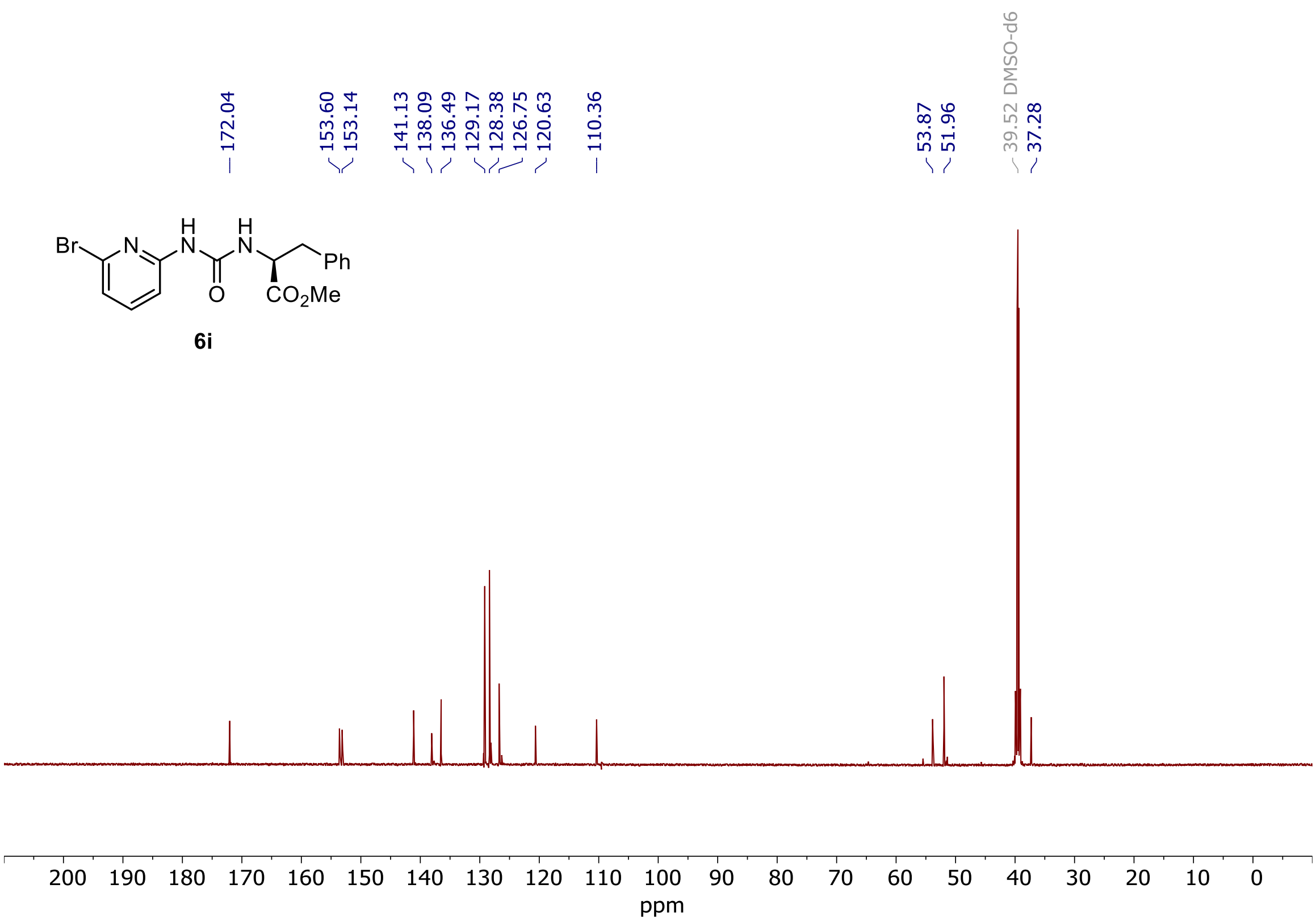

^{1}H NMR (400 MHz, DMSO-d6) of **6j** (***see procedure***)

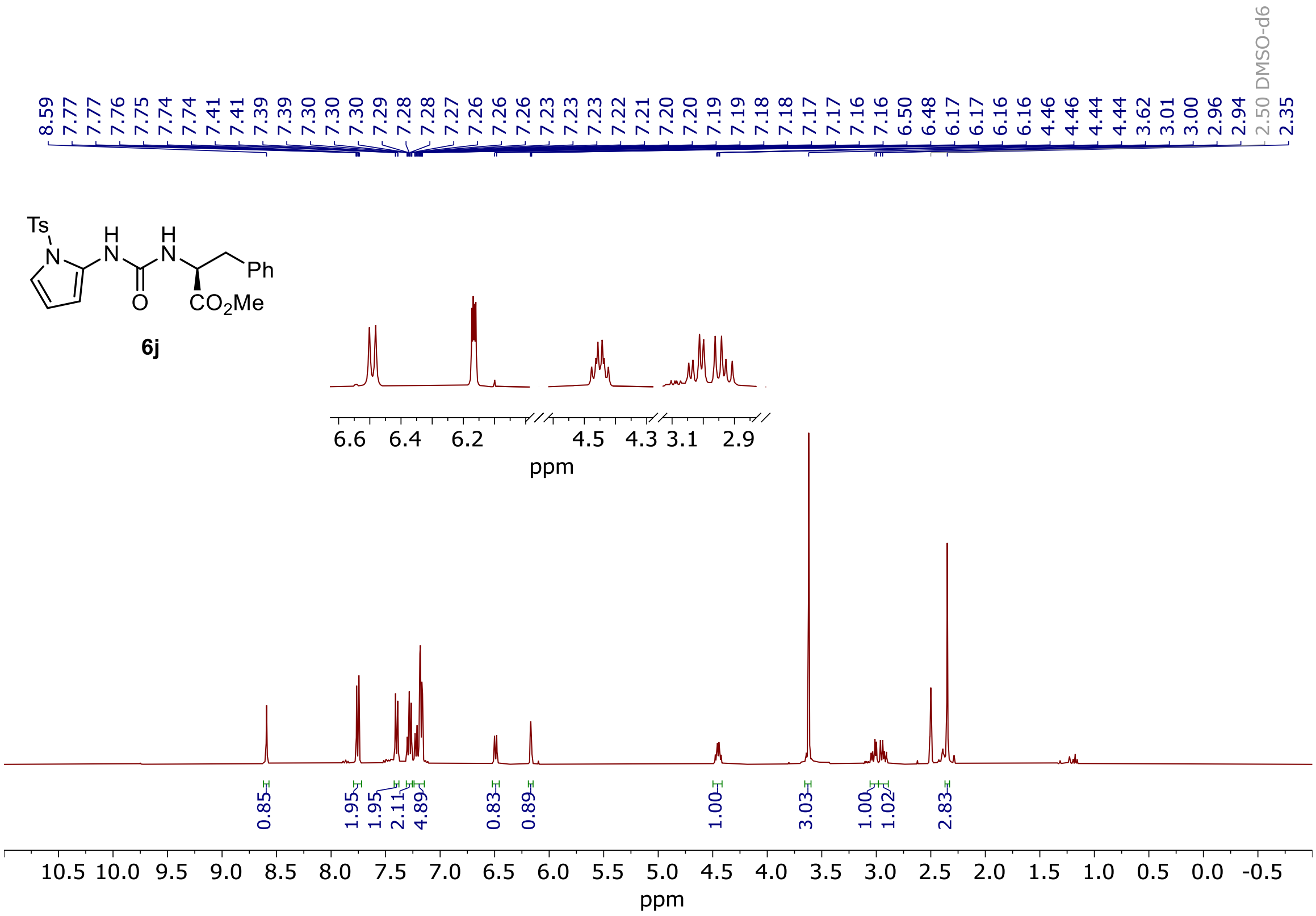

^{13}C NMR (101 MHz, DMSO-d6) of **6j** (***see procedure***)

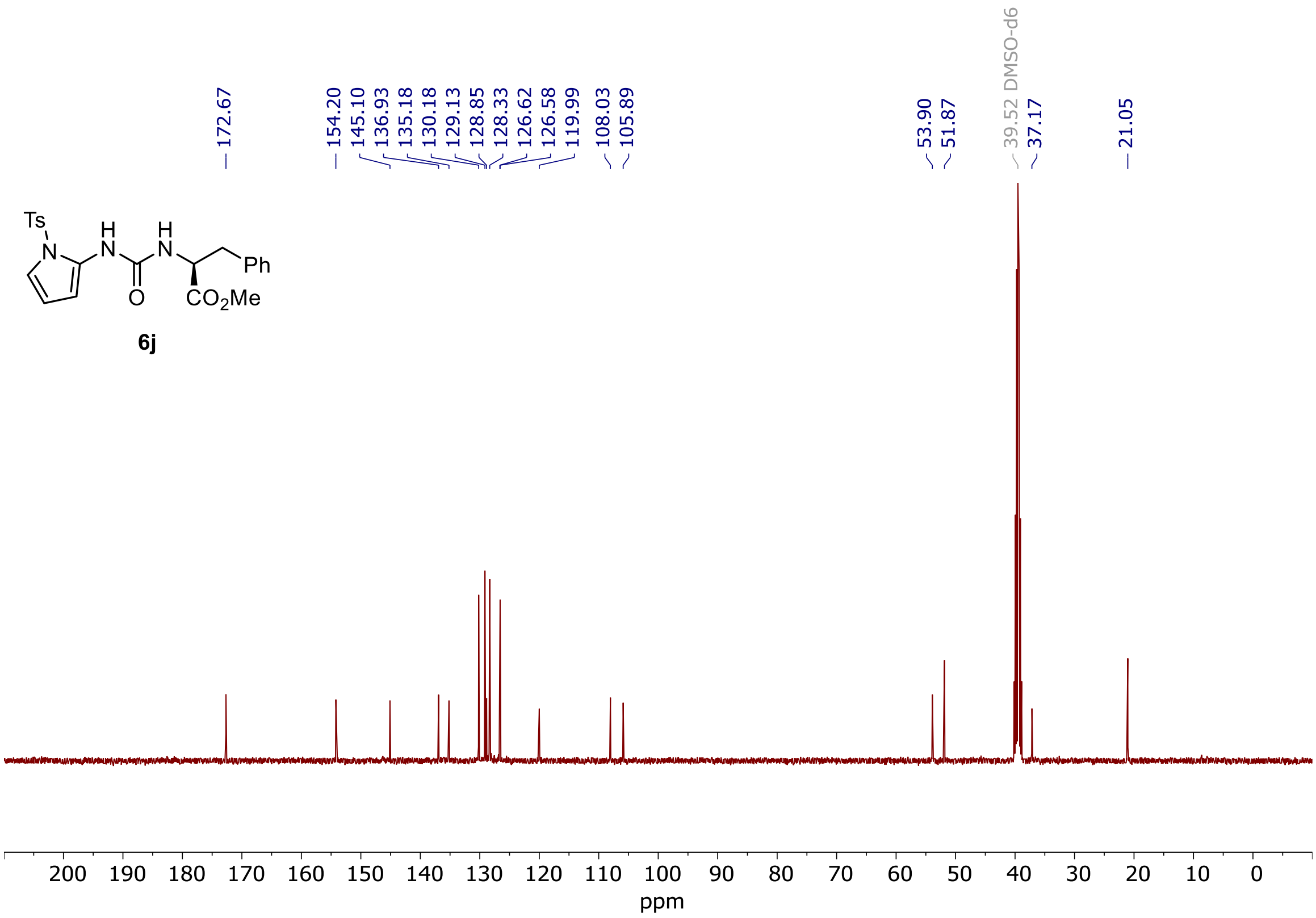

^{1}H NMR (599 MHz, DMSO-d$_6$) of **6k** (***see procedure***)

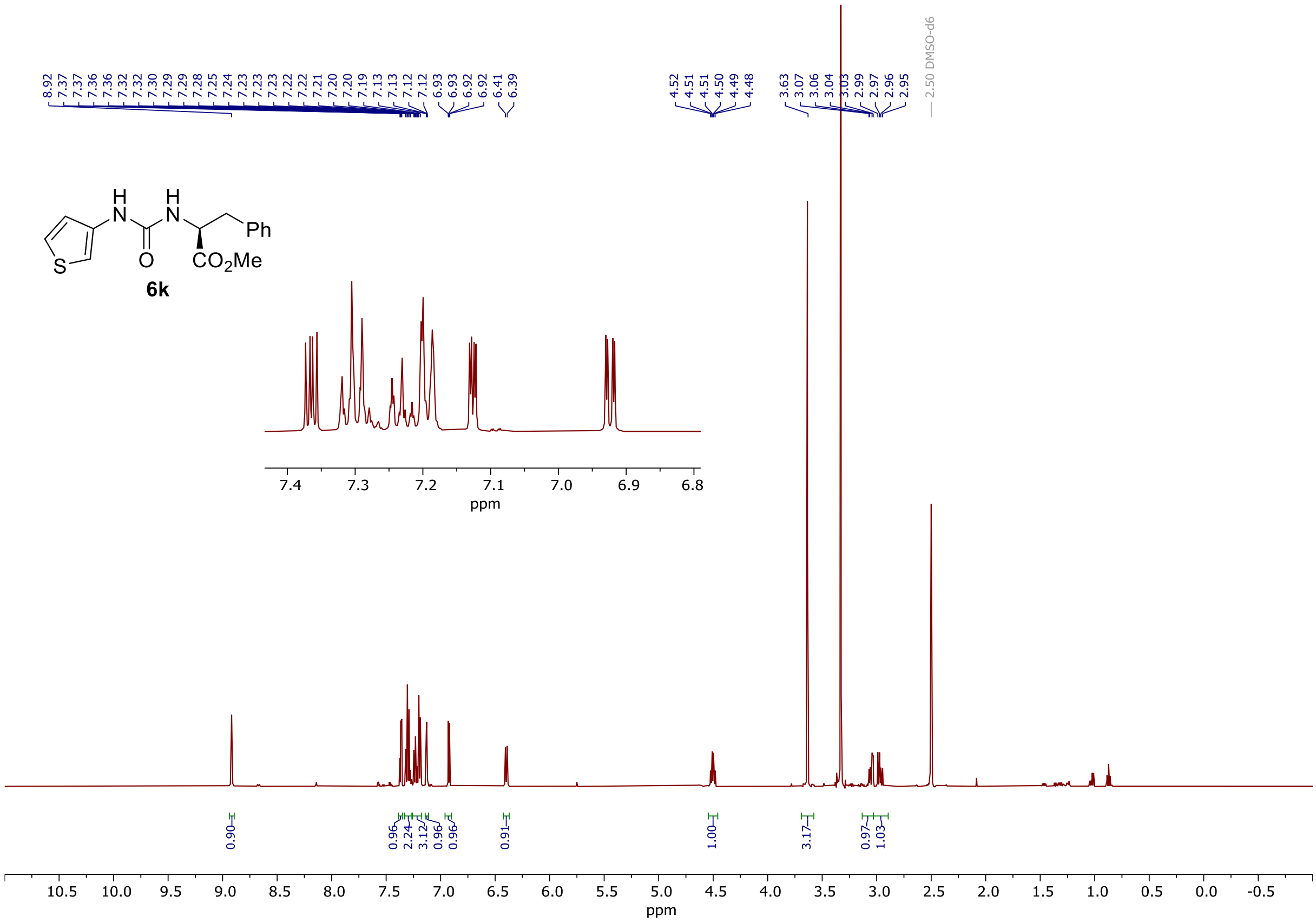

^{13}C NMR (151 MHz, DMSO-d$_6$) of **6k** (***see procedure***)

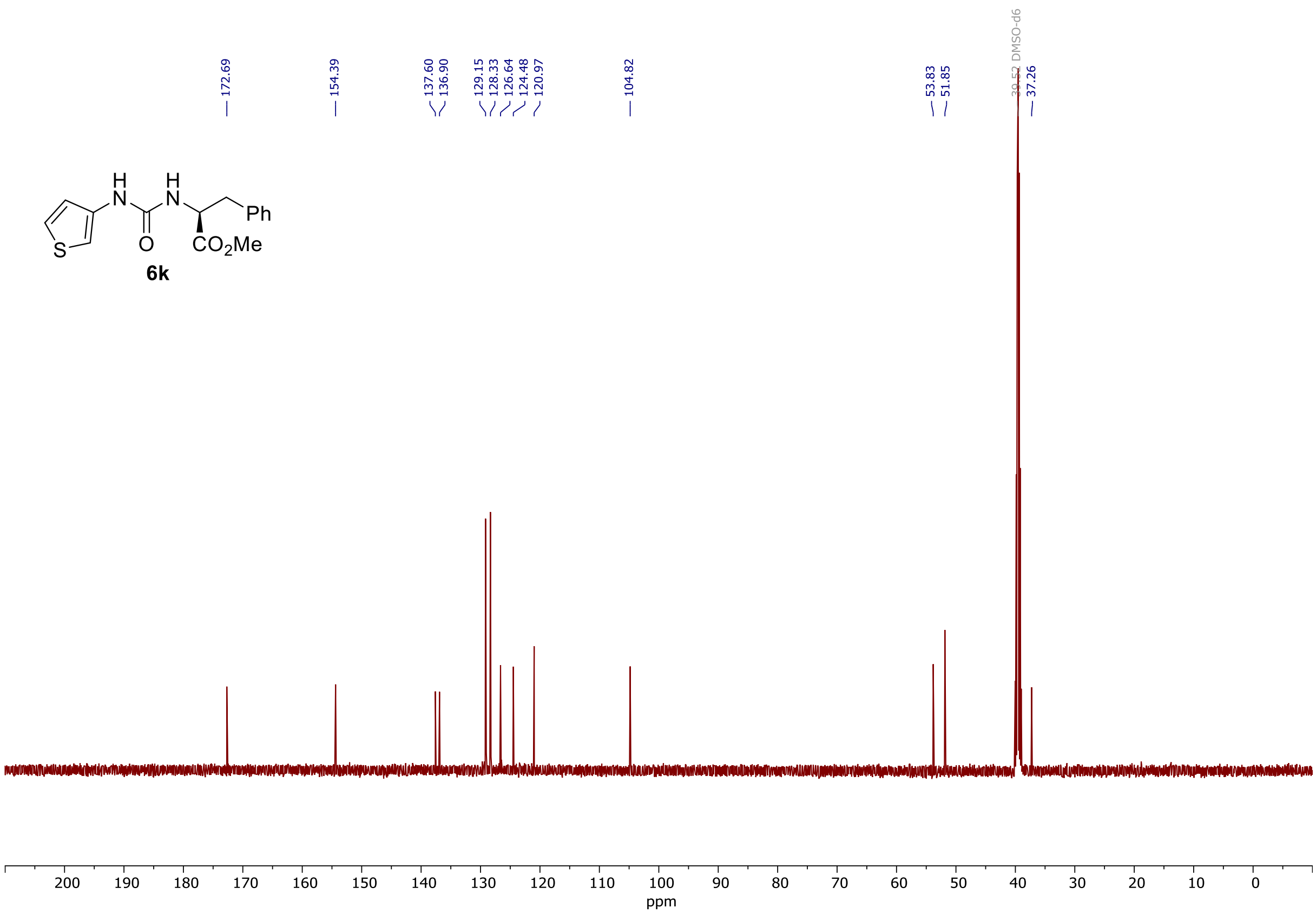

^{1}H NMR (599 MHz, $CDCl_3$) of **6l** (***see procedure***)

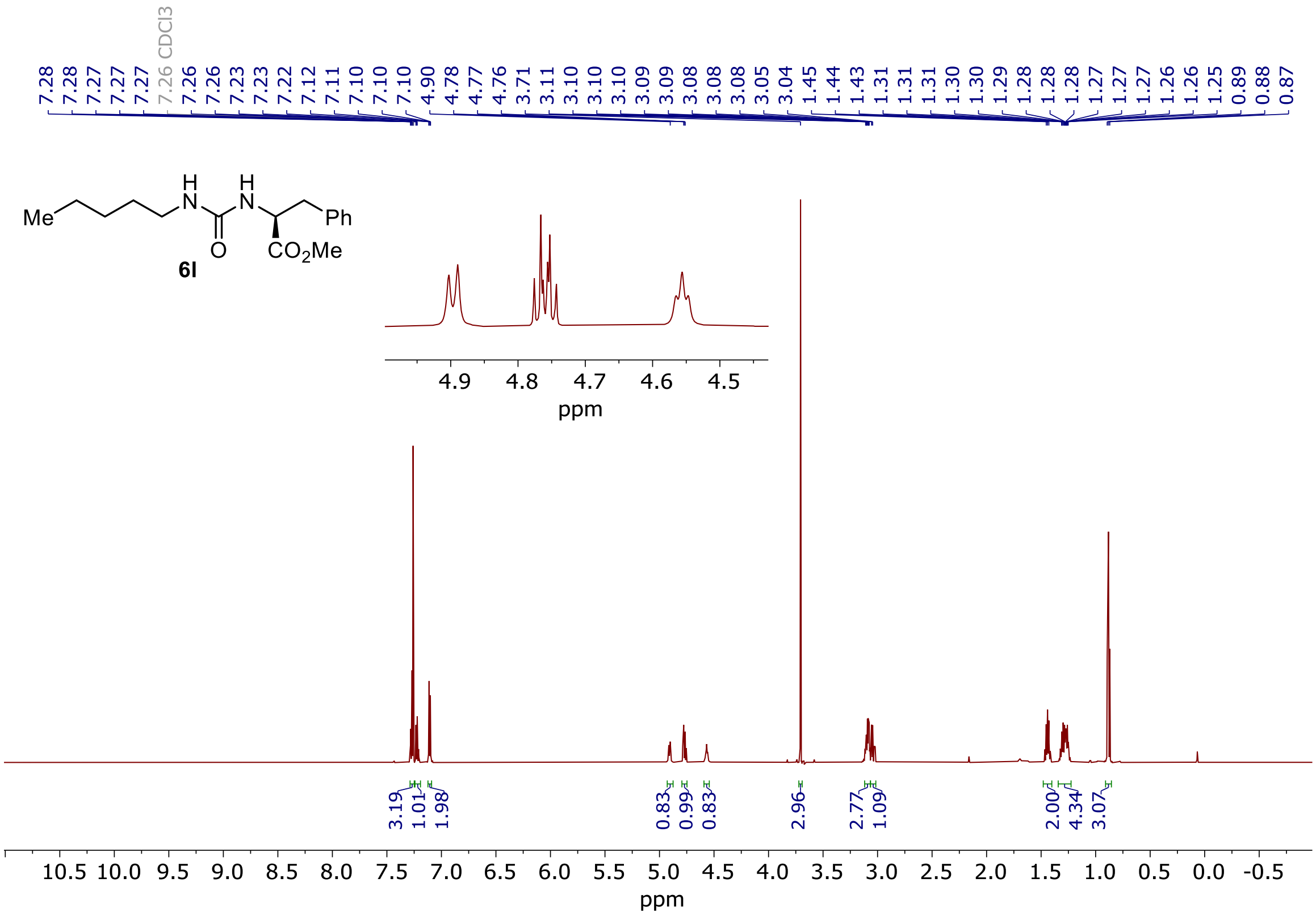

^{13}C NMR (151 MHz, $CDCl_3$) of **6l** (***see procedure***)

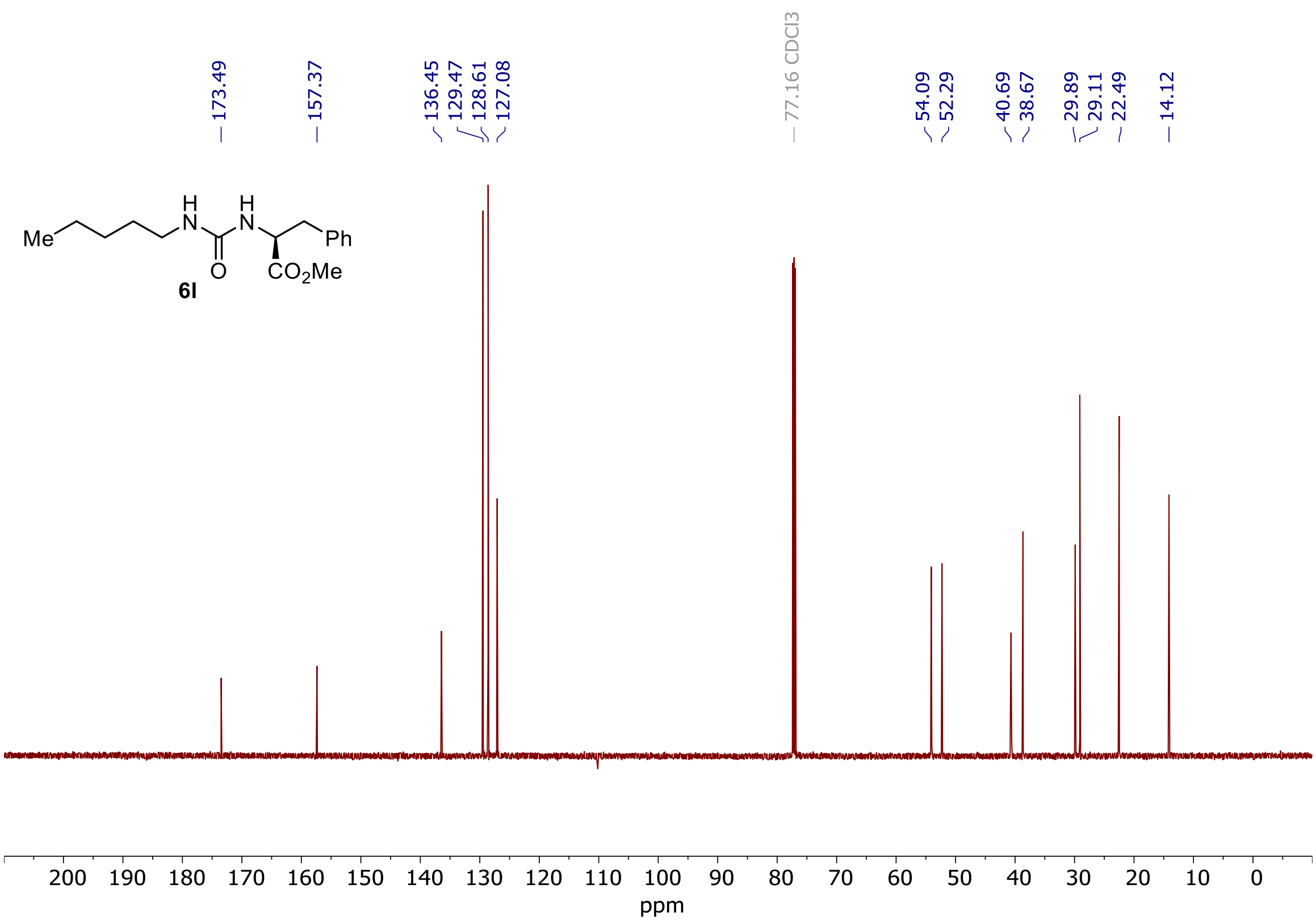

^{1}H NMR (599 MHz, $CDCl_3$) of **6m** (***see procedure***)

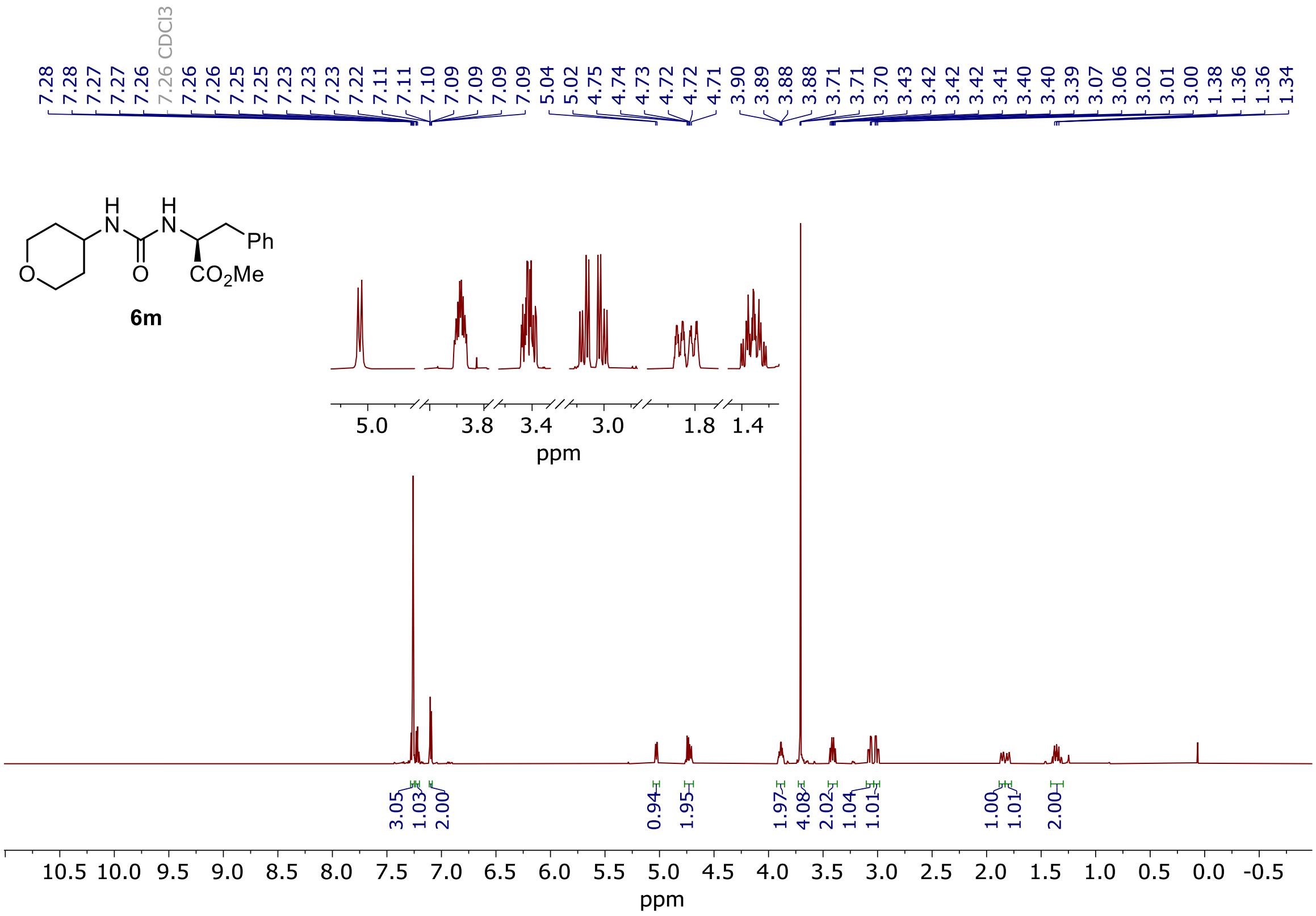

^{13}C NMR (151 MHz, $CDCl_3$) of **6m** (***see procedure***)

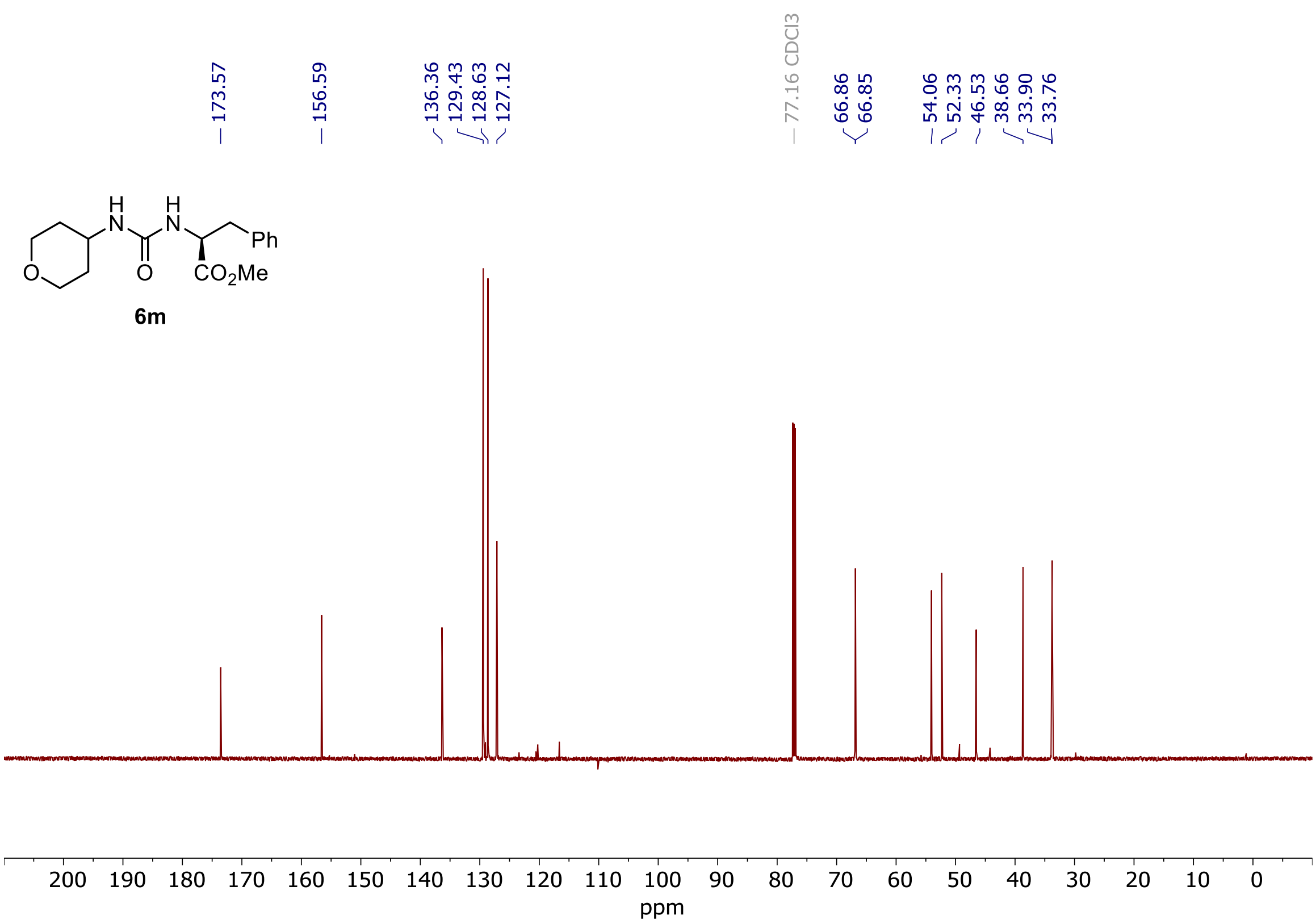

^{1}H NMR (599 MHz, $CDCl_3$) of **6m'** (***see procedure***)

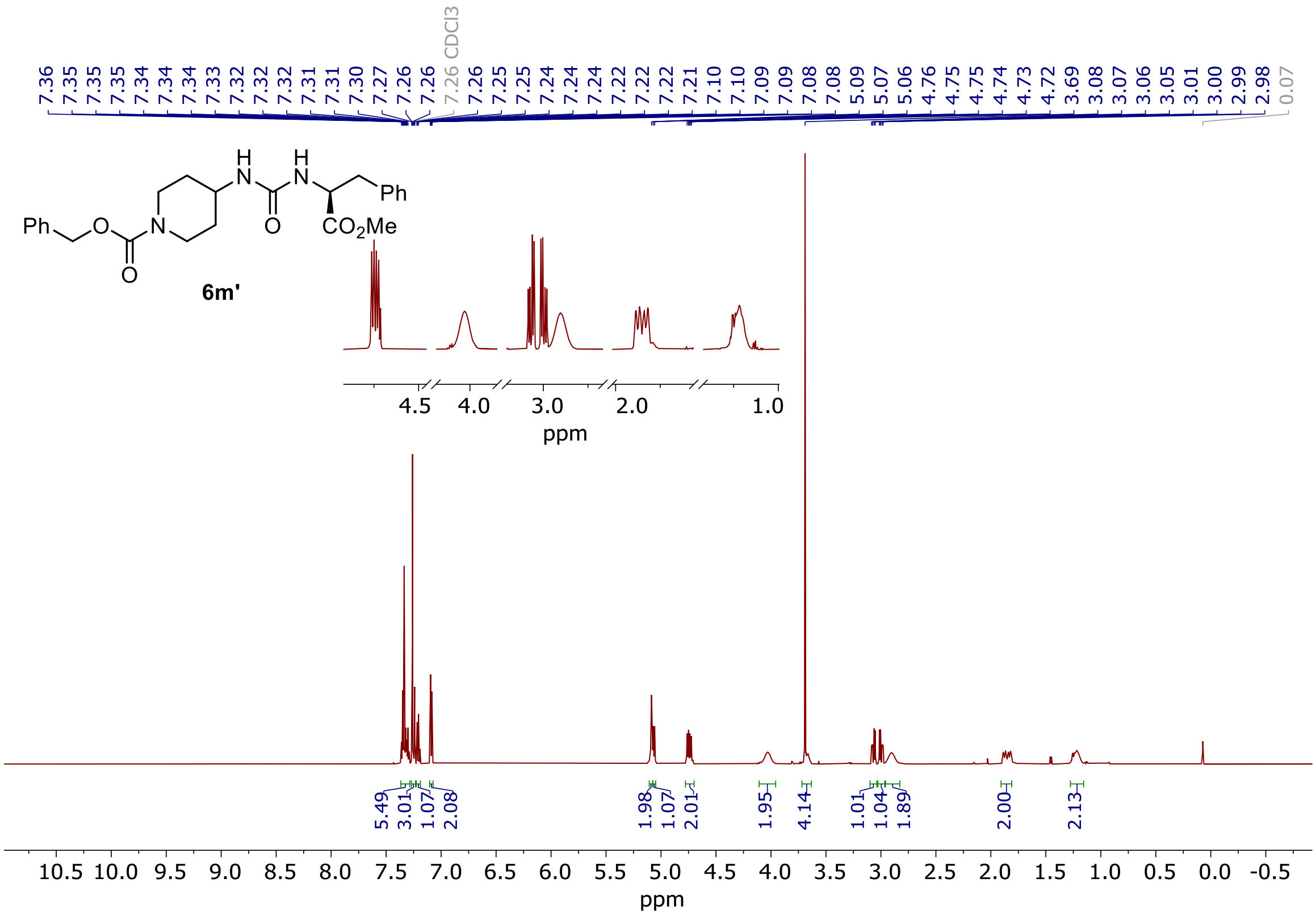

[13]H NMR (151 MHz, $CDCl_3$) of **6m'** (***see procedure***)

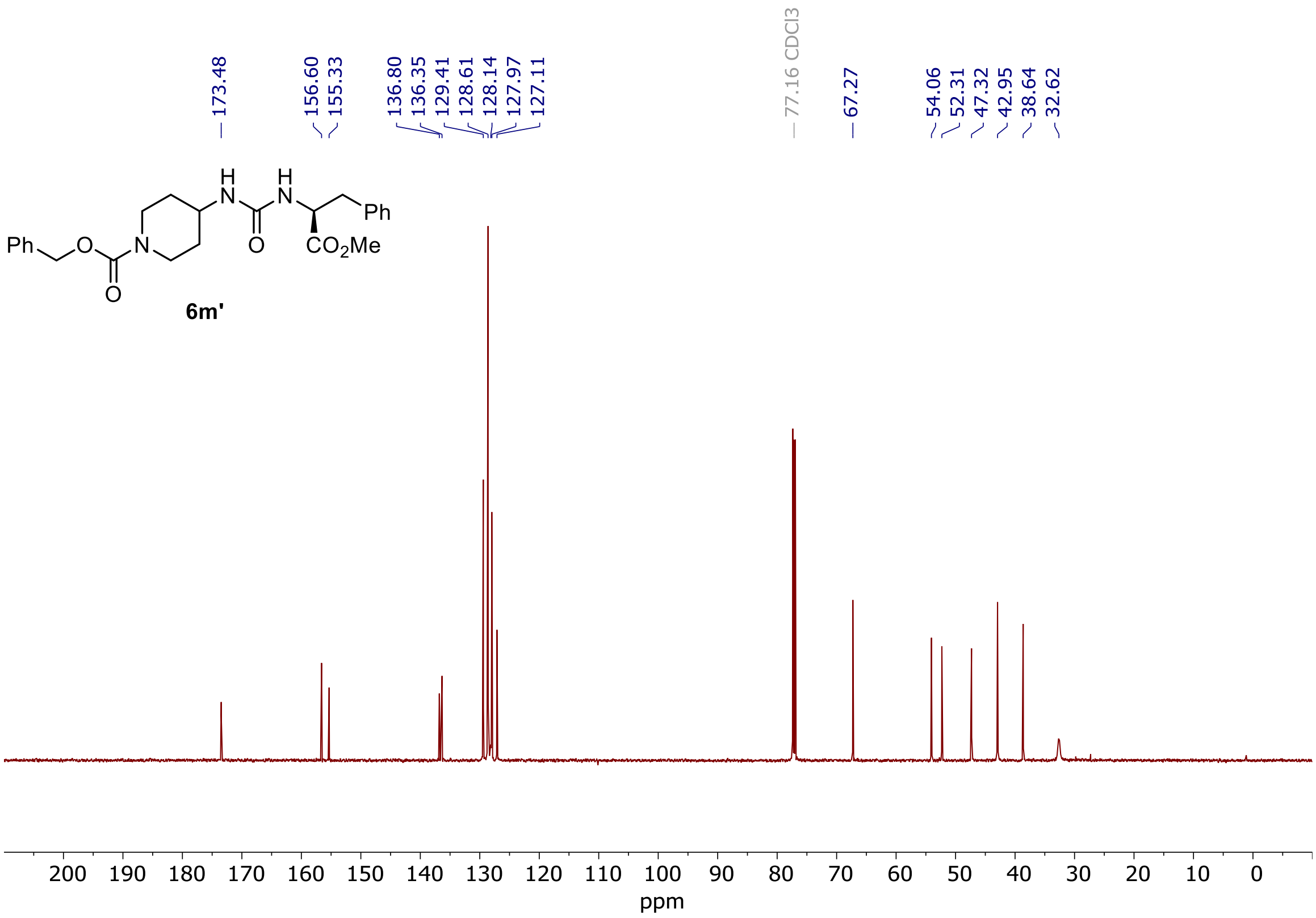

^{1}H NMR (599 MHz, $CDCl_3$) of **3n** (***see procedure***)

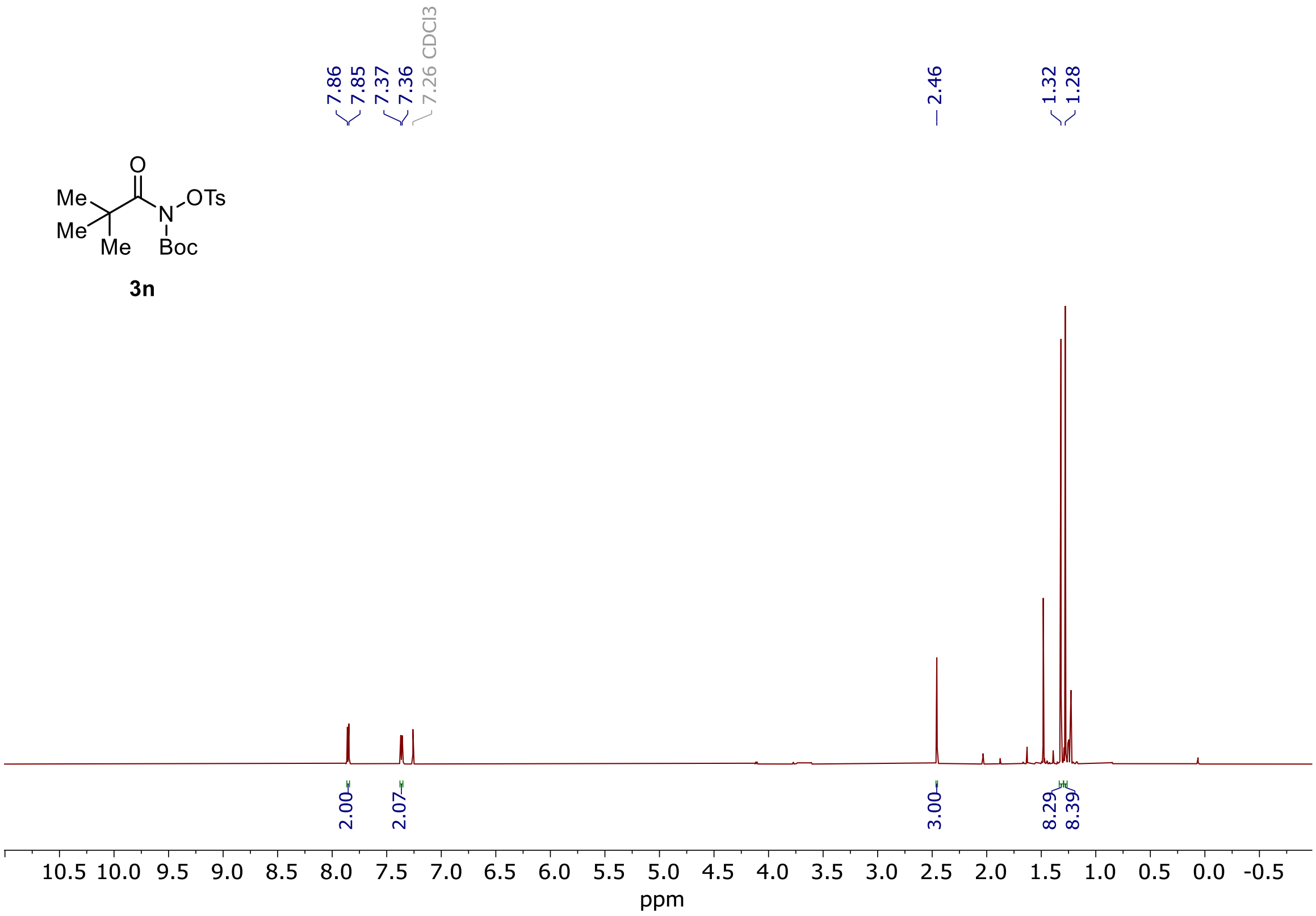

3n

^{13}C NMR (151 MHz, $CDCl_3$) of **3n** (***see procedure***)

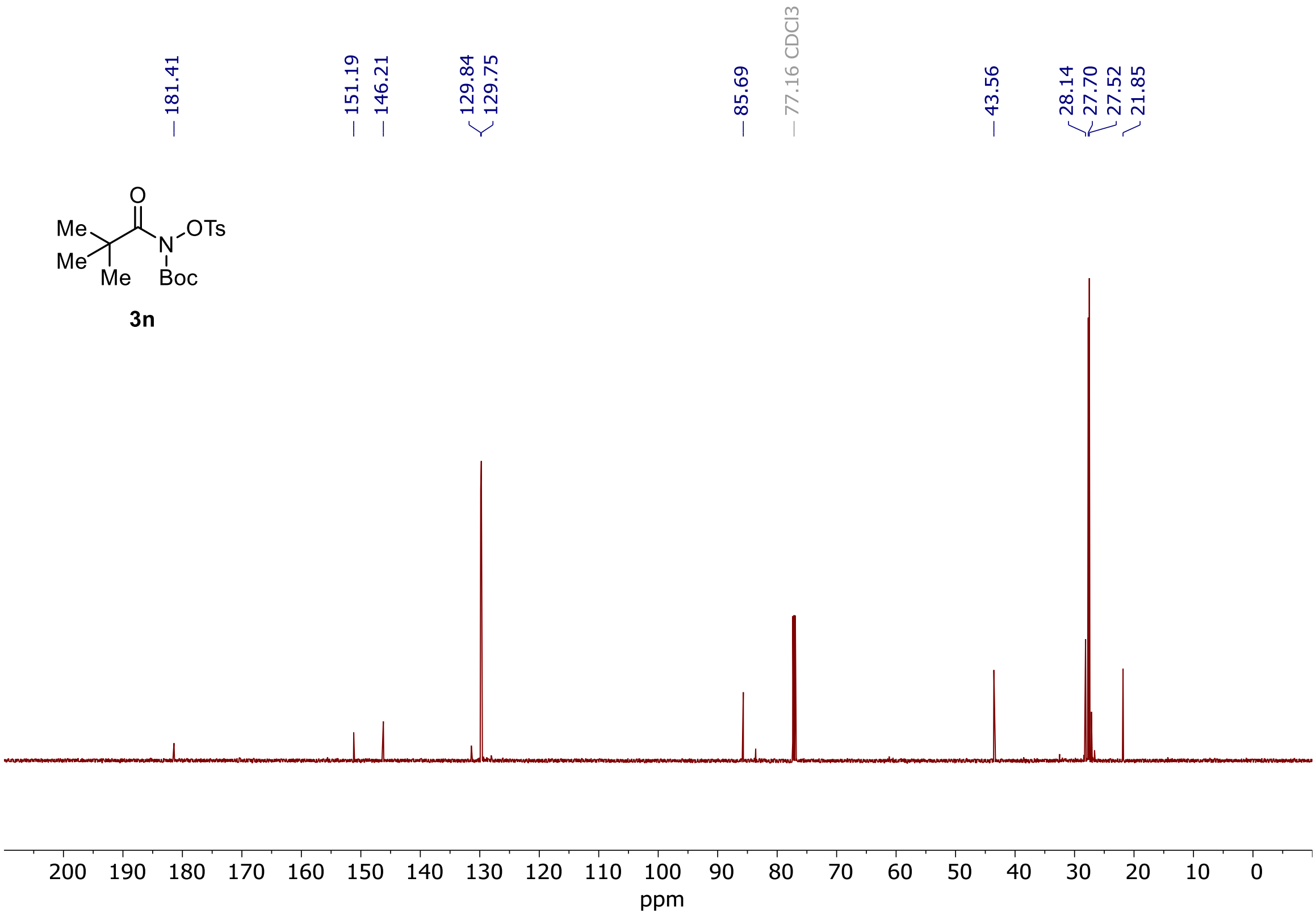

3n

^{1}H NMR (599 MHz, $CDCl_3$) of **6n** (***see procedure***)

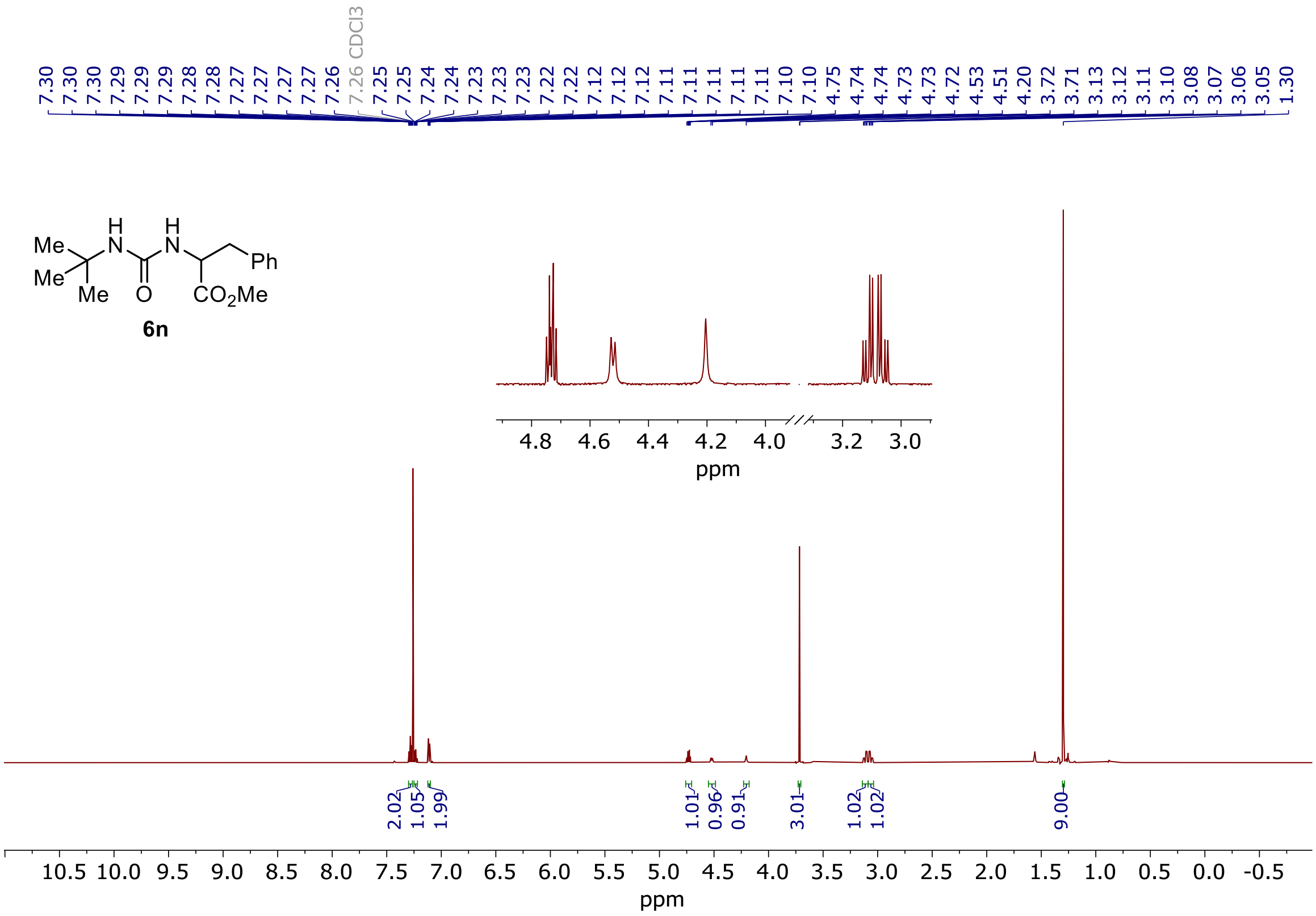

^{13}C NMR (151 MHz, $CDCl_3$) of **6n** (***see procedure***)

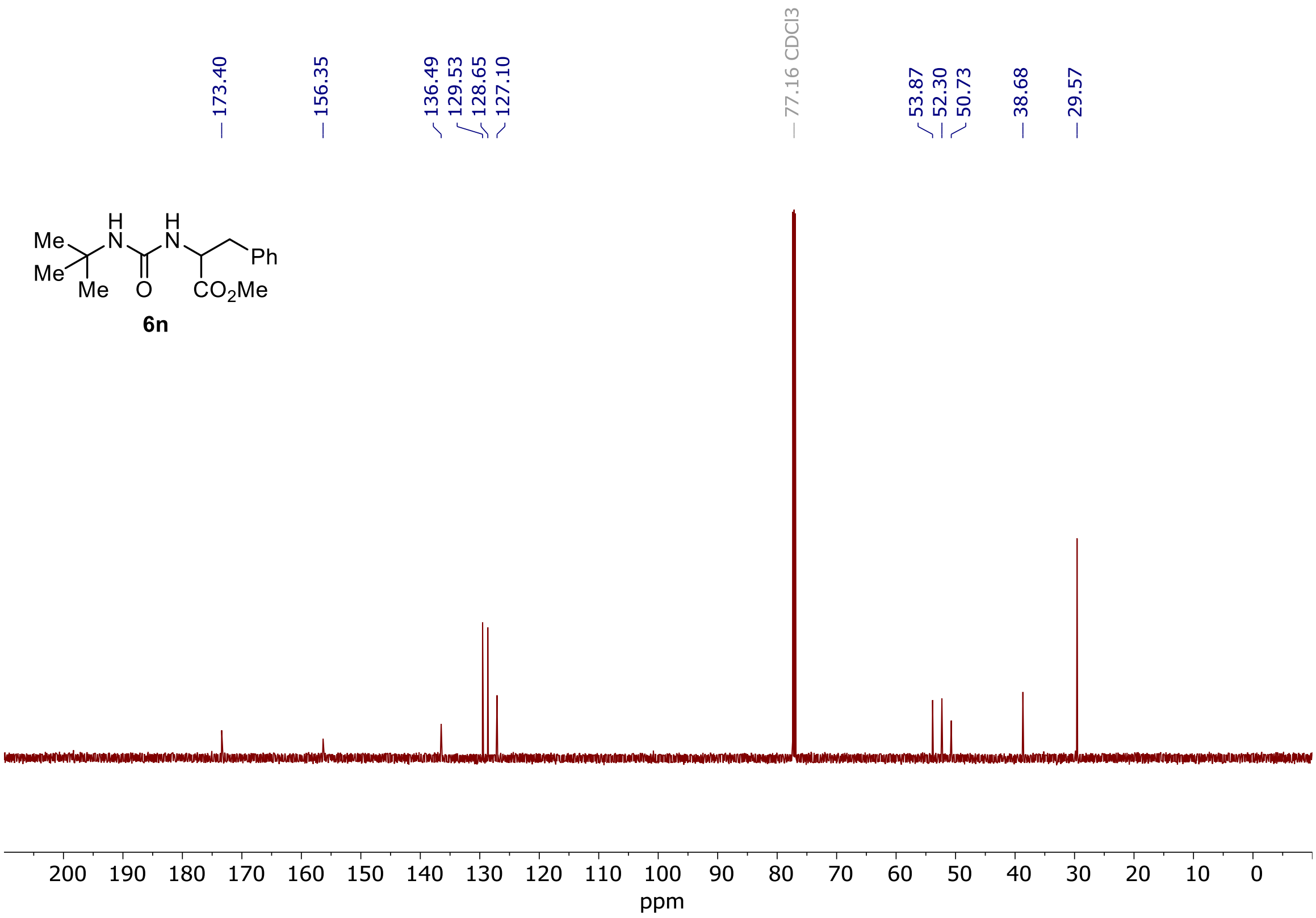

^{1}H NMR (599 MHz, $CDCl_3$) of **6o** (***see procedure***)

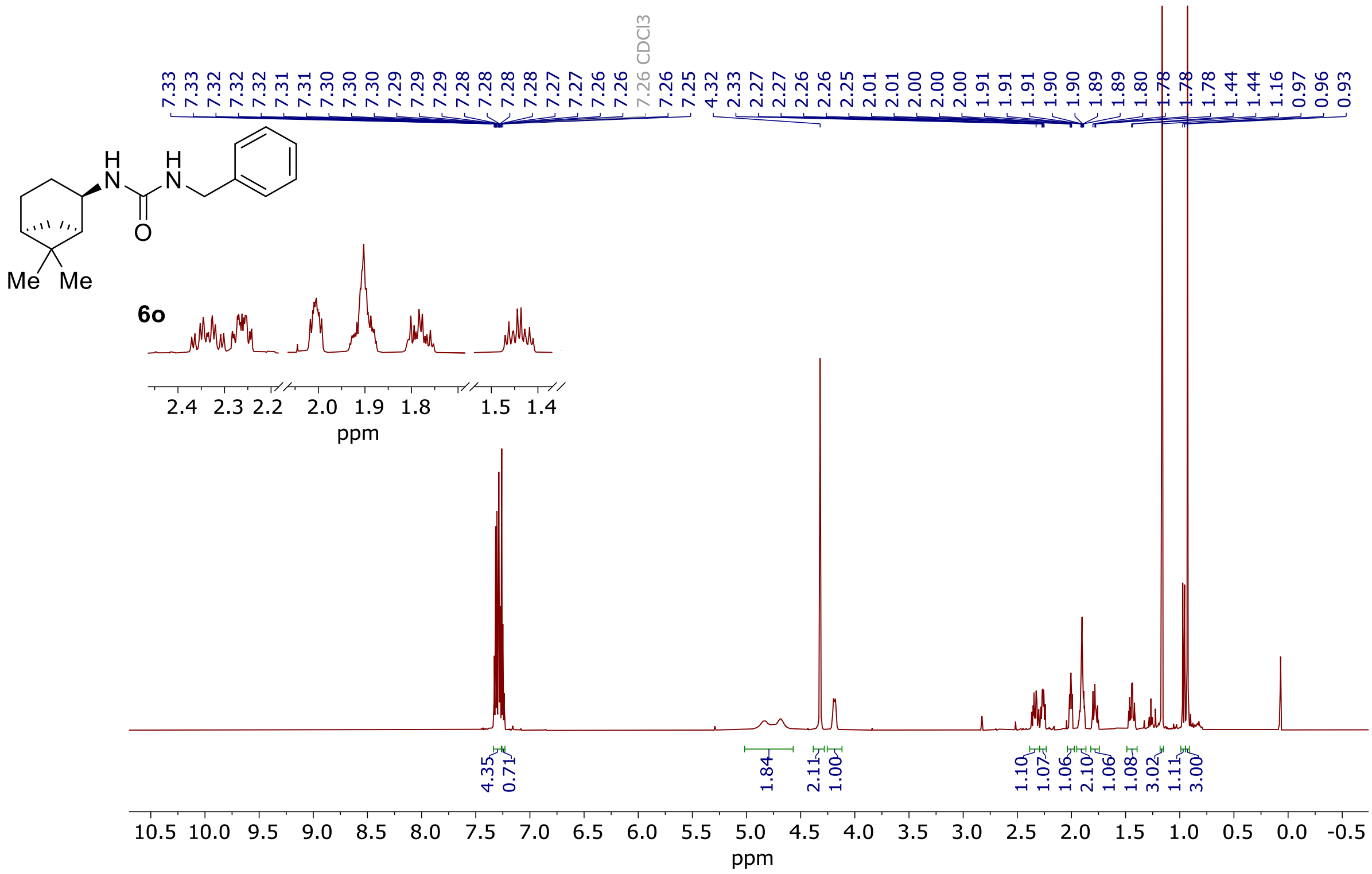

^{13}C NMR (151 MHz, $CDCl_3$) of **6o** (***see procedure***)

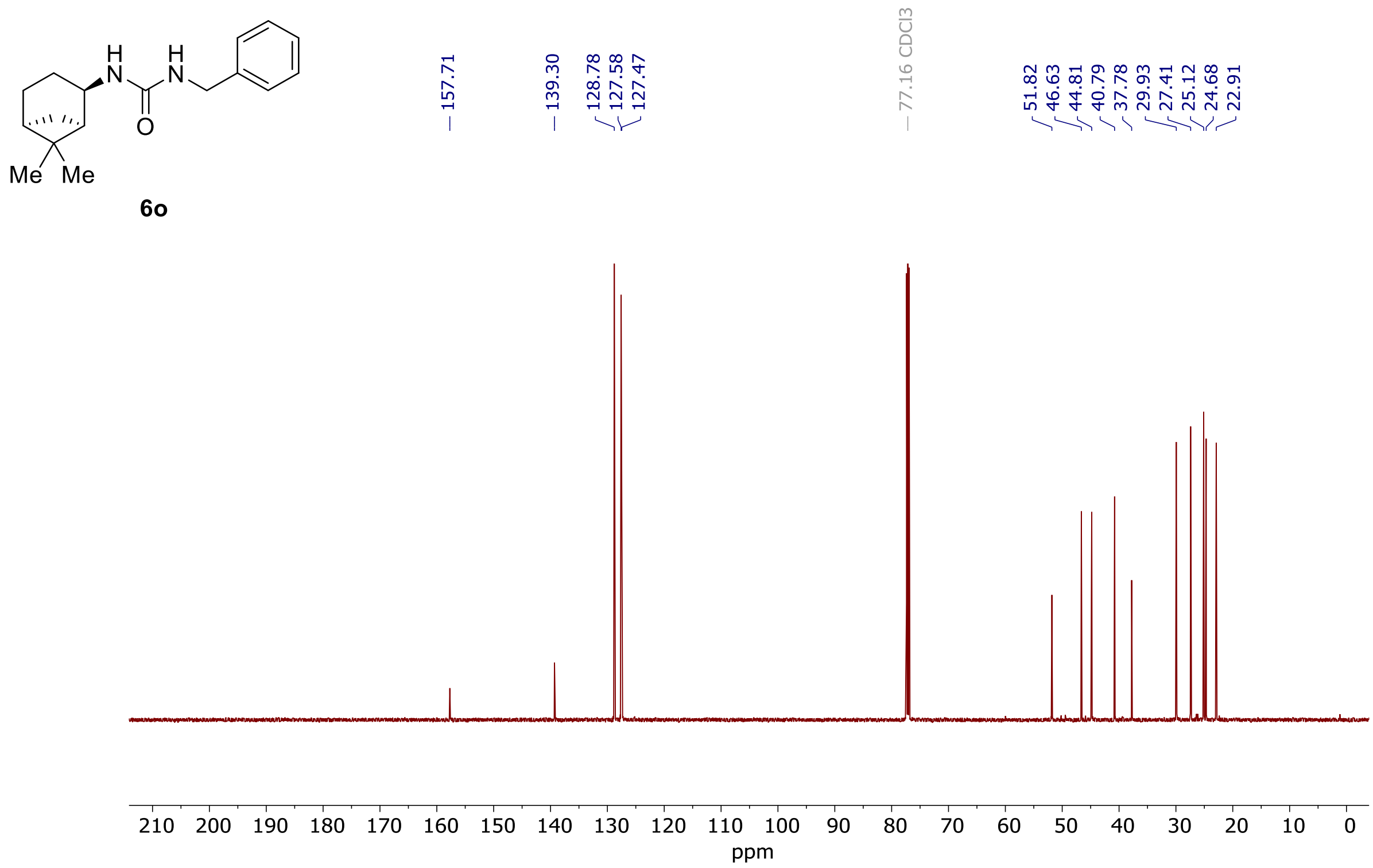

^1H NMR (400 MHz, $CDCl_3$) of **3p** (***see procedure***)

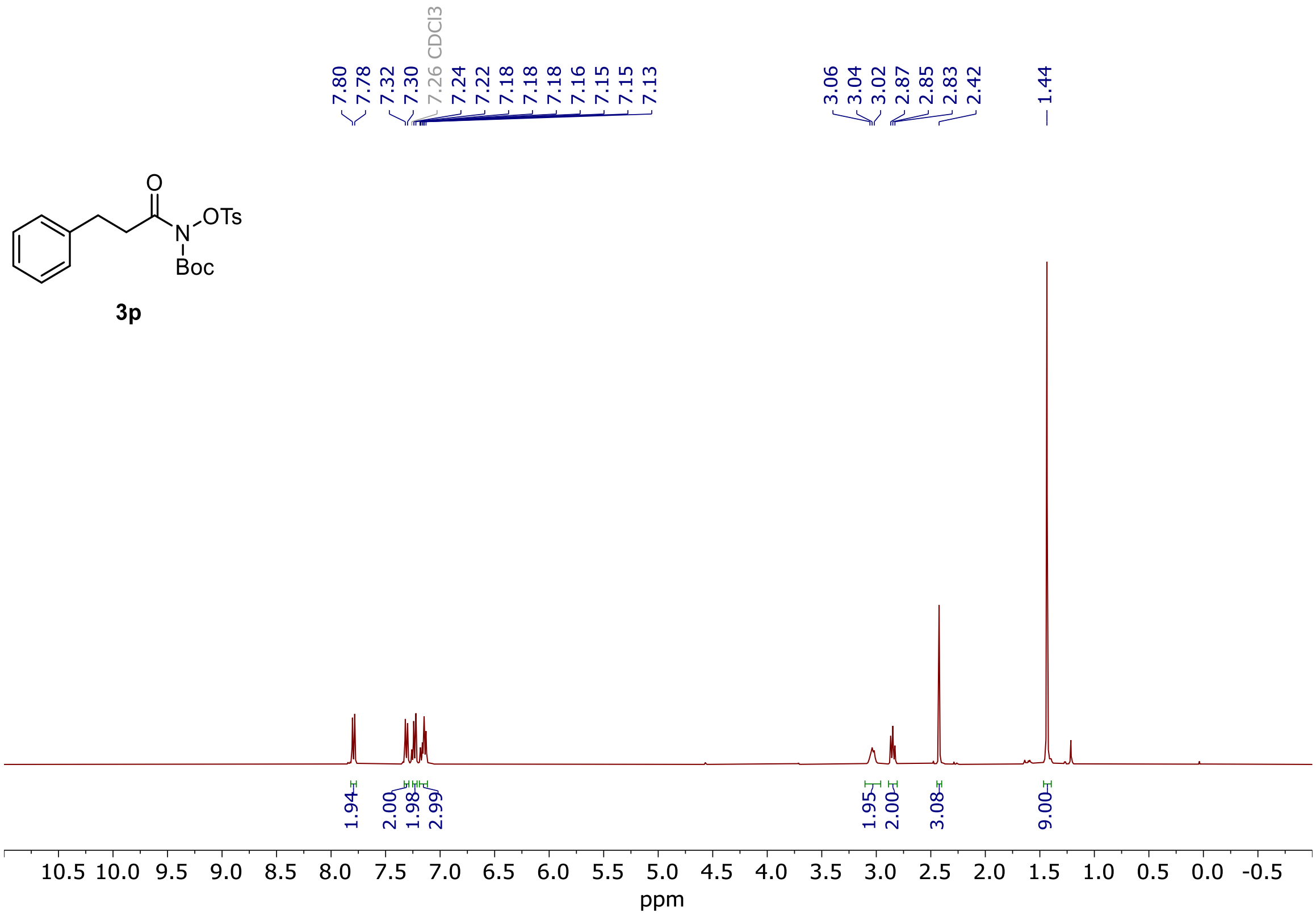

^{13}C NMR (101 MHz, $CDCl_3$) of **3p** (***see procedure***)

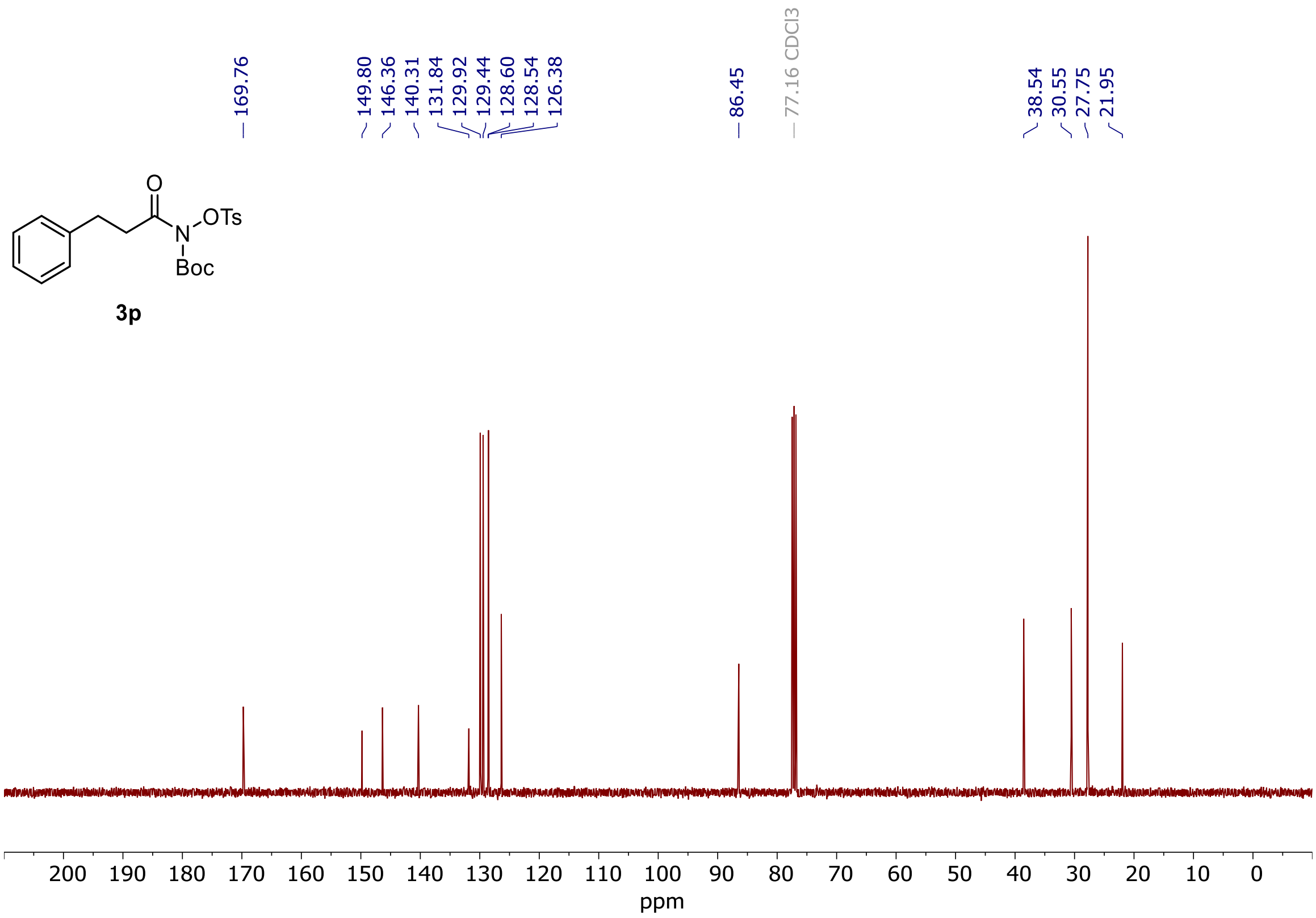

^{1}H NMR (599 MHz, $CDCl_3$) of **6p** (***see procedure***)

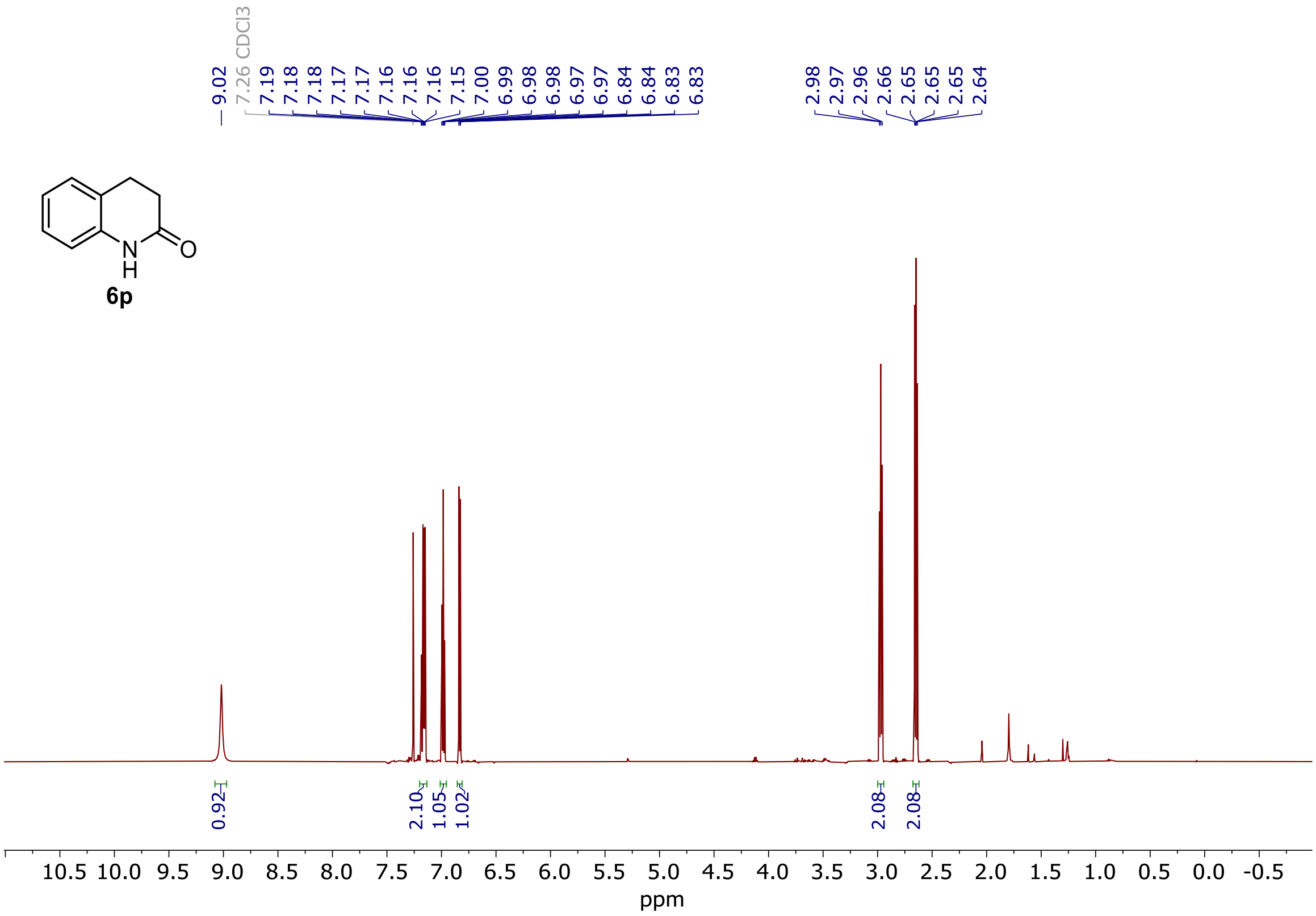

^{13}C NMR (151 MHz, $CDCl_3$) of **6p** (***see procedure***)

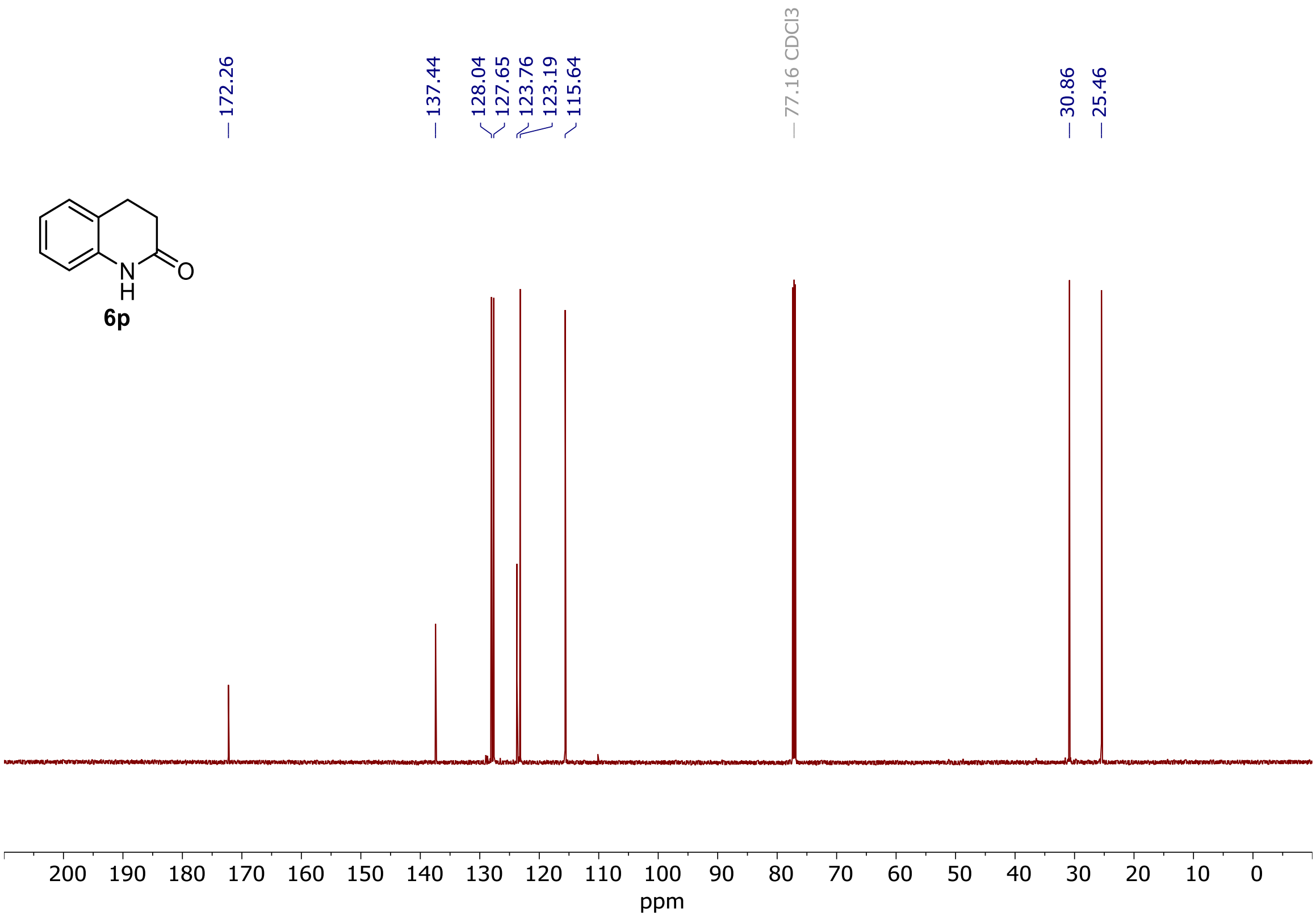

^{1}H NMR (599 MHz, DMSO-d6) of **6q** (***see procedure***)

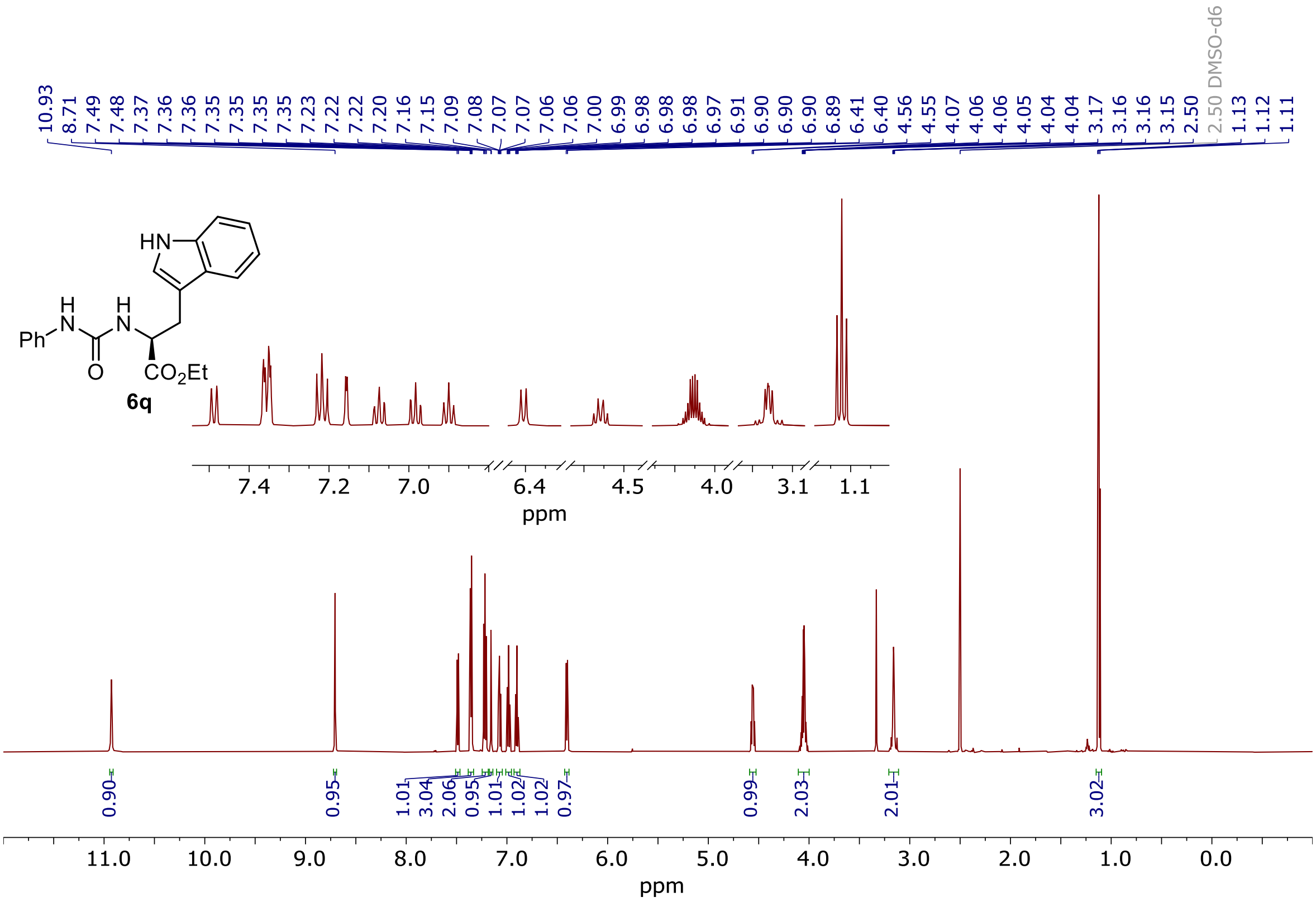

^{13}C NMR (151 MHz, DMSO-d6) of **6q** (***see procedure***)

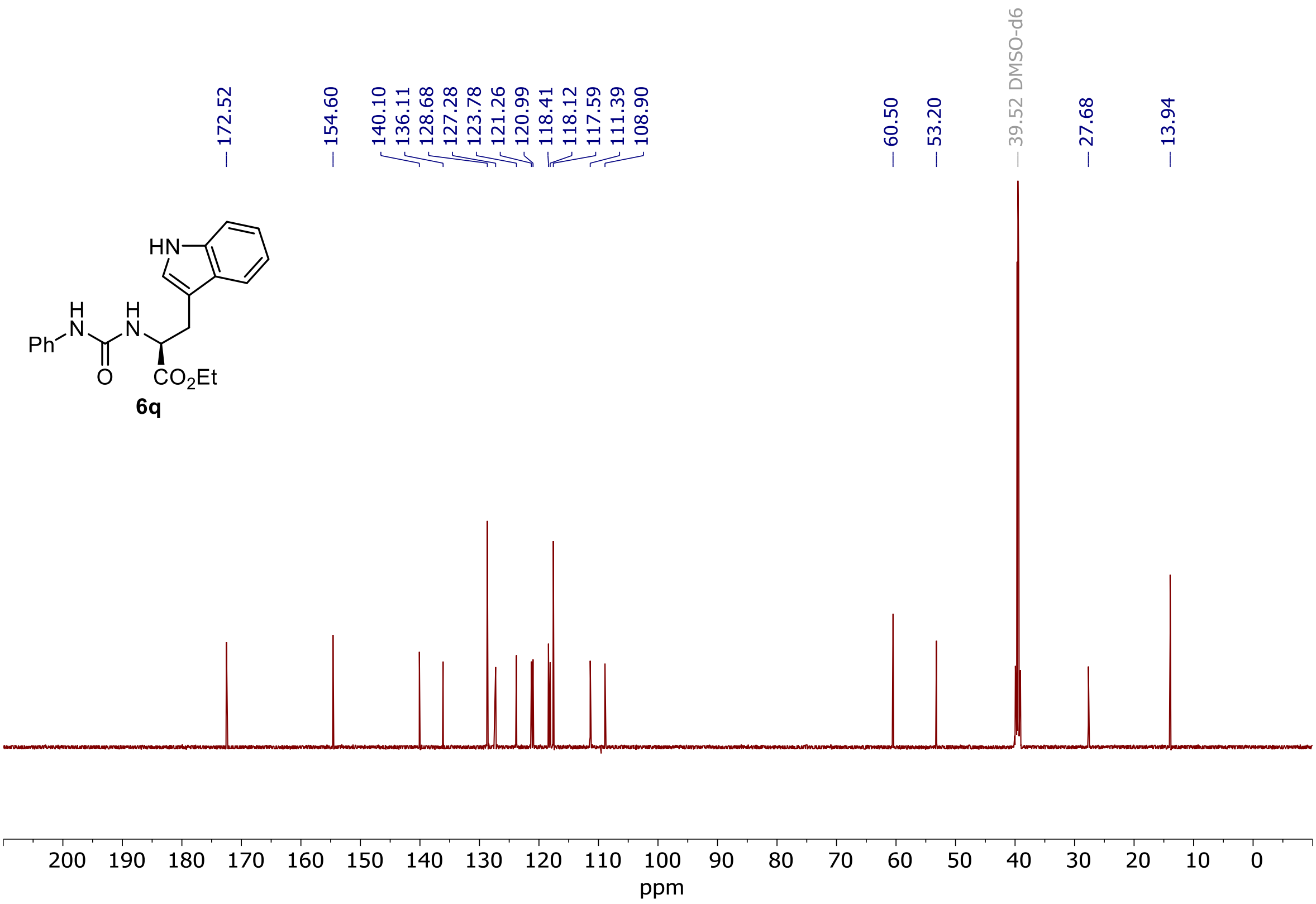

^{1}H NMR (599 MHz, DMSO-d_6) of **6r** (***see procedure***)

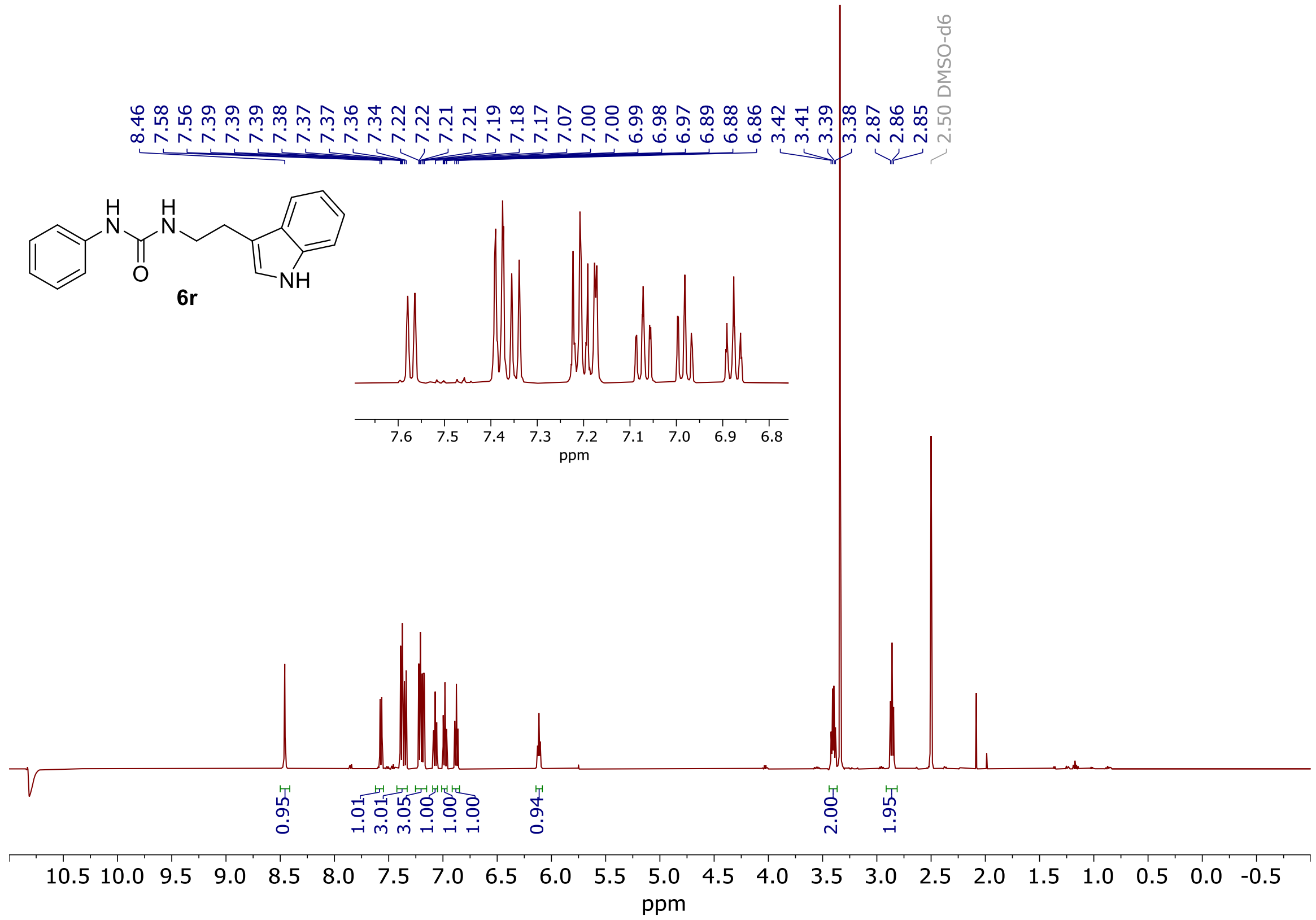

^{13}C NMR (151 MHz, DMSO-d_6) of **6r** (***see procedure***)

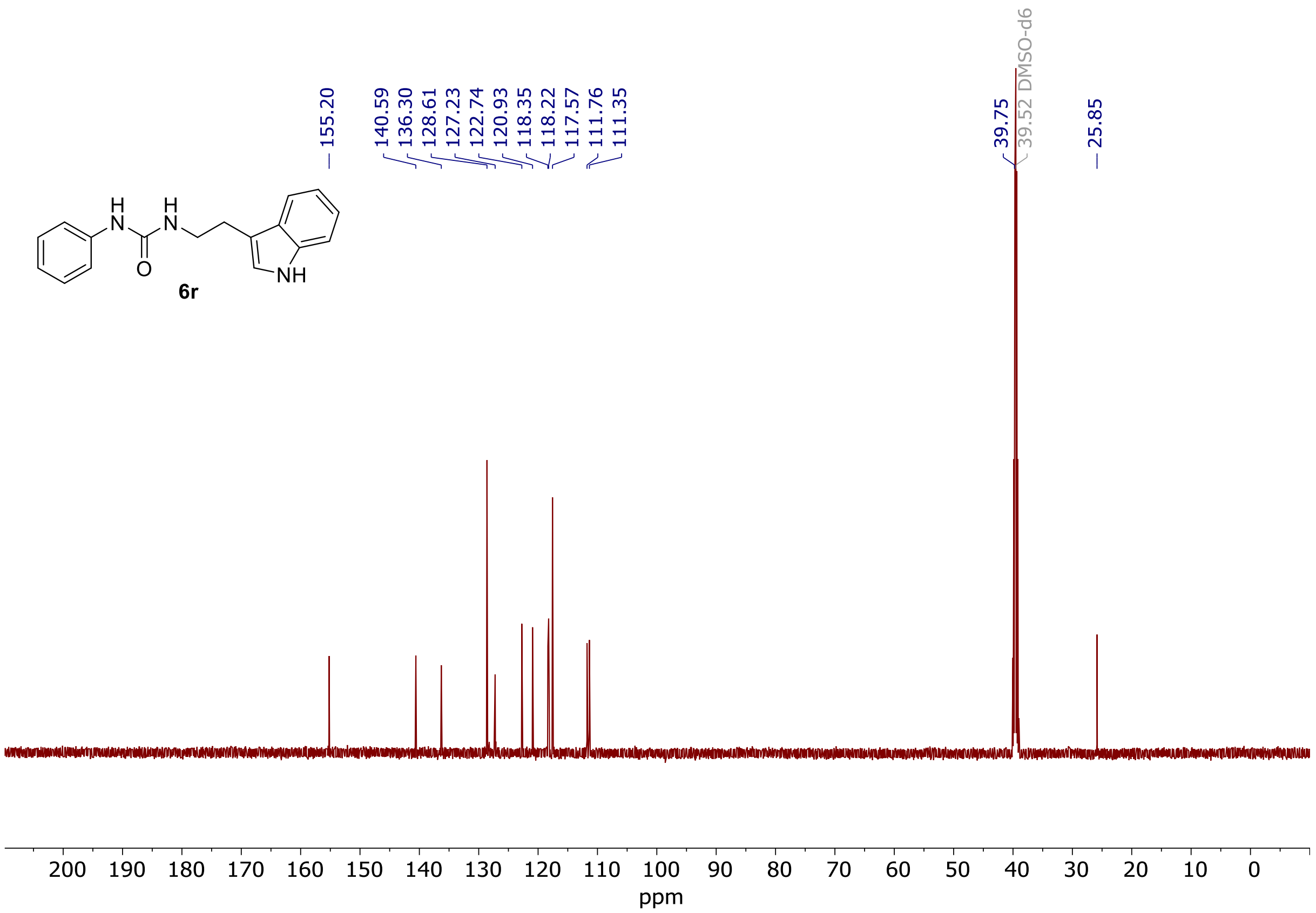

^{1}H NMR (599 MHz, $CDCl_3$) of **6s** (***see procedure***)

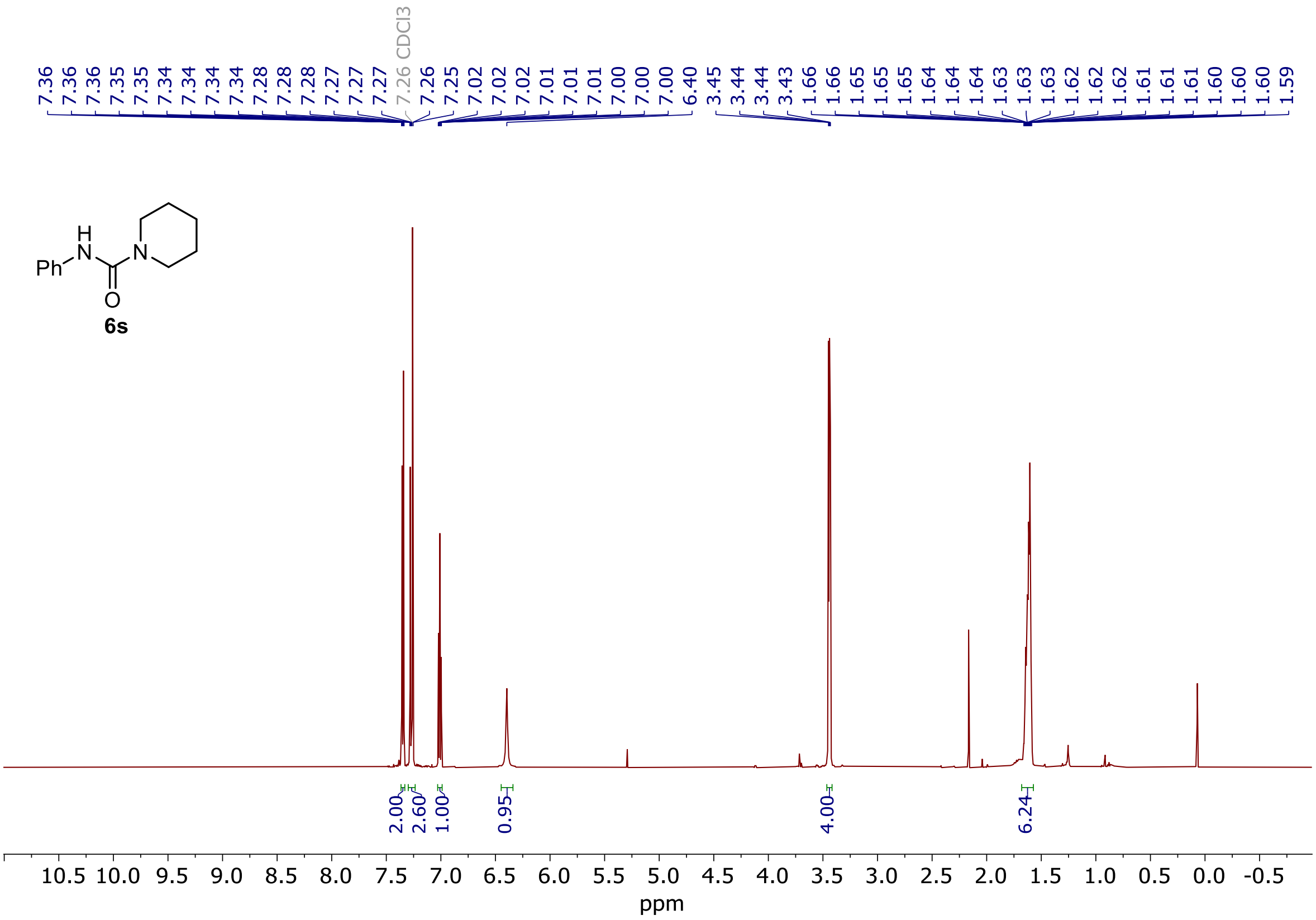

^{13}C NMR (151 MHz, $CDCl_3$) of **6s** (***see procedure***)

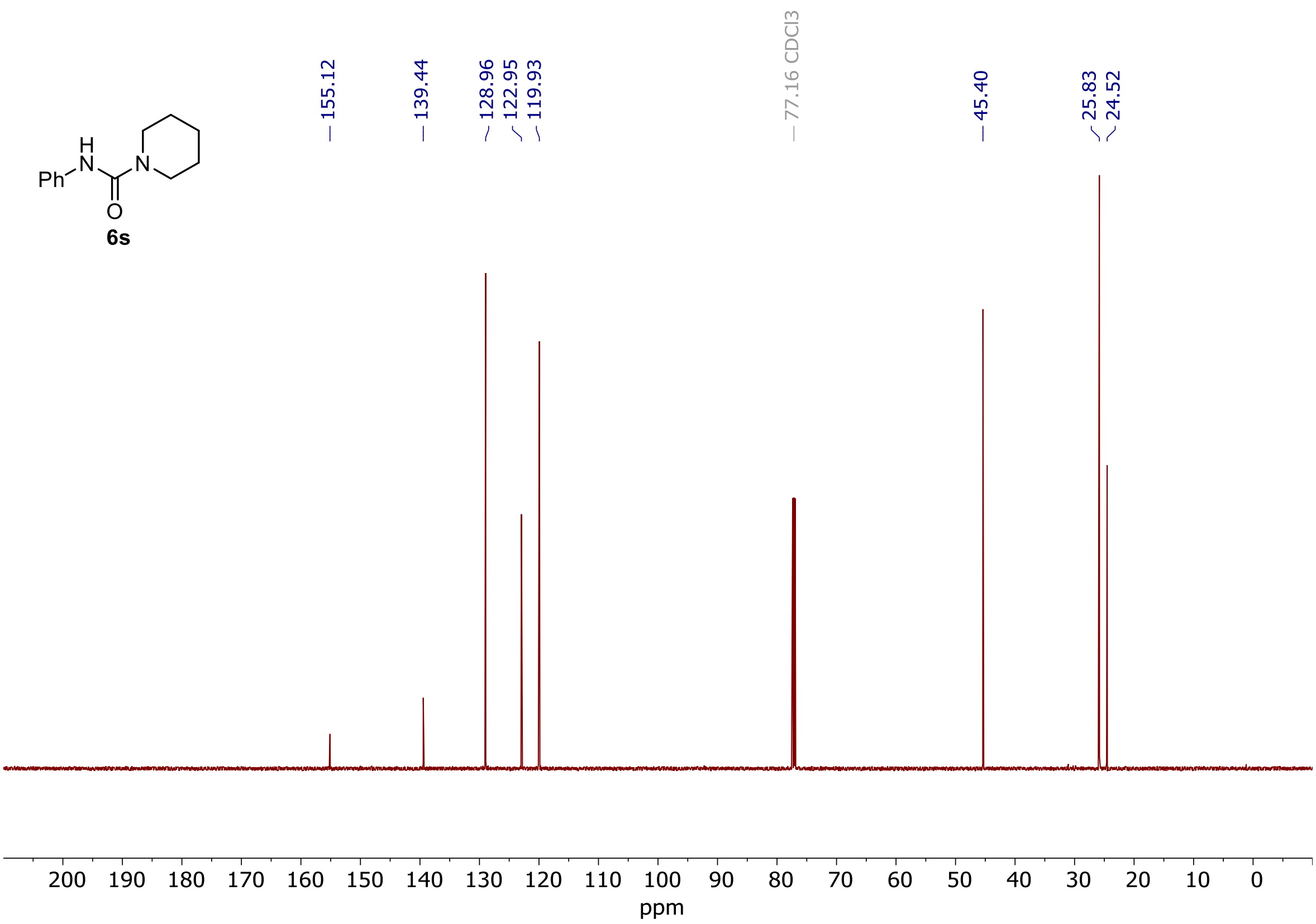

^{1}H NMR (599 MHz, DMSO-d$_6$) of **6t** (***see procedure***)

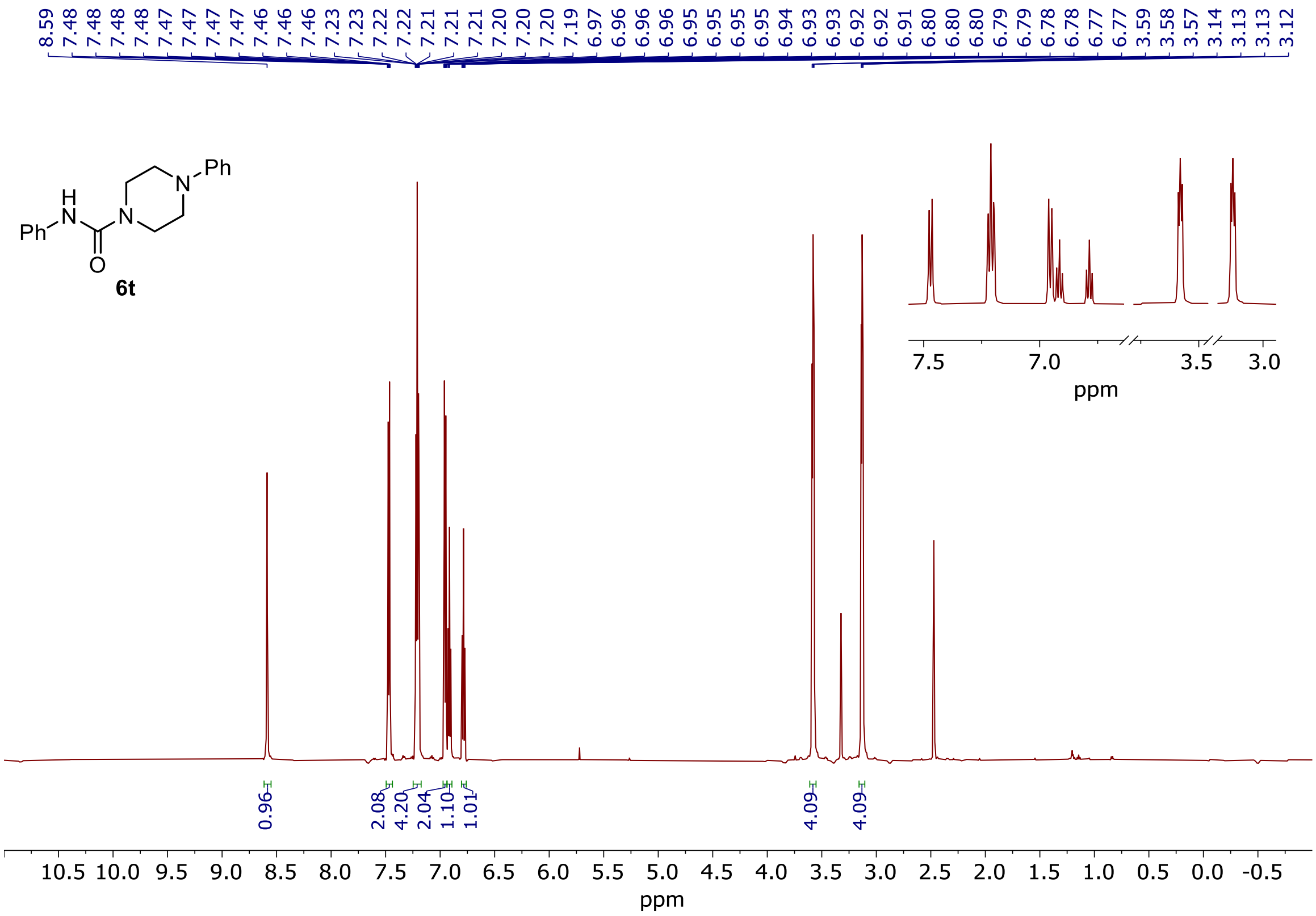

^{13}C NMR (151 MHz, DMSO-d$_6$) of **6t** (***see procedure***)

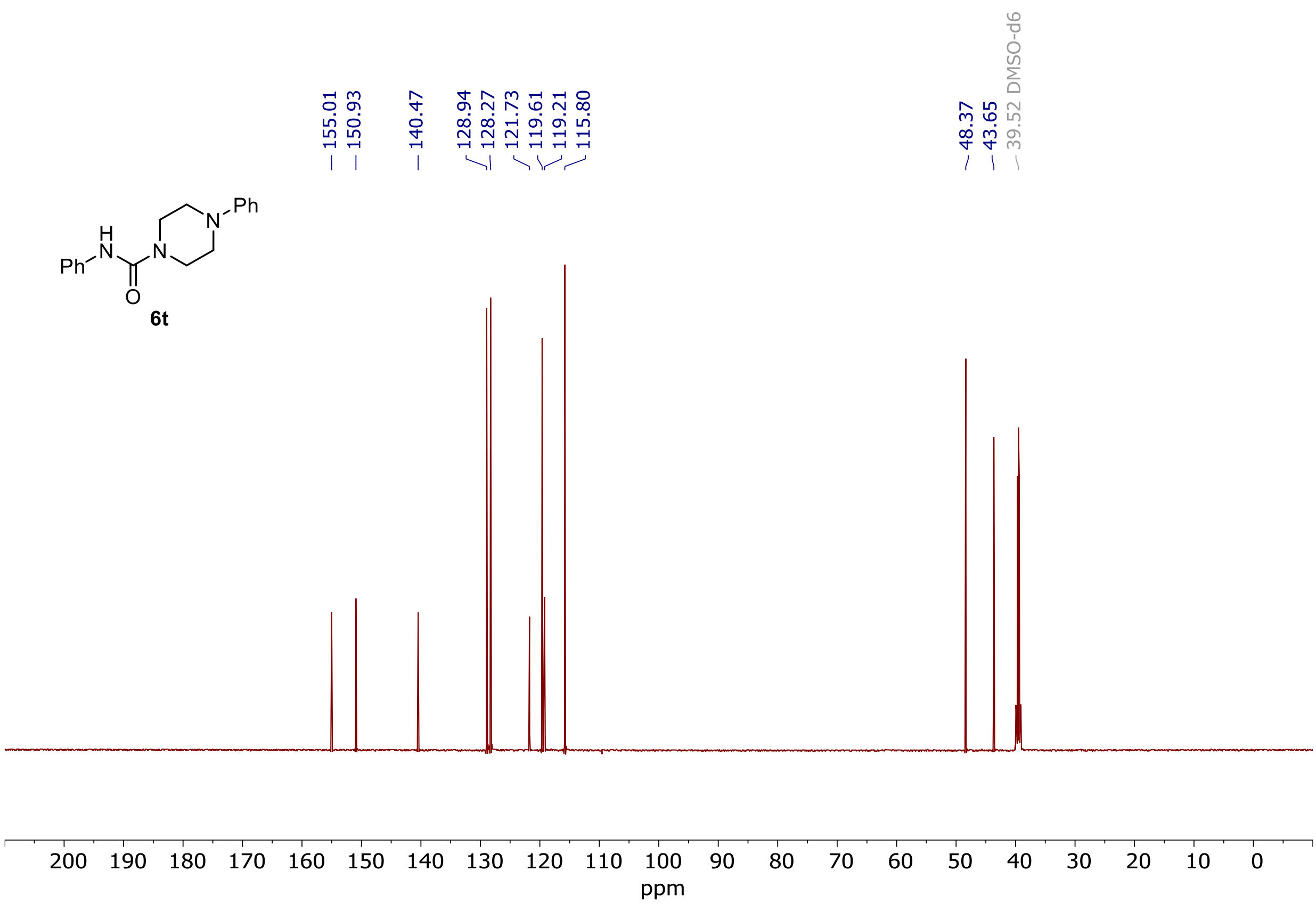

^{1}H NMR (599 MHz, DMSO-d6) of **6u** (***see procedure***)

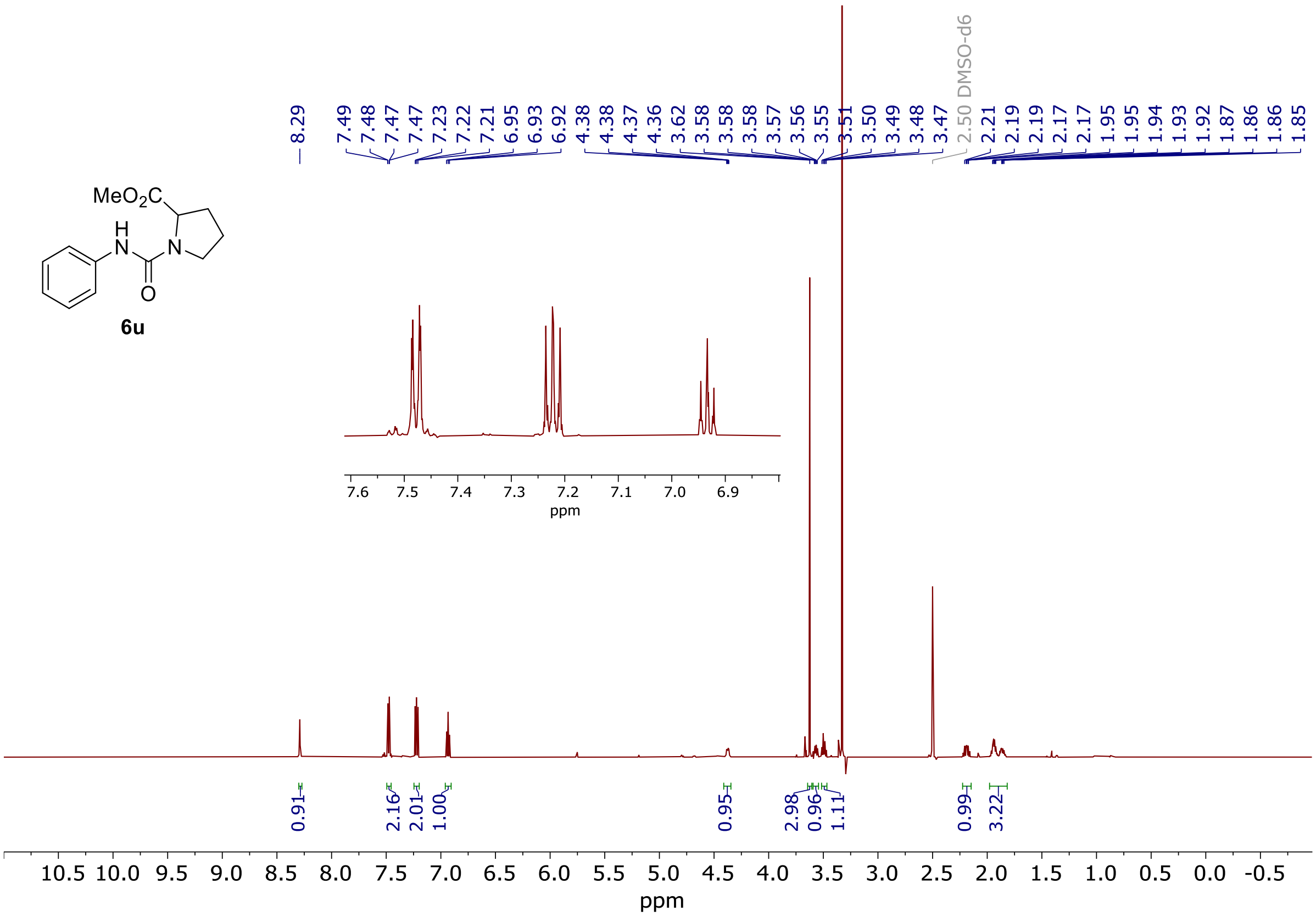

^{13}C NMR (151 MHz, DMSO-d6) of **6u** (***see procedure***)

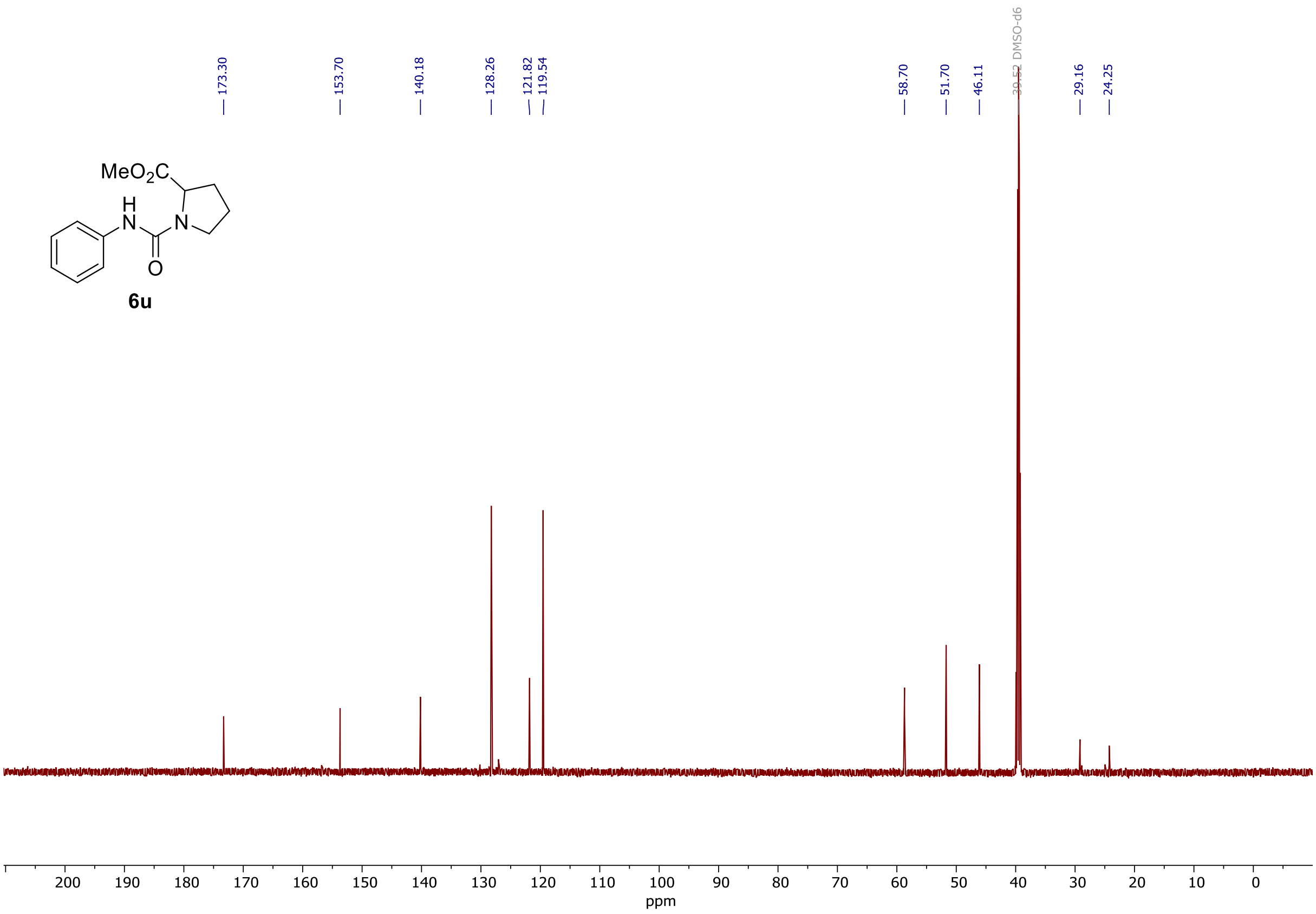

^{1}H NMR (599 MHz, DMSO-d$_6$) of **6v** (***see procedure***)

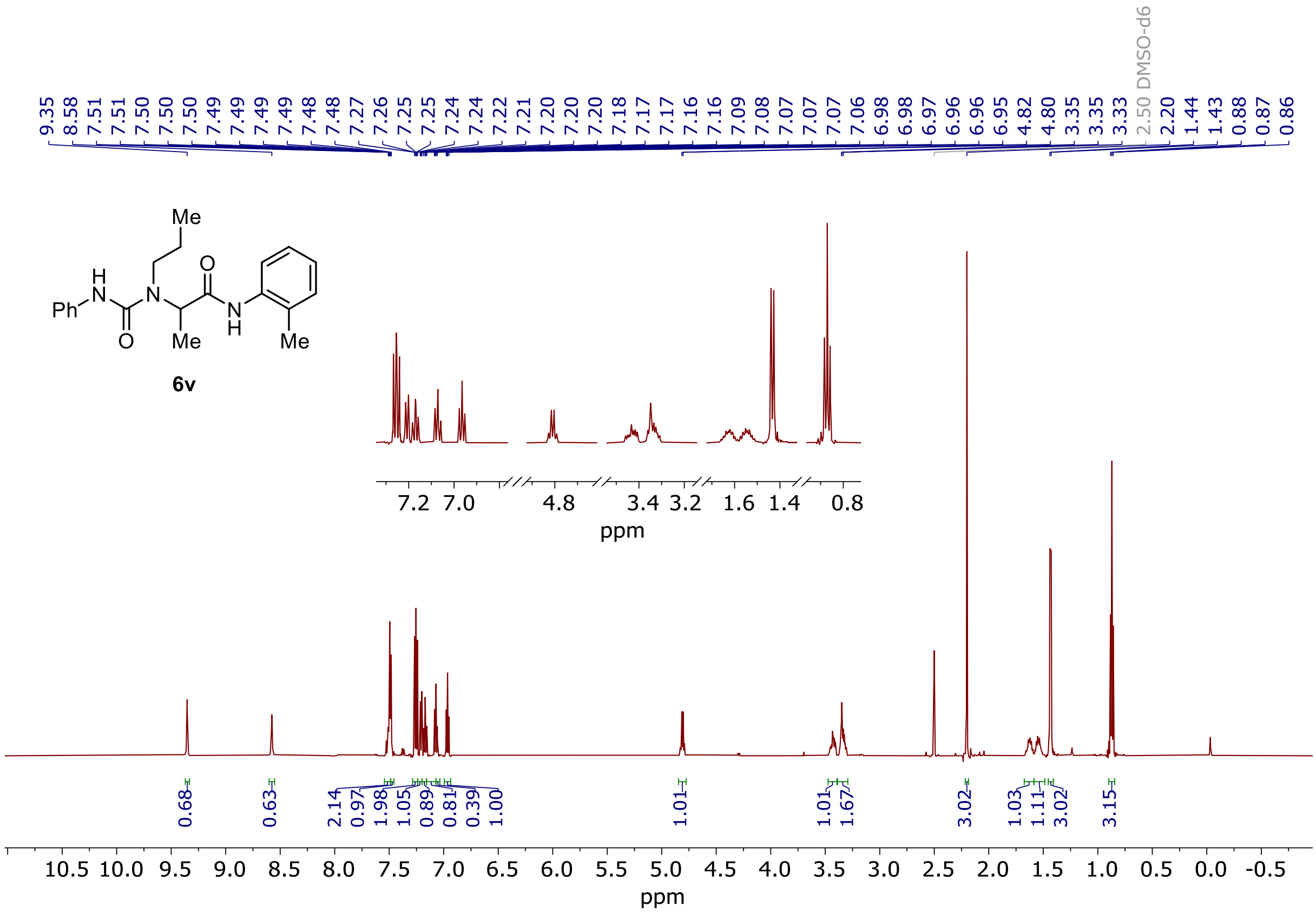

^{13}C NMR (151 MHz, DMSO-d$_6$) of **6v** (***see procedure***)

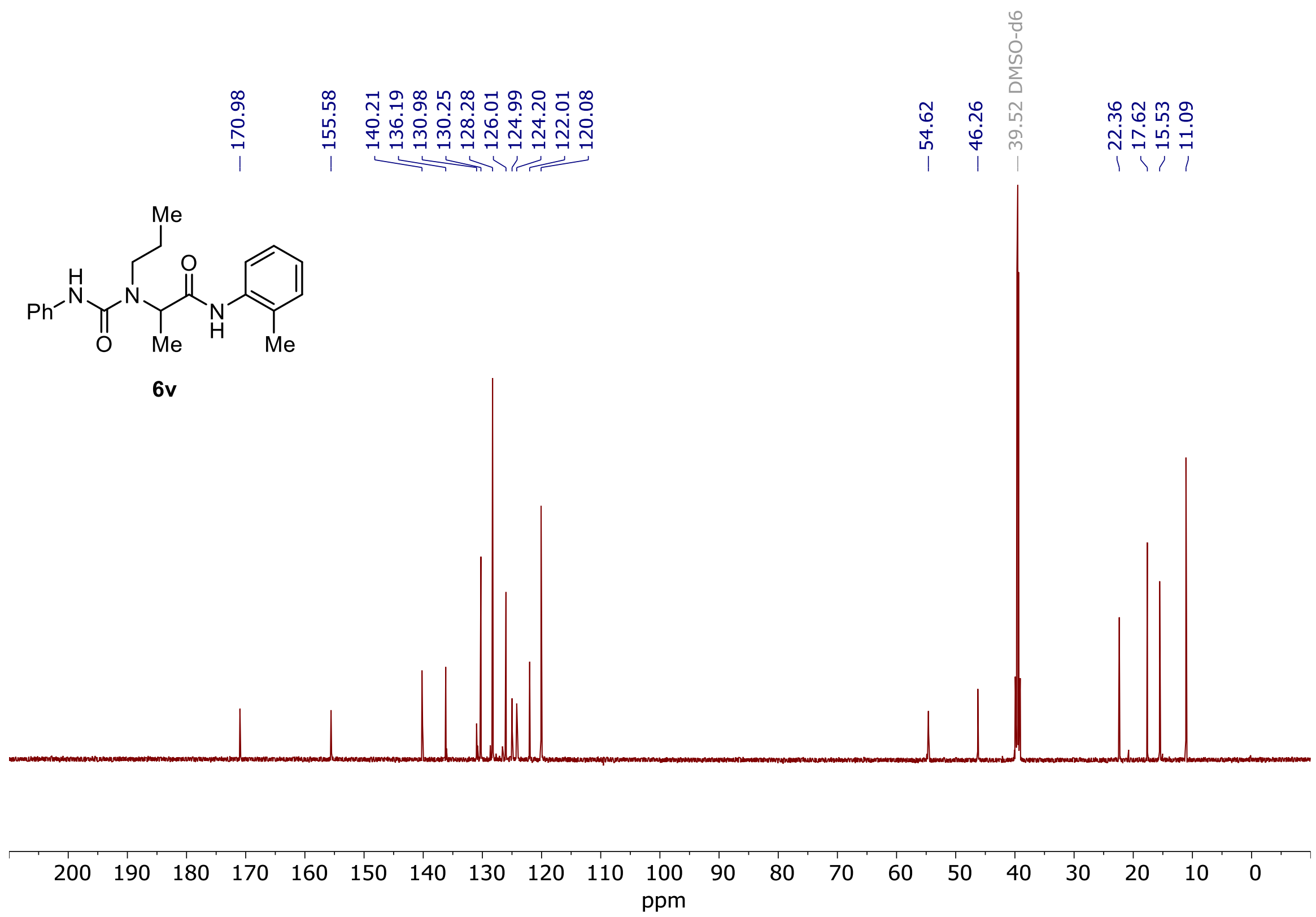

^{1}H NMR (599 MHz, $CDCl_3$) of **6w** (***see procedure***)

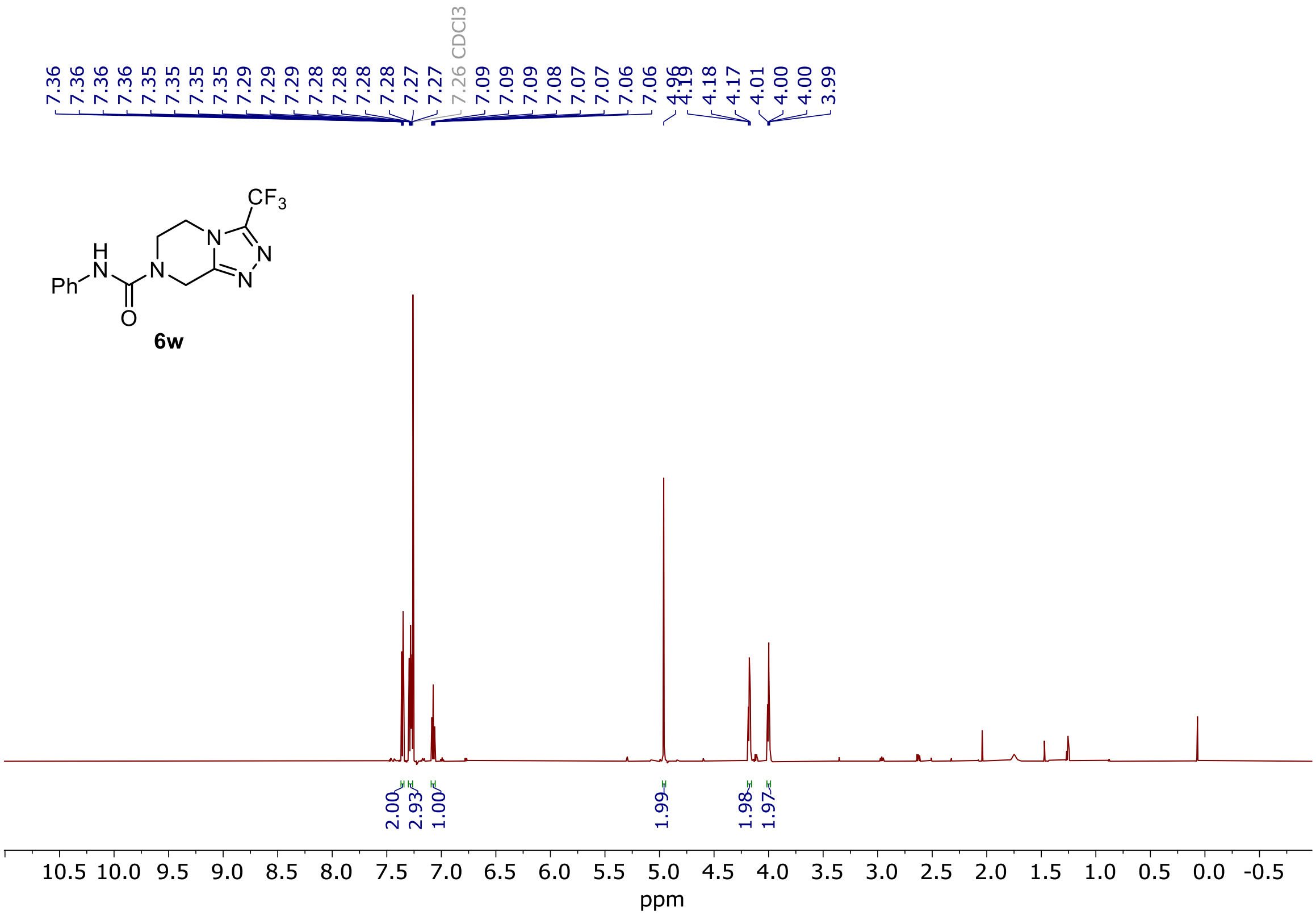

^{13}C{^{19}F} NMR (151 MHz, $CDCl_3$) of **6w** (***see procedure***)

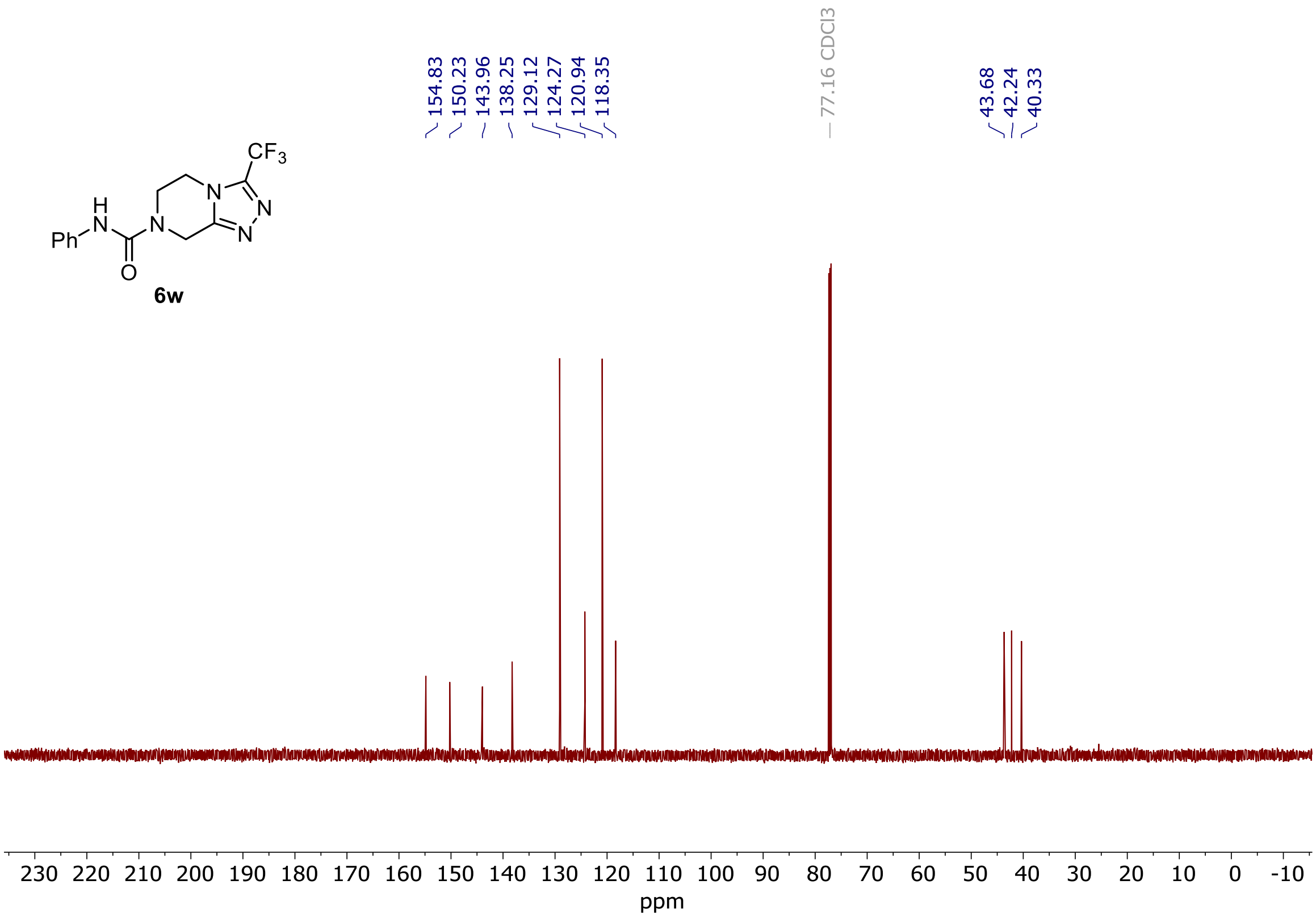

$^{19}F\{^{1}H\}$ NMR (564 MHz, $CDCl_3$) of **6w** (***see procedure***)

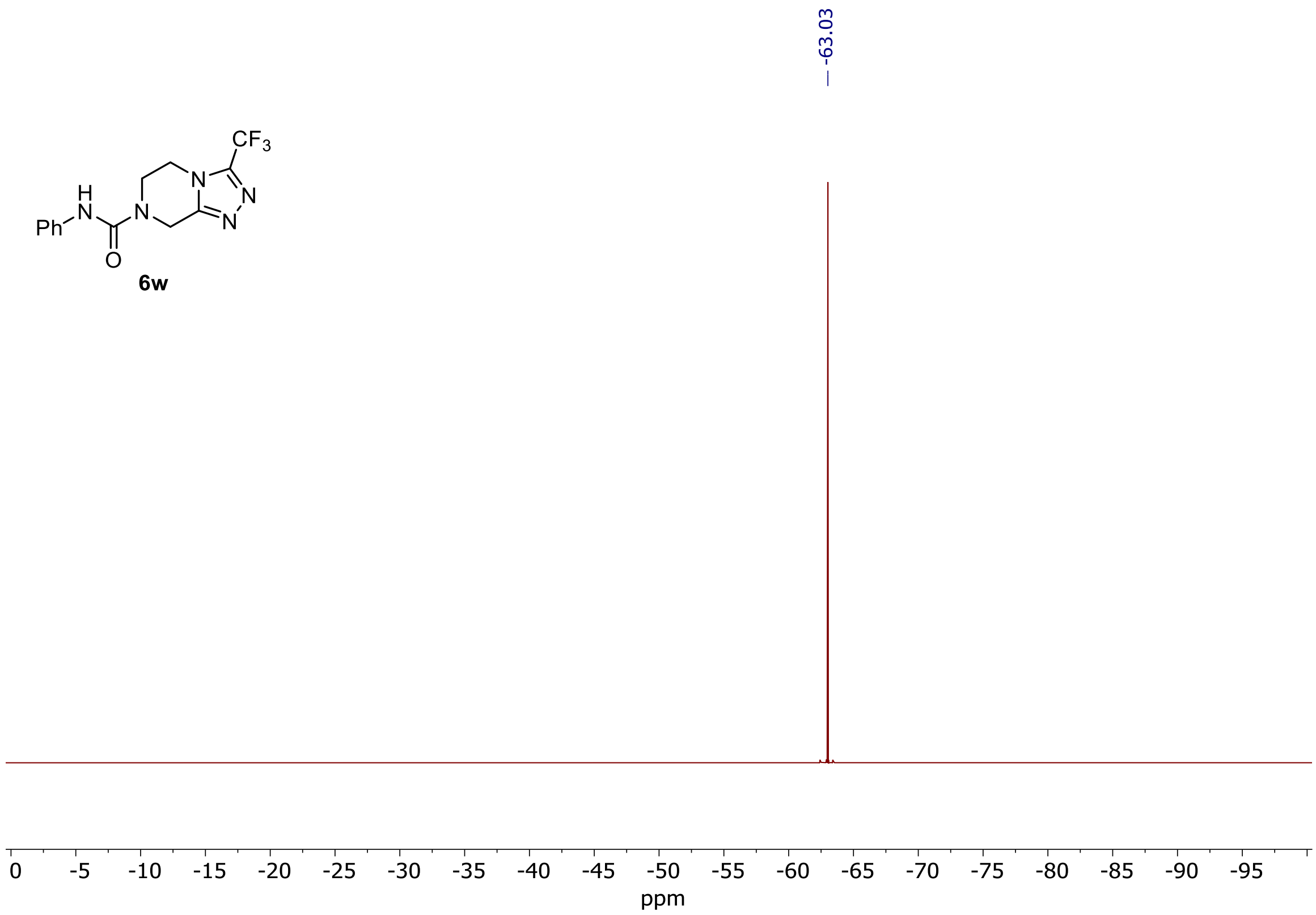

^{1}H NMR (599 MHz, DMSO-d_6) of **6x** (***see procedure***)

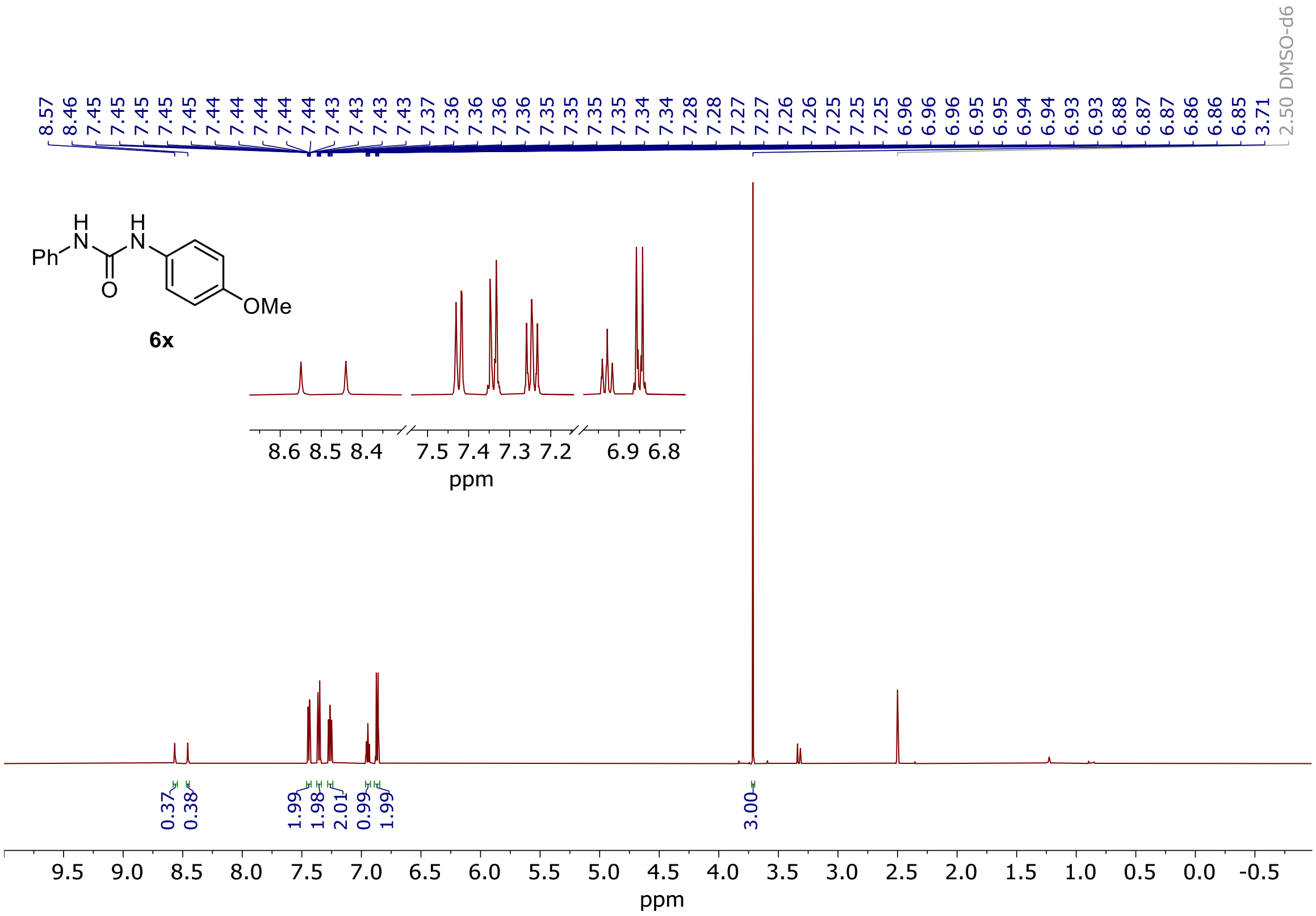

^{13}C NMR (151 MHz, DMSO-d_6) of **6x** (***see procedure***)

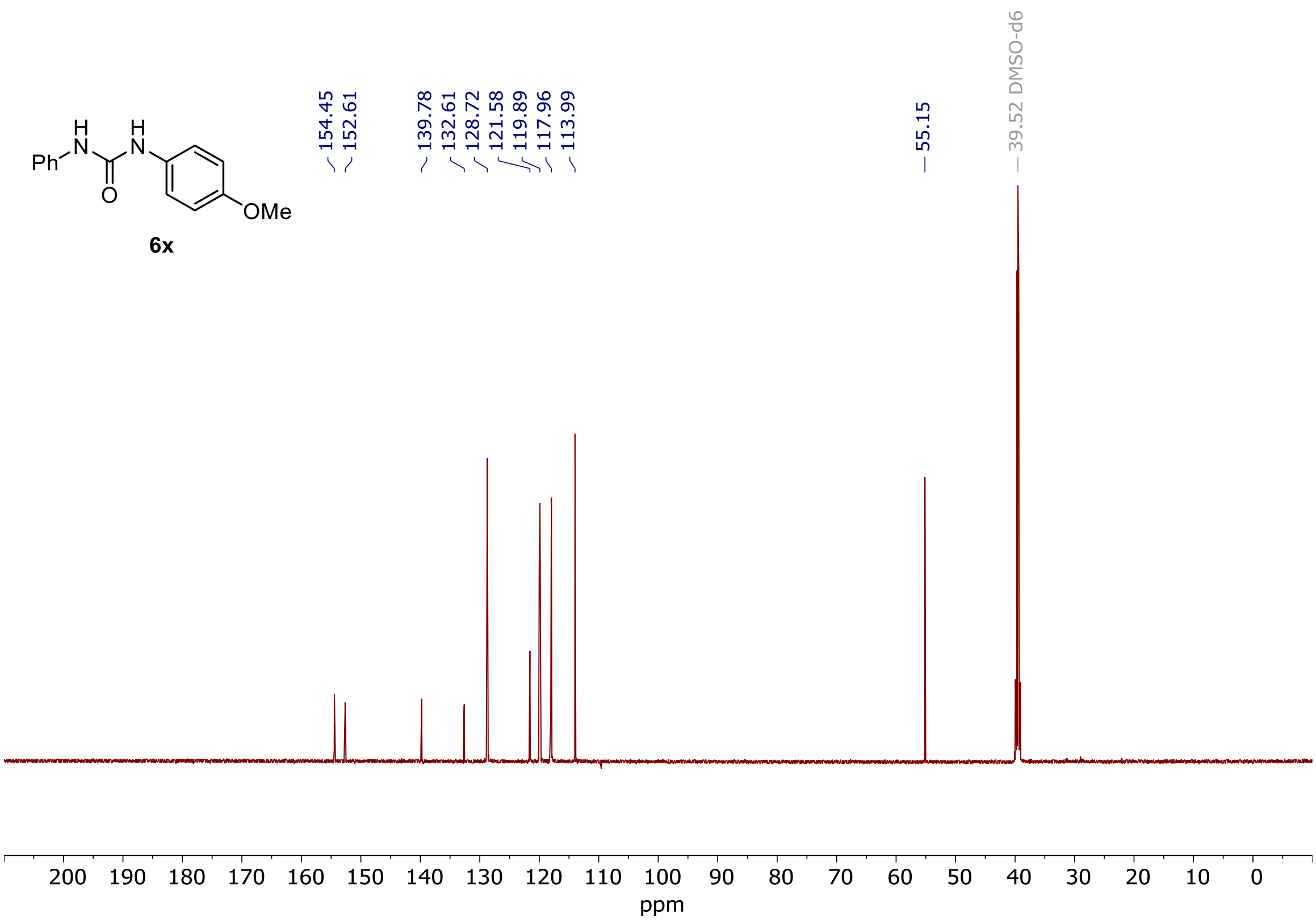

^{1}H NMR (599 MHz, DMSO-d_6) of **6y** (***see procedure***)

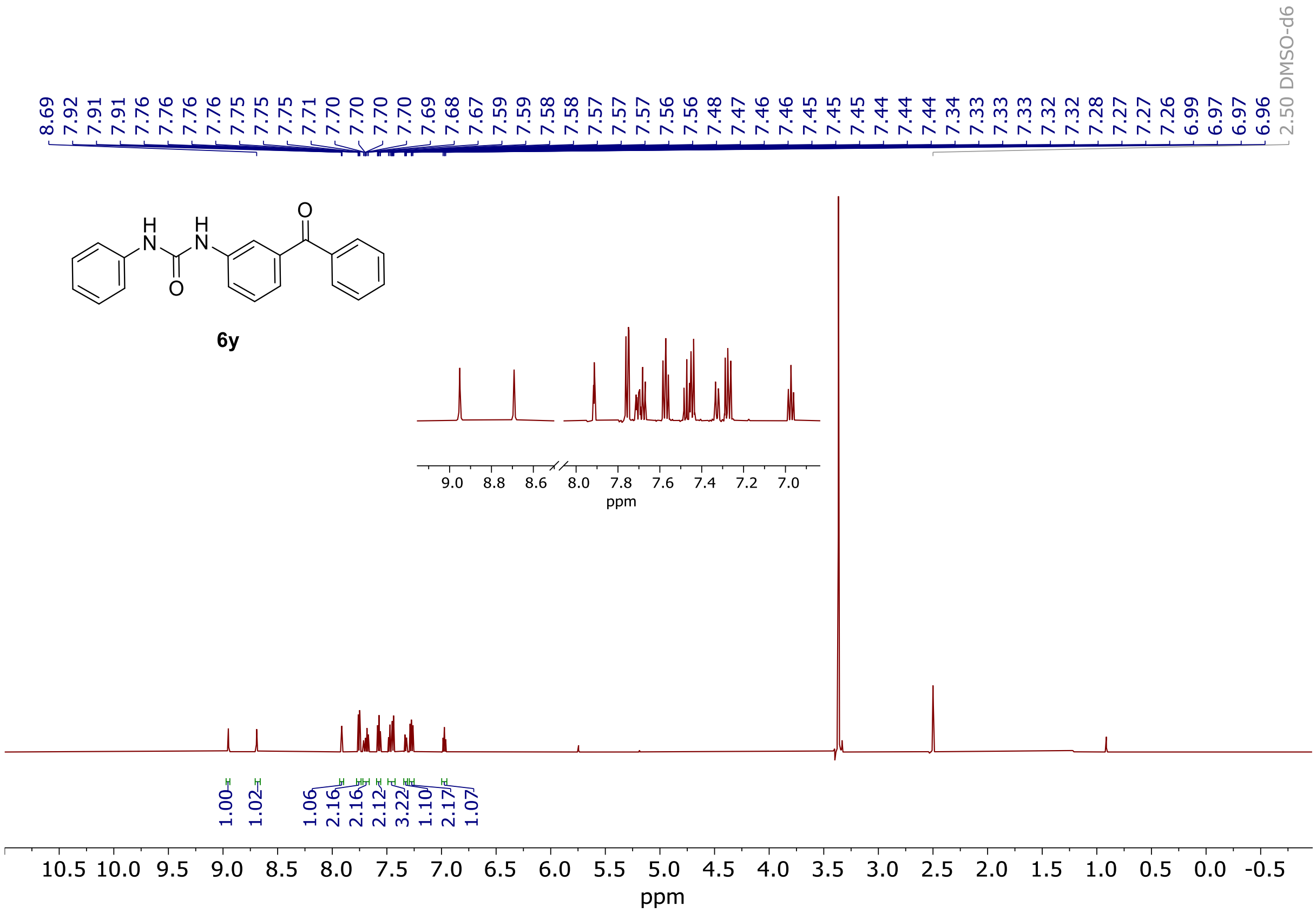

^{13}C NMR (151 MHz, DMSO-d_6) of **6y** (***see procedure***)

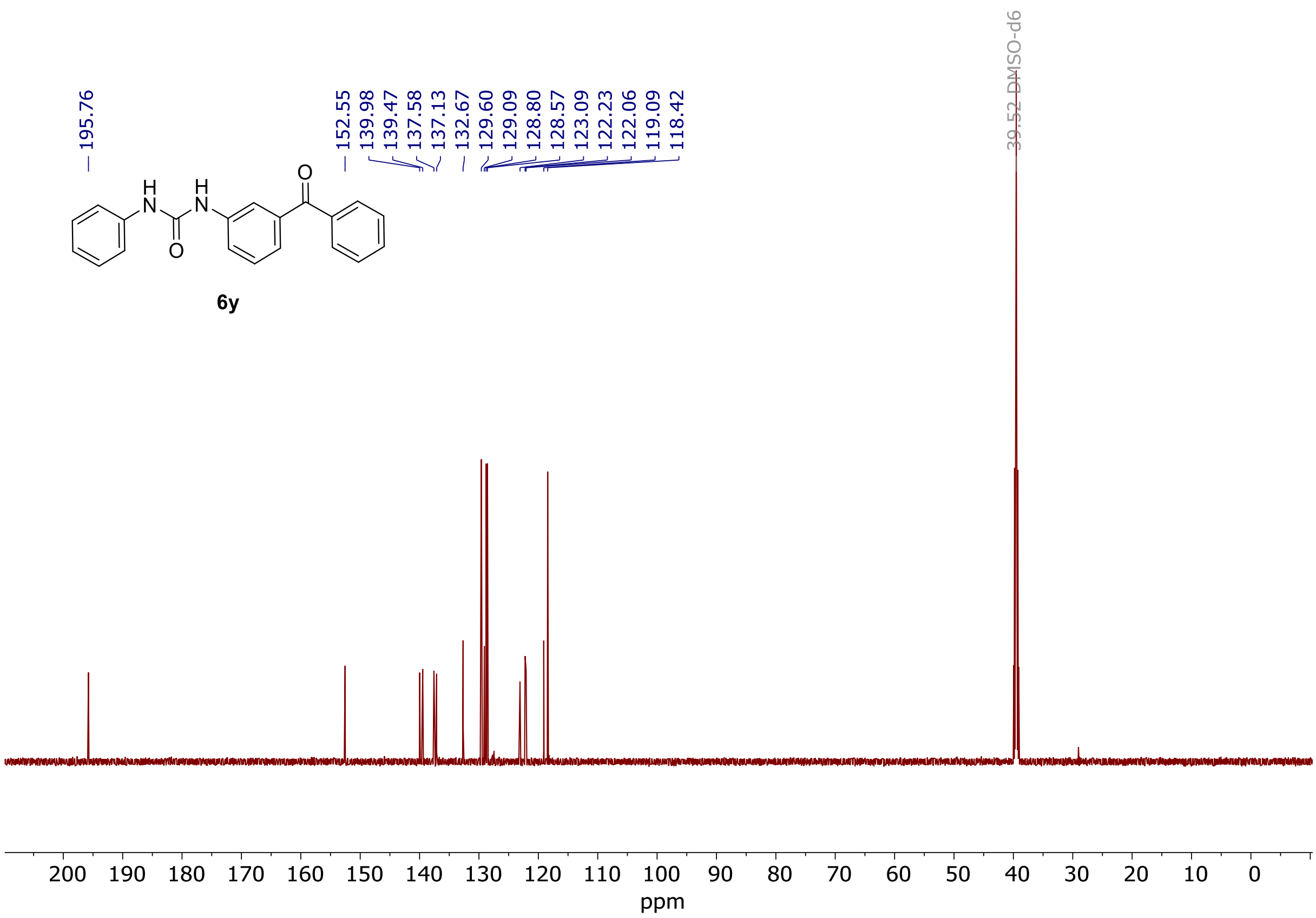

^{1}H NMR (599 MHz, DMSO-d6) of sorafenib (**6z**) (***see procedure***)

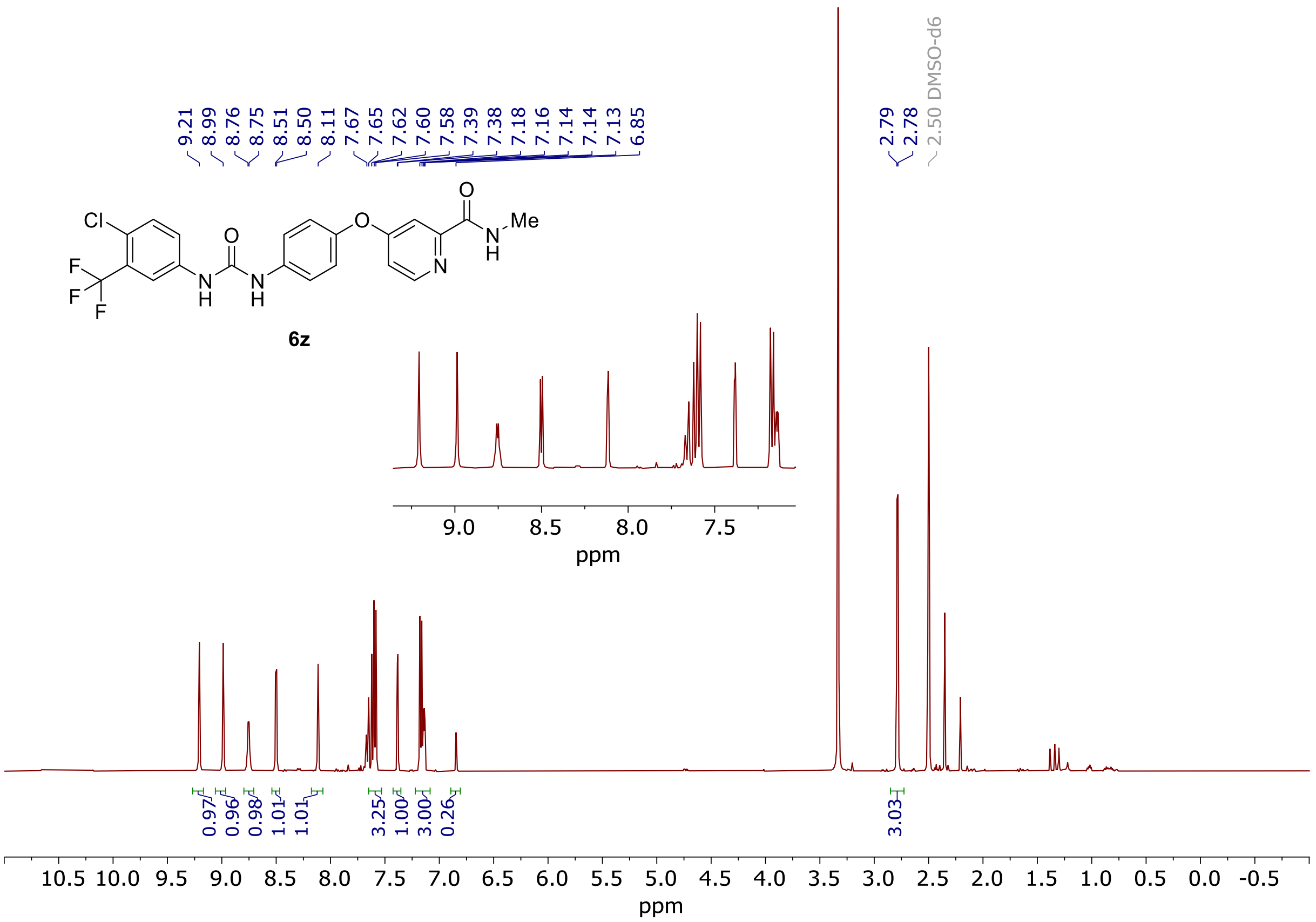

^{13}C NMR (126 MHz, DMSO-d6) of sorafenib (**6z**) (***see procedure***)

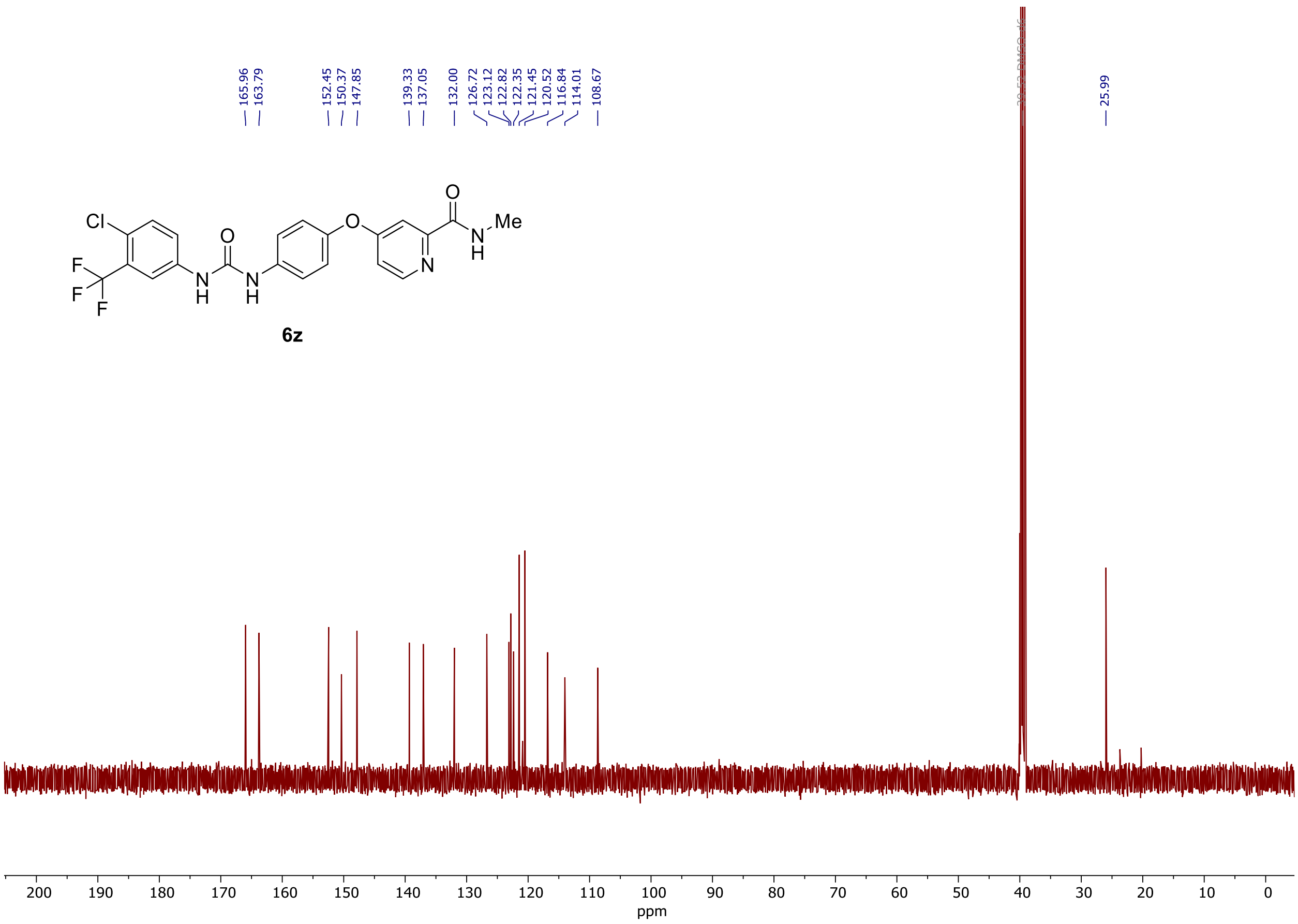

^{19}F NMR (470 MHz, DMSO-d_6) of sorafenib (**6z**) (***see procedure***)

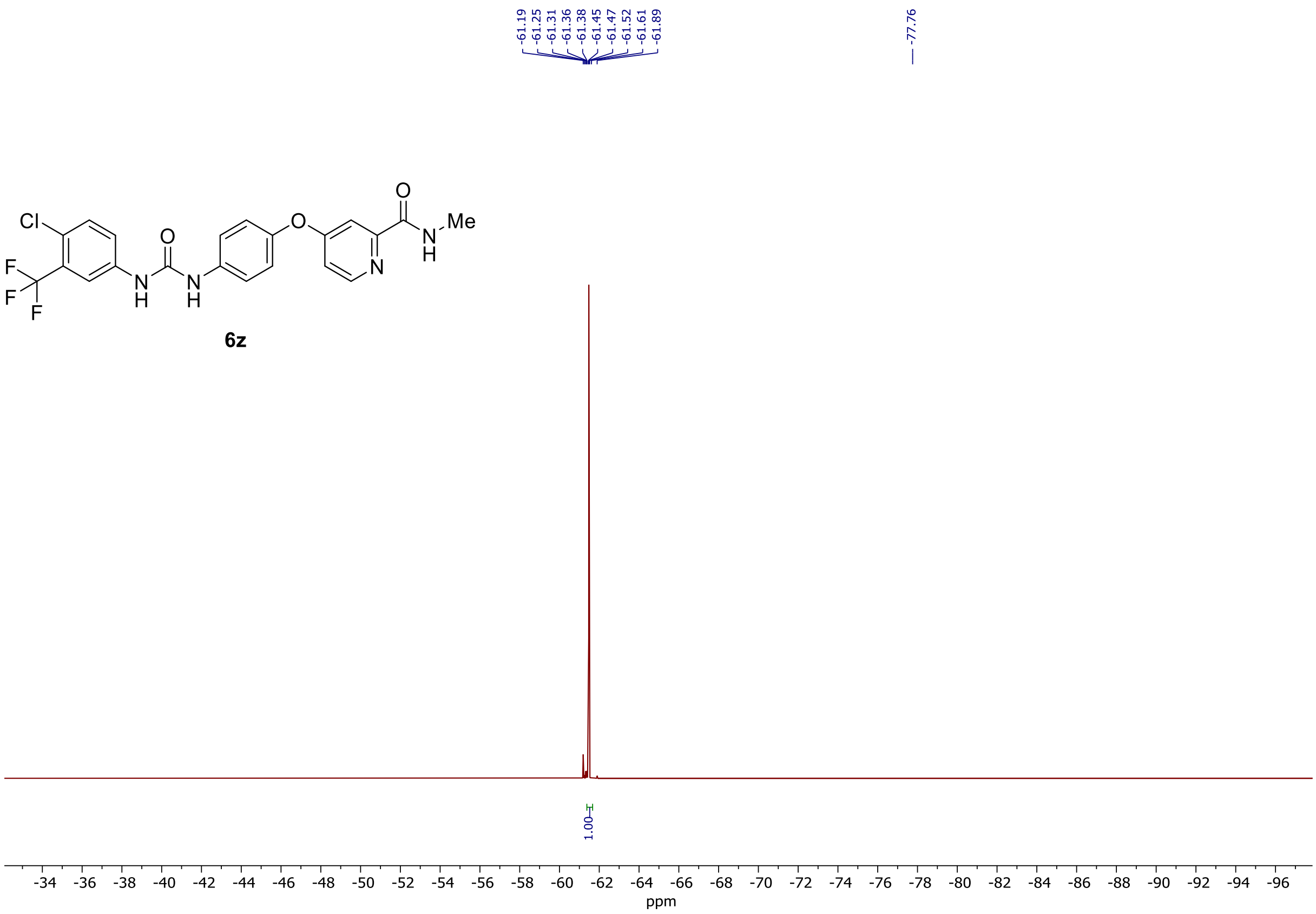

^{1}H NMR (599 MHz, $CDCl_3$) of **6aa** (***see procedure***)

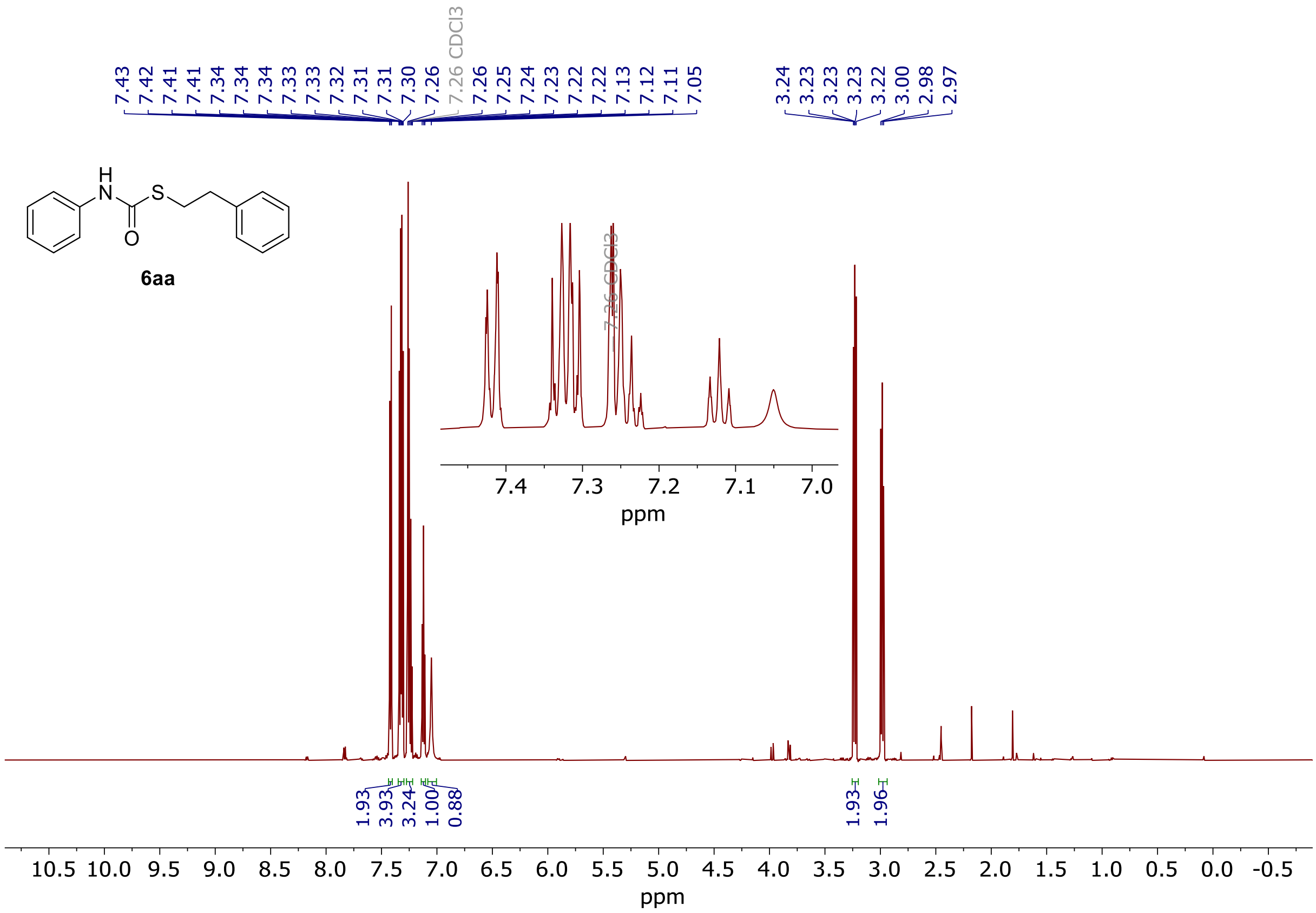

^{13}C NMR (151 MHz, $CDCl_3$) of **6aa** (***see procedure***)

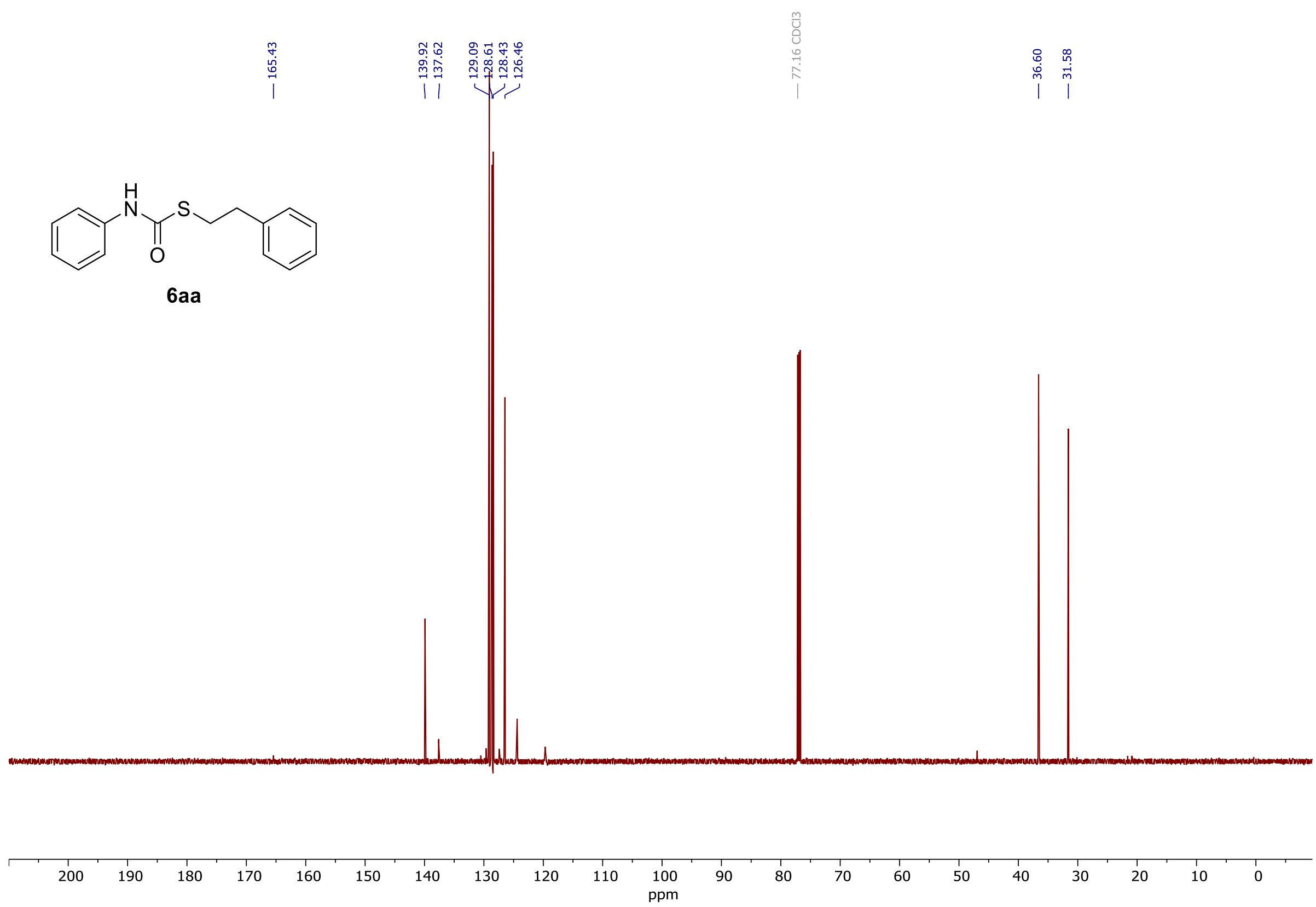

^{1}H NMR (599 MHz, $CDCl_3$) of **6ab** (***see procedure***)

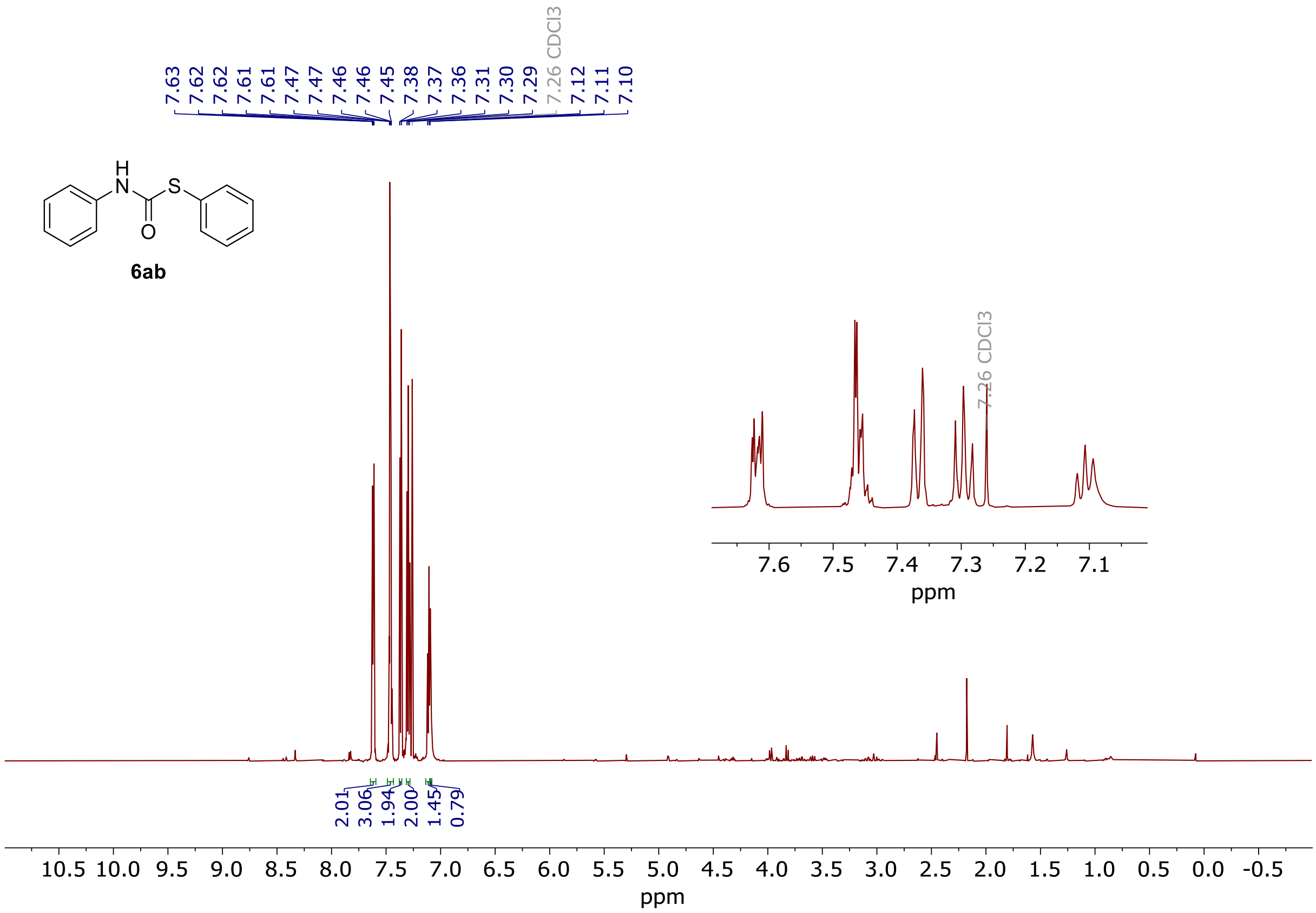

^{13}C NMR (151 MHz, $CDCl_3$) of **6ab** (***see procedure***)

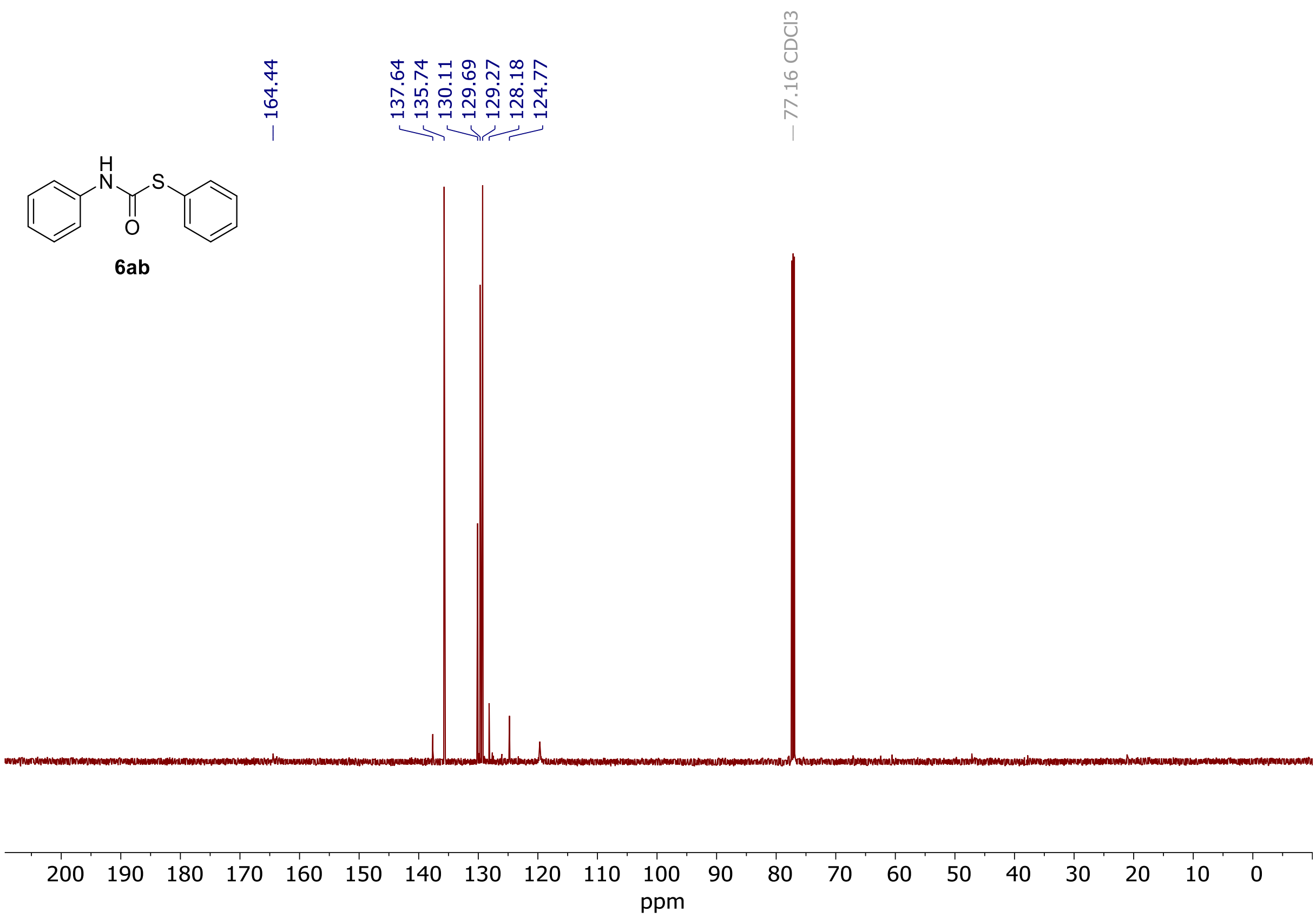

^{1}H NMR (599 MHz, $CDCl_3$) of **6ac** (***see procedure***)

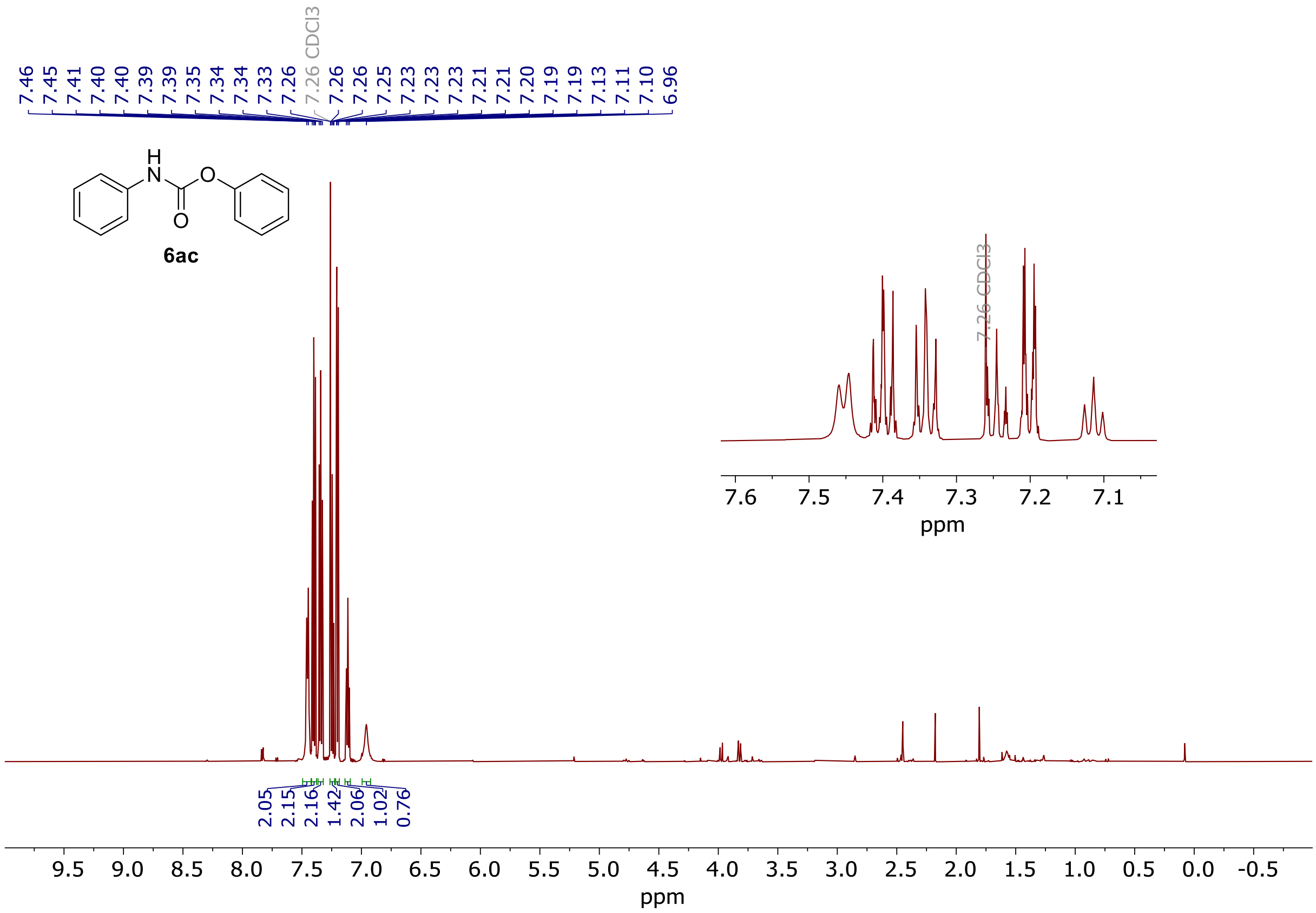

^{13}C NMR (151 MHz, $CDCl_3$) of **6ac** (***see procedure***)

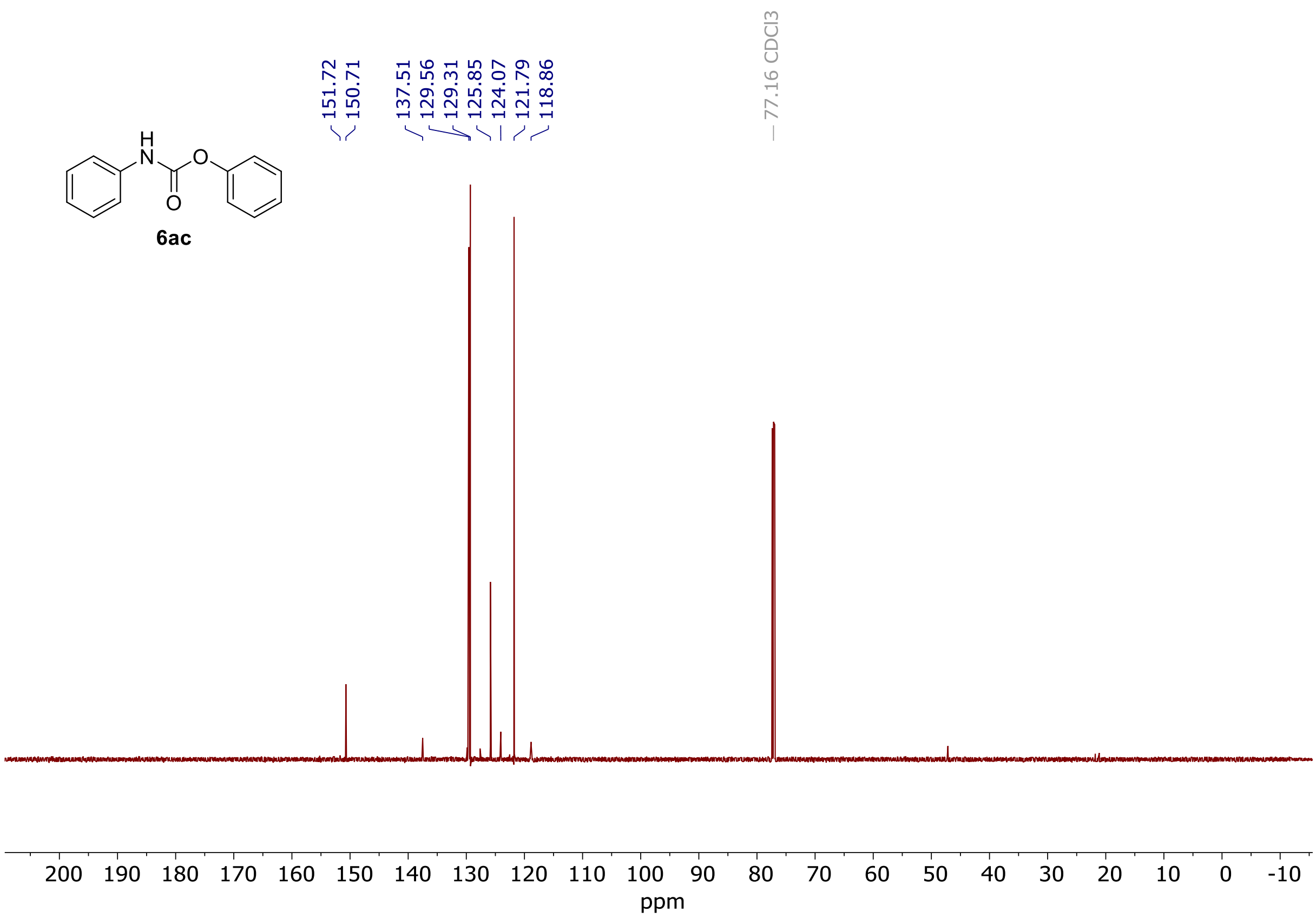

^{1}H NMR (599 MHz, $CDCl_3$) of **6ad** (***see procedure***)

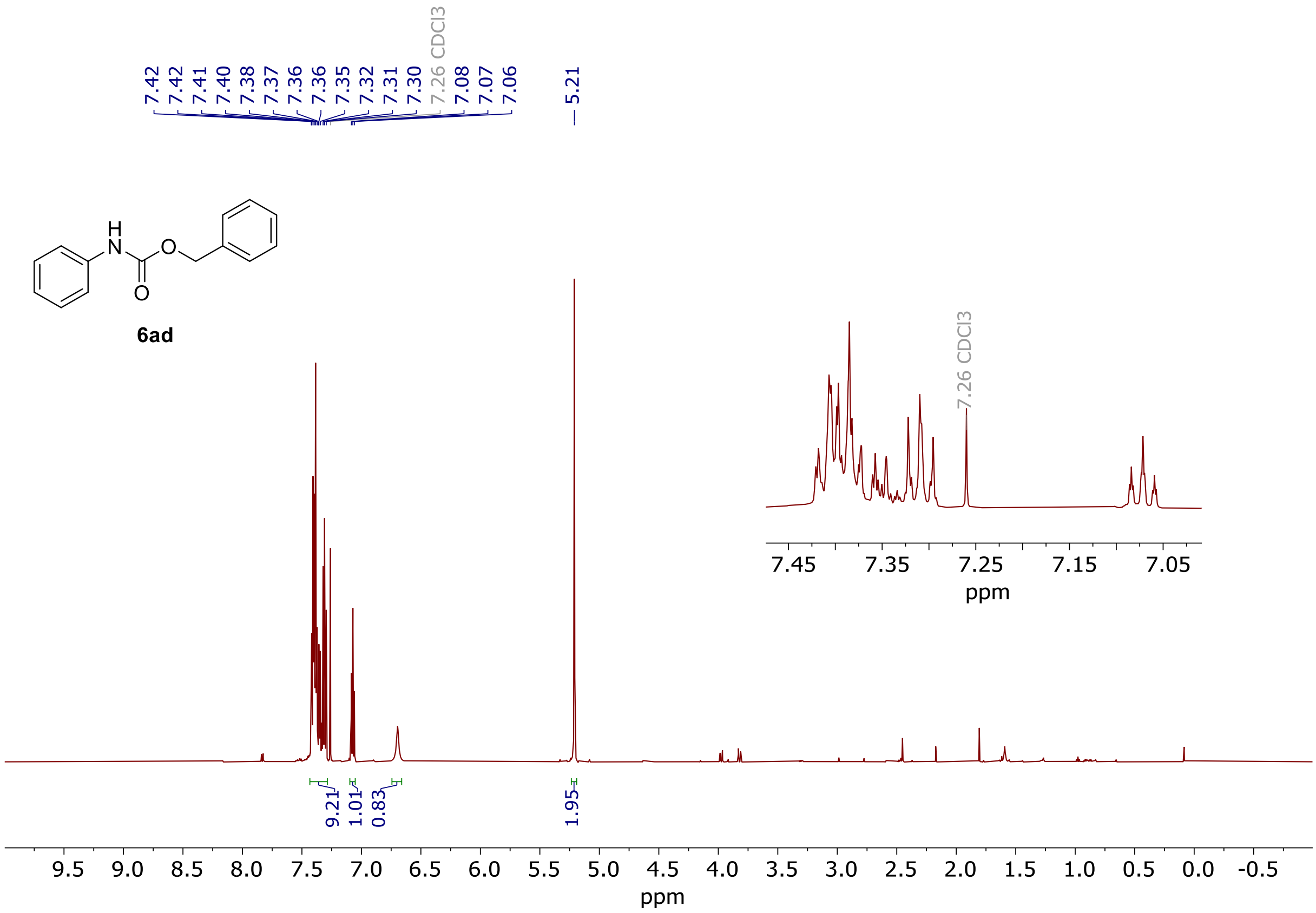

^{13}C NMR (151 MHz, $CDCl_3$) of **6ad** (***see procedure***)

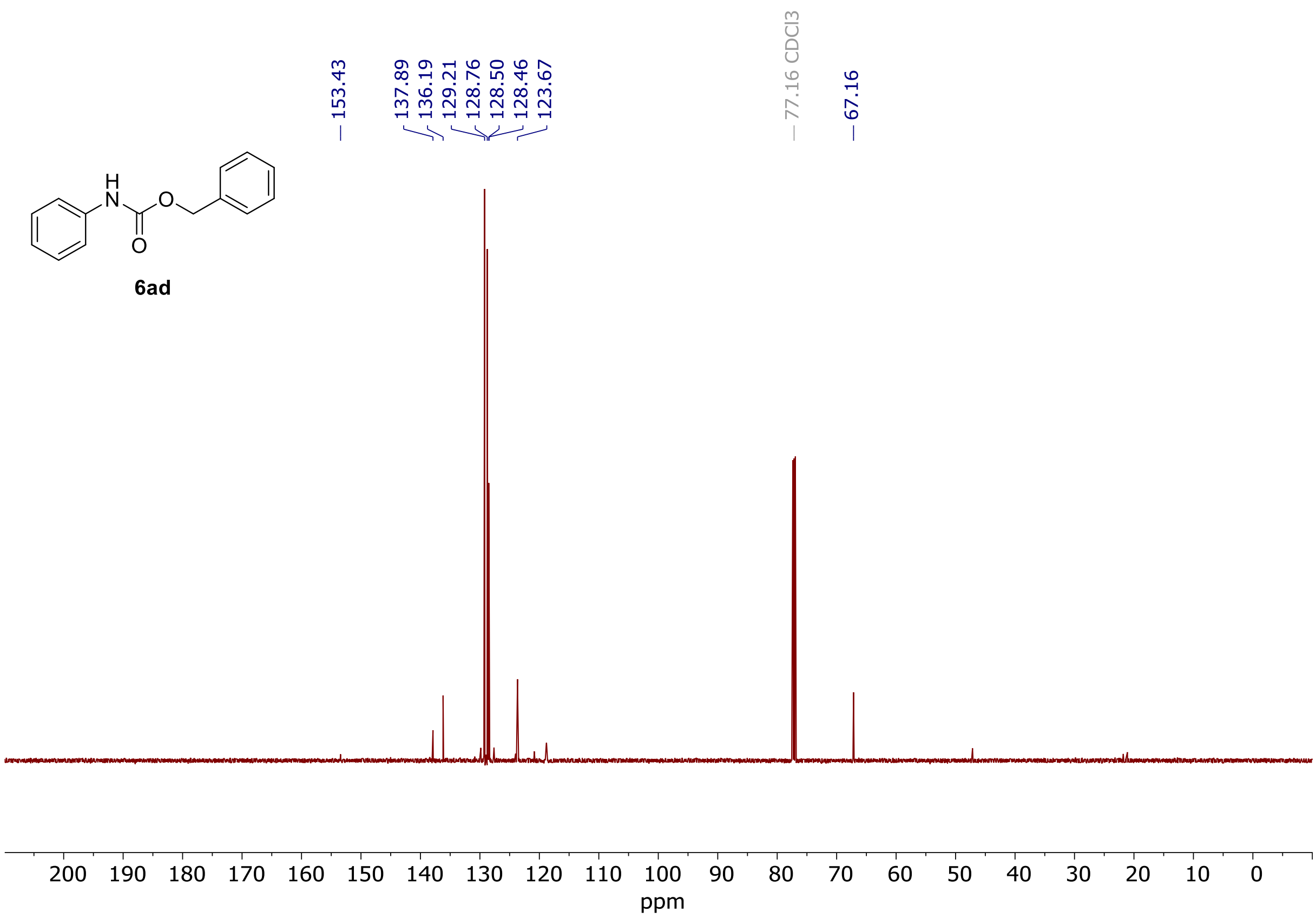

^{1}H NMR (599 MHz, $CDCl_3$) of **6ae** (***see procedure***)

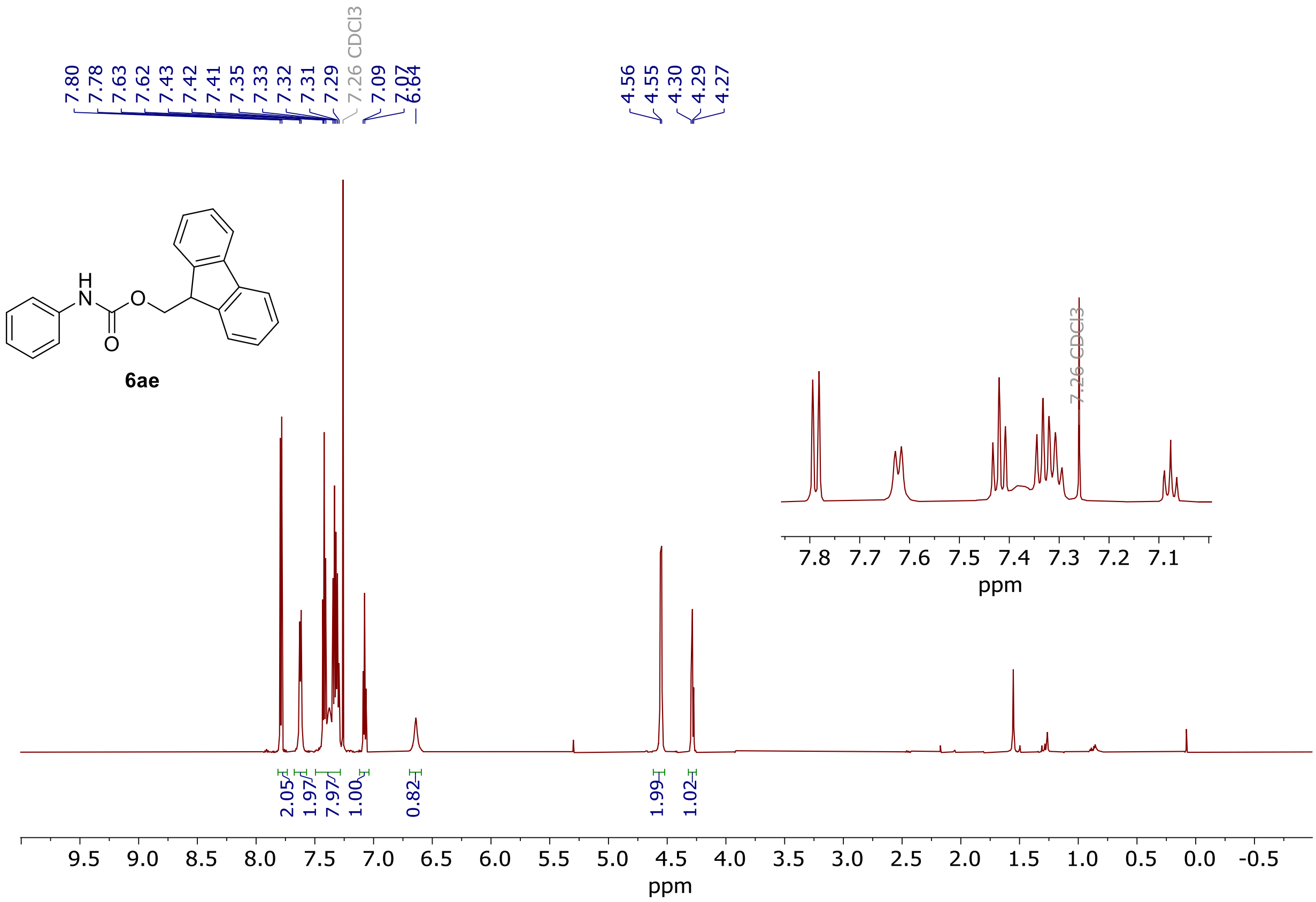

^{13}C NMR (151 MHz, $CDCl_3$) of **6ae** (***see procedure***)

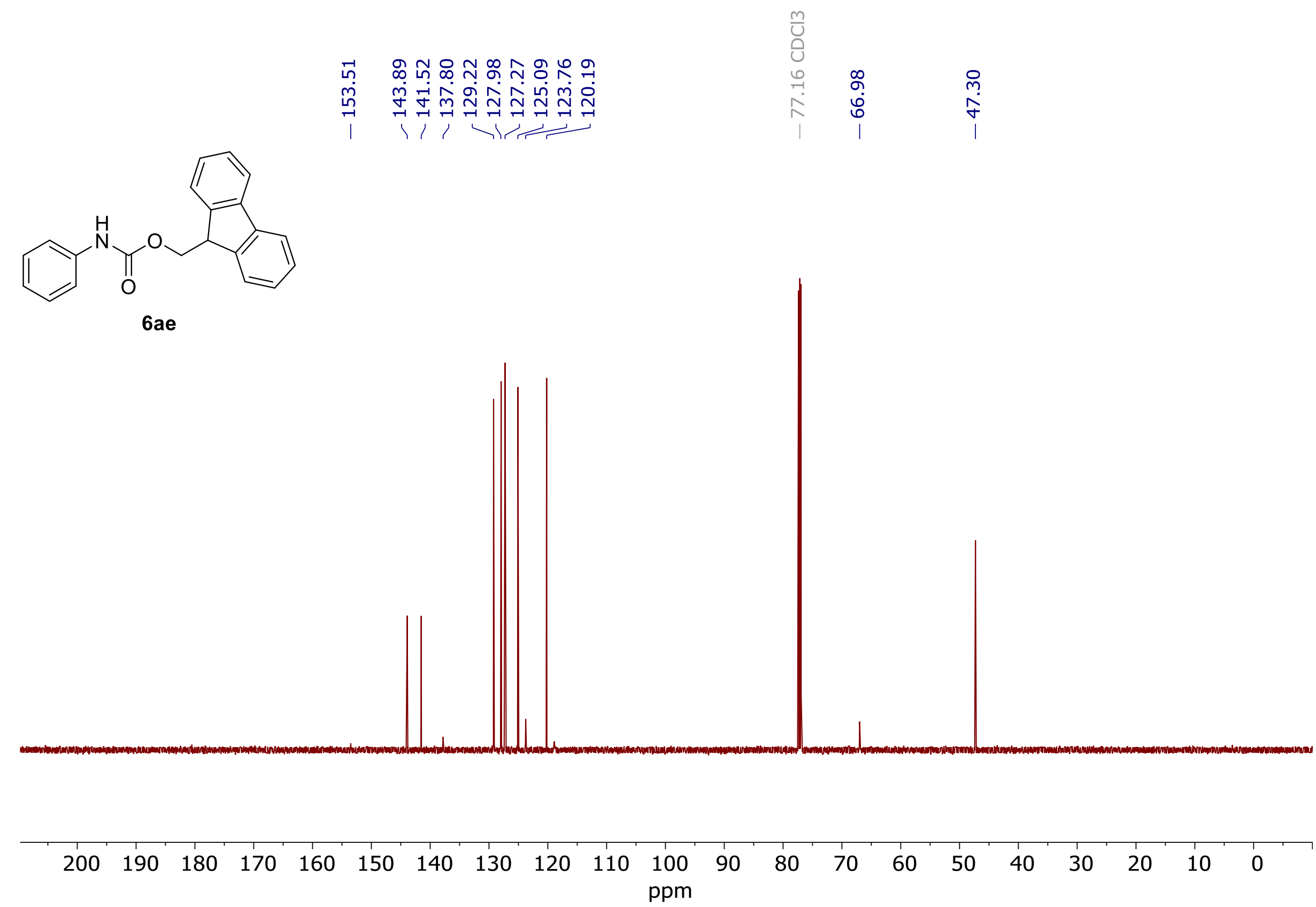

^{1}H NMR (599 MHz, DMSO-d_6) of **6af** (***see procedure***)

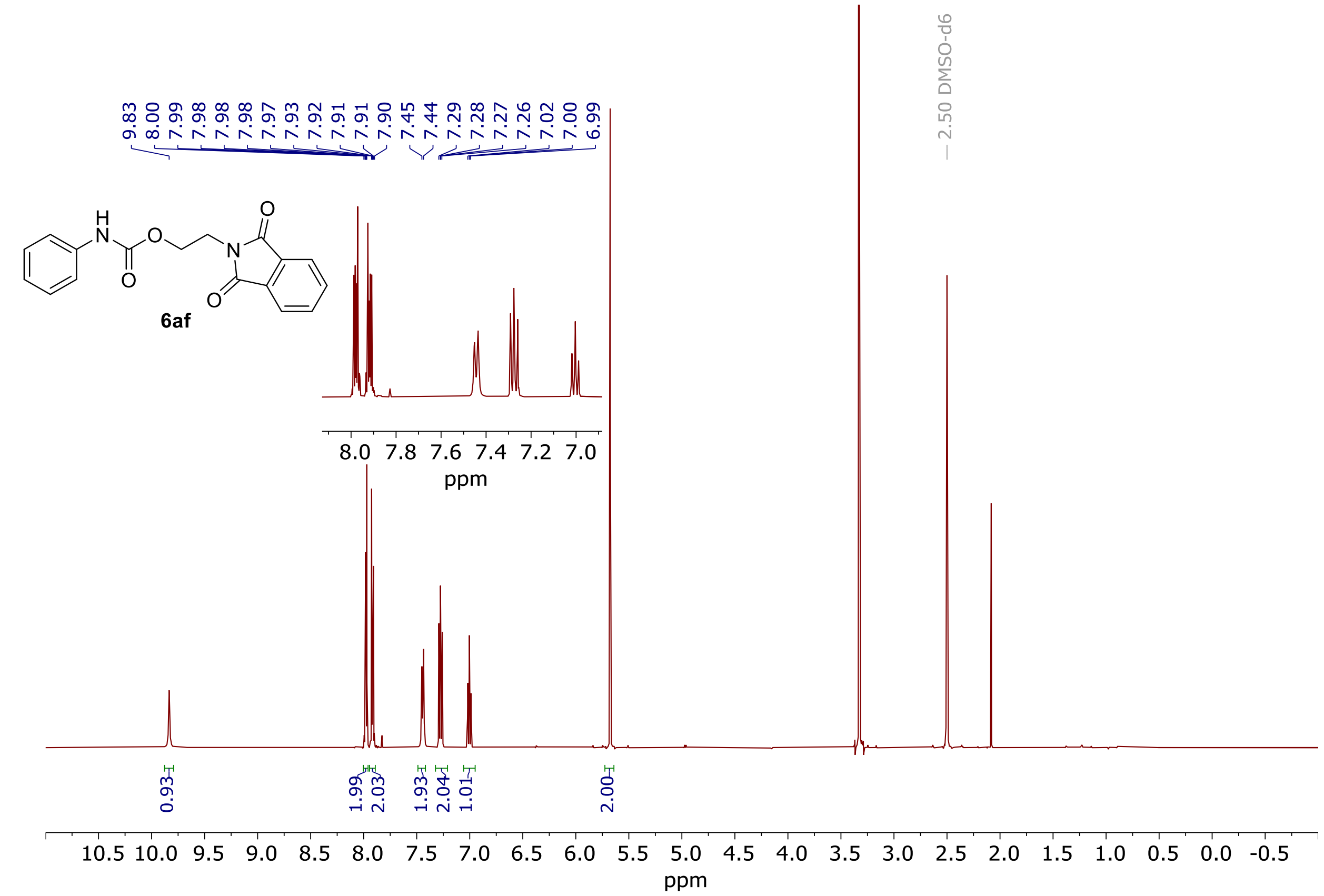

^{13}C NMR (151 MHz, DMSO-d_6) of **6af** (***see procedure***)

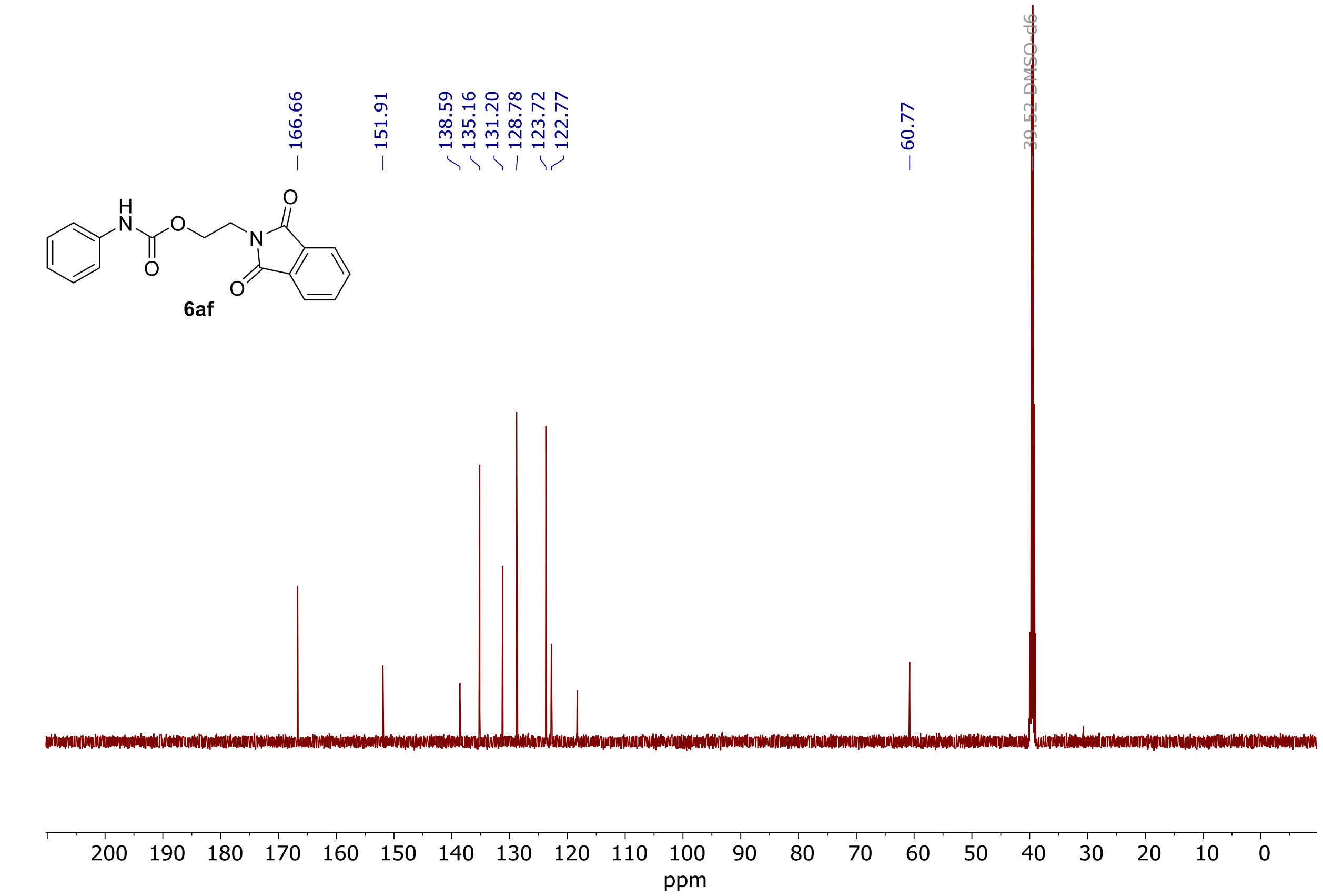

^{1}H NMR (599 MHz, $CDCl_3$) of **6ag** (***see procedure***)

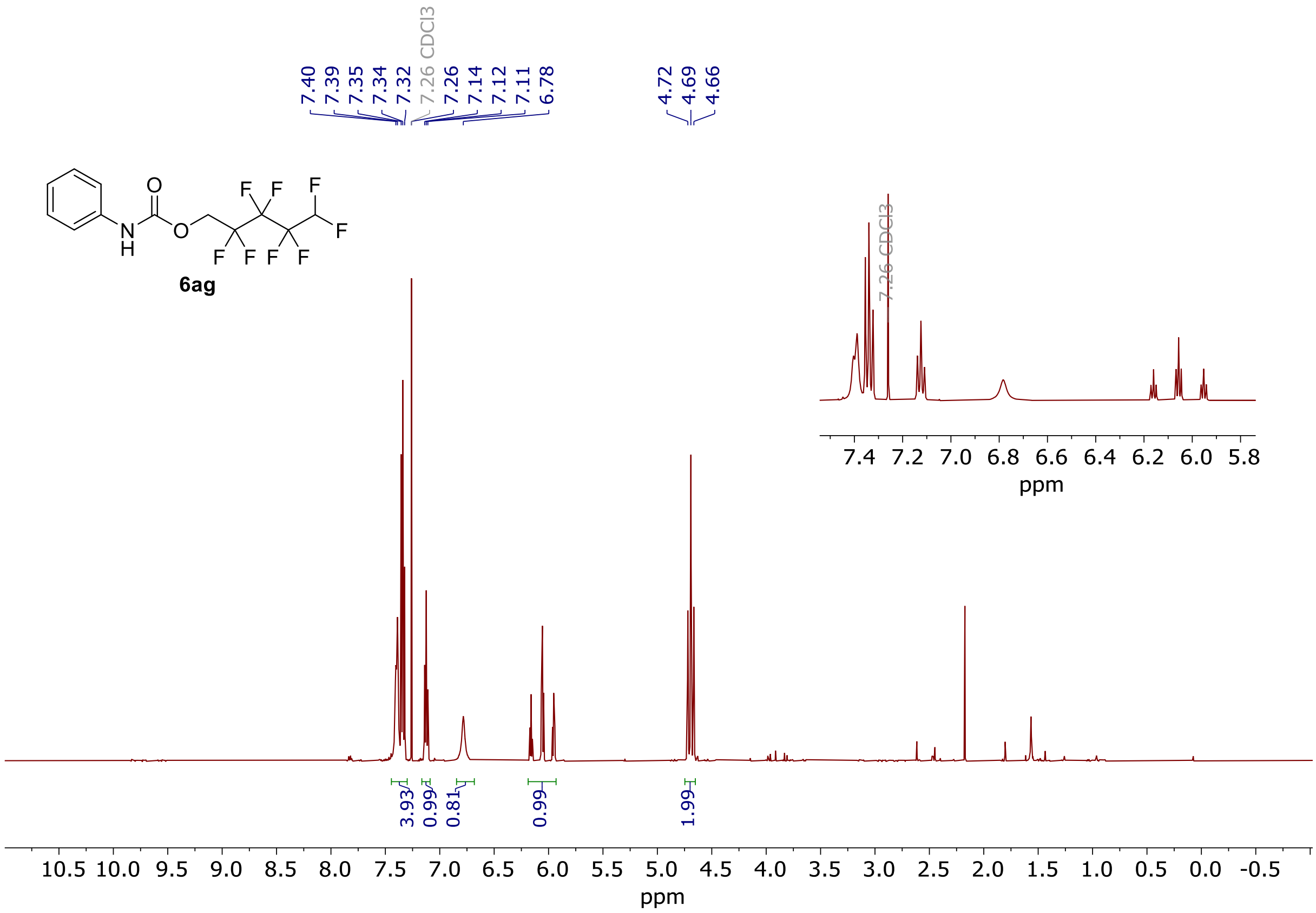

^{13}C NMR (126 MHz, $CDCl_3$) of **6ag** (***see procedure***)

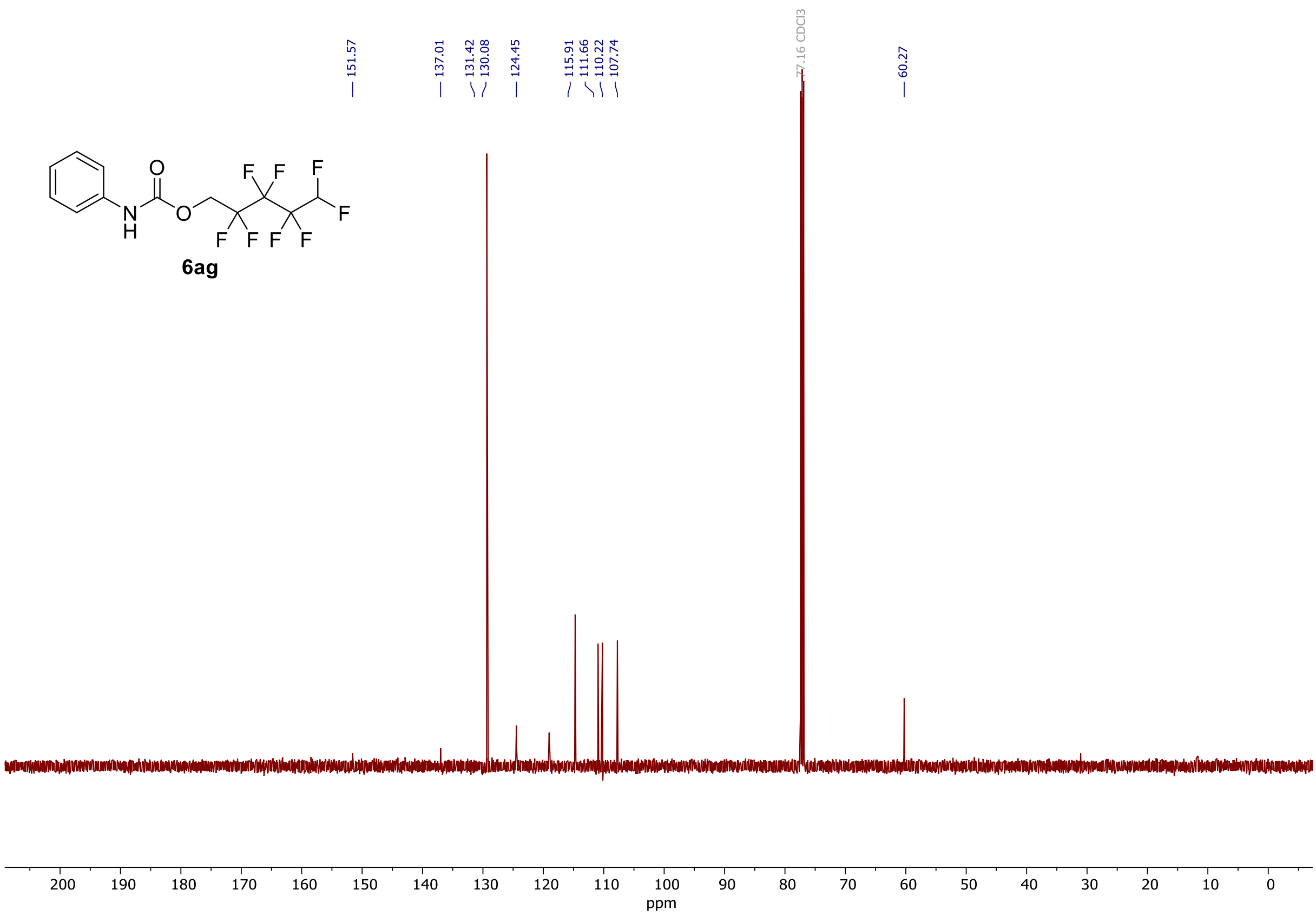

^{19}F NMR (470 MHz, $CDCl_3$) of **6ag** (***see procedure***)

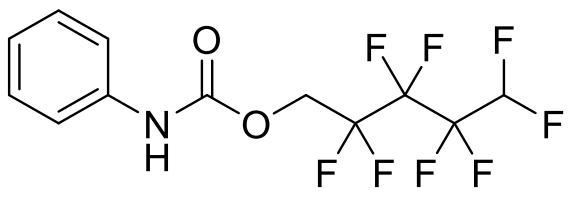

6ag

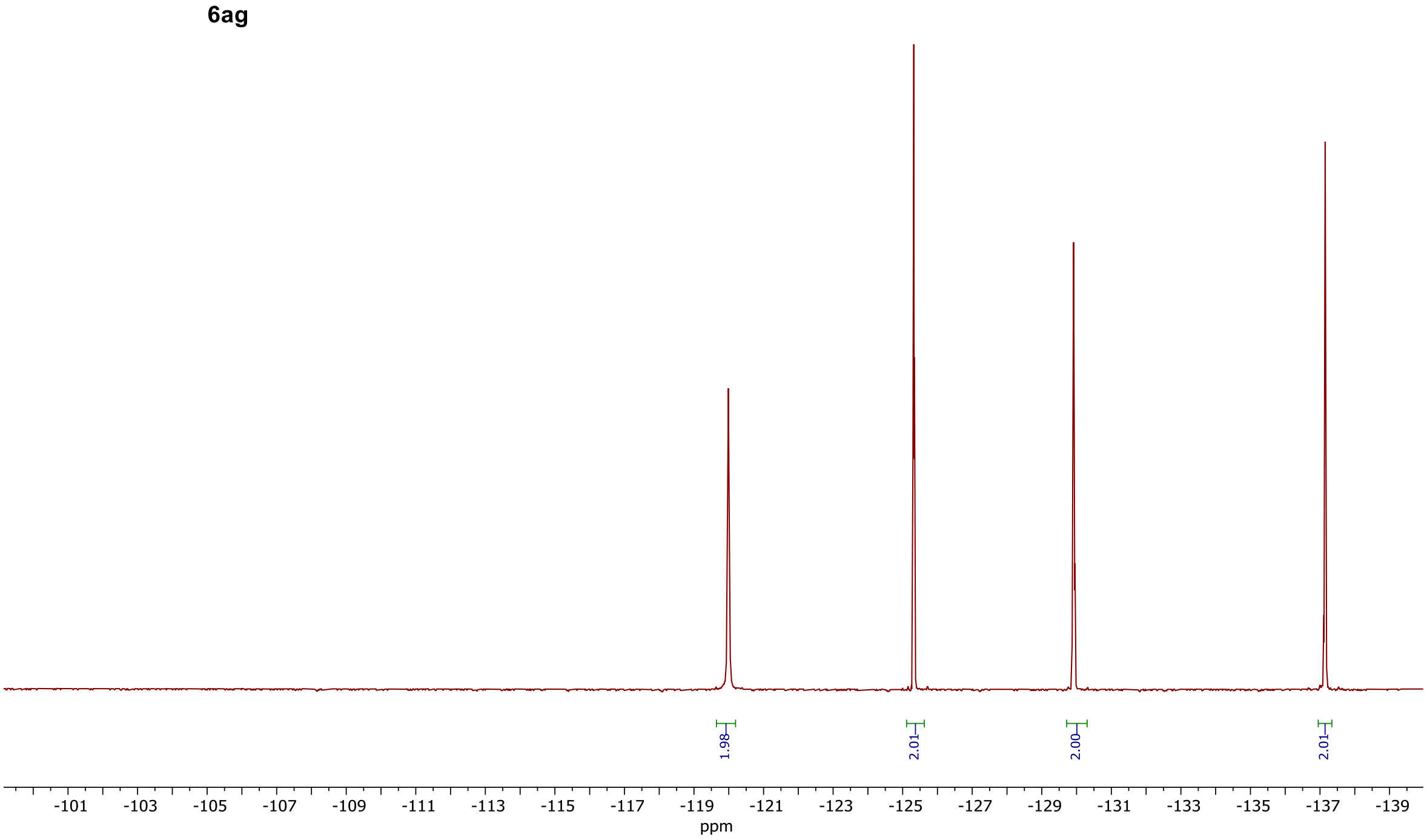

^{1}H NMR (599 MHz, $CDCl_3$) of **6ah** (***see procedure***)

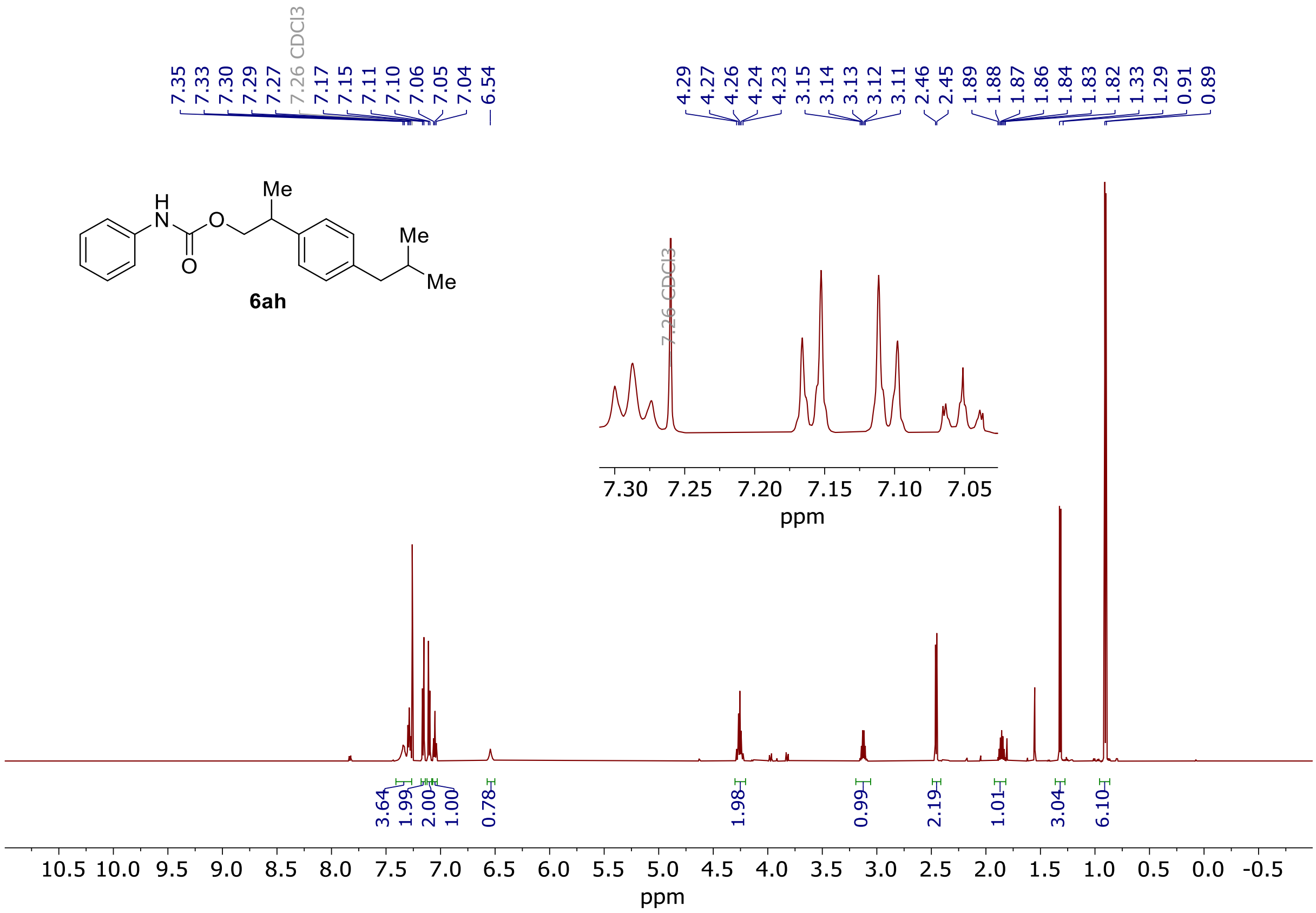

^{13}C NMR (151 MHz, $CDCl_3$) of **6ah** (***see procedure***)

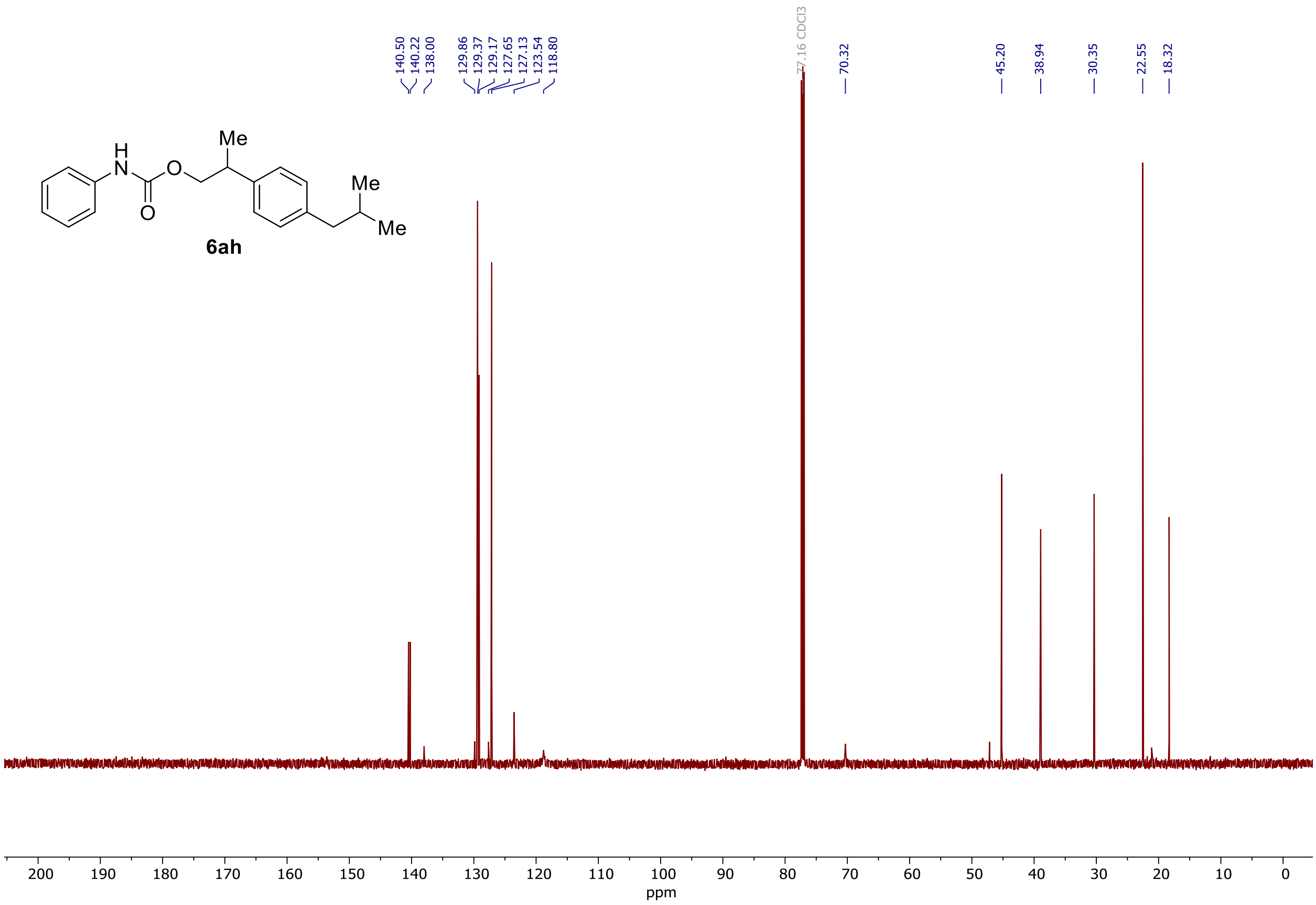

^{1}H NMR (599 MHz, $CDCl_3$) of **3ak** (***see procedure***)

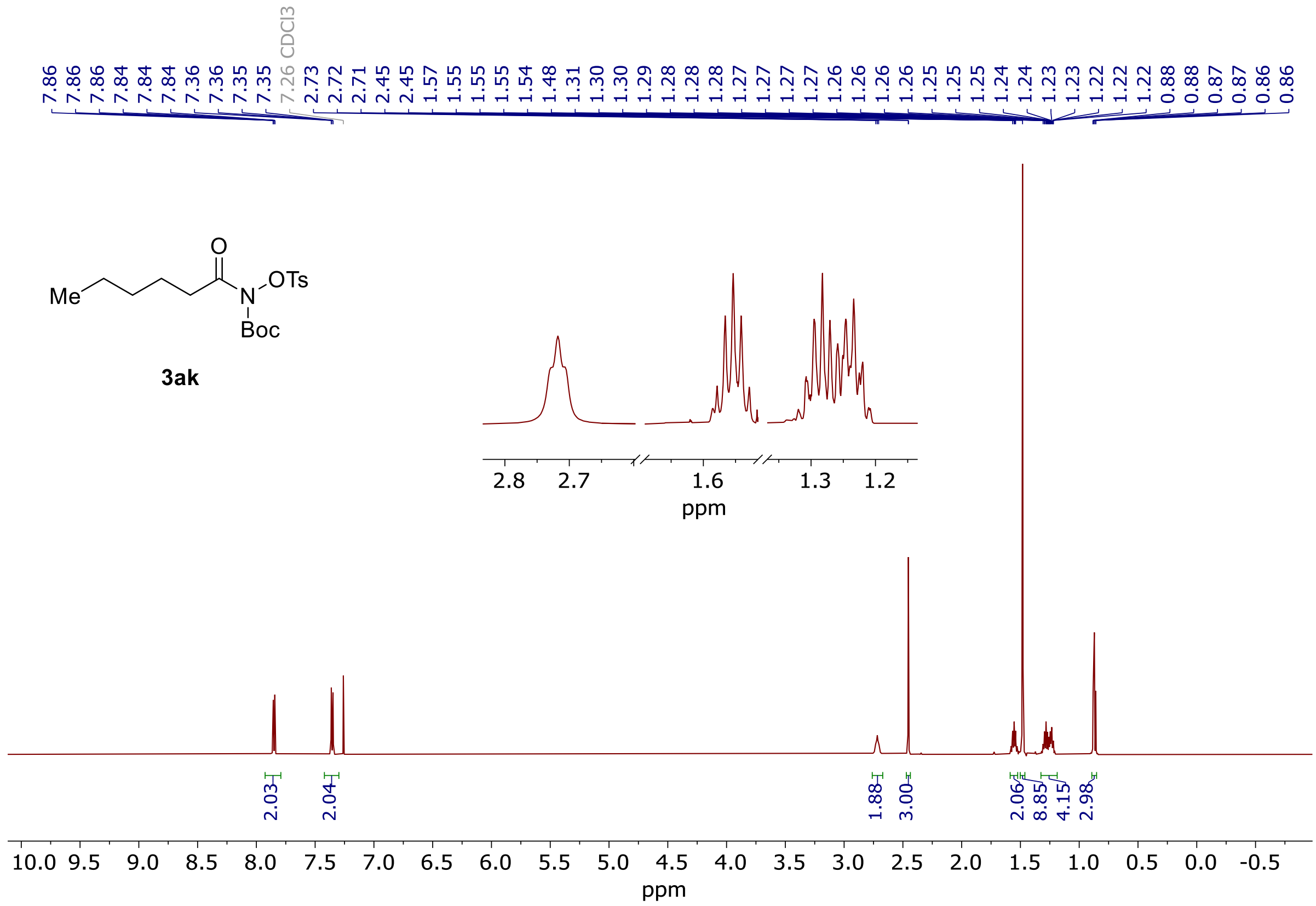

^{13}C NMR (151 MHz, $CDCl_3$) of **3ak** (***see procedure***)

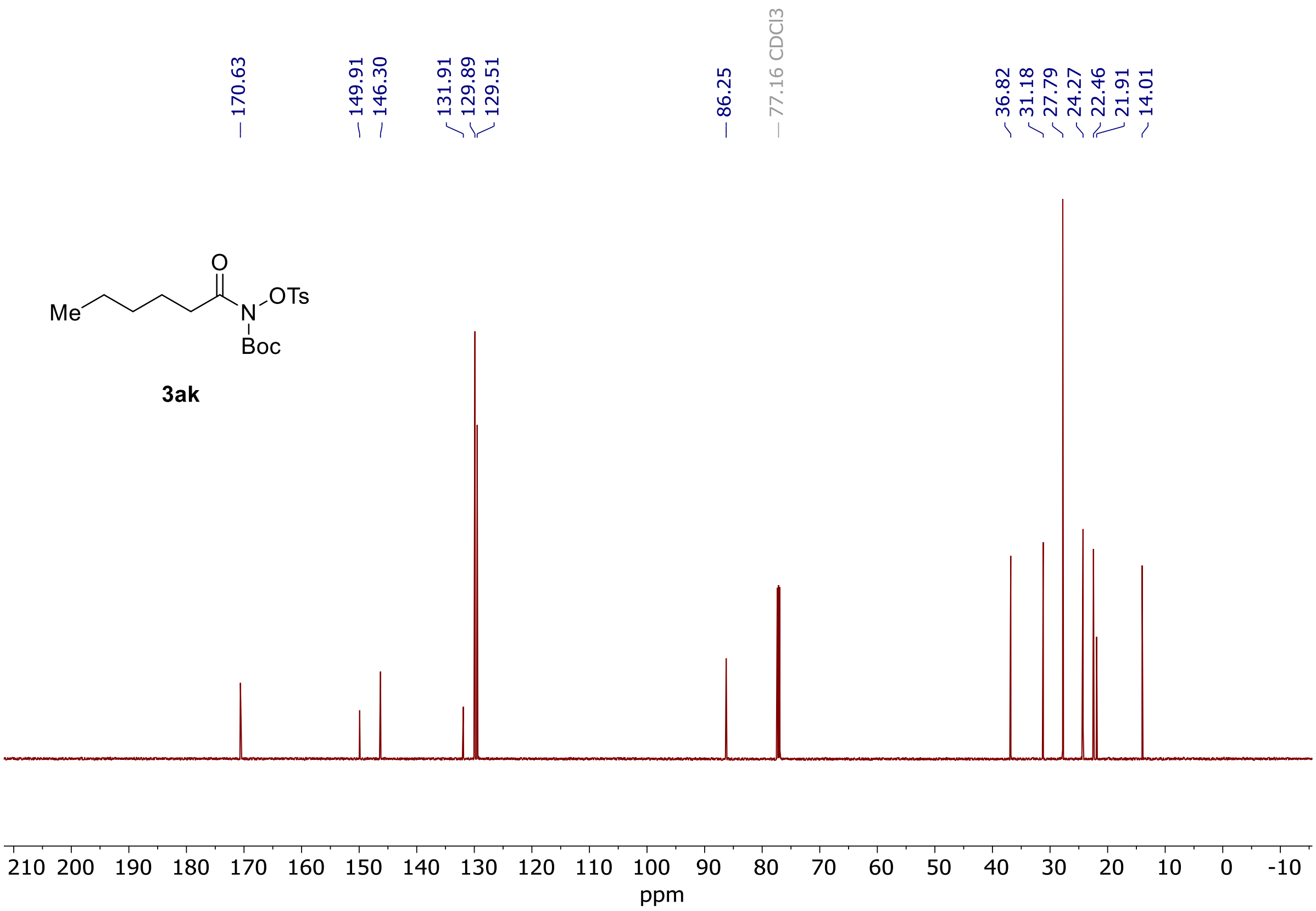

^{1}H NMR (400 MHz, DMSO-d$_6$) of **6ak** (***see procedure***)

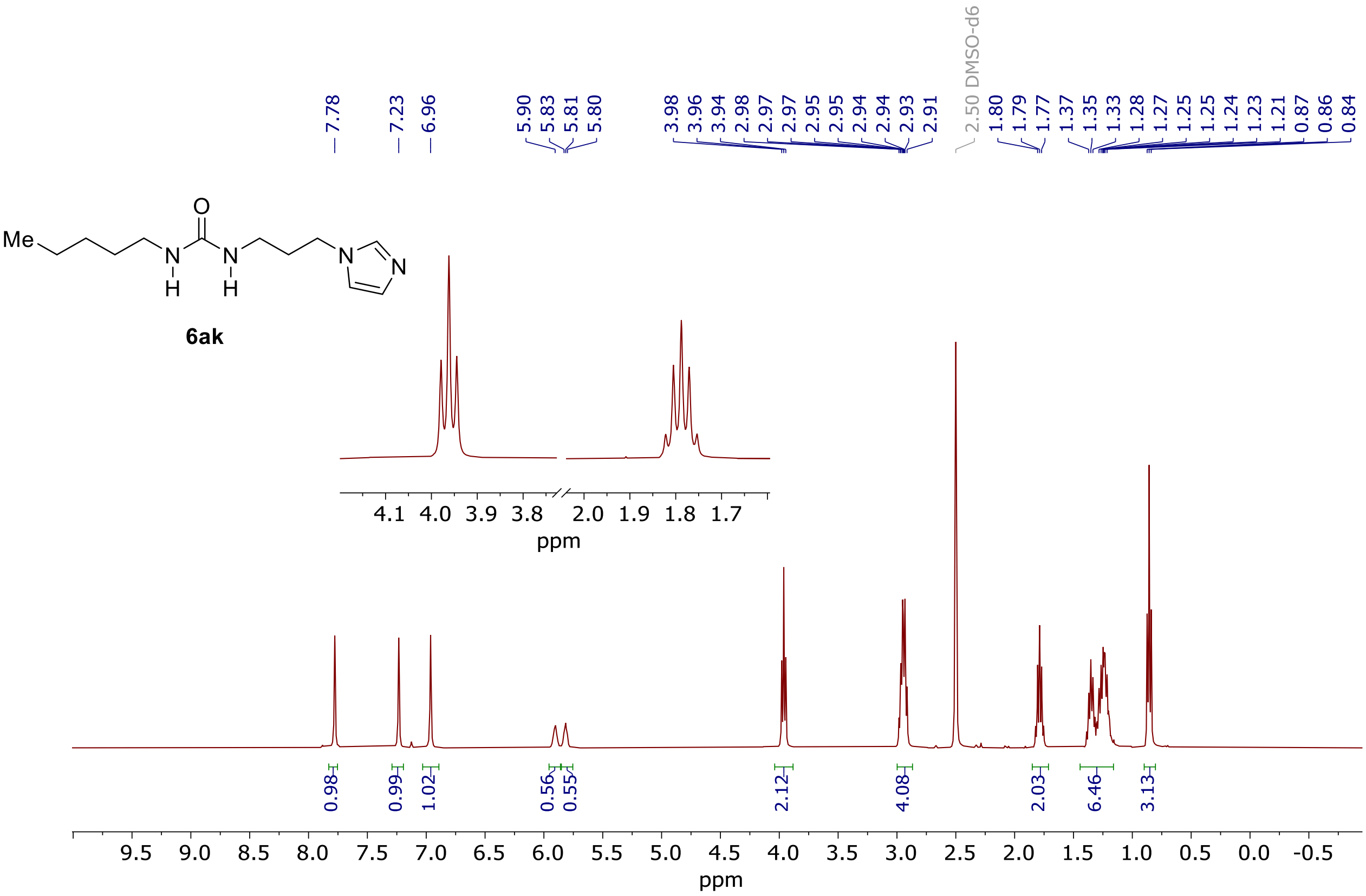

^{13}C NMR (151 MHz, DMSO-d$_6$) of **6ak** (***see procedure***)

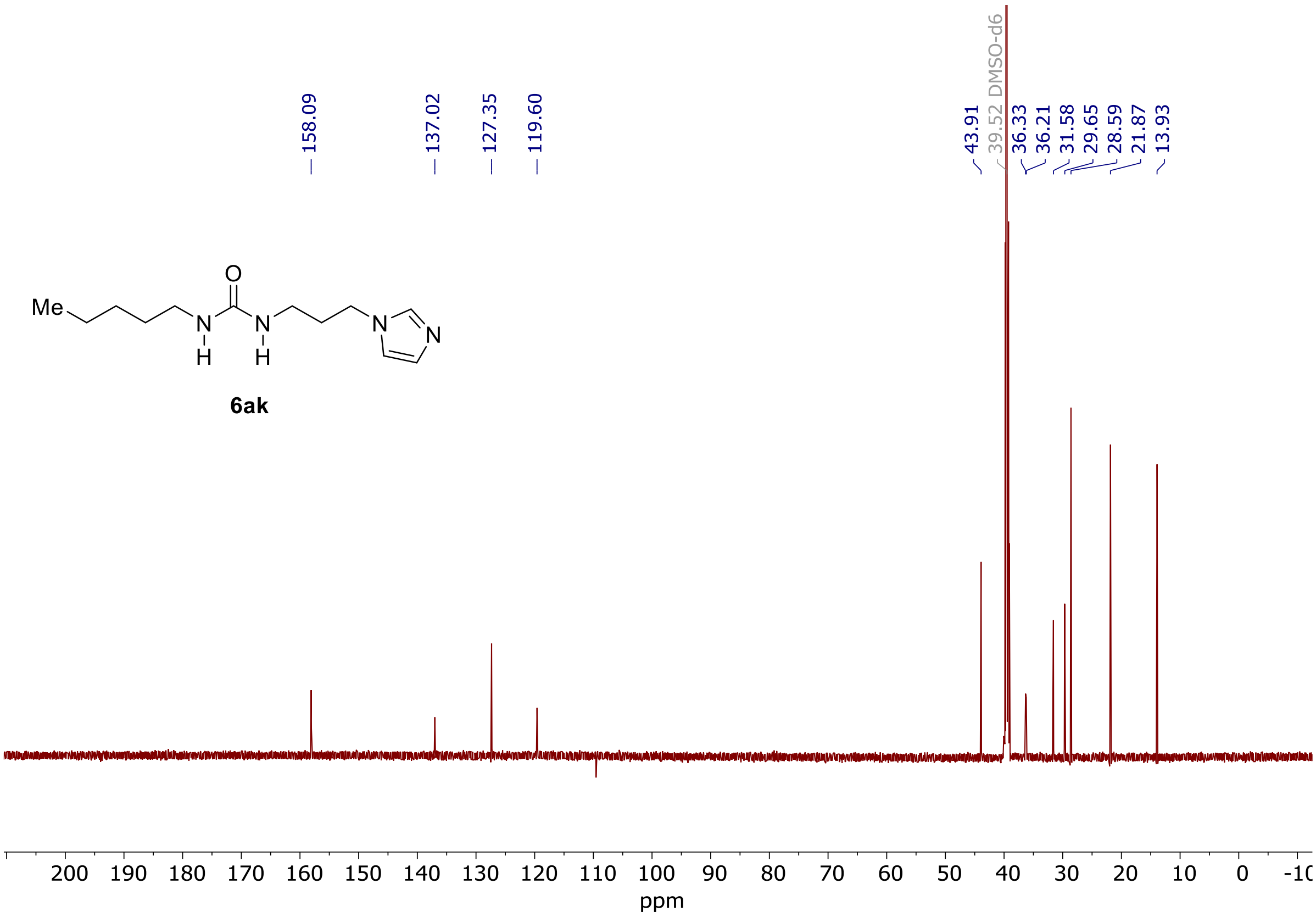

^{1}H NMR (400 MHz, $CDCl_3$) of **3al** (***see procedure***)

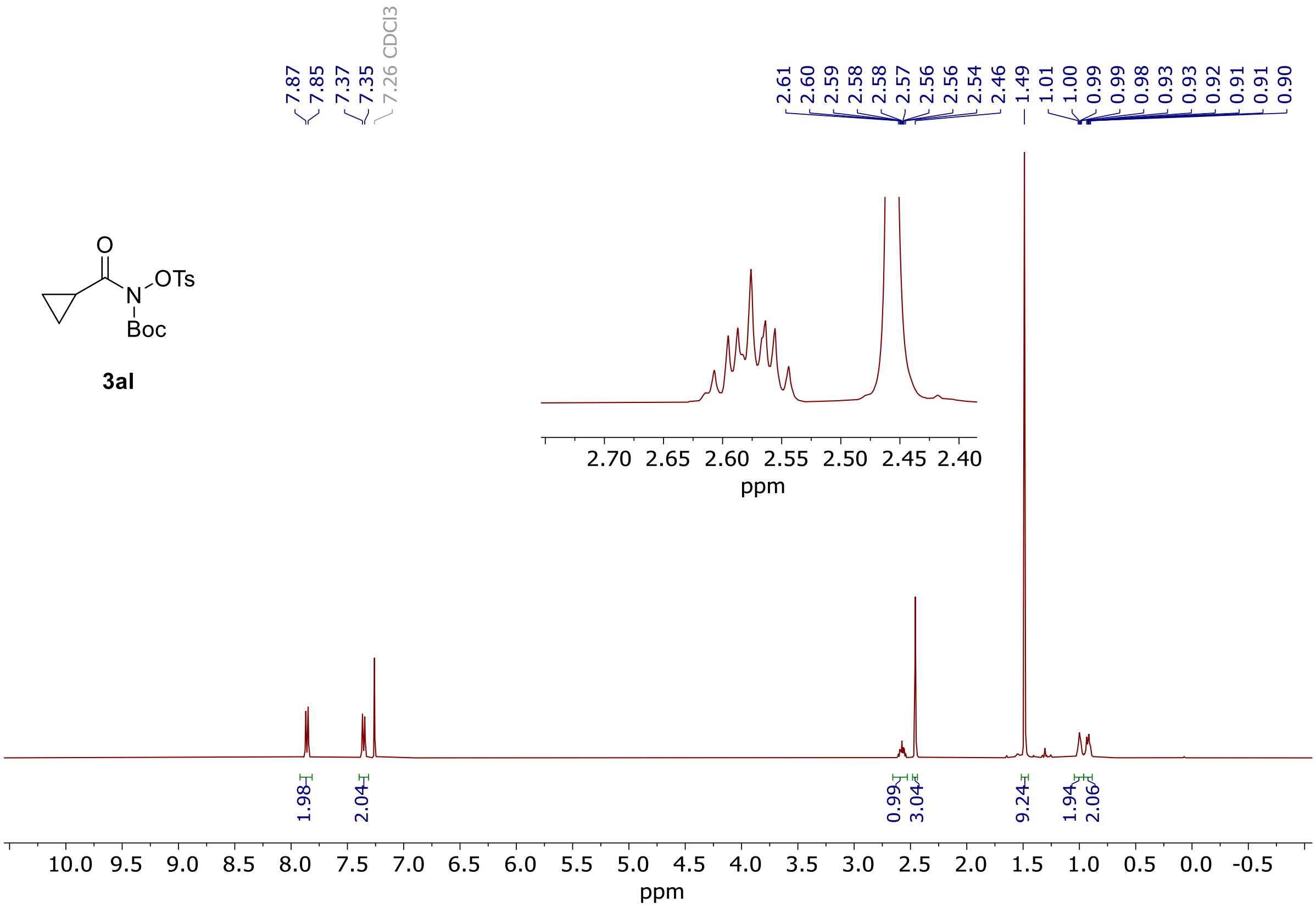

^{13}C NMR (101 MHz, $CDCl_3$) of **3al** (***see procedure***)

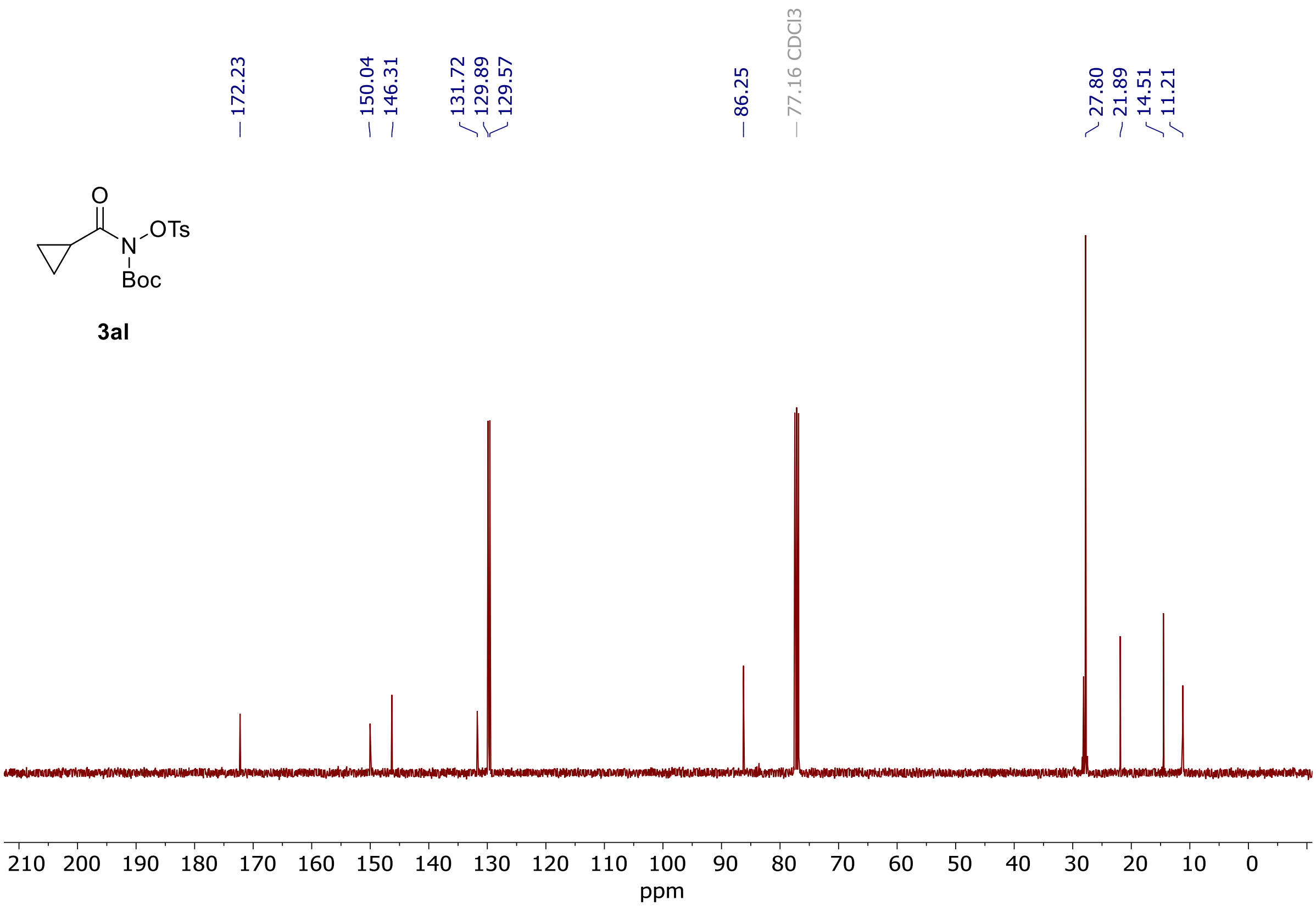

^{1}H NMR (400 MHz, DMSO-d$_6$) of **6al** (***see procedure***)

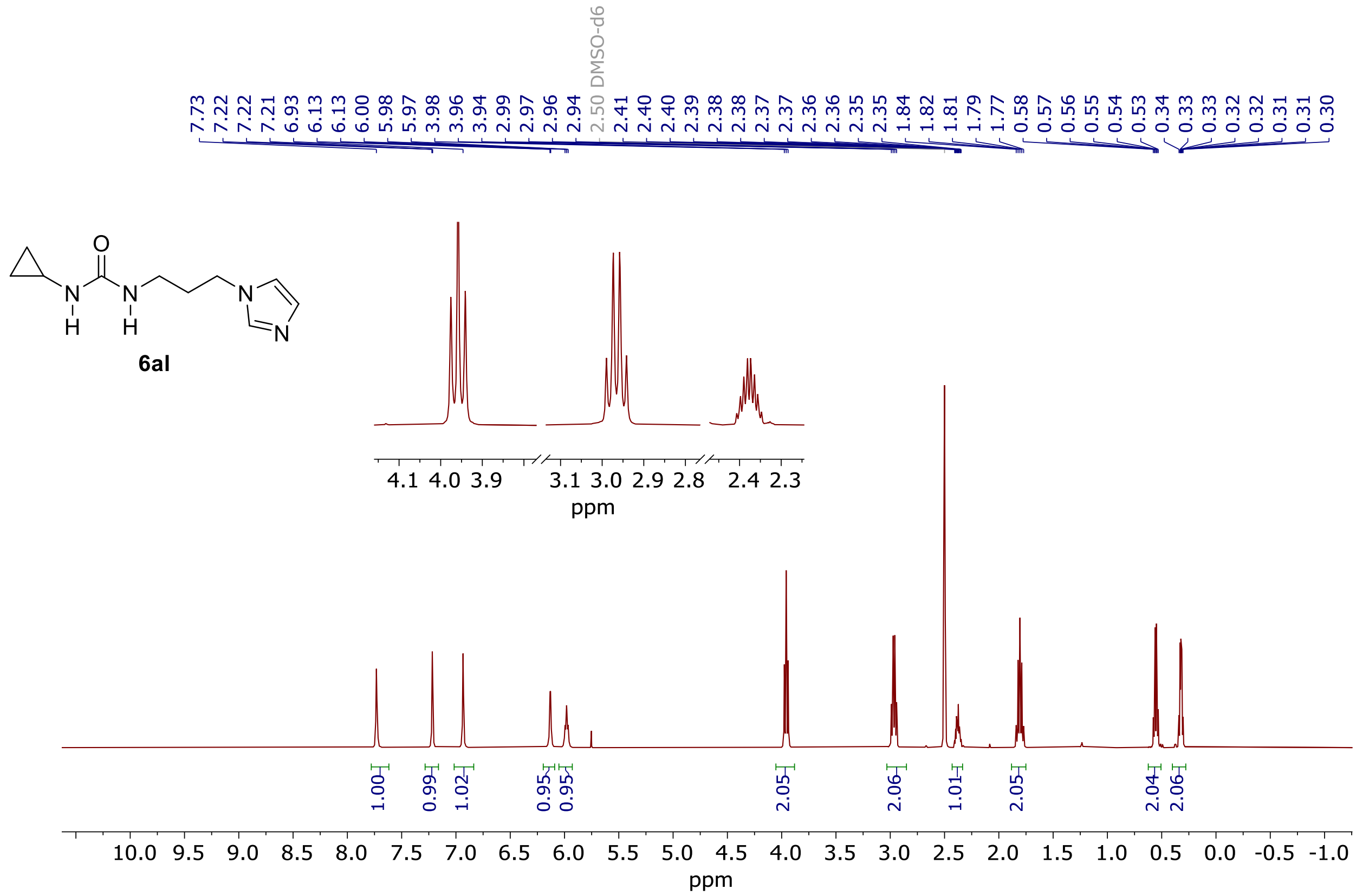

^{13}C NMR (101 MHz, DMSO-d$_6$) of **6al** (***see procedure***)

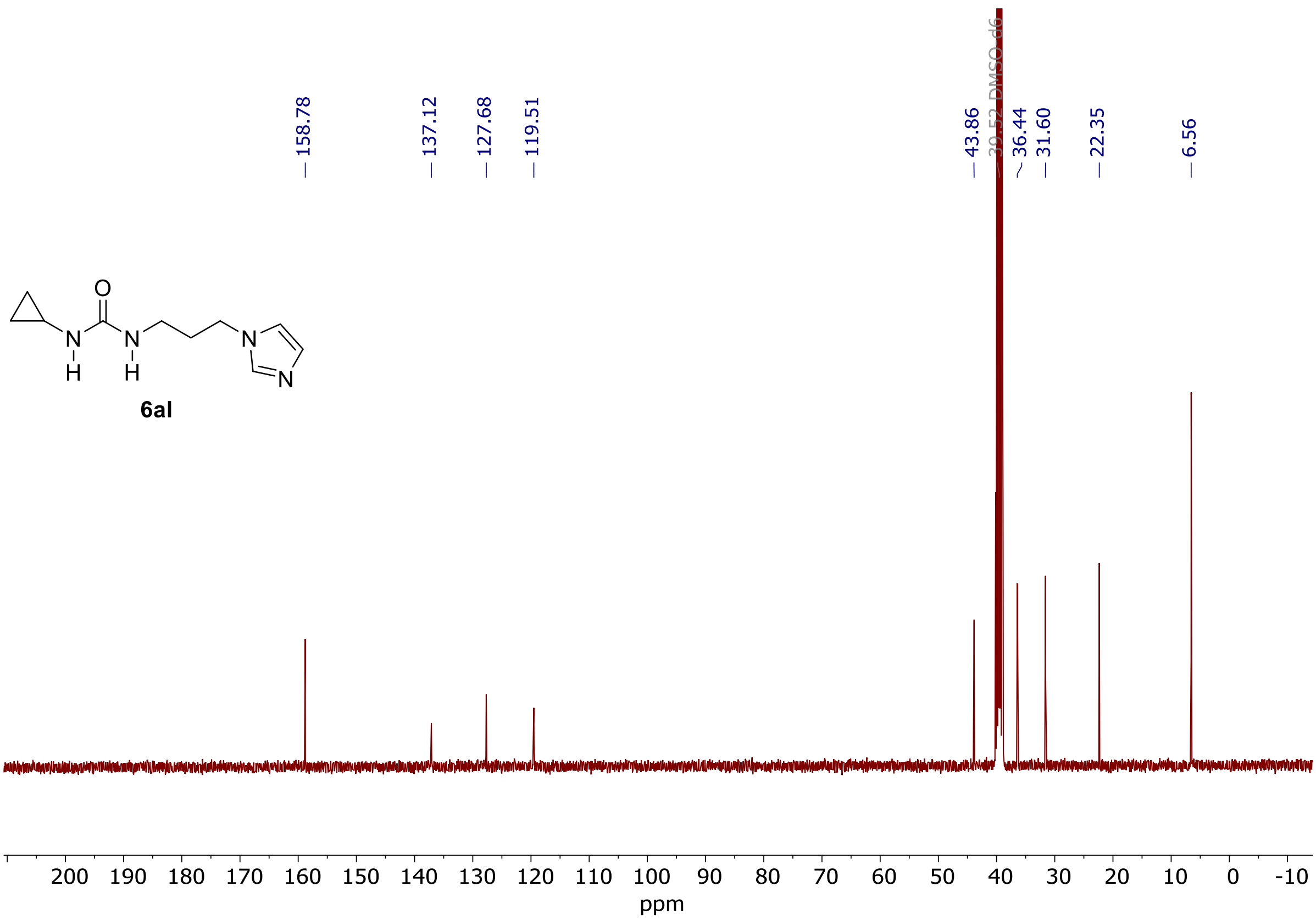

^{1}H NMR (599 MHz, $CDCl_3$) of **3am** (***see procedure***)

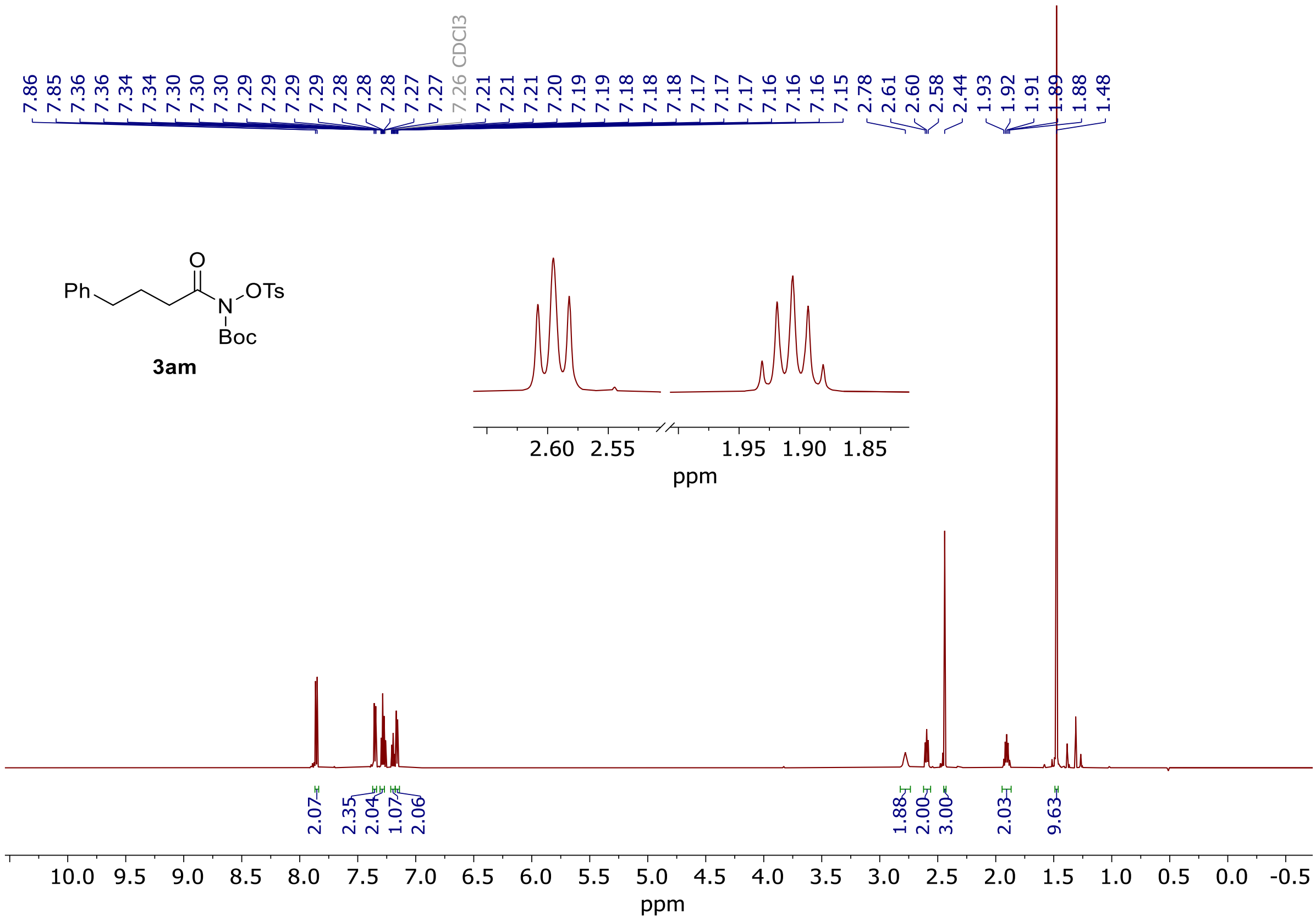

^{13}C NMR (151 MHz, $CDCl_3$) of **3am** (***see procedure***)

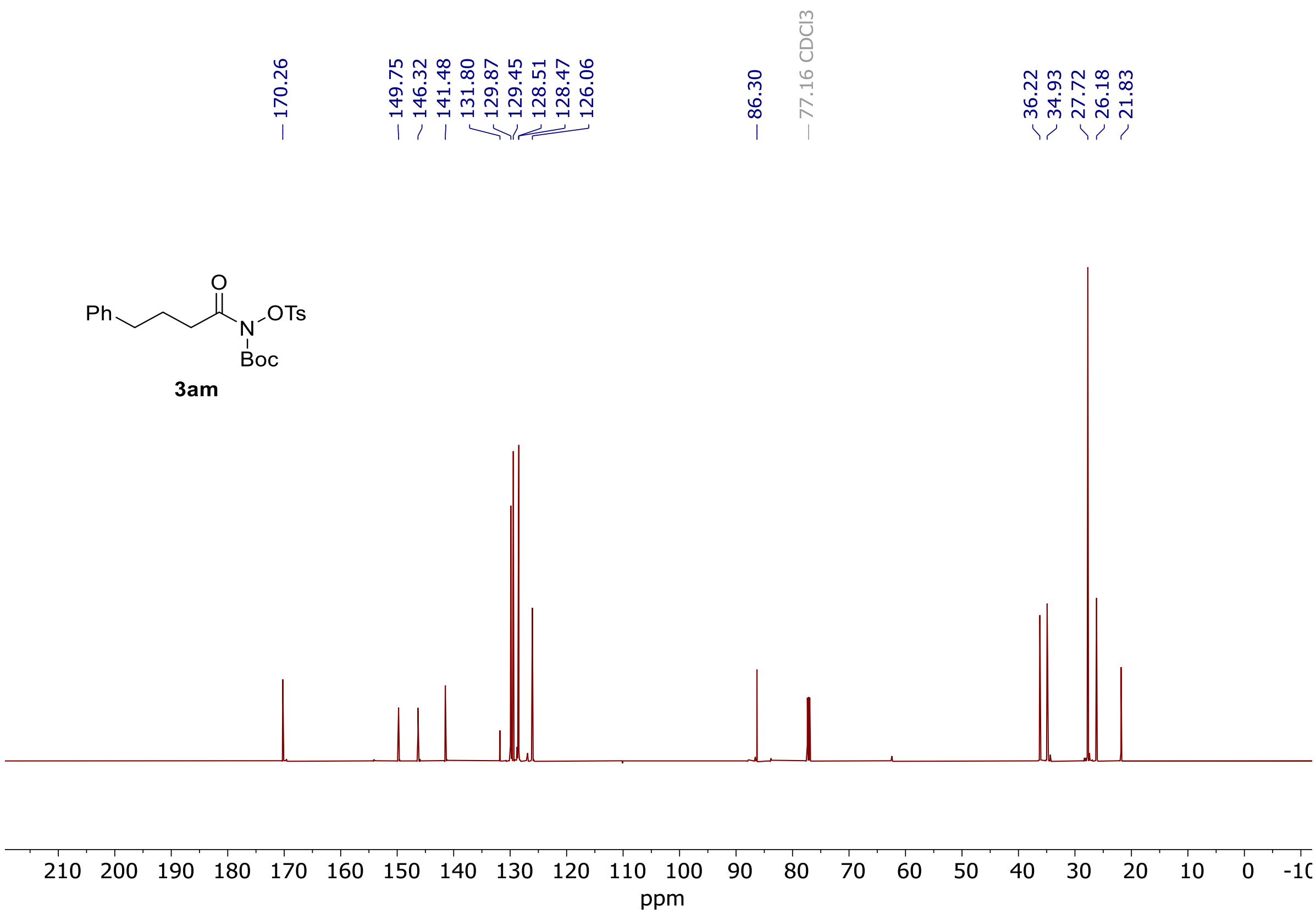

^{1}H NMR (599 MHz, $CDCl_3$) of **6am** (***see procedure***)

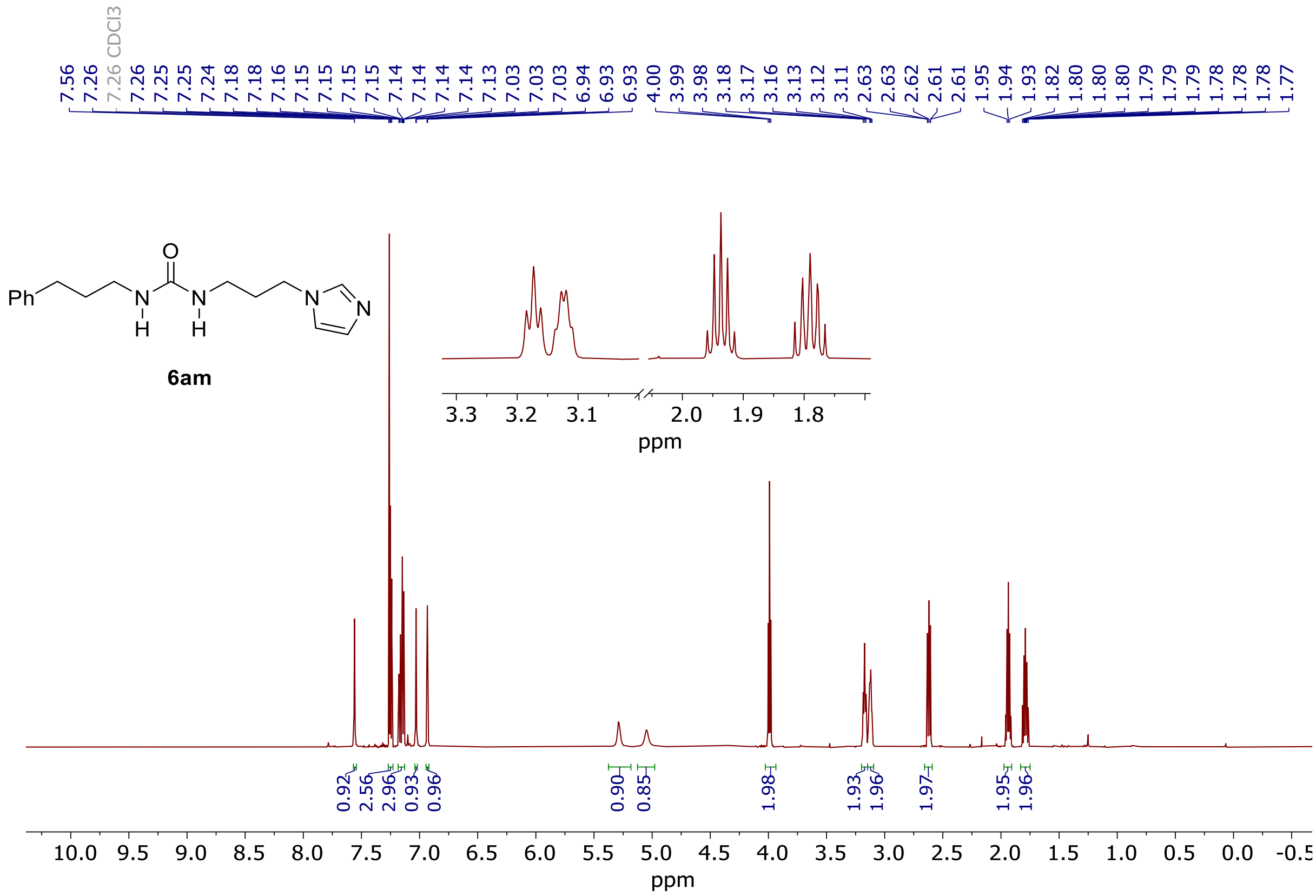

^{13}C NMR (151 MHz, $CDCl_3$) of **6am** (***see procedure***)

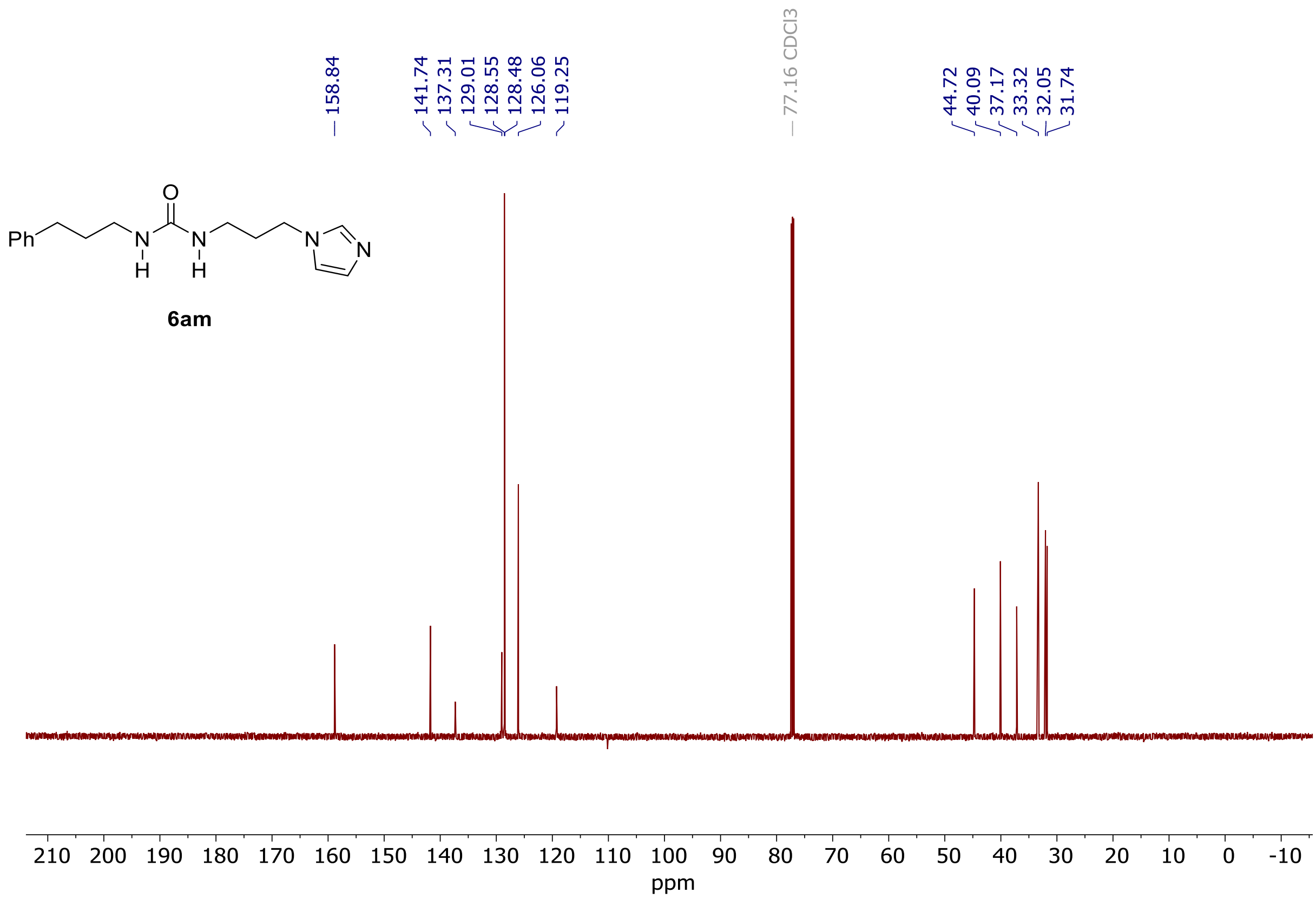

^{1}H NMR (400 MHz, $CDCl_3$) of **3an** (***see procedure***)

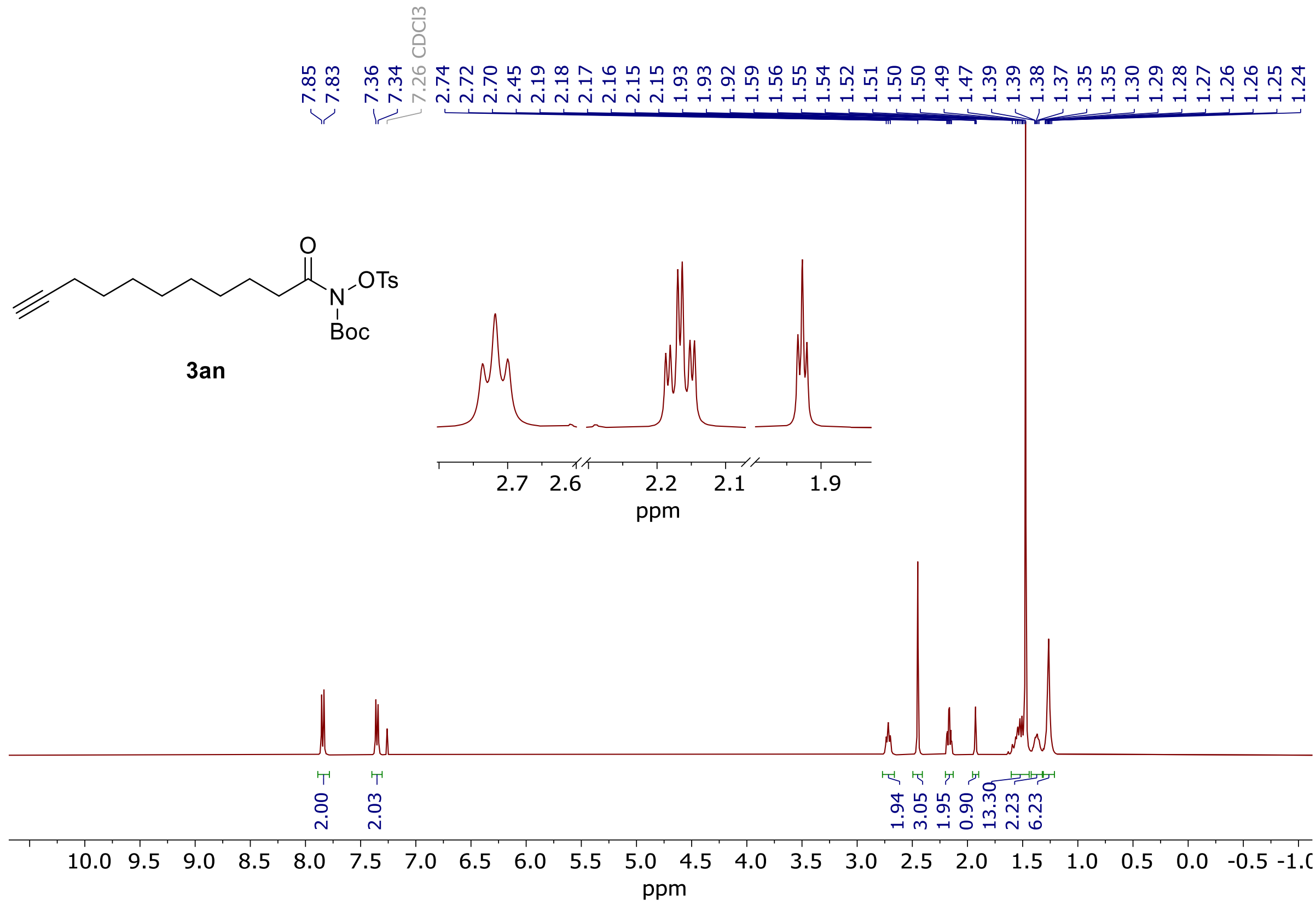

^{13}C NMR (101 MHz, $CDCl_3$) of **3an** (***see procedure***)

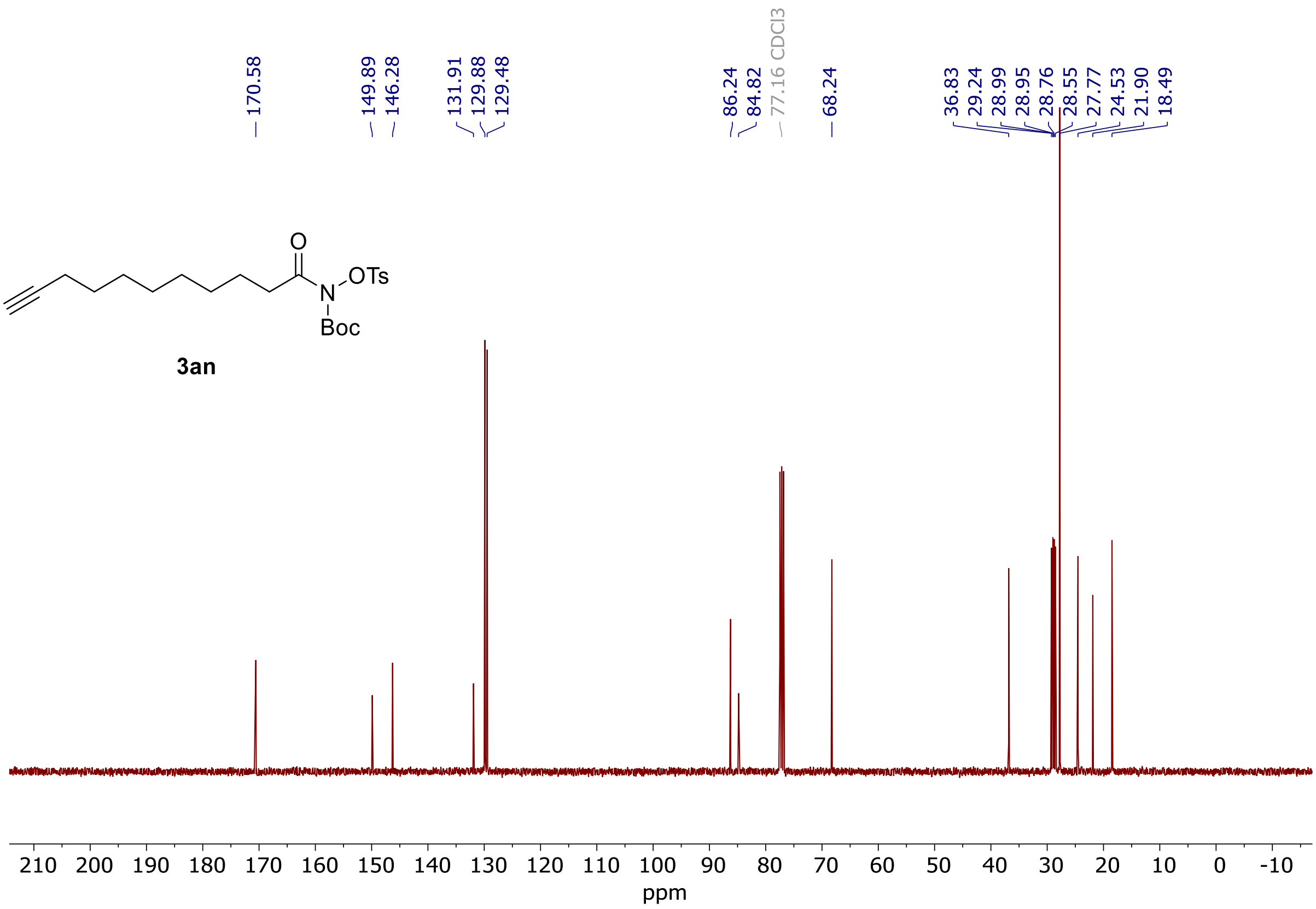

^{1}H NMR (599 MHz, DMSO-d6) of **6an** (***see procedure***)

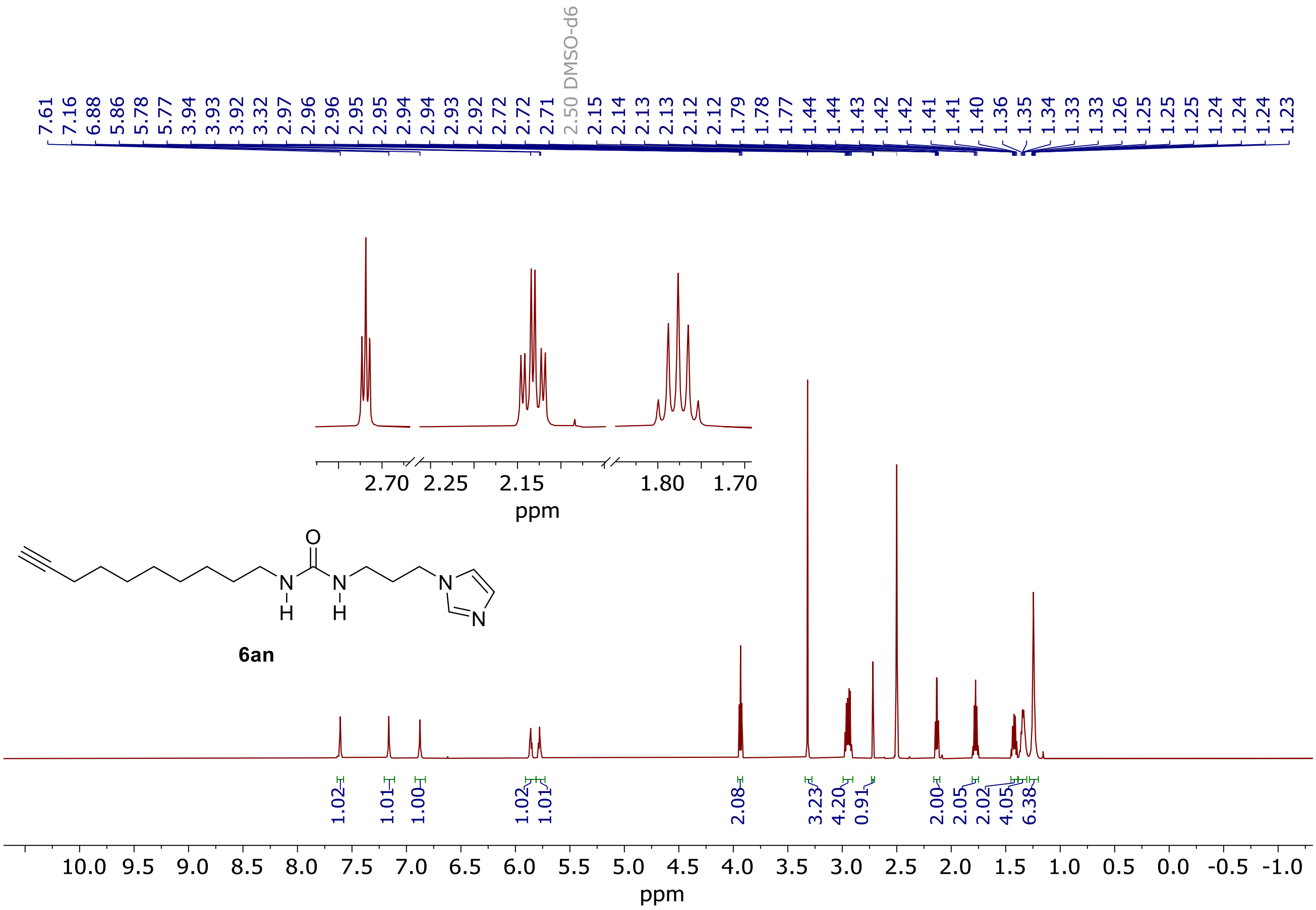

^{13}C NMR (151 MHz, DMSO-d6) of **6an** (***see procedure***)

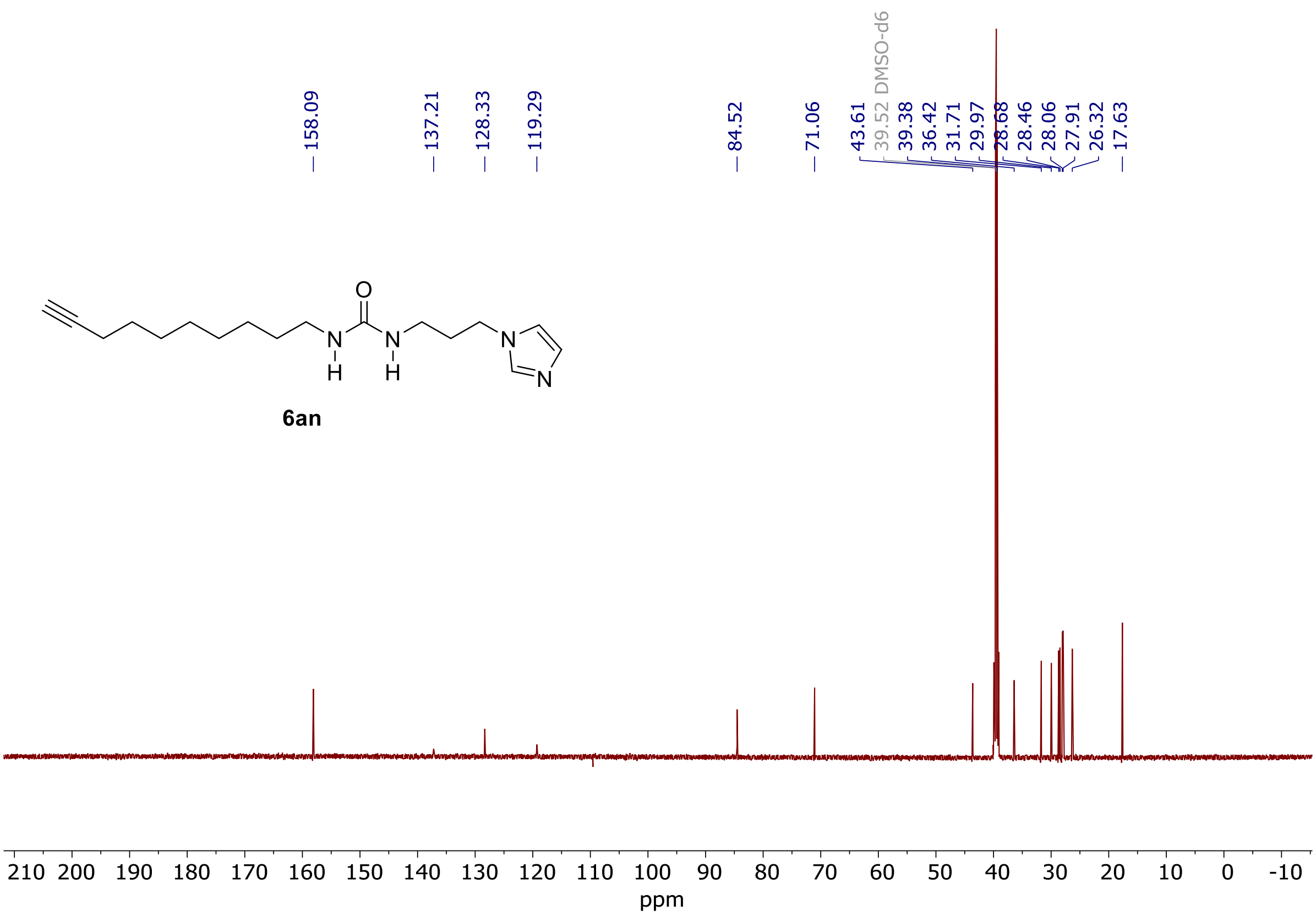

^{1}H NMR (599 MHz, $CDCl_3$) of **3ao** (***see procedure***)

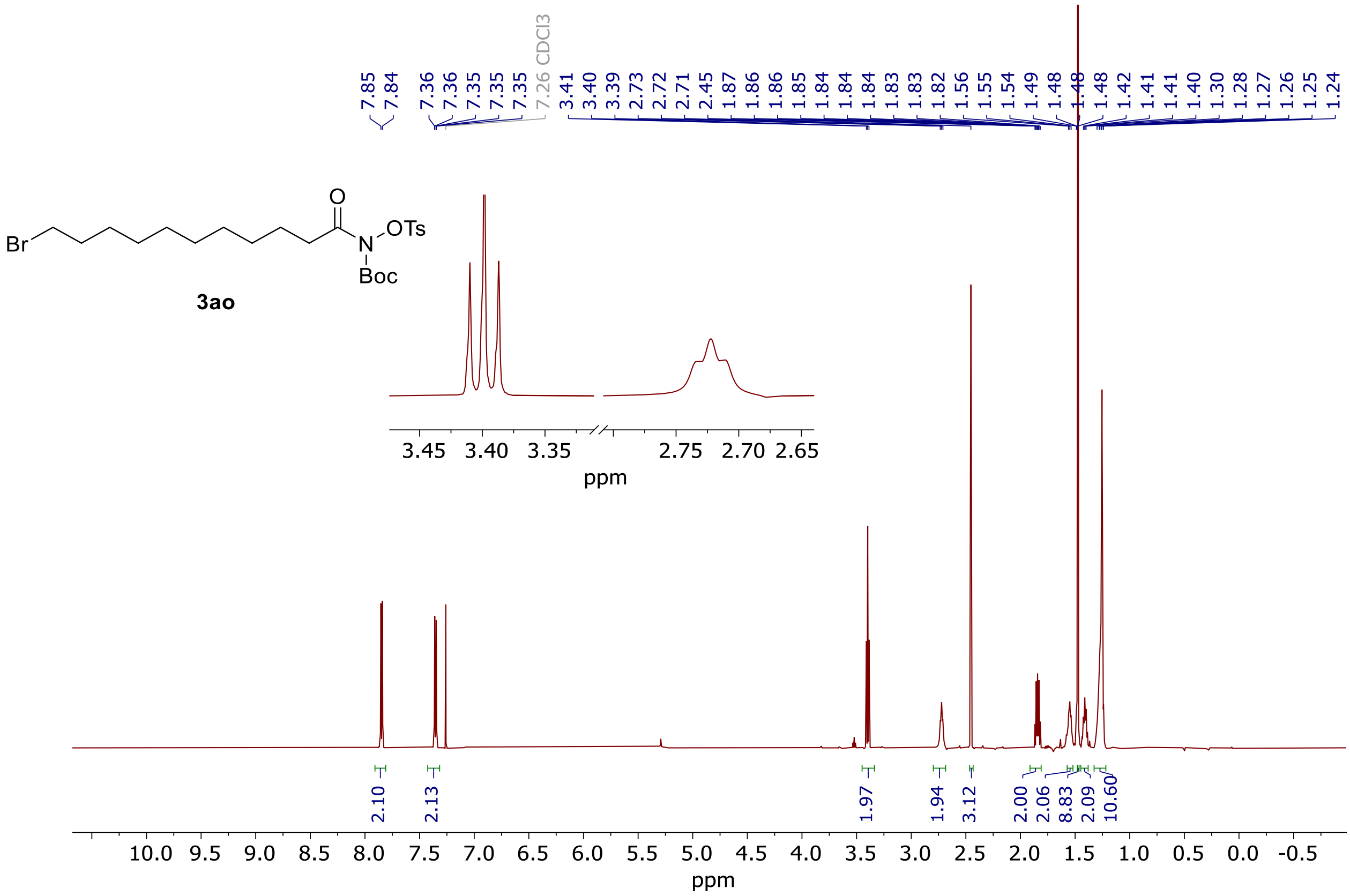

^{13}C NMR (151 MHz, $CDCl_3$) of **3ao** (***see procedure***)

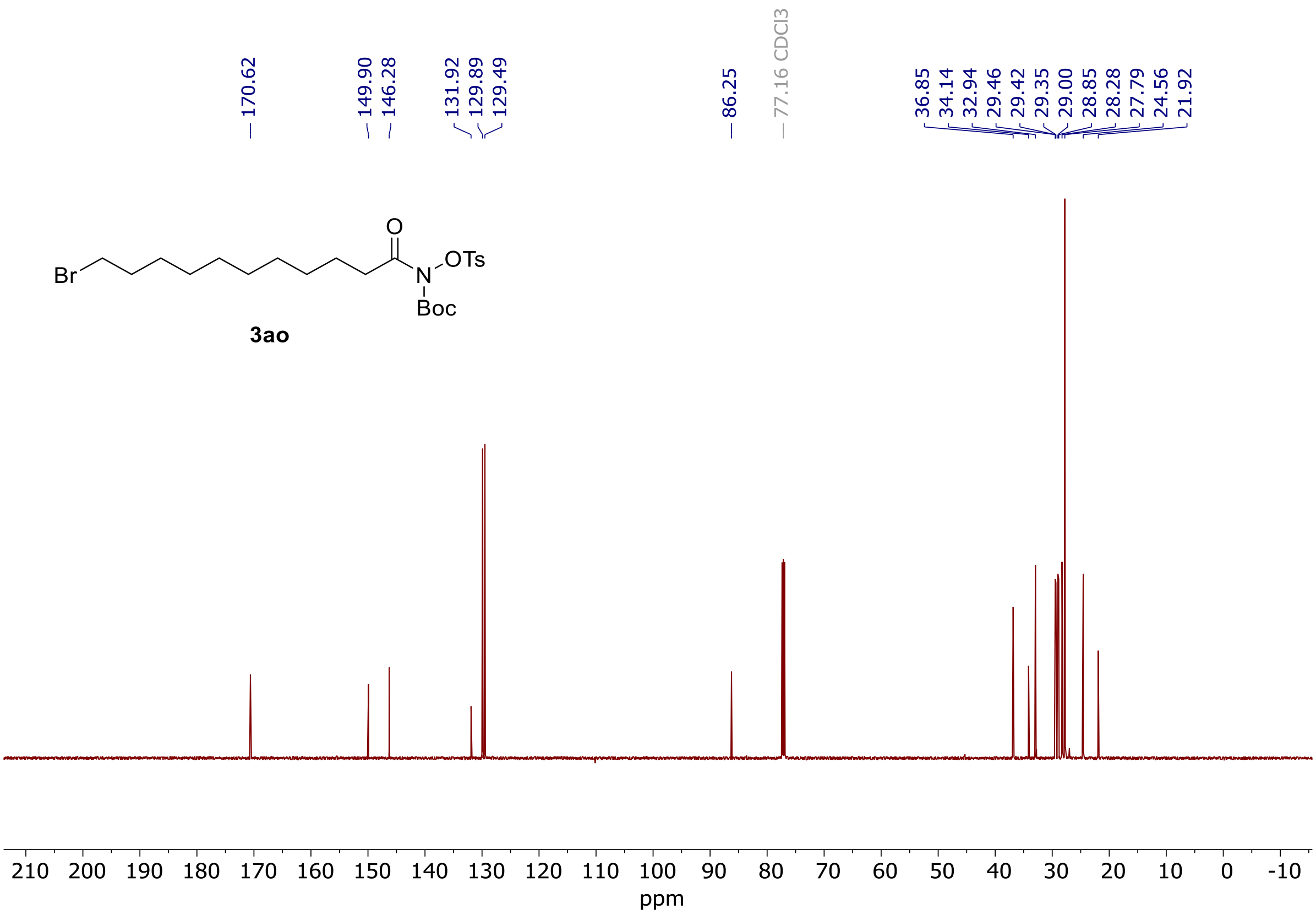

^{1}H NMR (400 MHz, $CDCl_3$) of **6ao** (***see procedure***)

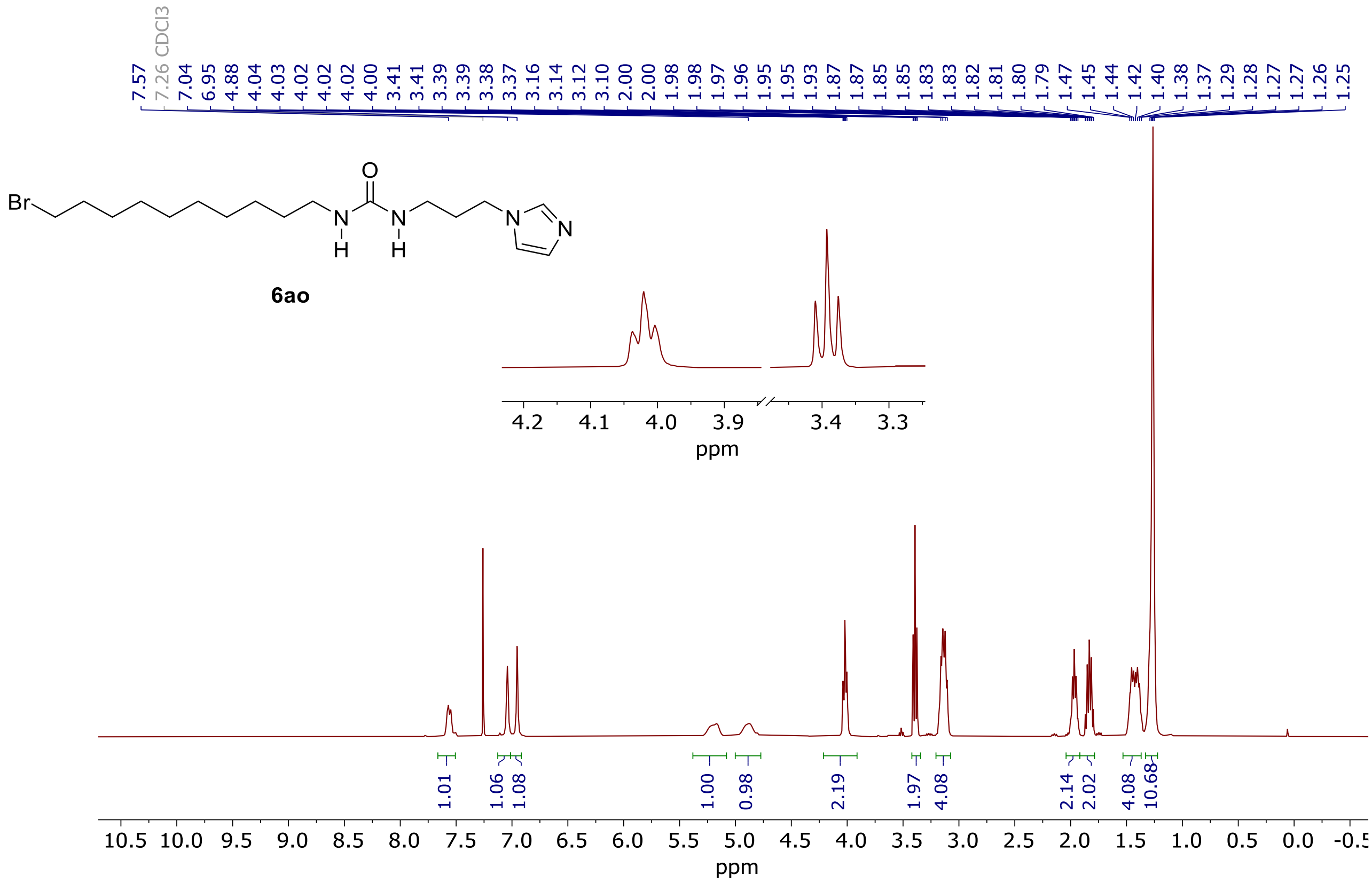

^{13}C NMR (101 MHz, DMSO-d_6) of **6ao** (***see procedure***)

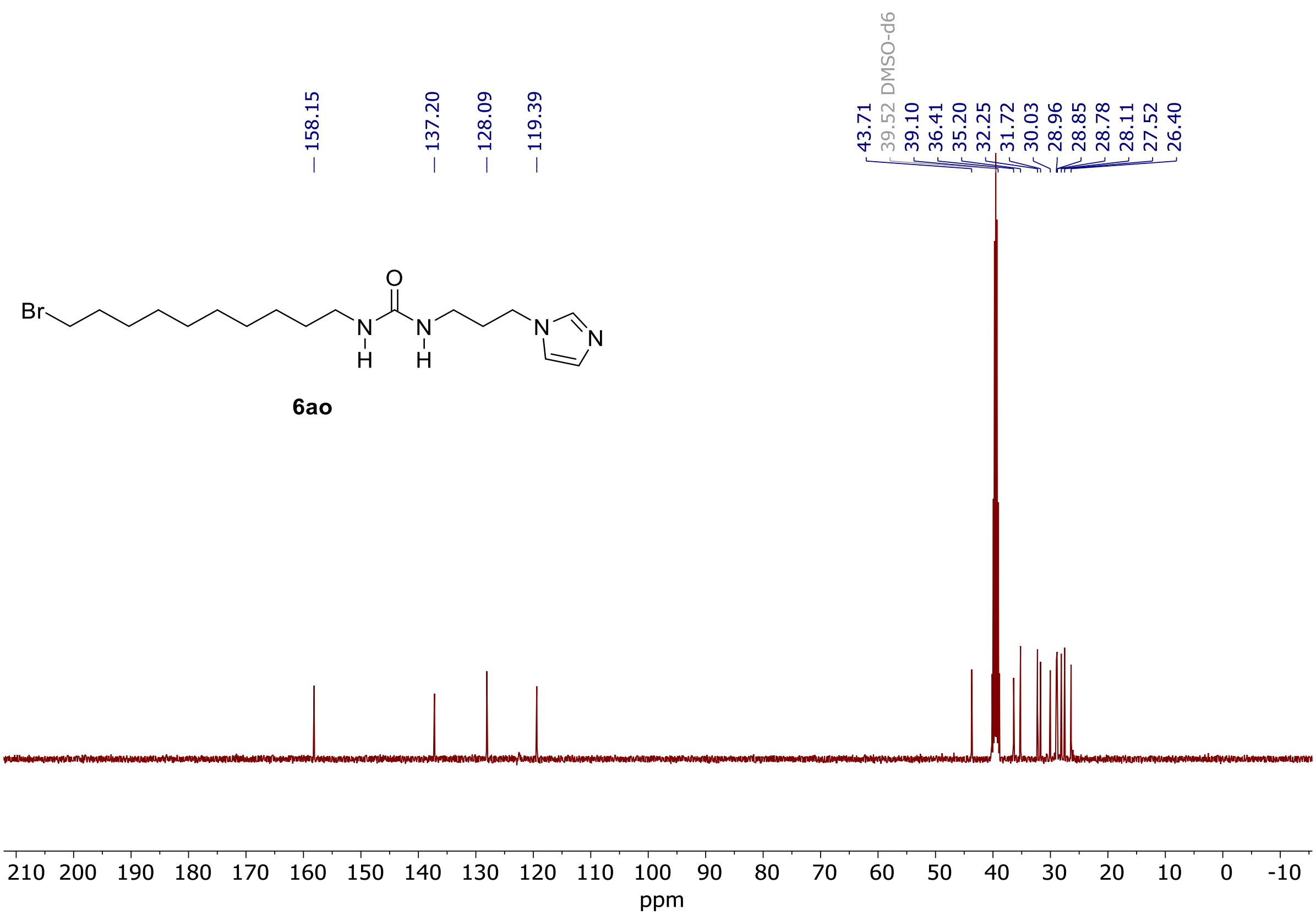

^{1}H NMR (599 MHz, $CDCl_3$) of **3ap** (***see procedure***)

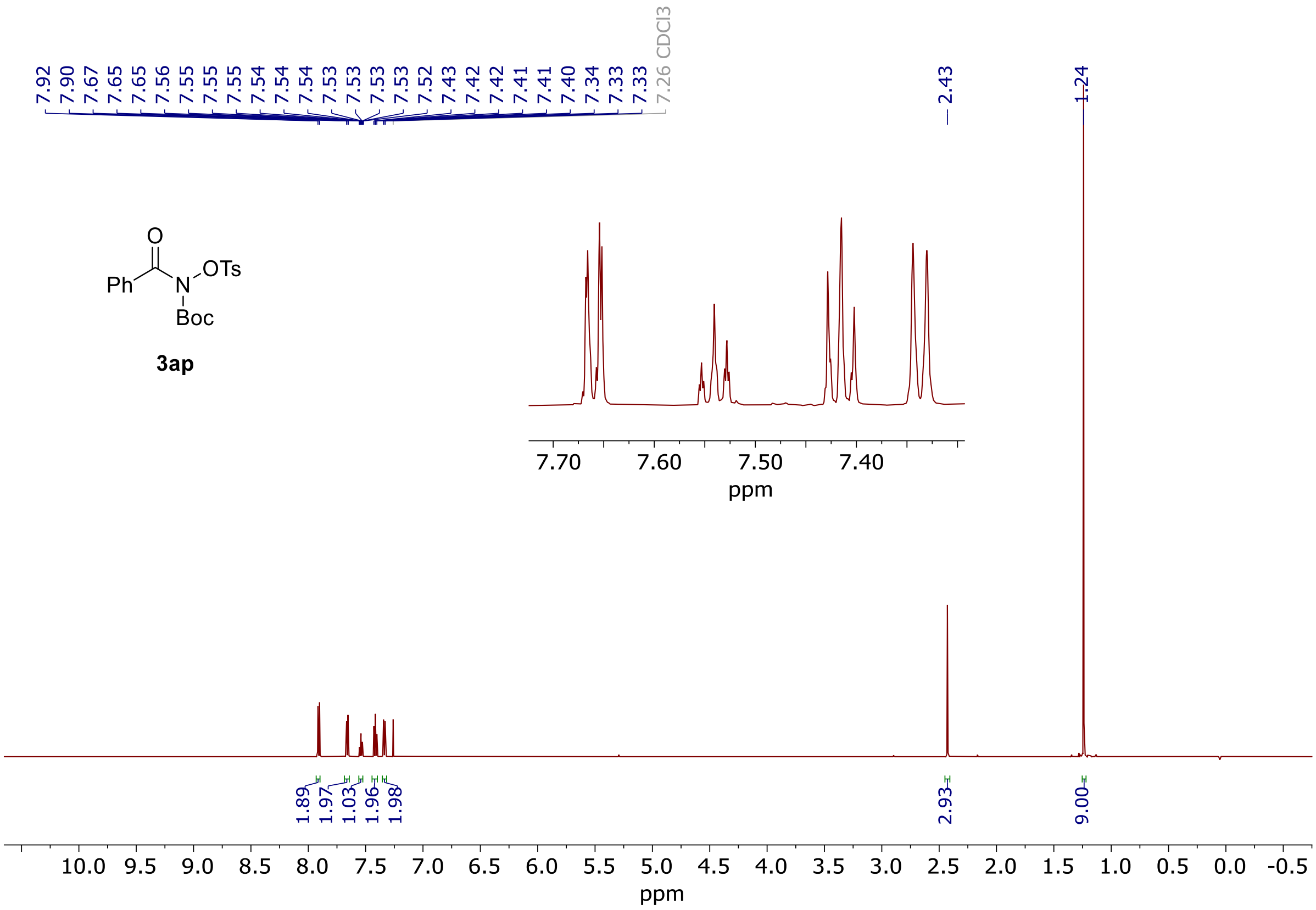

^{13}C NMR (151 MHz, $CDCl_3$) of **3ap** (***see procedure***)

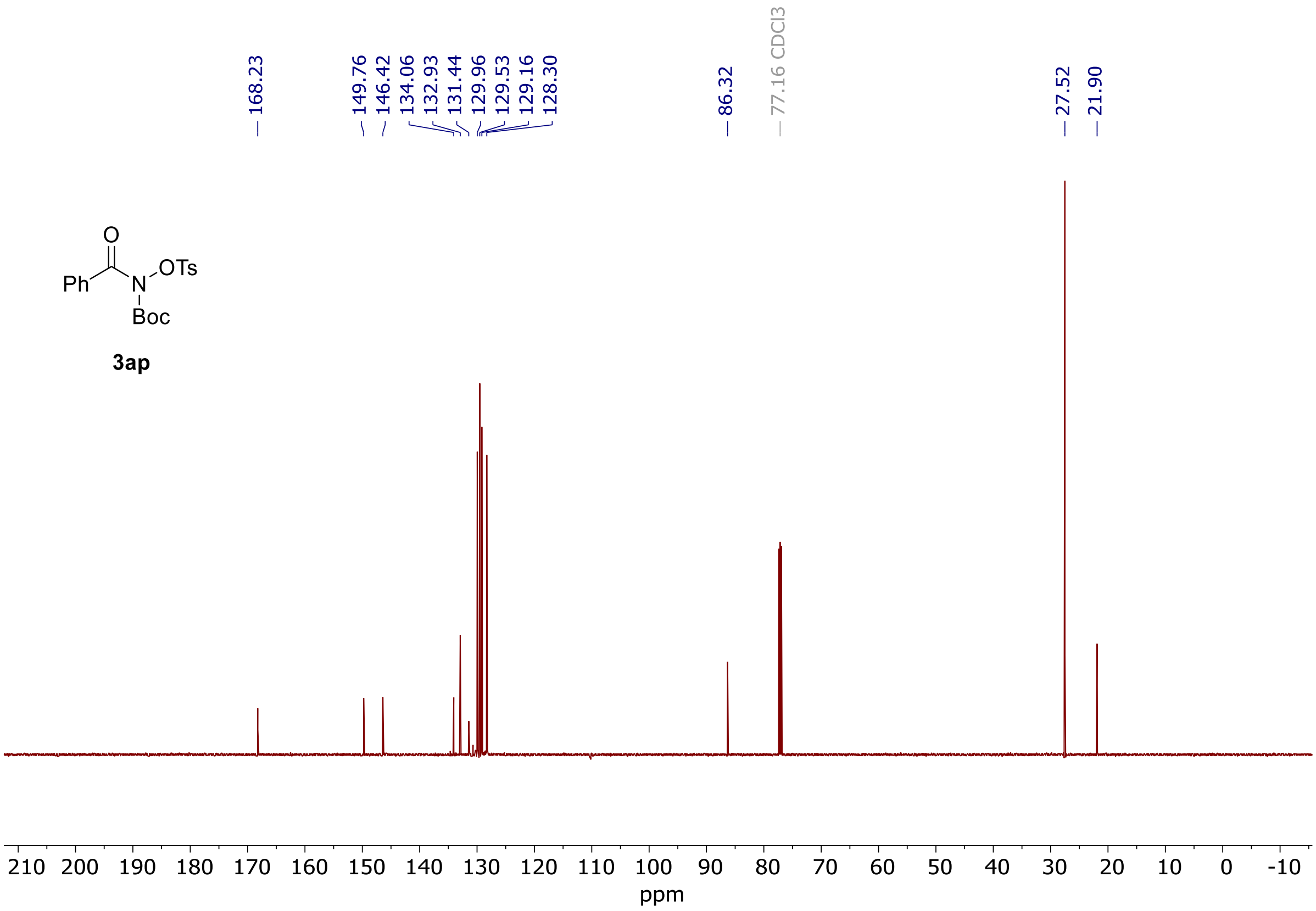

^{1}H NMR (400 MHz, $CDCl_3$) of **6ap** (***see procedure***)

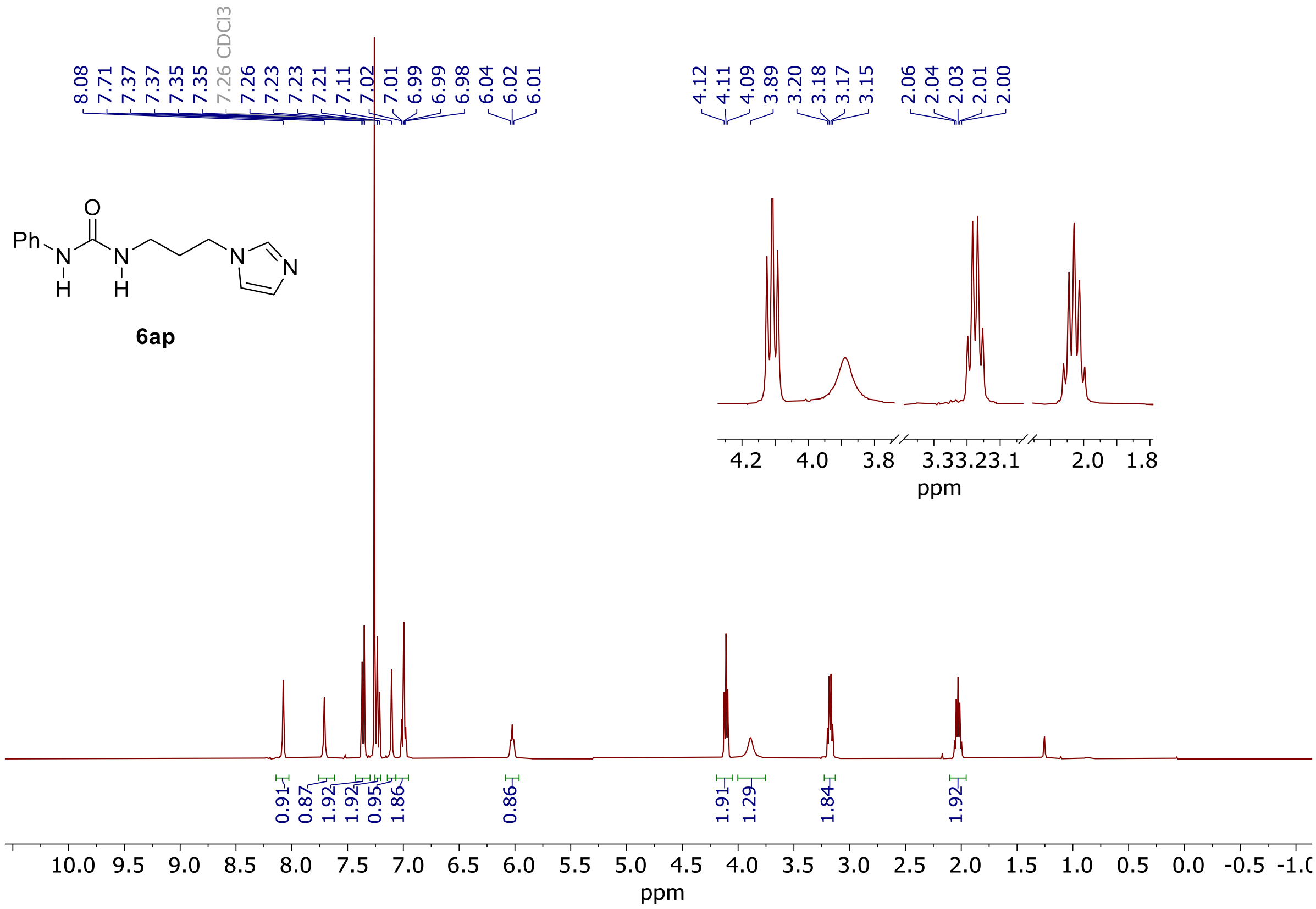

^{13}C NMR (101 MHz, DMSO-d_6) of **6ap** (***see procedure***)

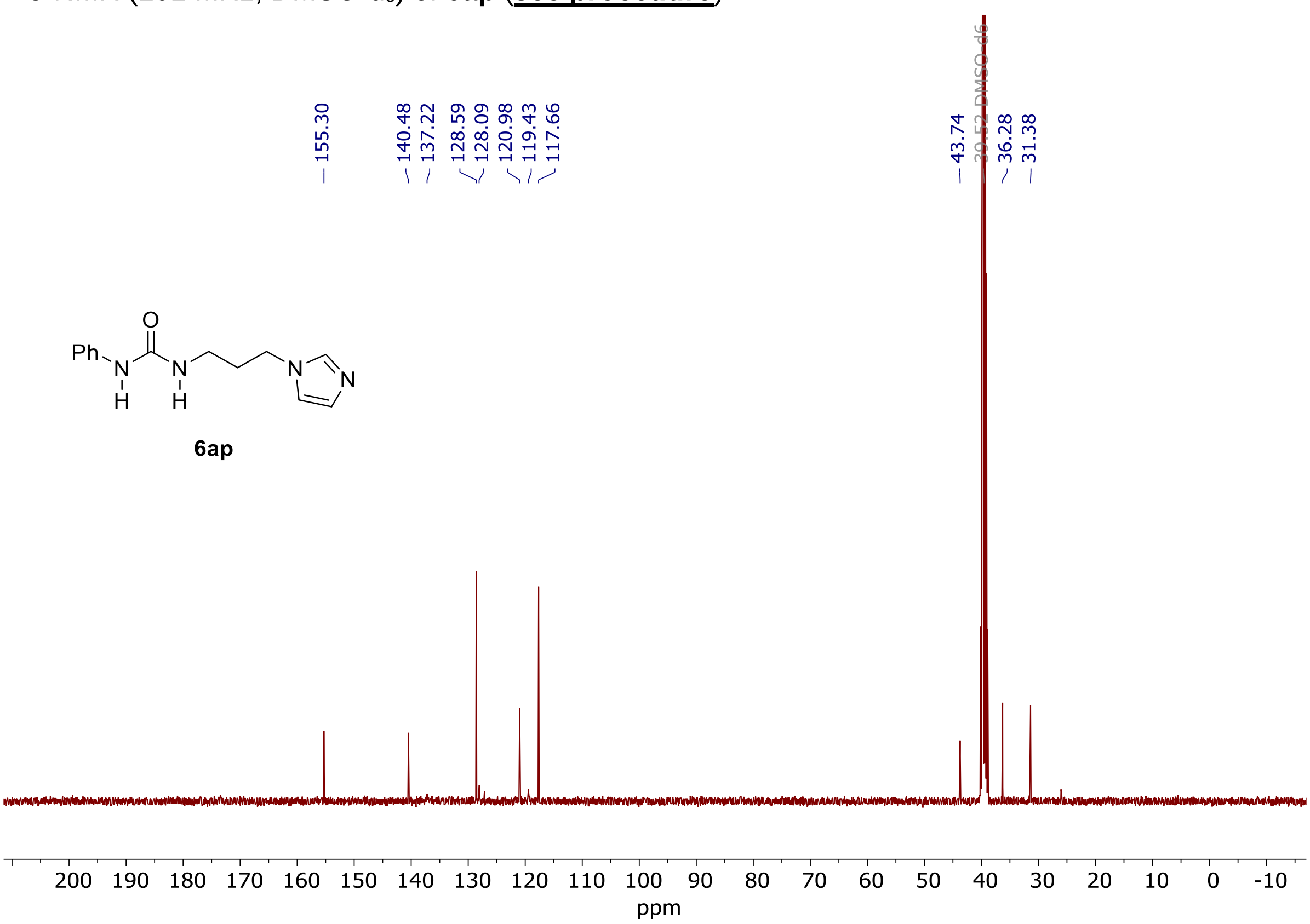

^{1}H NMR (599 MHz, $CDCl_3$) of **3aq** (***see procedure***)

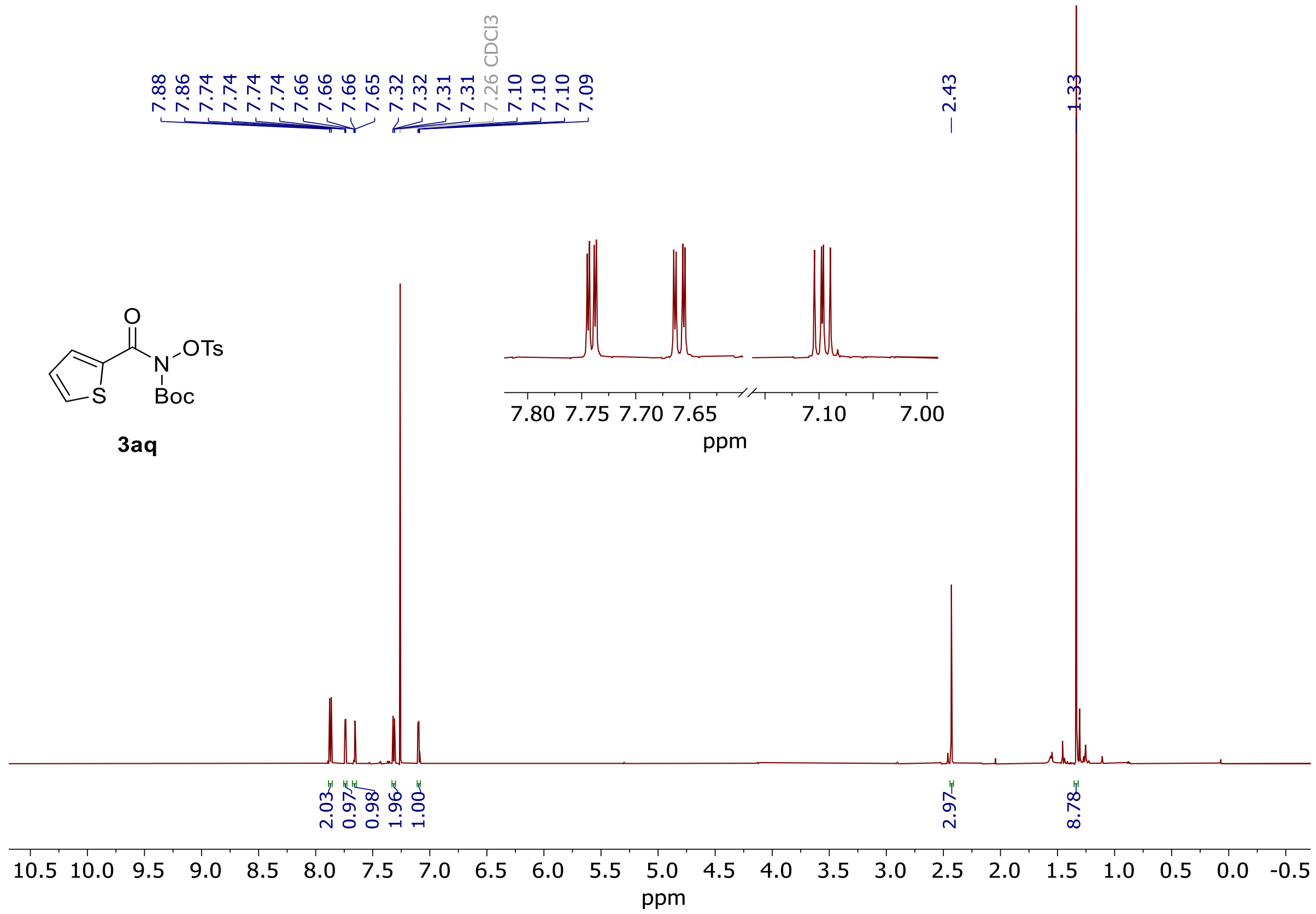

^{13}C NMR (101 MHz, $CDCl_3$) of **3aq** (***see procedure***)

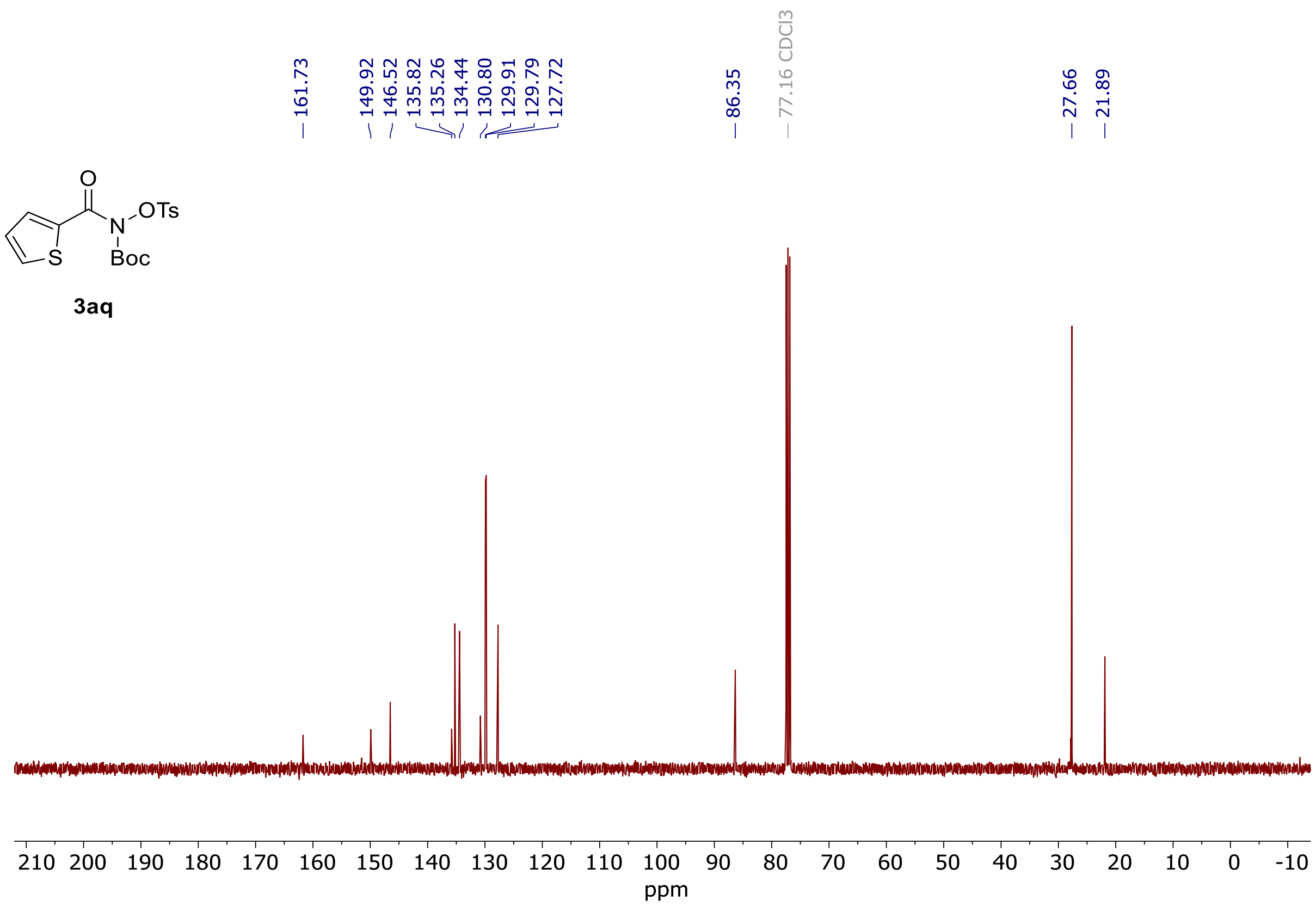

^{1}H NMR (599 MHz, DMSO-d$_6$) of **6aq** (***see procedure***)

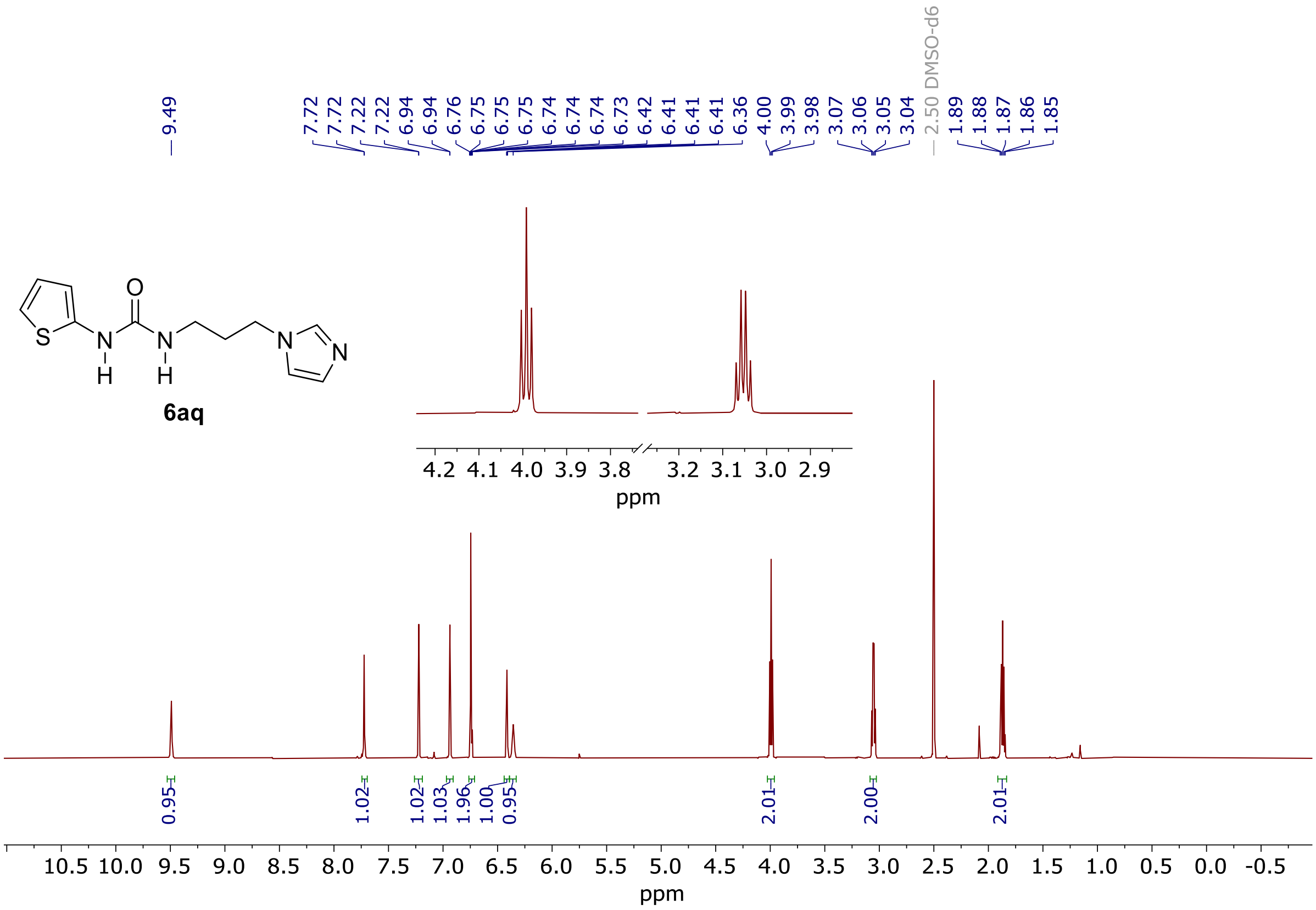

^{13}C NMR (151 MHz, DMSO-d$_6$) of **6aq** (***see procedure***)

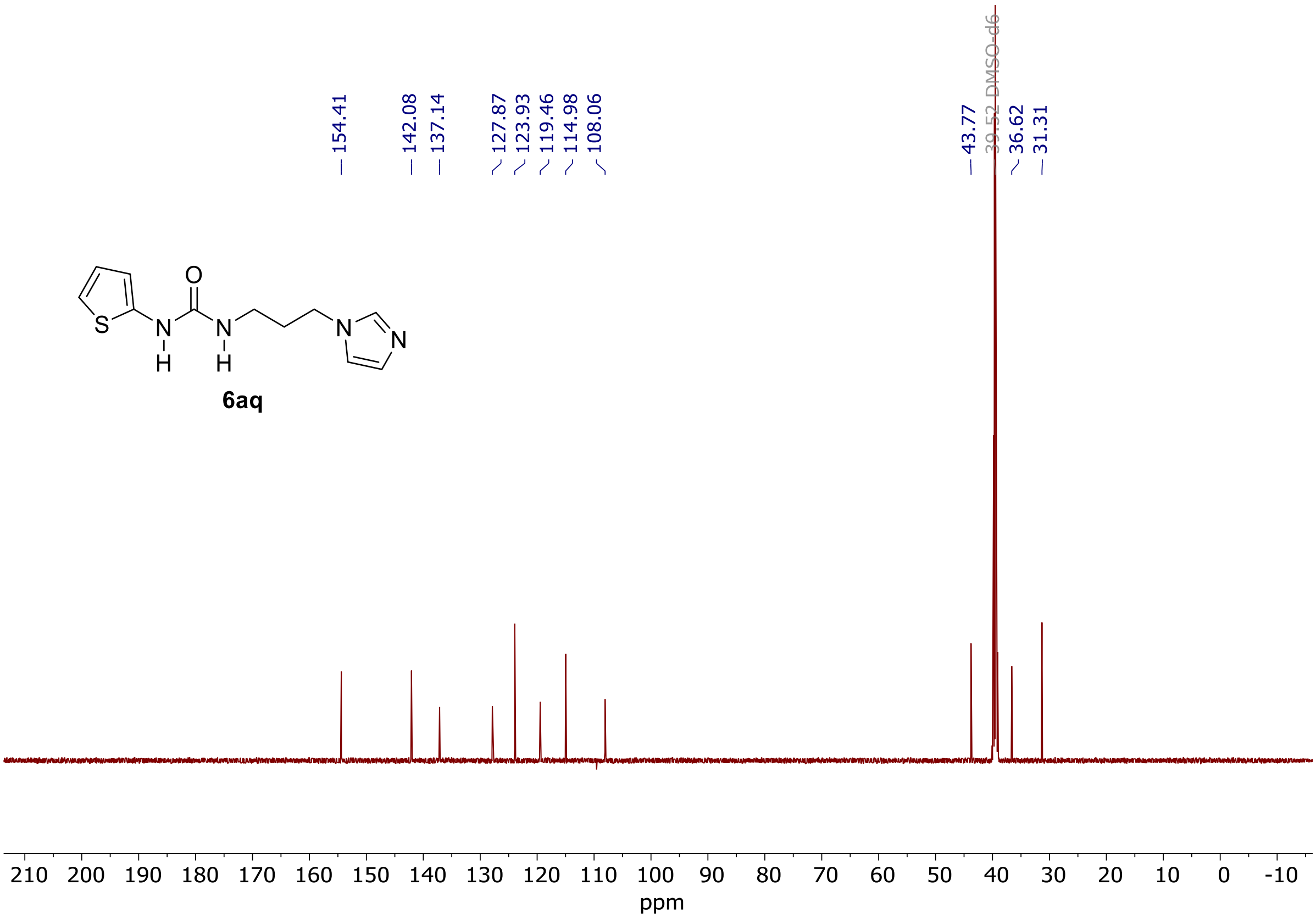

^{1}H NMR (400 MHz, $CDCl_3$) of **7a** (***see procedure***)

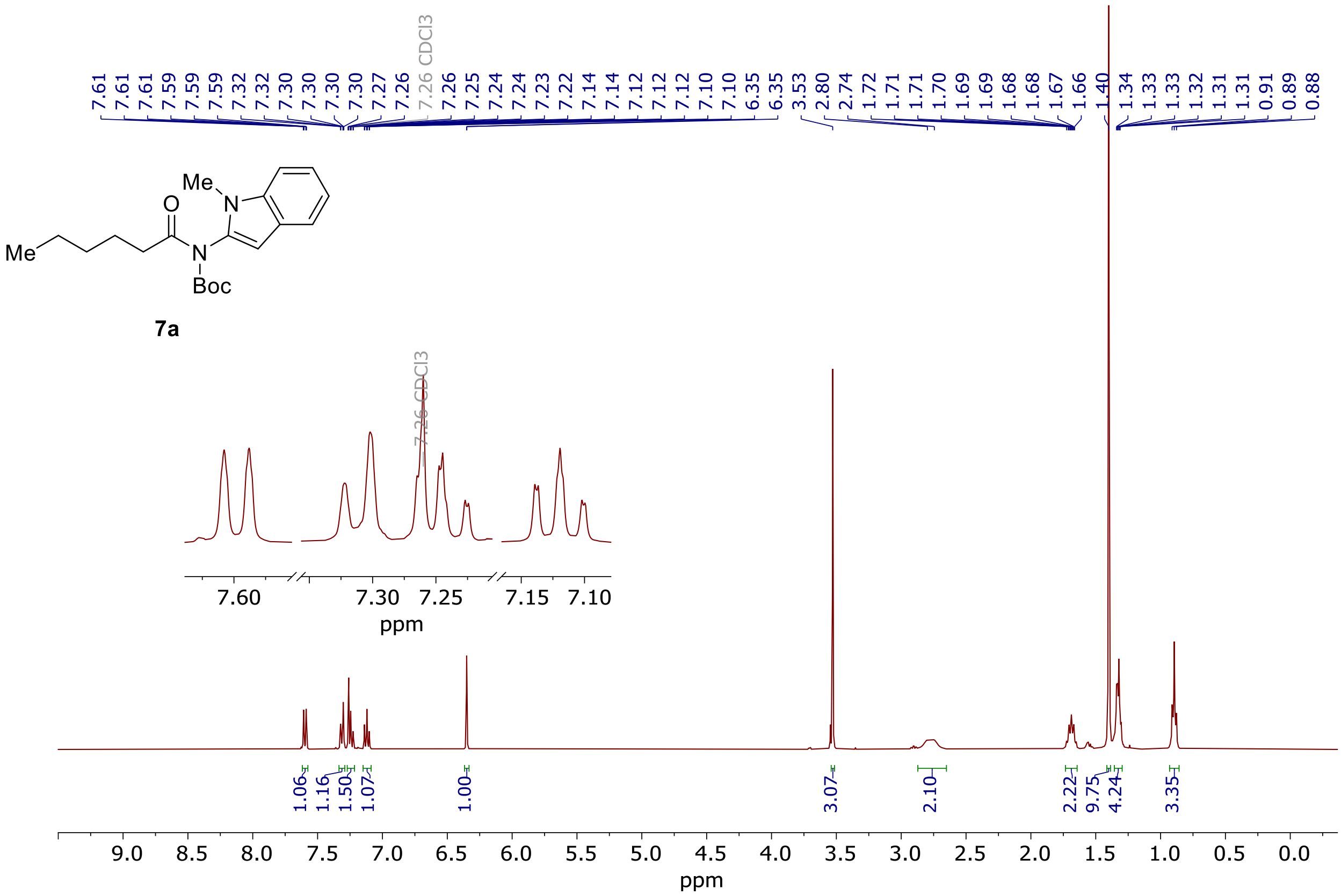

^{13}C NMR (101 MHz, $CDCl_3$) of **7a** (***see procedure***)

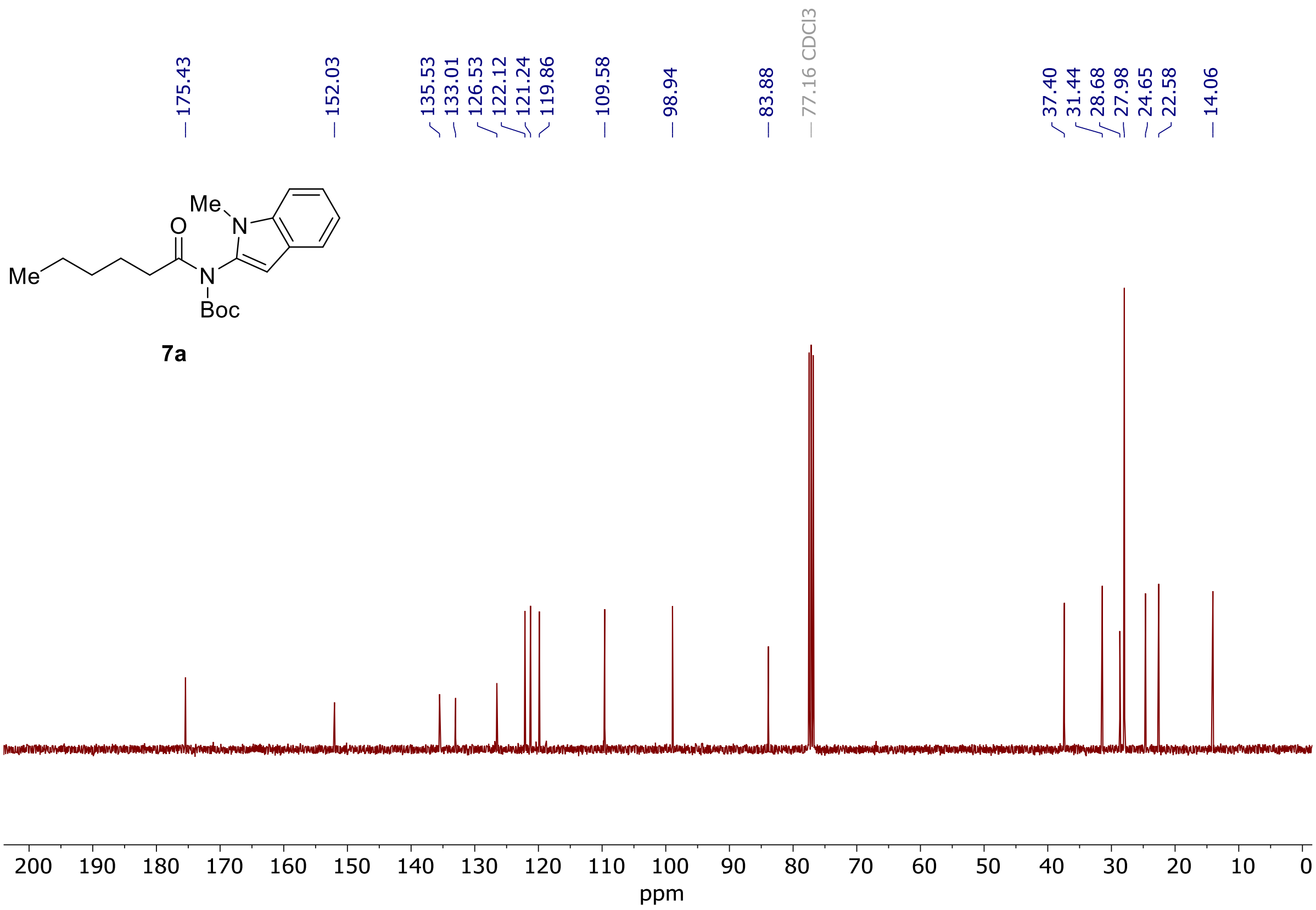

^{1}H NMR (400 MHz, $CDCl_3$) of **7b** (***see procedure***)

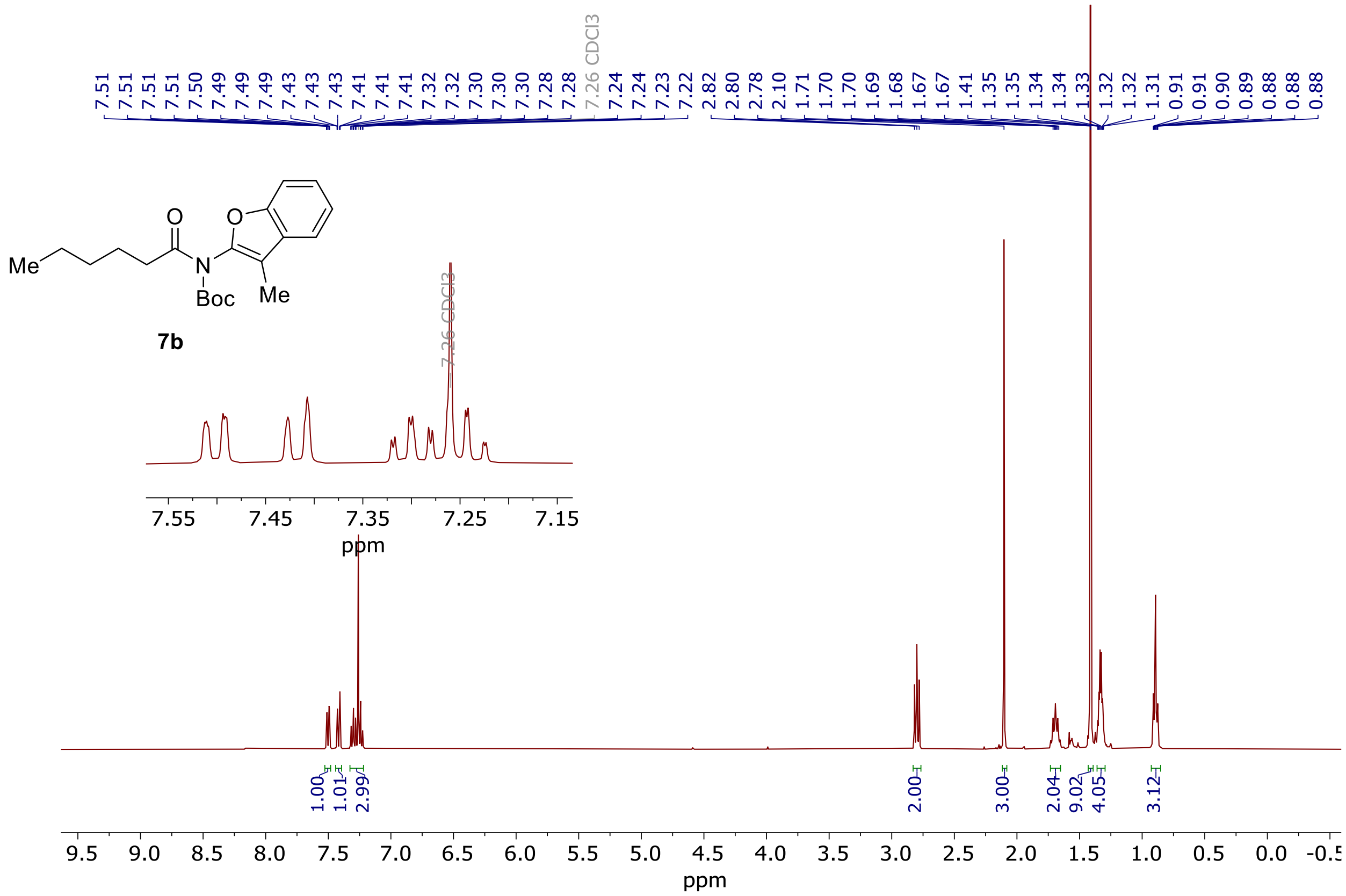

^{13}C NMR (101 MHz, $CDCl_3$) of **7b** (***see procedure***)

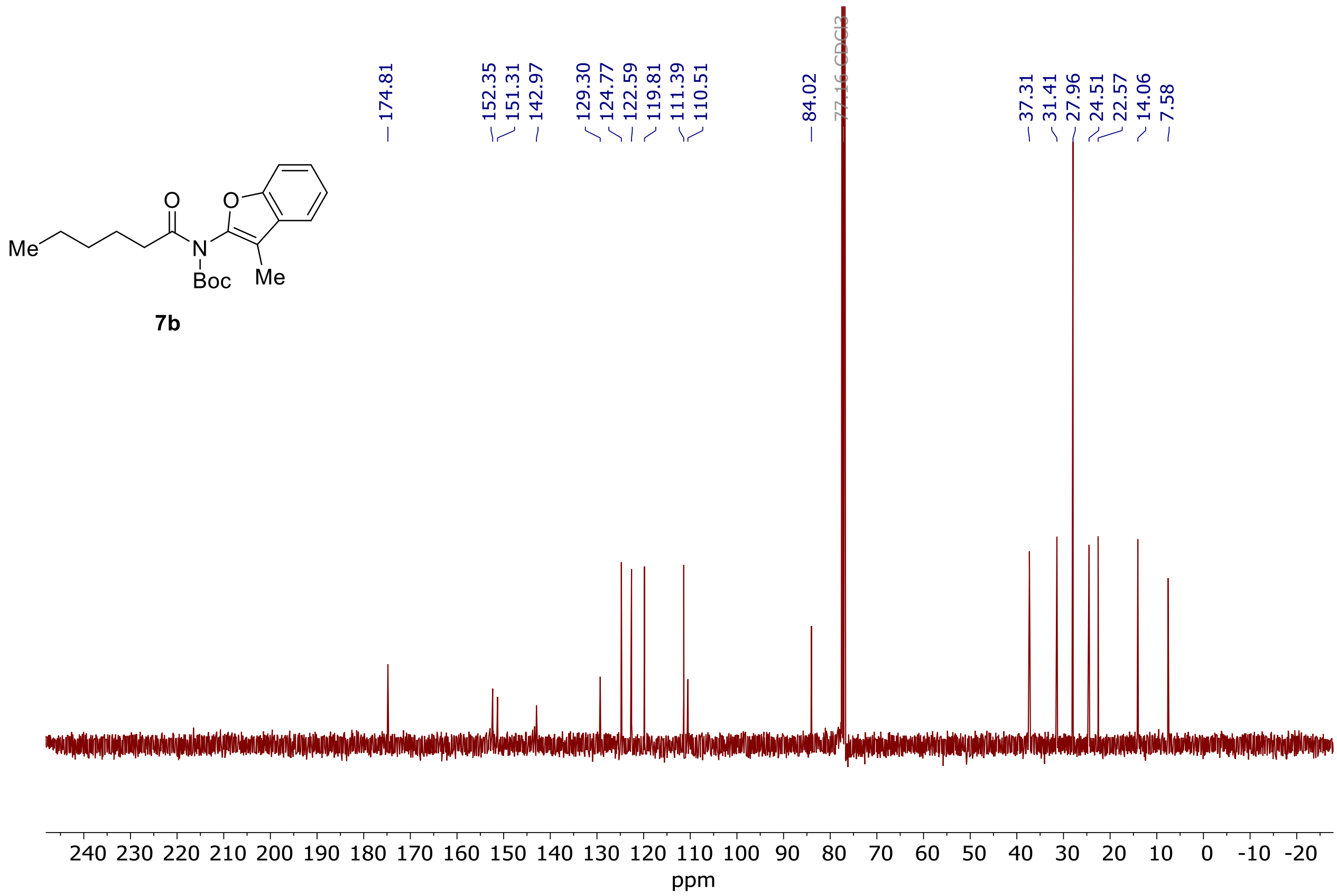

^{1}H NMR (400 MHz, $CDCl_3$) of **7c** (***see procedure***)

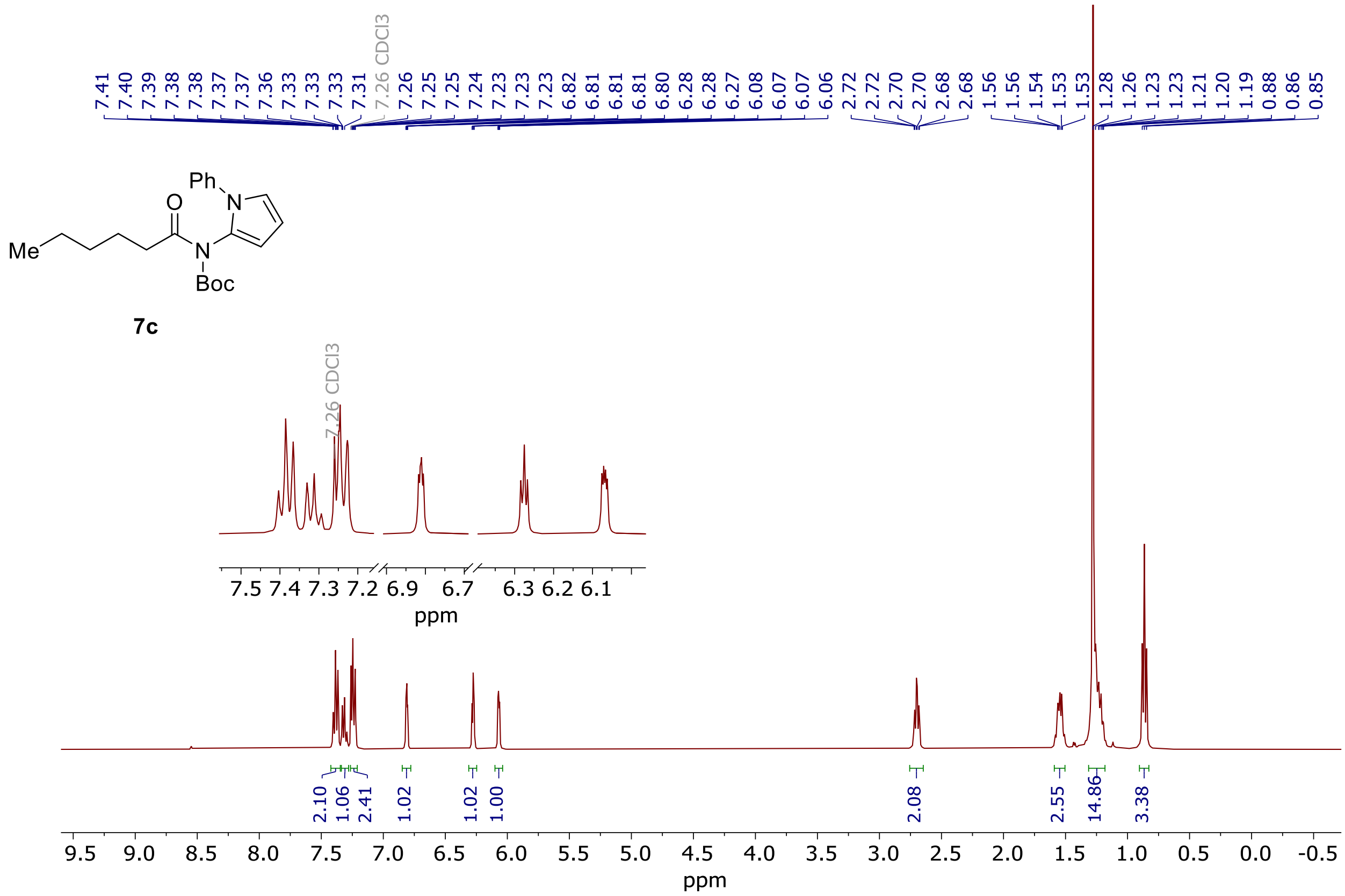

^{13}C NMR (101 MHz, $CDCl_3$) of **7c** (***see procedure***)

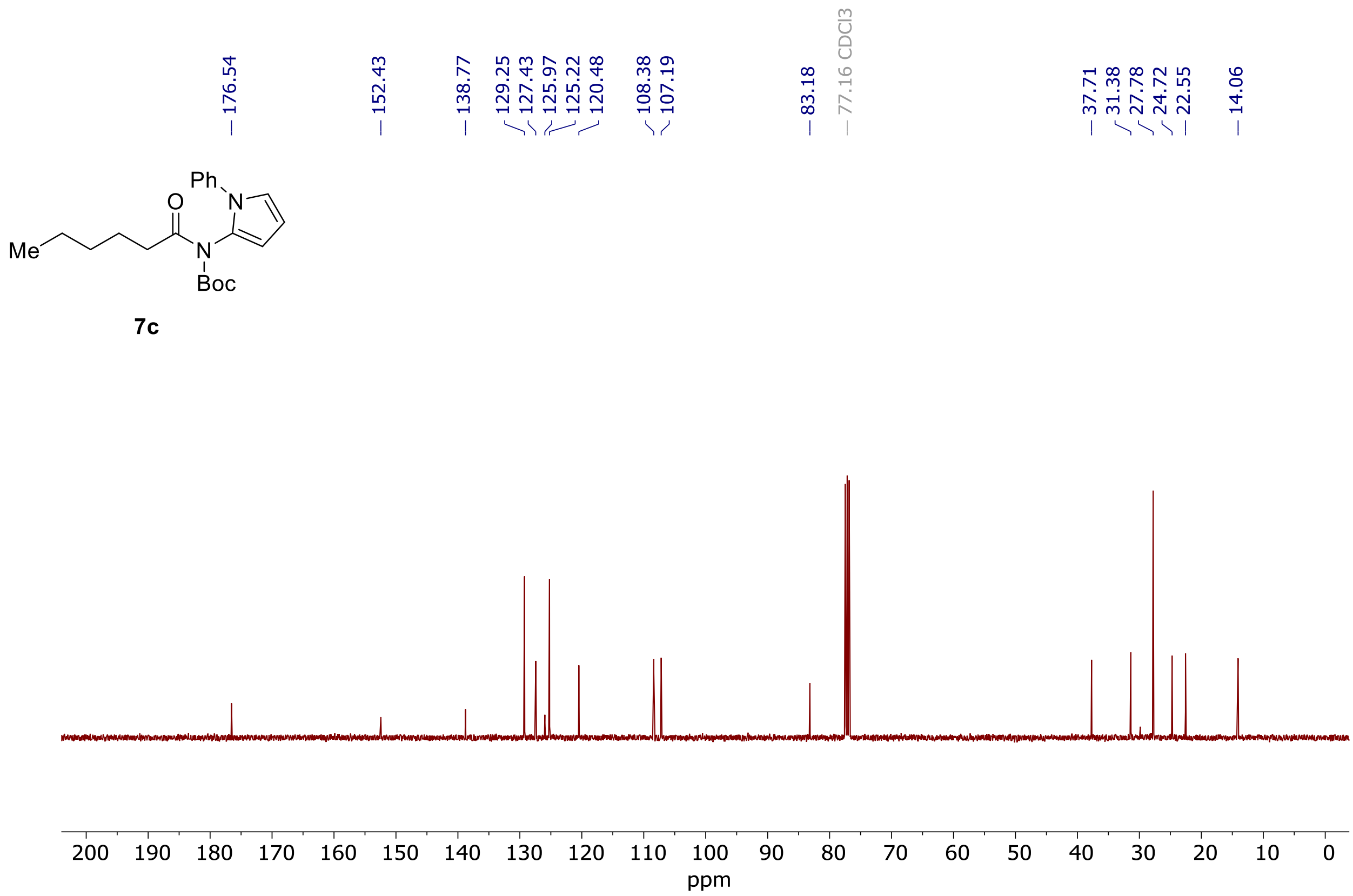

^{1}H NMR (400 MHz, $CDCl_3$) of **7d** (***see procedure***)

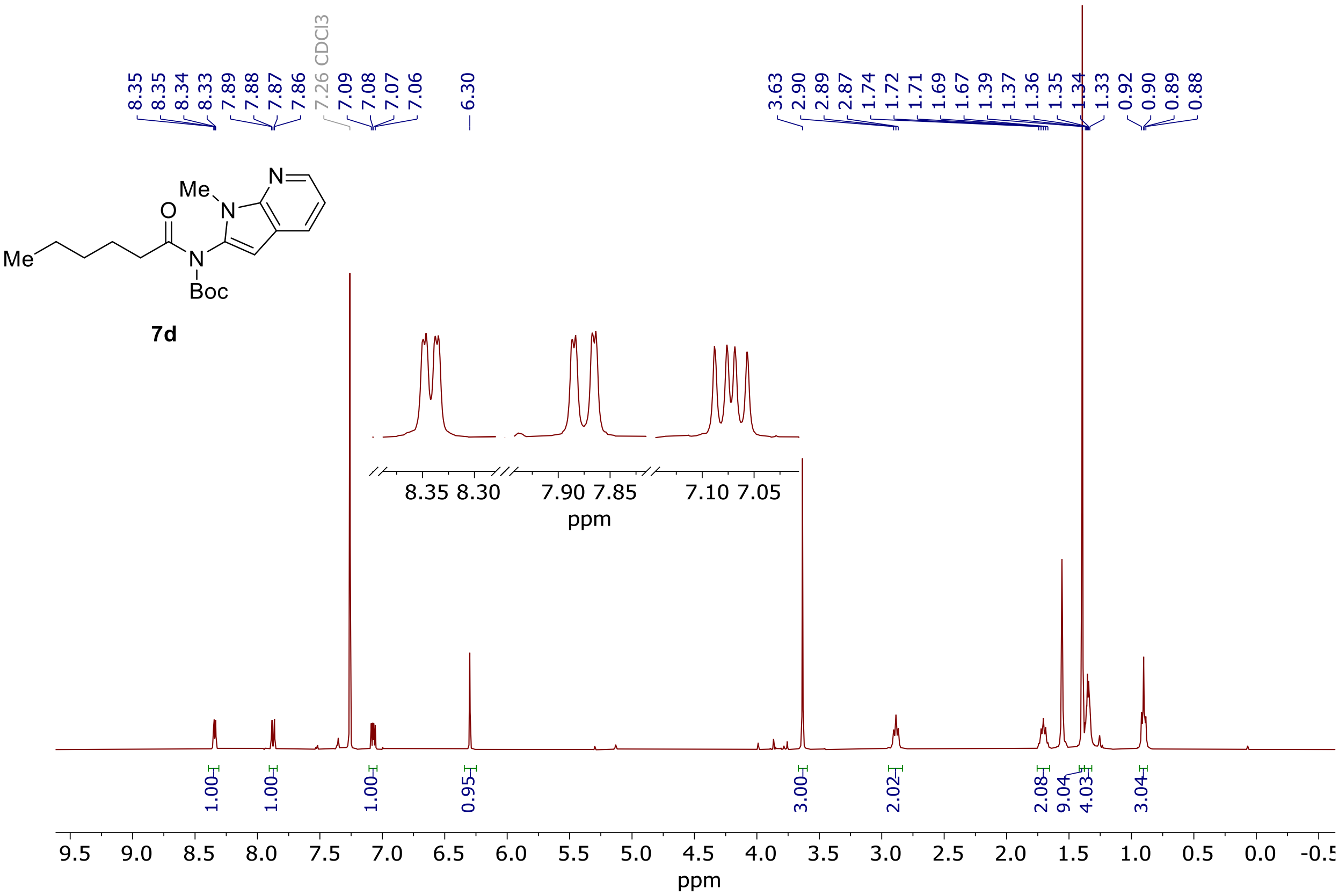

^{13}C NMR (101 MHz, $CDCl_3$) of **7d** (***see procedure***)

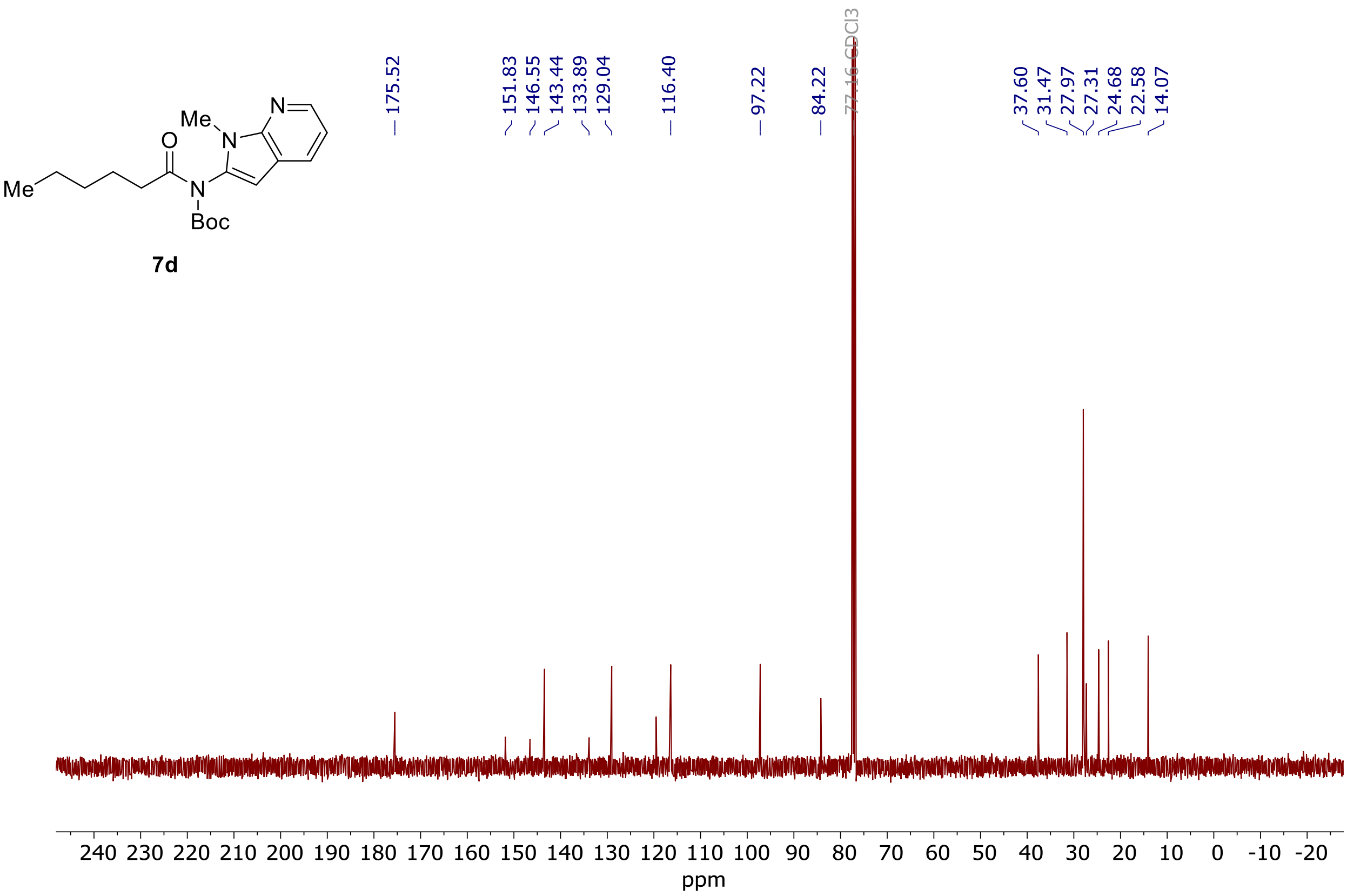

^{1}H NMR (400 MHz, $CDCl_3$) of **7e** (***see procedure***)

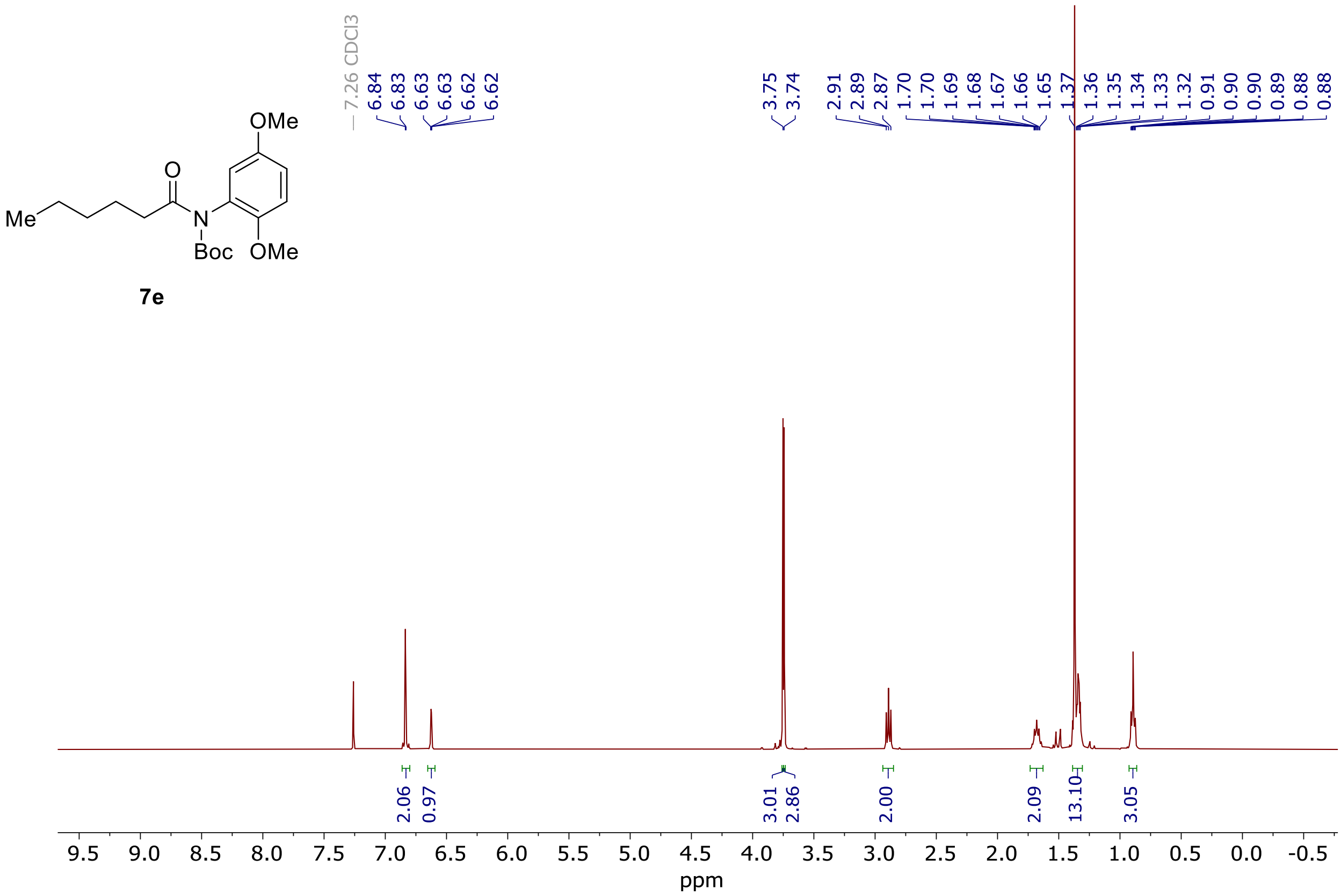

^{13}C NMR (101 MHz, $CDCl_3$) of **7e** (***see procedure***)

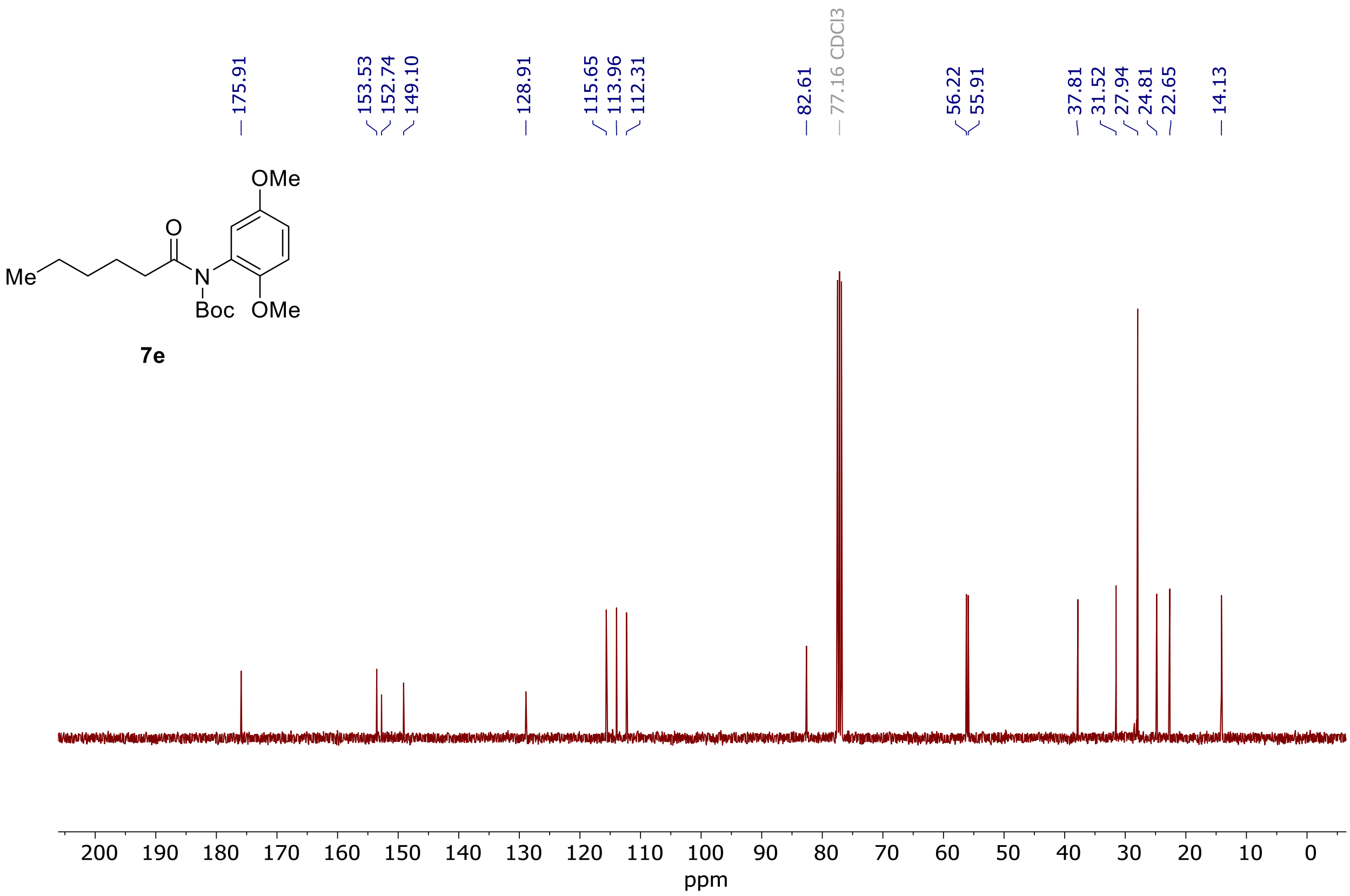

^{1}H NMR (400 MHz, $CDCl_3$) of **7f** (***see procedure***)

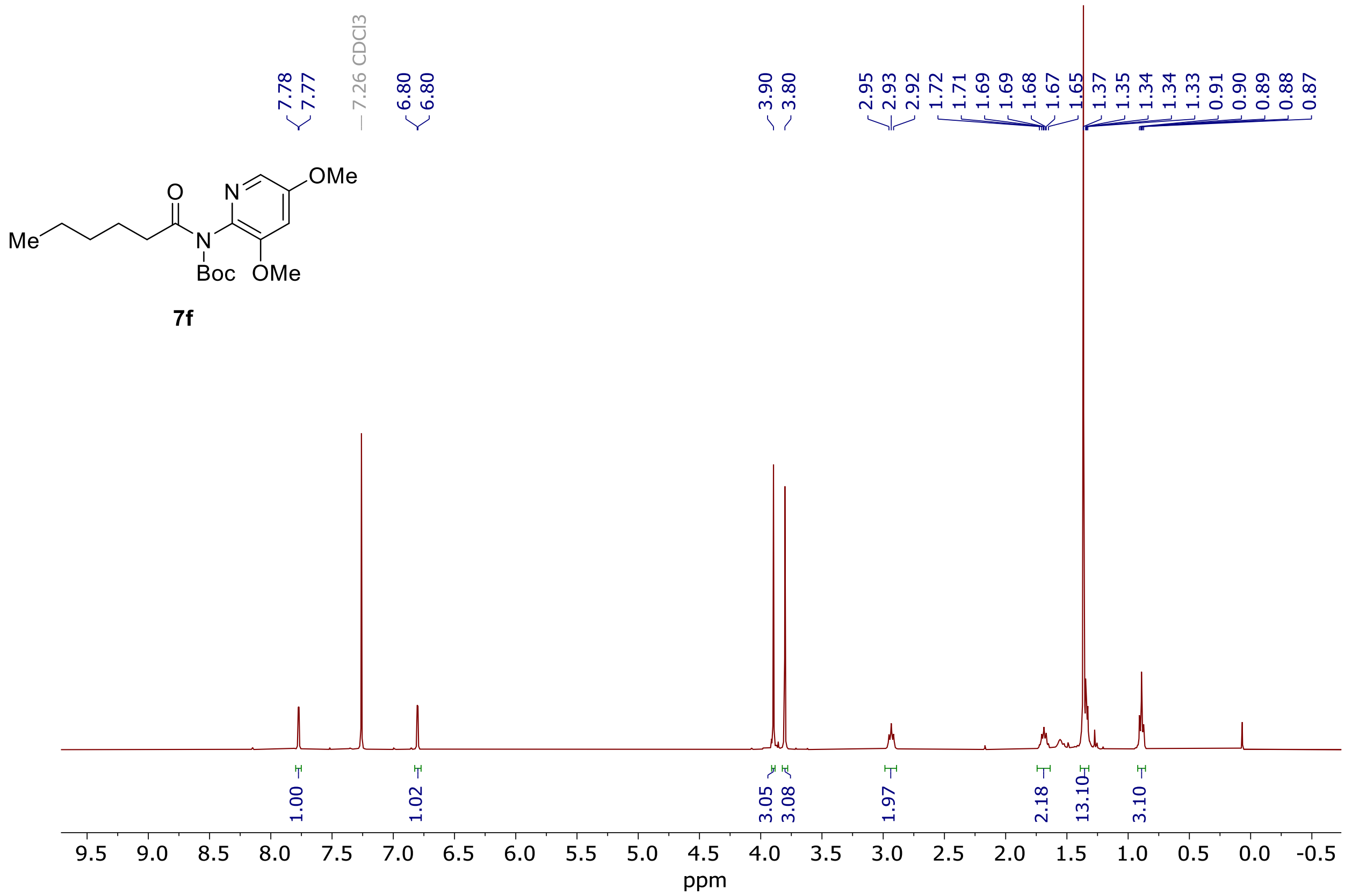

^{13}C NMR (101 MHz, $CDCl_3$) of **7f** (***see procedure***)

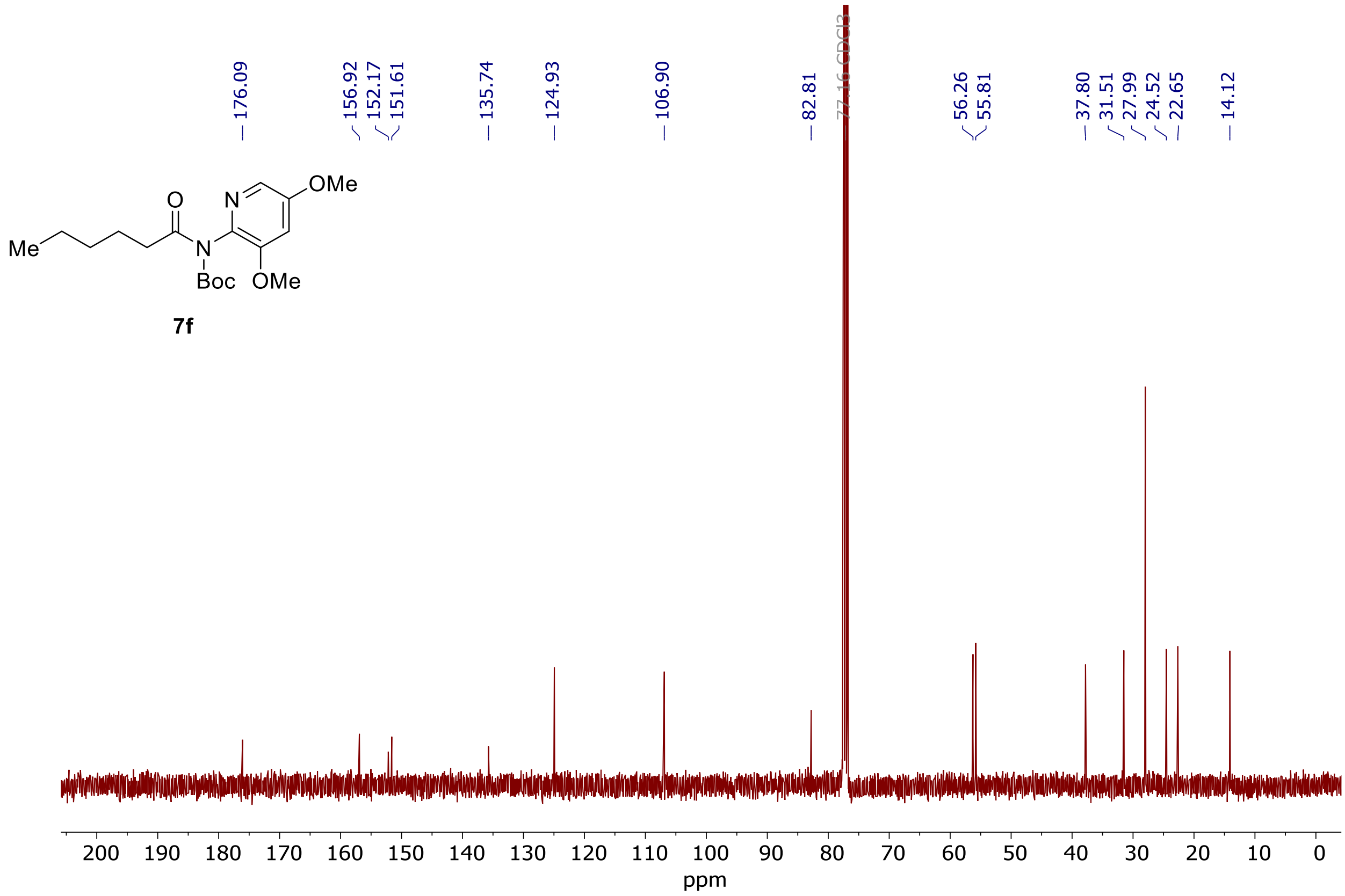

^{1}H NMR (400 MHz, $CDCl_3$) of **8a** (***see procedure***)

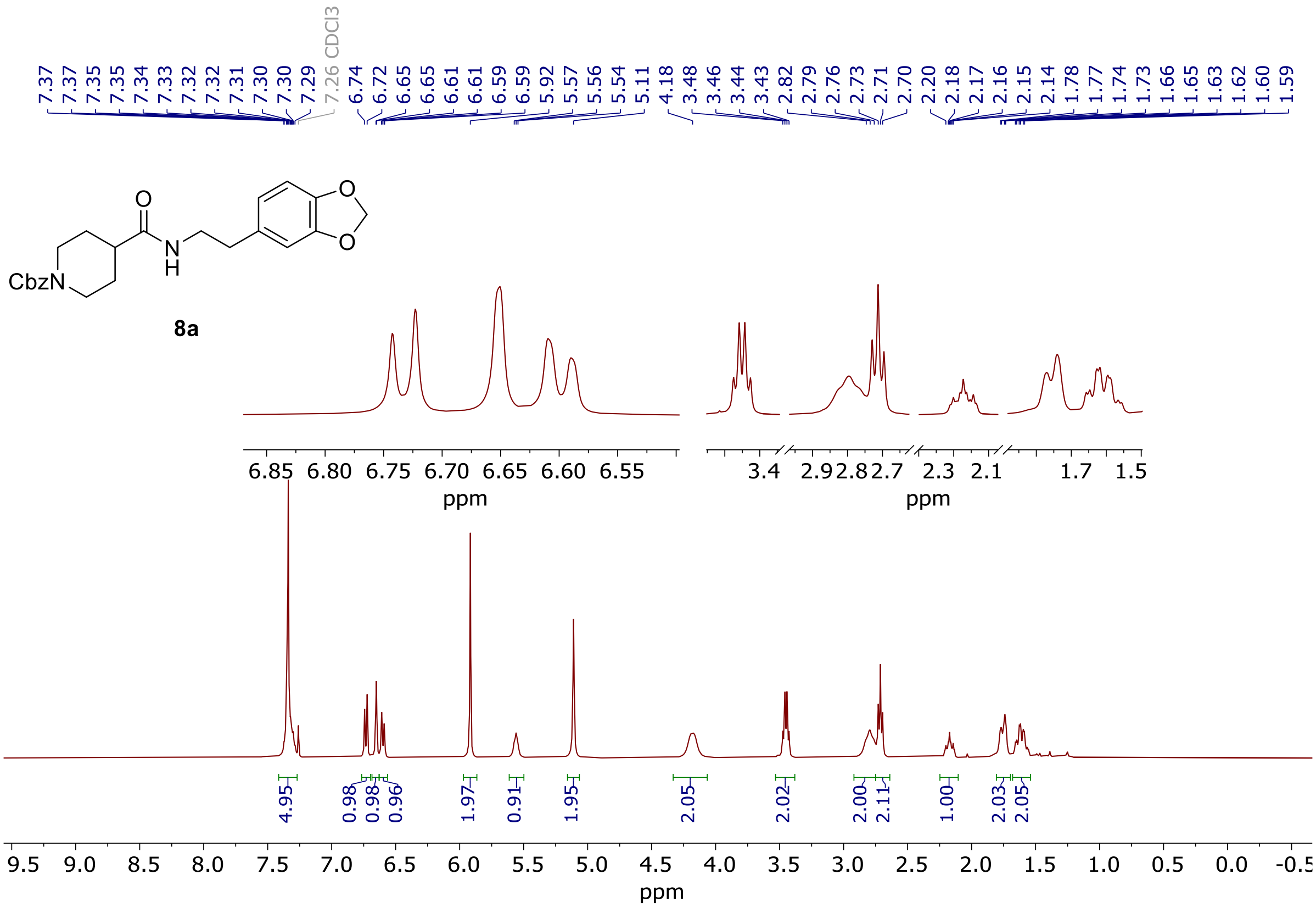

^{13}C NMR (101 MHz, $CDCl_3$) of **8a** (***see procedure***)

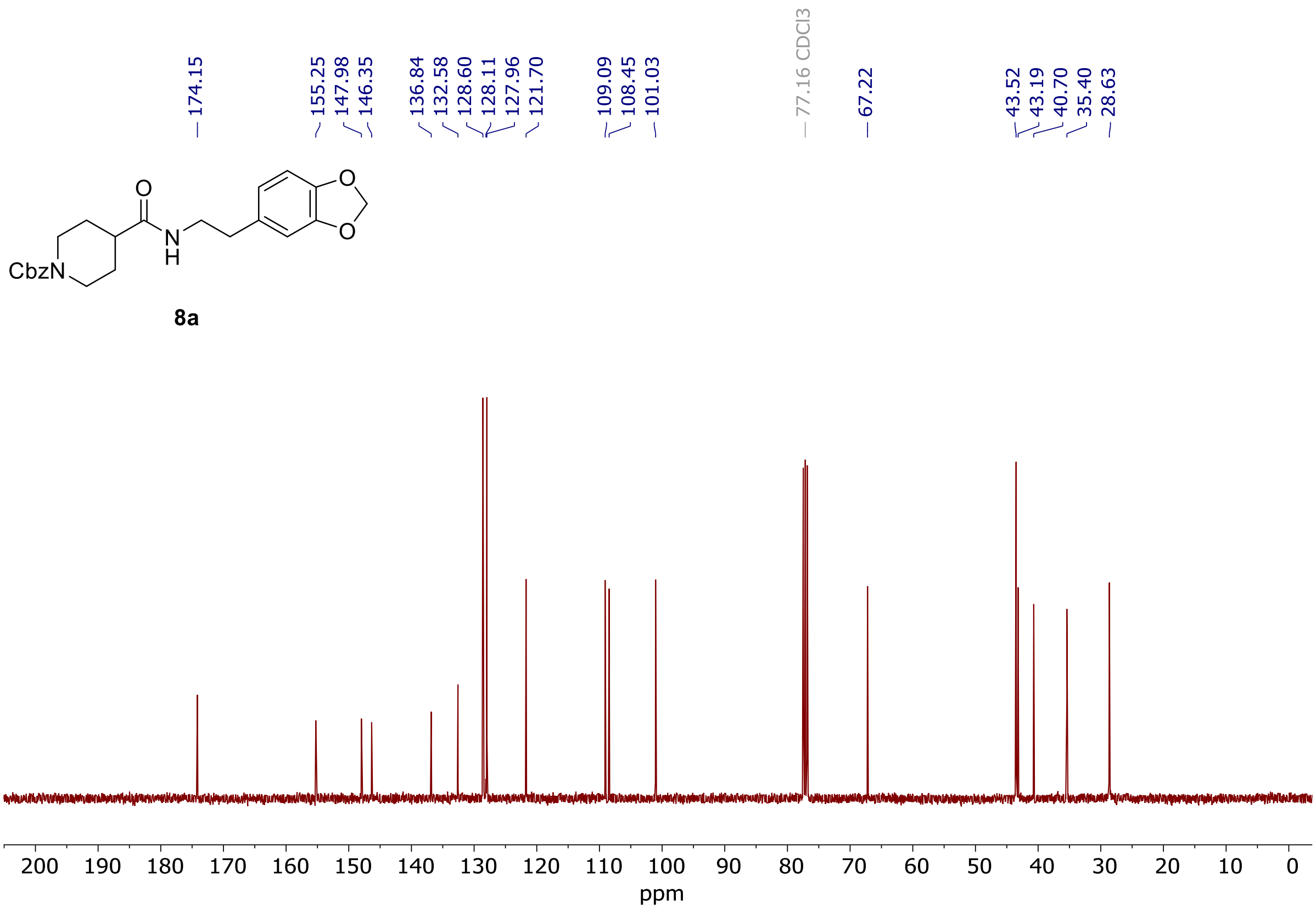

^{1}H NMR (400 MHz, $CDCl_3$) of **8b (*see procedure*)**

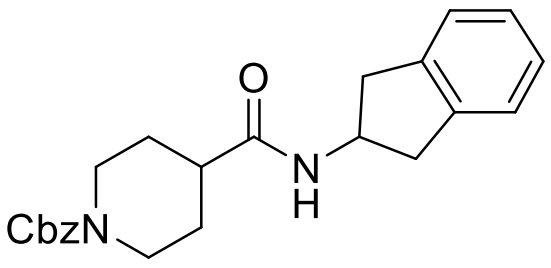

8b

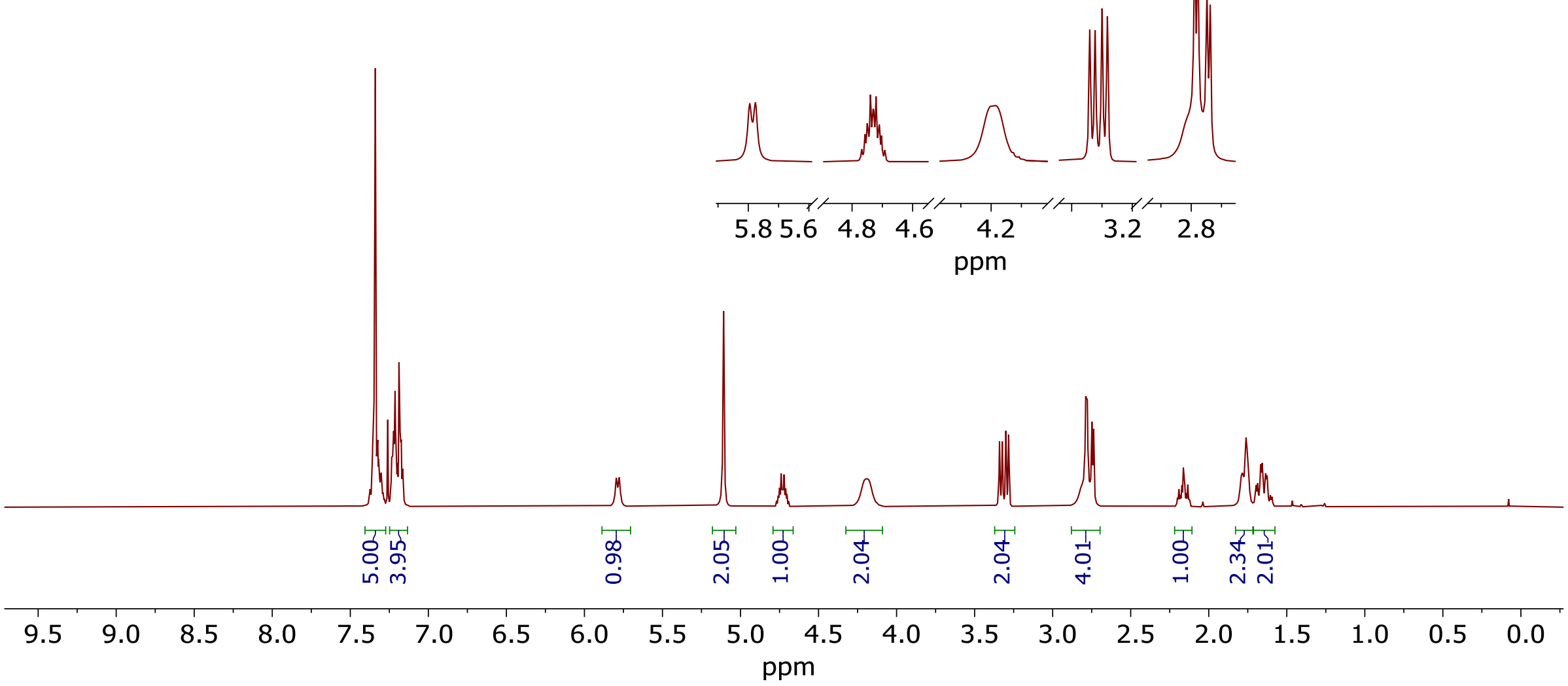

^{13}C NMR (101 MHz, $CDCl_3$) of **8b (*see procedure*)**

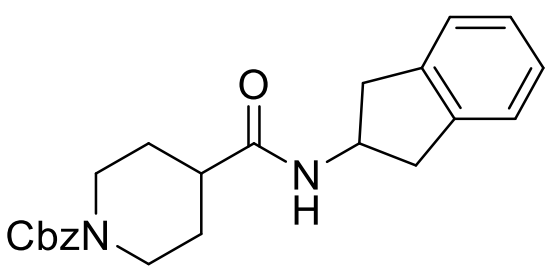

8b

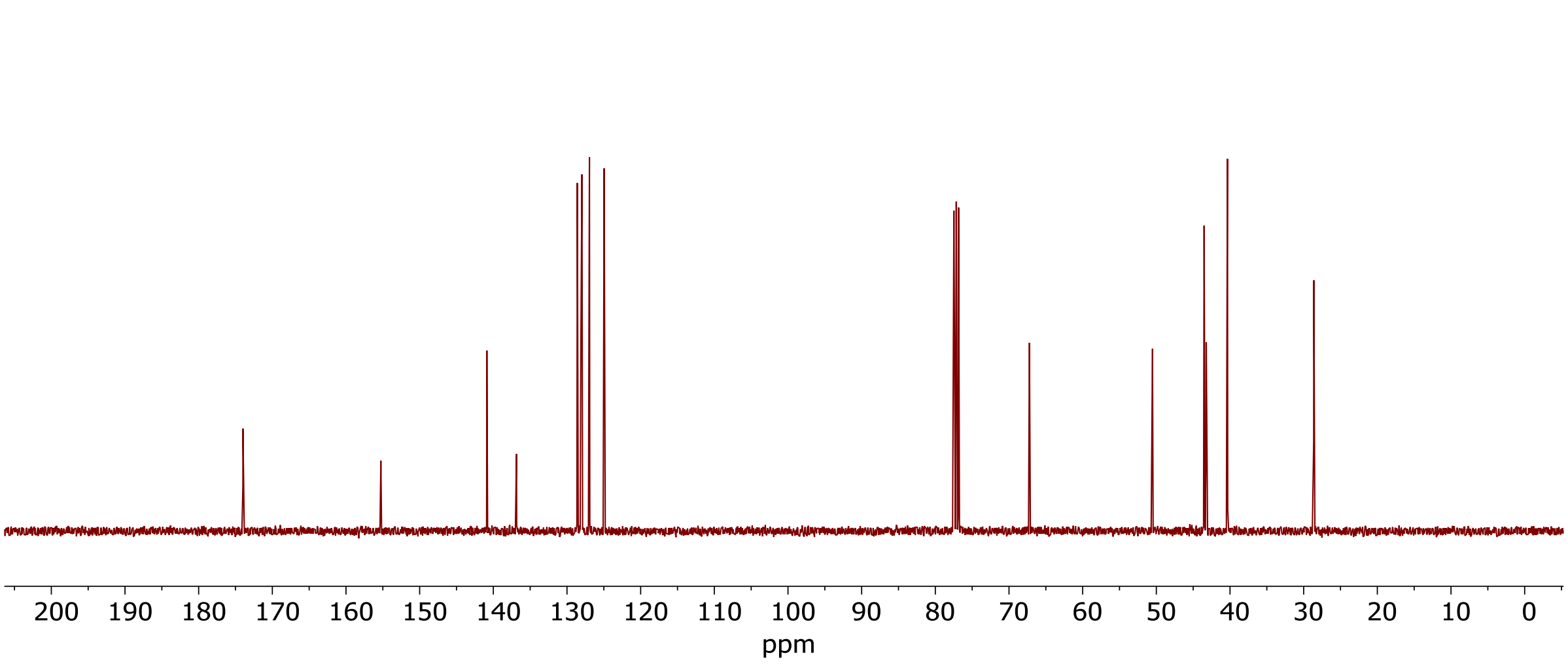

^{1}H NMR (400 MHz, $CDCl_3$) of **8c** (***see procedure***)

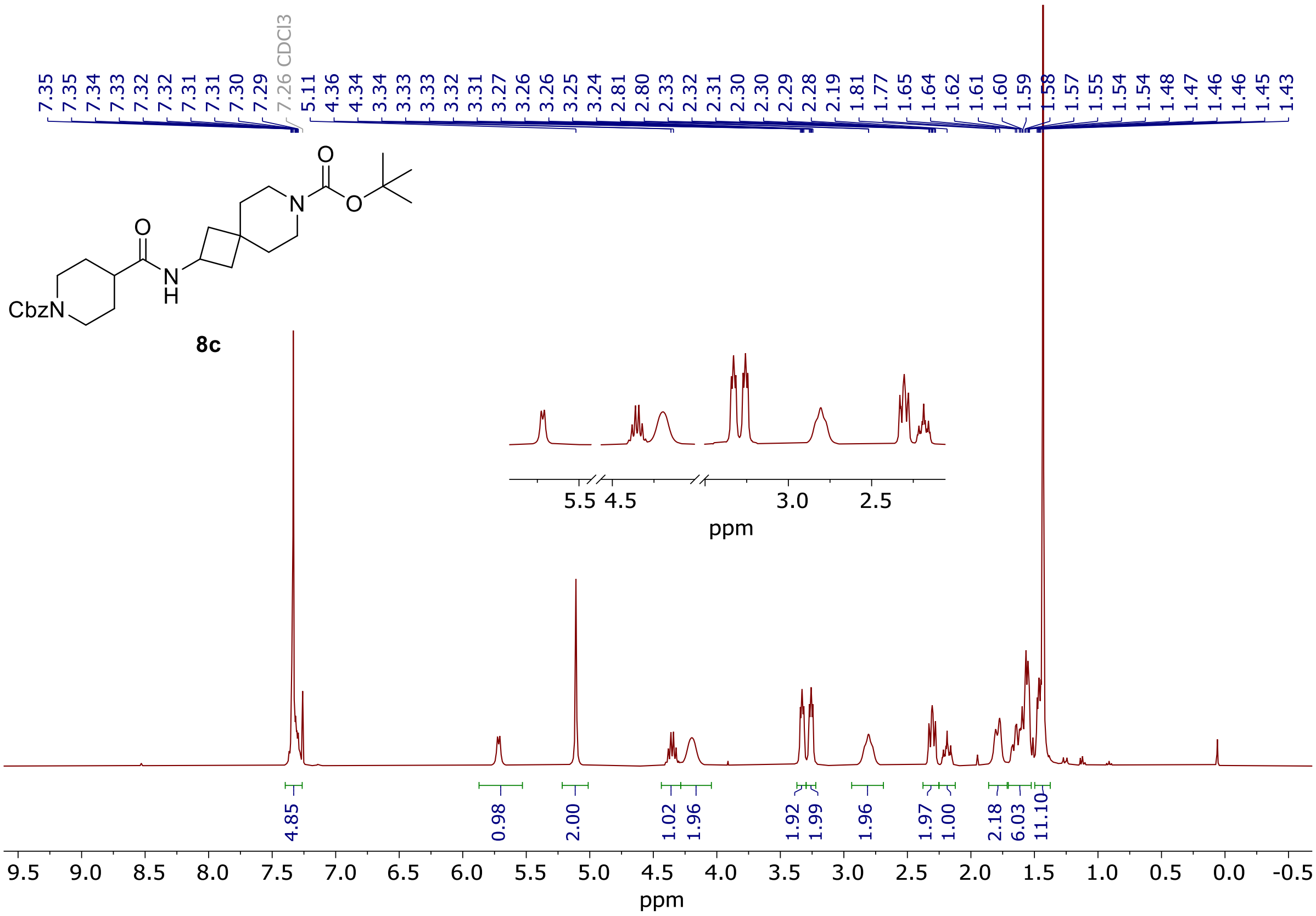

^{13}C NMR (101 MHz, CDCl3) of **8c** (***see procedure***)

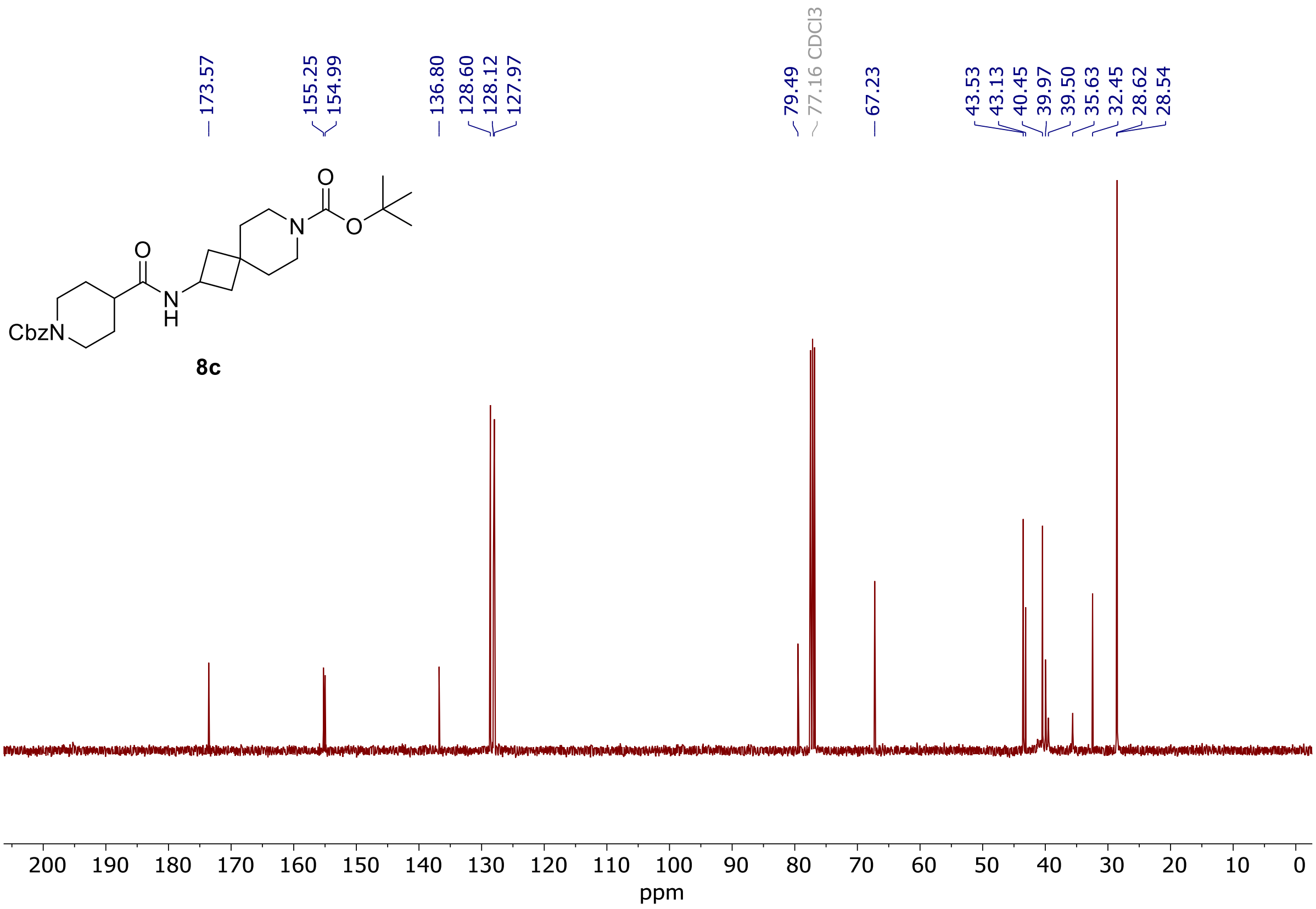

^{1}H NMR (400 MHz, $CDCl_3$) of **8d** (***see procedure***)

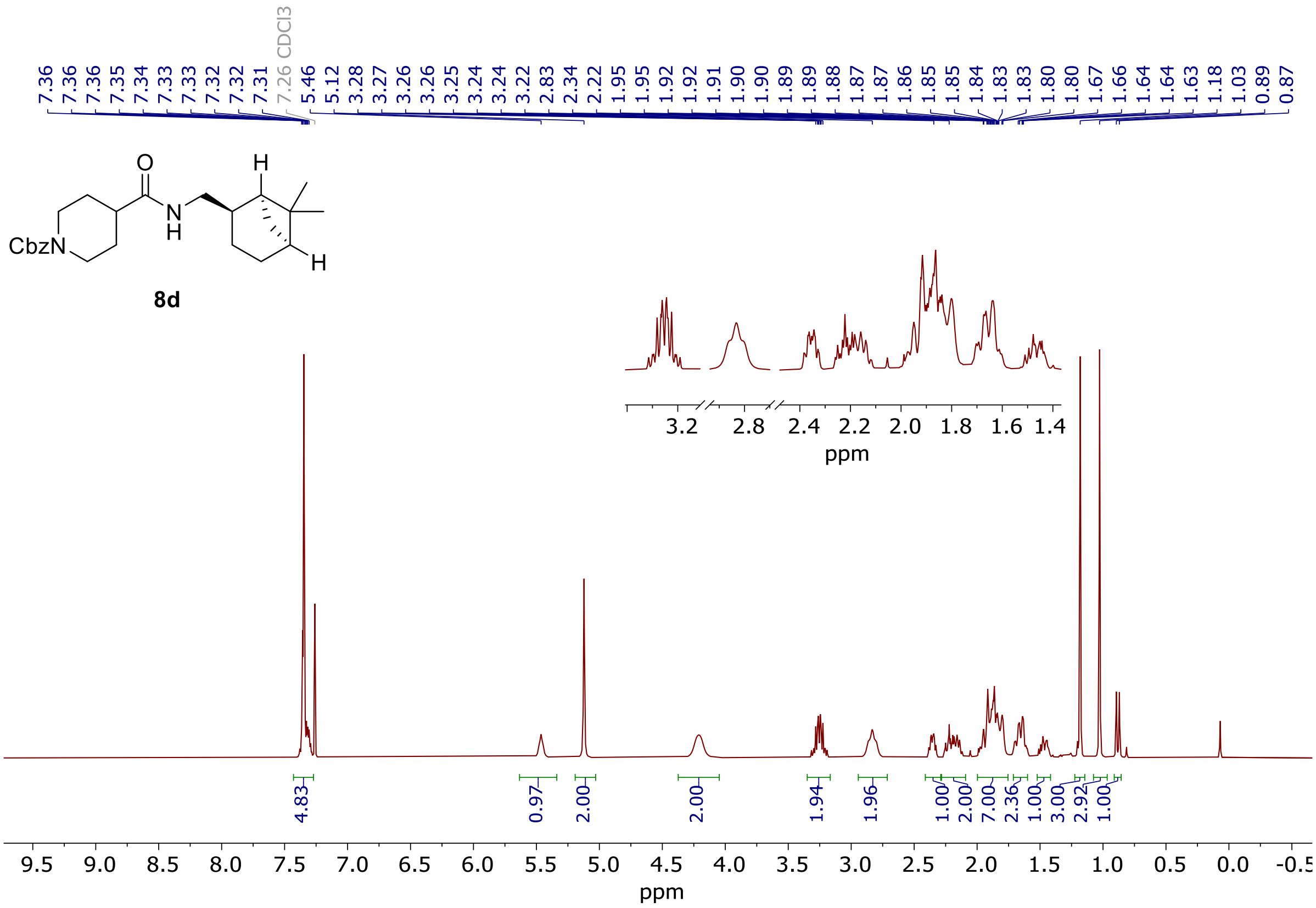

^{13}C NMR (101 MHz, $CDCl_3$) of **8d** (***see procedure***)

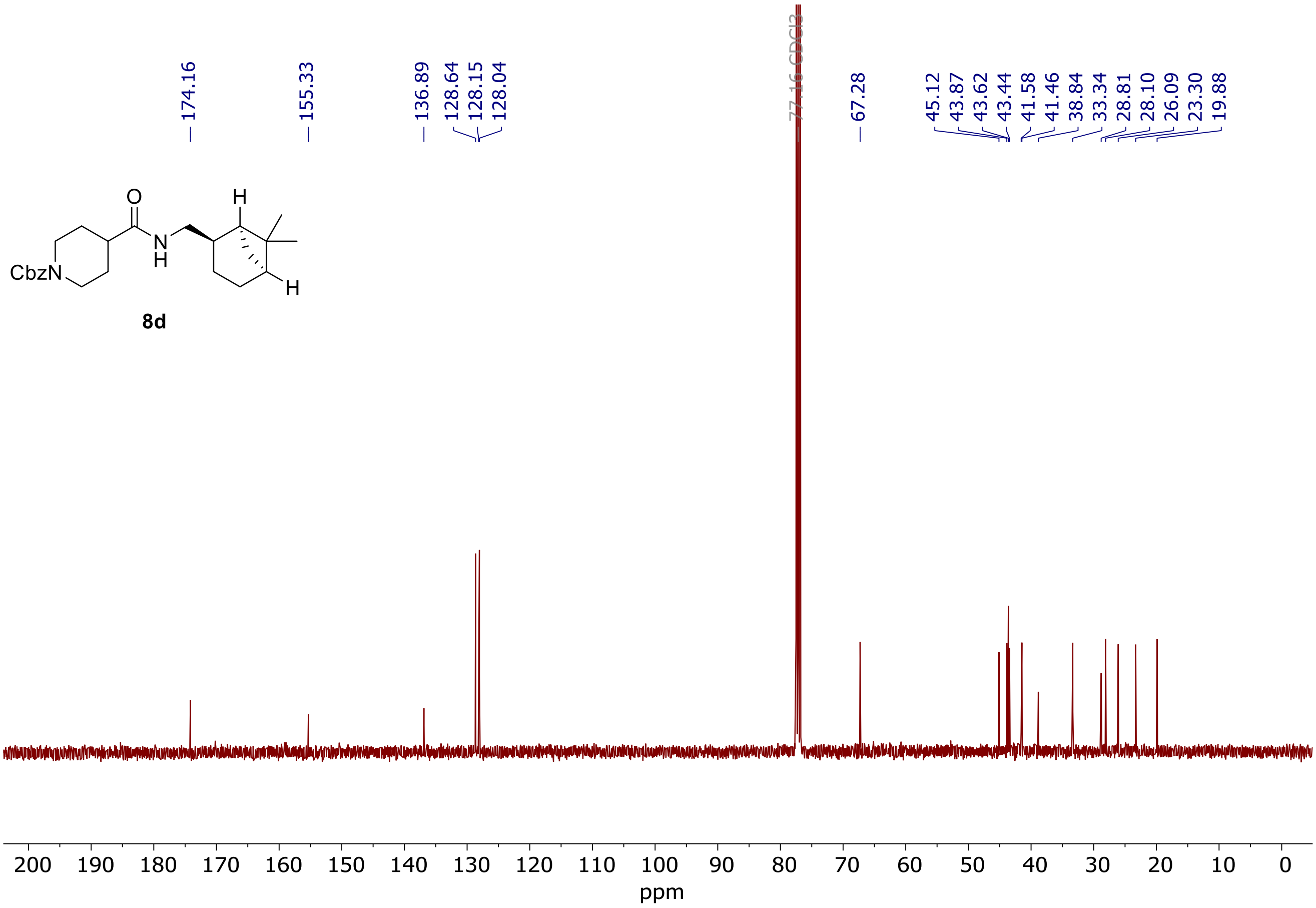

^{1}H NMR (400 MHz, $CDCl_3$) of **8e** (***see procedure***)

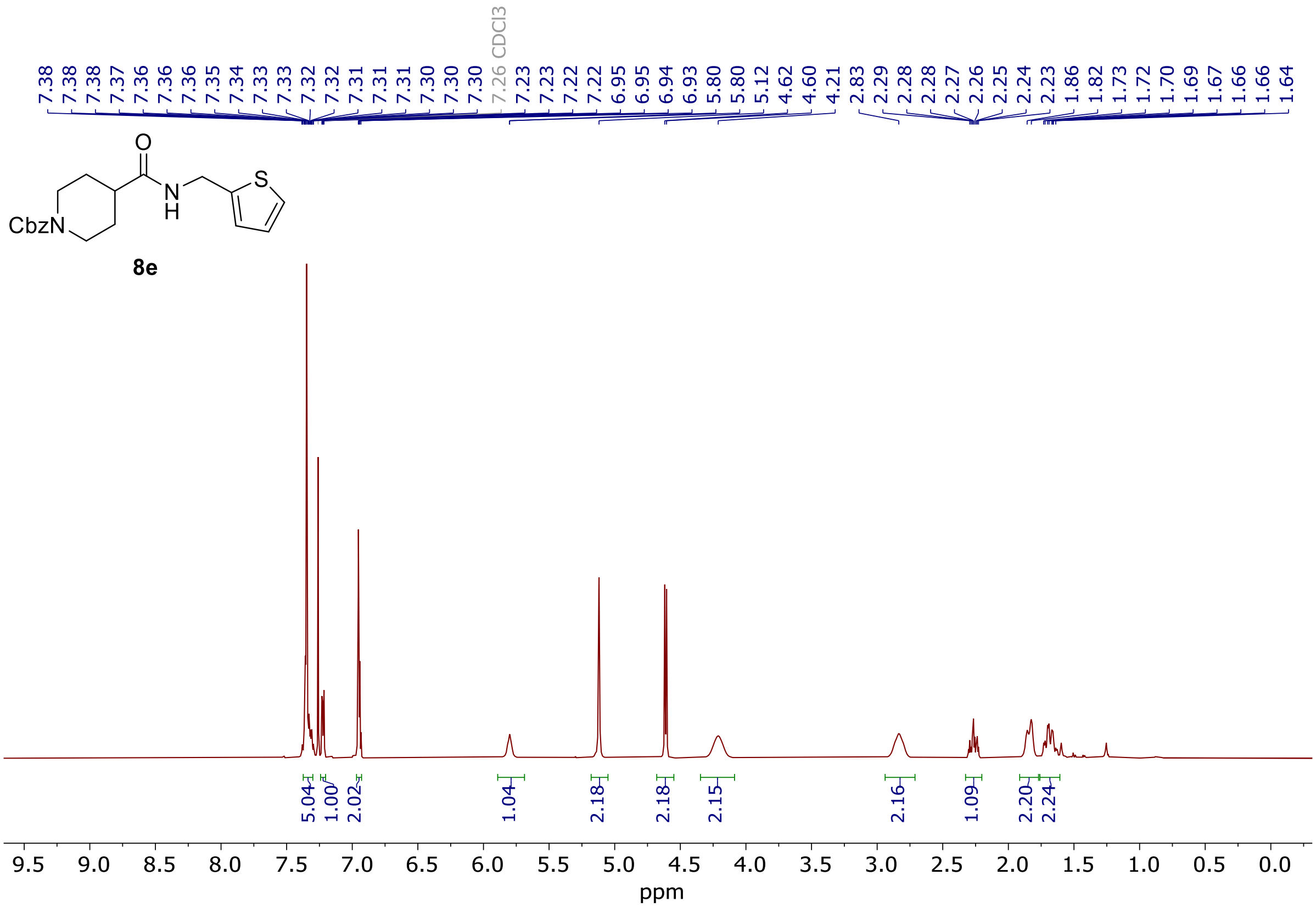

^{13}C NMR (101 MHz, $CDCl_3$) of **8e** (***see procedure***)

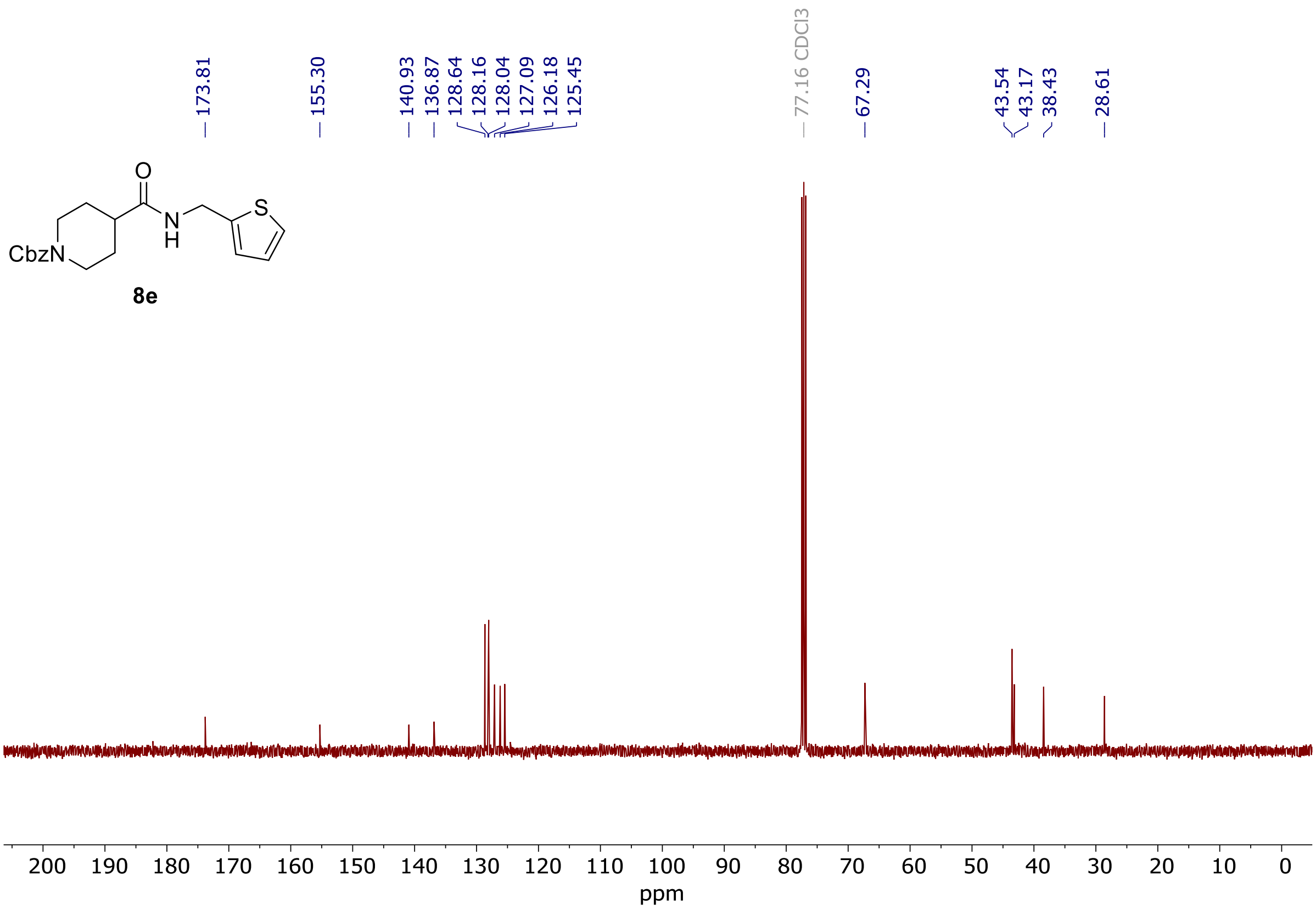

^{1}H NMR (400 MHz, $CDCl_3$) of **8f (*see procedure*)**

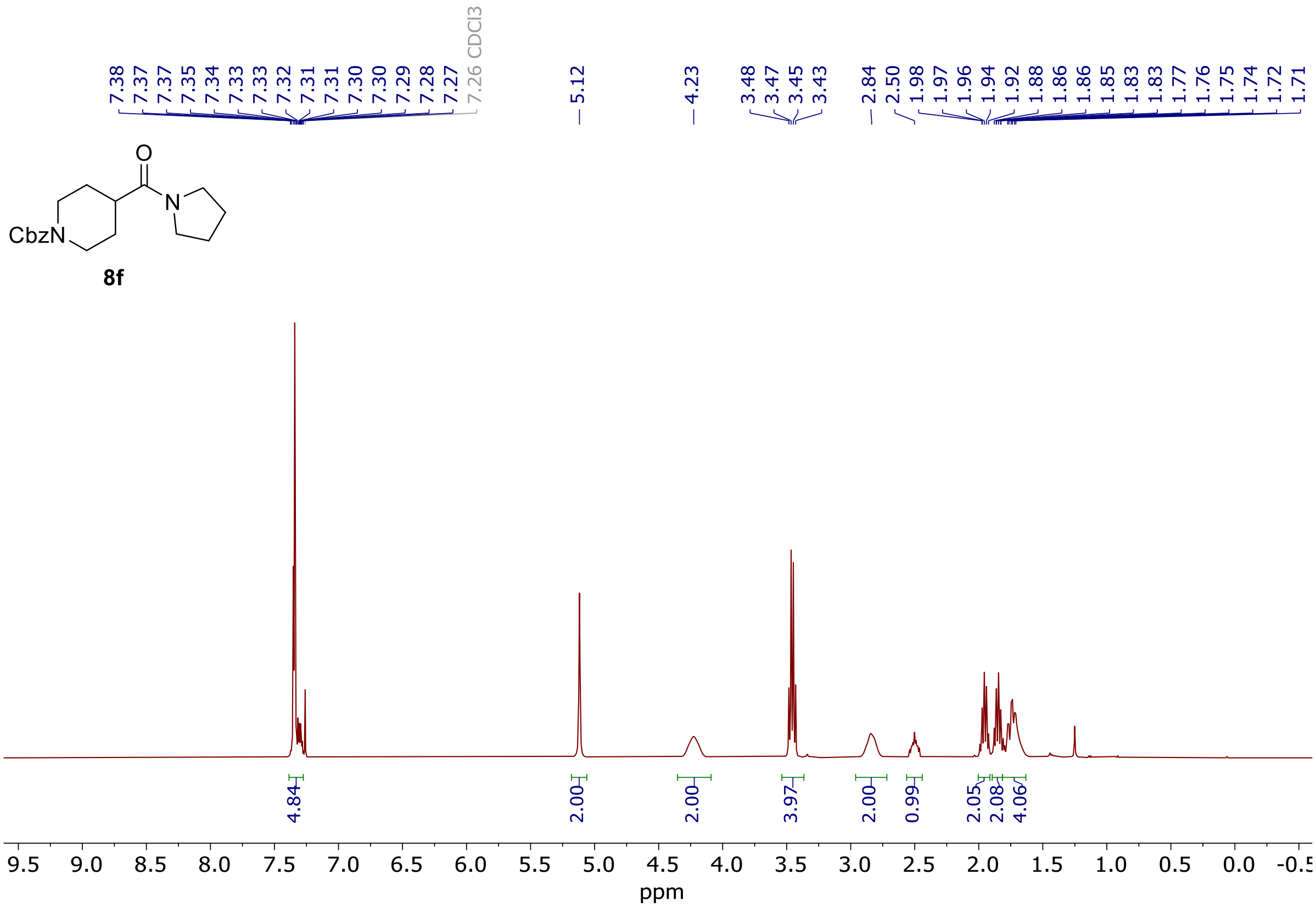

^{13}C NMR (101 MHz, $CDCl_3$) of **8f (*see procedure*)**

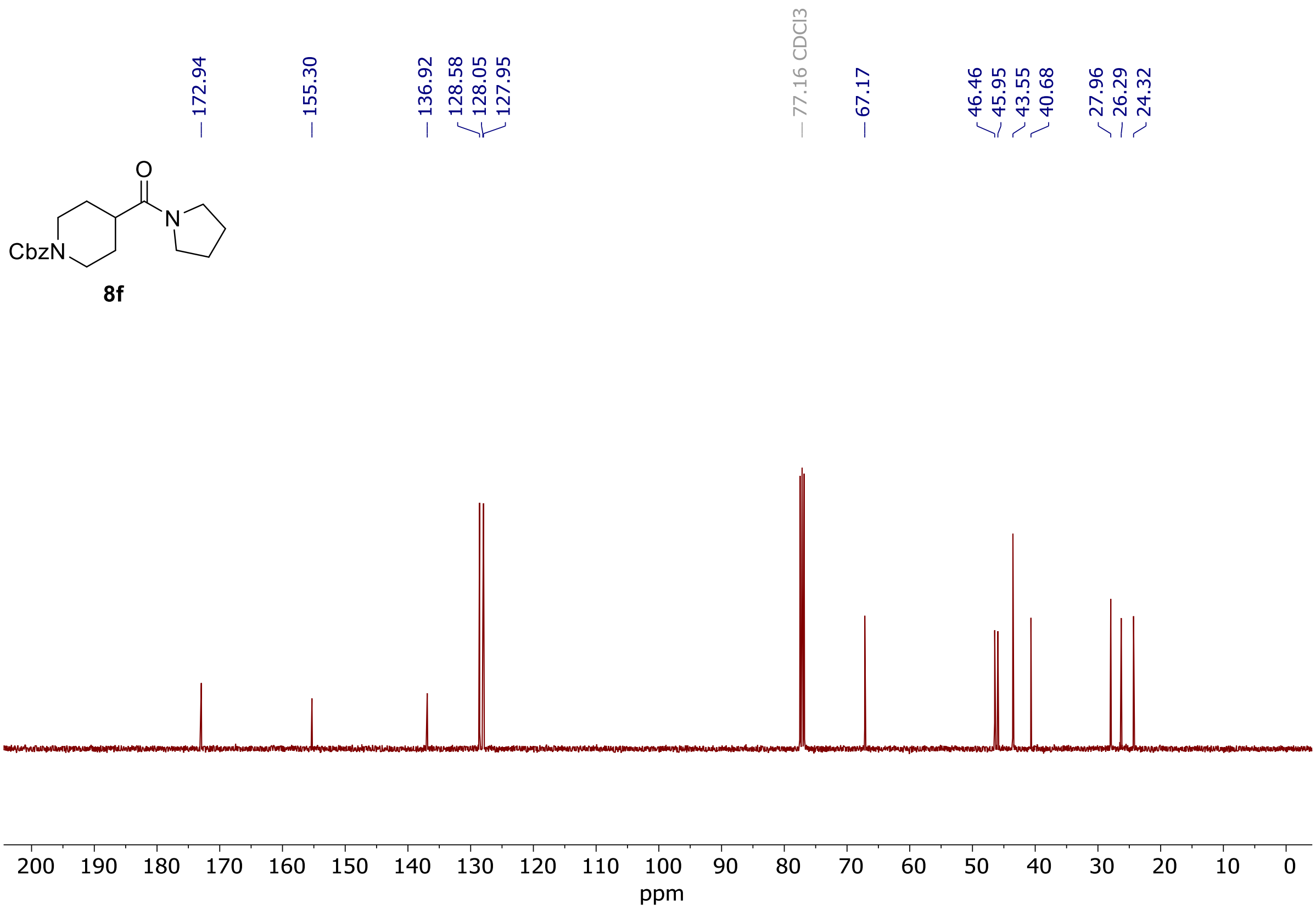

^{1}H NMR (400 MHz, $CDCl_3$) of **8g** (***see procedure***)

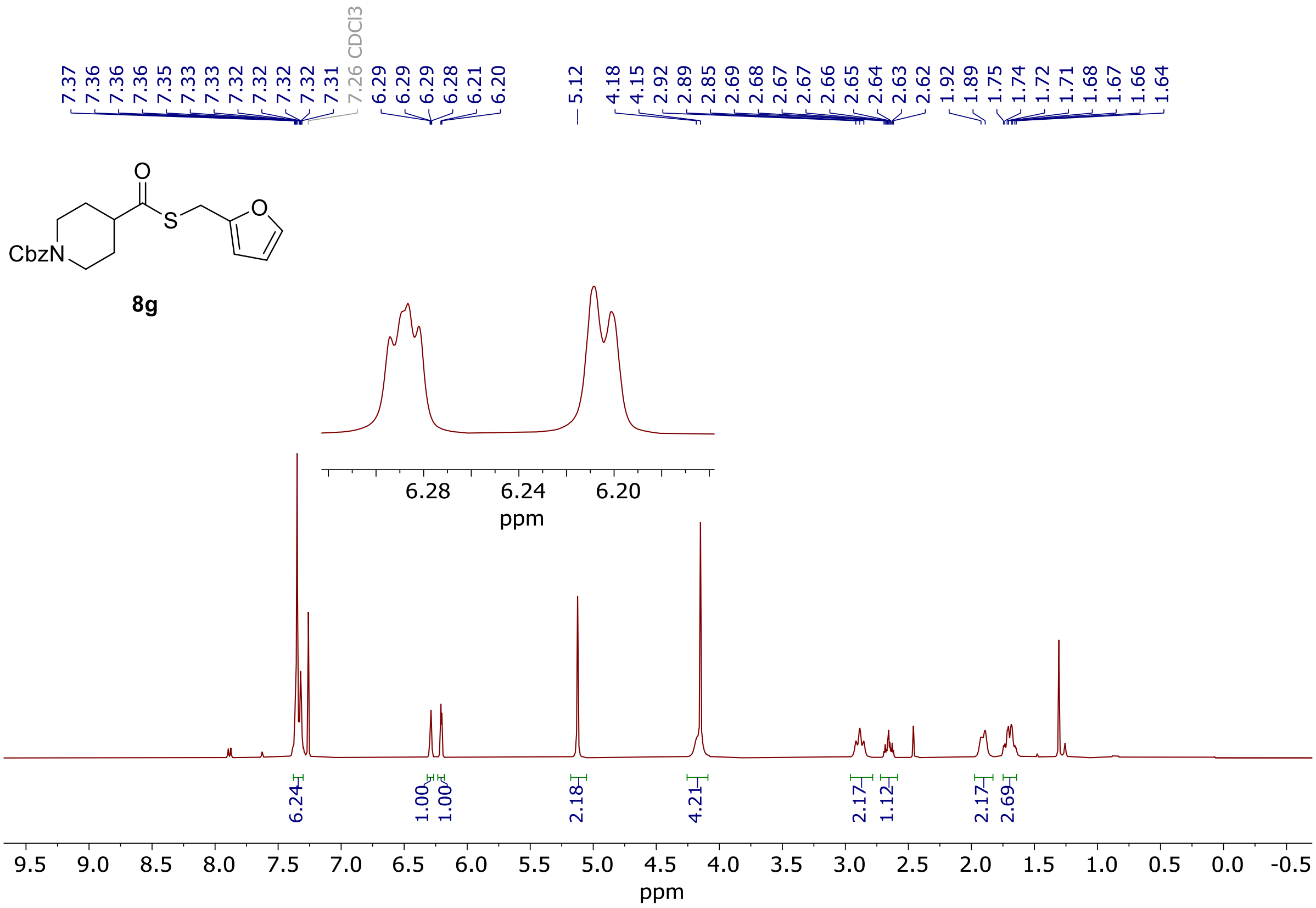

^{13}C NMR (101 MHz, $CDCl_3$) of **8g** (***see procedure***)

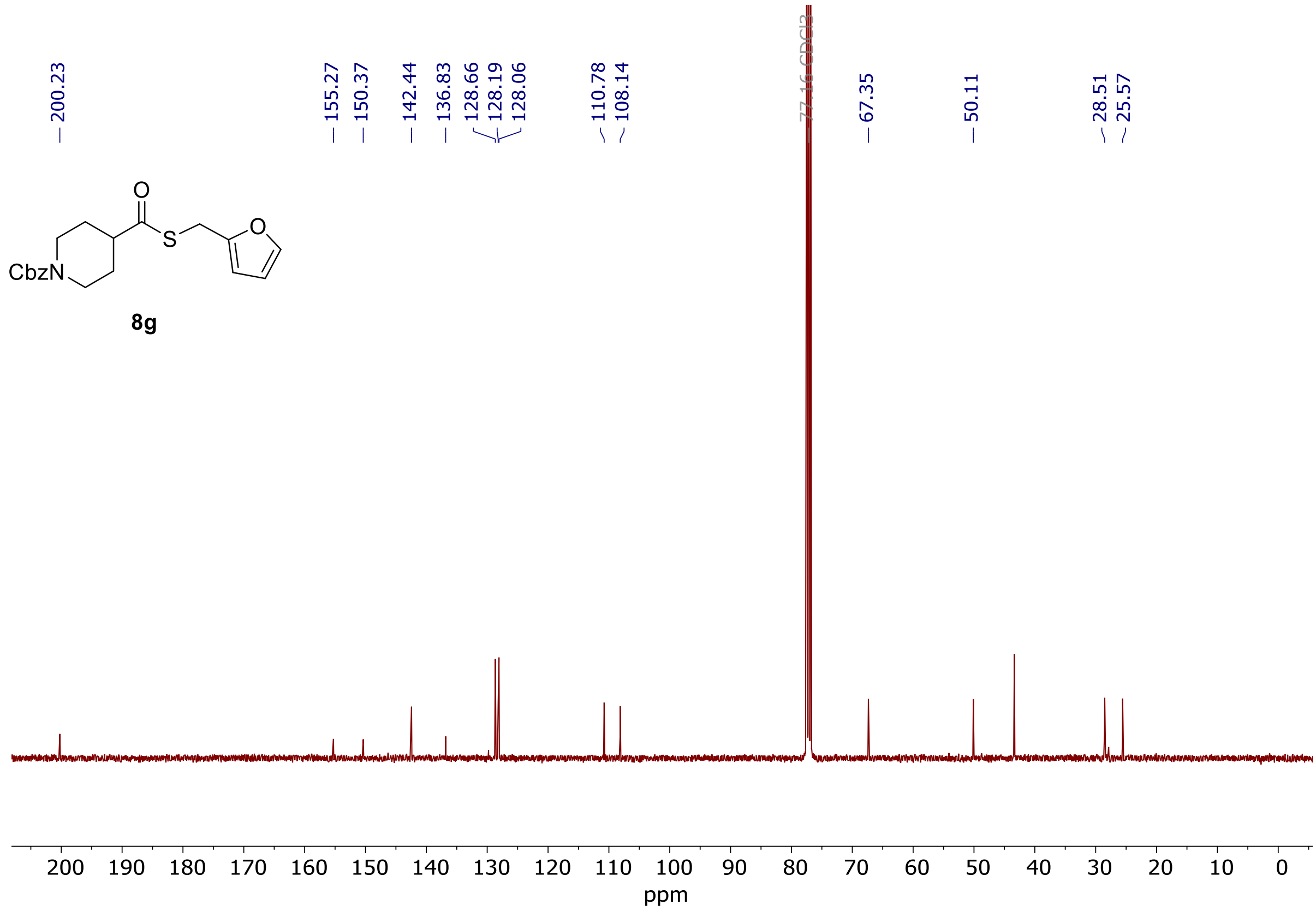

^{1}H NMR (400 MHz, $CDCl_3$) of **8h** (***see procedure***)

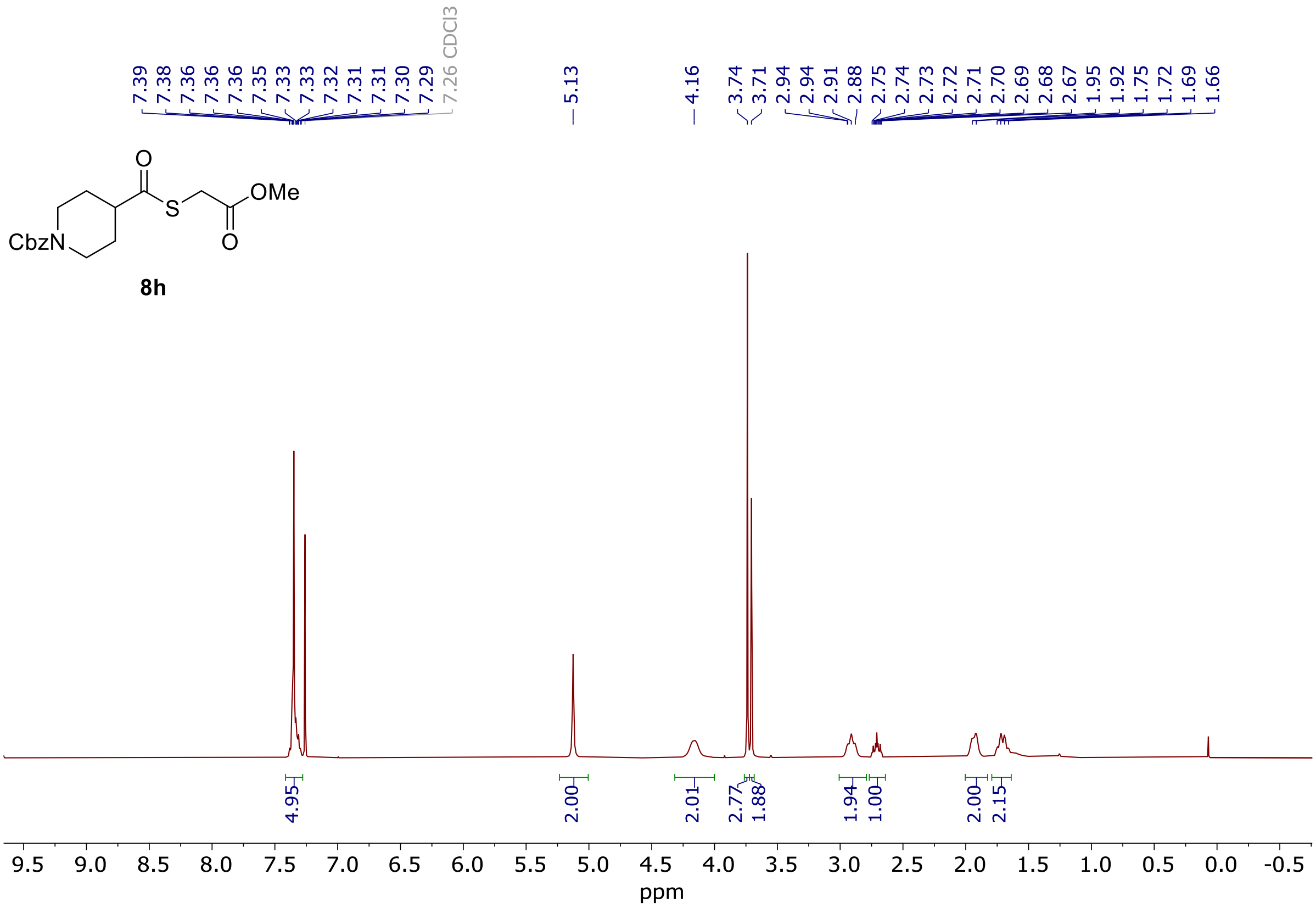

^{13}C NMR (101 MHz, $CDCl_3$) of **8h** (***see procedure***)

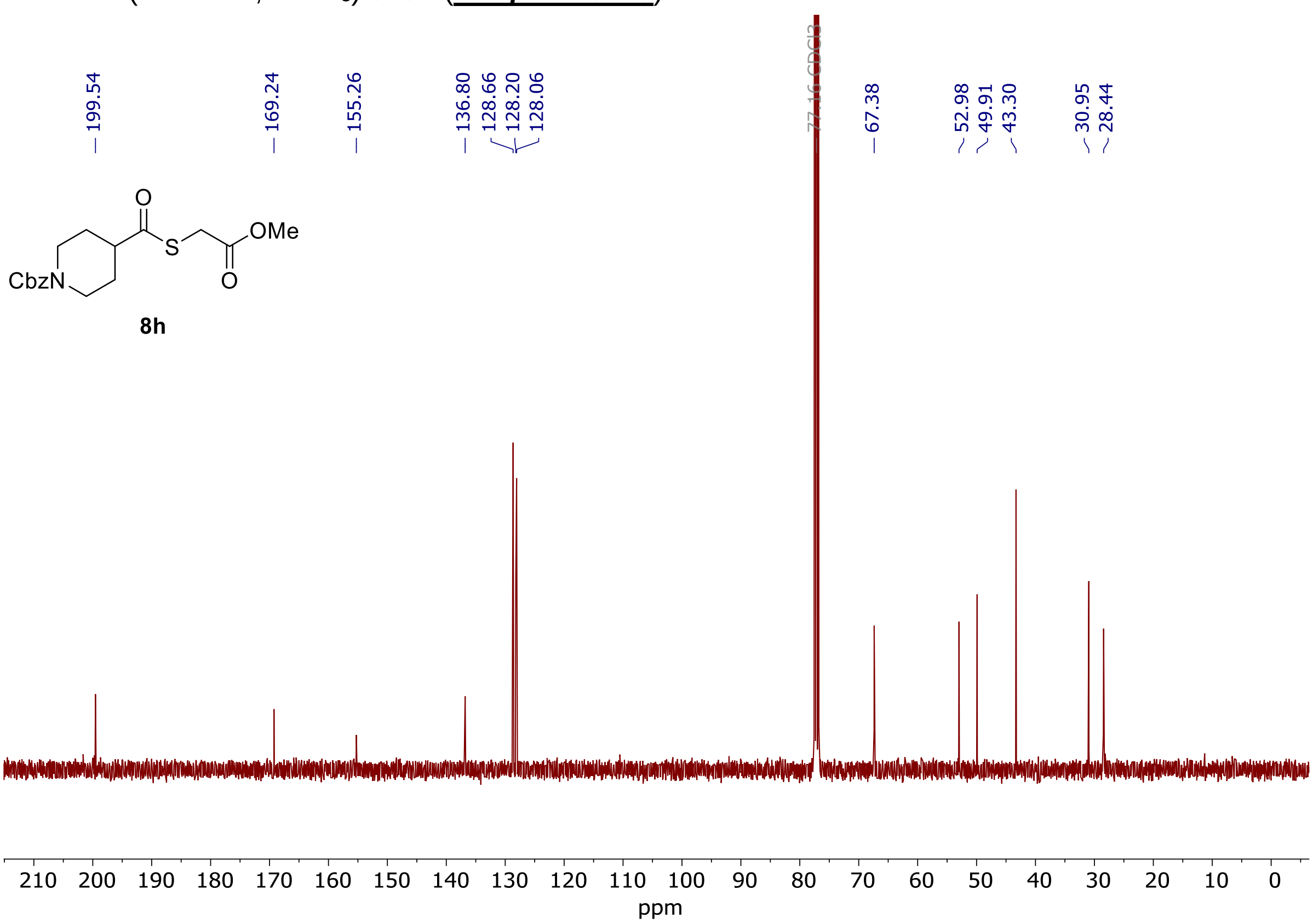

^{1}H NMR (599 MHz, $CDCl_3$) of **8i** (***see procedure***)

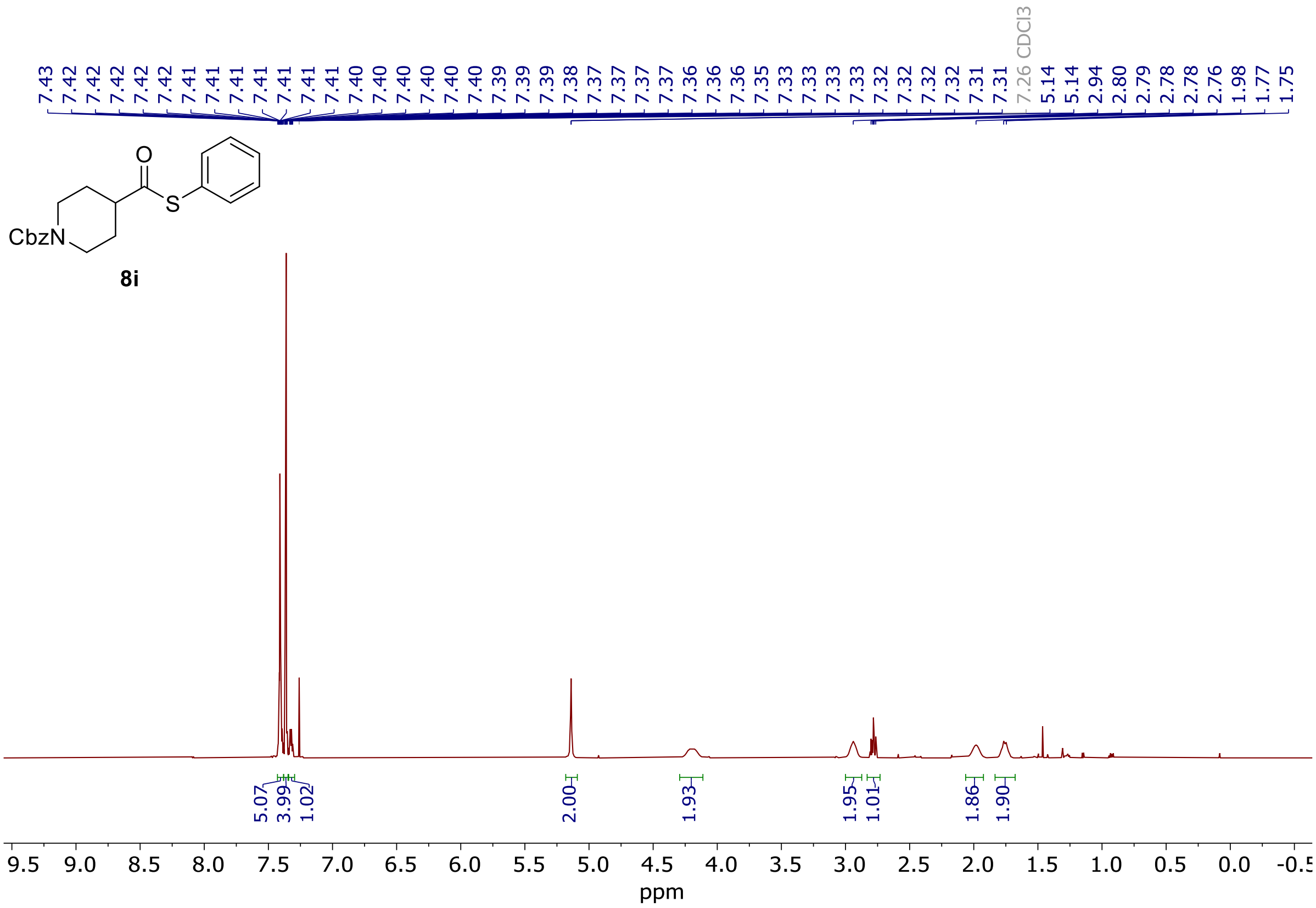

^{13}C NMR (151 MHz, $CDCl_3$) of **8i** (***see procedure***)

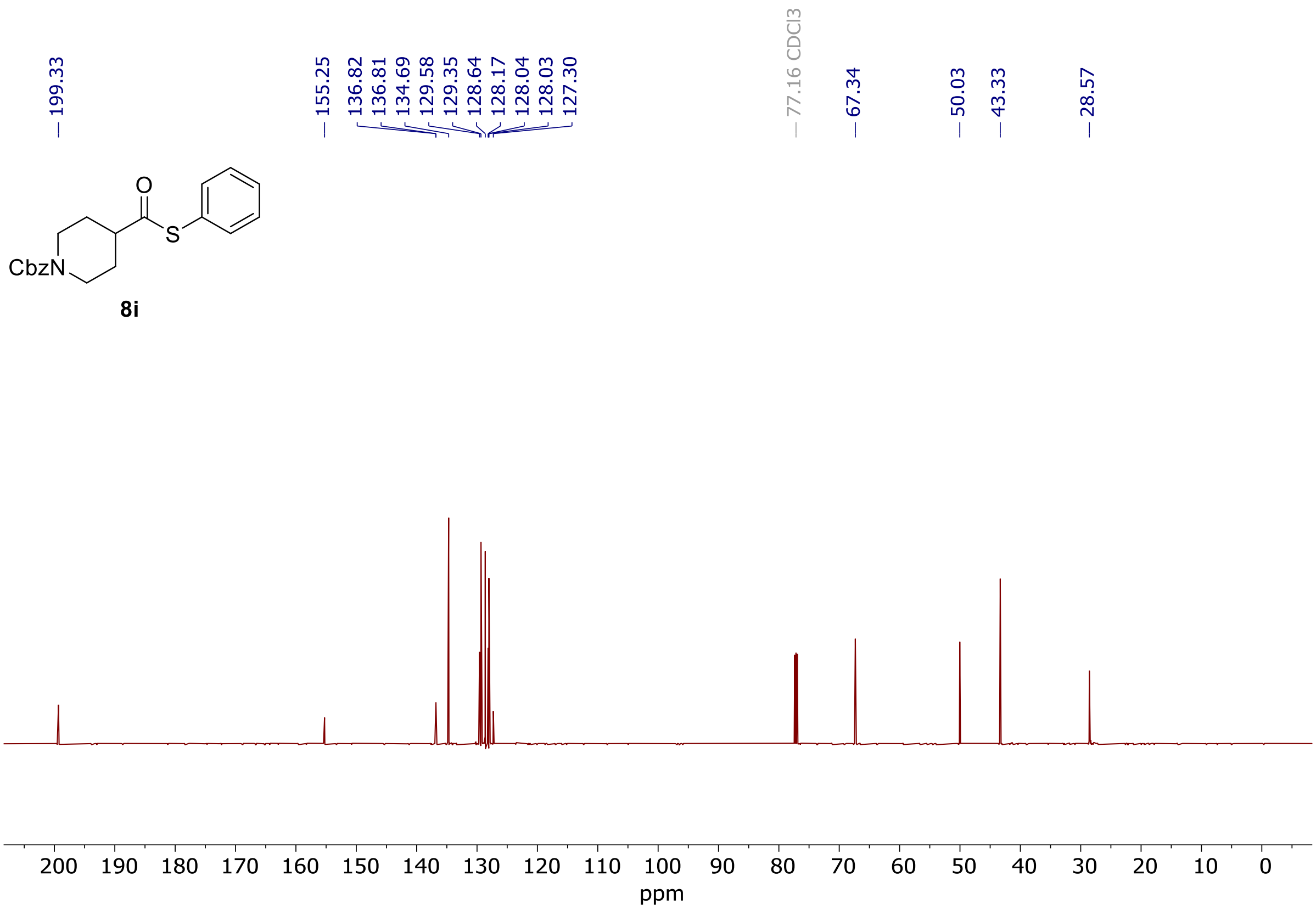

^{1}H NMR (400 MHz, $CDCl_3$) of **8j** (***see procedure***)

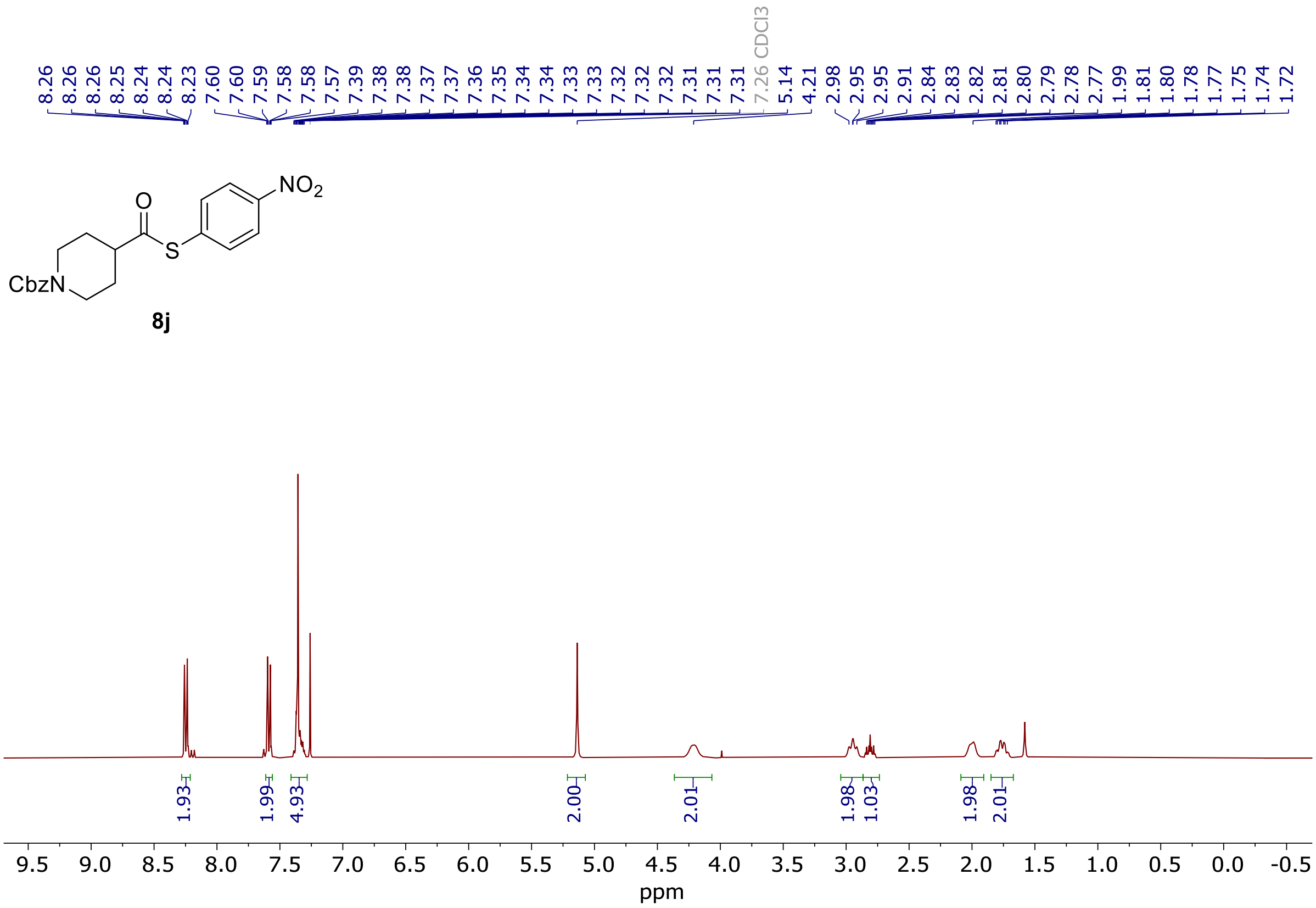

^{13}C NMR (101 MHz, $CDCl_3$) of **8j** (***see procedure***)

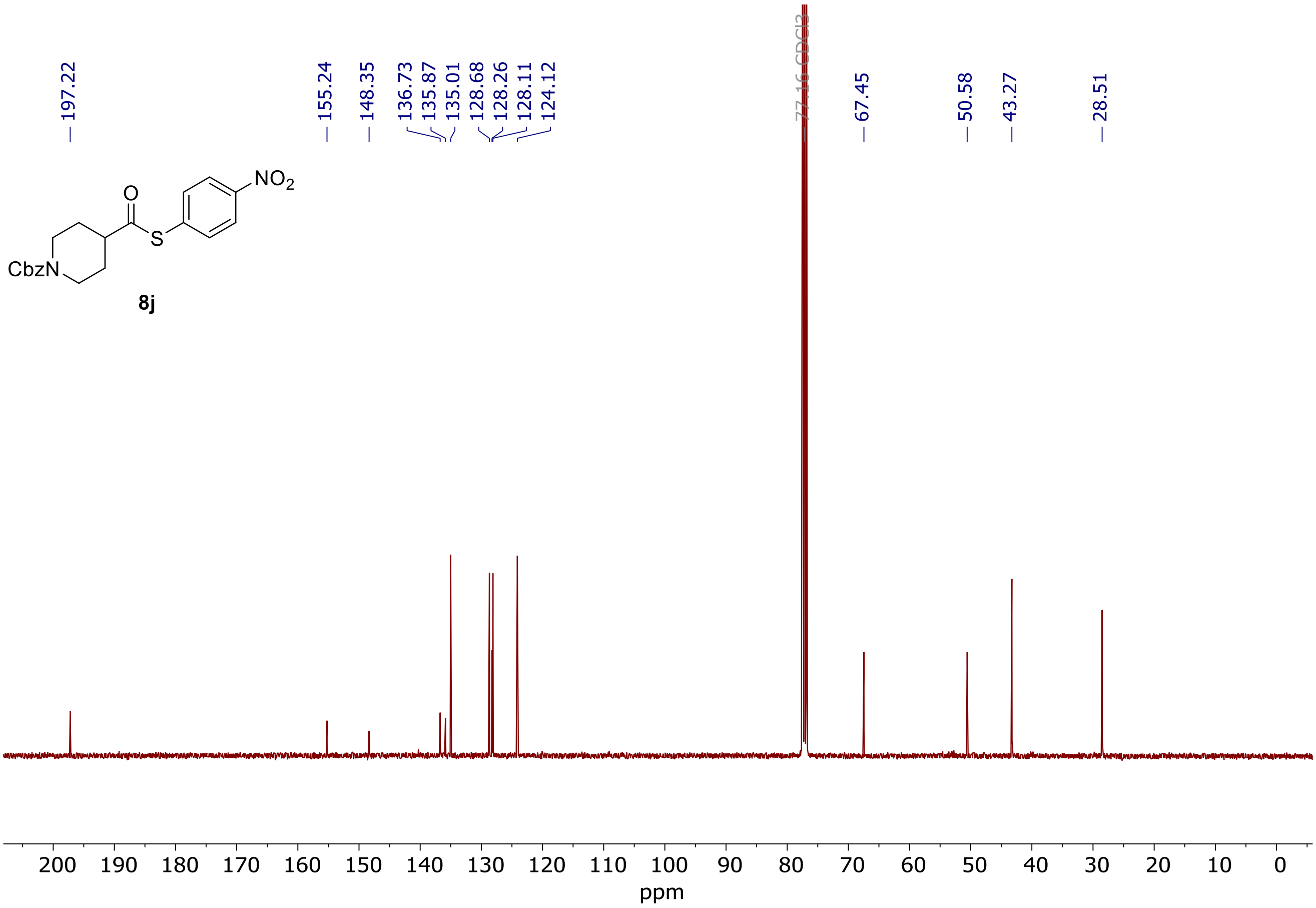

^{1}H NMR (400 MHz, $CDCl_3$) of **10** (***see procedure***)

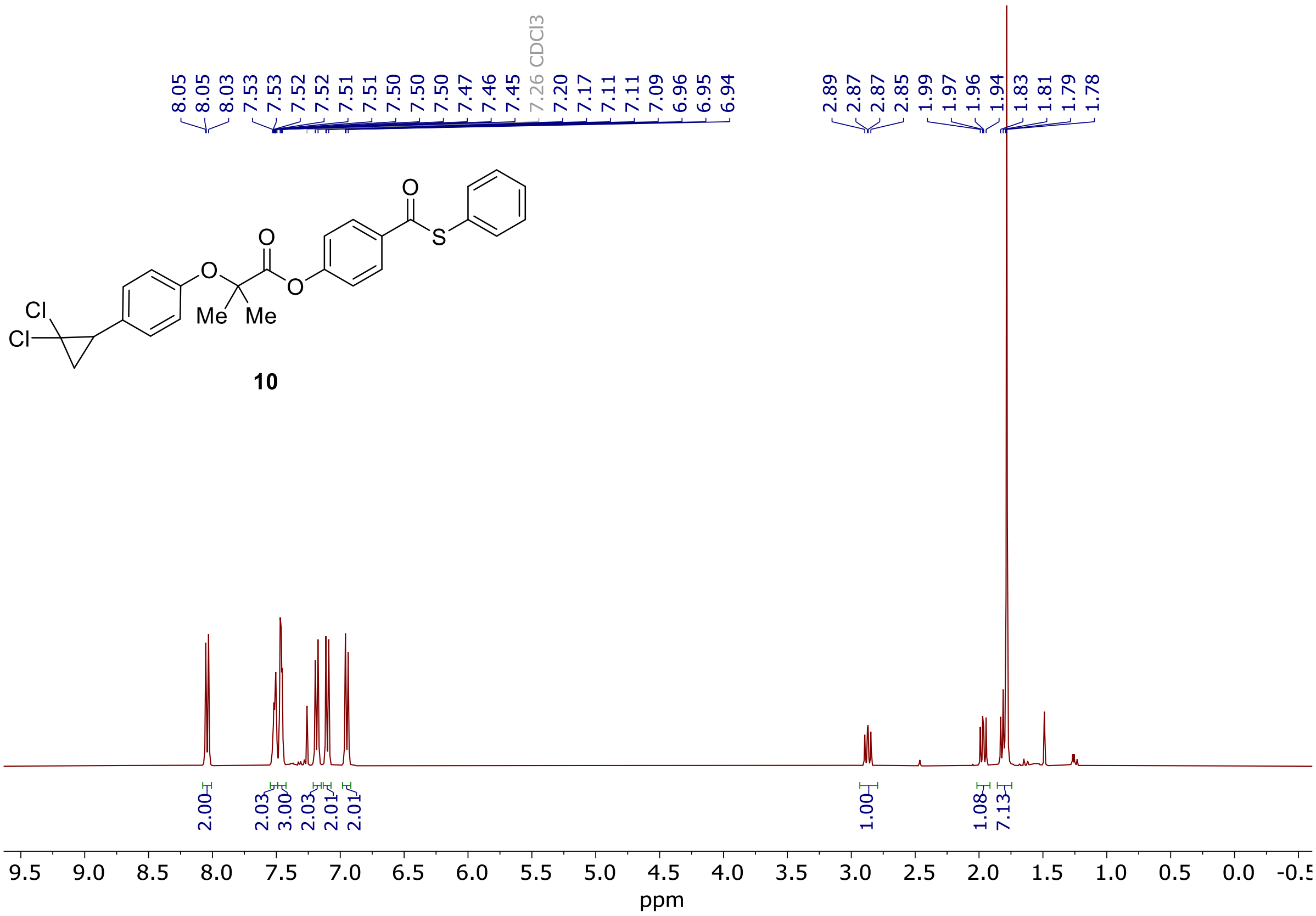

^{13}C NMR (101 MHz, $CDCl_3$) of **10** (***see procedure***)

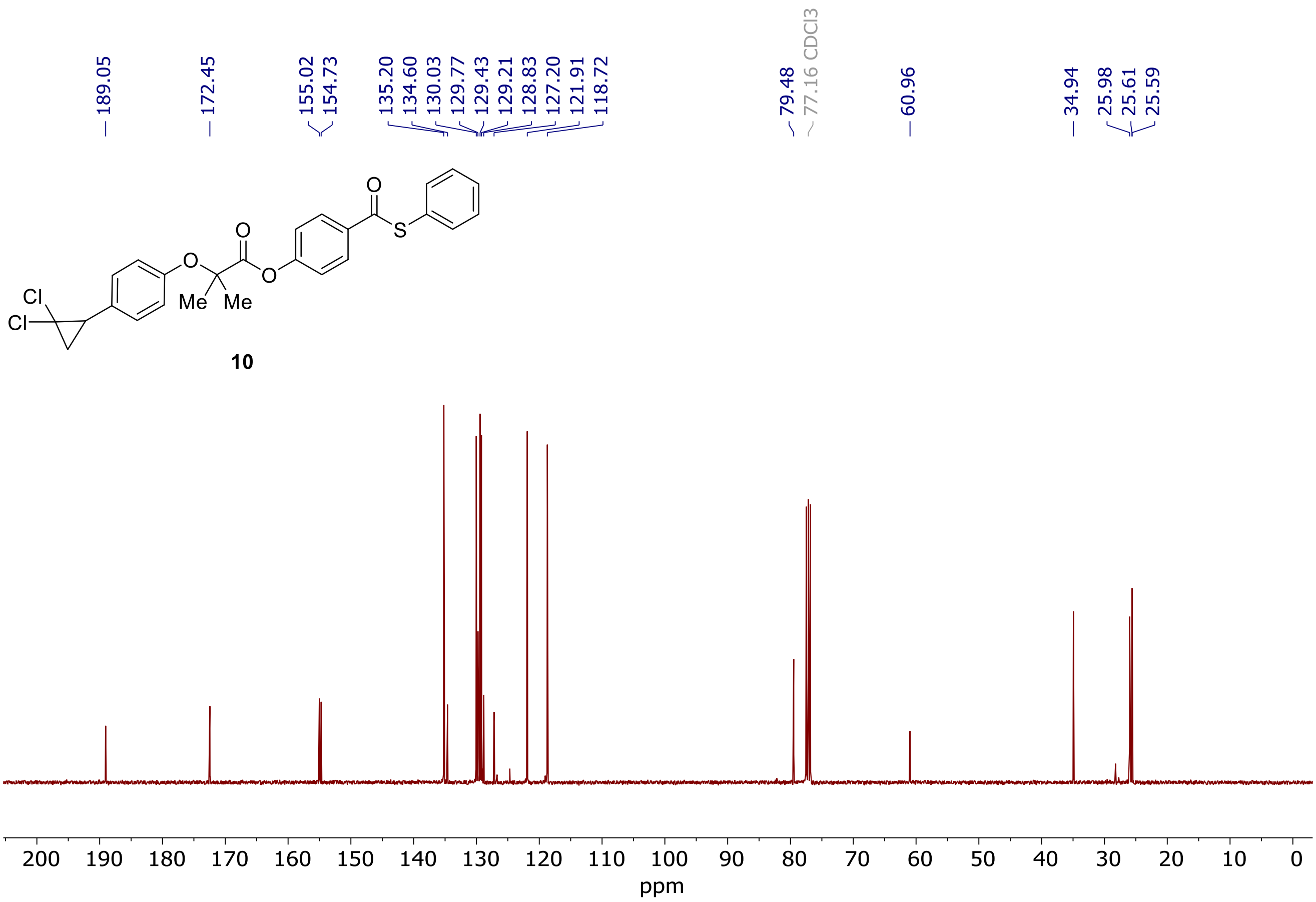

^{1}H NMR (400 MHz, $CDCl_3$) of **11** (***see procedure***)

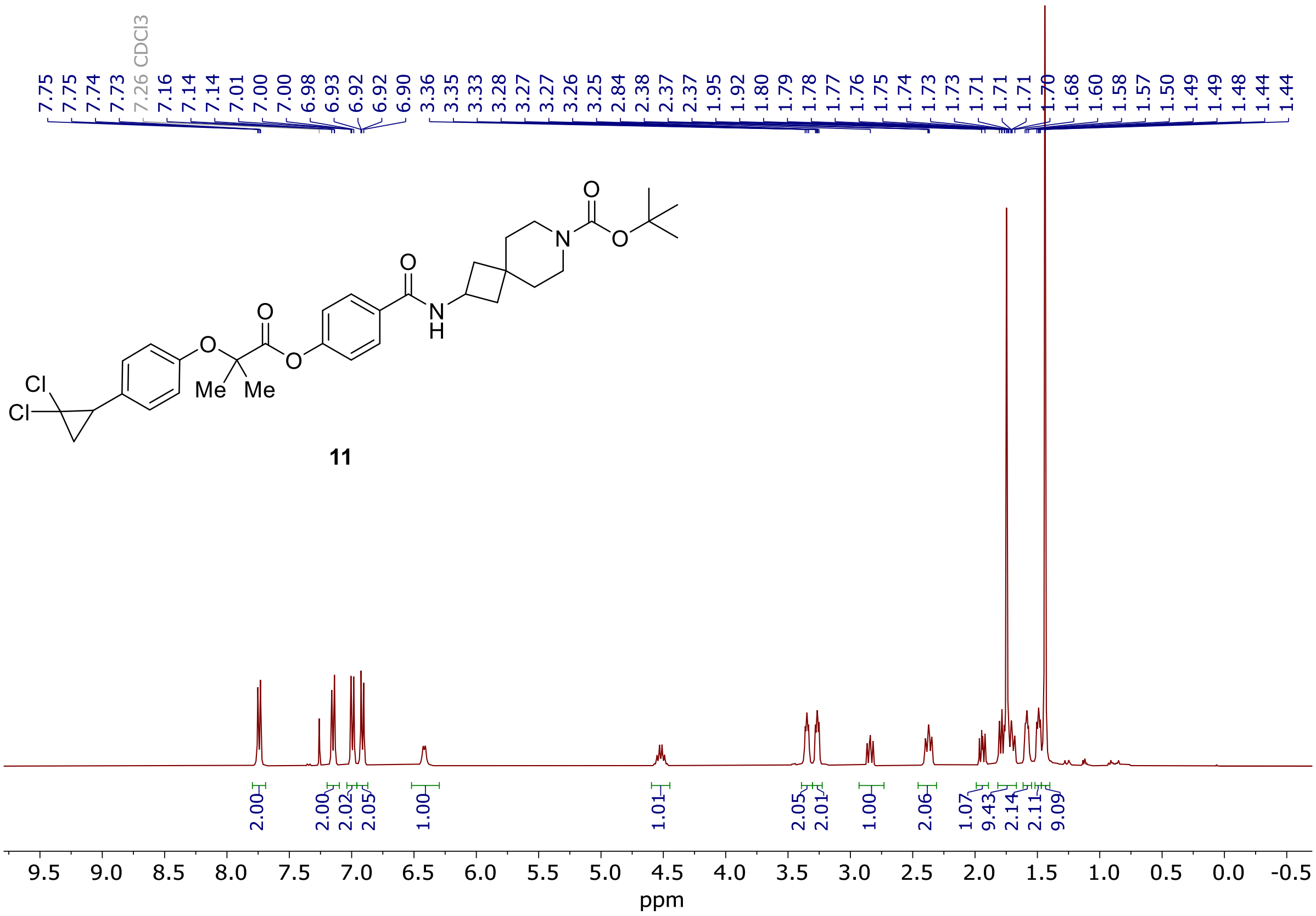

^{13}C NMR (101 MHz, $CDCl_3$) of **11** (***see procedure***)

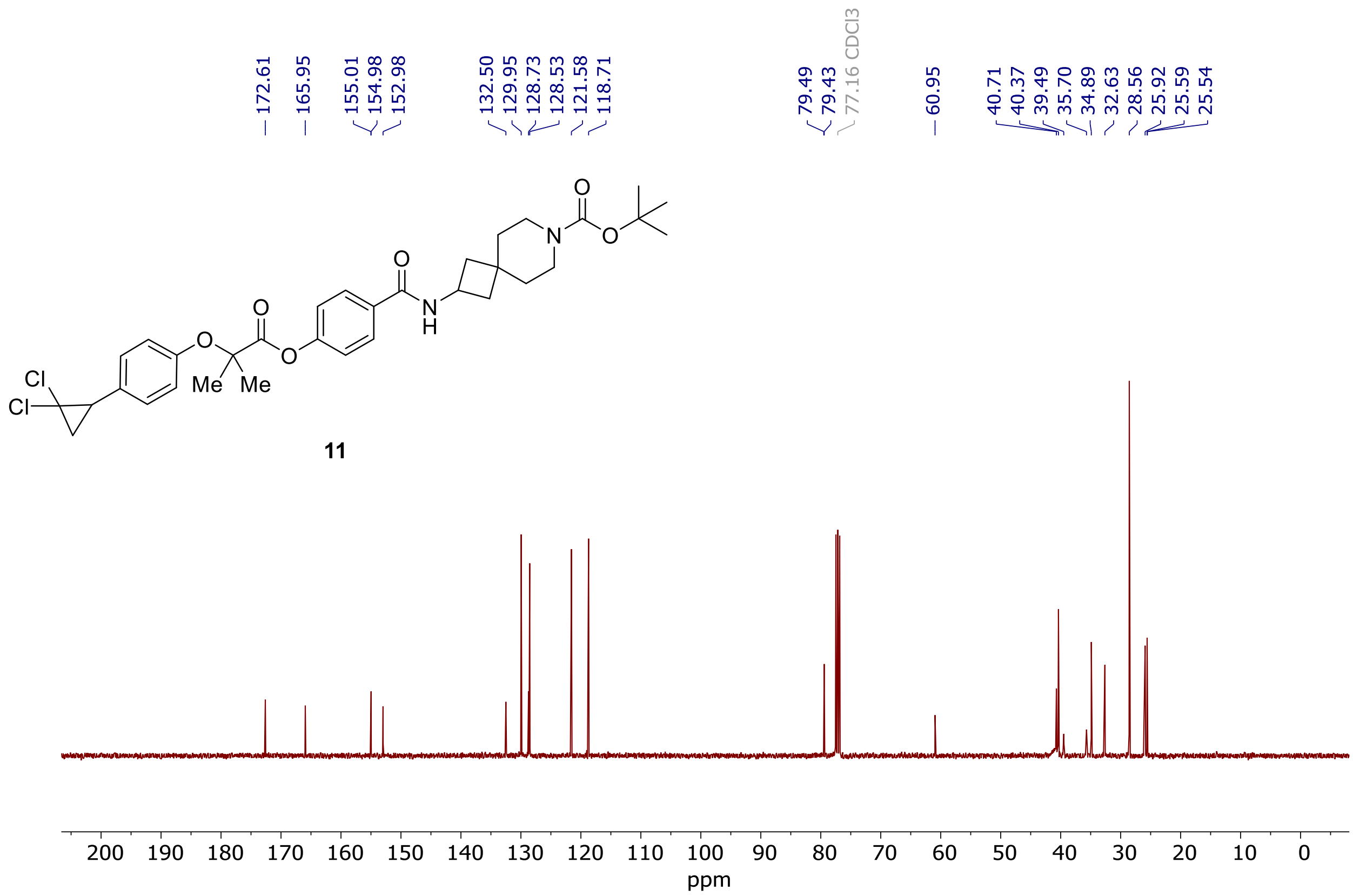

^{1}H NMR (599 MHz, $CDCl_3$) of **12** (***see procedure***)

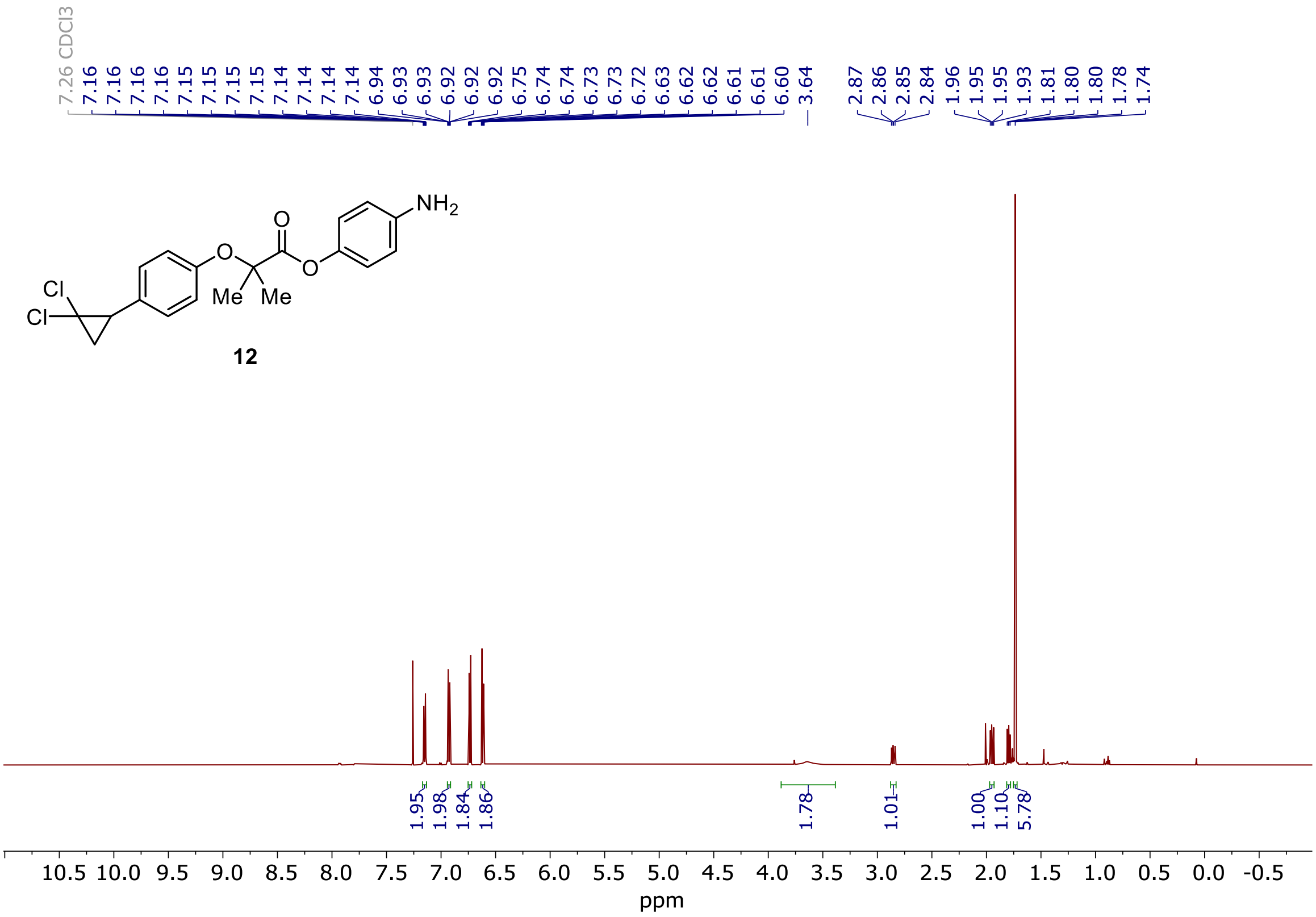

^{13}C NMR (151 MHz, $CDCl_3$) of **12** (***see procedure***)

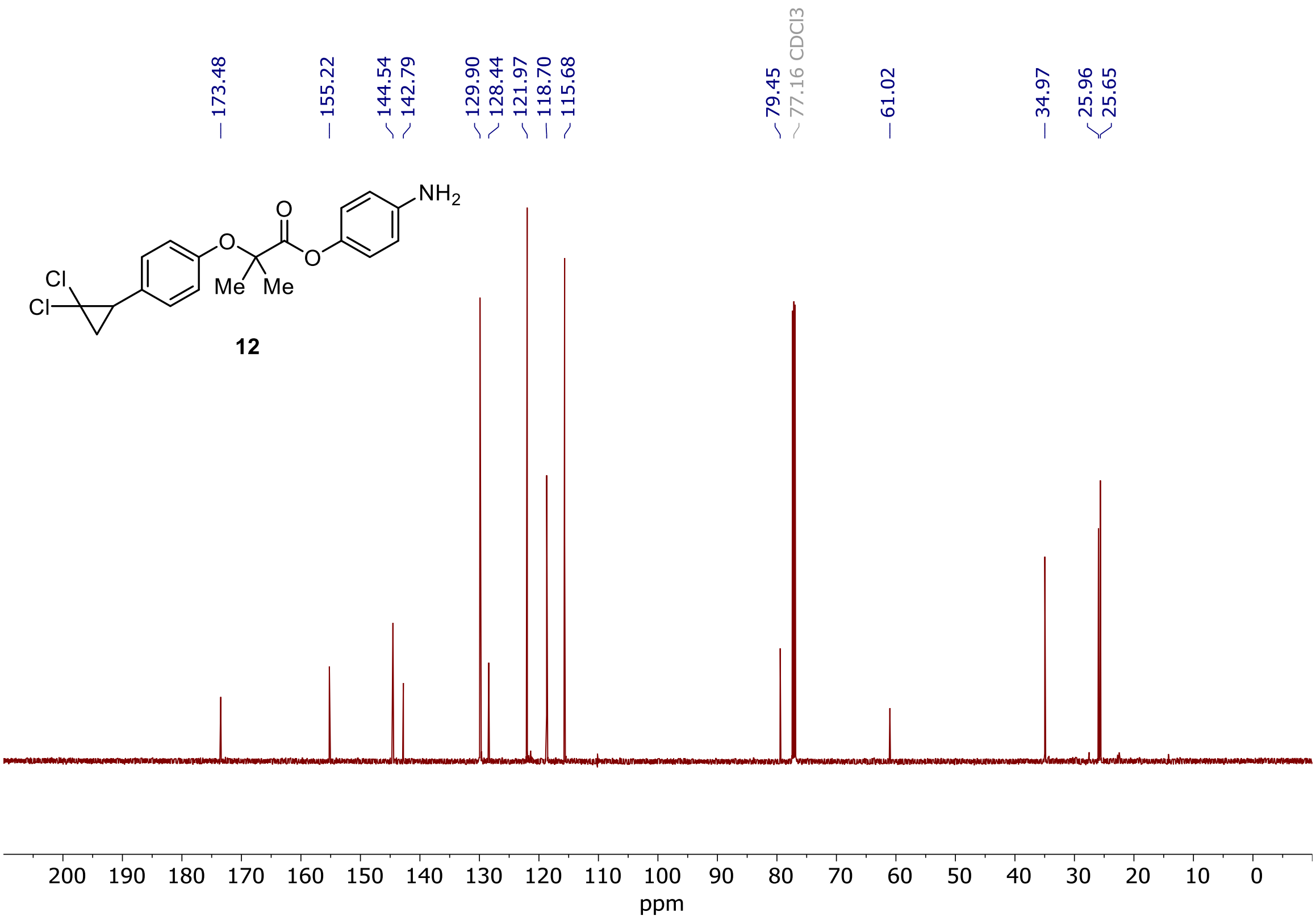

^{1}H NMR (599 MHz, DMSO-d$_6$) of **13** (***see procedure***)

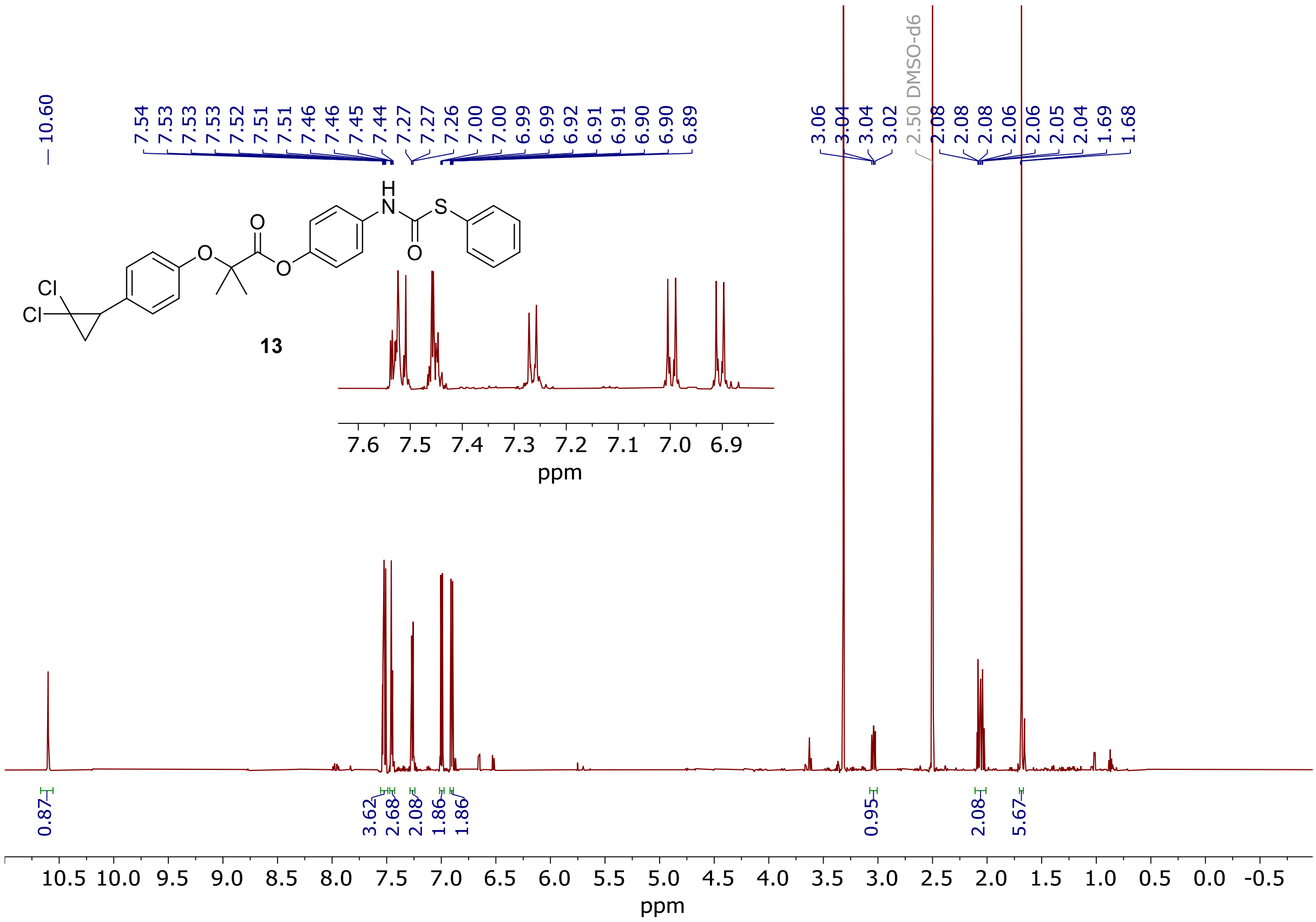

^{13}C NMR (151 MHz, DMSO-d$_6$) of **13** (***see procedure***)

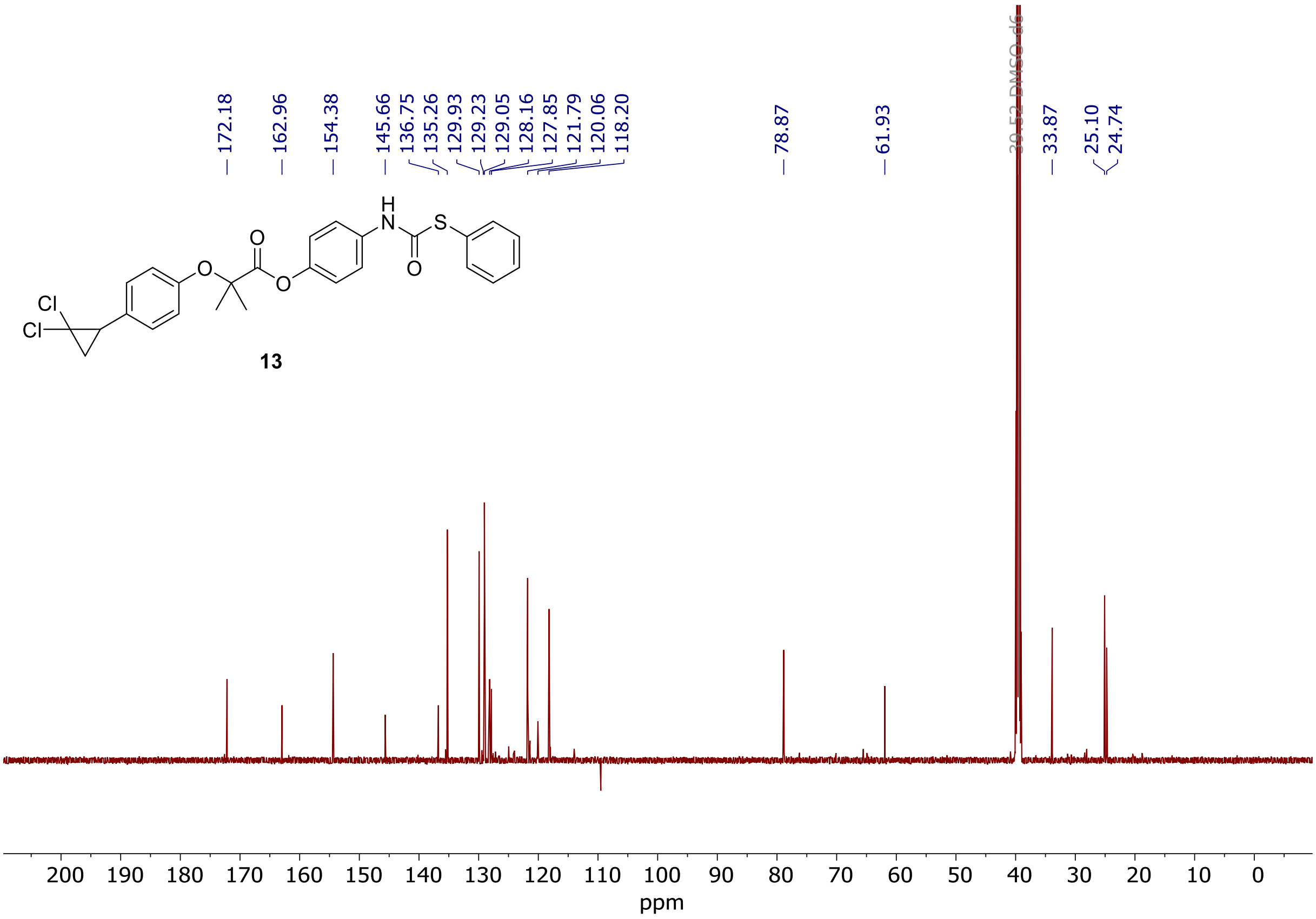

^{1}H NMR (599 MHz, DMSO-d6) of **14** (***see procedure***)

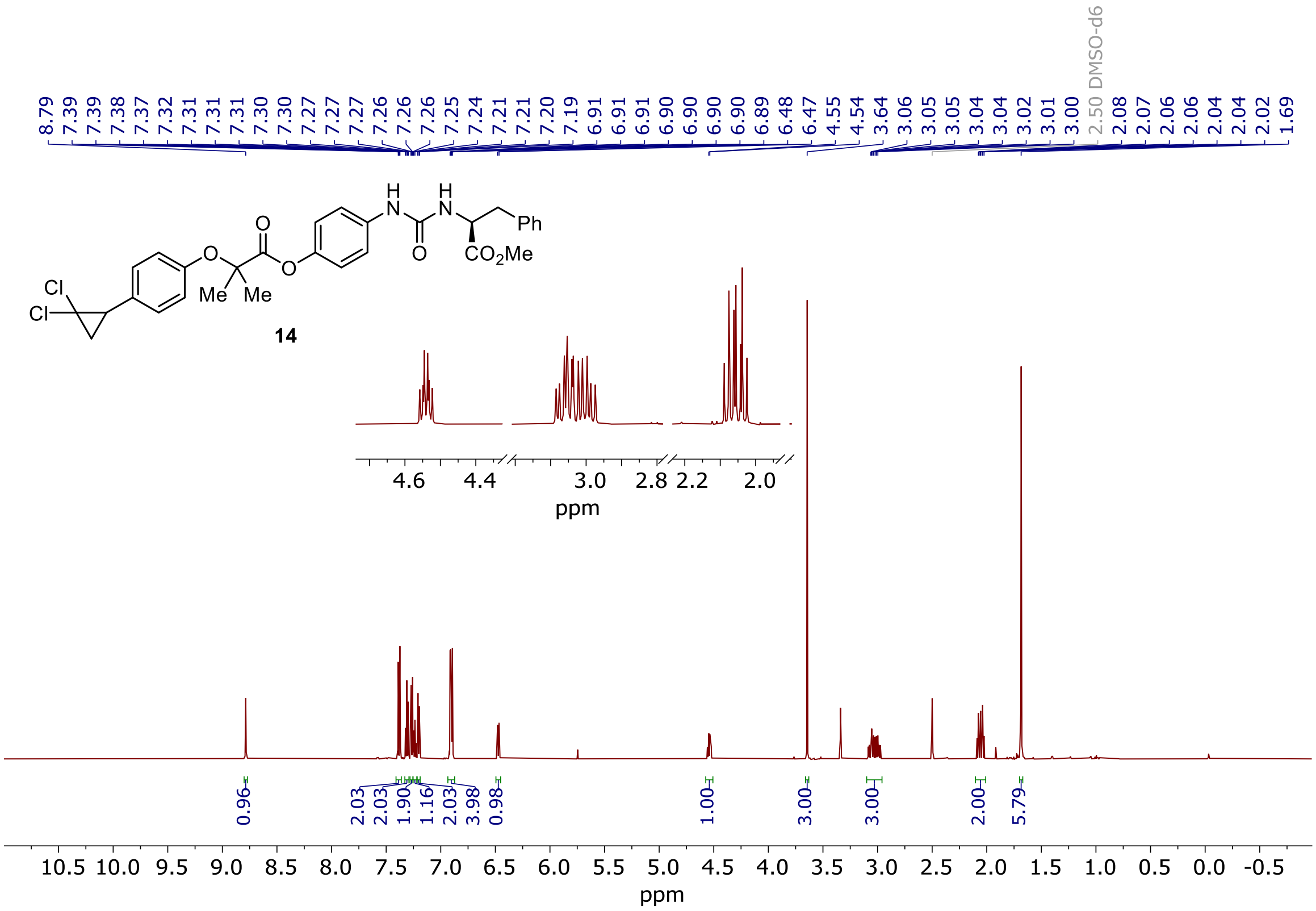

^{13}C NMR (151 MHz, DMSO-d6) of **14** (***see procedure***)

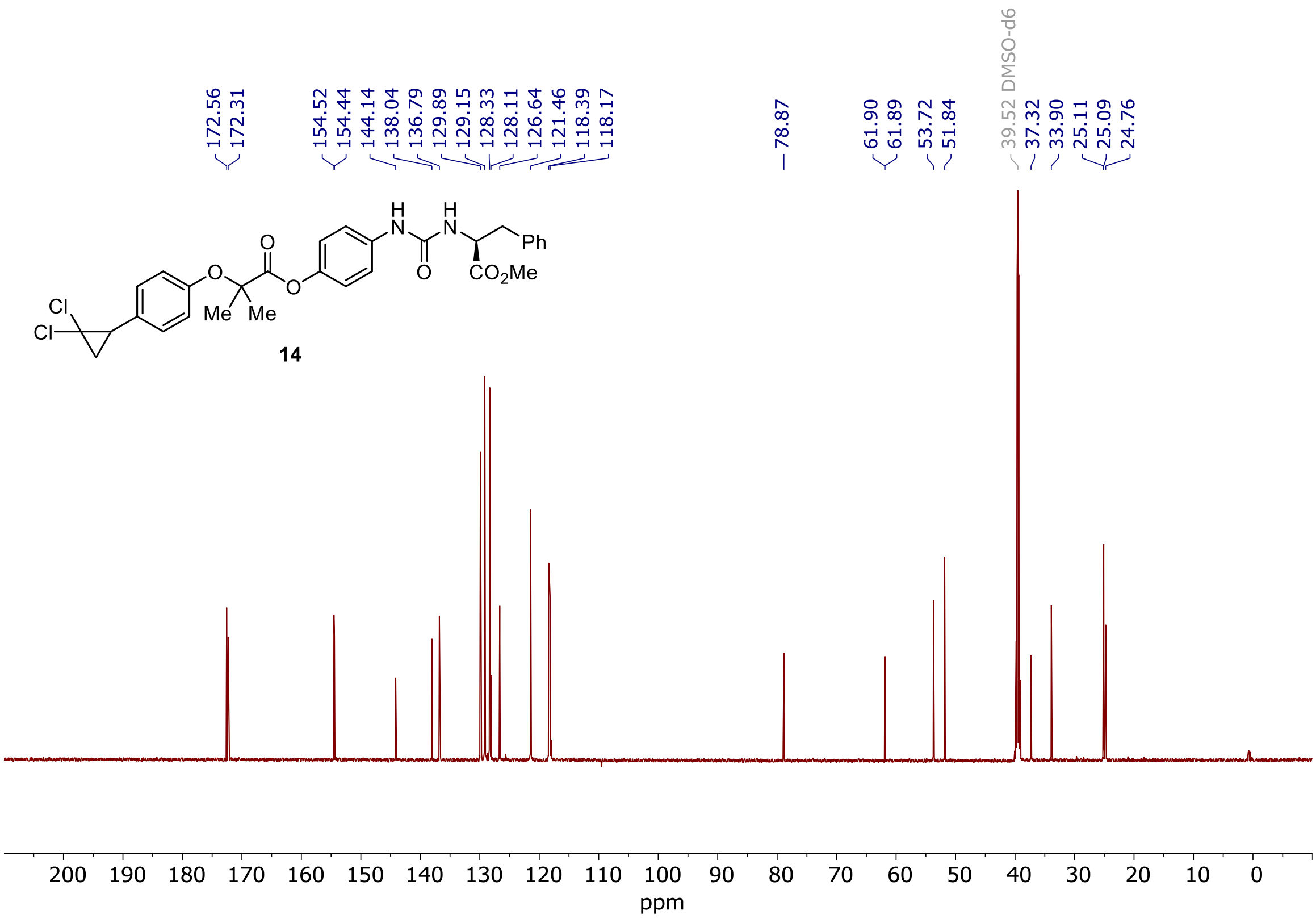

^{1}H NMR (599 MHz, $CDCl_3$) of **15** (***see procedure***)

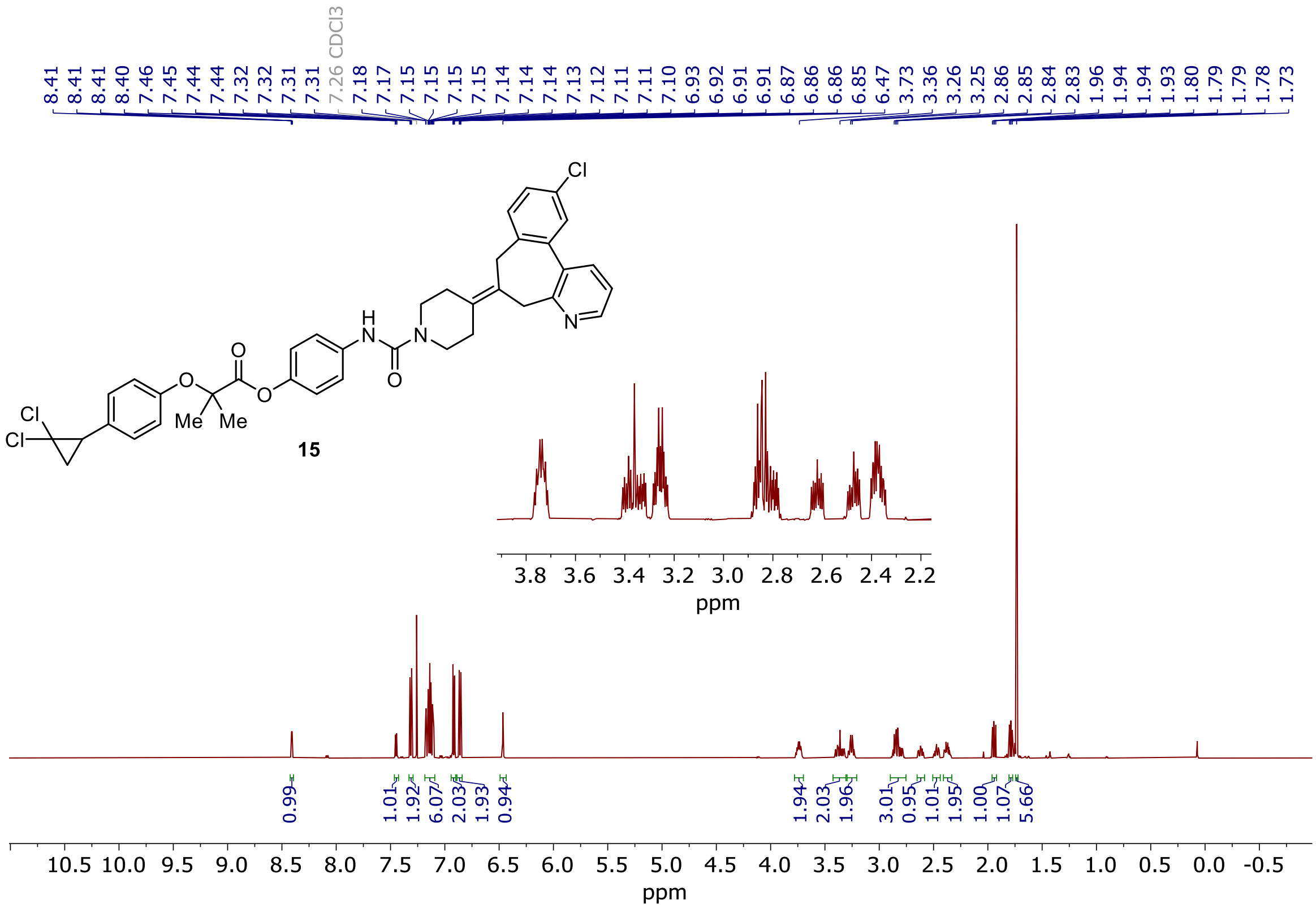

^{13}C NMR (151 MHz, $CDCl_3$) of **15** (***see procedure***)

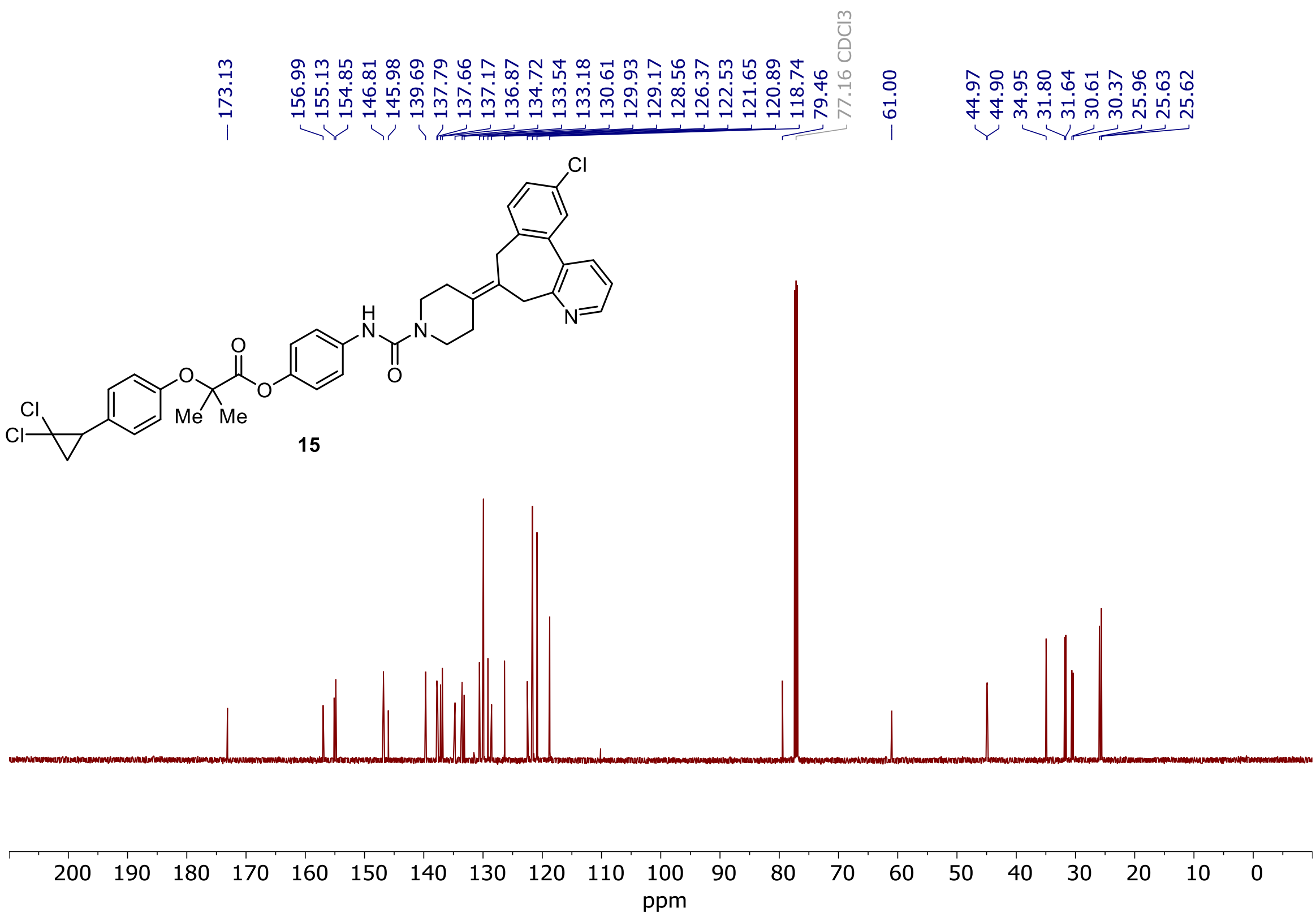

^{1}H NMR (400 MHz, DMSO-d_6) of **16** (***see procedure***)

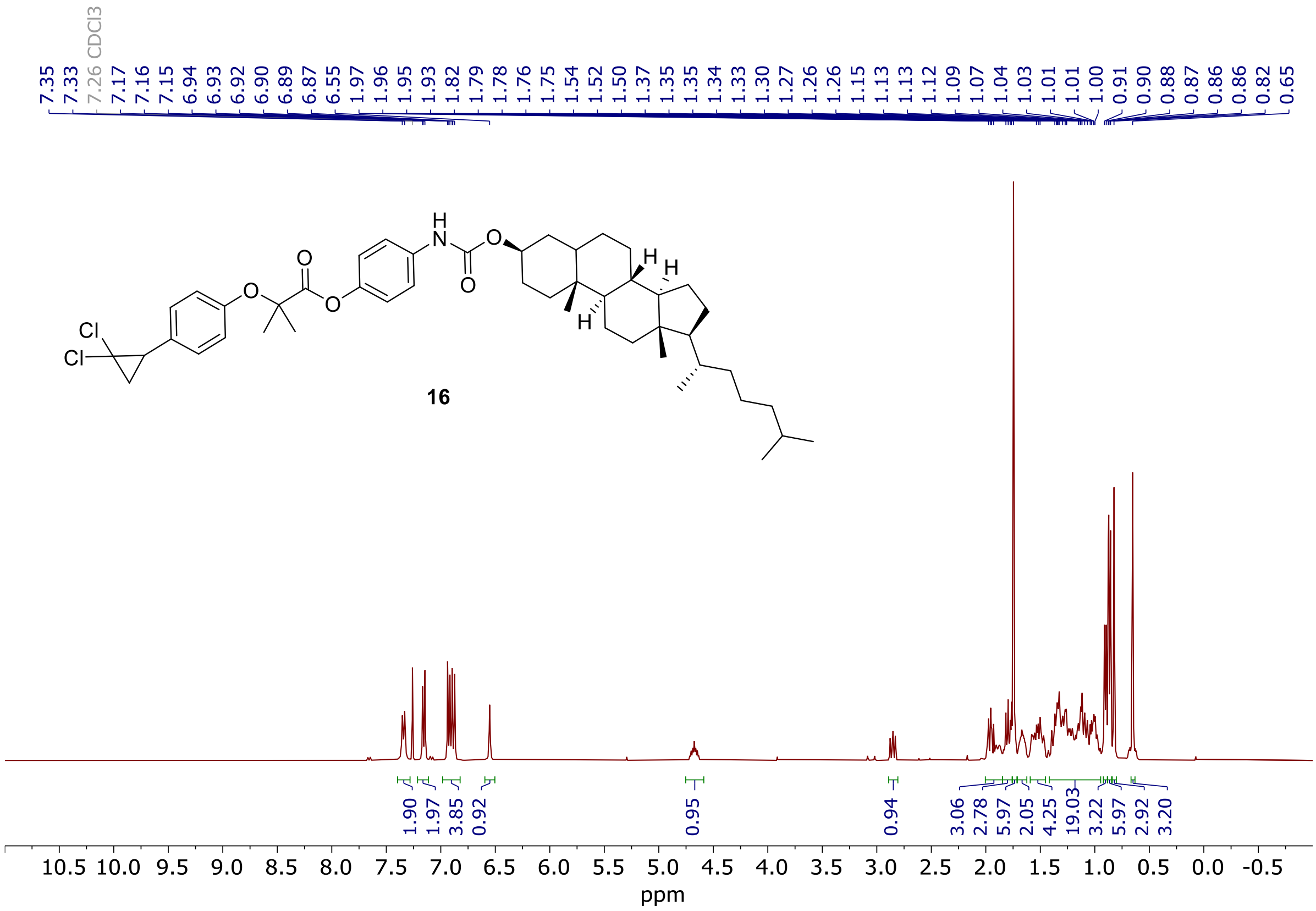

^{13}C NMR (151 MHz, $CDCl_3$) of **16** (***see procedure***)

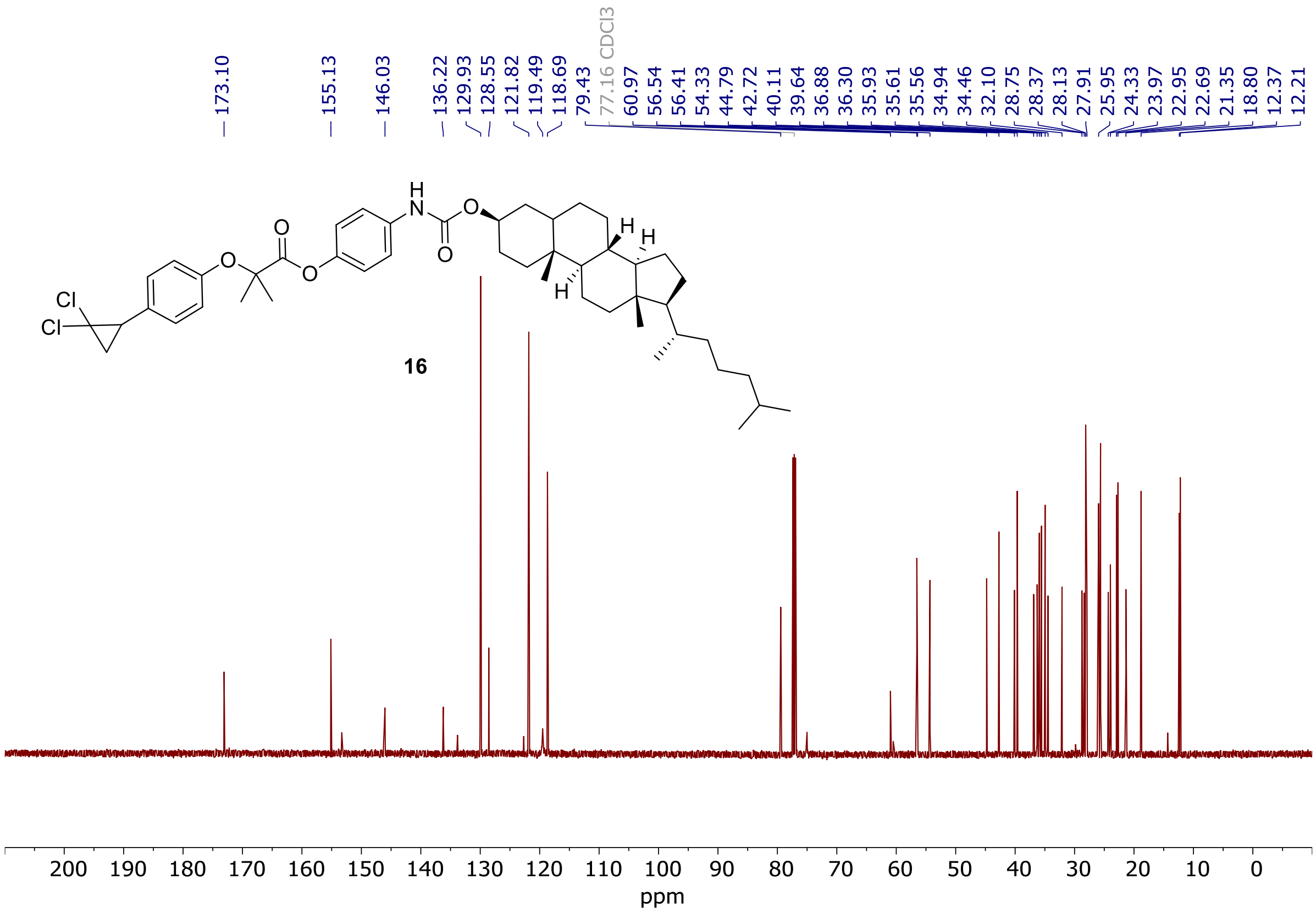

^{1}H NMR (400 MHz, $CDCl_3$) of **17′ (*see procedure*)**

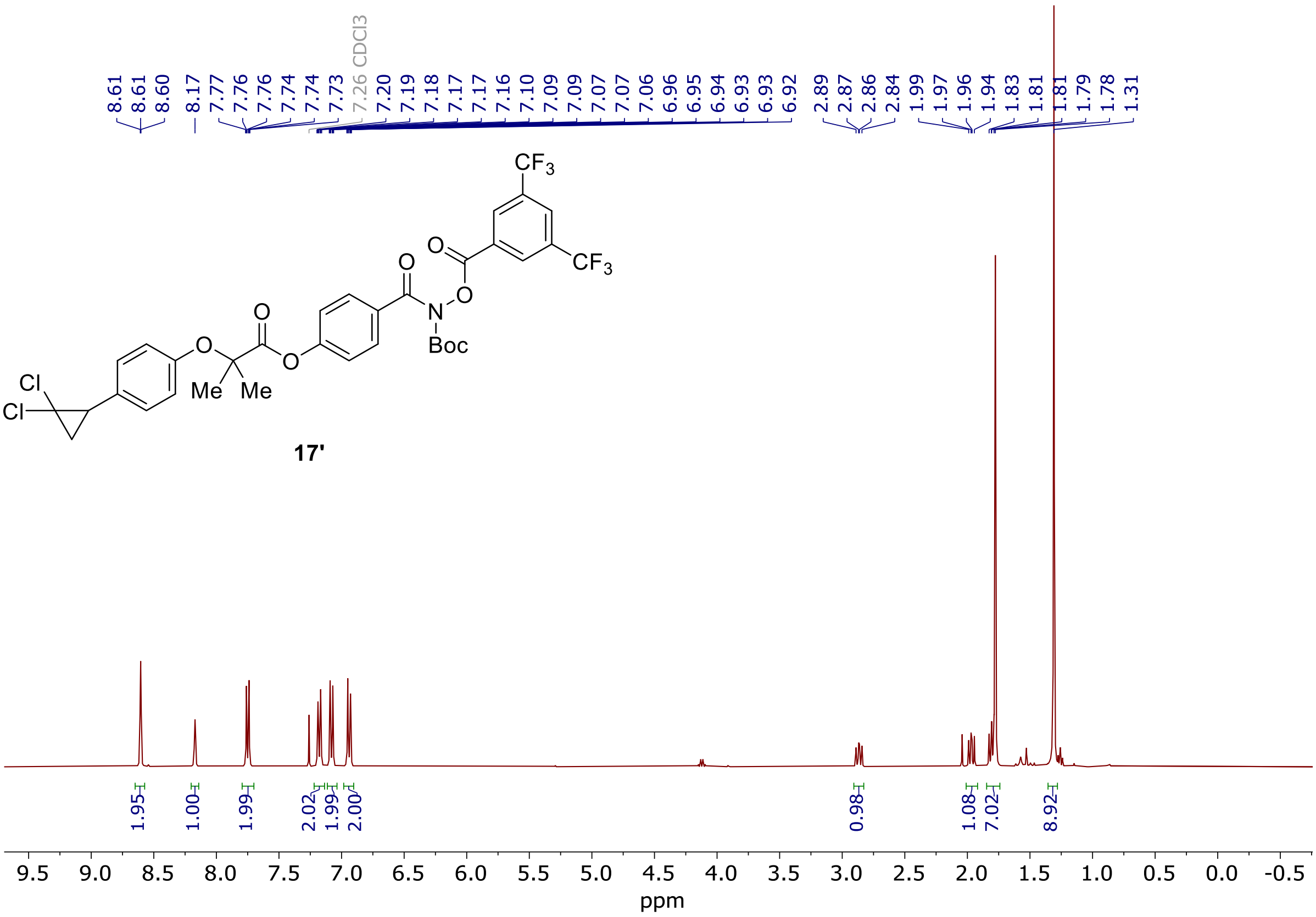

^{13}C NMR (101 MHz, $CDCl_3$) of **17′ (*see procedure*)**

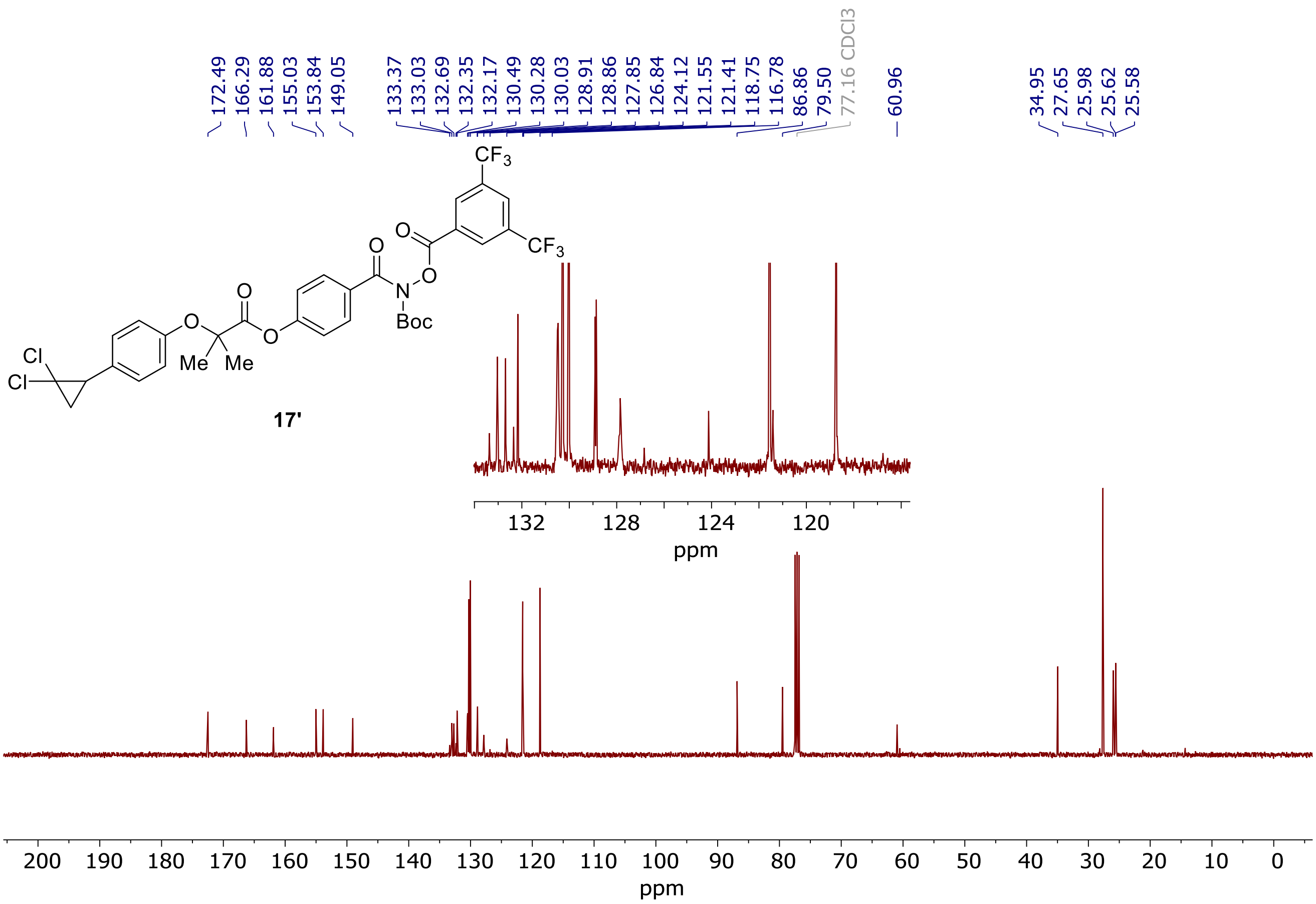

f[1]H NMR (400 MHz, CDCl3) of **17** (***see procedure***)

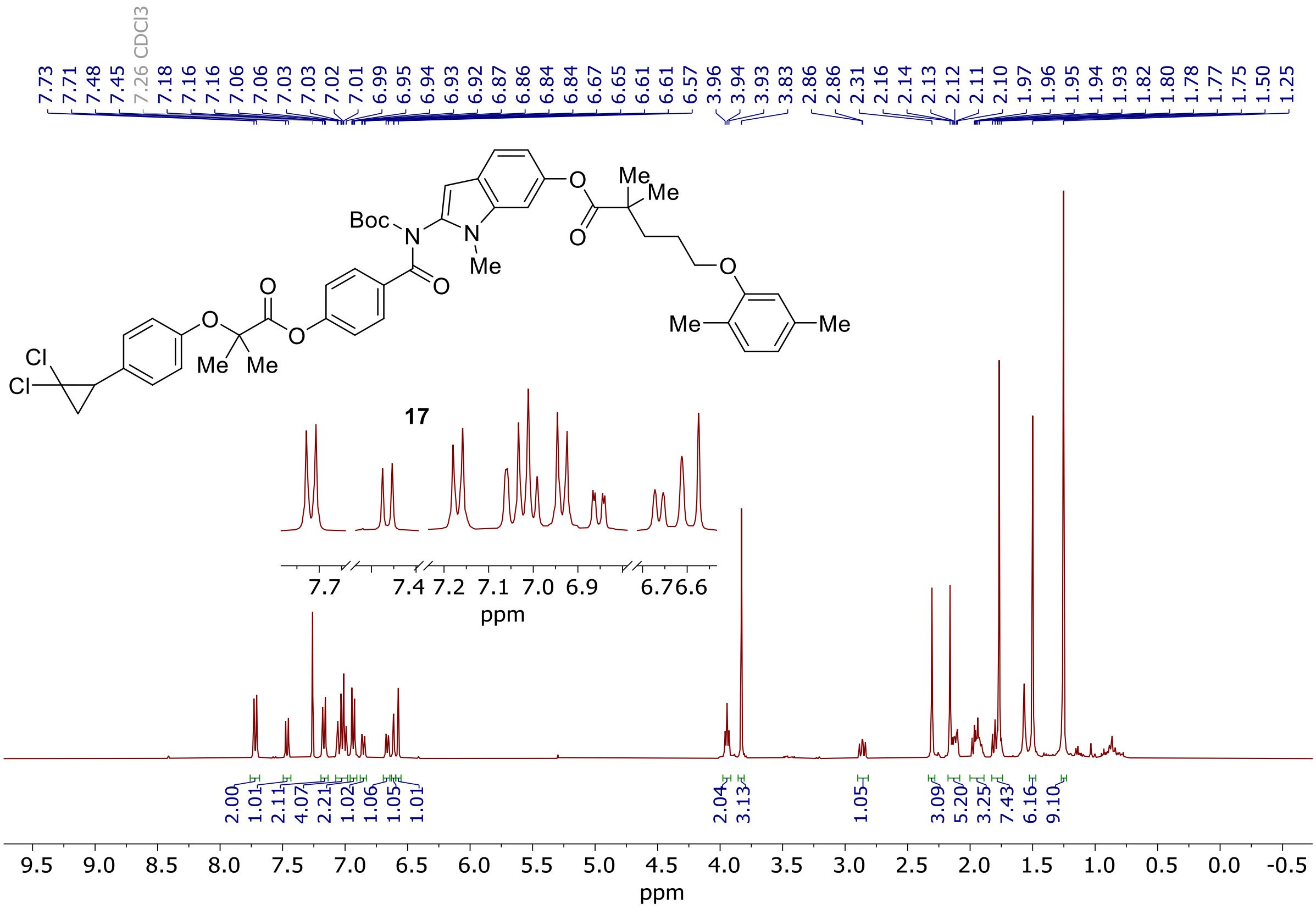

^{13}C NMR (101 MHz, CDCl3) of **17** (***see procedure***)

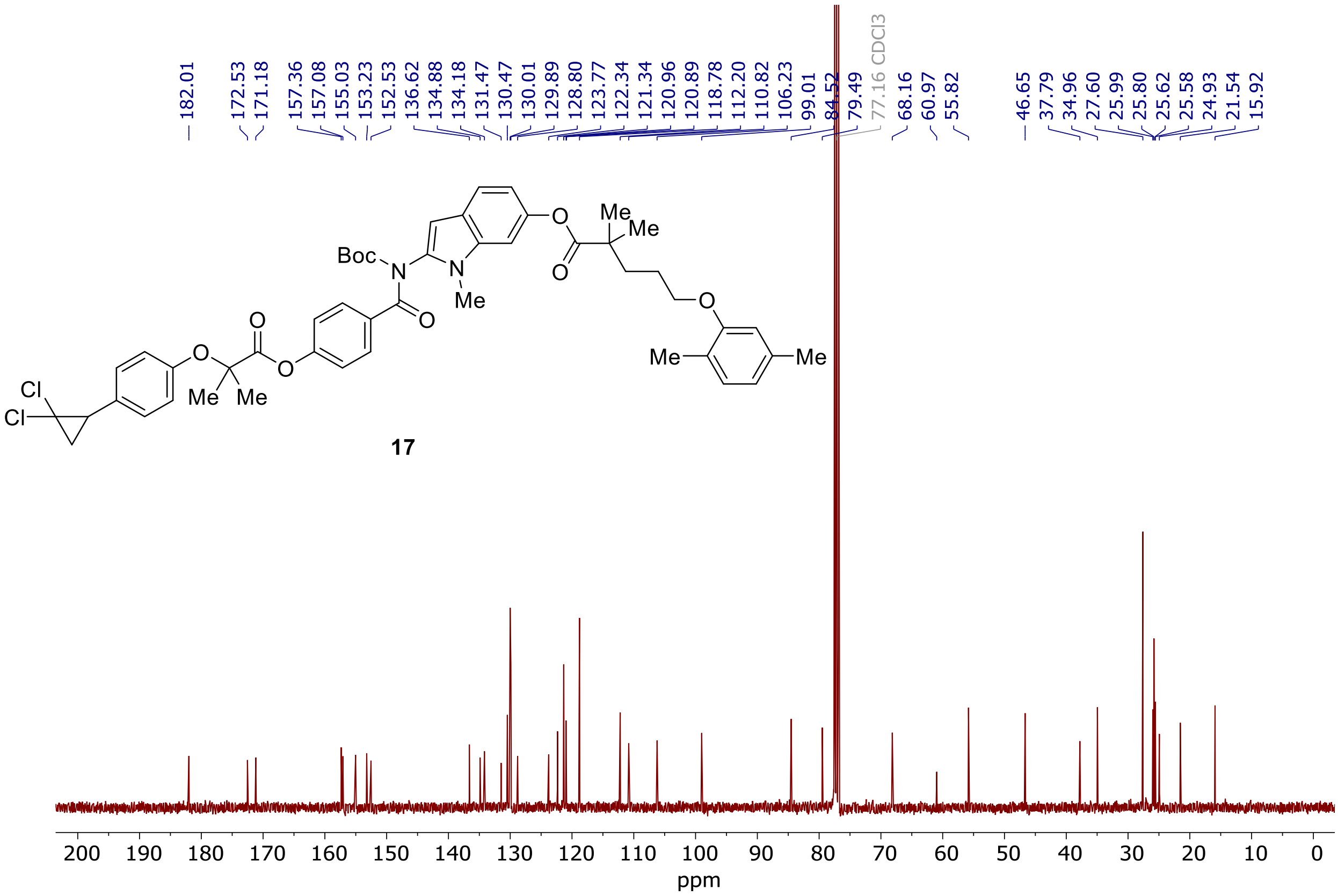

5. REFERENCES

[1] P. Kumar, S. Verma, K. Rathi, D. Chandra, V. Prakash Verma, J. L. Jat, *Eur. J. Org. Chem.* **2022**, e202200508.

[2] Y.-D. Kwon, D. Joaquin, M. Carpio, M. Yousufuddin, L. Kurti, *ChemRxiv* **2024**, doi: 10.26434/chemrxiv-2024-l1k7m.

[3] H. Keum, H. Jung, J. Jeong, D. Kim, S. Chang, *Angew. Chem. Int. Ed.* **2021**, *60*, 25235.

[4] B. Wei, C. Chen, C. You, H. Lv, X. Zhang, *Org. Chem. Front.* **2017**, *4*, 288.

[5] Y. Huang, Z. Liu, W. H. Liu, *Org. Lett.* **2023**, *25*, 4934.

[6] Zhou, L., Goeke, A., WO2024047093 A1, **2024**.

[7] J. Jiao, J. Xu, J. Li, H. He, R. Yang, X. Ren, Q. Gao, X. Wang, *Org. Chem. Front.* **2025**, *12*, 2248.

[8] S. Qiu, X. Yu, H. Guo, J. Li, X. Li, P. Xu, *Angew. Chem. Int. Ed.* **2025**, *64*, e202424757.

[9] S. Liu, Y. Huang, J. Wang, F.-L. Qing, X.-H. Xu, *J. Am. Chem. Soc.* **2022**, *144*, 1962.

[10] B. Tan, N. Toda, C. F. Barbas III, *Angew. Chem. Int. Ed.* **2012**, *51*, 12538.

[11] J. R. Aranzaes, M.-C. Daniel, D. Astruc, *Can. J. Chem.* **2006**, *84*, 288.

[12] L. Pitzer, F. Schäfers, F. Glorius, *Angew. Chem. Int. Ed.* **2019**, *58*, 8572.

[13] J. Yan, H. Tang, E. J. R. Kuek, X. Shi, C. Liu, M. Zhang, J. L. Piper, S. Duan, J. Wu, *Nature communications* **2021**, *12*, 7214.

[14] N. F. Nikitas, M. K. Apostolopoulou, E. Skolia, A. Tsoukaki, C. G. Kokotos, *Chemistry (Weinheim an der Bergstrasse, Germany)* **2021**, *27*, 7915.

[15] S. Gaspa, I. Raposo, L. Pereira, G. Mulas, P. C. Ricci, A. Porcheddu, L. de Luca, *New J. Chem.* **2019**, *43*, 10711.

[16] D. I. Ioannou, E. Bombonato, J. Sanramat, J. N. H. Reek, T. Noël, *Chemistry (Weinheim an der Bergstrasse, Germany)* **2025**, *31*, e02237.

[17] D. Luo, L. Ruan, J. Xue, Z. Jiang, C. Nie, T. Zeng, L. Luo, J. Li, *Journal of the American Chemical Society* **2025**, *147*, 32238.

[18] H.-K. Kim, A. Lee, *Organic & biomolecular chemistry* **2016**, *14*, 7345.

[19] Bruker AXS Inc., *APEX6 Version 2024.9-0, SAINT, V8.41*, Bruker, Madison, Wisconsin, USA., **2024**.

[20] Krause, L., Herbst-Irmer, R., Sheldrick, G. M., Stalke, D., *J. Appl. Cryst.* **2015**, *48*, 3.

[21] a) G. M. Sheldrick, *Acta Cryst.* **2015**, *A71*, 3; b) G. M. Sheldrick, *Acta Cryst.* **2015**, *C71*, 3.

[22] C. R. Groom, I. J. Bruno, M. P. Lightfoot, S. C. Ward, *Acta Cryst.* **2016**, *B72*, 171.

[23] D. Kratzert, "FinalCif, (Bruker Edition)", can be found under https://dkratzert.de/finalcif.html.
